# Twenty-two new species in the genus *Hyphantrophaga* Townsend (Diptera: Tachinidae) from Area de Conservación Guanacaste, with a key to the species of Mesoamerica

**DOI:** 10.3897/BDJ.7.e29553

**Published:** 2019-06-28

**Authors:** AJ Fleming, D. Monty Wood, M. Alex Smith, Tanya Dapkey, Winnie Hallwachs, Daniel Janzen

**Affiliations:** 1 Agriculture Agri-Food Canada, Ottawa, Canada Agriculture Agri-Food Canada Ottawa Canada; 2 University of Guelph, Guelph, Canada University of Guelph Guelph Canada; 3 Department of Biology, University of Pennsylvania, Philadelphia, United States of America Department of Biology, University of Pennsylvania Philadelphia United States of America

**Keywords:** caterpillar, tropical, Goniini, parasitoid, fly, rain forest, dry forest, cloud forest, ACG

## Abstract

**Background:**

We describe 22 new species in the genus *Hyphantrophaga* Townsend, 1892 (Diptera: Tachinidae) from Area de Conservación Guanacaste (ACG) in north-western Costa Rica. All species were reared from an ongoing inventory of wild-caught caterpillars spanning a variety of families (Lepidoptera: Bombycidae, Crambidae, Depressariidae, Doidae, Erebidae, Euteliidae, Gelechiidae, Geometridae, Hedylidae, Hesperiidae, Immidae, Lasiocampidae, Limacodidae, Megalopygidae, Mimaloniidae, Noctuidae, Nolidae, Notodontidae, Nymphalidae, Papilionidae, Pieridae, Phiditiidae, Pterophoridae, Pyralidae, Riodinidae, Saturniidae, Sphingidae, Thyrididae, Tortricidae and Zygaenidae). We provide a morphological description of each species together with information on life history, molecular data and photographic documentation. In addition to the new species, we provide a redescription of the genus, as well as the redescription of three previously described species, which were also collected within ACG during this study: *Hyphantrophaga angustata* (van der Wulp), *Hyphantrophaga myersi* (Aldrich) and *Hyphantrophaga virilis* (Aldrich & Webber).

**New information:**

The following 22 new species of *Hyphantrophaga* are described: *Hyphantrophaga adrianguadamuzi* Fleming & Wood **sp. n.**, *Hyphantrophaga albopilosa* Fleming & Wood **sp. n.**, *Hyphantrophaga anacordobae* Fleming & Wood **sp. n.**, *Hyphantrophaga calixtomoragai* Fleming & Wood **sp. n.**, *Hyphantrophaga calva* Fleming & Wood **sp. n..**, *Hyphantrophaga ciriloumanai* Fleming & Wood **sp. n.**, *Hyphantrophaga danausophaga* Fleming & Wood **sp. n.**, *Hyphantrophaga diniamartinezae* Fleming & Wood **sp. n.**, *Hyphantrophaga duniagarciae* Fleming & Wood **sp. n.**, *Hyphantrophaga edwinapui* Fleming & Wood **sp. n.**, *Hyphantrophaga eldaarayae* Fleming & Wood **sp. n.**, *Hyphantrophaga eliethcantillanoe* Fleming & Wood **sp. n.**, *Hyphantrophaga gilberthampiei* Fleming & Wood **sp. n.**, *Hyphantrophaga guillermopereirai* Fleming & Wood **sp. n.**, *Hyphantrophaga hazelcambroneroae* Fleming & Wood **sp. n.**, *Hyphantrophaga luciariosae* Fleming & Wood **sp. n.**, *Hyphantrophaga manuelriosi* Fleming & Wood **sp. n.**, *Hyphantrophaga morphophaga* Fleming & Wood **sp. n.**, *Hyphantrophaga nigricauda* Fleming & Wood **sp. n.**, *Hyphantrophaga osvaldoespinozai* Fleming & Wood **sp. n.**, *Hyphantrophaga pabloumanai* Fleming & Wood **sp. n.** and *Hyphantrophaga similis* Fleming & Wood **sp. n.**

The following are proposed by Wood as new synonyms of *Hyphantrophaga* Townsend, 1892: *Brachymasicera* Townsend, 1911 **syn. n.**, *Ommasicera* Townsend, 1911 **syn. n.**, *Ophirosturmia* Townsend, 1911 **syn. n.**, *Patillalia* Curran, 1934 **syn. n.** and *Ypophaemyiops* Townsend, 1935 **syn. n.**

The following nine new combinations are proposed as a result of the new synonymies: *Hyphantrophaga adamsoni* (Thompson, 1963), **comb. n.**, *Hyphantrophaga fasciata* (Curran, 1934), **comb. n.**, *Hyphantrophaga glauca* (Giglio-Tos, 1893), **comb. n.**, *Hyphantrophaga gowdeyi* (Curran, 1926), **comb. n.**, *Hyphantrophaga myersi* (Aldrich, 1933), **comb. n.**, *Hyphantrophaga nigripes* (Townsend, 1928), **comb. n.**, *Hyphantrophaga optica* (Schiner, 1868), **comb. n.**, *Hyphantrophaga polita* (Townsend, 1911), **comb. n.**, *Hyphantrophaga subpolita* (Townsend, 1912), **comb. n.**

## Introduction

The New World genus *Hyphantrophaga* (Exoristinae: Goniini) was erected by Townsend (1892). He initially described the type species, *Meigenia hyphantriae* Townsend, 1891, reluctantly placing it in the genus *Meigenia* Robineau-Desvoidy, 1830; one year later (Townsend 1892), after further analysis and clarification, he changed his mind and moved *M. hyphantriae* to the new genus *Hyphantrophaga*. The original description of *M. hyphantriae* was based on four specimens (three females, one male) reared from *Hyphantria cunea* (Drury, 1773) (Lepidoptera: Erebidae) collected during his time at Las Cruces, New Mexico. When Townsend (1892) described the new genus, he realised that what he had initially thought was a male was, in fact, a female. As a result, the new genus *Hyphantrophaga* was based on four female syntypes of which only two remain, deposited in SEMK and USNM, respectively. In the same work, he also added the description of two males collected later, in 1891.

In an afterword to the description, Townsend suggested that *M. hyphantriae* could be differentiated from all other species of *Meigenia* by the lack of discal setae present on all abdominal segments with the exception of T5 and the morphology of the face (two character states useful in differentiating species, but that we now see as variable within the genus). As in *Houghia* Coquillett, 1897, the accurate identification of *Hyphantrophaga* is based on a characteristic “gestalt” that comprises the combination of features within the genus and the fact that the females lay microtype eggs.

Since its original description, the genus has seen many changes. Sellers (1943) made *Hyphantrophaga* a junior synonym of *Zenillia* Robineau-Desvoidy, 1830. Twenty-two years later, Sabrosky and Arnaud (1965) treated the genus as valid (without officially resurrecting it), raising the number of valid species to three. It was not until much later that O’Hara and Wood (1998) officially resurrected the genus, acknowledging some of the changes to the generic limits suggested by Sellers (1943). They also synonimised *Eusisyropa* Townsend, 1908 and increased the number of valid species of *Hyphantrophaga* from three to 15, with 10 of these belonging to the Neotropical fauna.

*Hyphantrophaga* belongs to the tribe Goniini; females of all members of this tribe lay "microtype" eggs directly on the foliage, often in direct proximity to or around the host caterpillar. As the host feeds, the tachinid egg is consumed along with the leaf fragments. Upon hatching, the first instar larva traverses the gut wall and finds its way to a "safe" space within the caterpillar's body, where it remains until it eventually kills its host and continues feeding on the carcass until it pupates. Only very rarely does the larva not kill the caterpillar (e.g. DeVries 1984 recorded a reared adult butterfly from a caterpillar that had already produced viable tachinid larvae).

All of the new species of *Hyphantrophaga* reared from Area de Conservaci ón Guanacaste (ACG) described in this paper are based on differences in external morphology, male terminalia, CO1 (cox1 or cytochrome oxidase 1) gene sequences and on comparison by AJF and DMW with other named species of *Hyphantrophaga* from other regions. It is important to note, however, that these new species are not to be taken as an indication of the total number of species of *Hyphantrophaga*, even in such a small country as Costa Rica. Comparisons of tachinids collected during the ACG inventory with those present in the national collection in the Museo Nacional de Costa Rica (formerly INBIO) show minimal overlap in species, suggesting that the tachinid fauna in other parts of the country is quite different from that of ACG and requires much additional study. Our study provides the descriptions of 22 new species of *Hyphantrophaga*; we also synonymise five genera under *Hyphantrophaga*, leading to nine new combinations of species names under that genus and thereby increasing the total number of species in the genus from 15 to 46. There may also be a small number of apparent species of *Hyphantrophaga* that have been reared by the ACG inventory and which at present can only be distinguished by their gene sequences (henceforth referred to as DNA barcodes) and host records or of which there is insufficient material to make an accurate diagnosis. We have elected to leave such species for potential later description once additional material is available.

The present study is part of a larger group of studies documenting the tachinid species living within ACG (http://www.acguanacaste.ac.cr) and providing names for new species as they are discovered (Fleming et al. 2014b, Fleming et al. 2014a, Fleming et al. 2015a, Fleming et al. 2015c, Fleming et al. 2015b, Fleming et al. 2015d, Fleming et al. 2016a, Fleming et al. 2016b, Fleming et al. 2017). This series of taxonomic papers will represent a baseline for further, detailed ecological and behavioural accounts and studies extending across ACG ecological groups, whole ecosystems and taxonomic assemblages much larger than a genus.

## Materials and methods

### Project aims and rearing intensity

All flies and rearing information described here were collected by the ongoing ACG inventory of the caterpillars, their food plants and their parasitoids, throughout the major ACG terrestrial ecosystems (Smith et al. 2006, Smith et al. 2007, Smith et al. 2008, Janzen et al. 2009, Janzen and Hallwachs 2011, Smith et al. 2009, Smith et al. 2012, Rodriguez et al. 2012, Fleming et al. 2014a, Janzen et al. 2016). The parasitoid rearing methods are described at http://janzen.bio.upenn.edu/caterpillars/methodology/how/parasitoid_husbandry.htm. This inventory has reared more than 750,000+ wild-caught caterpillars since its inception (Janzen et al. 2009, Janzen and Hallwachs 2011, Fernandez-Triana et al. 2014). This effort is continuing to provide an unprecedented amount of data, providing an invaluable tri-trophic image on parasitoid biology including parasitoids, hosts and host plants. All frequencies of parasitisation reported here need to be considered against this background inventory.

The scope of our treatment of the genus *Hyphantrophaga* is limited to those species found in the Mesoamerican region, from the Isthmus of Tehuantepec in southern Mexico to the Darién gap along the southern border of Panama with Colombia. While we took into account all known species in our comparisons and diagnoses of the new species, only the species distributed within this region are included in the key.

### Imaging and dissections

The species accounts and descriptions presented in this paper are complemented by a series of colour photos, used to illustrate the morphological differences and similarities amongst the species. The morphological terminology used follows Cumming and Wood (2009). The characters in our descriptions are presented in order of appearance on the body from the front to the rear and arranged under the headings **Head**, **Thorax**, **Abdomen** and **Male terminalia**. All dissections and photography were carried out following the methods detailed by Fleming et al. (2014a). Measurements and examples of anatomical landmarks discussed herein are illustrated in Fig. 1. Whenever possible, males were selected preferentially as the holotype, since they bear the most differences in external morphology and are thus better for distinguishing the species. Note that, in cases where only one male was available, this was designated as the holotype and was not subjected to dissection.

### Voucher specimen management

The management of voucher specimens has been detailed in previous papers in this series (Fleming et al. 2014a). In brief, caterpillars reared from the ACG inventory receive a unique voucher code in the format yy–SRNP–xxxxx. Parasitoids emerging from a caterpillar receive the same voucher code; when/if they are later individually processed for DNA barcoding, each receives a second, unique voucher code in the format DHJPARxxxxxxx. The associated data for each voucher code are available at: http://janzen.bio.upenn.edu/caterpillars/database.lasso. All associated data and successful barcodes are permanently and publicly deposited in the Barcode of Life Data System (BOLD) (Ratnasingham and Hebert 2007), with a select set of these data also subsequently migrated to GenBank. Each barcoded specimen also receives accession numbers from the Barcode of Life Data System (BOLD) and GenBank, respectively. The dynamic nature of the inventory means that it is continually adding new specimens, which can be found by searching for the genus *Hyphantrophaga* in BOLD.

All inventoried specimens, discussed herein, were collected under Costa Rican government research permits issued to DHJ and the Tachinidae samples were exported under permit by DHJ from Costa Rica to their final depository in the CNC. Tachinid identifications for the inventory are conducted by DHJ in coordination with a) visual inspection of morphology by AJF and DMW, b) DNA barcoding by MAS and BIO and c) databasing and association with host caterpillars by DHJ and WH, through the inventory itself.

The date of capture cited for each specimen is the date of eclosion of the fly and not the date of capture of the caterpillar. Eclosion date is much more representative of the time when that fly species is on the wing than is the time of capture of the parasitised caterpillar. The “collector” is the parataxonomist who found the caterpillar, rather than the person who later retrieved the newly eclosed fly and processed it by freezing, pinning, labelling and oven-drying. The type-series of the newly-described species are housed in the Diptera collection of the Canadian National Collection (CNC).

### Acronyms for Depositories

AMNH American Museum of Natural History, New York, New York, USA

CAS California Academy of Sciences, San Francisco, California, USA

CNC Canadian National Collection of Insects, Arachnids and Nematodes, Ottawa, Canada

MCZ Museum of Comparative Zoology, Harvard University, Cambridge, Massachusetts, USA

MACN Museo Argentino de Ciencias Naturales Bernardino Rivadavia, Buenos Aires, Argentina

MNHN Muséum National d'Histoire Naturelle, Paris, France

NHMUK Natural History Museum, London, United Kingdom (formerly British Museum (Natural History))

NHMW Naturhistorisches Museum Wien, Vienna, Austria

SEMK Snow Entomological Museum, University of Kansas, Lawrence, Kansas, USA

USNM National Museum of Natural History, Washington, D.C., U.S.A. (formerly United States National Museum)

### Interim names for undescribed host species

As in the other papers in this series, our convention for naming undescribed host species follows a standardised, interim naming system used for taxonomic units considered as distinct species and identified by DNA barcodes. Interim names are given in the format "*Eois* Janzen52" or "*Caviria regina*DHJ01", where the "species epithet" is either composed of the name of the taxonomist who identified the species and a number or the name of a species-group followed by a code. This prevents confusion with already described species while maintaining traceability of each undescribed species within the ACG project.

### DNA barcoding

We generated DNA extracts from single legs using a standard glass fibre protocol (Ivanova et al. 2006), using the standard DNA barcode region (5’ cytochrome c oxidase I (COI) gene) for all specimens of ACG *Hyphantrophaga*. The DNA barcodes (658 bp near the 5’ terminus of the COI gene) were amplified using general insect primers, using standard protocols for both production and quality control (Smith et al. 2006, Smith et al. 2007, Smith et al. 2008, Smith et al. 2009, Smith et al. 2012). All DNA sequences, trace files and accessions were deposited in the Barcode of Life Data System (BOLD) (Ratnasingham and Hebert 2007). Metadata (including GenBank accession codes) associated with each sequence can be consulted on BOLD, by using the persistent DOI dx.doi.org/10.5883/DS-ASACGHYP.

In some cases of ecological specialisation displaying only slight (or in one case, no) barcode divergences, we amplified the internal transcribed spacer regions (ITS1 & ITS2) of the ribosomal RNA. Primers for ITS1 occur in conserved protein coding areas (18S and 5.8S). PCR reactions were carried out in 12.5 ml reaction volumes containing: 2.5 mM MgCl_2_, 25 pmol of each primer, 50 mM dNTPs, 10 mM Tris·HCl (pH 8.3), 50 mM KCl, 10-20 ng (1–2 ml) of genomic DNA and 1 unit of Platinum TaqDNA polymerase, using a thermocycling profile of 1 cycle of 2 min at 94°C, 40 cycles of 40 sec at 94°C, 40 sec at 67°C and 2 min at 72°C, with a final step of 5 min at 72°C. We directly sequenced the amplifications using the primers CAS18Fs1 and CAS5p8sB1d (Ji et al. 2003). Amplicons were evaluated using Sequencher version 5.0 (Gene Codes), examined by eye using Bioedit (Hall 1999) and aligned using Muscle (Edgar 2004).

### *Hyphantrophaga morphophaga* species group

Our analysis of the genus *Hyphantrophaga* required us to create a special group to separate a pair of cryptic species, including two of the new species described herein, *Hyphantrophaga morphophaga* **sp. n.** and *Hyphantrophaga danausophaga* **sp. n.** As of the writing of this paper, species in this group are distinguishable only by life history and behaviour. The implications and diagnosis of this special group, termed here the "*Hyphantrophaga morphophaga* species group", will be discussed in the analysis section below.

The species, belonging to this group, share morphological and molecular similarities but differ considerably in their host preference, life history and habitat. Both species share the following combination of character states: pedicel brown to black, concolorous with first flagellomere; thorax hirsute, covered in black setulae throughout; three katepisternal setae; median marginal setae present on all tergites including ST1+2; discal setae absent on all but T5; males with distinctive sex patch; and hind coxa either setose or bare.

## Taxon treatments

### Hyphantrophaga

Townsend, 1892

Hyphantrophaga Townsend, 1892: 247. Type species: *Meigenia hyphantriae* Townsend, 1891, by original designation.
Eusisyropa Townsend, 1908: 97. Type species: *Tachina blanda* Osten Sacken, 1887, by monotypy. Synonymy by O’Hara and Wood 1998
Brachymasicera Townsend, 1911: 133. Type species: *Brachymasicera polita* Townsend, 1911, by original designation. **Syn. n.**
Ommasicera Townsend, 1911: 145. Type species: *Ommasicera chaetosa* Townsend, 1911, by monotypy. **Syn. n.** [Original description of genus based on female reproductive system, full description of the adult was not provided until Townsend 1912: 337.]
Oomasicera . Incorrect subsequent spelling of *Ommasicera* Townsend, 1911 (Guimaraes 1971: 204).
Ophirosturmia Townsend, 1911: 133. Type species: *Ophirosturmia cincta* Townsend, 1911, by original designation. **Syn. n.**
Patillalia Curran, 1934: 459. Type species: *Patillalia fasciata* Curran, 1934, by original designation. **Syn. n.**
Ypophaemyiops Townsend, 1935: 233. Type species: *Prophryno myersi* Aldrich, 1933, by original designation. **Syn. n.**
Hyphantrophaga **Other species included in *Hyphantrophaga* Townsend, 1892**
adamsoni Thompson, 1963: 293 (*Zenillia*). Holotype female (CNC). Type locality: Trinidad, St. Augustine. **Comb. n.**
angustata van der Wulp, 1890: 70 (*Exorista*). Holotype male (NHMUK). Type locality: Mexico, Guerrero, Chilpancingo, 4600 ft.
coquilletti Aldrich & Webber, 1924: 18 (*Zenillia*). Holotype male (USNM). Type locality: USA, Texas, Belfrage.
auratofrontalis Brèthes, 1908: 475 (*Exorista*). Syntypes male and female (MACN). Type locality: Argentina, Buenos Aires.
autographae Sellers, 1943: 23 (*Zenillia*). Holotype male (USNM). Type locality: Cuba, Baraguá.
blanda Osten Sacken, 1887: 162 (Tachina (Exorista)). Holotype female (MCZ). Type locality: unknown (Massachusetts according to Townsend 1941:266.
boarmiae Coquillett, 1897: 95 (*Exorista*). Lectotype female (USNM), by designation of Aldrich and Webber 1924: 39. Type locality: USA, Massachusetts, Cotuit.
hypenae Coquillett *in* Howard 1897: 47 (*Exorista*). *Nomen nudum*
proserpina Williston, 1889: 1919 (*Exorista*, as subspecies of *blanda*). Holotype male (depository unknown). Type locality: unknown.
blandita Coquillett, 1897: 96 (*Exorista*). Holotype female (USNM). Type locality: USA, New Hampshire, Franconia.
blandoides Thompson, 1963: 297 (*Eusisyropa*). Holotype female (CNC). Type locality: Trinidad, Sta. Cruz Valley.
brasiliensis Moreira, 1915: 227 (*Masicera*). Type status unclear, depository unknown. Type locality: Brazil, Rio de Janeiro. ***Nomen dubium***
chaetosa Townsend, 1911: 145 [description based on female reproductive system; full description of adult in Townsend 1912: 337] (*Ommasicera*). Holotype female (USNM). Type locality: Peru, Piura, Valle del Río Chira, Sullana. **Comb. n.**
collina Reinhard, 1944: 68 (*Zenillia*). Holotype male (SEMK). Type locality: USA, Arizona, Chiricahua Mountains.
euchaetiae Sellers, 1943: 13 (*Zenillia*). Holotype male (USNM). Type locality: USA, New York, Clayton.
fasciata Curran, 1934: 469 (*Patillalia*). Holotype female (AMNH). Type locality: Panama, Canal Zone, Patilla Point. **Comb. n.**
glauca Giglio-Tos, 1893: 6 (*Masicera*). Holotype female (MZUT). Type locality: Mexico.
gowdeyi Curran, 1926: 112 (*Zenillia*). Holotype female (AMNH). Type locality: Jamaica, St. Andrew Parish, Cinchona Botanical Gardens (as Hill Gardens). **Comb. n.**
hyphantriae Townsend, 1891: 176 (*Meigenia*). Lectotype male (USNM), by present designation of Wood. Type locality: USA, New Mexico, Las Cruces.
ceratomiae Coquillett, 1897: 101 (*Exorista*). Holotype male (USNM). Type locality: USA, Texas, Fort Worth.
desmiae Sellers, 1943: 16 (*Zenillia*). Holotype male (USNM). Type locality: USA, California, Exeter.
myersi Aldrich, 1933: 173 (*Prophryno*). Holotype male (NHMUK). Type locality: Guyana (as British Guiana), Pakeraima Mts., Upper Ireng River. **Comb. n.**
nigripes Townsend, 1928: 159 (*Brachymasicera*). Holotype female (USNM). Type locality: Peru, Chiclayo, Pomalca. **Comb. n.**
niveifacies Macquart, 1851a: 162 [also Macquart, 1851b: 189] (*Exorista*). 2 syntypes: 1 male, 1 female (MNHN). Type locality: Brazil, Bahia, Salvador (as “Bahia”).
optica Schiner, 1868: 327 (*Exorista*). Holotype female (NHMW). Type locality: Brazil. **Comb. n.**
polita Townsend, 1911: 143 (*Brachymasicera*). Holotype female (USNM). Type locality: Peru, Piura. **Comb. n.**
scolex Reinhard, 1953: 56 (*Zenillia*) Holotype female (CAS). Type locality: USA, California, Los Angeles County, Tanbark Flat.
sellersi Sabrosky, 1983: 254 (*Eusisyropa*). 12 syntypes: 6 males, 6 females (USNM). Type locality: USA, Mississippi, Oxford; [new name for *boarmiae* of authors, not Coquillett, 1897 (Sabrosky 1983); name made available by Sabrosky (1983) in a bibliographic reference to the diagnosis of *boarmiae* in the Sellers' key (Sellers 1943: 6–7) to the species of *Zenillia*].
subpolita Townsend, 1912: 341 (*Brachymasicera*). Holotype female (USNM). Type locality: Peru, Piura. **Comb. n.**
tucumanensis Sellers, 1943: 21 (*Zenillia*). Holotype male (USNM). Type locality: Argentina, Tucuman.
virilis Aldrich & Webber, 1924: 40 (*Zenillia*). Holotype male (USNM). Type locality: USA, Illinois, Chicago [as New York, Rye, in error - see Arnaud 1963: 116.
Hyphantrophaga Meigenia hyphantriae Townsend, 1891Townsend 1891: 176.

#### Description

**Male. Head** (Fig. 1a): vertex 1/4–1/3 of head width; 1–3 reclinate upper orbital setae; ocellar setae proclinate, well-developed, long and arising either beside or behind anterior ocellus; eye haired in all species; parafacial bare; fronto-orbital plate ranging from shiny silver or gold to brownish with a silver sheen and displaying varying degrees of hirsuteness, with setulae not extending below lowest frontal seta; lower margin of face level with vibrissa, thus not visible in profile; facial ridge bare in most species with two notable exceptions, *H. hazelcambroneroae* **sp. n.** and *H. myersi*, in which the facial ridge is setulose; arista ranging from bare to minutely pubescent, usually distinctly thickened on basal 1/4 or 1/5, ranging in colour from orange to dark brown/black. **Thorax**: ranging from bright gold tomentose to dull grey; 2–4 prominent dorsal vittae, which can be thick and unbroken or thin and only scarcely visible under certain angles of light; prosternum setose; proepisternum with 1–5 main setae surrounded by a brush of shorter, weaker, hairlike setulae; postpronotum with 3–6 setae arranged in a triangle; chaetotaxy: acrostichal setae 3–4:3; dorsocentral setae 3–4:3–4 (only three exceptions displaying three postsutural dorsocentral setae: *H. calva* **sp. n.**, *H. fasciata*, *H. hazelcambroneroae* **sp. n.**); intra-alar setae 2–4:3; supra-alar setae 2:3; 2–3 katepisternal setae; scutellum with four pairs of marginal setae (basal, lateral, subapical and apical); basal scutellar setae often longer than or subequal to lateral scutellar setae; subapical setae typically the strongest of the scutellar marginal setae, ranging from slightly curved and medially convergent to parallel, straight or divergent; ranging in length from equal to or longer than basal scutellar setae; apical setae crossed and short, usually 1/4 length of subapical setae, slightly upturned, at a slight upward angle compared to the plane of the rest of the marginal scutellar setae. **Legs**: ranging in ground colour from yellow to black; hind coxa bare or setose, with a single seta along dorsal margin (Fig. 1b) (this character is sexually dimorphic within *H. calva* **sp. n.** and variable in both sexes of *H. proxima* **sp. n.** and *H. vicina* **sp. n.**). **Wings**: pale translucent, not strongly infuscate; vein R_4+5_ setose, with only 2–3 setulae at base. **Abdomen** (Fig. 1c, d): ground colour ranging from black to different tonalities of brown to orange; mid-dorsal depression on ST1+2 ranging from reaching halfway across the syntergite to almost reaching the hind margin; median marginal setae present on T3 and T4 and often on ST1+2; median discal setae most often confined to T5, but this varies amongst species, with some species displaying median discal setae on T3, T4 and T5; the presence of a sex patch is variable amongst species, ranging from absent to present anywhere between T3–T5. **Terminalia** (Fig. 1e, f): sternite 5 with a deeply excavated median cleft along posterior edge, smoothly U-shaped, with margins covered in dense tomentum; posterior lobes rounded apically, either bare with multiple fine hairlike setulae or with 2–3 strong setae surrounded by many shorter, weaker setulae. Anterior plate of sternite 5 subequal to or longer than posterior lobes; unsclerotised "window" on anterior plate of sternite 5 ranging from absent to almost entirely transparent, directly basal to posterior lobes; the shape of the window, as well as its presence, varies between species. Cerci in posterior view variable between species, ranging from rectangular, digitiform, to triangular; either longer than or only slightly shorter than surstyli; blunt and rounded at apex to apically pointed, either completely separate medially to fused along most of their length; in lateral view often with a strong downward curve at apex, giving it a clubbed appearance; densely setulose along basal 2/3, ventrally setose along entire length. Surstylus in lateral view almost parallel sided along its length, sometimes ending in a slightly downcurved apex, making the structure appear bladelike; when viewed dorsally, the surstyli range from being slightly divergent to slightly convergent or with inward-curved apices, but never strongly convergent. Pregonite usually broad, well-developed, apically squared off or rounded, usually blunt, typically devoid of setulae. Postgonite slightly narrowed, up to 1/3 as wide as pregonite, sharply pointed and curved at apex, typically short and scythelike, with few exceptions in which the postgonite is subequal in length to the pregonite. Epiphallus well-developed and apically hooked. Distiphallus broadly cone-shaped (in some species this cone or flare is much more pronounced, in others it is square or barrel-shaped), with a slender median longitudinal sclerotised reinforcement on its posterior surface and a broad, anterolateral sclerotised acrophallus on anterior surface near apex.

**Female.** As male except in the following traits: **head** with two pairs of proclinate orbital setae. **Abdomen** slightly more globose than in male; T5 folded over into a narrow slit, a trait stereotypical of the tribe Goniini. In cases where sexual dimorphism was observed, the differing character states are mentioned in the species descriptions.

#### Diagnosis

*Hyphantrophaga*, as all other Goniini, is difficult to characterise to tribe based on morphological character states and can only be reliably ascribed to this tribe (*sensu* Herting 1984) based on its microtype ovipary. However, *Hyphantrophaga* does possess a combination of traits that can be considered stereotypical of the group: prosternum setose; males of all species with two pairs of well-developed reclinate upper orbital setae, proclinate orbital setae only present in females (a character state that distinguishes them from males of *Houghia* Coquillet and *Carcelia* Robineau-Desvoidy, in which orbital setae are absent or proclinate); the ocellar setae are always proclinate and in most species arise beside the anterior ocellus; the parafacial, katepimeron and the upper half or more of the facial ridge, are bare; 3–4 well-developed and evenly-spaced postsutural supra-alar setae, the anteriormost being stouter than the first postsutural dorsocentral seta; median discal setae present only on abdominal T5 (in most species); the three major setae of the postpronotum are arranged in a triangle; wings lacking costal spine; hind coxa can be setose or bare (in some cases this character state can be sexually variable within species). The height of the gena is about 1/5 to 1/10 the height of the head, which approaches the condition found in some *Houghia* and differentiates *Hyphantrophaga* from the members of *Carcelia*. The eyes of all species of *Hyphantrophaga* are haired; however, in a few species, the ommatrichia can be short and sparse. The 22 species of *Hyphantrophaga* described herein can be identified to genus using the keys in both Wood 1987 and Wood and Zumbado 2010.

#### Distribution

Ubiquitous throughout the New World, inhabiting a wide variety of ecosystems from south-eastern Canada and the north-eastern USA, west to California and south to Argentina and Brazil.

#### Ecology

Within the ACG inventory, *Hyphantrophaga* has been reared from a wide variety of Lepidoptera hosts throughout the diverse ecosystems of the research area, including: Bombycidae, Crambidae, Depressariidae, Doidae, Erebidae, Euteliidae, Gelechiidae, Geometridae, Hedylidae, Hesperiidae, Immidae, Lasiocampidae, Limacodidae, Megalopygidae, Mimaloniidae, Noctuidae, Nolidae, Notodontidae, Nymphalidae, Papilionidae, Pieridae, Pterophoridae, Pyralidae, Riodinidae, Saturniidae, Sphingidae, Thyrididae, Tortricidae and Zygaenidae.

#### Taxon discussion

*Hyphantrophaga brasiliensis* (Moreira, 1915) is treated as a *nomen dubium* within *Hyphantrophaga*, as it has proven impossible to ascertain the repository or even the existence of the type material.

### Hyphantrophaga adrianguadamuzi

Fleming & Wood sp. n.

urn:lsid:zoobank.org:act:AC7B0563-B5FD-40F4-924B-D187838172B4

#### Materials

- **Type status:** Holotype. **Occurrence:** occurrenceDetails: http://janzen.sas.upenn.edu; catalogNumber: DHJPAR0007297; recordedBy: D.H. Janzen, W. Hallwachs, & Ruth Franco; individualID: DHJPAR0007297; individualCount: 1; sex: male; lifeStage: adult; preparations: pinned; otherCatalogNumbers: ASTAT069-06 ,04-SRNP-13432, BOLD:ABZ0529; **Taxon:** scientificName: Hyphantrophaga adrianguadamuzi; phylum: Arthropoda; class: Insecta; order: Diptera; family: Tachinidae; genus: Hyphantrophaga; specificEpithet: adrianguadamuzi; scientificNameAuthorship: Fleming & Wood, 2018; **Location:** continent: Central America; country: Costa Rica; countryCode: CR; stateProvince: Guanacaste; county: Sector Santa Rosa; locality: Area de Conservacion Guanacaste; verbatimLocality: Area Administrativa; verbatimElevation: 295; verbatimLatitude: 10.8376; verbatimLongitude: -85.6187; verbatimCoordinateSystem: Decimal; decimalLatitude: 10.8376; decimalLongitude: -85.6187; **Identification:** identifiedBy: AJ Fleming; dateIdentified: 2017; **Event:** samplingProtocol: Reared from the larva of the Pyralidae, Paridnea holophaealisDHJ02; verbatimEventDate: 07-Sep-2004; **Record Level:** language: en; institutionCode: CNC; collectionCode: Insects; basisOfRecord: Pinned Specimen
- **Type status:** Paratype. **Occurrence:** occurrenceDetails: http://janzen.sas.upenn.edu; catalogNumber: DHJPAR0037527; recordedBy: D.H. Janzen, W. Hallwachs, & Guillermo Pereira; individualID: DHJPAR0037527; individualCount: 1; sex: male; lifeStage: adult; preparations: pinned; otherCatalogNumbers: ASHYC4272-10 ,09-SRNP-14043, BOLD:ABZ0529; **Taxon:** scientificName: Hyphantrophaga adrianguadamuzi; phylum: Arthropoda; class: Insecta; order: Diptera; family: Tachinidae; genus: Hyphantrophaga; specificEpithet: adrianguadamuzi; scientificNameAuthorship: Fleming & Wood, 2018; **Location:** continent: Central America; country: Costa Rica; countryCode: CR; stateProvince: Guanacaste; county: Sector Santa Rosa; locality: Area de Conservacion Guanacaste; verbatimLocality: Camino Borrachos; verbatimElevation: 295; verbatimLatitude: 10.8429; verbatimLongitude: -85.6161; verbatimCoordinateSystem: Decimal; decimalLatitude: 10.8429; decimalLongitude: -85.6161; **Identification:** identifiedBy: AJ Fleming; dateIdentified: 2017; **Event:** samplingProtocol: Reared from the larva of the Pyralidae, Paridnea holophaealis; verbatimEventDate: 06-Aug-2009; **Record Level:** language: en; institutionCode: CNC; collectionCode: Insects; basisOfRecord: Pinned Specimen
- **Type status:** Paratype. **Occurrence:** occurrenceDetails: http://janzen.sas.upenn.edu; catalogNumber: DHJPAR0035662; recordedBy: D.H. Janzen, W. Hallwachs, & Jose Cortez; individualID: DHJPAR0035662; individualCount: 1; sex: male; lifeStage: adult; preparations: pinned; otherCatalogNumbers: ASHYD1043-09 ,09-SRNP-56575, BOLD:ABZ0529; **Taxon:** scientificName: Hyphantrophaga adrianguadamuzi; phylum: Arthropoda; class: Insecta; order: Diptera; family: Tachinidae; genus: Hyphantrophaga; specificEpithet: adrianguadamuzi; scientificNameAuthorship: Fleming & Wood, 2018; **Location:** continent: Central America; country: Costa Rica; countryCode: CR; stateProvince: Guanacaste; county: Sector Mundo Nuevo; locality: Area de Conservacion Guanacaste; verbatimLocality: Camino Pozo Tres; verbatimElevation: 733; verbatimLatitude: 10.7708; verbatimLongitude: -85.3742; verbatimCoordinateSystem: Decimal; decimalLatitude: 10.7708; decimalLongitude: -85.3742; **Identification:** identifiedBy: AJ Fleming; dateIdentified: 2017; **Event:** samplingProtocol: Reared from the larva of the Pyralidae, Paridnea holophaealis; verbatimEventDate: 16-Aug-2009; **Record Level:** language: en; institutionCode: CNC; collectionCode: Insects; basisOfRecord: Pinned Specimen
- **Type status:** Paratype. **Occurrence:** occurrenceDetails: http://janzen.sas.upenn.edu; catalogNumber: DHJPAR0035660; recordedBy: D.H. Janzen, W. Hallwachs, & Johan Vargas; individualID: DHJPAR0035660; individualCount: 1; sex: female; lifeStage: adult; preparations: pinned; otherCatalogNumbers: ASHYD1041-09 ,09-SRNP-14511, BOLD:ABZ0529; **Taxon:** scientificName: Hyphantrophaga adrianguadamuzi; phylum: Arthropoda; class: Insecta; order: Diptera; family: Tachinidae; genus: Hyphantrophaga; specificEpithet: adrianguadamuzi; scientificNameAuthorship: Fleming & Wood, 2018; **Location:** continent: Central America; country: Costa Rica; countryCode: CR; stateProvince: Guanacaste; county: Sector Santa Rosa; locality: Area de Conservacion Guanacaste; verbatimLocality: Area Administrativa; verbatimElevation: 295; verbatimLatitude: 10.8376; verbatimLongitude: -85.6187; verbatimCoordinateSystem: Decimal; decimalLatitude: 10.8376; decimalLongitude: -85.6187; **Identification:** identifiedBy: AJ Fleming; dateIdentified: 2017; **Event:** samplingProtocol: Reared from the larva of the Pyralidae, Paridnea holophaealis; verbatimEventDate: 29-Aug-2009; **Record Level:** language: en; institutionCode: CNC; collectionCode: Insects; basisOfRecord: Pinned Specimen
- **Type status:** Paratype. **Occurrence:** occurrenceDetails: http://janzen.sas.upenn.edu; catalogNumber: DHJPAR0046407; recordedBy: D.H. Janzen, W. Hallwachs, & Guillermo Pereira; individualID: DHJPAR0046407; individualCount: 1; sex: female; lifeStage: adult; preparations: pinned; otherCatalogNumbers: ACGBA580-12 ,11-SRNP-14585, BOLD:ABZ0529; **Taxon:** scientificName: Hyphantrophaga adrianguadamuzi; phylum: Arthropoda; class: Insecta; order: Diptera; family: Tachinidae; genus: Hyphantrophaga; specificEpithet: adrianguadamuzi; scientificNameAuthorship: Fleming & Wood, 2018; **Location:** continent: Central America; country: Costa Rica; countryCode: CR; stateProvince: Guanacaste; county: Sector Santa Rosa; locality: Area de Conservacion Guanacaste; verbatimLocality: Area Administrativa; verbatimElevation: 295; verbatimLatitude: 10.8376; verbatimLongitude: -85.6187; verbatimCoordinateSystem: Decimal; decimalLatitude: 10.8376; decimalLongitude: -85.6187; **Identification:** identifiedBy: AJ Fleming; dateIdentified: 2017; **Event:** samplingProtocol: Reared from the larva of the Pyralidae, Paridnea holophaealis; verbatimEventDate: 24-Nov-2011; **Record Level:** language: en; institutionCode: CNC; collectionCode: Insects; basisOfRecord: Pinned Specimen

#### Description

**Male** (Fig. 2). Length: 7–8 mm. **Head** (Fig. 2b): vertex 1/4 of head width; two reclinate upper orbital setae; ocellar setae arising behind anterior ocellus; ocellar triangle with a very slight gold tinge compared to rest of fronto-orbital plate; fronto-orbital plate shiny silver and setulose, setulae not extending below lowest frontal seta; parafacial bare and shiny silver; facial ridge bare; eye densely haired; pedicel black, concolorous with postpedicel; arista distinctly thickened on basal 1/3–1/4; palpus light brown basally, apically orange, haired throughout. **Thorax** (Fig. 2a, c): brassy tomentose dorsally, contrasting with silver grey laterally; four thin yet prominent dorsal vittae, outermost two broken across suture, innermost pair unbroken, not reaching beyond 2nd postsutural dorsocentral seta; postpronotum with three setae arranged in a triangle; chaetotaxy: acrostichal setae 3:3; dorsocentral setae 3:4; intra-alar setae 2:3; supra-alar setae 2:3; two katepisternal setae; basal scutellar setae subequal in length to lateral scutellar setae; subapical scutellar setae strongest and longest of marginal scutellar setae, strongly divergent; apical scutellar setae 1/3 of length of basal scutellar setae crossed apically; one pair of discal scutellar setae set as widely apart as subapical scutellar setae; scutellum concolorous with scutum. **Legs** (Fig. 2c): black in ground colour with dense covering of black hairs, making them appear darker; fore femur with dense silver tomentum on posterodorsal surface; hind coxa setose. **Wing** (Fig. 2a): hyaline, very slightly infuscate at base; vein R_4+5_ with two setulae at base. **Abdomen** (Fig. 2a, c): ground colour light brown; middorsal depression on ST1+2 not reaching hind margin; median marginal setae present on ST1+2–T3; a complete row of marginal setae present on T4 and T5; discal setae present on T3–T5; sex patch absent; distinct tomentose bands along anterior 4/5 of T3–T5. **Terminalia** (Fig. 2d, e, f): anterior edge of sternite 5 (Fig. 2f) with a deeply curved medial depression, posterior margin with a deep median cleft, smoothly U-shaped. Anterior plate of sternite 5 subequal to length of apical lobes; unsclerotised "window" ovoid to slightly rectangular, as wide as median cleft. Lateral lobe of sternite slightly pointed apically and with many short setae of equal length. Anterior plate of sternite 5 shorter than apical lobes. Cerci in posterior view (Fig. 2d) triangular with external edges inwardly wedged, slightly shorter than surstyli, blunt and rounded off towards apex, completely separate medially, diverging slightly at tips; in lateral view with a strong, almost 90 degree bend 1/5 along their length. Surstylus in lateral view (Fig. 2e) almost parallel-sided along its length, ending in a slightly downcurved apex, making the structure appear fingerlike; basal half twice as wide as apical half; surstylus appearing fused with epandrium. Surstyli when viewed dorsally strongly divergent and wide open. Pregonite well-developed, 1/2 as long as distiphallus, squared off, ending in a trumpet-like, flared apex. Postgonite elongate and slender, horn-shaped, subequal in length to pregonite, slightly narrowed towards apex, not strongly curved. Distiphallus sail-shaped, apically flared, with a slender median longitudinal sclerotised reinforcement on its posterior surface and a broad, anterolateral, sclerotised acrophallus on each side, joining the plate of opposite side on anterior surface near apex.

**Female**. Length: 6–9 mm. As male, differing only by the presence of two pairs of proclinate orbital setae.

#### Diagnosis

*Hyphantrophaga adrianguadamuzi* **sp. n.** can be distinguished from all other *Hyphantrophaga* species by the following combination of traits: pedicel black (concolorous with postpedicel), palpus brown basally and orange apically, two katepisternal setae, hind coxa setose, colouration of T5 matching rest of tergites, median marginal setae absent on ST1+2 and discal setae present on T3–T5.

#### Etymology

*Hyphantrophaga adrianguadamuzi* **sp. n.** is named in recognition of Adrian Guadamuz Chavarría's dedication and work in finding and rearing the ACG caterpillars that contained tachinid larvae.

#### Distribution

Costa Rica, ACG, Guanacaste Province, 295–733 m elevation.

#### Ecology

*Hyphantrophaga adrianguadamuzi* **sp. n.** has been reared five times from one species of Lepidoptera in the family Pyralidae, *Paridnea holophealis* Ragonot, 1892, in dry forest and dry-rain lowland intergrade.

### Hyphantrophaga albopilosa

Fleming & Wood sp. n.

urn:lsid:zoobank.org:act:1BCF3C29-CCCF-4AF3-9372-6BB7FA910C87

#### Materials

- **Type status:** Holotype. **Occurrence:** occurrenceDetails: http://janzen.sas.upenn.edu; catalogNumber: DHJPAR0007442; recordedBy: D.H. Janzen, W. Hallwachs & Manuel Rios; individualID: DHJPAR0007442; individualCount: 1; sex: male; lifeStage: adult; preparations: pinned; otherCatalogNumbers: ASTAT214-06, 04-SRNP-34031, BOLD:AAF3886; **Taxon:** scientificName: Hyphantrophaga albopilosa; phylum: Arthropoda; class: Insecta; order: Diptera; family: Tachinidae; genus: Hyphantrophaga; specificEpithet: albopilosa; scientificNameAuthorship: Fleming & Wood, 2018; **Location:** continent: Central America; country: Costa Rica; countryCode: CR; stateProvince: Guanacaste; county: Sector Pitilla; locality: Area de Conservacion Guanacaste; verbatimLocality: Sendero Laguna; verbatimElevation: 680; verbatimLatitude: 10.9888; verbatimLongitude: -85.4234; verbatimCoordinateSystem: Decimal; decimalLatitude: 10.9888; decimalLongitude: -85.4234; **Identification:** identifiedBy: AJ Fleming; dateIdentified: 2017; **Event:** samplingProtocol: Reared from the larva of the Sphingidae, Adhemarius ypsilon; verbatimEventDate: 18-Aug-2004; **Record Level:** language: en; institutionCode: CNC; collectionCode: Insects; basisOfRecord: Pinned Specimen
- **Type status:** Paratype. **Occurrence:** occurrenceDetails: http://janzen.sas.upenn.edu; catalogNumber: DHJPAR0054045; recordedBy: D.H. Janzen, W. Hallwachs & Elda Araya; individualID: DHJPAR0054045; individualCount: 1; sex: female; lifeStage: adult; preparations: pinned; otherCatalogNumbers: ASHYD3213-14, 13-SRNP-7080, BOLD:AAF3886; **Taxon:** scientificName: Hyphantrophaga albopilosa; phylum: Arthropoda; class: Insecta; order: Diptera; family: Tachinidae; genus: Hyphantrophaga; specificEpithet: albopilosa; scientificNameAuthorship: Fleming & Wood, 2018; **Location:** continent: Central America; country: Costa Rica; countryCode: CR; stateProvince: Alajuela; county: Sector San Cristobal; locality: Area de Conservacion Guanacaste; verbatimLocality: Finca San Gabriel; verbatimElevation: 645; verbatimLatitude: 10.8777; verbatimLongitude: -85.3934; verbatimCoordinateSystem: Decimal; decimalLatitude: 10.8777; decimalLongitude: -85.3934; **Identification:** identifiedBy: AJ Fleming; dateIdentified: 2017; **Event:** samplingProtocol: Reared from the larva of the Sphingidae, Adhemarius ypsilon; verbatimEventDate: 12-Jan-2014; **Record Level:** language: en; institutionCode: CNC; collectionCode: Insects; basisOfRecord: Pinned Specimen
- **Type status:** Paratype. **Occurrence:** occurrenceDetails: http://janzen.sas.upenn.edu; catalogNumber: DHJPAR0016631; recordedBy: D.H. Janzen, W. Hallwachs & Elda Araya; individualID: DHJPAR0016631; individualCount: 1; sex: female; lifeStage: adult; preparations: pinned; otherCatalogNumbers: ASTAP936-07, 06-SRNP-8879, BOLD:AAF3886; **Taxon:** scientificName: Hyphantrophaga albopilosa; phylum: Arthropoda; class: Insecta; order: Diptera; family: Tachinidae; genus: Hyphantrophaga; specificEpithet: albopilosa; scientificNameAuthorship: Fleming & Wood, 2018; **Location:** continent: Central America; country: Costa Rica; countryCode: CR; stateProvince: Alajuela; county: Sector San Cristobal; locality: Area de Conservacion Guanacaste; verbatimLocality: Sendero Corredor; verbatimElevation: 620; verbatimLatitude: 10.8787; verbatimLongitude: -85.3896; verbatimCoordinateSystem: Decimal; decimalLatitude: 10.8787; decimalLongitude: -85.3896; **Identification:** identifiedBy: AJ Fleming; dateIdentified: 2017; **Event:** samplingProtocol: Reared from the larva of the Sphingidae, Adhemarius ypsilon; verbatimEventDate: 29-Nov-2006; **Record Level:** language: en; institutionCode: CNC; collectionCode: Insects; basisOfRecord: Pinned Specimen
- **Type status:** Paratype. **Occurrence:** occurrenceDetails: http://janzen.sas.upenn.edu; catalogNumber: DHJPAR0007441; recordedBy: D.H. Janzen, W. Hallwachs & Osvaldo Espinoza; individualID: DHJPAR0007441; individualCount: 1; sex: female; lifeStage: adult; preparations: pinned; otherCatalogNumbers: ASTAT213-06, 04-SRNP-3482, BOLD:AAF3886; **Taxon:** scientificName: Hyphantrophaga albopilosa; phylum: Arthropoda; class: Insecta; order: Diptera; family: Tachinidae; genus: Hyphantrophaga; specificEpithet: albopilosa; scientificNameAuthorship: Fleming & Wood, 2018; **Location:** continent: Central America; country: Costa Rica; countryCode: CR; stateProvince: Alajuela; county: Sector San Cristobal; locality: Dos Rios; verbatimLocality: Finca San Gabriel; verbatimElevation: 645; verbatimLatitude: 10.8777; verbatimLongitude: -85.3934; verbatimCoordinateSystem: Decimal; decimalLatitude: 10.8777; decimalLongitude: -85.3934; **Identification:** identifiedBy: AJ Fleming; dateIdentified: 2017; **Event:** samplingProtocol: Reared from the larva of the Sphingidae, Adhemarius ypsilon; verbatimEventDate: 22-Aug-2004; **Record Level:** language: en; institutionCode: CNC; collectionCode: Insects; basisOfRecord: Pinned Specimen
- **Type status:** Paratype. **Occurrence:** occurrenceDetails: http://janzen.sas.upenn.edu; catalogNumber: DHJPAR0050333; recordedBy: D.H. Janzen, W. Hallwachs & Freddy Quesada; individualID: DHJPAR0050333; individualCount: 1; sex: male; lifeStage: adult; preparations: pinned; otherCatalogNumbers: ACGAZ1647-12, 12-SRNP-31215, BOLD:AAF3886; **Taxon:** scientificName: Hyphantrophaga albopilosa; phylum: Arthropoda; class: Insecta; order: Diptera; family: Tachinidae; genus: Hyphantrophaga; specificEpithet: albopilosa; scientificNameAuthorship: Fleming & Wood, 2018; **Location:** continent: Central America; country: Costa Rica; countryCode: CR; stateProvince: Guanacaste; county: Sector Pitilla; locality: Area de Conservacion Guanacaste; verbatimLocality: Amonias; verbatimElevation: 390; verbatimLatitude: 11.0425; verbatimLongitude: -85.4034; verbatimCoordinateSystem: Decimal; decimalLatitude: 11.0425; decimalLongitude: -85.4034; **Identification:** identifiedBy: AJ Fleming; dateIdentified: 2017; **Event:** samplingProtocol: Reared from the larva of the Sphingidae, Adhemarius ypsilon; verbatimEventDate: 08-Sep-2012; **Record Level:** language: en; institutionCode: CNC; collectionCode: Insects; basisOfRecord: Pinned Specimen

#### Description

**Male** (Fig. 3). Length: 7–9 mm. **Head** (Fig. 3b): vertex 1/5 of head width; one pair of reclinate upper orbital setae; ocellar setae arising behind anterior ocellus; ocellar triangle silver (slight gold tinge present but overall concolorous with fronto-orbital plate); fronto-orbital plate shiny silver and setulose, setulae not extending below lowest frontal seta; parafacial brilliant silver and bare; facial ridge bare; eye with short sparse ommatrichia up to 2X as long as one ommatidium; pedicel brownish-black with some orange, concolorous with postpedicel; arista brown, very minutely pubescent, distinctly thickened on basal 1/3–1/4; palpus yellow and densely haired, apically clubbed. **Thorax** (Fig. 3a, c): bright brassy-gold tomentose dorsally, contrasting with silver grey laterally; densely covered in blond setulae along anterior and lateral surfaces, dorsally with dense dark setulae interspersed amongst setae; four thin yet prominent dorsal vittae, outermost two broken across suture, innermost pair unbroken, reaching just beyond 2nd postsutural dorsocentral seta; postpronotum with 4–5 setae arranged in a triangle; chaetotaxy: acrostichal setae 3:3; dorsocentral setae 3:4; intra-alar setae 2:3; supra-alar setae 2:3; two katepisternal setae; basal scutellar setae subequal in length to subapical scutellar setae; lateral scutellar setae less than 1/2 as long as subapical scutellar setae, curving inwards medially; apical scutellar setae subequal in length to lateral scutellar setae, crossed apically; one pair of discal scutellar setae more widely set than apical setae but more narrowly set than subapical setae; scutellum concolorous with scutum. **Legs** (Fig. 3c): brilliant yellow in ground colour; fore femur with dense silver tomentum on posterodorsal surface; hind coxa bare. **Wing** (Fig. 3a): pale translucent, hyaline, not distinctly infuscate; vein R_4+5_ with 2–3 setulae at base. **Abdomen** (Fig. 3a, c): ground colour yellow; middorsal depression on ST1+2 almost reaching hind margin; median marginal setae absent on ST1+2, present on T3; a complete row of marginal setae present on T4; discal setae only on T5; sex patch covering ventral surfaces of T4–T5; distinct brassy tomentose bands along anterior edge of T3 and T4, broken medially by a dorsocentral stripe and covering almost 80% of tergites; T5 with brassy tomentum throughout. **Terminalia** (Fig. 3d, e, f): anterior margin of sternite 5 (Fig. 3f) with a deeply curved medial depression, posterior margin with a deep median cleft, smoothly U-shaped. Lateral lobe of sternite slightly pointed apically and with many short setulae of equal length. Anterior plate of sternite 5 shorter than apical lobes. Unsclerotised "window" anterior to median cleft broadly rounded, stalked and convex reminiscent of a mushroom cap, extending as widely as median cleft. Cerci in posterior view (Fig. 3d) triangular with external edges inwardly wedged, slightly shorter than surstyli, blunt and rounded off towards apex, completely separate medially, diverging slightly at tips; in lateral view with a strong, almost 90 degree bend 1/5 along their length. Surstylus in lateral view (Fig. 3e) almost parallel-sided along its length, ending in a slightly downcurved apex, making the structure appear fingerlike; basal half, twice as wide as apical half; surstylus appearing fused with epandrium. Surstyli when viewed dorsally strongly divergent and wide open. Pregonite well-developed, 1/2 as long as distiphallus, squared off, ending in a trumpet-like, flared apex. Postgonite elongate and slender, horn-shaped, subequal in length to pregonite, slightly narrowed towards apex, not strongly curved. Distiphallus sail-shaped, apically flared, with a slender median longitudinal sclerotised reinforcement on its posterior surface and a broad, anterolateral, sclerotised acrophallus on each side, joining the plate of opposite side on anterior surface near apex.

**Female**. Length: 7–9 mm. As male, differing only by the presence of two pairs of proclinate orbital setae.

#### Diagnosis

*Hyphantrophaga albopilosa* **sp. n.** can be distinguished from all other *Hyphantrophaga* species by the following combination of traits: brilliant silver parafacial, two katepisternal setae, dense covering of blond setulae along lateral surface of thorax, brilliant yellow legs, hind coxa bare and abdomen yellow in ground colour.

#### Etymology

From the Latin adjective “*albopilosa*”, meaning "white-haired", in reference to its blond thoracic setulae.

#### Distribution

Costa Rica, ACG, Alajuela and Guanacaste Provinces, 390–645 m elevation.

#### Ecology

*Hyphantrophaga albopilosa* **sp. n.** has been reared six times from a single species of Lepidoptera in the family Sphingidae, *Adhemarius ypsilon* (Rothschild & Jordan, 1903), in rain forest.

### Hyphantrophaga anacordobae

Fleming & Wood sp. n.

urn:lsid:zoobank.org:act:5F5907DE-B5AD-4947-823F-527624FDE70B

#### Materials

- **Type status:** Holotype. **Occurrence:** occurrenceDetails: http://janzen.sas.upenn.edu; catalogNumber: DHJPAR0007484; recordedBy: D.H. Janzen, W. Hallwachs & Freddy Quesada; individualID: DHJPAR0007484; individualCount: 1; sex: male; lifeStage: adult; preparations: pinned; otherCatalogNumbers: ASTAT256-06, 02-SRNP-23842, BOLD:AAC5448; **Taxon:** scientificName: Hyphantrophaga anacordobae; phylum: Arthropoda; class: Insecta; order: Diptera; family: Tachinidae; genus: Hyphantrophaga; specificEpithet: anacordobae; scientificNameAuthorship: Fleming & Wood, 2018; **Location:** continent: Central America; country: Costa Rica; countryCode: CR; stateProvince: Guanacaste; county: Sector Cacao; locality: Area de Conservacion Guanacaste; verbatimLocality: Sendero Ponderosa; verbatimElevation: 1060; verbatimLatitude: 10.9146; verbatimLongitude: -85.4626; verbatimCoordinateSystem: Decimal; decimalLatitude: 10.9146; decimalLongitude: -85.4626; **Identification:** identifiedBy: AJ Fleming; dateIdentified: 2017; **Event:** samplingProtocol: Reared from the larva of the Nymphalidae, Morpho justitiae; verbatimEventDate: 04-Nov-2002; **Record Level:** language: en; institutionCode: CNC; collectionCode: Insects; basisOfRecord: Pinned Specimen
- **Type status:** Paratype. **Occurrence:** occurrenceDetails: http://janzen.sas.upenn.edu; catalogNumber: DHJPAR0007481; recordedBy: D.H. Janzen, W. Hallwachs & gusaneros; individualID: DHJPAR0007481; individualCount: 1; sex: female; lifeStage: adult; preparations: pinned; otherCatalogNumbers: ASTAT253-06, 95-SRNP-10486, BOLD:AAC5448; **Taxon:** scientificName: Hyphantrophaga anacordobae; phylum: Arthropoda; class: Insecta; order: Diptera; family: Tachinidae; genus: Hyphantrophaga; specificEpithet: anacordobae; scientificNameAuthorship: Fleming & Wood, 2018; **Location:** continent: Central America; country: Costa Rica; countryCode: CR; stateProvince: Guanacaste; county: Sector Santa Maria; locality: Area de Conservacion Guanacaste; verbatimLocality: Estacion Santa Maria; verbatimElevation: 840; verbatimLatitude: 10.7645; verbatimLongitude: -85.3116; verbatimCoordinateSystem: Decimal; decimalLatitude: 10.7645; decimalLongitude: -85.3116; **Identification:** identifiedBy: AJ Fleming; dateIdentified: 2017; **Event:** samplingProtocol: Reared from the larva of the Nymphalidae, Morpho justitiae; verbatimEventDate: 16-Nov-1995; **Record Level:** language: en; institutionCode: CNC; collectionCode: Insects; basisOfRecord: Pinned Specimen
- **Type status:** Paratype. **Occurrence:** occurrenceDetails: http://janzen.sas.upenn.edu; catalogNumber: DHJPAR0007482; recordedBy: D.H. Janzen, W. Hallwachs & Mariano Pereira; individualID: DHJPAR0007482; individualCount: 1; sex: male; lifeStage: adult; preparations: pinned; otherCatalogNumbers: ASTAT254-06, 02-SRNP-23679, BOLD:AAC5448; **Taxon:** scientificName: Hyphantrophaga anacordobae; phylum: Arthropoda; class: Insecta; order: Diptera; family: Tachinidae; genus: Hyphantrophaga; specificEpithet: anacordobae; scientificNameAuthorship: Fleming & Wood, 2018; **Location:** continent: Central America; country: Costa Rica; countryCode: CR; stateProvince: Guanacaste; county: Sector Cacao; locality: Area de Conservacion Guanacaste; verbatimLocality: Sendero Ponderosa; verbatimElevation: 1060; verbatimLatitude: 10.9146; verbatimLongitude: -85.4626; verbatimCoordinateSystem: Decimal; decimalLatitude: 10.9146; decimalLongitude: -85.4626; **Identification:** identifiedBy: AJ Fleming; dateIdentified: 2017; **Event:** samplingProtocol: Reared from the larva of the Nymphalidae, Morpho justitiae; verbatimEventDate: 14-Nov-2002; **Record Level:** language: en; institutionCode: CNC; collectionCode: Insects; basisOfRecord: Pinned Specimen
- **Type status:** Paratype. **Occurrence:** occurrenceDetails: http://janzen.sas.upenn.edu; catalogNumber: DHJPAR0007483; recordedBy: D.H. Janzen, W. Hallwachs & Mariano Pereira; individualID: DHJPAR0007483; individualCount: 1; sex: female; lifeStage: adult; preparations: pinned; otherCatalogNumbers: ASTAT255-06, 02-SRNP-23680, BOLD:AAC5448; **Taxon:** scientificName: Hyphantrophaga anacordobae; phylum: Arthropoda; class: Insecta; order: Diptera; family: Tachinidae; genus: Hyphantrophaga; specificEpithet: anacordobae; scientificNameAuthorship: Fleming & Wood, 2018; **Location:** continent: Central America; country: Costa Rica; countryCode: CR; stateProvince: Guanacaste; county: Sector Cacao; locality: Area de Conservacion Guanacaste; verbatimLocality: Sendero Ponderosa; verbatimElevation: 1060; verbatimLatitude: 10.9146; verbatimLongitude: -85.4626; verbatimCoordinateSystem: Decimal; decimalLatitude: 10.9146; decimalLongitude: -85.4626; **Identification:** identifiedBy: AJ Fleming; dateIdentified: 2017; **Event:** samplingProtocol: Reared from the larva of the Nymphalidae, Morpho justitiae; verbatimEventDate: 11-Oct-2002; **Record Level:** language: en; institutionCode: CNC; collectionCode: Insects; basisOfRecord: Pinned Specimen
- **Type status:** Paratype. **Occurrence:** occurrenceDetails: http://janzen.sas.upenn.edu; catalogNumber: DHJPAR0007480; recordedBy: D.H. Janzen, W. Hallwachs & gusaneros; individualID: DHJPAR0007480; individualCount: 1; sex: female; lifeStage: adult; preparations: pinned; otherCatalogNumbers: ASTAT252-06, 95-SRNP-10486.1, BOLD:AAC5448; **Taxon:** scientificName: Hyphantrophaga anacordobae; phylum: Arthropoda; class: Insecta; order: Diptera; family: Tachinidae; genus: Hyphantrophaga; specificEpithet: anacordobae; scientificNameAuthorship: Fleming & Wood, 2018; **Location:** continent: Central America; country: Costa Rica; countryCode: CR; stateProvince: Guanacaste; county: Sector Santa Maria; locality: Area de Conservacion Guanacaste; verbatimLocality: Estacion Santa Maria; verbatimElevation: 840; verbatimLatitude: 10.7645; verbatimLongitude: -85.3116; verbatimCoordinateSystem: Decimal; decimalLatitude: 10.7645; decimalLongitude: -85.3116; **Identification:** identifiedBy: AJ Fleming; dateIdentified: 2017; **Event:** samplingProtocol: Reared from the larva of the Nymphalidae, Morpho justitiae; verbatimEventDate: 19-Nov-1995; **Record Level:** language: en; institutionCode: CNC; collectionCode: Insects; basisOfRecord: Pinned Specimen
- **Type status:** Paratype. **Occurrence:** occurrenceDetails: http://janzen.sas.upenn.edu; catalogNumber: DHJPAR0007485; recordedBy: D.H. Janzen, W. Hallwachs & Guillermo Pereira; individualID: DHJPAR0007485; individualCount: 1; sex: male; lifeStage: adult; preparations: pinned; otherCatalogNumbers: ASTAT257-06, 02-SRNP-23678, BOLD:AAC5448; **Taxon:** scientificName: Hyphantrophaga anacordobae; phylum: Arthropoda; class: Insecta; order: Diptera; family: Tachinidae; genus: Hyphantrophaga; specificEpithet: anacordobae; scientificNameAuthorship: Fleming & Wood, 2018; **Location:** continent: Central America; country: Costa Rica; countryCode: CR; stateProvince: Guanacaste; county: Sector Cacao; locality: Area de Conservacion Guanacaste; verbatimLocality: Sendero Ponderosa; verbatimElevation: 1060; verbatimLatitude: 10.9146; verbatimLongitude: -85.4626; verbatimCoordinateSystem: Decimal; decimalLatitude: 10.9146; decimalLongitude: -85.4626; **Identification:** identifiedBy: AJ Fleming; dateIdentified: 2017; **Event:** samplingProtocol: Reared from the larva of the Nymphalidae, Morpho justitiae; verbatimEventDate: 16-Nov-2002; **Record Level:** language: en; institutionCode: CNC; collectionCode: Insects; basisOfRecord: Pinned Specimen
- **Type status:** Paratype. **Occurrence:** occurrenceDetails: http://janzen.sas.upenn.edu; catalogNumber: DHJPAR0007486; recordedBy: D.H. Janzen, W. Hallwachs & Freddy Quesada; individualID: DHJPAR0007486; individualCount: 1; sex: female; lifeStage: adult; preparations: pinned; otherCatalogNumbers: ASTAT258-06, 02-SRNP-24540.7, BOLD:AAC5448; **Taxon:** scientificName: Hyphantrophaga anacordobae; phylum: Arthropoda; class: Insecta; order: Diptera; family: Tachinidae; genus: Hyphantrophaga; specificEpithet: anacordobae; scientificNameAuthorship: Fleming & Wood, 2018; **Location:** continent: Central America; country: Costa Rica; countryCode: CR; stateProvince: Guanacaste; county: Sector Cacao; locality: Area de Conservacion Guanacaste; verbatimLocality: Sendero Ponderosa; verbatimElevation: 1060; verbatimLatitude: 10.9146; verbatimLongitude: -85.4626; verbatimCoordinateSystem: Decimal; decimalLatitude: 10.9146; decimalLongitude: -85.4626; **Identification:** identifiedBy: AJ Fleming; dateIdentified: 2017; **Event:** samplingProtocol: Reared from the larva of the Nymphalidae, Morpho justitiae; verbatimEventDate: 28-Jan-2003; **Record Level:** language: en; institutionCode: CNC; collectionCode: Insects; basisOfRecord: Pinned Specimen
- **Type status:** Paratype. **Occurrence:** occurrenceDetails: http://janzen.sas.upenn.edu; catalogNumber: DHJPAR0007487; recordedBy: D.H. Janzen, W. Hallwachs & Harry Ramirez; individualID: DHJPAR0007487; individualCount: 1; sex: male; lifeStage: adult; preparations: pinned; otherCatalogNumbers: ASTAT259-06, 02-SRNP-24536.7, BOLD:AAC5448; **Taxon:** scientificName: Hyphantrophaga anacordobae; phylum: Arthropoda; class: Insecta; order: Diptera; family: Tachinidae; genus: Hyphantrophaga; specificEpithet: anacordobae; scientificNameAuthorship: Fleming & Wood, 2018; **Location:** continent: Central America; country: Costa Rica; countryCode: CR; stateProvince: Guanacaste; county: Sector Cacao; locality: Area de Conservacion Guanacaste; verbatimLocality: Sendero Ponderosa; verbatimElevation: 1060; verbatimLatitude: 10.9146; verbatimLongitude: -85.4626; verbatimCoordinateSystem: Decimal; decimalLatitude: 10.9146; decimalLongitude: -85.4626; **Identification:** identifiedBy: AJ Fleming; dateIdentified: 2017; **Event:** samplingProtocol: Reared from the larva of the Nymphalidae, Morpho justitiae; verbatimEventDate: 31-Jan-2003; **Record Level:** language: en; institutionCode: CNC; collectionCode: Insects; basisOfRecord: Pinned Specimen
- **Type status:** Paratype. **Occurrence:** occurrenceDetails: http://janzen.sas.upenn.edu; catalogNumber: DHJPAR0007488; recordedBy: D.H. Janzen, W. Hallwachs & Mariano Pereira; individualID: DHJPAR0007488; individualCount: 1; sex: male; lifeStage: adult; preparations: pinned; otherCatalogNumbers: ASTAT260-06, 02-SRNP-24200, BOLD:AAC5448; **Taxon:** scientificName: Hyphantrophaga anacordobae; phylum: Arthropoda; class: Insecta; order: Diptera; family: Tachinidae; genus: Hyphantrophaga; specificEpithet: anacordobae; scientificNameAuthorship: Fleming & Wood, 2018; **Location:** continent: Central America; country: Costa Rica; countryCode: CR; stateProvince: Guanacaste; county: Sector Cacao; locality: Area de Conservacion Guanacaste; verbatimLocality: Sendero Ponderosa; verbatimElevation: 1060; verbatimLatitude: 10.9146; verbatimLongitude: -85.4626; verbatimCoordinateSystem: Decimal; decimalLatitude: 10.9146; decimalLongitude: -85.4626; **Identification:** identifiedBy: AJ Fleming; dateIdentified: 2017; **Event:** samplingProtocol: Reared from the larva of the Nymphalidae, Morpho justitiae; verbatimEventDate: 11-Dec-2002; **Record Level:** language: en; institutionCode: CNC; collectionCode: Insects; basisOfRecord: Pinned Specimen
- **Type status:** Paratype. **Occurrence:** occurrenceDetails: http://janzen.sas.upenn.edu; catalogNumber: DHJPAR0007489; recordedBy: D.H. Janzen, W. Hallwachs & Mariano Pereira; individualID: DHJPAR0007489; individualCount: 1; sex: female; lifeStage: adult; preparations: pinned; otherCatalogNumbers: ASTAT261-06, 02-SRNP-24542, BOLD:AAC5448; **Taxon:** scientificName: Hyphantrophaga anacordobae; phylum: Arthropoda; class: Insecta; order: Diptera; family: Tachinidae; genus: Hyphantrophaga; specificEpithet: anacordobae; scientificNameAuthorship: Fleming & Wood, 2018; **Location:** continent: Central America; country: Costa Rica; countryCode: CR; stateProvince: Guanacaste; county: Sector Cacao; locality: Area de Conservacion Guanacaste; verbatimLocality: Sendero Ponderosa; verbatimElevation: 1060; verbatimLatitude: 10.9146; verbatimLongitude: -85.4626; verbatimCoordinateSystem: Decimal; decimalLatitude: 10.9146; decimalLongitude: -85.4626; **Identification:** identifiedBy: AJ Fleming; dateIdentified: 2017; **Event:** samplingProtocol: Reared from the larva of the Nymphalidae, Morpho justitiae; verbatimEventDate: 21-Feb-2003; **Record Level:** language: en; institutionCode: CNC; collectionCode: Insects; basisOfRecord: Pinned Specimen
- **Type status:** Paratype. **Occurrence:** occurrenceDetails: http://janzen.sas.upenn.edu; catalogNumber: DHJPAR0019597; recordedBy: D.H. Janzen, W. Hallwachs & Roster Moraga; individualID: DHJPAR0019597; individualCount: 1; sex: female; lifeStage: adult; preparations: pinned; otherCatalogNumbers: ASTAB145-07, 07-SRNP-21398, BOLD:AAC5448; **Taxon:** scientificName: Hyphantrophaga anacordobae; phylum: Arthropoda; class: Insecta; order: Diptera; family: Tachinidae; genus: Hyphantrophaga; specificEpithet: anacordobae; scientificNameAuthorship: Fleming & Wood, 2018; **Location:** continent: Central America; country: Costa Rica; countryCode: CR; stateProvince: Guanacaste; county: Sector Del Oro; locality: Area de Conservacion Guanacaste; verbatimLocality: Monte Cristo; verbatimElevation: 525; verbatimLatitude: 11.0137; verbatimLongitude: -85.4253; verbatimCoordinateSystem: Decimal; decimalLatitude: 11.0137; decimalLongitude: -85.4253; **Identification:** identifiedBy: AJ Fleming; dateIdentified: 2017; **Event:** samplingProtocol: Reared from the larva of the Nymphalidae, Morpho justitiae; verbatimEventDate: 26-May-2007; **Record Level:** language: en; institutionCode: CNC; collectionCode: Insects; basisOfRecord: Pinned Specimen
- **Type status:** Paratype. **Occurrence:** occurrenceDetails: http://janzen.sas.upenn.edu; catalogNumber: DHJPAR0040955; recordedBy: D.H. Janzen, W. Hallwachs & Carolina Cano; individualID: DHJPAR0040955; individualCount: 1; sex: male; lifeStage: adult; preparations: pinned; otherCatalogNumbers: ASHYF870-11, 10-SRNP-4655, BOLD:AAC5448; **Taxon:** scientificName: Hyphantrophaga anacordobae; phylum: Arthropoda; class: Insecta; order: Diptera; family: Tachinidae; genus: Hyphantrophaga; specificEpithet: anacordobae; scientificNameAuthorship: Fleming & Wood, 2018; **Location:** continent: Central America; country: Costa Rica; countryCode: CR; stateProvince: Alajuela; county: Sector San Cristobal; locality: Area de Conservacion Guanacaste; verbatimLocality: Tajo Angeles; verbatimElevation: 540; verbatimLatitude: 10.8647; verbatimLongitude: -85.4153; verbatimCoordinateSystem: Decimal; decimalLatitude: 10.8647; decimalLongitude: -85.4153; **Identification:** identifiedBy: AJ Fleming; dateIdentified: 2017; **Event:** samplingProtocol: Reared from the larva of the Nymphalidae, Morpho amathonte; verbatimEventDate: 24-Nov-2010; **Record Level:** language: en; institutionCode: CNC; collectionCode: Insects; basisOfRecord: Pinned Specimen
- **Type status:** Paratype. **Occurrence:** occurrenceDetails: http://janzen.sas.upenn.edu; catalogNumber: DHJPAR0059877; recordedBy: D.H. Janzen, W. Hallwachs & Manuel Rios; individualID: DHJPAR0059877; individualCount: 1; sex: male; lifeStage: adult; preparations: pinned; otherCatalogNumbers: ACGBA6298-16, 16-SRNP-31601, BOLD:AAC5448; **Taxon:** scientificName: Hyphantrophaga anacordobae; phylum: Arthropoda; class: Insecta; order: Diptera; family: Tachinidae; genus: Hyphantrophaga; specificEpithet: anacordobae; scientificNameAuthorship: Fleming & Wood, 2018; **Location:** continent: Central America; country: Costa Rica; countryCode: CR; stateProvince: Guanacaste; county: Sector Pitilla; locality: Area de Conservacion Guanacaste; verbatimLocality: Sendero Cuestona; **Identification:** identifiedBy: AJ Fleming; dateIdentified: 2107; **Event:** verbatimEventDate: 09-Oct-2016; **Record Level:** language: en; institutionCode: CNC; collectionCode: Insects; basisOfRecord: Pinned Specimen

#### Description

**Male** (Fig. 4). Length: 9–12 mm. **Head** (Fig. 4b): vertex 1/4 of head width; two pairs of reclinate upper orbital setae; ocellar setae arising behind anterior ocellus; ocellar triangle black medially, brassy/silver laterally; fronto-orbital plate shiny silver and densely setulose, setulae not extending below lowest frontal seta; parafacial silver and bare; eye densely haired; facial ridge bare; pedicel black, concolorous with postpedicel; arista dark brown, very minutely pubescent, distinctly thickened on basal 1/3–1/4; palpus yellow and densely haired so as to appear darkened, apically with a slight clubbed appearance. **Thorax** (Fig. 4a, c): slightly brassy tomentose dorsally, contrasting with dark silver grey laterally; densely covered in black setulae along all surfaces, interspersed amongst setae; four thick and prominent dorsal vittae, outermost two broken across suture, innermost pair unbroken, reaching just beyond 2nd postsutural dorsocentral seta; postpronotum with 5–6 setae arranged in a triangle; chaetotaxy: acrostichal setae 3:3; dorsocentral setae 3:4; intra-alar setae 2–3:3; supra-alar setae 2:3; three katepisternal setae; basal scutellar setae subequal to slightly shorter than subapical scutellar setae; lateral scutellar setae subequal in length to apical setae, with a strong inward curve medially; apical scutellar setae 1/2 as long as subapical scutellar setae, crossed apically; one pair of discal scutellar setae set as widely apart as subapical setae; scutellum darkened across basal 20%, remainder concolorous with scutum. **Legs** (Fig. 4c): ground colour black; fore femur with dense silver tomentum on posterodorsal surface; hind coxa bare. **Wing** (Fig. 4a): pale translucent, hyaline, not distinctly infuscate; vein R_4+5_ with 3–4 setulae at base. **Abdomen** (Fig. 4a, c): ground colour dark reddish-brown, appearing black; middorsal depression on ST1+2 almost reaching hind margin; median marginal setae absent on ST1+2, present on T3; complete row of marginal setae present on T4; discal setae only on T5; sex patch covering 50% of the laterodorsal surface of T4 and the ventral surfaces of T4–T5; distinct brassy tomentose bands along anterior edge of T3 and T4, broken medially by a dorsocentral stripe and covering almost 40% of tergites; T5 with brassy tomentum throughout. **Terminalia** (Fig. 4d, e, f): sternite 5 (Fig. 4f) with a deeply excavated median cleft, squared off U-shaped, margins covered in dense tomentum. Lateral lobes of sternite rounded triangular apically, with 2–3 strong setae surrounded by many shorter, weaker setulae. Anterior plate of sternite 5 subequal to length of apical lobes; unsclerotised "window" slightly rectangular, two palmlike arms extending as wide as median cleft. Lateral lobe of sternite slightly pointed apically and with many short setae of equal length. Cerci in posterior view (Fig. 4d) slightly triangular and subequal in length to surstyli, blunt and rounded at apex, completely separate medially, appearing but not divergent; in lateral view with a strong downward angle commencing at midpoint; densely setulose along basal 2/3 dorsally, setulose ventrally along entire length (visible in lateral view). Surstylus in lateral view (Fig. 4e) almost parallel-sided along its length, ending in a slightly downcurved apex, making the structure appear blade-like; surstylus appearing to be fused with epandrium; when viewed dorsally surstyli appearing concave with a bulbous base where surstyli meet epandrium, inner margin straight slightly divergent at apices. Pregonite short, 0.5 times as long as distiphallus, bare and rounded off apically. Postgonite slightly narrow, 1/3 as wide as pregonite, sharply pointed and curved, vaguely C-shaped. Distiphallus ~1.5 times as long as basiphallus. Distiphallus sail-shaped, with a slender median longitudinal sclerotised reinforcement on its posterior surface and a broad, anterolateral, sclerotised acrophallus on each side, joining the plate of opposite side on anterior surface near apex.

**Female**. Length: 8–11 mm. As male, differing only by the presence of two pairs of proclinate orbital setae.

#### Diagnosis

*Hyphantrophaga anacordobae* **sp. n.** can be distinguished from all other *Hyphantrophaga* species by the following combination of traits: three katepisternal setae, legs entirely black, hind coxa bare, ground colour of abdomen dark reddish-brown (appearing black under certain angles of light) and median marginal setae absent from ST1+2.

#### Etymology

*Hyphantrophaga anacordobae* **sp. n.** is named in recognition of Anabelle Córdoba Alemán's dedication and work in finding and rearing the ACG caterpillars that contained tachinid larvae.

#### Distribution

Costa Rica, ACG, Guanacaste Province, 525–1060 m elevation.

#### Ecology

*Hyphantrophaga anacordobae* **sp. n.** has been reared 13 times from two species of Lepidoptera in the family Nymphalidae, *Morpho helenor* (Cramer, 1776) and *Morpho justitiae* (Salvin & Godman, 1868), in rain forest, cloud forest and dry-rain lowland intergrade.

### Hyphantrophaga angustata

(van der Wulp, 1890)

#### Materials

- **Type status:** Holotype. **Occurrence:** recordedBy: H.H. Smith; individualCount: 1; sex: male; lifeStage: adult; preparations: pinned; **Taxon:** scientificName: Hyphantrophaga angustata; phylum: Arthropoda; class: Insecta; order: Diptera; family: Tachinidae; genus: Exorista; specificEpithet: angustata; scientificNameAuthorship: (van der Wulp, 1890); **Location:** continent: Central America; country: Mexico; countryCode: MX; stateProvince: Guerrero; county: Chilpancingo; verbatimElevation: 4600; **Identification:** identifiedBy: Wood; dateIdentified: 2015; **Record Level:** language: en; institutionCode: NHMUK; collectionCode: Insects; basisOfRecord: Pinned Specimen
- **Type status:** Other material. **Occurrence:** recordedBy: C.V. Riley; individualID: 25702; individualCount: 1; sex: male; lifeStage: adult; preparations: pinned; otherCatalogNumbers: Type. No. 25702 USNM; **Taxon:** scientificName: Hyphantrophaga angustata; phylum: Arthropoda; class: Insecta; order: Diptera; family: Tachinidae; genus: Zenillia; specificEpithet: coquilletti; scientificNameAuthorship: (Aldrich & Webber, 1924); **Location:** continent: North America; country: United States of America; countryCode: US; stateProvince: Texas; county: Belfrage; **Identification:** identifiedBy: AJ Fleming; dateIdentified: 2017; **Record Level:** language: en; institutionCode: USNM; collectionCode: Insects; basisOfRecord: Pinned Specimen
- **Type status:** Other material. **Occurrence:** occurrenceDetails: http://janzen.sas.upenn.edu; catalogNumber: DHJPAR0052588; recordedBy: D.H. Janzen, W. Hallwachs & Jose Cortez; individualID: DHJPAR0052588; individualCount: 1; sex: male; lifeStage: adult; preparations: pinned; otherCatalogNumbers: ASHYM1942-13, 13-SRNP-55696, BOLD:AAD0627; **Taxon:** scientificName: Hyphantrophaga angustata; phylum: Arthropoda; class: Insecta; order: Diptera; family: Tachinidae; genus: Hyphantrophaga; specificEpithet: angustata; scientificNameAuthorship: (van der Wulp, 1890); **Location:** continent: Central America; country: Costa Rica; countryCode: CR; stateProvince: Guanacaste; county: Sector Mundo Nuevo; locality: Area de Conservacion Guanacaste; verbatimLocality: Estacion La Perla; verbatimElevation: 325; verbatimLatitude: 10.7674; verbatimLongitude: -85.4331; verbatimCoordinateSystem: Decimal; decimalLatitude: 10.7674; decimalLongitude: -85.4331; **Identification:** identifiedBy: AJ Fleming; dateIdentified: 2017; **Event:** samplingProtocol: Reared from the larva of the Thyrididae, Dysodia immargo; verbatimEventDate: 17-Aug-2013; **Record Level:** language: en; institutionCode: CNC; collectionCode: Insects; basisOfRecord: Pinned Specimen
- **Type status:** Other material. **Occurrence:** occurrenceDetails: http://janzen.sas.upenn.edu; catalogNumber: DHJPAR0007320; recordedBy: D.H. Janzen, W. Hallwachs & gusaneros; individualID: DHJPAR0007320; individualCount: 1; sex: female; lifeStage: adult; preparations: pinned; otherCatalogNumbers: ASTAT092-06, 01-SRNP-14940, BOLD:AAD0627; **Taxon:** scientificName: Hyphantrophaga angustata; phylum: Arthropoda; class: Insecta; order: Diptera; family: Tachinidae; genus: Hyphantrophaga; specificEpithet: angustata; scientificNameAuthorship: (van der Wulp, 1890); **Location:** continent: Central America; country: Costa Rica; countryCode: CR; stateProvince: Guanacaste; county: Sector Santa Rosa; locality: Area de Conservacion Guanacaste; verbatimLocality: Bosque San Emilio; verbatimElevation: 300; verbatimLatitude: 10.8439; verbatimLongitude: -85.6138; verbatimCoordinateSystem: Decimal; decimalLatitude: 10.8439; decimalLongitude: -85.6138; **Identification:** identifiedBy: AJ Fleming; dateIdentified: 2017; **Event:** samplingProtocol: Reared from the larva of the Thyrididae, Dysodia speculifera; verbatimEventDate: 14-Oct-2001; **Record Level:** language: en; institutionCode: CNC; collectionCode: Insects; basisOfRecord: Pinned Specimen
- **Type status:** Other material. **Occurrence:** occurrenceDetails: http://janzen.sas.upenn.edu; catalogNumber: DHJPAR0007317; recordedBy: D.H. Janzen & W. Hallwachs; individualID: DHJPAR0007317; individualCount: 1; sex: male; lifeStage: adult; preparations: pinned; otherCatalogNumbers: ASTAT089-06, 02-SRNP-12711, BOLD:AAD0627; **Taxon:** scientificName: Hyphantrophaga angustata; phylum: Arthropoda; class: Insecta; order: Diptera; family: Tachinidae; genus: Hyphantrophaga; specificEpithet: angustata; scientificNameAuthorship: (van der Wulp, 1890); **Location:** continent: Central America; country: Costa Rica; countryCode: CR; stateProvince: Guanacaste; county: Sector Santa Rosa; locality: Area de Conservacion Guanacaste; verbatimLocality: Area Administrativa; verbatimElevation: 295; verbatimLatitude: 10.8376; verbatimLongitude: -85.6187; verbatimCoordinateSystem: Decimal; decimalLatitude: 10.8376; decimalLongitude: -85.6187; **Identification:** identifiedBy: AJ Fleming; dateIdentified: 2017; **Event:** samplingProtocol: Reared from the larva of the Thyrididae, Dysodia speculifera; verbatimEventDate: 15-Aug-2002; **Record Level:** language: en; institutionCode: CNC; collectionCode: Insects; basisOfRecord: Pinned Specimen
- **Type status:** Other material. **Occurrence:** occurrenceDetails: http://janzen.sas.upenn.edu; catalogNumber: DHJPAR0007323; recordedBy: D.H. Janzen & W. Hallwachs; individualID: DHJPAR0007323; individualCount: 1; sex: male; lifeStage: adult; preparations: pinned; otherCatalogNumbers: ASTAT095-06, 02-SRNP-12709, BOLD:AAD0627; **Taxon:** scientificName: Hyphantrophaga angustata; phylum: Arthropoda; class: Insecta; order: Diptera; family: Tachinidae; genus: Hyphantrophaga; specificEpithet: angustata; scientificNameAuthorship: (van der Wulp, 1890); **Location:** continent: Central America; country: Costa Rica; countryCode: CR; stateProvince: Guanacaste; county: Sector Santa Rosa; locality: Area de Conservacion Guanacaste; verbatimLocality: Area Administrativa; verbatimElevation: 295; verbatimLatitude: 10.8376; verbatimLongitude: -85.6187; verbatimCoordinateSystem: Decimal; decimalLatitude: 10.8376; decimalLongitude: -85.6187; **Identification:** identifiedBy: AJ Fleming; dateIdentified: 2017; **Event:** samplingProtocol: Reared from the larva of the Thyrididae, Dysodia speculifera; verbatimEventDate: 04-Aug-2002; **Record Level:** language: en; institutionCode: CNC; collectionCode: Insects; basisOfRecord: Pinned Specimen
- **Type status:** Other material. **Occurrence:** occurrenceDetails: http://janzen.sas.upenn.edu; catalogNumber: DHJPAR0007319; recordedBy: D.H. Janzen, W. Hallwachs & gusaneros; individualID: DHJPAR0007319; individualCount: 1; sex: male; lifeStage: adult; preparations: pinned; otherCatalogNumbers: ASTAT091-06, 01-SRNP-14941, BOLD:AAD0627; **Taxon:** scientificName: Hyphantrophaga angustata; phylum: Arthropoda; class: Insecta; order: Diptera; family: Tachinidae; genus: Hyphantrophaga; specificEpithet: angustata; scientificNameAuthorship: (van der Wulp, 1890); **Location:** continent: Central America; country: Costa Rica; countryCode: CR; stateProvince: Guanacaste; county: Sector Santa Rosa; locality: Area de Conservacion Guanacaste; verbatimLocality: Bosque San Emilio; verbatimElevation: 300; verbatimLatitude: 10.8439; verbatimLongitude: -85.6138; verbatimCoordinateSystem: Decimal; decimalLatitude: 10.8439; decimalLongitude: -85.6138; **Identification:** identifiedBy: AJ Fleming; dateIdentified: 2017; **Event:** samplingProtocol: Reared from the larva of the Thyrididae, Dysodia speculifera; verbatimEventDate: 25-Aug-2001; **Record Level:** language: en; institutionCode: CNC; collectionCode: Insects; basisOfRecord: Pinned Specimen
- **Type status:** Other material. **Occurrence:** occurrenceDetails: http://janzen.sas.upenn.edu; catalogNumber: DHJPAR0007321; recordedBy: D.H. Janzen, W. Hallwachs & gusaneros; individualID: DHJPAR0007321; individualCount: 1; sex: female; lifeStage: adult; preparations: pinned; otherCatalogNumbers: ASTAT093-06, 01-SRNP-14942, BOLD:AAD0627; **Taxon:** scientificName: Hyphantrophaga angustata; phylum: Arthropoda; class: Insecta; order: Diptera; family: Tachinidae; genus: Hyphantrophaga; specificEpithet: angustata; scientificNameAuthorship: (van der Wulp, 1890); **Location:** continent: Central America; country: Costa Rica; countryCode: CR; stateProvince: Guanacaste; county: Sector Santa Rosa; locality: Area de Conservacion Guanacaste; verbatimLocality: Bosque San Emilio; verbatimElevation: 300; verbatimLatitude: 10.8439; verbatimLongitude: -85.6138; verbatimCoordinateSystem: Decimal; decimalLatitude: 10.8439; decimalLongitude: -85.6138; **Identification:** identifiedBy: AJ Fleming; dateIdentified: 2017; **Event:** samplingProtocol: Reared from the larva of the Thyrididae, Dysodia speculifera; verbatimEventDate: 13-Oct-2001; **Record Level:** language: en; institutionCode: CNC; collectionCode: Insects; basisOfRecord: Pinned Specimen
- **Type status:** Other material. **Occurrence:** occurrenceDetails: http://janzen.sas.upenn.edu; catalogNumber: DHJPAR0007322; recordedBy: D.H. Janzen & W. Hallwachs; individualID: DHJPAR0007322; individualCount: 1; lifeStage: adult; preparations: pinned; otherCatalogNumbers: ASTAT094-06, 02-SRNP-12710,; **Taxon:** scientificName: Hyphantrophaga angustata; phylum: Arthropoda; class: Insecta; order: Diptera; family: Tachinidae; genus: Hyphantrophaga; specificEpithet: angustata; scientificNameAuthorship: (van der Wulp, 1890); **Location:** continent: Central America; country: Costa Rica; countryCode: CR; stateProvince: Guanacaste; county: Sector Santa Rosa; locality: Area de Conservacion Guanacaste; verbatimLocality: Area Administrativa; verbatimElevation: 295; verbatimLatitude: 10.8376; verbatimLongitude: -85.6187; verbatimCoordinateSystem: Decimal; decimalLatitude: 10.8376; decimalLongitude: -85.6187; **Identification:** identifiedBy: AJ Fleming; dateIdentified: 2017; **Event:** samplingProtocol: Reared from the larva of the Thyrididae, Dysodia speculifera; verbatimEventDate: 30-Sep-2002; **Record Level:** language: en; institutionCode: CNC; collectionCode: Insects; basisOfRecord: Pinned Specimen
- **Type status:** Other material. **Occurrence:** occurrenceDetails: http://janzen.sas.upenn.edu; catalogNumber: DHJPAR0007324; recordedBy: D.H. Janzen, W. Hallwachs & gusaneros; individualID: DHJPAR0007324; individualCount: 1; lifeStage: adult; preparations: pinned; otherCatalogNumbers: ASTAT096-06, 90-SRNP-975, BOLD:AAD0627; **Taxon:** scientificName: Hyphantrophaga angustata; phylum: Arthropoda; class: Insecta; order: Diptera; family: Tachinidae; genus: Hyphantrophaga; specificEpithet: angustata; scientificNameAuthorship: (van der Wulp, 1890); **Location:** continent: Central America; country: Costa Rica; countryCode: CR; stateProvince: Guanacaste; county: Sector Santa Rosa; locality: Area de Conservacion Guanacaste; verbatimLocality: Luces; verbatimElevation: 300; verbatimLatitude: 10.8536; verbatimLongitude: -85.6094; verbatimCoordinateSystem: Decimal; decimalLatitude: 10.8536; decimalLongitude: -85.6094; **Identification:** identifiedBy: AJ Fleming; dateIdentified: 2017; **Event:** samplingProtocol: Reared from the larva of the Thyrididae, Dysodia speculifera; verbatimEventDate: 24-Jul-1990; **Record Level:** language: en; institutionCode: CNC; collectionCode: Insects; basisOfRecord: Pinned Specimen
- **Type status:** Other material. **Occurrence:** occurrenceDetails: http://janzen.sas.upenn.edu; catalogNumber: DHJPAR0007318; recordedBy: D.H. Janzen & W. Hallwachs; individualID: DHJPAR0007318; individualCount: 1; lifeStage: adult; preparations: pinned; otherCatalogNumbers: ASTAT090-06, 02-SRNP-12713, BOLD:AAD0627; **Taxon:** scientificName: Hyphantrophaga angustata; phylum: Arthropoda; class: Insecta; order: Diptera; family: Tachinidae; genus: Hyphantrophaga; specificEpithet: angustata; scientificNameAuthorship: (van der Wulp, 1890); **Location:** continent: Central America; country: Costa Rica; countryCode: CR; stateProvince: Guanacaste; county: Sector Santa Rosa; locality: Area de Conservacion Guanacaste; verbatimLocality: Area Administrativa; verbatimElevation: 295; verbatimLatitude: 10.8376; verbatimLongitude: -85.6187; verbatimCoordinateSystem: Decimal; decimalLatitude: 10.8376; decimalLongitude: -85.6187; **Identification:** identifiedBy: AJ Fleming; dateIdentified: 2017; **Event:** samplingProtocol: Reared from the larva of the Thyrididae, Dysodia speculifera; verbatimEventDate: 18-Jun-2003; **Record Level:** language: en; institutionCode: CNC; collectionCode: Insects; basisOfRecord: Pinned Specimen

#### Description

**Male** (Fig. 5). Length 10–12 mm. **Head** (Fig. 5b): vertex 1/3 of head width; three pairs of reclinate upper orbital setae; ocellar setae arising behind anterior ocellus; ocellar triangle slightly gold-tinged compared to rest of fronto-orbital plate; fronto-orbital plate shiny silver and setulose, setulae not extending below lowest frontal seta; parafacial silver and bare; eye densely haired; facial ridge bare; pedicel black, with orange at base where it joins the postpedicel, remainder concolorous with postpedicel; arista orange basally, very minutely pubescent, distinctly thickened on basal 1/3–1/4; palpus dark brown and haired basally, apically orange and bare. **Thorax** (Fig. 5a, c): silver grey tomentose dorsally and laterally; four thin dorsal vittae, outermost two broken across suture, innermost pair unbroken, reaching just beyond 1st postsutural dorsocentral seta; postpronotum with 4–5 setae arranged in a triangle; chaetotaxy: acrostichal setae 3–4:3; dorsocentral setae 3–4:3; intra-alar setae 3:3; supra-alar setae 2:3; three katepisternal setae; lateral scutellar setae subequal in length to basal scutellar setae; subapical scutellar setae strongest and longest of marginal scutellar setae, strongly divergent; apical scutellar setae subequal to lateral scutellar setae 2/3 length of subapical scutellar setae, crossed apically; one pair of discal scutellar setae set more widely set than apical scutellar setae but more narrowly than subapical scutellar setae; scutellum very slightly darkened across basal 20%, remainder concolorous with scutum. **Legs** (Fig. 5c): yellow in ground colour with dense covering of black hairs, making them appear darker; fore femur with dense silver tomentum on posterodorsal surface; hind coxa bare. **Wing** (Fig. 5a): pale translucent, slightly infuscate; vein R_4+5_ with two setulae at base. **Abdomen** (Fig. 5a, c): ground colour black; mid-dorsal depression on ST1+2 not reaching hind margin; median marginal setae present on ST1+2 and T3; a complete row of marginal setae present on T4; discal setae only on T5; sex patch covering ventral surfaces of T4–T5; distinct tomentose bands along anterior 2/3 of T3 and T4, broken medially by a dorsocentral stripe; T5 with silver tomentum covering anterior 1/3, apparent only laterally but appearing as if it covers entire tergite under certain angles of light. **Terminalia** (Fig. 5d, e, f): anterior plate of sternite 5 (Fig. 5f) with broadly rounded edge, with a very slight, almost absent medial depression; posterior edge with a deeply excavated median cleft, smoothly U-shaped, margins covered in dense tomentum; unsclerotised "window" anterior to median cleft umbonate, 2X as wide as median cleft. Posterior lobes of sternite rounded apically, with 4–6 strong setae surrounded by many shorter, weaker setulae; anterior plate of sternite 2X as long as length of posterior lobes. Cerci in posterior view (Fig. 5d) long, slender and digitiform, longer than surstyli; blunt and rounded at apex, completely separate medially; densely setulose along basal 2/3 dorsally, setulose ventrally along entire length (visible in lateral view) ; in lateral view (Fig. 5e) with a slight downward curve apically. Surstylus in lateral view almost parallel-sided along its length, with a rounded apex, giving the whole structure a spatulate appearance; when viewed dorsally the surstyli appearing to converge slightly. Pregonite broad and well-developed, tapering to a rounded, slightly blunted apex; devoid of setulae. Postgonite narrow up to 1/3, as wide as pregonite, sharply pointed and curved at apex, scythe-like and subequal in length to pregonite. Epiphallus well-developed and apically hooked. Distiphallus rectangular in shape, with a slender median longitudinal sclerotised reinforcement on its posterior surface and a broad, anterolateral, sclerotised acrophallus, joining on both sides of anterior surface near apex.

**Female**. Length: 8–11 mm. As male, differing only by the presence of two pairs of proclinate orbital setae.

#### Diagnosis

*Hyphantrophaga angustata* (van der Wulp) can be distinguished from all other *Hyphantrophaga* species by the following combination of traits: pedicel orange at base, palpus reddish-orange apically, black basally, three katepisternal setae, legs yellow with dense tomentum making them appear black, hind coxa bare, abdominal ground colour black, abdomen with silver tomentum, T5 with silver tomentum ventrolaterally, median marginal setae present on ST1+2.

#### Distribution

From south-western USA south to Costa Rica; Costa Rica, ACG, Guanacaste Province, 295–325 m elevation.

#### Ecology

Within ACG inventory, *Hyphantrophaga angustata* (van der Wulp) has been reared 10 times from two species of Lepidoptera in the family Thyrididae, *Dysodia speculifera* (Sepp, 1852) and *Dysodia immargo* Dyar, 1913, in dry forest and dry-rain lowland intergrade.

### Hyphantrophaga calixtomoragai

Fleming & Wood sp. n.

urn:lsid:zoobank.org:act:14DA9F7F-80ED-4D69-93CD-C185F76FC148

#### Materials

- **Type status:** Holotype. **Occurrence:** occurrenceDetails: http://janzen.sas.upenn.edu; catalogNumber: DHJPAR0007342; recordedBy: D.H. Janzen, W. Hallwachs & Freddy Quesada; individualID: DHJPAR0007342; individualCount: 1; sex: male; lifeStage: adult; preparations: pinned; otherCatalogNumbers: ASTAT114-06, 92-SRNP-1048.11, BOLD:ACE3368; **Taxon:** scientificName: Hyphantrophaga calixtomoragai; phylum: Arthropoda; class: Insecta; order: Diptera; family: Tachinidae; genus: Hyphantrophaga; specificEpithet: calixtomoragai; scientificNameAuthorship: Fleming & Wood, 2018; **Location:** continent: Central America; country: Costa Rica; countryCode: CR; stateProvince: Guanacaste; county: Sector Santa Rosa; locality: Area de Conservacion Guanacaste; verbatimLocality: Chiringon; verbatimElevation: 250; verbatimLatitude: 10.8388; verbatimLongitude: -85.601; verbatimCoordinateSystem: Decimal; decimalLatitude: 10.8388; decimalLongitude: -85.601; **Identification:** identifiedBy: AJ Fleming; dateIdentified: 2017; **Event:** samplingProtocol: Reared from the larva of the Erebidae, Melipotis perpendicularisDHJ02; verbatimEventDate: 24-Jun-1992; **Record Level:** language: en; institutionCode: CNC; collectionCode: Insects; basisOfRecord: Pinned Specimen
- **Type status:** Paratype. **Occurrence:** occurrenceDetails: http://janzen.sas.upenn.edu; catalogNumber: DHJPAR0007343; recordedBy: D.H. Janzen, W. Hallwachs & gusaneros; individualID: DHJPAR0007343; individualCount: 1; sex: female; lifeStage: adult; preparations: pinned; otherCatalogNumbers: ASTAT115-06, 91-SRNP-522, BOLD:ACE3368; **Taxon:** scientificName: Hyphantrophaga calixtomoragai; phylum: Arthropoda; class: Insecta; order: Diptera; family: Tachinidae; genus: Hyphantrophaga; specificEpithet: calixtomoragai; scientificNameAuthorship: Fleming & Wood, 2018; **Location:** continent: Central America; country: Costa Rica; countryCode: CR; stateProvince: Guanacaste; county: Sector Santa Rosa; locality: Area de Conservacion Guanacaste; verbatimLocality: Quebrada Costa Rica; verbatimElevation: 275; verbatimLatitude: 10.8274; verbatimLongitude: -85.6365; verbatimCoordinateSystem: Decimal; decimalLatitude: 10.8274; decimalLongitude: -85.6365; **Identification:** identifiedBy: AJ Fleming; dateIdentified: 2017; **Event:** samplingProtocol: Reared from the larva of the Erebidae, Metria leucoplaga; verbatimEventDate: 28-Apr-1992; **Record Level:** language: en; institutionCode: CNC; collectionCode: Insects; basisOfRecord: Pinned Specimen
- **Type status:** Paratype. **Occurrence:** occurrenceDetails: http://janzen.sas.upenn.edu; catalogNumber: DHJPAR0019834; recordedBy: D.H. Janzen, W. Hallwachs & Mariano Pereira; individualID: DHJPAR0019834; individualCount: 1; sex: male; lifeStage: adult; preparations: pinned; otherCatalogNumbers: ASTAB382-07, 06-SRNP-15096, BOLD:ACE3368; **Taxon:** scientificName: Hyphantrophaga calixtomoragai; phylum: Arthropoda; class: Insecta; order: Diptera; family: Tachinidae; genus: Hyphantrophaga; specificEpithet: calixtomoragai; scientificNameAuthorship: Fleming & Wood, 2018; **Location:** continent: Central America; country: Costa Rica; countryCode: CR; stateProvince: Guanacaste; county: Sector Santa Rosa; locality: Area de Conservacion Guanacaste; verbatimLocality: Vado Poza Salada; verbatimElevation: 8; verbatimLatitude: 10.798; verbatimLongitude: -85.6498; verbatimCoordinateSystem: Decimal; decimalLatitude: 10.798; decimalLongitude: -85.6498; **Identification:** identifiedBy: AJ Fleming; dateIdentified: 2017; **Event:** samplingProtocol: Reared from the larva of the Erebidae, Toxonprucha Poole01; verbatimEventDate: 08-Apr-2006; **Record Level:** language: en; institutionCode: CNC; collectionCode: Insects; basisOfRecord: Pinned Specimen
- **Type status:** Paratype. **Occurrence:** occurrenceDetails: http://janzen.sas.upenn.edu; catalogNumber: DHJPAR0052586; recordedBy: D.H. Janzen, W. Hallwachs & Mariano Pereira; individualID: DHJPAR0052586; individualCount: 1; sex: male; lifeStage: adult; preparations: pinned; otherCatalogNumbers: ASHYM1940-13, 13-SRNP-55372, BOLD:ACE3368; **Taxon:** scientificName: Hyphantrophaga calixtomoragai; phylum: Arthropoda; class: Insecta; order: Diptera; family: Tachinidae; genus: Hyphantrophaga; specificEpithet: calixtomoragai; scientificNameAuthorship: Fleming & Wood, 2018; **Location:** continent: Central America; country: Costa Rica; countryCode: CR; stateProvince: Guanacaste; county: Sector Mundo Nuevo; locality: Area de Conservacion Guanacaste; verbatimLocality: Estacion La Perla; verbatimElevation: 325; verbatimLatitude: 10.7674; verbatimLongitude: -85.4331; verbatimCoordinateSystem: Decimal; decimalLatitude: 10.7674; decimalLongitude: -85.4331; **Identification:** identifiedBy: AJ Fleming; dateIdentified: 2017; **Event:** samplingProtocol: Reared from the larva of the Erebidae, Ramphia albizona; verbatimEventDate: 08-Aug-2013; **Record Level:** language: en; institutionCode: CNC; collectionCode: Insects; basisOfRecord: Pinned Specimen
- **Type status:** Paratype. **Occurrence:** occurrenceDetails: http://janzen.sas.upenn.edu; catalogNumber: DHJPAR0055058; recordedBy: D.H. Janzen, W. Hallwachs & gusaneros; individualID: DHJPAR0055058; individualCount: 1; sex: male; lifeStage: adult; preparations: pinned; otherCatalogNumbers: ASHYH1605-14, 13-SRNP-55366, BOLD:ACE3368; **Taxon:** scientificName: Hyphantrophaga calixtomoragai; phylum: Arthropoda; class: Insecta; order: Diptera; family: Tachinidae; genus: Hyphantrophaga; specificEpithet: calixtomoragai; scientificNameAuthorship: Fleming & Wood, 2018; **Location:** continent: Central America; country: Costa Rica; countryCode: CR; stateProvince: Guanacaste; county: Sector Mundo Nuevo; locality: Area de Conservacion Guanacaste; verbatimLocality: Estacion La Perla; verbatimElevation: 325; verbatimLatitude: 10.7674; verbatimLongitude: -85.4331; verbatimCoordinateSystem: Decimal; decimalLatitude: 10.7674; decimalLongitude: -85.4331; **Identification:** identifiedBy: AJ Fleming; dateIdentified: 2017; **Event:** samplingProtocol: Reared from the larva of the Erebidae, Ramphia albizona; verbatimEventDate: 05-Mar-2014; **Record Level:** language: en; institutionCode: CNC; collectionCode: Insects; basisOfRecord: Pinned Specimen
- **Type status:** Paratype. **Occurrence:** occurrenceDetails: http://janzen.sas.upenn.edu; catalogNumber: DHJPAR0055059; recordedBy: D.H. Janzen, W. Hallwachs & Guillermo Pereira; individualID: DHJPAR0055059; individualCount: 1; sex: female; lifeStage: adult; preparations: pinned; otherCatalogNumbers: ASHYH1606-14, 13-SRNP-55364, BOLD:ACE3368; **Taxon:** scientificName: Hyphantrophaga calixtomoragai; phylum: Arthropoda; class: Insecta; order: Diptera; family: Tachinidae; genus: Hyphantrophaga; specificEpithet: calixtomoragai; scientificNameAuthorship: Fleming & Wood, 2018; **Location:** continent: Central America; country: Costa Rica; countryCode: CR; stateProvince: Guanacaste; county: Sector Mundo Nuevo; locality: Area de Conservacion Guanacaste; verbatimLocality: Estacion La Perla; verbatimElevation: 325; verbatimLatitude: 10.7674; verbatimLongitude: -85.4331; verbatimCoordinateSystem: Decimal; decimalLatitude: 10.7674; decimalLongitude: -85.4331; **Identification:** identifiedBy: AJ Fleming; dateIdentified: 2017; **Event:** samplingProtocol: Reared from the larva of the Erebidae, Ramphia albizona; verbatimEventDate: 22-Feb-2014; **Record Level:** language: en; institutionCode: CNC; collectionCode: Insects; basisOfRecord: Pinned Specimen
- **Type status:** Paratype. **Occurrence:** occurrenceDetails: http://janzen.sas.upenn.edu; catalogNumber: DHJPAR0055854; recordedBy: D.H. Janzen, W. Hallwachs & Freddy Quesada; individualID: DHJPAR0055854; individualCount: 1; sex: female; lifeStage: adult; preparations: pinned; otherCatalogNumbers: ASHYH2586-14, 14-SRNP-55795, BOLD:ACE3368; **Taxon:** scientificName: Hyphantrophaga calixtomoragai; phylum: Arthropoda; class: Insecta; order: Diptera; family: Tachinidae; genus: Hyphantrophaga; specificEpithet: calixtomoragai; scientificNameAuthorship: Fleming & Wood, 2018; **Location:** continent: Central America; country: Costa Rica; countryCode: CR; stateProvince: Guanacaste; county: Sector Mundo Nuevo; locality: Area de Conservacion Guanacaste; verbatimLocality: Sendero Aguacate; verbatimElevation: 335; verbatimLatitude: 10.769; verbatimLongitude: -85.4346; verbatimCoordinateSystem: Decimal; decimalLatitude: 10.769; decimalLongitude: -85.4346; **Identification:** identifiedBy: AJ Fleming; dateIdentified: 2017; **Event:** samplingProtocol: Reared from the larva of the Erebidae, Smyra stipatura; verbatimEventDate: 31-Jul-2014; **Record Level:** language: en; institutionCode: CNC; collectionCode: Insects; basisOfRecord: Pinned Specimen
- **Type status:** Paratype. **Occurrence:** occurrenceDetails: http://janzen.sas.upenn.edu; catalogNumber: DHJPAR0055856; recordedBy: D.H. Janzen, W. Hallwachs & Harry Ramirez; individualID: DHJPAR0055856; individualCount: 1; sex: male; lifeStage: adult; preparations: pinned; otherCatalogNumbers: ASHYH2588-14, 14-SRNP-55796, BOLD:ACE3368; **Taxon:** scientificName: Hyphantrophaga calixtomoragai; phylum: Arthropoda; class: Insecta; order: Diptera; family: Tachinidae; genus: Hyphantrophaga; specificEpithet: calixtomoragai; scientificNameAuthorship: Fleming & Wood, 2018; **Location:** continent: Central America; country: Costa Rica; countryCode: CR; stateProvince: Guanacaste; county: Sector Mundo Nuevo; locality: Area de Conservacion Guanacaste; verbatimLocality: Sendero Aguacate; verbatimElevation: 335; verbatimLatitude: 10.769; verbatimLongitude: -85.4346; verbatimCoordinateSystem: Decimal; decimalLatitude: 10.769; decimalLongitude: -85.4346; **Identification:** identifiedBy: AJ Fleming; dateIdentified: 2017; **Event:** samplingProtocol: Reared from the larva of the Erebidae, Smyra stipatura; verbatimEventDate: 03-Jul-2014; **Record Level:** language: en; institutionCode: CNC; collectionCode: Insects; basisOfRecord: Pinned Specimen

#### Description

**Male** (Fig. 6). Length: 8–12 mm. **Head** (Fig. 6b): vertex 1/4 of head width; two pairs of reclinate upper orbital setae; ocellar setae arising behind anterior ocellus; ocellar triangle gold; fronto-orbital plate brilliant silver and setulose, setulae not extending below lowest frontal seta; parafacial shiny silver and bare; eye densely haired; facial ridge bare; pedicel orange, postpedicel dark brown-black; arista brown, very minutely pubescent, distinctly thickened on basal 1/3–1/4; palpus orange, clubbed and densely haired apically. **Thorax** (Fig. 6a, c): silver tomentose dorsally and laterally; densely covered with many short black setulae along anterior and lateral surfaces, interspersed amongst the setae; four thin dorsal vittae, outermost two broken across suture, innermost pair unbroken, almost reaching 2nd postsutural dorsocentral seta; postpronotum with 4–5 setae arranged in a triangle; chaetotaxy: acrostichal setae 3:3; dorsocentral setae 3–4:4; intra-alar setae 3–4:3; supra-alar setae 2:3; three katepisternal setae; basal scutellar setae curving inwards medially, as long as subapical setae; lateral scutellar setae less than 1/2 as long as subapical scutellar setae; apical scutellar setae 1/3 as long as subapical setae, crossed apically; one pair of discal scutellar setae set as widely apart as subapical scutellar setae; scutellum concolorous with scutum. **Legs** (Fig. 6c): brilliant yellow in ground colour except femora which appear reddish-brown; fore femur with dense silver tomentum on posterodorsal surface; hind coxa setose. **Wing** (Fig. 6a): pale translucent, hyaline, not distinctly infuscate; vein R_4+5_ with only 2–3 setulae at base. **Abdomen** (Fig. 6a, c): ground colour dark brown-black; middorsal depression on ST1+2 almost reaching hind margin; median marginal setae reduced but present on ST1+2, strong on T3; a complete row of marginal setae present on T4; discal setae only on T5; sex patch indistinct/absent; silver tomentose bands present along anterior edge of T3 and T4, unbroken medially and covering almost 90% of tergal surface; T5 with silver tomentum throughout. **Terminalia** (Fig. 6d, e, f): sternite 5 (Fig. 6f) with a deeply excavated median cleft, smoothly U-shaped, margins covered in dense tomentum. Lateral lobes of sternite rounded apically, devoid of any strong setae. Anterior plate of sternite 5 from subequal to slightly shorter than apical lobes; unsclerotised "window" anterior to median cleft rectangular, narrow and as wide as median cleft. Cerci in posterior view (Fig. 6d) subrectangular and slightly shorter than surstyli, blunt and slightly clubbed at apex, completely separate medially but not significantly divergent; in lateral view straight with a blunt apical hook slightly downwardly curved; densely setulose along entire length dorsally, setulose ventrally along entire length (visible in lateral view). Surstylus in lateral view (Fig. 6e) almost parallel-sided along its length, ending in a slightly pointed apex, making the structure appear blade-like; when viewed dorsally, surstyli appearing to point outwards, not strongly convergent. Pregonite broad and well-developed, tapering apically to a rounded, slightly blunted apex; devoid of setulae. Postgonite narrow, up to 1/2 as wide as pregonite, sharply pointed and curved at apex, scythe-like and subequal in length to pregonite. Epiphallus well-developed and apically hooked. Distiphallus rectangular in shape, with a slender median longitudinal sclerotised reinforcement on its posterior surface and a broad, anterolateral, sclerotised acrophallus, joining on both sides of anterior surface near apex.

**Female**. Length: 9–12 mm. As male, differing only by the presence of two pairs of proclinate orbital setae.

#### Diagnosis

*Hyphantrophaga calixtomoragai* **sp. n.** can be distinguished from all other *Hyphantrophaga* species by the following combination of traits: ocellar triangle gold, fronto-orbital plate silver and setulose, pedicel orange, three katepisternal setae, hind coxa setose, abdominal tomentum silver throughout, median marginal setae present on ST1+2, discal setae absent from T3 and T4 and sex patch absent.

#### Etymology

*Hyphantrophaga calixtomoragai* **sp. n.** is named in recognition of Calixto Moraga Medina's dedication and work at finding and rearing the ACG caterpillars that contained tachinid larvae.

#### Distribution

Costa Rica, ACG, Guanacaste Province, 8–215 m elevation.

#### Ecology

*Hyphantrophaga calixtomoragai* **sp. n.** has been reared six times from six different species of Lepidoptera in the family Erebidae, *Metria leucoplaga* (Hampson, 1910), *Melipotis* perpendicularisDHJ02, *Toxonprucha* Poole01, *Abacena accincta* Felder & Rogenhofer, 1874, *Ramphia albizona* (Latreille, 1817) and *Smyra stipatura* (Walker, 1858); in dry forest and dry-rain lowland intergrades.

### Hyphantrophaga calva

Fleming & Wood sp. n.

urn:lsid:zoobank.org:act:27A02B9A-6716-4EC2-B2B7-7129B6466B27

#### Materials

- **Type status:** Holotype. **Occurrence:** occurrenceDetails: http://janzen.sas.upenn.edu; catalogNumber: DHJPAR0019630; recordedBy: D.H. Janzen, W. Hallwachs & Osvaldo Espinoza; individualID: DHJPAR0019630; individualCount: 1; sex: male; lifeStage: adult; preparations: pinned; otherCatalogNumbers: ASTAB178-07, 07-SRNP-2267, BOLD:AAC5447; **Taxon:** scientificName: Hyphantrophaga calva; phylum: Arthropoda; class: Insecta; order: Diptera; family: Tachinidae; genus: Hyphantrophaga; specificEpithet: calva; scientificNameAuthorship: Fleming & Wood, 2018; **Location:** continent: Central America; country: Costa Rica; countryCode: CR; stateProvince: Alajuela; county: Sector San Cristobal; locality: Area de Conservacion Guanacaste; verbatimLocality: Puente Palma; verbatimElevation: 460; verbatimLatitude: 10.9163; verbatimLongitude: -85.3787; verbatimCoordinateSystem: Decimal; decimalLatitude: 10.9163; decimalLongitude: -85.3787; **Identification:** identifiedBy: AJ Fleming; dateIdentified: 2017; **Event:** samplingProtocol: Reared from the larvae of the Depressariidae, Anadasmus Janzen11; verbatimEventDate: 14-Jun-2007; **Record Level:** language: en; institutionCode: CNC; collectionCode: Insects; basisOfRecord: Pinned Specimen
- **Type status:** Paratype. **Occurrence:** occurrenceDetails: http://janzen.sas.upenn.edu; catalogNumber: DHJPAR0006678; recordedBy: D.H. Janzen, W. Hallwachs & Lucia Rios; individualID: DHJPAR0006678; individualCount: 1; sex: male; lifeStage: adult; preparations: pinned; otherCatalogNumbers: ASTA856-06, 05-SRNP-25287, BOLD:AAC5447; **Taxon:** scientificName: Hyphantrophaga calva; phylum: Arthropoda; class: Insecta; order: Diptera; family: Tachinidae; genus: Hyphantrophaga; specificEpithet: calva; scientificNameAuthorship: Fleming & Wood, 2018; **Location:** continent: Central America; country: Costa Rica; countryCode: CR; stateProvince: Guanacaste; county: Sector Del Oro; locality: Area de Conservacion Guanacaste; verbatimLocality: Quebrada Oro; verbatimElevation: 290; verbatimLatitude: 11.0338; verbatimLongitude: -85.4771; verbatimCoordinateSystem: Decimal; decimalLatitude: 11.0338; decimalLongitude: -85.4771; **Identification:** identifiedBy: AJ Fleming; dateIdentified: 2017; **Event:** samplingProtocol: Reared from the larvae of the Depressariidae, Anadasmus Janzen11; verbatimEventDate: 22-Dec-2005; **Record Level:** language: en; institutionCode: CNC; collectionCode: Insects; basisOfRecord: Pinned Specimen
- **Type status:** Paratype. **Occurrence:** occurrenceDetails: http://janzen.sas.upenn.edu; catalogNumber: DHJPAR0007491; recordedBy: D.H. Janzen, W. Hallwachs & Minor Carmona; individualID: DHJPAR0007491; individualCount: 1; sex: male; lifeStage: adult; preparations: pinned; otherCatalogNumbers: ASTAT263-06, 04-SRNP-40127, BOLD:AAC5447; **Taxon:** scientificName: Hyphantrophaga calva; phylum: Arthropoda; class: Insecta; order: Diptera; family: Tachinidae; genus: Hyphantrophaga; specificEpithet: calva; scientificNameAuthorship: Fleming & Wood, 2018; **Location:** continent: Central America; country: Costa Rica; countryCode: CR; stateProvince: Alajuela; county: Sector Rincon Rain Forest; locality: Area de Conservacion Guanacaste; verbatimLocality: Puente Rio Negro; verbatimElevation: 340; verbatimLatitude: 10.9038; verbatimLongitude: -85.3027; verbatimCoordinateSystem: Decimal; decimalLatitude: 10.9038; decimalLongitude: -85.3027; **Identification:** identifiedBy: AJ Fleming; dateIdentified: 2017; **Event:** samplingProtocol: Reared from the larvae of the Depressariidae, Cerconota Janzen102; verbatimEventDate: 13-Feb-2004; **Record Level:** language: en; institutionCode: CNC; collectionCode: Insects; basisOfRecord: Pinned Specimen
- **Type status:** Paratype. **Occurrence:** occurrenceDetails: http://janzen.sas.upenn.edu; catalogNumber: DHJPAR0007492; recordedBy: D.H. Janzen, W. Hallwachs & Minor Carmona; individualID: DHJPAR0007492; individualCount: 1; sex: male; lifeStage: adult; preparations: pinned; otherCatalogNumbers: ASTAT264-06, 04-SRNP-40125, BOLD:AAC5447; **Taxon:** scientificName: Hyphantrophaga calva; phylum: Arthropoda; class: Insecta; order: Diptera; family: Tachinidae; genus: Hyphantrophaga; specificEpithet: calva; scientificNameAuthorship: Fleming & Wood, 2018; **Location:** continent: Central America; country: Costa Rica; countryCode: CR; stateProvince: Alajuela; county: Sector Rincon Rain Forest; locality: Area de Conservacion Guanacaste; verbatimLocality: Puente Rio Negro; verbatimElevation: 340; verbatimLatitude: 10.9038; verbatimLongitude: -85.3027; verbatimCoordinateSystem: Decimal; decimalLatitude: 10.9038; decimalLongitude: -85.3027; **Identification:** identifiedBy: AJ Fleming; dateIdentified: 2017; **Event:** samplingProtocol: Reared from the larvae of the Depressariidae, Cerconota Janzen102; verbatimEventDate: 14-Feb-2004; **Record Level:** language: en; institutionCode: CNC; collectionCode: Insects; basisOfRecord: Pinned Specimen
- **Type status:** Paratype. **Occurrence:** occurrenceDetails: http://janzen.sas.upenn.edu; catalogNumber: DHJPAR0007493; recordedBy: D.H. Janzen, W. Hallwachs & Minor Carmona; individualID: DHJPAR0007493; individualCount: 1; sex: female; lifeStage: adult; preparations: pinned; otherCatalogNumbers: ASTAT265-06, 04-SRNP-40128, BOLD:AAC5447; **Taxon:** scientificName: Hyphantrophaga calva; phylum: Arthropoda; class: Insecta; order: Diptera; family: Tachinidae; genus: Hyphantrophaga; specificEpithet: calva; scientificNameAuthorship: Fleming & Wood, 2018; **Location:** continent: Central America; country: Costa Rica; countryCode: CR; stateProvince: Alajuela; county: Sector Rincon Rain Forest; locality: Area de Conservacion Guanacaste; verbatimLocality: Puente Rio Negro; verbatimElevation: 340; verbatimLatitude: 10.9038; verbatimLongitude: -85.3027; verbatimCoordinateSystem: Decimal; decimalLatitude: 10.9038; decimalLongitude: -85.3027; **Identification:** identifiedBy: AJ Fleming; dateIdentified: 2017; **Event:** samplingProtocol: Reared from the larvae of the Depressariidae, Cerconota Janzen102; verbatimEventDate: 24-Feb-2004; **Record Level:** language: en; institutionCode: CNC; collectionCode: Insects; basisOfRecord: Pinned Specimen
- **Type status:** Paratype. **Occurrence:** occurrenceDetails: http://janzen.sas.upenn.edu; catalogNumber: DHJPAR0007494; recordedBy: D.H. Janzen, W. Hallwachs & Jose Perez; individualID: DHJPAR0007494; individualCount: 1; sex: male; lifeStage: adult; preparations: pinned; otherCatalogNumbers: ASTAT266-06, 04-SRNP-40543, BOLD:AAC5447; **Taxon:** scientificName: Hyphantrophaga calva; phylum: Arthropoda; class: Insecta; order: Diptera; family: Tachinidae; genus: Hyphantrophaga; specificEpithet: calva; scientificNameAuthorship: Fleming & Wood, 2018; **Location:** continent: Central America; country: Costa Rica; countryCode: CR; stateProvince: Alajuela; county: Sector Rincon Rain Forest; locality: Area de Conservacion Guanacaste; verbatimLocality: San Lucas; verbatimElevation: 320; verbatimLatitude: 10.9185; verbatimLongitude: -85.3034; verbatimCoordinateSystem: Decimal; decimalLatitude: 10.9185; decimalLongitude: -85.3034; **Identification:** identifiedBy: AJ Fleming; dateIdentified: 2017; **Event:** samplingProtocol: Reared from the larvae of the Depressariidae, Cerconota Janzen102; verbatimEventDate: 31-Mar-2004; **Record Level:** language: en; institutionCode: CNC; collectionCode: Insects; basisOfRecord: Pinned Specimen
- **Type status:** Paratype. **Occurrence:** occurrenceDetails: http://janzen.sas.upenn.edu; catalogNumber: DHJPAR0007495; recordedBy: D.H. Janzen, W. Hallwachs & Gloria Sihezar; individualID: DHJPAR0007495; individualCount: 1; sex: female; lifeStage: adult; preparations: pinned; otherCatalogNumbers: ASTAT267-06, 04-SRNP-60321, BOLD:AAC5447; **Taxon:** scientificName: Hyphantrophaga calva; phylum: Arthropoda; class: Insecta; order: Diptera; family: Tachinidae; genus: Hyphantrophaga; specificEpithet: calva; scientificNameAuthorship: Fleming & Wood, 2018; **Location:** continent: Central America; country: Costa Rica; countryCode: CR; stateProvince: Alajuela; county: Sector San Cristobal; locality: Area de Conservacion Guanacaste; verbatimLocality: Puente Palma; verbatimElevation: 460; verbatimLatitude: 10.9163; verbatimLongitude: -85.3787; verbatimCoordinateSystem: Decimal; decimalLatitude: 10.9163; decimalLongitude: -85.3787; **Identification:** identifiedBy: AJ Fleming; dateIdentified: 2017; **Event:** samplingProtocol: Reared from the larvae of the Depressariidae, Anadasmus Janzen11; verbatimEventDate: 10-Nov-2004; **Record Level:** language: en; institutionCode: CNC; collectionCode: Insects; basisOfRecord: Pinned Specimen
- **Type status:** Paratype. **Occurrence:** occurrenceDetails: http://janzen.sas.upenn.edu; catalogNumber: DHJPAR0007496; recordedBy: D.H. Janzen, W. Hallwachs & Elda Araya; individualID: DHJPAR0007496; individualCount: 1; sex: female; lifeStage: adult; preparations: pinned; otherCatalogNumbers: ASTAT268-06, 04-SRNP-4735, BOLD:AAC5447; **Taxon:** scientificName: Hyphantrophaga calva; phylum: Arthropoda; class: Insecta; order: Diptera; family: Tachinidae; genus: Hyphantrophaga; specificEpithet: calva; scientificNameAuthorship: Fleming & Wood, 2018; **Location:** continent: Central America; country: Costa Rica; countryCode: CR; stateProvince: Alajuela; county: Sector San Cristobal; locality: Area de Conservacion Guanacaste; verbatimLocality: Sendero Corredor; verbatimElevation: 620; verbatimLatitude: 10.8787; verbatimLongitude: -85.3896; verbatimCoordinateSystem: Decimal; decimalLatitude: 10.8787; decimalLongitude: -85.3896; **Identification:** identifiedBy: AJ Fleming; dateIdentified: 2017; **Event:** samplingProtocol: Reared from the larvae of the Depressariidae, Anadasmus Janzen25; verbatimEventDate: 23-Oct-2004; **Record Level:** language: en; institutionCode: CNC; collectionCode: Insects; basisOfRecord: Pinned Specimen
- **Type status:** Paratype. **Occurrence:** occurrenceDetails: http://janzen.sas.upenn.edu; catalogNumber: DHJPAR0008084; recordedBy: D.H. Janzen, W. Hallwachs & Minor Carmona; individualID: DHJPAR0008084; individualCount: 1; sex: female; lifeStage: adult; preparations: pinned; otherCatalogNumbers: ASTAT856-06, 05-SRNP-41074, BOLD:AAC5447; **Taxon:** scientificName: Hyphantrophaga calva; phylum: Arthropoda; class: Insecta; order: Diptera; family: Tachinidae; genus: Hyphantrophaga; specificEpithet: calva; scientificNameAuthorship: Fleming & Wood, 2018; **Location:** continent: Central America; country: Costa Rica; countryCode: CR; stateProvince: Alajuela; county: Sector Rincon Rain Forest; locality: Area de Conservacion Guanacaste; verbatimLocality: Estacion Caribe; verbatimElevation: 415; verbatimLatitude: 10.9019; verbatimLongitude: -85.2749; verbatimCoordinateSystem: Decimal; decimalLatitude: 10.9019; decimalLongitude: -85.2749; **Identification:** identifiedBy: AJ Fleming; dateIdentified: 2017; **Event:** samplingProtocol: Reared from the larvae of the Depressariidae, Anadasmus Janzen11; verbatimEventDate: 20-May-2005; **Record Level:** language: en; institutionCode: CNC; collectionCode: Insects; basisOfRecord: Pinned Specimen
- **Type status:** Paratype. **Occurrence:** occurrenceDetails: http://janzen.sas.upenn.edu; catalogNumber: DHJPAR0003327; recordedBy: D.H. Janzen, W. Hallwachs & Carolina Cano; individualID: DHJPAR0003327; individualCount: 1; sex: female; lifeStage: adult; preparations: pinned; otherCatalogNumbers: ASTA234-05, 03-SRNP-13029.1, BOLD:AAC5447; **Taxon:** scientificName: Hyphantrophaga calva; phylum: Arthropoda; class: Insecta; order: Diptera; family: Tachinidae; genus: Hyphantrophaga; specificEpithet: calva; scientificNameAuthorship: Fleming & Wood, 2018; **Location:** continent: Central America; country: Costa Rica; countryCode: CR; stateProvince: Alajuela; county: Sector Rincon Rain Forest; locality: Area de Conservacion Guanacaste; verbatimLocality: San Lucas; verbatimElevation: 320; verbatimLatitude: 10.9185; verbatimLongitude: -85.3034; verbatimCoordinateSystem: Decimal; decimalLatitude: 10.9185; decimalLongitude: -85.3034; **Identification:** identifiedBy: AJ Fleming; dateIdentified: 2017; **Event:** samplingProtocol: Reared from the larvae of the Depressariidae, Anadasmus Janzen11; verbatimEventDate: 12-Nov-2003; **Record Level:** language: en; institutionCode: CNC; collectionCode: Insects; basisOfRecord: Pinned Specimen
- **Type status:** Paratype. **Occurrence:** occurrenceDetails: http://janzen.sas.upenn.edu; catalogNumber: DHJPAR0021046; recordedBy: D.H. Janzen, W. Hallwachs & Duvalier Briceno; individualID: DHJPAR0021046; individualCount: 1; sex: female; lifeStage: adult; preparations: pinned; otherCatalogNumbers: ASTA1389-07, 07-SRNP-65192, BOLD:AAC5447; **Taxon:** scientificName: Hyphantrophaga calva; phylum: Arthropoda; class: Insecta; order: Diptera; family: Tachinidae; genus: Hyphantrophaga; specificEpithet: calva; scientificNameAuthorship: Fleming & Wood, 2018; **Location:** continent: Central America; country: Costa Rica; countryCode: CR; stateProvince: Alajuela; county: Brasilia; locality: Area de Conservacion Guanacaste; verbatimLocality: Brisanta; verbatimElevation: 290; verbatimLatitude: 11.0274; verbatimLongitude: -85.3368; verbatimCoordinateSystem: Decimal; decimalLatitude: 11.0274; decimalLongitude: -85.3368; **Identification:** identifiedBy: AJ Fleming; dateIdentified: 2017; **Event:** samplingProtocol: Reared from the larvae of the Depressariidae, Cerconota Janzen707; verbatimEventDate: 30-Jul-2007; **Record Level:** language: en; institutionCode: CNC; collectionCode: Insects; basisOfRecord: Pinned Specimen
- **Type status:** Paratype. **Occurrence:** occurrenceDetails: http://janzen.sas.upenn.edu; catalogNumber: DHJPAR0035699; recordedBy: D.H. Janzen, W. Hallwachs & Pablo Umaña Calderon; individualID: DHJPAR0035699; individualCount: 1; sex: female; lifeStage: adult; preparations: pinned; otherCatalogNumbers: ASHYD1080-09, 09-SRNP-41475, BOLD:AAC5447; **Taxon:** scientificName: Hyphantrophaga calva; phylum: Arthropoda; class: Insecta; order: Diptera; family: Tachinidae; genus: Hyphantrophaga; specificEpithet: calva; scientificNameAuthorship: Fleming & Wood, 2018; **Location:** continent: Central America; country: Costa Rica; countryCode: CR; stateProvince: Alajuela; county: Sector Rincon Rain Forest; locality: Area de Conservacion Guanacaste; verbatimLocality: Estacion Caribe; verbatimElevation: 415; verbatimLatitude: 10.9019; verbatimLongitude: -85.2749; verbatimCoordinateSystem: Decimal; decimalLatitude: 10.9019; decimalLongitude: -85.2749; **Identification:** identifiedBy: AJ Fleming; dateIdentified: 2017; **Event:** samplingProtocol: Reared from the larvae of the Depressariidae, Anadasmus Janzen11; verbatimEventDate: 18-Jul-2009; **Record Level:** language: en; institutionCode: CNC; collectionCode: Insects; basisOfRecord: Pinned Specimen
- **Type status:** Paratype. **Occurrence:** occurrenceDetails: http://janzen.sas.upenn.edu; catalogNumber: DHJPAR0040132; recordedBy: D.H. Janzen, W. Hallwachs & Jose Perez; individualID: DHJPAR0040132; individualCount: 1; sex: female; lifeStage: adult; preparations: pinned; otherCatalogNumbers: ASHYE2299-11, 10-SRNP-42130, BOLD:AAC5447; **Taxon:** scientificName: Hyphantrophaga calva; phylum: Arthropoda; class: Insecta; order: Diptera; family: Tachinidae; genus: Hyphantrophaga; specificEpithet: calva; scientificNameAuthorship: Fleming & Wood, 2018; **Location:** continent: Central America; country: Costa Rica; countryCode: CR; stateProvince: Alajuela; county: Sector Rincon Rain Forest; locality: Area de Conservacion Guanacaste; verbatimLocality: Sendero Parcelas; verbatimElevation: 375; verbatimLatitude: 10.9078; verbatimLongitude: -85.2914; verbatimCoordinateSystem: Decimal; decimalLatitude: 10.9078; decimalLongitude: -85.2914; **Identification:** identifiedBy: AJ Fleming; dateIdentified: 2017; **Event:** samplingProtocol: Reared from the larvae of the Depressariidae, Anadasmus Janzen11; verbatimEventDate: 18-Jul-2010; **Record Level:** language: en; institutionCode: CNC; collectionCode: Insects; basisOfRecord: Pinned Specimen
- **Type status:** Paratype. **Occurrence:** occurrenceDetails: http://janzen.sas.upenn.edu; catalogNumber: DHJPAR0042269; recordedBy: D.H. Janzen, W. Hallwachs & Ricardo Calero; individualID: DHJPAR0042269; individualCount: 1; sex: female; lifeStage: adult; preparations: pinned; otherCatalogNumbers: ASHYH033-11, 11-SRNP-70323, BOLD:AAC5447; **Taxon:** scientificName: Hyphantrophaga calva; phylum: Arthropoda; class: Insecta; order: Diptera; family: Tachinidae; genus: Hyphantrophaga; specificEpithet: calva; scientificNameAuthorship: Fleming & Wood, 2018; **Location:** continent: Central America; country: Costa Rica; countryCode: CR; stateProvince: Guanacaste; county: Sector Pitilla; locality: Area de Conservacion Guanacaste; verbatimLocality: Medrano; verbatimElevation: 380; verbatimLatitude: 11.016; verbatimLongitude: -85.3805; verbatimCoordinateSystem: Decimal; decimalLatitude: 11.016; decimalLongitude: -85.3805; **Identification:** identifiedBy: AJ Fleming; dateIdentified: 2017; **Event:** samplingProtocol: Reared from the larvae of the Depressariidae, Anadasmus Janzen11; verbatimEventDate: 18-Mar-2011; **Record Level:** language: en; institutionCode: CNC; collectionCode: Insects; basisOfRecord: Pinned Specimen
- **Type status:** Paratype. **Occurrence:** occurrenceDetails: http://janzen.sas.upenn.edu; catalogNumber: DHJPAR0042298; recordedBy: D.H. Janzen, W. Hallwachs & Duvalier Briceno; individualID: DHJPAR0042298; individualCount: 1; sex: male; lifeStage: adult; preparations: pinned; otherCatalogNumbers: ASHYH062-11, 11-SRNP-65093, BOLD:AAC5447; **Taxon:** scientificName: Hyphantrophaga calva; phylum: Arthropoda; class: Insecta; order: Diptera; family: Tachinidae; genus: Hyphantrophaga; specificEpithet: calva; scientificNameAuthorship: Fleming & Wood, 2018; **Location:** continent: Central America; country: Costa Rica; countryCode: CR; stateProvince: Guanacaste; county: Sector Pitilla; locality: Area de Conservacion Guanacaste; verbatimLocality: Bullas; verbatimElevation: 440; verbatimLatitude: 10.9867; verbatimLongitude: -85.385; verbatimCoordinateSystem: Decimal; decimalLatitude: 10.9867; decimalLongitude: -85.385; **Identification:** identifiedBy: AJ Fleming; dateIdentified: 2017; **Event:** samplingProtocol: Reared from the larvae of the Depressariidae, Anadasmus Janzen11; verbatimEventDate: 09-Mar-2011; **Record Level:** language: en; institutionCode: CNC; collectionCode: Insects; basisOfRecord: Pinned Specimen
- **Type status:** Paratype. **Occurrence:** occurrenceDetails: http://janzen.sas.upenn.edu; catalogNumber: DHJPAR0045566; recordedBy: D.H. Janzen, W. Hallwachs & Anabelle Cordoba; individualID: DHJPAR0045566; individualCount: 1; sex: male; lifeStage: adult; preparations: pinned; otherCatalogNumbers: ACGAZ755-11, 11-SRNP-43978, BOLD:AAC5447; **Taxon:** scientificName: Hyphantrophaga calva; phylum: Arthropoda; class: Insecta; order: Diptera; family: Tachinidae; genus: Hyphantrophaga; specificEpithet: calva; scientificNameAuthorship: Fleming & Wood, 2018; **Location:** continent: Central America; country: Costa Rica; countryCode: CR; stateProvince: Alajuela; county: Sector Rincon Rain Forest; locality: Area de Conservacion Guanacaste; verbatimLocality: San Lucas; verbatimElevation: 320; verbatimLatitude: 10.9185; verbatimLongitude: -85.3034; verbatimCoordinateSystem: Decimal; decimalLatitude: 10.9185; decimalLongitude: -85.3034; **Identification:** identifiedBy: AJ Fleming; dateIdentified: 2017; **Event:** samplingProtocol: Reared from the larvae of the Depressariidae, Anadasmus Janzen11; verbatimEventDate: 12-Oct-2011; **Record Level:** language: en; institutionCode: CNC; collectionCode: Insects; basisOfRecord: Pinned Specimen
- **Type status:** Paratype. **Occurrence:** occurrenceDetails: http://janzen.sas.upenn.edu; catalogNumber: DHJPAR0045573; recordedBy: D.H. Janzen, W. Hallwachs & Anabelle Cordoba; individualID: DHJPAR0045573; individualCount: 1; sex: male; lifeStage: adult; preparations: pinned; otherCatalogNumbers: ACGAZ762-11, 11-SRNP-43974, BOLD:AAC5447; **Taxon:** scientificName: Hyphantrophaga calva; phylum: Arthropoda; class: Insecta; order: Diptera; family: Tachinidae; genus: Hyphantrophaga; specificEpithet: calva; scientificNameAuthorship: Fleming & Wood, 2018; **Location:** continent: Central America; country: Costa Rica; countryCode: CR; stateProvince: Alajuela; county: Sector Rincon Rain Forest; locality: Area de Conservacion Guanacaste; verbatimLocality: San Lucas; verbatimElevation: 320; verbatimLatitude: 10.9185; verbatimLongitude: -85.3034; verbatimCoordinateSystem: Decimal; decimalLatitude: 10.9185; decimalLongitude: -85.3034; **Identification:** identifiedBy: AJ Fleming; dateIdentified: 2017; **Event:** samplingProtocol: Reared from the larvae of the Depressariidae, Anadasmus Janzen11; verbatimEventDate: 29-Sep-2011; **Record Level:** language: en; institutionCode: CNC; collectionCode: Insects; basisOfRecord: Pinned Specimen
- **Type status:** Paratype. **Occurrence:** occurrenceDetails: http://janzen.sas.upenn.edu; catalogNumber: DHJPAR0046412; recordedBy: D.H. Janzen, W. Hallwachs & Osvaldo Espinoza; individualID: DHJPAR0046412; individualCount: 1; sex: male; lifeStage: adult; preparations: pinned; otherCatalogNumbers: ACGBA585-12, 11-SRNP-4193, BOLD:AAC5447; **Taxon:** scientificName: Hyphantrophaga calva; phylum: Arthropoda; class: Insecta; order: Diptera; family: Tachinidae; genus: Hyphantrophaga; specificEpithet: calva; scientificNameAuthorship: Fleming & Wood, 2018; **Location:** continent: Central America; country: Costa Rica; countryCode: CR; stateProvince: Alajuela; county: Sector Rincon Rain Forest; locality: Area de Conservacion Guanacaste; verbatimLocality: Camino Albergue Oscar; verbatimElevation: 560; verbatimLatitude: 10.8774; verbatimLongitude: -85.3236; verbatimCoordinateSystem: Decimal; decimalLatitude: 10.8774; decimalLongitude: -85.3236; **Identification:** identifiedBy: AJ Fleming; dateIdentified: 2017; **Event:** samplingProtocol: Reared from the larvae of the Depressariidae, Anadasmus Janzen11; verbatimEventDate: 22-Nov-2011; **Record Level:** language: en; institutionCode: CNC; collectionCode: Insects; basisOfRecord: Pinned Specimen
- **Type status:** Paratype. **Occurrence:** occurrenceDetails: http://janzen.sas.upenn.edu; catalogNumber: DHJPAR0049599; recordedBy: D.H. Janzen, W. Hallwachs & Anabelle Cordoba; individualID: DHJPAR0049599; individualCount: 1; sex: male; lifeStage: adult; preparations: pinned; otherCatalogNumbers: ASHYB2393-12, 12-SRNP-44077, BOLD:AAC5447; **Taxon:** scientificName: Hyphantrophaga calva; phylum: Arthropoda; class: Insecta; order: Diptera; family: Tachinidae; genus: Hyphantrophaga; specificEpithet: calva; scientificNameAuthorship: Fleming & Wood, 2018; **Location:** continent: Central America; country: Costa Rica; countryCode: CR; stateProvince: Alajuela; county: Sector Rincon Rain Forest; locality: Area de Conservacion Guanacaste; verbatimLocality: San Lucas; verbatimElevation: 320; verbatimLatitude: 10.9185; verbatimLongitude: -85.3034; verbatimCoordinateSystem: Decimal; decimalLatitude: 10.9185; decimalLongitude: -85.3034; **Identification:** identifiedBy: AJ Fleming; dateIdentified: 2017; **Event:** samplingProtocol: Reared from the larvae of the Depressariidae, Anadasmus Janzen11; verbatimEventDate: 25-Aug-2012; **Record Level:** language: en; institutionCode: CNC; collectionCode: Insects; basisOfRecord: Pinned Specimen
- **Type status:** Paratype. **Occurrence:** occurrenceDetails: http://janzen.sas.upenn.edu; catalogNumber: DHJPAR0050265; recordedBy: D.H. Janzen, W. Hallwachs & Anabelle Cordoba; individualID: DHJPAR0050265; individualCount: 1; sex: female; lifeStage: adult; preparations: pinned; otherCatalogNumbers: ACGAZ1579-12, 12-SRNP-44076, BOLD:AAC5447; **Taxon:** scientificName: Hyphantrophaga calva; phylum: Arthropoda; class: Insecta; order: Diptera; family: Tachinidae; genus: Hyphantrophaga; specificEpithet: calva; scientificNameAuthorship: Fleming & Wood, 2018; **Location:** continent: Central America; country: Costa Rica; countryCode: CR; stateProvince: Alajuela; county: Sector Rincon Rain Forest; locality: Area de Conservacion Guanacaste; verbatimLocality: San Lucas; verbatimElevation: 320; verbatimLatitude: 10.9185; verbatimLongitude: -85.3034; verbatimCoordinateSystem: Decimal; decimalLatitude: 10.9185; decimalLongitude: -85.3034; **Identification:** identifiedBy: AJ Fleming; dateIdentified: 2017; **Event:** samplingProtocol: Reared from the larvae of the Depressariidae, Anadasmus Janzen11; verbatimEventDate: 26-Aug-2012; **Record Level:** language: en; institutionCode: CNC; collectionCode: Insects; basisOfRecord: Pinned Specimen
- **Type status:** Paratype. **Occurrence:** occurrenceDetails: http://janzen.sas.upenn.edu; catalogNumber: DHJPAR0050273; recordedBy: D.H. Janzen, W. Hallwachs & Anabelle Cordoba; individualID: DHJPAR0050273; individualCount: 1; sex: female; lifeStage: adult; preparations: pinned; otherCatalogNumbers: ACGAZ1587-12, 12-SRNP-44078, BOLD:AAC5447; **Taxon:** scientificName: Hyphantrophaga calva; phylum: Arthropoda; class: Insecta; order: Diptera; family: Tachinidae; genus: Hyphantrophaga; specificEpithet: calva; scientificNameAuthorship: Fleming & Wood, 2018; **Location:** continent: Central America; country: Costa Rica; countryCode: CR; stateProvince: Alajuela; county: Sector Rincon Rain Forest; locality: Area de Conservacion Guanacaste; verbatimLocality: San Lucas; verbatimElevation: 320; verbatimLatitude: 10.9185; verbatimLongitude: -85.3034; verbatimCoordinateSystem: Decimal; decimalLatitude: 10.9185; decimalLongitude: -85.3034; **Identification:** identifiedBy: AJ Fleming; dateIdentified: 2017; **Event:** samplingProtocol: Reared from the larvae of the Depressariidae, Anadasmus Janzen11; verbatimEventDate: 26-Aug-2012; **Record Level:** language: en; institutionCode: CNC; collectionCode: Insects; basisOfRecord: Pinned Specimen
- **Type status:** Paratype. **Occurrence:** occurrenceDetails: http://janzen.sas.upenn.edu; catalogNumber: DHJPAR0050501; recordedBy: D.H. Janzen, W. Hallwachs & Manuel Rios; individualID: DHJPAR0050501; individualCount: 1; sex: female; lifeStage: adult; preparations: pinned; otherCatalogNumbers: ACGBA3093-13, 12-SRNP-31968, BOLD:AAC5447; **Taxon:** scientificName: Hyphantrophaga calva; phylum: Arthropoda; class: Insecta; order: Diptera; family: Tachinidae; genus: Hyphantrophaga; specificEpithet: calva; scientificNameAuthorship: Fleming & Wood, 2018; **Location:** continent: Central America; country: Costa Rica; countryCode: CR; stateProvince: Guanacaste; county: Sector Pitilla; locality: Area de Conservacion Guanacaste; verbatimLocality: Casa Roberto; verbatimElevation: 520; verbatimLatitude: 11.011; verbatimLongitude: -85.4209; verbatimCoordinateSystem: Decimal; decimalLatitude: 11.011; decimalLongitude: -85.4209; **Identification:** identifiedBy: AJ Fleming; dateIdentified: 2017; **Event:** samplingProtocol: Reared from the larvae of the Depressariidae, Chlamydastis phytopteraEPR03; verbatimEventDate: 18-Jan-2013; **Record Level:** language: en; institutionCode: CNC; collectionCode: Insects; basisOfRecord: Pinned Specimen
- **Type status:** Paratype. **Occurrence:** occurrenceDetails: http://janzen.sas.upenn.edu; catalogNumber: DHJPAR0050544; recordedBy: D.H. Janzen, W. Hallwachs & Mercedes Moraga; individualID: DHJPAR0050544; individualCount: 1; sex: female; lifeStage: adult; preparations: pinned; otherCatalogNumbers: ACGBA3136-13, 12-SRNP-77287, BOLD:AAC5447; **Taxon:** scientificName: Hyphantrophaga calva; phylum: Arthropoda; class: Insecta; order: Diptera; family: Tachinidae; genus: Hyphantrophaga; specificEpithet: calva; scientificNameAuthorship: Fleming & Wood, 2018; **Location:** continent: Central America; country: Costa Rica; countryCode: CR; stateProvince: Alajuela; county: Sector Rincon Rain Forest; locality: Area de Conservacion Guanacaste; verbatimLocality: Finca Esmeralda; verbatimElevation: 123; verbatimLatitude: 10.9355; verbatimLongitude: -85.2531; verbatimCoordinateSystem: Decimal; decimalLatitude: 10.9355; decimalLongitude: -85.2531; **Identification:** identifiedBy: AJ Fleming; dateIdentified: 2017; **Event:** samplingProtocol: Reared from the larvae of the Depressariidae, Anadasmus Janzen11; verbatimEventDate: 16-Nov-2012; **Record Level:** language: en; institutionCode: CNC; collectionCode: Insects; basisOfRecord: Pinned Specimen
- **Type status:** Paratype. **Occurrence:** occurrenceDetails: http://janzen.sas.upenn.edu; catalogNumber: DHJPAR0050624; recordedBy: D.H. Janzen, W. Hallwachs & Jose Perez; individualID: DHJPAR0050624; individualCount: 1; sex: female; lifeStage: adult; preparations: pinned; otherCatalogNumbers: ACGBA3216-13, 13-SRNP-40038, BOLD:AAC5447; **Taxon:** scientificName: Hyphantrophaga calva; phylum: Arthropoda; class: Insecta; order: Diptera; family: Tachinidae; genus: Hyphantrophaga; specificEpithet: calva; scientificNameAuthorship: Fleming & Wood, 2018; **Location:** continent: Central America; country: Costa Rica; countryCode: CR; stateProvince: Alajuela; county: Sector Rincon Rain Forest; locality: Area de Conservacion Guanacaste; verbatimLocality: Quebrada Escondida; verbatimElevation: 420; verbatimLatitude: 10.8993; verbatimLongitude: -85.2749; verbatimCoordinateSystem: Decimal; decimalLatitude: 10.8993; decimalLongitude: -85.2749; **Identification:** identifiedBy: AJ Fleming; dateIdentified: 2017; **Event:** samplingProtocol: Reared from the larvae of the Depressariidae, Anadasmus Janzen11; verbatimEventDate: 06-Feb-2013; **Record Level:** language: en; institutionCode: CNC; collectionCode: Insects; basisOfRecord: Pinned Specimen
- **Type status:** Paratype. **Occurrence:** occurrenceDetails: http://janzen.sas.upenn.edu; catalogNumber: DHJPAR0050651; recordedBy: D.H. Janzen, W. Hallwachs & Cirilo Umaña; individualID: DHJPAR0050651; individualCount: 1; sex: female; lifeStage: adult; preparations: pinned; otherCatalogNumbers: ACGBA3243-13, 12-SRNP-77921, BOLD:AAC5447; **Taxon:** scientificName: Hyphantrophaga calva; phylum: Arthropoda; class: Insecta; order: Diptera; family: Tachinidae; genus: Hyphantrophaga; specificEpithet: calva; scientificNameAuthorship: Fleming & Wood, 2018; **Location:** continent: Central America; country: Costa Rica; countryCode: CR; stateProvince: Alajuela; county: Sector Rincon Rain Forest; locality: Area de Conservacion Guanacaste; verbatimLocality: Quebrada Bambu; verbatimElevation: 109; verbatimLatitude: 10.9301; verbatimLongitude: -85.2521; verbatimCoordinateSystem: Decimal; decimalLatitude: 10.9301; decimalLongitude: -85.2521; **Identification:** identifiedBy: AJ Fleming; dateIdentified: 2017; **Event:** samplingProtocol: Reared from the larvae of the Depressariidae, Anadasmus Janzen30; verbatimEventDate: 24-Dec-2012; **Record Level:** language: en; institutionCode: CNC; collectionCode: Insects; basisOfRecord: Pinned Specimen
- **Type status:** Paratype. **Occurrence:** occurrenceDetails: http://janzen.sas.upenn.edu; catalogNumber: DHJPAR0055031; recordedBy: D.H. Janzen, W. Hallwachs & Keiner Aragon; individualID: DHJPAR0055031; individualCount: 1; sex: male; lifeStage: adult; preparations: pinned; otherCatalogNumbers: ASHYH1578-14, 14-SRNP-45343, BOLD:AAC5447; **Taxon:** scientificName: Hyphantrophaga calva; phylum: Arthropoda; class: Insecta; order: Diptera; family: Tachinidae; genus: Hyphantrophaga; specificEpithet: calva; scientificNameAuthorship: Fleming & Wood, 2018; **Location:** continent: Central America; country: Costa Rica; countryCode: CR; stateProvince: Alajuela; county: Sector Rincon Rain Forest; locality: Area de Conservacion Guanacaste; verbatimLocality: Palomo; verbatimElevation: 96; verbatimLatitude: 10.9619; verbatimLongitude: -85.2804; verbatimCoordinateSystem: Decimal; decimalLatitude: 10.9619; decimalLongitude: -85.2804; **Identification:** identifiedBy: AJ Fleming; dateIdentified: 2017; **Event:** samplingProtocol: Reared from the larvae of the Depressariidae, Stenoma aterpes; **Record Level:** language: en; institutionCode: CNC; collectionCode: Insects; basisOfRecord: Pinned Specimen
- **Type status:** Paratype. **Occurrence:** occurrenceDetails: http://janzen.sas.upenn.edu; catalogNumber: DHJPAR0061376; recordedBy: D.H. Janzen, W. Hallwachs & Cirilo Umaña; individualID: DHJPAR0061376; individualCount: 1; sex: female; lifeStage: adult; preparations: pinned; otherCatalogNumbers: ACGBA7759-17, 17-SRNP-75888, BOLD:AAC5447; **Taxon:** scientificName: Hyphantrophaga calva; phylum: Arthropoda; class: Insecta; order: Diptera; family: Tachinidae; genus: Hyphantrophaga; specificEpithet: calva; scientificNameAuthorship: Fleming & Wood, 2018; **Location:** continent: Central America; country: Costa Rica; countryCode: CR; stateProvince: Guanacaste; county: Sector Rincon Rain Forest; locality: Area de Conservacion Guanacaste; verbatimLocality: Quebrada Bambu; verbatimElevation: 109; verbatimLatitude: 10.9301; verbatimLongitude: -85.2521; verbatimCoordinateSystem: Decimal; decimalLatitude: 10.9301; decimalLongitude: -85.2521; **Identification:** identifiedBy: AJ Fleming; dateIdentified: 2017; **Event:** samplingProtocol: Reared from the larvae of the Depressariidae, same as 05-SRNP-24825; verbatimEventDate: 28-Jul-2017; **Record Level:** language: en; institutionCode: CNC; collectionCode: Insects; basisOfRecord: Pinned Specimen

#### Description

**Male** (Fig. 7). Length: 7–11 mm. **Head** (Fig. 7b): vertex 2/9 of head width; two pairs of reclinate upper orbital setae; ocellar setae arising behind anterior ocellus; ocellar triangle gold; fronto-orbital plate shiny silver with a slight brassy-gold tinge on upper 3/4, setulose, setulae not extending below lowest frontal seta; parafacial silver and bare; eye with short sparse ommatrichia up to 2X as long as one ommatidium; facial ridge bare; pedicel brownish-black, concolorous with postpedicel; arista brown, very minutely pubescent, distinctly thickened on basal 1/8; palpus dark orange and haired apically, oar-shaped. **Thorax** (Fig. 7a, c): brassy tomentose dorsally, contrasting with dark silver grey laterally; densely covered in black setulae on all surfaces; four thin dorsal vittae, outermost two broken across suture, innermost pair unbroken, reaching 2nd postsutural dorsocentral seta; postpronotum with 3–4 setae arranged in a triangle; chaetotaxy: acrostichal setae 3:3; dorsocentral setae 3:3; intra-alar setae 2:3; supra-alar setae 2:3; two katepisternal setae; basal scutellar setae subequal in length to subapical scutellar setae; lateral scutellar setae less than 2/3 as long as subapical setae, curving inwards medially; apical scutellar setae short, crossed apically; one pair of discal scutellar setae more widely set than subapical setae; scutellum very slightly darkened across basal 20%, remainder concolorous with scutum. **Legs** (Fig. 7c): femora black in ground colour, remaining segments yellow in ground colour, covered in dark hairs; fore femur with dense silver tomentum on posterodorsal surface; hind coxa bare. **Wing** (Fig. 7a): pale translucent, hyaline, not distinctly infuscate; vein R_4+5_ with only 2–3 setulae at base. **Abdomen** (Fig. 7a, c): ground colour dorsally black, yellow-orange ventrally; mid-dorsal depression on ST1+2 almost reaching hind margin; median marginal setae present on ST1+2–T3; a complete row of marginal setae present on T4 and T5; discal setae only on present T5; sex patch covering ventral surfaces of T4–T5; distinct brassy-silver tomentose bands along anterior edge of tergites, covering 50% of T3 and almost 80% of T4; T5 brassy tomentose throughout. **Terminalia** (Fig. 7d, e, f): sternite 5 (Fig. 7f) with a deeply excavated median cleft, smoothly U-shaped, margins covered in dense tomentum. Lateral lobes of sternite rounded apically, 3–5 strong setae surrounded by many shorter, weaker setulae. Anterior plate of sternite 5 from subequal to slightly longer than apical lobes; unsclerotised "window" anterior to median cleft absent. Cerci in posterior view (Fig. 7d) rectangular and slightly shorter than surstyli, blunt and rounded at apex, completely separate medially but not divergent; in lateral view with a strong downward curve in apical 1/3; densely setulose along basal 2/3 dorsally, setulose ventrally along entire length (visible in lateral view). Surstylus in lateral view (Fig. 7e) almost triangular, ending in a slightly downcurved apex, making the structure appear blade-like; when viewed dorsally, surstyli appearing to point outward, not strongly convergent. Pregonite short, not well-developed, 1/3 as long as distiphallus, trumpet-like, at apex. Postgonite slightly narrow, 1/3 as wide as pregonite, sharply pointed and curved at apex. Distiphallus tubular with a slender median longitudinal sclerotised reinforcement on its posterior surface and a broad, anterolateral, sclerotised acrophallus on each side, joining the plate of opposite side on anterior surface near apex.

**Female**. Length: 8–11 mm. As males, differing by the presence of two pairs of proclinate orbital setae and the presence of setae on the hind coxa.

#### Diagnosis

*Hyphantrophaga calva* **sp. n.** can be distinguished from all other *Hyphantrophaga* species by the following combination of traits: thorax with three postsutural dorsocentral setae, two katepisternal setae, hind coxa bare in males, setose in females, median marginal setae present on ST1+2, discal setae only present on T5.

#### Etymology

From the Latin adjective “*calvus*”, meaning "bald", in reference to the lack of hairs on the hind coxa in the males of the species.

#### Distribution

Costa Rica, ACG, Alajuela and Guanacaste Provinces, 96–620 m elevation.

#### Ecology

*Hyphantrophaga calva* **sp. n.** has been reared 13 times from seven species of Lepidoptera in the family Depressariidae, *Andasmus* Janzen11, *Andasmus* Janzen25, *Andasmus* Janzen30, *Cerconota* Janzen102, *Cerconota* Janzen707, *Chlamydastis christhompsoni* (m.s. name), *Stenoma aterpes* Walsingham, 1913; in rain forest and dry-rain lowland intergrade.

### Hyphantrophaga ciriloumanai

Fleming & Wood sp. n.

urn:lsid:zoobank.org:act:10820514-749C-4741-82BF-3570BAD441DB

#### Materials

- **Type status:** Holotype. **Occurrence:** occurrenceDetails: http://janzen.sas.upenn.edu; catalogNumber: DHJPAR0021010; recordedBy: D.H. Janzen, W. Hallwachs & Jose Perez; individualID: DHJPAR0021010; individualCount: 1; sex: male; lifeStage: adult; preparations: pinned; otherCatalogNumbers: ASTA1353-07, 07-SRNP-42179, BOLD:AAA1908; **Taxon:** scientificName: Hyphantrophaga ciriloumanai; phylum: Arthropoda; class: Insecta; order: Diptera; family: Tachinidae; genus: Hyphantrophaga; specificEpithet: ciriloumanai; scientificNameAuthorship: Fleming & Wood, 2018; **Location:** continent: Central America; country: Costa Rica; countryCode: CR; stateProvince: Alajuela; county: Sector Rincon Rain Forest; locality: Area de Conservacion Guanacaste; verbatimLocality: Estacion Caribe; verbatimElevation: 415; verbatimLatitude: 10.9019; verbatimLongitude: -85.2749; verbatimCoordinateSystem: Decimal; decimalLatitude: 10.9019; decimalLongitude: -85.2749; **Identification:** identifiedBy: AJ Fleming; dateIdentified: 2017; **Event:** samplingProtocol: Reared from the larva of the Depressariidae, Anadasmus Janzen25; verbatimEventDate: 24-Aug-2007; **Record Level:** language: en; institutionCode: CNC; collectionCode: Insects; basisOfRecord: Pinned Specimen
- **Type status:** Paratype. **Occurrence:** occurrenceDetails: http://janzen.sas.upenn.edu; catalogNumber: DHJPAR0007288; recordedBy: D.H. Janzen, W. Hallwachs & Osvaldo Espinoza; individualID: DHJPAR0007288; individualCount: 1; sex: male; lifeStage: adult; preparations: pinned; otherCatalogNumbers: ASTAT060-06, 04-SRNP-60408, BOLD:AAA1908; **Taxon:** scientificName: Hyphantrophaga ciriloumanai; phylum: Arthropoda; class: Insecta; order: Diptera; family: Tachinidae; genus: Hyphantrophaga; specificEpithet: ciriloumanai; scientificNameAuthorship: Fleming & Wood, 2018; **Location:** continent: Central America; country: Costa Rica; countryCode: CR; stateProvince: Alajuela; county: Sector San Cristobal; locality: Area de Conservacion Guanacaste; verbatimLocality: Quebrada San Francisco; verbatimElevation: 690; verbatimLatitude: 10.8725; verbatimLongitude: -85.3793; verbatimCoordinateSystem: Decimal; decimalLatitude: 10.8725; decimalLongitude: -85.3793; **Identification:** identifiedBy: AJ Fleming; dateIdentified: 2017; **Event:** samplingProtocol: Reared from the larva of the Depressariidae, Anadasmus Janzen25; verbatimEventDate: 20-Nov-2004; **Record Level:** language: en; institutionCode: CNC; collectionCode: Insects; basisOfRecord: Pinned Specimen
- **Type status:** Paratype. **Occurrence:** occurrenceDetails: http://janzen.sas.upenn.edu; catalogNumber: DHJPAR0011465; recordedBy: D.H. Janzen, W. Hallwachs & Carolina Cano; individualID: DHJPAR0011465; individualCount: 1; sex: male; lifeStage: adult; preparations: pinned; otherCatalogNumbers: ASTAQ852-06, 01-SRNP-2659, BOLD:AAA1908; **Taxon:** scientificName: Hyphantrophaga ciriloumanai; phylum: Arthropoda; class: Insecta; order: Diptera; family: Tachinidae; genus: Hyphantrophaga; specificEpithet: ciriloumanai; scientificNameAuthorship: Fleming & Wood, 2018; **Location:** continent: Central America; country: Costa Rica; countryCode: CR; stateProvince: Alajuela; county: Sector San Cristobal; locality: Area de Conservacion Guanacaste; verbatimLocality: Quebrada Cementerio; verbatimElevation: 700; verbatimLatitude: 10.8712; verbatimLongitude: -85.3875; verbatimCoordinateSystem: Decimal; decimalLatitude: 10.8712; decimalLongitude: -85.3875; **Identification:** identifiedBy: AJ Fleming; dateIdentified: 2017; **Event:** samplingProtocol: Reared from the larva of the Depressariidae, Stenoma convexicostata; verbatimEventDate: 28-Aug-2001; **Record Level:** language: en; institutionCode: CNC; collectionCode: Insects; basisOfRecord: Pinned Specimen
- **Type status:** Paratype. **Occurrence:** occurrenceDetails: http://janzen.sas.upenn.edu; catalogNumber: DHJPAR0011466; recordedBy: D.H. Janzen, W. Hallwachs & Carolina Cano; individualID: DHJPAR0011466; individualCount: 1; sex: female; lifeStage: adult; preparations: pinned; otherCatalogNumbers: ASTAQ853-06, 01-SRNP-2561, BOLD:AAA1908; **Taxon:** scientificName: Hyphantrophaga ciriloumanai; phylum: Arthropoda; class: Insecta; order: Diptera; family: Tachinidae; genus: Hyphantrophaga; specificEpithet: ciriloumanai; scientificNameAuthorship: Fleming & Wood, 2018; **Location:** continent: Central America; country: Costa Rica; countryCode: CR; stateProvince: Alajuela; county: Sector San Cristobal; locality: Area de Conservacion Guanacaste; verbatimLocality: Quebrada Cementerio; verbatimElevation: 700; verbatimLatitude: 10.8712; verbatimLongitude: -85.3875; verbatimCoordinateSystem: Decimal; decimalLatitude: 10.8712; decimalLongitude: -85.3875; **Identification:** identifiedBy: AJ Fleming; dateIdentified: 2017; **Event:** samplingProtocol: Reared from the larva of the Depressariidae, Stenoma convexicostata; verbatimEventDate: 19-Aug-2001; **Record Level:** language: en; institutionCode: CNC; collectionCode: Insects; basisOfRecord: Pinned Specimen
- **Type status:** Paratype. **Occurrence:** occurrenceDetails: http://janzen.sas.upenn.edu; catalogNumber: DHJPAR0016239; recordedBy: D.H. Janzen, W. Hallwachs & Gloria Sihezar; individualID: DHJPAR0016239; individualCount: 1; sex: male; lifeStage: adult; preparations: pinned; otherCatalogNumbers: ASTAP268-06, 06-SRNP-6396, BOLD:AAA1908; **Taxon:** scientificName: Hyphantrophaga ciriloumanai; phylum: Arthropoda; class: Insecta; order: Diptera; family: Tachinidae; genus: Hyphantrophaga; specificEpithet: ciriloumanai; scientificNameAuthorship: Fleming & Wood, 2018; **Location:** continent: Central America; country: Costa Rica; countryCode: CR; stateProvince: Alajuela; county: Sector San Cristobal; locality: Area de Conservacion Guanacaste; verbatimLocality: Quebrada Cementerio; verbatimElevation: 700; verbatimLatitude: 10.8712; verbatimLongitude: -85.3875; verbatimCoordinateSystem: Decimal; decimalLatitude: 10.8712; decimalLongitude: -85.3875; **Identification:** identifiedBy: AJ Fleming; dateIdentified: 2017; **Event:** samplingProtocol: Reared from the larva of the Depressariidae, Anadasmus Janzen11; verbatimEventDate: 03-Sep-2006; **Record Level:** language: en; institutionCode: CNC; collectionCode: Insects; basisOfRecord: Pinned Specimen
- **Type status:** Paratype. **Occurrence:** occurrenceDetails: http://janzen.sas.upenn.edu; catalogNumber: DHJPAR0017158; recordedBy: D.H. Janzen, W. Hallwachs & Anabelle Cordoba; individualID: DHJPAR0017158; individualCount: 1; sex: female; lifeStage: adult; preparations: pinned; otherCatalogNumbers: ASTAP596-07, 07-SRNP-464, BOLD:AAA1908; **Taxon:** scientificName: Hyphantrophaga ciriloumanai; phylum: Arthropoda; class: Insecta; order: Diptera; family: Tachinidae; genus: Hyphantrophaga; specificEpithet: ciriloumanai; scientificNameAuthorship: Fleming & Wood, 2018; **Location:** continent: Central America; country: Costa Rica; countryCode: CR; stateProvince: Alajuela; county: Sector San Cristobal; locality: Area de Conservacion Guanacaste; verbatimLocality: Finca San Gabriel; verbatimElevation: 645; verbatimLatitude: 10.8777; verbatimLongitude: -85.3934; verbatimCoordinateSystem: Decimal; decimalLatitude: 10.8777; decimalLongitude: -85.3934; **Identification:** identifiedBy: AJ Fleming; dateIdentified: 2017; **Event:** samplingProtocol: Reared from the larva of the Depressariidae, Anadasmus Janzen25; verbatimEventDate: 07-Mar-2007; **Record Level:** language: en; institutionCode: CNC; collectionCode: Insects; basisOfRecord: Pinned Specimen
- **Type status:** Paratype. **Occurrence:** occurrenceDetails: http://janzen.sas.upenn.edu; catalogNumber: DHJPAR0019707; recordedBy: D.H. Janzen, W. Hallwachs & Gloria Sihezar; individualID: DHJPAR0019707; individualCount: 1; sex: female; lifeStage: adult; preparations: pinned; otherCatalogNumbers: ASTAB255-07, 07-SRNP-2003, BOLD:AAA1908; **Taxon:** scientificName: Hyphantrophaga ciriloumanai; phylum: Arthropoda; class: Insecta; order: Diptera; family: Tachinidae; genus: Hyphantrophaga; specificEpithet: ciriloumanai; scientificNameAuthorship: Fleming & Wood, 2018; **Location:** continent: Central America; country: Costa Rica; countryCode: CR; stateProvince: Alajuela; county: Sector San Cristobal; locality: Area de Conservacion Guanacaste; verbatimLocality: Estacion San Ramon; verbatimElevation: 660; verbatimLatitude: 10.8835; verbatimLongitude: -85.4097; verbatimCoordinateSystem: Decimal; decimalLatitude: 10.8835; decimalLongitude: -85.4097; **Identification:** identifiedBy: AJ Fleming; dateIdentified: 2017; **Event:** samplingProtocol: Reared from the larva of the Depressariidae, Anadasmus Janzen25; verbatimEventDate: 28-May-2007; **Record Level:** language: en; institutionCode: CNC; collectionCode: Insects; basisOfRecord: Pinned Specimen
- **Type status:** Paratype. **Occurrence:** occurrenceDetails: http://janzen.sas.upenn.edu; catalogNumber: DHJPAR0020971; recordedBy: D.H. Janzen, W. Hallwachs & Jose Perez; individualID: DHJPAR0020971; individualCount: 1; sex: female; lifeStage: adult; preparations: pinned; otherCatalogNumbers: ASTA1314-07, 07-SRNP-42176, BOLD:AAA1908; **Taxon:** scientificName: Hyphantrophaga ciriloumanai; phylum: Arthropoda; class: Insecta; order: Diptera; family: Tachinidae; genus: Hyphantrophaga; specificEpithet: ciriloumanai; scientificNameAuthorship: Fleming & Wood, 2018; **Location:** continent: Central America; country: Costa Rica; countryCode: CR; stateProvince: Alajuela; county: Sector Rincon Rain Forest; locality: Area de Conservacion Guanacaste; verbatimLocality: Estacion Caribe; verbatimElevation: 415; verbatimLatitude: 10.9019; verbatimLongitude: -85.2749; verbatimCoordinateSystem: Decimal; decimalLatitude: 10.9019; decimalLongitude: -85.2749; **Identification:** identifiedBy: AJ Fleming; dateIdentified: 2017; **Event:** samplingProtocol: Reared from the larva of the Depressariidae, Anadasmus Janzen25; verbatimEventDate: 27-Aug-2007; **Record Level:** language: en; institutionCode: CNC; collectionCode: Insects; basisOfRecord: Pinned Specimen
- **Type status:** Paratype. **Occurrence:** occurrenceDetails: http://janzen.sas.upenn.edu; catalogNumber: DHJPAR0007270; recordedBy: D.H. Janzen, W. Hallwachs & Petrona Rios; individualID: DHJPAR0007270; individualCount: 1; sex: male; lifeStage: adult; preparations: pinned; otherCatalogNumbers: ASTAT042-06, 04-SRNP-31074, BOLD:AAA1908; **Taxon:** scientificName: Hyphantrophaga ciriloumanai; phylum: Arthropoda; class: Insecta; order: Diptera; family: Tachinidae; genus: Hyphantrophaga; specificEpithet: ciriloumanai; scientificNameAuthorship: Fleming & Wood, 2018; **Location:** continent: Central America; country: Costa Rica; countryCode: CR; stateProvince: Guanacaste; county: Sector Pitilla; locality: Area de Conservacion Guanacaste; verbatimLocality: Estacion Pitilla; verbatimElevation: 675; verbatimLatitude: 10.9893; verbatimLongitude: -85.4258; verbatimCoordinateSystem: Decimal; decimalLatitude: 10.9893; decimalLongitude: -85.4258; **Identification:** identifiedBy: AJ Fleming; dateIdentified: 2017; **Event:** samplingProtocol: Reared from the larva of the Depressariidae, Anadasmus Janzen42; verbatimEventDate: 05-Apr-2004; **Record Level:** language: en; institutionCode: CNC; collectionCode: Insects; basisOfRecord: Pinned Specimen
- **Type status:** Paratype. **Occurrence:** occurrenceDetails: http://janzen.sas.upenn.edu; catalogNumber: DHJPAR0022956; recordedBy: D.H. Janzen, W. Hallwachs & Lucia Rios; individualID: DHJPAR0022956; individualCount: 1; sex: male; lifeStage: adult; preparations: pinned; otherCatalogNumbers: ASTAW120-08, 01-SRNP-24061, BOLD:AAA1908; **Taxon:** scientificName: Hyphantrophaga ciriloumanai; phylum: Arthropoda; class: Insecta; order: Diptera; family: Tachinidae; genus: Hyphantrophaga; specificEpithet: ciriloumanai; scientificNameAuthorship: Fleming & Wood, 2018; **Location:** continent: Central America; country: Costa Rica; countryCode: CR; stateProvince: Guanacaste; county: Sector El Hacha; locality: Area de Conservacion Guanacaste; verbatimLocality: Estacion Los Almendros; verbatimElevation: 290; verbatimLatitude: 11.0323; verbatimLongitude: -85.5278; verbatimCoordinateSystem: Decimal; decimalLatitude: 11.0323; decimalLongitude: -85.5278; **Identification:** identifiedBy: AJ Fleming; dateIdentified: 2017; **Event:** samplingProtocol: Reared from the larva of the Pyralidae, Accinctapubes albifasciataDHJ01; verbatimEventDate: 22-Dec-2001; **Record Level:** language: en; institutionCode: CNC; collectionCode: Insects; basisOfRecord: Pinned Specimen
- **Type status:** Paratype. **Occurrence:** occurrenceDetails: http://janzen.sas.upenn.edu; catalogNumber: DHJPAR0029604; recordedBy: D.H. Janzen, W. Hallwachs & Elda Araya; individualID: DHJPAR0029604; individualCount: 1; sex: female; lifeStage: adult; preparations: pinned; otherCatalogNumbers: ASHYM1025-09, 08-SRNP-4436, BOLD:AAA1908; **Taxon:** scientificName: Hyphantrophaga ciriloumanai; phylum: Arthropoda; class: Insecta; order: Diptera; family: Tachinidae; genus: Hyphantrophaga; specificEpithet: ciriloumanai; scientificNameAuthorship: Fleming & Wood, 2018; **Location:** continent: Central America; country: Costa Rica; countryCode: CR; stateProvince: Alajuela; county: Sector San Cristobal; locality: Area de Conservacion Guanacaste; verbatimLocality: Sendero Perdido; verbatimElevation: 620; verbatimLatitude: 10.8794; verbatimLongitude: -85.3861; verbatimCoordinateSystem: Decimal; decimalLatitude: 10.8794; decimalLongitude: -85.3861; **Identification:** identifiedBy: AJ Fleming; dateIdentified: 2017; **Event:** samplingProtocol: Reared from the larva of the Pyralidae, Deuterollyta oediperalisDHJ02; verbatimEventDate: 24-Aug-2008; **Record Level:** language: en; institutionCode: CNC; collectionCode: Insects; basisOfRecord: Pinned Specimen
- **Type status:** Paratype. **Occurrence:** occurrenceDetails: http://janzen.sas.upenn.edu; catalogNumber: DHJPAR0029607; recordedBy: D.H. Janzen, W. Hallwachs & Elda Araya; individualID: DHJPAR0029607; individualCount: 1; sex: male; lifeStage: adult; preparations: pinned; otherCatalogNumbers: ASHYM1028-09, 08-SRNP-4437, BOLD:AAA1908; **Taxon:** scientificName: Hyphantrophaga ciriloumanai; phylum: Arthropoda; class: Insecta; order: Diptera; family: Tachinidae; genus: Hyphantrophaga; specificEpithet: ciriloumanai; scientificNameAuthorship: Fleming & Wood, 2018; **Location:** continent: Central America; country: Costa Rica; countryCode: CR; stateProvince: Alajuela; county: Sector San Cristobal; locality: Area de Conservacion Guanacaste; verbatimLocality: Sendero Perdido; verbatimElevation: 620; verbatimLatitude: 10.8794; verbatimLongitude: -85.3861; verbatimCoordinateSystem: Decimal; decimalLatitude: 10.8794; decimalLongitude: -85.3861; **Identification:** identifiedBy: AJ Fleming; dateIdentified: 2017; **Event:** samplingProtocol: Reared from the larva of the Pyralidae, Deuterollyta oediperalisDHJ02; verbatimEventDate: 22-Aug-2008; **Record Level:** language: en; institutionCode: CNC; collectionCode: Insects; basisOfRecord: Pinned Specimen
- **Type status:** Paratype. **Occurrence:** occurrenceDetails: http://janzen.sas.upenn.edu; catalogNumber: DHJPAR0029619; recordedBy: D.H. Janzen, W. Hallwachs & Elda Araya; individualID: DHJPAR0029619; individualCount: 1; sex: female; lifeStage: adult; preparations: pinned; otherCatalogNumbers: ASHYM1040-09, 08-SRNP-4439, BOLD:AAA1908; **Taxon:** scientificName: Hyphantrophaga ciriloumanai; phylum: Arthropoda; class: Insecta; order: Diptera; family: Tachinidae; genus: Hyphantrophaga; specificEpithet: ciriloumanai; scientificNameAuthorship: Fleming & Wood, 2018; **Location:** continent: Central America; country: Costa Rica; countryCode: CR; stateProvince: Alajuela; county: Sector San Cristobal; locality: Area de Conservacion Guanacaste; verbatimLocality: Sendero Perdido; verbatimElevation: 620; verbatimLatitude: 10.8794; verbatimLongitude: -85.3861; verbatimCoordinateSystem: Decimal; decimalLatitude: 10.8794; decimalLongitude: -85.3861; **Identification:** identifiedBy: AJ Fleming; dateIdentified: 2017; **Event:** samplingProtocol: Reared from the larva of the Pyralidae, Deuterollyta oediperalisDHJ02; verbatimEventDate: 30-Aug-2008; **Record Level:** language: en; institutionCode: CNC; collectionCode: Insects; basisOfRecord: Pinned Specimen
- **Type status:** Paratype. **Occurrence:** occurrenceDetails: http://janzen.sas.upenn.edu; catalogNumber: DHJPAR0029992; recordedBy: D.H. Janzen, W. Hallwachs & Jose Perez; individualID: DHJPAR0029992; individualCount: 1; sex: male; lifeStage: adult; preparations: pinned; otherCatalogNumbers: ASHYB736-09, 08-SRNP-41992, BOLD:AAA1908; **Taxon:** scientificName: Hyphantrophaga ciriloumanai; phylum: Arthropoda; class: Insecta; order: Diptera; family: Tachinidae; genus: Hyphantrophaga; specificEpithet: ciriloumanai; scientificNameAuthorship: Fleming & Wood, 2018; **Location:** continent: Central America; country: Costa Rica; countryCode: CR; stateProvince: Alajuela; county: Sector Rincon Rain Forest; locality: Area de Conservacion Guanacaste; verbatimLocality: Rio Francia Arriba; verbatimElevation: 400; verbatimLatitude: 10.8967; verbatimLongitude: -85.29; verbatimCoordinateSystem: Decimal; decimalLatitude: 10.8967; decimalLongitude: -85.29; **Identification:** identifiedBy: AJ Fleming; dateIdentified: 2017; **Event:** samplingProtocol: Reared from the larva of the Depressariidae, Anadasmus Janzen25; verbatimEventDate: 10-Nov-2008; **Record Level:** language: en; institutionCode: CNC; collectionCode: Insects; basisOfRecord: Pinned Specimen
- **Type status:** Paratype. **Occurrence:** occurrenceDetails: http://janzen.sas.upenn.edu; catalogNumber: DHJPAR0030228; recordedBy: D.H. Janzen, W. Hallwachs & Duvalier Briceno; individualID: DHJPAR0030228; individualCount: 1; sex: male; lifeStage: adult; preparations: pinned; otherCatalogNumbers: ASHYB972-09, 09-SRNP-65048, BOLD:AAA1908; **Taxon:** scientificName: Hyphantrophaga ciriloumanai; phylum: Arthropoda; class: Insecta; order: Diptera; family: Tachinidae; genus: Hyphantrophaga; specificEpithet: ciriloumanai; scientificNameAuthorship: Fleming & Wood, 2018; **Location:** continent: Central America; country: Costa Rica; countryCode: CR; stateProvince: Alajuela; county: Brasilia; locality: Area de Conservacion Guanacaste; verbatimLocality: Moga; verbatimElevation: 320; verbatimLatitude: 11.0123; verbatimLongitude: -85.3493; verbatimCoordinateSystem: Decimal; decimalLatitude: 11.0123; decimalLongitude: -85.3493; **Identification:** identifiedBy: AJ Fleming; dateIdentified: 2017; **Event:** samplingProtocol: Reared from the larva of the Depressariidae, Anadasmus Janzen51; verbatimEventDate: 07-Feb-2009; **Record Level:** language: en; institutionCode: CNC; collectionCode: Insects; basisOfRecord: Pinned Specimen
- **Type status:** Paratype. **Occurrence:** occurrenceDetails: http://janzen.sas.upenn.edu; catalogNumber: DHJPAR0035708; recordedBy: D.H. Janzen, W. Hallwachs & Anabelle Cordoba; individualID: DHJPAR0035708; individualCount: 1; sex: female; lifeStage: adult; preparations: pinned; otherCatalogNumbers: ASHYD1089-09, 09-SRNP-41293, BOLD:AAA1908; **Taxon:** scientificName: Hyphantrophaga ciriloumanai; phylum: Arthropoda; class: Insecta; order: Diptera; family: Tachinidae; genus: Hyphantrophaga; specificEpithet: ciriloumanai; scientificNameAuthorship: Fleming & Wood, 2018; **Location:** continent: Central America; country: Costa Rica; countryCode: CR; stateProvince: Alajuela; county: Sector Rincon Rain Forest; locality: Area de Conservacion Guanacaste; verbatimLocality: San Lucas; verbatimElevation: 320; verbatimLatitude: 10.9185; verbatimLongitude: -85.3034; verbatimCoordinateSystem: Decimal; decimalLatitude: 10.9185; decimalLongitude: -85.3034; **Identification:** identifiedBy: AJ Fleming; dateIdentified: 2017; **Event:** samplingProtocol: Reared from the larva of the Depressariidae, Anadasmus Janzen28; verbatimEventDate: 14-Jul-2009; **Record Level:** language: en; institutionCode: CNC; collectionCode: Insects; basisOfRecord: Pinned Specimen
- **Type status:** Paratype. **Occurrence:** occurrenceDetails: http://janzen.sas.upenn.edu; catalogNumber: DHJPAR0035717; recordedBy: D.H. Janzen, W. Hallwachs & Osvaldo Espinoza; individualID: DHJPAR0035717; individualCount: 1; sex: female; lifeStage: adult; preparations: pinned; otherCatalogNumbers: ASHYD1098-09, 09-SRNP-3444, BOLD:AAA1908; **Taxon:** scientificName: Hyphantrophaga ciriloumanai; phylum: Arthropoda; class: Insecta; order: Diptera; family: Tachinidae; genus: Hyphantrophaga; specificEpithet: ciriloumanai; scientificNameAuthorship: Fleming & Wood, 2018; **Location:** continent: Central America; country: Costa Rica; countryCode: CR; stateProvince: Alajuela; county: Sector San Cristobal; locality: Area de Conservacion Guanacaste; verbatimLocality: Quebrada Cementerio; verbatimElevation: 700; verbatimLatitude: 10.8712; verbatimLongitude: -85.3875; verbatimCoordinateSystem: Decimal; decimalLatitude: 10.8712; decimalLongitude: -85.3875; **Identification:** identifiedBy: AJ Fleming; dateIdentified: 2017; **Event:** samplingProtocol: Reared from the larva of the Depressariidae, Anadasmus Janzen11; verbatimEventDate: 03-Aug-2009; **Record Level:** language: en; institutionCode: CNC; collectionCode: Insects; basisOfRecord: Pinned Specimen
- **Type status:** Paratype. **Occurrence:** occurrenceDetails: http://janzen.sas.upenn.edu; catalogNumber: DHJPAR0035721; recordedBy: D.H. Janzen, W. Hallwachs & Osvaldo Espinoza; individualID: DHJPAR0035721; individualCount: 1; sex: male; lifeStage: adult; preparations: pinned; otherCatalogNumbers: ASHYD1102-09, 09-SRNP-3436,; **Taxon:** scientificName: Hyphantrophaga ciriloumanai; phylum: Arthropoda; class: Insecta; order: Diptera; family: Tachinidae; genus: Hyphantrophaga; specificEpithet: ciriloumanai; scientificNameAuthorship: Fleming & Wood, 2018; **Location:** continent: Central America; country: Costa Rica; countryCode: CR; stateProvince: Alajuela; county: Sector San Cristobal; locality: Area de Conservacion Guanacaste; verbatimLocality: Quebrada Cementerio; verbatimElevation: 700; verbatimLatitude: 10.8712; verbatimLongitude: -85.3875; verbatimCoordinateSystem: Decimal; decimalLatitude: 10.8712; decimalLongitude: -85.3875; **Identification:** identifiedBy: AJ Fleming; dateIdentified: 2017; **Event:** samplingProtocol: Reared from the larva of the Depressariidae, Anadasmus Janzen11; verbatimEventDate: 07-Jul-2009; **Record Level:** language: en; institutionCode: CNC; collectionCode: Insects; basisOfRecord: Pinned Specimen
- **Type status:** Paratype. **Occurrence:** occurrenceDetails: http://janzen.sas.upenn.edu; catalogNumber: DHJPAR0035733; recordedBy: D.H. Janzen, W. Hallwachs & Osvaldo Espinoza; individualID: DHJPAR0035733; individualCount: 1; sex: male; lifeStage: adult; preparations: pinned; otherCatalogNumbers: ASHYD1114-09, 09-SRNP-3450, BOLD:AAA1908; **Taxon:** scientificName: Hyphantrophaga ciriloumanai; phylum: Arthropoda; class: Insecta; order: Diptera; family: Tachinidae; genus: Hyphantrophaga; specificEpithet: ciriloumanai; scientificNameAuthorship: Fleming & Wood, 2018; **Location:** continent: Central America; country: Costa Rica; countryCode: CR; stateProvince: Alajuela; county: Sector San Cristobal; locality: Area de Conservacion Guanacaste; verbatimLocality: Quebrada Cementerio; verbatimElevation: 700; verbatimLatitude: 10.8712; verbatimLongitude: -85.3875; verbatimCoordinateSystem: Decimal; decimalLatitude: 10.8712; decimalLongitude: -85.3875; **Identification:** identifiedBy: AJ Fleming; dateIdentified: 2017; **Event:** samplingProtocol: Reared from the larva of the Depressariidae, Anadasmus Janzen11; verbatimEventDate: 08-Aug-2009; **Record Level:** language: en; institutionCode: CNC; collectionCode: Insects; basisOfRecord: Pinned Specimen
- **Type status:** Paratype. **Occurrence:** occurrenceDetails: http://janzen.sas.upenn.edu; catalogNumber: DHJPAR0035734; recordedBy: D.H. Janzen, W. Hallwachs & Osvaldo Espinoza; individualID: DHJPAR0035734; individualCount: 1; sex: female; lifeStage: adult; preparations: pinned; otherCatalogNumbers: ASHYD1115-09, 09-SRNP-3845, BOLD:AAA1908; **Taxon:** scientificName: Hyphantrophaga ciriloumanai; phylum: Arthropoda; class: Insecta; order: Diptera; family: Tachinidae; genus: Hyphantrophaga; specificEpithet: ciriloumanai; scientificNameAuthorship: Fleming & Wood, 2018; **Location:** continent: Central America; country: Costa Rica; countryCode: CR; stateProvince: Alajuela; county: Sector San Cristobal; locality: Area de Conservacion Guanacaste; verbatimLocality: Finca San Gabriel; verbatimElevation: 645; verbatimLatitude: 10.8777; verbatimLongitude: -85.3934; verbatimCoordinateSystem: Decimal; decimalLatitude: 10.8777; decimalLongitude: -85.3934; **Identification:** identifiedBy: AJ Fleming; dateIdentified: 2017; **Event:** samplingProtocol: Reared from the larva of the Depressariidae, Anadasmus Janzen25; verbatimEventDate: 21-Aug-2009; **Record Level:** language: en; institutionCode: CNC; collectionCode: Insects; basisOfRecord: Pinned Specimen
- **Type status:** Paratype. **Occurrence:** occurrenceDetails: http://janzen.sas.upenn.edu; catalogNumber: DHJPAR0035846; recordedBy: D.H. Janzen, W. Hallwachs & Pablo Umaña Calderon; individualID: DHJPAR0035846; individualCount: 1; sex: female; lifeStage: adult; preparations: pinned; otherCatalogNumbers: ASHYD1227-09, 09-SRNP-41403, BOLD:AAA1908; **Taxon:** scientificName: Hyphantrophaga ciriloumanai; phylum: Arthropoda; class: Insecta; order: Diptera; family: Tachinidae; genus: Hyphantrophaga; specificEpithet: ciriloumanai; scientificNameAuthorship: Fleming & Wood, 2018; **Location:** continent: Central America; country: Costa Rica; countryCode: CR; stateProvince: Alajuela; county: Sector Rincon Rain Forest; locality: Area de Conservacion Guanacaste; verbatimLocality: Sendero Rincon; verbatimElevation: 430; verbatimLatitude: 10.8962; verbatimLongitude: -85.2777; verbatimCoordinateSystem: Decimal; decimalLatitude: 10.8962; decimalLongitude: -85.2777; **Identification:** identifiedBy: AJ Fleming; dateIdentified: 2017; **Event:** samplingProtocol: Reared from the larva of the Depressariidae, Stenoma Janzen06; verbatimEventDate: 02-Aug-2009; **Record Level:** language: en; institutionCode: CNC; collectionCode: Insects; basisOfRecord: Pinned Specimen
- **Type status:** Paratype. **Occurrence:** occurrenceDetails: http://janzen.sas.upenn.edu; catalogNumber: DHJPAR0035851; recordedBy: D.H. Janzen, W. Hallwachs & Noe Castillo; individualID: DHJPAR0035851; individualCount: 1; sex: female; lifeStage: adult; preparations: pinned; otherCatalogNumbers: ASHYD1232-09, 09-SRNP-69674, BOLD:AAA1908; **Taxon:** scientificName: Hyphantrophaga ciriloumanai; phylum: Arthropoda; class: Insecta; order: Diptera; family: Tachinidae; genus: Hyphantrophaga; specificEpithet: ciriloumanai; scientificNameAuthorship: Fleming & Wood, 2018; **Location:** continent: Central America; country: Costa Rica; countryCode: CR; stateProvince: Alajuela; county: Sector Rincon Rain Forest; locality: Area de Conservacion Guanacaste; verbatimLocality: Cafecito; verbatimElevation: 455; verbatimLatitude: 10.944; verbatimLongitude: -85.3174; verbatimCoordinateSystem: Decimal; decimalLatitude: 10.944; decimalLongitude: -85.3174; **Identification:** identifiedBy: AJ Fleming; dateIdentified: 2017; **Event:** samplingProtocol: Reared from the larva of the Depressariidae, Anadasmus Janzen16; verbatimEventDate: 29-Aug-2009; **Record Level:** language: en; institutionCode: CNC; collectionCode: Insects; basisOfRecord: Pinned Specimen
- **Type status:** Paratype. **Occurrence:** occurrenceDetails: http://janzen.sas.upenn.edu; catalogNumber: DHJPAR0036550; recordedBy: D.H. Janzen, W. Hallwachs & Osvaldo Espinoza; individualID: DHJPAR0036550; individualCount: 1; sex: male; lifeStage: adult; preparations: pinned; otherCatalogNumbers: ASHYE1461-09, 09-SRNP-4831, BOLD:AAA1908; **Taxon:** scientificName: Hyphantrophaga ciriloumanai; phylum: Arthropoda; class: Insecta; order: Diptera; family: Tachinidae; genus: Hyphantrophaga; specificEpithet: ciriloumanai; scientificNameAuthorship: Fleming & Wood, 2018; **Location:** continent: Central America; country: Costa Rica; countryCode: CR; stateProvince: Alajuela; county: Sector San Cristobal; locality: Area de Conservacion Guanacaste; verbatimLocality: Quebrada Cementerio; verbatimElevation: 700; verbatimLatitude: 10.8712; verbatimLongitude: -85.3875; verbatimCoordinateSystem: Decimal; decimalLatitude: 10.8712; decimalLongitude: -85.3875; **Identification:** identifiedBy: AJ Fleming; dateIdentified: 2017; **Event:** samplingProtocol: Reared from the larva of the Depressariidae, Anadasmus Janzen11; verbatimEventDate: 21-Oct-2009; **Record Level:** language: en; institutionCode: CNC; collectionCode: Insects; basisOfRecord: Pinned Specimen
- **Type status:** Paratype. **Occurrence:** occurrenceDetails: http://janzen.sas.upenn.edu; catalogNumber: DHJPAR0036562; recordedBy: D.H. Janzen, W. Hallwachs & Pablo Umaña Calderon; individualID: DHJPAR0036562; individualCount: 1; sex: male; lifeStage: adult; preparations: pinned; otherCatalogNumbers: ASHYE1473-09, 09-SRNP-42159, BOLD:AAA1908; **Taxon:** scientificName: Hyphantrophaga ciriloumanai; phylum: Arthropoda; class: Insecta; order: Diptera; family: Tachinidae; genus: Hyphantrophaga; specificEpithet: ciriloumanai; scientificNameAuthorship: Fleming & Wood, 2018; **Location:** continent: Central America; country: Costa Rica; countryCode: CR; stateProvince: Alajuela; county: Sector Rincon Rain Forest; locality: Area de Conservacion Guanacaste; verbatimLocality: San Lucas; verbatimElevation: 320; verbatimLatitude: 10.9185; verbatimLongitude: -85.3034; verbatimCoordinateSystem: Decimal; decimalLatitude: 10.9185; decimalLongitude: -85.3034; **Identification:** identifiedBy: AJ Fleming; dateIdentified: 2017; **Event:** samplingProtocol: Reared from the larva of the Depressariidae, Anadasmus Janzen25; verbatimEventDate: 12-Sep-2009; **Record Level:** language: en; institutionCode: CNC; collectionCode: Insects; basisOfRecord: Pinned Specimen
- **Type status:** Paratype. **Occurrence:** occurrenceDetails: http://janzen.sas.upenn.edu; catalogNumber: DHJPAR0036577; recordedBy: D.H. Janzen, W. Hallwachs & Osvaldo Espinoza; individualID: DHJPAR0036577; individualCount: 1; sex: female; lifeStage: adult; preparations: pinned; otherCatalogNumbers: ASHYE1488-09, 09-SRNP-3858, BOLD:AAA1908; **Taxon:** scientificName: Hyphantrophaga ciriloumanai; phylum: Arthropoda; class: Insecta; order: Diptera; family: Tachinidae; genus: Hyphantrophaga; specificEpithet: ciriloumanai; scientificNameAuthorship: Fleming & Wood, 2018; **Location:** continent: Central America; country: Costa Rica; countryCode: CR; stateProvince: Alajuela; county: Sector San Cristobal; locality: Area de Conservacion Guanacaste; verbatimLocality: Finca San Gabriel; verbatimElevation: 645; verbatimLatitude: 10.8777; verbatimLongitude: -85.3934; verbatimCoordinateSystem: Decimal; decimalLatitude: 10.8777; decimalLongitude: -85.3934; **Identification:** identifiedBy: AJ Fleming; dateIdentified: 2017; **Event:** samplingProtocol: Reared from the larva of the Depressariidae, Anadasmus Janzen25; verbatimEventDate: 27-Aug-2009; **Record Level:** language: en; institutionCode: CNC; collectionCode: Insects; basisOfRecord: Pinned Specimen
- **Type status:** Paratype. **Occurrence:** occurrenceDetails: http://janzen.sas.upenn.edu; catalogNumber: DHJPAR0037338; recordedBy: D.H. Janzen, W. Hallwachs & Elda Araya; individualID: DHJPAR0037338; individualCount: 1; sex: female; lifeStage: adult; preparations: pinned; otherCatalogNumbers: ASHYC4083-10, 09-SRNP-5595, BOLD:AAA1908; **Taxon:** scientificName: Hyphantrophaga ciriloumanai; phylum: Arthropoda; class: Insecta; order: Diptera; family: Tachinidae; genus: Hyphantrophaga; specificEpithet: ciriloumanai; scientificNameAuthorship: Fleming & Wood, 2018; **Location:** continent: Central America; country: Costa Rica; countryCode: CR; stateProvince: Alajuela; county: Sector San Cristobal; locality: Area de Conservacion Guanacaste; verbatimLocality: Finca San Gabriel; verbatimElevation: 645; verbatimLatitude: 10.8777; verbatimLongitude: -85.3934; verbatimCoordinateSystem: Decimal; decimalLatitude: 10.8777; decimalLongitude: -85.3934; **Identification:** identifiedBy: AJ Fleming; dateIdentified: 2017; **Event:** samplingProtocol: Reared from the larva of the Depressariidae, Anadasmus Janzen25; verbatimEventDate: 01-Dec-2009; **Record Level:** language: en; institutionCode: CNC; collectionCode: Insects; basisOfRecord: Pinned Specimen
- **Type status:** Paratype. **Occurrence:** occurrenceDetails: http://janzen.sas.upenn.edu; catalogNumber: DHJPAR0037481; recordedBy: D.H. Janzen, W. Hallwachs & Elda Araya; individualID: DHJPAR0037481; individualCount: 1; sex: male; lifeStage: adult; preparations: pinned; otherCatalogNumbers: ASHYC4226-10, 09-SRNP-7106, BOLD:AAA1908; **Taxon:** scientificName: Hyphantrophaga ciriloumanai; phylum: Arthropoda; class: Insecta; order: Diptera; family: Tachinidae; genus: Hyphantrophaga; specificEpithet: ciriloumanai; scientificNameAuthorship: Fleming & Wood, 2018; **Location:** continent: Central America; country: Costa Rica; countryCode: CR; stateProvince: Alajuela; county: Sector San Cristobal; locality: Area de Conservacion Guanacaste; verbatimLocality: Puente Palma; verbatimElevation: 460; verbatimLatitude: 10.9163; verbatimLongitude: -85.3787; verbatimCoordinateSystem: Decimal; decimalLatitude: 10.9163; decimalLongitude: -85.3787; **Identification:** identifiedBy: AJ Fleming; dateIdentified: 2017; **Event:** samplingProtocol: Reared from the larva of the Depressariidae, Anadasmus Janzen51; verbatimEventDate: 31-Jan-2010; **Record Level:** language: en; institutionCode: CNC; collectionCode: Insects; basisOfRecord: Pinned Specimen
- **Type status:** Paratype. **Occurrence:** occurrenceDetails: http://janzen.sas.upenn.edu; catalogNumber: DHJPAR0037563; recordedBy: D.H. Janzen, W. Hallwachs & Maricruz Castillo; individualID: DHJPAR0037563; individualCount: 1; sex: female; lifeStage: adult; preparations: pinned; otherCatalogNumbers: ASHYC4308-10, 09-SRNP-80100, BOLD:AAA1908; **Taxon:** scientificName: Hyphantrophaga ciriloumanai; phylum: Arthropoda; class: Insecta; order: Diptera; family: Tachinidae; genus: Hyphantrophaga; specificEpithet: ciriloumanai; scientificNameAuthorship: Fleming & Wood, 2018; **Location:** continent: Central America; country: Costa Rica; countryCode: CR; stateProvince: Alajuela; county: Sector Rincon Rain Forest; locality: Area de Conservacion Guanacaste; verbatimLocality: Jacobo; verbatimElevation: 461; verbatimLatitude: 10.9408; verbatimLongitude: -85.3177; verbatimCoordinateSystem: Decimal; decimalLatitude: 10.9408; decimalLongitude: -85.3177; **Identification:** identifiedBy: AJ Fleming; dateIdentified: 2017; **Event:** samplingProtocol: Reared from the larva of the Depressariidae, Anadasmus Janzen16; verbatimEventDate: 31-Oct-2009; **Record Level:** language: en; institutionCode: CNC; collectionCode: Insects; basisOfRecord: Pinned Specimen
- **Type status:** Paratype. **Occurrence:** occurrenceDetails: http://janzen.sas.upenn.edu; catalogNumber: DHJPAR0040175; recordedBy: D.H. Janzen, W. Hallwachs & Calixto Moraga; individualID: DHJPAR0040175; individualCount: 1; sex: male; lifeStage: adult; preparations: pinned; otherCatalogNumbers: ASHYE2342-11, 09-SRNP-32287, BOLD:AAA1908; **Taxon:** scientificName: Hyphantrophaga ciriloumanai; phylum: Arthropoda; class: Insecta; order: Diptera; family: Tachinidae; genus: Hyphantrophaga; specificEpithet: ciriloumanai; scientificNameAuthorship: Fleming & Wood, 2018; **Location:** continent: Central America; country: Costa Rica; countryCode: CR; stateProvince: Guanacaste; county: Sector Pitilla; locality: Area de Conservacion Guanacaste; verbatimLocality: Sendero Laguna; verbatimElevation: 680; verbatimLatitude: 10.9888; verbatimLongitude: -85.4234; verbatimCoordinateSystem: Decimal; decimalLatitude: 10.9888; decimalLongitude: -85.4234; **Identification:** identifiedBy: AJ Fleming; dateIdentified: 2017; **Event:** samplingProtocol: Reared from the larva of the Depressariidae, Anadasmus Janzen41; verbatimEventDate: 21-Aug-2009; **Record Level:** language: en; institutionCode: CNC; collectionCode: Insects; basisOfRecord: Pinned Specimen
- **Type status:** Other material. **Occurrence:** occurrenceDetails: http://janzen.sas.upenn.edu; catalogNumber: DHJPAR0040927; recordedBy: D.H. Janzen, W. Hallwachs & Gloria Sihezar; individualID: DHJPAR0040927; individualCount: 1; lifeStage: adult; preparations: pinned; otherCatalogNumbers: ASHYF842-11, 10-SRNP-6211, BOLD:AAA1908; **Taxon:** scientificName: Hyphantrophaga ciriloumanai; phylum: Arthropoda; class: Insecta; order: Diptera; family: Tachinidae; genus: Hyphantrophaga; specificEpithet: ciriloumanai; scientificNameAuthorship: Fleming & Wood, 2018; **Location:** continent: Central America; country: Costa Rica; countryCode: CR; stateProvince: Alajuela; county: Sector San Cristobal; locality: Area de Conservacion Guanacaste; verbatimLocality: Tajo Angeles; verbatimElevation: 540; verbatimLatitude: 10.8647; verbatimLongitude: -85.4153; verbatimCoordinateSystem: Decimal; decimalLatitude: 10.8647; decimalLongitude: -85.4153; **Identification:** identifiedBy: AJ Fleming; dateIdentified: 2017; **Event:** samplingProtocol: Reared from the larva of the Depressariidae, Anadasmus Janzen42; verbatimEventDate: 22-Nov-2010; **Record Level:** language: en; institutionCode: CNC; collectionCode: Insects; basisOfRecord: Pinned Specimen
- **Type status:** Other material. **Occurrence:** occurrenceDetails: http://janzen.sas.upenn.edu; catalogNumber: DHJPAR0040928; recordedBy: D.H. Janzen, W. Hallwachs & Gloria Sihezar; individualID: DHJPAR0040928; individualCount: 1; lifeStage: adult; preparations: pinned; otherCatalogNumbers: ASHYF843-11, 10-SRNP-6212, BOLD:AAA1908; **Taxon:** scientificName: Hyphantrophaga ciriloumanai; phylum: Arthropoda; class: Insecta; order: Diptera; family: Tachinidae; genus: Hyphantrophaga; specificEpithet: ciriloumanai; scientificNameAuthorship: Fleming & Wood, 2018; **Location:** continent: Central America; country: Costa Rica; countryCode: CR; stateProvince: Alajuela; county: Sector San Cristobal; locality: Area de Conservacion Guanacaste; verbatimLocality: Tajo Angeles; verbatimElevation: 540; verbatimLatitude: 10.8647; verbatimLongitude: -85.4153; verbatimCoordinateSystem: Decimal; decimalLatitude: 10.8647; decimalLongitude: -85.4153; **Identification:** identifiedBy: AJ Fleming; dateIdentified: 2017; **Event:** samplingProtocol: Reared from the larva of the Depressariidae, Anadasmus Janzen42; verbatimEventDate: 20-Nov-2010; **Record Level:** language: en; institutionCode: CNC; collectionCode: Insects; basisOfRecord: Pinned Specimen
- **Type status:** Other material. **Occurrence:** occurrenceDetails: http://janzen.sas.upenn.edu; catalogNumber: DHJPAR0040935; recordedBy: D.H. Janzen, W. Hallwachs & Gloria Sihezar; individualID: DHJPAR0040935; individualCount: 1; lifeStage: adult; preparations: pinned; otherCatalogNumbers: ASHYF850-11, 10-SRNP-6221, BOLD:AAA1908; **Taxon:** scientificName: Hyphantrophaga ciriloumanai; phylum: Arthropoda; class: Insecta; order: Diptera; family: Tachinidae; genus: Hyphantrophaga; specificEpithet: ciriloumanai; scientificNameAuthorship: Fleming & Wood, 2018; **Location:** continent: Central America; country: Costa Rica; countryCode: CR; stateProvince: Alajuela; county: Sector San Cristobal; locality: Area de Conservacion Guanacaste; verbatimLocality: Tajo Angeles; verbatimElevation: 540; verbatimLatitude: 10.8647; verbatimLongitude: -85.4153; verbatimCoordinateSystem: Decimal; decimalLatitude: 10.8647; decimalLongitude: -85.4153; **Identification:** identifiedBy: AJ Fleming; dateIdentified: 2017; **Event:** samplingProtocol: Reared from the larva of the Depressariidae, Anadasmus Janzen25; verbatimEventDate: 02-Dec-2010; **Record Level:** language: en; institutionCode: CNC; collectionCode: Insects; basisOfRecord: Pinned Specimen
- **Type status:** Other material. **Occurrence:** occurrenceDetails: http://janzen.sas.upenn.edu; catalogNumber: DHJPAR0042651; recordedBy: D.H. Janzen, W. Hallwachs & Osvaldo Espinoza; individualID: DHJPAR0042651; individualCount: 1; lifeStage: adult; preparations: pinned; otherCatalogNumbers: ASHYH409-11, 11-SRNP-70, BOLD:AAA1908; **Taxon:** scientificName: Hyphantrophaga ciriloumanai; phylum: Arthropoda; class: Insecta; order: Diptera; family: Tachinidae; genus: Hyphantrophaga; specificEpithet: ciriloumanai; scientificNameAuthorship: Fleming & Wood, 2018; **Location:** continent: Central America; country: Costa Rica; countryCode: CR; stateProvince: Alajuela; county: Sector San Cristobal; locality: Area de Conservacion Guanacaste; verbatimLocality: Tajo Angeles; verbatimElevation: 540; verbatimLatitude: 10.8647; verbatimLongitude: -85.4153; verbatimCoordinateSystem: Decimal; decimalLatitude: 10.8647; decimalLongitude: -85.4153; **Identification:** identifiedBy: AJ Fleming; dateIdentified: 2017; **Event:** samplingProtocol: Reared from the larva of the Depressariidae, Anadasmus Janzen17; verbatimEventDate: 20-Feb-2011; **Record Level:** language: en; institutionCode: CNC; collectionCode: Insects; basisOfRecord: Pinned Specimen
- **Type status:** Other material. **Occurrence:** occurrenceDetails: http://janzen.sas.upenn.edu; catalogNumber: DHJPAR0046574; recordedBy: D.H. Janzen, W. Hallwachs & Jose Cortez; individualID: DHJPAR0046574; individualCount: 1; sex: female; lifeStage: adult; preparations: pinned; otherCatalogNumbers: ACGBA747-12, 11-SRNP-57582, BOLD:AAA1908; **Taxon:** scientificName: Hyphantrophaga ciriloumanai; phylum: Arthropoda; class: Insecta; order: Diptera; family: Tachinidae; genus: Hyphantrophaga; specificEpithet: ciriloumanai; scientificNameAuthorship: Fleming & Wood, 2018; **Location:** continent: Central America; country: Costa Rica; countryCode: CR; stateProvince: Guanacaste; county: Sector Mundo Nuevo; locality: Area de Conservacion Guanacaste; verbatimLocality: Cerro Gongora Pelado; verbatimElevation: 740; verbatimLatitude: 10.7631; verbatimLongitude: -85.4133; verbatimCoordinateSystem: Decimal; decimalLatitude: 10.7631; decimalLongitude: -85.4133; **Identification:** identifiedBy: AJ Fleming; dateIdentified: 2017; **Event:** samplingProtocol: Reared from the larva of the Depressariidae, Anadasmus Janzen26; verbatimEventDate: 28-Jan-2012; **Record Level:** language: en; institutionCode: CNC; collectionCode: Insects; basisOfRecord: Pinned Specimen
- **Type status:** Other material. **Occurrence:** occurrenceDetails: http://janzen.sas.upenn.edu; catalogNumber: DHJPAR0050694; recordedBy: D.H. Janzen, W. Hallwachs & Elda Araya; individualID: DHJPAR0050694; individualCount: 1; sex: female; lifeStage: adult; preparations: pinned; otherCatalogNumbers: ACGBA3286-13, 12-SRNP-4461, BOLD:AAA1908; **Taxon:** scientificName: Hyphantrophaga ciriloumanai; phylum: Arthropoda; class: Insecta; order: Diptera; family: Tachinidae; genus: Hyphantrophaga; specificEpithet: ciriloumanai; scientificNameAuthorship: Fleming & Wood, 2018; **Location:** continent: Central America; country: Costa Rica; countryCode: CR; stateProvince: Alajuela; county: Sector San Cristobal; locality: Area de Conservacion Guanacaste; verbatimLocality: Sendero Vivero; verbatimElevation: 730; verbatimLatitude: 10.8674; verbatimLongitude: -85.3874; verbatimCoordinateSystem: Decimal; decimalLatitude: 10.8674; decimalLongitude: -85.3874; **Identification:** identifiedBy: AJ Fleming; dateIdentified: 2017; **Event:** samplingProtocol: Reared from the larva of the Depressariidae, Anadasmus Janzen28; verbatimEventDate: 17-Nov-2012; **Record Level:** language: en; institutionCode: CNC; collectionCode: Insects; basisOfRecord: Pinned Specimen
- **Type status:** Other material. **Occurrence:** occurrenceDetails: http://janzen.sas.upenn.edu; catalogNumber: DHJPAR0050697; recordedBy: D.H. Janzen, W. Hallwachs & Elda Araya; individualID: DHJPAR0050697; individualCount: 1; sex: female; lifeStage: adult; preparations: pinned; otherCatalogNumbers: ACGBA3289-13, 12-SRNP-4462, BOLD:AAA1908; **Taxon:** scientificName: Hyphantrophaga ciriloumanai; phylum: Arthropoda; class: Insecta; order: Diptera; family: Tachinidae; genus: Hyphantrophaga; specificEpithet: ciriloumanai; scientificNameAuthorship: Fleming & Wood, 2018; **Location:** continent: Central America; country: Costa Rica; countryCode: CR; stateProvince: Alajuela; county: Sector San Cristobal; locality: Area de Conservacion Guanacaste; verbatimLocality: Sendero Vivero; verbatimElevation: 730; verbatimLatitude: 10.8674; verbatimLongitude: -85.3874; verbatimCoordinateSystem: Decimal; decimalLatitude: 10.8674; decimalLongitude: -85.3874; **Identification:** identifiedBy: AJ Fleming; dateIdentified: 2017; **Event:** samplingProtocol: Reared from the larva of the Depressariidae, Anadasmus Janzen28; verbatimEventDate: 21-Nov-2012; **Record Level:** language: en; institutionCode: CNC; collectionCode: Insects; basisOfRecord: Pinned Specimen
- **Type status:** Other material. **Occurrence:** occurrenceDetails: http://janzen.sas.upenn.edu; catalogNumber: DHJPAR0061299; recordedBy: D.H. Janzen, W. Hallwachs & Anabelle Cordoba; individualID: DHJPAR0061299; individualCount: 1; lifeStage: adult; preparations: pinned; otherCatalogNumbers: ACGBA7682-17, 17-SRNP-27005, BOLD:AAA1908; **Taxon:** scientificName: Hyphantrophaga ciriloumanai; phylum: Arthropoda; class: Insecta; order: Diptera; family: Tachinidae; genus: Hyphantrophaga; specificEpithet: ciriloumanai; scientificNameAuthorship: Fleming & Wood, 2018; **Location:** continent: Central America; country: Costa Rica; countryCode: CR; stateProvince: Guanacaste; county: Sector Rincon Rain Forest; locality: Area de Conservacion Guanacaste; verbatimLocality: Camino Rio Nino; verbatimElevation: 326; verbatimLatitude: 10.9707; verbatimLongitude: -85.3143; verbatimCoordinateSystem: Decimal; decimalLatitude: 10.9707; decimalLongitude: -85.3143; **Identification:** identifiedBy: AJ Fleming; dateIdentified: 2017; **Event:** samplingProtocol: Reared from the larva of the Depressariidae, Anadasmus Janzen17; verbatimEventDate: 25-Jul-2017; **Record Level:** language: en; institutionCode: CNC; collectionCode: Insects; basisOfRecord: Pinned Specimen
- **Type status:** Other material. **Occurrence:** occurrenceDetails: http://janzen.sas.upenn.edu; catalogNumber: DHJPAR0061302; recordedBy: D.H. Janzen, W. Hallwachs & Anabelle Cordoba; individualID: DHJPAR0061302; individualCount: 1; lifeStage: adult; preparations: pinned; otherCatalogNumbers: ACGBA7685-17, 17-SRNP-27002, BOLD:AAA1908; **Taxon:** scientificName: Hyphantrophaga ciriloumanai; phylum: Arthropoda; class: Insecta; order: Diptera; family: Tachinidae; genus: Hyphantrophaga; specificEpithet: ciriloumanai; scientificNameAuthorship: Fleming & Wood, 2018; **Location:** continent: Central America; country: Costa Rica; countryCode: CR; stateProvince: Guanacaste; county: Sector Rincon Rain Forest; locality: Area de Conservacion Guanacaste; verbatimLocality: Camino Rio Nino; verbatimElevation: 326; verbatimLatitude: 10.9707; verbatimLongitude: -85.3143; verbatimCoordinateSystem: Decimal; decimalLatitude: 10.9707; decimalLongitude: -85.3143; **Identification:** identifiedBy: AJ Fleming; dateIdentified: 2017; **Event:** samplingProtocol: Reared from the larva of the Depressariidae, Anadasmus Janzen17; verbatimEventDate: 30-Jul-2017; **Record Level:** language: en; institutionCode: CNC; collectionCode: Insects; basisOfRecord: Pinned Specimen
- **Type status:** Other material. **Occurrence:** occurrenceDetails: http://janzen.sas.upenn.edu; catalogNumber: DHJPAR0061395; recordedBy: D.H. Janzen, W. Hallwachs & Cirilo Umaña; individualID: DHJPAR0061395; individualCount: 1; lifeStage: adult; preparations: pinned; otherCatalogNumbers: ACGBA7778-17, 17-SRNP-75609, BOLD:AAA1908; **Taxon:** scientificName: Hyphantrophaga ciriloumanai; phylum: Arthropoda; class: Insecta; order: Diptera; family: Tachinidae; genus: Hyphantrophaga; specificEpithet: ciriloumanai; scientificNameAuthorship: Fleming & Wood, 2018; **Location:** continent: Central America; country: Costa Rica; countryCode: CR; stateProvince: Guanacaste; county: Sector Rincon Rain Forest; locality: Area de Conservacion Guanacaste; verbatimLocality: Quebrada Bambu; verbatimElevation: 109; verbatimLatitude: 10.9301; verbatimLongitude: -85.2521; verbatimCoordinateSystem: Decimal; decimalLatitude: 10.9301; decimalLongitude: -85.2521; **Identification:** identifiedBy: AJ Fleming; dateIdentified: 2017; **Event:** samplingProtocol: Reared from the larva of the Depressariidae, Anadasmus Janzen25; verbatimEventDate: 22-Jun-2017; **Record Level:** language: en; institutionCode: CNC; collectionCode: Insects; basisOfRecord: Pinned Specimen

#### Description

**Male** (Fig. 8). Length: 7–8 mm. **Head** (Fig. 8b): vertex 1/4 of head width; two pairs of reclinate upper orbital setae; ocellar setae arising behind anterior ocellus; ocellar triangle gold; fronto-orbital plate shiny silver with brilliant gold at vertex and in a narrow strip along frontal setae, sparsely setulose, setulae not extending below lowest frontal seta; parafacial silver and bare; eye densely haired; facial ridge bare; pedicel black, concolorous with postpedicel; arista black, very minutely pubescent, distinctly thickened on basal 1/3–1/4; palpus yellow and haired apically, oar-shaped. **Thorax** (Fig. 8a, c): brilliant gold tomentose dorsally, contrasting with silver grey laterally; dorsally with dense dark setulae interspersed amongst setae; four thin dorsal vittae, outermost two broken across suture, innermost pair narrowly broken across suture, reaching 2nd postsutural dorsocentral seta; postpronotum with 3–5 setae arranged in a triangle; chaetotaxy: acrostichal setae 3:3; dorsocentral setae 3:4; intra-alar setae 2:3; supra-alar setae 2:3; two katepisternal setae; basal scutellar setae subequal to subapical setae with a strong inward curve; lateral scutellar setae 2/3 as long as subapical setae, slightly curving inwards medially; apical scutellar setae 1/3 as long as subapical setae, crossed apically; one pair of discal scutellar setae set as widely apart as subapical setae; scutellum concolorous with scutum. **Legs** (Fig. 8c): black ground colour; fore femur with dense silver tomentum on posterodorsal surface; hind coxa setose. **Wing** (Fig. 8a): pale translucent, hyaline, only slightly infuscate near base; vein R_4+5_ with 2–3 setulae at base. **Abdomen** (Fig. 8a, c): ground colour black; middorsal depression on ST1+2 reaching hind margin; median marginal setae present on ST1+2–T3; a complete row of marginal setae present on T4; discal setae present on T3–T5; sex patch absent; distinct gold tomentose bands along anterior edge of T3 and T4, unbroken medially and covering almost 80% of tergites; T5 with gold tomentum throughout. **Terminalia** (Fig. 8d, e, f): sternite 5 (Fig. 8f) with a deeply excavated median cleft, narrow U-shaped, margins covered in dense tomentum. Lateral lobes of sternite rounded apically, 3–5 strong setae surrounded by many shorter, weaker setulae. Anterior plate of sternite 5 ranging from subequal to 1/2 as long as apical lobes; unsclerotised "window" anterior to median cleft absent. Cerci in posterior view (Fig. 8d) rectangular and slightly shorter than surstyli, very slightly clubbed and rounded at apex, completely separate medially but not divergent; in lateral view with a strong downward curve in apical 1/3; densely setulose along basal 2/3 dorsally, setulose ventrally along entire length (visible in lateral view). Surstylus in lateral view (Fig. 8e) slender, almost evenly parallel sided, ending in a slightly downcurved apex, making the structure appear blade-like; when viewed dorsally, surstyli appearing to point outward, not strongly convergent. Pregonite short, not well-developed, 1/3 as long as distiphallus, trumpet-like, at apex. Postgonite slightly narrow, 1/3 as wide as pregonite, sharply pointed and curved at apex. Distiphallus sail-shaped, with a slender median longitudinal sclerotised reinforcement on its posterior surface and a broad, anterolateral, sclerotised acrophallus on each side, joining the plate of opposite side on anterior surface near apex.

**Female**. Length: 7 mm. As male, differing only by the presence of two pairs of proclinate orbital setae.

#### Diagnosis

*Hyphantrophaga ciriloumanai* **sp. n.** can be distinguished from all other *Hyphantrophaga* species by the following combination of traits: fronto-orbital plate gold tomentose, pedicel and arista black, thorax gold dorsally, silver/grey tomentose laterally, four thoracic vittae, inner pair broken at suture, four postsutural dorsocentral setae, legs black, hind coxa setose, abdominal tomentum gold, covering more than 50% of tergal surface, median marginal setae present on ST1+2, discal setae present T3–T5.

#### Etymology

*Hyphantrophaga ciriloumanai* **sp. n.** is named in recognition of Cirilo Umaña Dominguez's dedication and work in finding and rearing the ACG caterpillars that contained tachinid larvae.

#### Distribution

Costa Rica, ACG, Alajuela and Guanacaste Provinces, 320–740 m elevation.

#### Ecology

*Hyphantrophaga ciriloumanai* **sp. n.** has been reared 37 times from 12 species of Lepidoptera in the following families: Depressariidae, (*Andasmus* Janzen11, *Andasmus* Janzen16, *Andasmus* Janzen17, *Andasmus* Janzen25, *Andasmus* Janzen26, *Andasmus* Janzen28, *Andasmus* Janzen41, *Andasmus* Janzen42, *Andasmus* Janzen51, *Stenoma* convexicosta, *Stenoma* Janzen06) and Pyralidae, (*Accinctapubes albifasciata*DHJ01, Deuterollyta oediperalisDHJ02); in rain forest, dry forest and dry-rain lowland intergrade.

### Hyphantrophaga danausophaga

Fleming & Wood sp. n.

urn:lsid:zoobank.org:act:0E6B3A67-A03B-4113-9031-78E40CF00FC3

#### Materials

- **Type status:** Holotype. **Occurrence:** occurrenceDetails: http://janzen.sas.upenn.edu; catalogNumber: DHJPAR0010000; recordedBy: D.H. Janzen, W. Hallwachs & Anabelle Cordoba; individualID: DHJPAR0010000; individualCount: 1; sex: male; lifeStage: adult; preparations: pinned; otherCatalogNumbers: ASTAV601-06, 06-SRNP-2806, BOLD:AAA5134; **Taxon:** scientificName: Hyphantrophaga danausophaga; phylum: Arthropoda; class: Insecta; order: Diptera; family: Tachinidae; genus: Hyphantrophaga; specificEpithet: danausophaga; scientificNameAuthorship: Fleming & Wood, 2018; **Location:** continent: Central America; country: Costa Rica; countryCode: CR; stateProvince: Alajuela; county: Sector San Cristobal; locality: Area de Conservacion Guanacaste; verbatimLocality: Sendero Pinyal; verbatimElevation: 630; verbatimLatitude: 10.8716; verbatimLongitude: -85.3933; verbatimCoordinateSystem: Decimal; decimalLatitude: 10.8716; decimalLongitude: -85.3933; **Identification:** identifiedBy: AJ Fleming; dateIdentified: 2017; **Event:** samplingProtocol: Reared from the larva of the Nymphalidae, Danaus plexippus; verbatimEventDate: 04-May-2006; **Record Level:** language: en; institutionCode: CNC; collectionCode: Insects; basisOfRecord: Pinned Specimen
- **Type status:** Paratype. **Occurrence:** occurrenceDetails: http://janzen.sas.upenn.edu; catalogNumber: DHJPAR0005447; recordedBy: D.H. Janzen, W. Hallwachs & Gloria Sihezar; individualID: DHJPAR0005447; individualCount: 1; sex: male; lifeStage: adult; preparations: pinned; otherCatalogNumbers: ASTA567-06, 05-SRNP-6883, BOLD:AAA5134; **Taxon:** scientificName: Hyphantrophaga danausophaga; phylum: Arthropoda; class: Insecta; order: Diptera; family: Tachinidae; genus: Hyphantrophaga; specificEpithet: danausophaga; scientificNameAuthorship: Fleming & Wood, 2018; **Location:** continent: Central America; country: Costa Rica; countryCode: CR; stateProvince: Alajuela; county: Sector San Cristobal; locality: Area de Conservacion Guanacaste; verbatimLocality: Sendero Vivero; verbatimElevation: 730; verbatimLatitude: 10.8674; verbatimLongitude: -85.3874; verbatimCoordinateSystem: Decimal; decimalLatitude: 10.8674; decimalLongitude: -85.3874; **Identification:** identifiedBy: AJ Fleming; dateIdentified: 2017; **Event:** samplingProtocol: Reared from the larva of the Nymphalidae, Danaus plexippus; verbatimEventDate: 30-Nov-2005; **Record Level:** language: en; institutionCode: USNM; collectionCode: Insects; basisOfRecord: Pinned Specimen
- **Type status:** Paratype. **Occurrence:** occurrenceDetails: http://janzen.sas.upenn.edu; catalogNumber: DHJPAR0005539; recordedBy: D.H. Janzen, W. Hallwachs & Jose Alberto Sanchez; individualID: DHJPAR0005539; individualCount: 1; sex: male; lifeStage: adult; preparations: pinned; otherCatalogNumbers: ASTA658-06, 05-SRNP-66253, BOLD:AAA5134; **Taxon:** scientificName: Hyphantrophaga danausophaga; phylum: Arthropoda; class: Insecta; order: Diptera; family: Tachinidae; genus: Hyphantrophaga; specificEpithet: danausophaga; scientificNameAuthorship: Fleming & Wood, 2018; **Location:** continent: Central America; country: Costa Rica; countryCode: CR; stateProvince: Guanacaste; county: Sector Mundo Nuevo; locality: Area de Conservacion Guanacaste; verbatimLocality: Porton Rivas; verbatimElevation: 570; verbatimLatitude: 10.7586; verbatimLongitude: -85.3727; verbatimCoordinateSystem: Decimal; decimalLatitude: 10.7586; decimalLongitude: -85.3727; **Identification:** identifiedBy: AJ Fleming; dateIdentified: 2017; **Event:** samplingProtocol: Reared from the larva of the Nymphalidae, Lycorea atergatis; verbatimEventDate: 06-Jan-2006; **Record Level:** language: en; institutionCode: BMNH; collectionCode: Insects; basisOfRecord: Pinned Specimen
- **Type status:** Paratype. **Occurrence:** occurrenceDetails: http://janzen.sas.upenn.edu; catalogNumber: DHJPAR0006686; recordedBy: D.H. Janzen, W. Hallwachs & Jose Alberto Sanchez; individualID: DHJPAR0006686; individualCount: 1; sex: male; lifeStage: adult; preparations: pinned; otherCatalogNumbers: ASTA864-06, 05-SRNP-66243,; **Taxon:** scientificName: Hyphantrophaga danausophaga; phylum: Arthropoda; class: Insecta; order: Diptera; family: Tachinidae; genus: Hyphantrophaga; specificEpithet: danausophaga; scientificNameAuthorship: Fleming & Wood, 2018; **Location:** continent: Central America; country: Costa Rica; countryCode: CR; stateProvince: Guanacaste; county: Sector Mundo Nuevo; locality: Area de Conservacion Guanacaste; verbatimLocality: Porton Rivas; verbatimElevation: 570; verbatimLatitude: 10.7586; verbatimLongitude: -85.3727; verbatimCoordinateSystem: Decimal; decimalLatitude: 10.7586; decimalLongitude: -85.3727; **Identification:** identifiedBy: AJ Fleming; dateIdentified: 2017; **Event:** samplingProtocol: Reared from the larva of the Nymphalidae, Lycorea atergatis; verbatimEventDate: 31-Dec-2005; **Record Level:** language: en; institutionCode: CNC; collectionCode: Insects; basisOfRecord: Pinned Specimen
- **Type status:** Paratype. **Occurrence:** occurrenceDetails: http://janzen.sas.upenn.edu; catalogNumber: DHJPAR0007382; recordedBy: D.H. Janzen, W. Hallwachs & Osvaldo Espinoza; individualID: DHJPAR0007382; individualCount: 1; sex: female; lifeStage: adult; preparations: pinned; otherCatalogNumbers: ASTAT154-06, 04-SRNP-3571, BOLD:AAA5134; **Taxon:** scientificName: Hyphantrophaga danausophaga; phylum: Arthropoda; class: Insecta; order: Diptera; family: Tachinidae; genus: Hyphantrophaga; specificEpithet: danausophaga; scientificNameAuthorship: Fleming & Wood, 2018; **Location:** continent: Central America; country: Costa Rica; countryCode: CR; stateProvince: Alajuela; county: Sector San Cristobal; locality: Area de Conservacion Guanacaste; verbatimLocality: Puente Palma; verbatimElevation: 460; verbatimLatitude: 10.9163; verbatimLongitude: -85.3787; verbatimCoordinateSystem: Decimal; decimalLatitude: 10.9163; decimalLongitude: -85.3787; **Identification:** identifiedBy: AJ Fleming; dateIdentified: 2017; **Event:** samplingProtocol: Reared from the larva of the Nymphalidae, Danaus plexippus; verbatimEventDate: 11-Aug-2004; **Record Level:** language: en; institutionCode: CNC; collectionCode: Insects; basisOfRecord: Pinned Specimen
- **Type status:** Paratype. **Occurrence:** occurrenceDetails: http://janzen.sas.upenn.edu; catalogNumber: DHJPAR0007412; recordedBy: D.H. Janzen, W. Hallwachs & Gloria Sihezar; individualID: DHJPAR0007412; individualCount: 1; sex: male; lifeStage: adult; preparations: pinned; otherCatalogNumbers: ASTAT184-06, 00-SRNP-474, BOLD:AAA5134; **Taxon:** scientificName: Hyphantrophaga danausophaga; phylum: Arthropoda; class: Insecta; order: Diptera; family: Tachinidae; genus: Hyphantrophaga; specificEpithet: danausophaga; scientificNameAuthorship: Fleming & Wood, 2018; **Location:** continent: Central America; country: Costa Rica; countryCode: CR; stateProvince: Alajuela; county: Sector San Cristobal; locality: Area de Conservacion Guanacaste; verbatimLocality: Vado Rio Cucaracho; verbatimElevation: 640; verbatimLatitude: 10.8702; verbatimLongitude: -85.3915; verbatimCoordinateSystem: Decimal; decimalLatitude: 10.8702; decimalLongitude: -85.3915; **Identification:** identifiedBy: AJ Fleming; dateIdentified: 2017; **Event:** samplingProtocol: Reared from the larva of the Nymphalidae, Danaus plexippus; verbatimEventDate: 16-Mar-2000; **Record Level:** language: en; institutionCode: CNC; collectionCode: Insects; basisOfRecord: Pinned Specimen
- **Type status:** Paratype. **Occurrence:** occurrenceDetails: http://janzen.sas.upenn.edu; catalogNumber: DHJPAR0007413; recordedBy: D.H. Janzen, W. Hallwachs & Osvaldo Espinoza; individualID: DHJPAR0007413; individualCount: 1; sex: female; lifeStage: adult; preparations: pinned; otherCatalogNumbers: ASTAT185-06, 00-SRNP-449, BOLD:AAA5134; **Taxon:** scientificName: Hyphantrophaga danausophaga; phylum: Arthropoda; class: Insecta; order: Diptera; family: Tachinidae; genus: Hyphantrophaga; specificEpithet: danausophaga; scientificNameAuthorship: Fleming & Wood, 2018; **Location:** continent: Central America; country: Costa Rica; countryCode: CR; stateProvince: Alajuela; county: Sector San Cristobal; locality: Area de Conservacion Guanacaste; verbatimLocality: Vado Rio Cucaracho; verbatimElevation: 640; verbatimLatitude: 10.8702; verbatimLongitude: -85.3915; verbatimCoordinateSystem: Decimal; decimalLatitude: 10.8702; decimalLongitude: -85.3915; **Identification:** identifiedBy: AJ Fleming; dateIdentified: 2017; **Event:** samplingProtocol: Reared from the larva of the Nymphalidae, Danaus plexippus; verbatimEventDate: 14-Mar-2000; **Record Level:** language: en; institutionCode: CNC; collectionCode: Insects; basisOfRecord: Pinned Specimen
- **Type status:** Paratype. **Occurrence:** occurrenceDetails: http://janzen.sas.upenn.edu; catalogNumber: DHJPAR0007414; recordedBy: D.H. Janzen, W. Hallwachs & Anabelle Cordoba; individualID: DHJPAR0007414; individualCount: 1; sex: male; lifeStage: adult; preparations: pinned; otherCatalogNumbers: ASTAT186-06, 05-SRNP-4056, BOLD:AAA5134; **Taxon:** scientificName: Hyphantrophaga danausophaga; phylum: Arthropoda; class: Insecta; order: Diptera; family: Tachinidae; genus: Hyphantrophaga; specificEpithet: danausophaga; scientificNameAuthorship: Fleming & Wood, 2018; **Location:** continent: Central America; country: Costa Rica; countryCode: CR; stateProvince: Alajuela; county: Sector San Cristobal; locality: Area de Conservacion Guanacaste; verbatimLocality: Puente Palma; verbatimElevation: 460; verbatimLatitude: 10.9163; verbatimLongitude: -85.3787; verbatimCoordinateSystem: Decimal; decimalLatitude: 10.9163; decimalLongitude: -85.3787; **Identification:** identifiedBy: AJ Fleming; dateIdentified: 2017; **Event:** samplingProtocol: Reared from the larva of the Nymphalidae, Danaus plexippus; verbatimEventDate: 12-Aug-2005; **Record Level:** language: en; institutionCode: CNC; collectionCode: Insects; basisOfRecord: Pinned Specimen
- **Type status:** Paratype. **Occurrence:** occurrenceDetails: http://janzen.sas.upenn.edu; catalogNumber: DHJPAR0007415; recordedBy: D.H. Janzen, W. Hallwachs & Osvaldo Espinoza; individualID: DHJPAR0007415; individualCount: 1; sex: female; lifeStage: adult; preparations: pinned; otherCatalogNumbers: ASTAT187-06, 00-SRNP-618, BOLD:AAA5134; **Taxon:** scientificName: Hyphantrophaga danausophaga; phylum: Arthropoda; class: Insecta; order: Diptera; family: Tachinidae; genus: Hyphantrophaga; specificEpithet: danausophaga; scientificNameAuthorship: Fleming & Wood, 2018; **Location:** continent: Central America; country: Costa Rica; countryCode: CR; stateProvince: Alajuela; county: Sector San Cristobal; locality: Area de Conservacion Guanacaste; verbatimLocality: Vado Rio Cucaracho; verbatimElevation: 640; verbatimLatitude: 10.8702; verbatimLongitude: -85.3915; verbatimCoordinateSystem: Decimal; decimalLatitude: 10.8702; decimalLongitude: -85.3915; **Identification:** identifiedBy: AJ Fleming; dateIdentified: 2017; **Event:** samplingProtocol: Reared from the larva of the Nymphalidae, Danaus plexippus; verbatimEventDate: 24-Mar-2000; **Record Level:** language: en; institutionCode: CNC; collectionCode: Insects; basisOfRecord: Pinned Specimen
- **Type status:** Paratype. **Occurrence:** occurrenceDetails: http://janzen.sas.upenn.edu; catalogNumber: DHJPAR0007416; recordedBy: D.H. Janzen, W. Hallwachs & Osvaldo Espinoza; individualID: DHJPAR0007416; individualCount: 1; sex: female; lifeStage: adult; preparations: pinned; otherCatalogNumbers: ASTAT188-06, 00-SRNP-614, BOLD:AAA5134; **Taxon:** scientificName: Hyphantrophaga danausophaga; phylum: Arthropoda; class: Insecta; order: Diptera; family: Tachinidae; genus: Hyphantrophaga; specificEpithet: danausophaga; scientificNameAuthorship: Fleming & Wood, 2018; **Location:** continent: Central America; country: Costa Rica; countryCode: CR; stateProvince: Alajuela; county: Sector San Cristobal; locality: Area de Conservacion Guanacaste; verbatimLocality: Vado Rio Cucaracho; verbatimElevation: 640; verbatimLatitude: 10.8702; verbatimLongitude: -85.3915; verbatimCoordinateSystem: Decimal; decimalLatitude: 10.8702; decimalLongitude: -85.3915; **Identification:** identifiedBy: AJ Fleming; dateIdentified: 2017; **Event:** samplingProtocol: Reared from the larva of the Nymphalidae, Danaus plexippus; verbatimEventDate: 21-Mar-2000; **Record Level:** language: en; institutionCode: CNC; collectionCode: Insects; basisOfRecord: Pinned Specimen
- **Type status:** Paratype. **Occurrence:** occurrenceDetails: http://janzen.sas.upenn.edu; catalogNumber: DHJPAR0007417; recordedBy: D.H. Janzen, W. Hallwachs & Gloria Sihezar; individualID: DHJPAR0007417; individualCount: 1; sex: male; lifeStage: adult; preparations: pinned; otherCatalogNumbers: ASTAT189-06, 98-SRNP-7256, BOLD:AAA5134; **Taxon:** scientificName: Hyphantrophaga danausophaga; phylum: Arthropoda; class: Insecta; order: Diptera; family: Tachinidae; genus: Hyphantrophaga; specificEpithet: danausophaga; scientificNameAuthorship: Fleming & Wood, 2018; **Location:** continent: Central America; country: Costa Rica; countryCode: CR; stateProvince: Alajuela; county: Sector San Cristobal; locality: Area de Conservacion Guanacaste; verbatimLocality: Sendero Pinyal; verbatimElevation: 630; verbatimLatitude: 10.8716; verbatimLongitude: -85.3933; verbatimCoordinateSystem: Decimal; decimalLatitude: 10.8716; decimalLongitude: -85.3933; **Identification:** identifiedBy: AJ Fleming; dateIdentified: 2017; **Event:** samplingProtocol: Reared from the larva of the Nymphalidae, Danaus plexippus; verbatimEventDate: 09-Jul-1998; **Record Level:** language: en; institutionCode: CNC; collectionCode: Insects; basisOfRecord: Pinned Specimen
- **Type status:** Paratype. **Occurrence:** occurrenceDetails: http://janzen.sas.upenn.edu; catalogNumber: DHJPAR0007418; recordedBy: D.H. Janzen, W. Hallwachs & Gloria Sihezar; individualID: DHJPAR0007418; individualCount: 1; sex: female; lifeStage: adult; preparations: pinned; otherCatalogNumbers: ASTAT190-06, 98-SRNP-7255,; **Taxon:** scientificName: Hyphantrophaga danausophaga; phylum: Arthropoda; class: Insecta; order: Diptera; family: Tachinidae; genus: Hyphantrophaga; specificEpithet: danausophaga; scientificNameAuthorship: Fleming & Wood, 2018; **Location:** continent: Central America; country: Costa Rica; countryCode: CR; stateProvince: Alajuela; county: Sector San Cristobal; locality: Area de Conservacion Guanacaste; verbatimLocality: Sendero Pinyal; verbatimElevation: 630; verbatimLatitude: 10.8716; verbatimLongitude: -85.3933; verbatimCoordinateSystem: Decimal; decimalLatitude: 10.8716; decimalLongitude: -85.3933; **Identification:** identifiedBy: AJ Fleming; dateIdentified: 2017; **Event:** samplingProtocol: Reared from the larva of the Nymphalidae, Danaus plexippus; verbatimEventDate: 09-Aug-1998; **Record Level:** language: en; institutionCode: CNC; collectionCode: Insects; basisOfRecord: Pinned Specimen
- **Type status:** Paratype. **Occurrence:** occurrenceDetails: http://janzen.sas.upenn.edu; catalogNumber: DHJPAR0007419; recordedBy: D.H. Janzen, W. Hallwachs & Carolina Cano; individualID: DHJPAR0007419; individualCount: 1; sex: male; lifeStage: adult; preparations: pinned; otherCatalogNumbers: ASTAT191-06, 00-SRNP-412, BOLD:AAA5134; **Taxon:** scientificName: Hyphantrophaga danausophaga; phylum: Arthropoda; class: Insecta; order: Diptera; family: Tachinidae; genus: Hyphantrophaga; specificEpithet: danausophaga; scientificNameAuthorship: Fleming & Wood, 2018; **Location:** continent: Central America; country: Costa Rica; countryCode: CR; stateProvince: Alajuela; county: Sector San Cristobal; locality: Area de Conservacion Guanacaste; verbatimLocality: Melina Bufalo; verbatimElevation: 560; verbatimLatitude: 10.884; verbatimLongitude: -85.386; verbatimCoordinateSystem: Decimal; decimalLatitude: 10.884; decimalLongitude: -85.386; **Identification:** identifiedBy: AJ Fleming; dateIdentified: 2017; **Event:** samplingProtocol: Reared from the larva of the Nymphalidae, Danaus plexippus; verbatimEventDate: 16-Mar-2000; **Record Level:** language: en; institutionCode: CNC; collectionCode: Insects; basisOfRecord: Pinned Specimen
- **Type status:** Paratype. **Occurrence:** occurrenceDetails: http://janzen.sas.upenn.edu; catalogNumber: DHJPAR0007420; recordedBy: D.H. Janzen, W. Hallwachs & Freddy Quesada; individualID: DHJPAR0007420; individualCount: 1; sex: female; lifeStage: adult; preparations: pinned; otherCatalogNumbers: ASTAT192-06, 00-SRNP-774, BOLD:AAA5134; **Taxon:** scientificName: Hyphantrophaga danausophaga; phylum: Arthropoda; class: Insecta; order: Diptera; family: Tachinidae; genus: Hyphantrophaga; specificEpithet: danausophaga; scientificNameAuthorship: Fleming & Wood, 2018; **Location:** continent: Central America; country: Costa Rica; countryCode: CR; stateProvince: Alajuela; county: Sector San Cristobal; locality: Area de Conservacion Guanacaste; verbatimLocality: Quebrada Cementerio; verbatimElevation: 700; verbatimLatitude: 10.8712; verbatimLongitude: -85.3875; verbatimCoordinateSystem: Decimal; decimalLatitude: 10.8712; decimalLongitude: -85.3875; **Identification:** identifiedBy: AJ Fleming; dateIdentified: 2017; **Event:** samplingProtocol: Reared from the larva of the Nymphalidae, Danaus plexippus; verbatimEventDate: 05-Apr-2000; **Record Level:** language: en; institutionCode: CNC; collectionCode: Insects; basisOfRecord: Pinned Specimen
- **Type status:** Paratype. **Occurrence:** occurrenceDetails: http://janzen.sas.upenn.edu; catalogNumber: DHJPAR0007421; recordedBy: D.H. Janzen, W. Hallwachs & Gloria Sihezar; individualID: DHJPAR0007421; individualCount: 1; sex: male; lifeStage: adult; preparations: pinned; otherCatalogNumbers: ASTAT193-06, 00-SRNP-473, BOLD:AAA5134; **Taxon:** scientificName: Hyphantrophaga danausophaga; phylum: Arthropoda; class: Insecta; order: Diptera; family: Tachinidae; genus: Hyphantrophaga; specificEpithet: danausophaga; scientificNameAuthorship: Fleming & Wood, 2018; **Location:** continent: Central America; country: Costa Rica; countryCode: CR; stateProvince: Alajuela; county: Sector San Cristobal; locality: Area de Conservacion Guanacaste; verbatimLocality: Vado Rio Cucaracho; verbatimElevation: 640; verbatimLatitude: 10.8702; verbatimLongitude: -85.3915; verbatimCoordinateSystem: Decimal; decimalLatitude: 10.8702; decimalLongitude: -85.3915; **Identification:** identifiedBy: AJ Fleming; dateIdentified: 2017; **Event:** samplingProtocol: Reared from the larva of the Nymphalidae, Danaus plexippus; verbatimEventDate: 14-Mar-2000; **Record Level:** language: en; institutionCode: CNC; collectionCode: Insects; basisOfRecord: Pinned Specimen
- **Type status:** Paratype. **Occurrence:** occurrenceDetails: http://janzen.sas.upenn.edu; catalogNumber: DHJPAR0007422; recordedBy: D.H. Janzen, W. Hallwachs & Carolina Cano; individualID: DHJPAR0007422; individualCount: 1; sex: female; lifeStage: adult; preparations: pinned; otherCatalogNumbers: ASTAT194-06, 00-SRNP-551, BOLD:AAA5134; **Taxon:** scientificName: Hyphantrophaga danausophaga; phylum: Arthropoda; class: Insecta; order: Diptera; family: Tachinidae; genus: Hyphantrophaga; specificEpithet: danausophaga; scientificNameAuthorship: Fleming & Wood, 2018; **Location:** continent: Central America; country: Costa Rica; countryCode: CR; stateProvince: Alajuela; county: Sector San Cristobal; locality: Area de Conservacion Guanacaste; verbatimLocality: Vado Rio Cucaracho; verbatimElevation: 640; verbatimLatitude: 10.8702; verbatimLongitude: -85.3915; verbatimCoordinateSystem: Decimal; decimalLatitude: 10.8702; decimalLongitude: -85.3915; **Identification:** identifiedBy: AJ Fleming; dateIdentified: 2017; **Event:** samplingProtocol: Reared from the larva of the Nymphalidae, Danaus plexippus; verbatimEventDate: 19-Mar-2000; **Record Level:** language: en; institutionCode: CNC; collectionCode: Insects; basisOfRecord: Pinned Specimen
- **Type status:** Paratype. **Occurrence:** occurrenceDetails: http://janzen.sas.upenn.edu; catalogNumber: DHJPAR0007423; recordedBy: D.H. Janzen, W. Hallwachs & Carolina Cano; individualID: DHJPAR0007423; individualCount: 1; sex: male; lifeStage: adult; preparations: pinned; otherCatalogNumbers: ASTAT195-06, 00-SRNP-413,; **Taxon:** scientificName: Hyphantrophaga danausophaga; phylum: Arthropoda; class: Insecta; order: Diptera; family: Tachinidae; genus: Hyphantrophaga; specificEpithet: danausophaga; scientificNameAuthorship: Fleming & Wood, 2018; **Location:** continent: Central America; country: Costa Rica; countryCode: CR; stateProvince: Alajuela; county: Sector San Cristobal; locality: Area de Conservacion Guanacaste; verbatimLocality: Melina Bufalo; verbatimElevation: 560; verbatimLatitude: 10.884; verbatimLongitude: -85.386; verbatimCoordinateSystem: Decimal; decimalLatitude: 10.884; decimalLongitude: -85.386; **Identification:** identifiedBy: AJ Fleming; dateIdentified: 2017; **Event:** samplingProtocol: Reared from the larva of the Nymphalidae, Danaus plexippus; verbatimEventDate: 19-Mar-2000; **Record Level:** language: en; institutionCode: CNC; collectionCode: Insects; basisOfRecord: Pinned Specimen
- **Type status:** Paratype. **Occurrence:** occurrenceDetails: http://janzen.sas.upenn.edu; catalogNumber: DHJPAR0007424; recordedBy: D.H. Janzen, W. Hallwachs & Osvaldo Espinoza; individualID: DHJPAR0007424; individualCount: 1; sex: male; lifeStage: adult; preparations: pinned; otherCatalogNumbers: ASTAT196-06, 00-SRNP-446, BOLD:AAA5134; **Taxon:** scientificName: Hyphantrophaga danausophaga; phylum: Arthropoda; class: Insecta; order: Diptera; family: Tachinidae; genus: Hyphantrophaga; specificEpithet: danausophaga; scientificNameAuthorship: Fleming & Wood, 2018; **Location:** continent: Central America; country: Costa Rica; countryCode: CR; stateProvince: Alajuela; county: Sector San Cristobal; locality: Area de Conservacion Guanacaste; verbatimLocality: Vado Rio Cucaracho; verbatimElevation: 640; verbatimLatitude: 10.8702; verbatimLongitude: -85.3915; verbatimCoordinateSystem: Decimal; decimalLatitude: 10.8702; decimalLongitude: -85.3915; **Identification:** identifiedBy: AJ Fleming; dateIdentified: 2017; **Event:** samplingProtocol: Reared from the larva of the Nymphalidae, Danaus plexippus; verbatimEventDate: 05-Mar-2000; **Record Level:** language: en; institutionCode: CNC; collectionCode: Insects; basisOfRecord: Pinned Specimen
- **Type status:** Paratype. **Occurrence:** occurrenceDetails: http://janzen.sas.upenn.edu; catalogNumber: DHJPAR0007425; recordedBy: D.H. Janzen, W. Hallwachs & Harry Ramirez; individualID: DHJPAR0007425; individualCount: 1; sex: female; lifeStage: adult; preparations: pinned; otherCatalogNumbers: ASTAT197-06, 04-SRNP-47445,; **Taxon:** scientificName: Hyphantrophaga danausophaga; phylum: Arthropoda; class: Insecta; order: Diptera; family: Tachinidae; genus: Hyphantrophaga; specificEpithet: danausophaga; scientificNameAuthorship: Fleming & Wood, 2018; **Location:** continent: Central America; country: Costa Rica; countryCode: CR; stateProvince: Guanacaste; county: Sector Cacao; locality: Area de Conservacion Guanacaste; verbatimLocality: Quebrada Otilio; verbatimElevation: 550; verbatimLatitude: 10.89; verbatimLongitude: -85.4797; verbatimCoordinateSystem: Decimal; decimalLatitude: 10.89; decimalLongitude: -85.4797; **Identification:** identifiedBy: AJ Fleming; dateIdentified: 2017; **Event:** samplingProtocol: Reared from the larva of the Nymphalidae, Lycorea atergatis; verbatimEventDate: 18-Aug-2004; **Record Level:** language: en; institutionCode: CNC; collectionCode: Insects; basisOfRecord: Pinned Specimen
- **Type status:** Paratype. **Occurrence:** occurrenceDetails: http://janzen.sas.upenn.edu; catalogNumber: DHJPAR0007426; recordedBy: D.H. Janzen, W. Hallwachs & Carolina Cano; individualID: DHJPAR0007426; individualCount: 1; sex: male; lifeStage: adult; preparations: pinned; otherCatalogNumbers: ASTAT198-06, 00-SRNP-410,; **Taxon:** scientificName: Hyphantrophaga danausophaga; phylum: Arthropoda; class: Insecta; order: Diptera; family: Tachinidae; genus: Hyphantrophaga; specificEpithet: danausophaga; scientificNameAuthorship: Fleming & Wood, 2018; **Location:** continent: Central America; country: Costa Rica; countryCode: CR; stateProvince: Alajuela; county: Sector San Cristobal; locality: Area de Conservacion Guanacaste; verbatimLocality: Melina Bufalo; verbatimElevation: 560; verbatimLatitude: 10.884; verbatimLongitude: -85.386; verbatimCoordinateSystem: Decimal; decimalLatitude: 10.884; decimalLongitude: -85.386; **Identification:** identifiedBy: AJ Fleming; dateIdentified: 2017; **Event:** samplingProtocol: Reared from the larva of the Nymphalidae, Danaus plexippus; verbatimEventDate: 17-Mar-2000; **Record Level:** language: en; institutionCode: CNC; collectionCode: Insects; basisOfRecord: Pinned Specimen
- **Type status:** Paratype. **Occurrence:** occurrenceDetails: http://janzen.sas.upenn.edu; catalogNumber: DHJPAR0007427; recordedBy: D.H. Janzen, W. Hallwachs & Osvaldo Espinoza; individualID: DHJPAR0007427; individualCount: 1; sex: male; lifeStage: adult; preparations: pinned; otherCatalogNumbers: ASTAT199-06, 00-SRNP-780, BOLD:AAA5134; **Taxon:** scientificName: Hyphantrophaga danausophaga; phylum: Arthropoda; class: Insecta; order: Diptera; family: Tachinidae; genus: Hyphantrophaga; specificEpithet: danausophaga; scientificNameAuthorship: Fleming & Wood, 2018; **Location:** continent: Central America; country: Costa Rica; countryCode: CR; stateProvince: Alajuela; county: Sector San Cristobal; locality: Area de Conservacion Guanacaste; verbatimLocality: Quebrada Cementerio; verbatimElevation: 700; verbatimLatitude: 10.8712; verbatimLongitude: -85.3875; verbatimCoordinateSystem: Decimal; decimalLatitude: 10.8712; decimalLongitude: -85.3875; **Identification:** identifiedBy: AJ Fleming; dateIdentified: 2017; **Event:** samplingProtocol: Reared from the larva of the Nymphalidae, Danaus plexippus; verbatimEventDate: 05-Apr-2000; **Record Level:** language: en; institutionCode: CNC; collectionCode: Insects; basisOfRecord: Pinned Specimen
- **Type status:** Paratype. **Occurrence:** occurrenceDetails: http://janzen.sas.upenn.edu; catalogNumber: DHJPAR0007428; recordedBy: D.H. Janzen, W. Hallwachs & Carolina Cano; individualID: DHJPAR0007428; individualCount: 1; sex: female; lifeStage: adult; preparations: pinned; otherCatalogNumbers: ASTAT200-06, 00-SRNP-400, BOLD:AAA5134; **Taxon:** scientificName: Hyphantrophaga danausophaga; phylum: Arthropoda; class: Insecta; order: Diptera; family: Tachinidae; genus: Hyphantrophaga; specificEpithet: danausophaga; scientificNameAuthorship: Fleming & Wood, 2018; **Location:** continent: Central America; country: Costa Rica; countryCode: CR; stateProvince: Alajuela; county: Sector San Cristobal; locality: Area de Conservacion Guanacaste; verbatimLocality: Sendero Vivero; verbatimElevation: 730; verbatimLatitude: 10.8674; verbatimLongitude: -85.3874; verbatimCoordinateSystem: Decimal; decimalLatitude: 10.8674; decimalLongitude: -85.3874; **Identification:** identifiedBy: AJ Fleming; dateIdentified: 2017; **Event:** samplingProtocol: Reared from the larva of the Nymphalidae, Danaus plexippus; verbatimEventDate: 13-Mar-2000; **Record Level:** language: en; institutionCode: CNC; collectionCode: Insects; basisOfRecord: Pinned Specimen
- **Type status:** Paratype. **Occurrence:** occurrenceDetails: http://janzen.sas.upenn.edu; catalogNumber: DHJPAR0007429; recordedBy: D.H. Janzen, W. Hallwachs & Osvaldo Espinoza; individualID: DHJPAR0007429; individualCount: 1; sex: male; lifeStage: adult; preparations: pinned; otherCatalogNumbers: ASTAT201-06, 00-SRNP-477,; **Taxon:** scientificName: Hyphantrophaga danausophaga; phylum: Arthropoda; class: Insecta; order: Diptera; family: Tachinidae; genus: Hyphantrophaga; specificEpithet: danausophaga; scientificNameAuthorship: Fleming & Wood, 2018; **Location:** continent: Central America; country: Costa Rica; countryCode: CR; stateProvince: Alajuela; county: Sector San Cristobal; locality: Area de Conservacion Guanacaste; verbatimLocality: Vado Rio Cucaracho; verbatimElevation: 640; verbatimLatitude: 10.8702; verbatimLongitude: -85.3915; verbatimCoordinateSystem: Decimal; decimalLatitude: 10.8702; decimalLongitude: -85.3915; **Identification:** identifiedBy: AJ Fleming; dateIdentified: 2017; **Event:** samplingProtocol: Reared from the larva of the Nymphalidae, Danaus plexippus; verbatimEventDate: 21-Mar-2000; **Record Level:** language: en; institutionCode: CNC; collectionCode: Insects; basisOfRecord: Pinned Specimen
- **Type status:** Paratype. **Occurrence:** occurrenceDetails: http://janzen.sas.upenn.edu; catalogNumber: DHJPAR0007430; recordedBy: D.H. Janzen, W. Hallwachs & Harry Ramirez; individualID: DHJPAR0007430; individualCount: 1; sex: male; lifeStage: adult; preparations: pinned; otherCatalogNumbers: ASTAT202-06, 02-SRNP-8502,; **Taxon:** scientificName: Hyphantrophaga danausophaga; phylum: Arthropoda; class: Insecta; order: Diptera; family: Tachinidae; genus: Hyphantrophaga; specificEpithet: danausophaga; scientificNameAuthorship: Fleming & Wood, 2018; **Location:** continent: Central America; country: Costa Rica; countryCode: CR; stateProvince: Guanacaste; county: Sector Cacao; locality: Area de Conservacion Guanacaste; verbatimLocality: Sendero Maritza; verbatimElevation: 760; verbatimLatitude: 10.9364; verbatimLongitude: -85.4776; verbatimCoordinateSystem: Decimal; decimalLatitude: 10.9364; decimalLongitude: -85.4776; **Identification:** identifiedBy: AJ Fleming; dateIdentified: 2017; **Event:** samplingProtocol: Reared from the larva of the Nymphalidae, Danaus plexippus; verbatimEventDate: 18-Apr-2002; **Record Level:** language: en; institutionCode: CNC; collectionCode: Insects; basisOfRecord: Pinned Specimen
- **Type status:** Paratype. **Occurrence:** occurrenceDetails: http://janzen.sas.upenn.edu; catalogNumber: DHJPAR0007431; recordedBy: D.H. Janzen, W. Hallwachs & Guillermo Pereira; individualID: DHJPAR0007431; individualCount: 1; sex: female; lifeStage: adult; preparations: pinned; otherCatalogNumbers: ASTAT203-06, 02-SRNP-5392, BOLD:AAA5134; **Taxon:** scientificName: Hyphantrophaga danausophaga; phylum: Arthropoda; class: Insecta; order: Diptera; family: Tachinidae; genus: Hyphantrophaga; specificEpithet: danausophaga; scientificNameAuthorship: Fleming & Wood, 2018; **Location:** continent: Central America; country: Costa Rica; countryCode: CR; stateProvince: Guanacaste; county: Sector Del Oro; locality: Area de Conservacion Guanacaste; verbatimLocality: Quebrada Romero; verbatimElevation: 490; verbatimLatitude: 11.0052; verbatimLongitude: -85.474; verbatimCoordinateSystem: Decimal; decimalLatitude: 11.0052; decimalLongitude: -85.474; **Identification:** identifiedBy: AJ Fleming; dateIdentified: 2017; **Event:** samplingProtocol: Reared from the larva of the Nymphalidae, Danaus plexippus; verbatimEventDate: 14-Mar-2002; **Record Level:** language: en; institutionCode: CNC; collectionCode: Insects; basisOfRecord: Pinned Specimen
- **Type status:** Paratype. **Occurrence:** occurrenceDetails: http://janzen.sas.upenn.edu; catalogNumber: DHJPAR0007432; recordedBy: D.H. Janzen, W. Hallwachs & Fraysi Vargas; individualID: DHJPAR0007432; individualCount: 1; sex: male; lifeStage: adult; preparations: pinned; otherCatalogNumbers: ASTAT204-06, 02-SRNP-6987,; **Taxon:** scientificName: Hyphantrophaga danausophaga; phylum: Arthropoda; class: Insecta; order: Diptera; family: Tachinidae; genus: Hyphantrophaga; specificEpithet: danausophaga; scientificNameAuthorship: Fleming & Wood, 2018; **Location:** continent: Central America; country: Costa Rica; countryCode: CR; stateProvince: Alajuela; county: Sector Rincon Rain Forest; locality: Area de Conservacion Guanacaste; verbatimLocality: Estacion Caribe; verbatimElevation: 415; verbatimLatitude: 10.9019; verbatimLongitude: -85.2749; verbatimCoordinateSystem: Decimal; decimalLatitude: 10.9019; decimalLongitude: -85.2749; **Identification:** identifiedBy: AJ Fleming; dateIdentified: 2017; **Event:** samplingProtocol: Reared from the larva of the Nymphalidae, Danaus plexippus; verbatimEventDate: 09-May-2002; **Record Level:** language: en; institutionCode: CNC; collectionCode: Insects; basisOfRecord: Pinned Specimen
- **Type status:** Paratype. **Occurrence:** occurrenceDetails: http://janzen.sas.upenn.edu; catalogNumber: DHJPAR0007433; recordedBy: D.H. Janzen, W. Hallwachs & Freddy Quesada; individualID: DHJPAR0007433; individualCount: 1; lifeStage: adult; preparations: pinned; otherCatalogNumbers: ASTAT205-06, 03-SRNP-22759, BOLD:AAA5134; **Taxon:** scientificName: Hyphantrophaga danausophaga; phylum: Arthropoda; class: Insecta; order: Diptera; family: Tachinidae; genus: Hyphantrophaga; specificEpithet: danausophaga; scientificNameAuthorship: Fleming & Wood, 2018; **Location:** continent: Central America; country: Costa Rica; countryCode: CR; stateProvince: Guanacaste; county: Sector Cacao; locality: Area de Conservacion Guanacaste; verbatimLocality: Estacion Cacao; verbatimElevation: 1150; verbatimLatitude: 10.9269; verbatimLongitude: -85.4682; verbatimCoordinateSystem: Decimal; decimalLatitude: 10.9269; decimalLongitude: -85.4682; **Identification:** identifiedBy: AJ Fleming; dateIdentified: 2017; **Event:** samplingProtocol: Reared from the larva of the Nymphalidae, Danaus plexippus; verbatimEventDate: 17-Oct-2003; **Record Level:** language: en; institutionCode: CNC; collectionCode: Insects; basisOfRecord: Pinned Specimen
- **Type status:** Paratype. **Occurrence:** occurrenceDetails: http://janzen.sas.upenn.edu; catalogNumber: DHJPAR0007434; recordedBy: D.H. Janzen, W. Hallwachs & Lucia Rios; individualID: DHJPAR0007434; individualCount: 1; sex: female; lifeStage: adult; preparations: pinned; otherCatalogNumbers: ASTAT206-06, 04-SRNP-22623, BOLD:AAA5134; **Taxon:** scientificName: Hyphantrophaga danausophaga; phylum: Arthropoda; class: Insecta; order: Diptera; family: Tachinidae; genus: Hyphantrophaga; specificEpithet: danausophaga; scientificNameAuthorship: Fleming & Wood, 2018; **Location:** continent: Central America; country: Costa Rica; countryCode: CR; stateProvince: Guanacaste; county: Sector Del Oro; locality: Area de Conservacion Guanacaste; verbatimLocality: Quebrada Raiz; verbatimElevation: 280; verbatimLatitude: 11.0287; verbatimLongitude: -85.4867; verbatimCoordinateSystem: Decimal; decimalLatitude: 11.0287; decimalLongitude: -85.4867; **Identification:** identifiedBy: AJ Fleming; dateIdentified: 2017; **Event:** samplingProtocol: Reared from the larva of the Nymphalidae, Danaus plexippus; verbatimEventDate: 28-Jun-2004; **Record Level:** language: en; institutionCode: CNC; collectionCode: Insects; basisOfRecord: Pinned Specimen
- **Type status:** Paratype. **Occurrence:** occurrenceDetails: http://janzen.sas.upenn.edu; catalogNumber: DHJPAR0007435; recordedBy: D.H. Janzen, W. Hallwachs & Minor Carmona; individualID: DHJPAR0007435; individualCount: 1; sex: female; lifeStage: adult; preparations: pinned; otherCatalogNumbers: ASTAT207-06, 03-SRNP-31832,; **Taxon:** scientificName: Hyphantrophaga danausophaga; phylum: Arthropoda; class: Insecta; order: Diptera; family: Tachinidae; genus: Hyphantrophaga; specificEpithet: danausophaga; scientificNameAuthorship: Fleming & Wood, 2018; **Location:** continent: Central America; country: Costa Rica; countryCode: CR; stateProvince: Alajuela; county: Sector Rincon Rain Forest; locality: Area de Conservacion Guanacaste; verbatimLocality: San Lucas; verbatimElevation: 320; verbatimLatitude: 10.9185; verbatimLongitude: -85.3034; verbatimCoordinateSystem: Decimal; decimalLatitude: 10.9185; decimalLongitude: -85.3034; **Identification:** identifiedBy: AJ Fleming; dateIdentified: 2017; **Event:** samplingProtocol: Reared from the larva of the Nymphalidae, Danaus plexippus; verbatimEventDate: 16-Jan-2004; **Record Level:** language: en; institutionCode: CNC; collectionCode: Insects; basisOfRecord: Pinned Specimen
- **Type status:** Paratype. **Occurrence:** occurrenceDetails: http://janzen.sas.upenn.edu; catalogNumber: DHJPAR0007436; recordedBy: D.H. Janzen, W. Hallwachs & Manuel Pereira; individualID: DHJPAR0007436; individualCount: 1; sex: female; lifeStage: adult; preparations: pinned; otherCatalogNumbers: ASTAT208-06, 05-SRNP-45425, BOLD:AAA5134; **Taxon:** scientificName: Hyphantrophaga danausophaga; phylum: Arthropoda; class: Insecta; order: Diptera; family: Tachinidae; genus: Hyphantrophaga; specificEpithet: danausophaga; scientificNameAuthorship: Fleming & Wood, 2018; **Location:** continent: Central America; country: Costa Rica; countryCode: CR; stateProvince: Guanacaste; county: Sector Cacao; locality: Area de Conservacion Guanacaste; verbatimLocality: Cuesta Caimito; verbatimElevation: 640; verbatimLatitude: 10.8908; verbatimLongitude: -85.4719; verbatimCoordinateSystem: Decimal; decimalLatitude: 10.8908; decimalLongitude: -85.4719; **Identification:** identifiedBy: AJ Fleming; dateIdentified: 2017; **Event:** samplingProtocol: Reared from the larva of the Nymphalidae, Danaus plexippus; verbatimEventDate: 26-Jun-2005; **Record Level:** language: en; institutionCode: CNC; collectionCode: Insects; basisOfRecord: Pinned Specimen
- **Type status:** Paratype. **Occurrence:** occurrenceDetails: http://janzen.sas.upenn.edu; catalogNumber: DHJPAR0007437; recordedBy: D.H. Janzen, W. Hallwachs & Minor Carmona; individualID: DHJPAR0007437; individualCount: 1; sex: male; lifeStage: adult; preparations: pinned; otherCatalogNumbers: ASTAT209-06, 05-SRNP-41041, BOLD:AAA5134; **Taxon:** scientificName: Hyphantrophaga danausophaga; phylum: Arthropoda; class: Insecta; order: Diptera; family: Tachinidae; genus: Hyphantrophaga; specificEpithet: danausophaga; scientificNameAuthorship: Fleming & Wood, 2018; **Location:** continent: Central America; country: Costa Rica; countryCode: CR; stateProvince: Alajuela; county: Sector Rincon Rain Forest; locality: Area de Conservacion Guanacaste; verbatimLocality: Sendero Venado; verbatimElevation: 420; verbatimLatitude: 10.8968; verbatimLongitude: -85.27; verbatimCoordinateSystem: Decimal; decimalLatitude: 10.8968; decimalLongitude: -85.27; **Identification:** identifiedBy: AJ Fleming; dateIdentified: 2017; **Event:** samplingProtocol: Reared from the larva of the Nymphalidae, Danaus plexippus; verbatimEventDate: 17-May-2005; **Record Level:** language: en; institutionCode: CNC; collectionCode: Insects; basisOfRecord: Pinned Specimen
- **Type status:** Paratype. **Occurrence:** occurrenceDetails: http://janzen.sas.upenn.edu; catalogNumber: DHJPAR0007438; recordedBy: D.H. Janzen, W. Hallwachs & Elda Araya; individualID: DHJPAR0007438; individualCount: 1; sex: male; lifeStage: adult; preparations: pinned; otherCatalogNumbers: ASTAT210-06, 05-SRNP-744, BOLD:AAA5134; **Taxon:** scientificName: Hyphantrophaga danausophaga; phylum: Arthropoda; class: Insecta; order: Diptera; family: Tachinidae; genus: Hyphantrophaga; specificEpithet: danausophaga; scientificNameAuthorship: Fleming & Wood, 2018; **Location:** continent: Central America; country: Costa Rica; countryCode: CR; stateProvince: Alajuela; county: Sector San Cristobal; locality: Area de Conservacion Guanacaste; verbatimLocality: Sitio San Geronimo; verbatimElevation: 680; verbatimLatitude: 10.873; verbatimLongitude: -85.3814; verbatimCoordinateSystem: Decimal; decimalLatitude: 10.873; decimalLongitude: -85.3814; **Identification:** identifiedBy: AJ Fleming; dateIdentified: 2017; **Event:** samplingProtocol: Reared from the larva of the Nymphalidae, Danaus plexippus; verbatimEventDate: 12-Mar-2005; **Record Level:** language: en; institutionCode: CNC; collectionCode: Insects; basisOfRecord: Pinned Specimen
- **Type status:** Paratype. **Occurrence:** occurrenceDetails: http://janzen.sas.upenn.edu; catalogNumber: DHJPAR0007439; recordedBy: D.H. Janzen, W. Hallwachs & Minor Carmona; individualID: DHJPAR0007439; individualCount: 1; sex: male; lifeStage: adult; preparations: pinned; otherCatalogNumbers: ASTAT211-06, 03-SRNP-5767, BOLD:AAA5134; **Taxon:** scientificName: Hyphantrophaga danausophaga; phylum: Arthropoda; class: Insecta; order: Diptera; family: Tachinidae; genus: Hyphantrophaga; specificEpithet: danausophaga; scientificNameAuthorship: Fleming & Wood, 2018; **Location:** continent: Central America; country: Costa Rica; countryCode: CR; stateProvince: Alajuela; county: Sector San Cristobal; locality: Area de Conservacion Guanacaste; verbatimLocality: Rio Blanco Abajo; verbatimElevation: 500; verbatimLatitude: 10.9004; verbatimLongitude: -85.3725; verbatimCoordinateSystem: Decimal; decimalLatitude: 10.9004; decimalLongitude: -85.3725; **Identification:** identifiedBy: AJ Fleming; dateIdentified: 2017; **Event:** samplingProtocol: Reared from the larva of the Nymphalidae, Danaus plexippus; verbatimEventDate: 09-Apr-2003; **Record Level:** language: en; institutionCode: CNC; collectionCode: Insects; basisOfRecord: Pinned Specimen
- **Type status:** Paratype. **Occurrence:** occurrenceDetails: http://janzen.sas.upenn.edu; catalogNumber: DHJPAR0007122; recordedBy: D.H. Janzen, W. Hallwachs & Elda Araya; individualID: DHJPAR0007122; individualCount: 1; lifeStage: adult; preparations: pinned; otherCatalogNumbers: ASTAV364-06, 06-SRNP-1738, BOLD:AAA5134; **Taxon:** scientificName: Hyphantrophaga danausophaga; phylum: Arthropoda; class: Insecta; order: Diptera; family: Tachinidae; genus: Hyphantrophaga; specificEpithet: danausophaga; scientificNameAuthorship: Fleming & Wood, 2018; **Location:** continent: Central America; country: Costa Rica; countryCode: CR; stateProvince: Alajuela; county: Sector San Cristobal; locality: Area de Conservacion Guanacaste; verbatimLocality: Vado Rio Cucaracho; verbatimElevation: 640; verbatimLatitude: 10.8702; verbatimLongitude: -85.3915; verbatimCoordinateSystem: Decimal; decimalLatitude: 10.8702; decimalLongitude: -85.3915; **Identification:** identifiedBy: AJ Fleming; dateIdentified: 2017; **Event:** samplingProtocol: Reared from the larva of the Nymphalidae, Danaus plexippus; verbatimEventDate: 25-Mar-2006; **Record Level:** language: en; institutionCode: CNC; collectionCode: Insects; basisOfRecord: Pinned Specimen
- **Type status:** Paratype. **Occurrence:** occurrenceDetails: http://janzen.sas.upenn.edu; catalogNumber: DHJPAR0009992; recordedBy: D.H. Janzen, W. Hallwachs & Anabelle Cordoba; individualID: DHJPAR0009992; individualCount: 1; sex: male; lifeStage: adult; preparations: pinned; otherCatalogNumbers: ASTAV593-06, 06-SRNP-2664, BOLD:AAA5134; **Taxon:** scientificName: Hyphantrophaga danausophaga; phylum: Arthropoda; class: Insecta; order: Diptera; family: Tachinidae; genus: Hyphantrophaga; specificEpithet: danausophaga; scientificNameAuthorship: Fleming & Wood, 2018; **Location:** continent: Central America; country: Costa Rica; countryCode: CR; stateProvince: Alajuela; county: Sector San Cristobal; locality: Area de Conservacion Guanacaste; verbatimLocality: Tajo Angeles; verbatimElevation: 540; verbatimLatitude: 10.8647; verbatimLongitude: -85.4153; verbatimCoordinateSystem: Decimal; decimalLatitude: 10.8647; decimalLongitude: -85.4153; **Identification:** identifiedBy: AJ Fleming; dateIdentified: 2017; **Event:** samplingProtocol: Reared from the larva of the Nymphalidae, Danaus plexippus; verbatimEventDate: 25-Apr-2006; **Record Level:** language: en; institutionCode: CNC; collectionCode: Insects; basisOfRecord: Pinned Specimen
- **Type status:** Paratype. **Occurrence:** occurrenceDetails: http://janzen.sas.upenn.edu; catalogNumber: DHJPAR0010026; recordedBy: D.H. Janzen, W. Hallwachs & Mariano Pereira; individualID: DHJPAR0010026; individualCount: 1; sex: male; lifeStage: adult; preparations: pinned; otherCatalogNumbers: ASTAV627-06, 06-SRNP-45198, BOLD:AAA5134; **Taxon:** scientificName: Hyphantrophaga danausophaga; phylum: Arthropoda; class: Insecta; order: Diptera; family: Tachinidae; genus: Hyphantrophaga; specificEpithet: danausophaga; scientificNameAuthorship: Fleming & Wood, 2018; **Location:** continent: Central America; country: Costa Rica; countryCode: CR; stateProvince: Guanacaste; county: Sector Cacao; locality: Area de Conservacion Guanacaste; verbatimLocality: Cuesta Caimito; verbatimElevation: 640; verbatimLatitude: 10.8908; verbatimLongitude: -85.4719; verbatimCoordinateSystem: Decimal; decimalLatitude: 10.8908; decimalLongitude: -85.4719; **Identification:** identifiedBy: AJ Fleming; dateIdentified: 2017; **Event:** samplingProtocol: Reared from the larva of the Nymphalidae, Danaus plexippus; verbatimEventDate: 13-Apr-2006; **Record Level:** language: en; institutionCode: CNC; collectionCode: Insects; basisOfRecord: Pinned Specimen
- **Type status:** Other material. **Occurrence:** occurrenceDetails: http://janzen.sas.upenn.edu; catalogNumber: DHJPAR0017161; recordedBy: D.H. Janzen, W. Hallwachs & Anabelle Cordoba; individualID: DHJPAR0017161; individualCount: 1; sex: female; lifeStage: adult; preparations: pinned; otherCatalogNumbers: ASTAP599-07, 07-SRNP-760, BOLD:AAA5134; **Taxon:** scientificName: Hyphantrophaga danausophaga; phylum: Arthropoda; class: Insecta; order: Diptera; family: Tachinidae; genus: Hyphantrophaga; specificEpithet: danausophaga; scientificNameAuthorship: Fleming & Wood, 2018; **Location:** continent: Central America; country: Costa Rica; countryCode: CR; stateProvince: Alajuela; county: Sector San Cristobal; locality: Area de Conservacion Guanacaste; verbatimLocality: Rio Areno; verbatimElevation: 460; verbatimLatitude: 10.9141; verbatimLongitude: -85.3817; verbatimCoordinateSystem: Decimal; decimalLatitude: 10.9141; decimalLongitude: -85.3817; **Identification:** identifiedBy: AJ Fleming; dateIdentified: 2017; **Event:** samplingProtocol: Reared from the larva of the Nymphalidae, Danaus plexippus; verbatimEventDate: 16-Mar-2007; **Record Level:** language: en; institutionCode: CNC; collectionCode: Insects; basisOfRecord: Pinned Specimen
- **Type status:** Other material. **Occurrence:** occurrenceDetails: http://janzen.sas.upenn.edu; catalogNumber: DHJPAR0019692; recordedBy: D.H. Janzen, W. Hallwachs & Anabelle Cordoba; individualID: DHJPAR0019692; individualCount: 1; sex: female; lifeStage: adult; preparations: pinned; otherCatalogNumbers: ASTAB240-07, 07-SRNP-1366, BOLD:AAA5134; **Taxon:** scientificName: Hyphantrophaga danausophaga; phylum: Arthropoda; class: Insecta; order: Diptera; family: Tachinidae; genus: Hyphantrophaga; specificEpithet: danausophaga; scientificNameAuthorship: Fleming & Wood, 2018; **Location:** continent: Central America; country: Costa Rica; countryCode: CR; stateProvince: Alajuela; county: Sector San Cristobal; locality: Area de Conservacion Guanacaste; verbatimLocality: Sendero Vivero; verbatimElevation: 730; verbatimLatitude: 10.8674; verbatimLongitude: -85.3874; verbatimCoordinateSystem: Decimal; decimalLatitude: 10.8674; decimalLongitude: -85.3874; **Identification:** identifiedBy: AJ Fleming; dateIdentified: 2017; **Event:** samplingProtocol: Reared from the larva of the Nymphalidae, Danaus plexippus; verbatimEventDate: 18-Apr-2007; **Record Level:** language: en; institutionCode: CNC; collectionCode: Insects; basisOfRecord: Pinned Specimen
- **Type status:** Other material. **Occurrence:** occurrenceDetails: http://janzen.sas.upenn.edu; catalogNumber: DHJPAR0019735; recordedBy: D.H. Janzen, W. Hallwachs & Calixto Moraga; individualID: DHJPAR0019735; individualCount: 1; sex: female; lifeStage: adult; preparations: pinned; otherCatalogNumbers: ASTAB283-07, 07-SRNP-31425,; **Taxon:** scientificName: Hyphantrophaga danausophaga; phylum: Arthropoda; class: Insecta; order: Diptera; family: Tachinidae; genus: Hyphantrophaga; specificEpithet: danausophaga; scientificNameAuthorship: Fleming & Wood, 2018; **Location:** continent: Central America; country: Costa Rica; countryCode: CR; stateProvince: Guanacaste; county: Sector Pitilla; locality: Area de Conservacion Guanacaste; verbatimLocality: Sendero Carica; verbatimElevation: 660; verbatimLatitude: 10.9928; verbatimLongitude: -85.4294; verbatimCoordinateSystem: Decimal; decimalLatitude: 10.9928; decimalLongitude: -85.4294; **Identification:** identifiedBy: AJ Fleming; dateIdentified: 2017; **Event:** samplingProtocol: Reared from the larva of the Nymphalidae, Danaus plexippus; verbatimEventDate: 01-Apr-2007; **Record Level:** language: en; institutionCode: CNC; collectionCode: Insects; basisOfRecord: Pinned Specimen
- **Type status:** Other material. **Occurrence:** occurrenceDetails: http://janzen.sas.upenn.edu; catalogNumber: DHJPAR0019736; recordedBy: D.H. Janzen, W. Hallwachs & Calixto Moraga; individualID: DHJPAR0019736; individualCount: 1; sex: female; lifeStage: adult; preparations: pinned; otherCatalogNumbers: ASTAB284-07, 07-SRNP-31560, BOLD:AAA5134; **Taxon:** scientificName: Hyphantrophaga danausophaga; phylum: Arthropoda; class: Insecta; order: Diptera; family: Tachinidae; genus: Hyphantrophaga; specificEpithet: danausophaga; scientificNameAuthorship: Fleming & Wood, 2018; **Location:** continent: Central America; country: Costa Rica; countryCode: CR; stateProvince: Guanacaste; county: Sector Pitilla; locality: Area de Conservacion Guanacaste; verbatimLocality: Sendero Carica; verbatimElevation: 660; verbatimLatitude: 10.9928; verbatimLongitude: -85.4294; verbatimCoordinateSystem: Decimal; decimalLatitude: 10.9928; decimalLongitude: -85.4294; **Identification:** identifiedBy: AJ Fleming; dateIdentified: 2017; **Event:** samplingProtocol: Reared from the larva of the Nymphalidae, Danaus plexippus; verbatimEventDate: 01-Apr-2007; **Record Level:** language: en; institutionCode: CNC; collectionCode: Insects; basisOfRecord: Pinned Specimen
- **Type status:** Other material. **Occurrence:** occurrenceDetails: http://janzen.sas.upenn.edu; catalogNumber: DHJPAR0019740; recordedBy: D.H. Janzen, W. Hallwachs & Calixto Moraga; individualID: DHJPAR0019740; individualCount: 1; sex: male; lifeStage: adult; preparations: pinned; otherCatalogNumbers: ASTAB288-07, 07-SRNP-31561, BOLD:AAA5134; **Taxon:** scientificName: Hyphantrophaga danausophaga; phylum: Arthropoda; class: Insecta; order: Diptera; family: Tachinidae; genus: Hyphantrophaga; specificEpithet: danausophaga; scientificNameAuthorship: Fleming & Wood, 2018; **Location:** continent: Central America; country: Costa Rica; countryCode: CR; stateProvince: Guanacaste; county: Sector Pitilla; locality: Area de Conservacion Guanacaste; verbatimLocality: Sendero Carica; verbatimElevation: 660; verbatimLatitude: 10.9928; verbatimLongitude: -85.4294; verbatimCoordinateSystem: Decimal; decimalLatitude: 10.9928; decimalLongitude: -85.4294; **Identification:** identifiedBy: AJ Fleming; dateIdentified: 2017; **Event:** samplingProtocol: Reared from the larva of the Nymphalidae, Danaus plexippus; verbatimEventDate: 31-Mar-2007; **Record Level:** language: en; institutionCode: CNC; collectionCode: Insects; basisOfRecord: Pinned Specimen
- **Type status:** Other material. **Occurrence:** occurrenceDetails: http://janzen.sas.upenn.edu; catalogNumber: DHJPAR0019784; recordedBy: D.H. Janzen, W. Hallwachs & Manuel Rios; individualID: DHJPAR0019784; individualCount: 1; sex: male; lifeStage: adult; preparations: pinned; otherCatalogNumbers: ASTAB332-07, 07-SRNP-31929, BOLD:AAA5134; **Taxon:** scientificName: Hyphantrophaga danausophaga; phylum: Arthropoda; class: Insecta; order: Diptera; family: Tachinidae; genus: Hyphantrophaga; specificEpithet: danausophaga; scientificNameAuthorship: Fleming & Wood, 2018; **Location:** continent: Central America; country: Costa Rica; countryCode: CR; stateProvince: Guanacaste; county: Sector Pitilla; locality: Area de Conservacion Guanacaste; verbatimLocality: Estacion Pitilla; verbatimElevation: 675; verbatimLatitude: 10.9893; verbatimLongitude: -85.4258; verbatimCoordinateSystem: Decimal; decimalLatitude: 10.9893; decimalLongitude: -85.4258; **Identification:** identifiedBy: AJ Fleming; dateIdentified: 2017; **Event:** samplingProtocol: Reared from the larva of the Nymphalidae, Danaus plexippus; verbatimEventDate: 29-Apr-2007; **Record Level:** language: en; institutionCode: CNC; collectionCode: Insects; basisOfRecord: Pinned Specimen
- **Type status:** Other material. **Occurrence:** occurrenceDetails: http://janzen.sas.upenn.edu; catalogNumber: DHJPAR0021826; recordedBy: D.H. Janzen, W. Hallwachs & Guillermo Pereira; individualID: DHJPAR0021826; individualCount: 1; lifeStage: adult; preparations: pinned; otherCatalogNumbers: ASTAT964-07, 07-SRNP-14384, BOLD:AAA5134; **Taxon:** scientificName: Hyphantrophaga danausophaga; phylum: Arthropoda; class: Insecta; order: Diptera; family: Tachinidae; genus: Hyphantrophaga; specificEpithet: danausophaga; scientificNameAuthorship: Fleming & Wood, 2018; **Location:** continent: Central America; country: Costa Rica; countryCode: CR; stateProvince: Guanacaste; county: Sector Horizontes; locality: Area de Conservacion Guanacaste; verbatimLocality: Quebrada San Pancho; verbatimElevation: 90; verbatimLatitude: 10.7477; verbatimLongitude: -85.5858; verbatimCoordinateSystem: Decimal; decimalLatitude: 10.7477; decimalLongitude: -85.5858; **Identification:** identifiedBy: AJ Fleming; dateIdentified: 2017; **Event:** samplingProtocol: Reared from the larva of the Nymphalidae, Danaus plexippus; verbatimEventDate: 17-Jul-2007; **Record Level:** language: en; institutionCode: CNC; collectionCode: Insects; basisOfRecord: Pinned Specimen
- **Type status:** Other material. **Occurrence:** occurrenceDetails: http://janzen.sas.upenn.edu; catalogNumber: DHJPAR0021829; recordedBy: D.H. Janzen, W. Hallwachs & Guillermo Pereira; individualID: DHJPAR0021829; individualCount: 1; sex: female; lifeStage: adult; preparations: pinned; otherCatalogNumbers: ASTAT967-07, 07-SRNP-14379, BOLD:AAA5134; **Taxon:** scientificName: Hyphantrophaga danausophaga; phylum: Arthropoda; class: Insecta; order: Diptera; family: Tachinidae; genus: Hyphantrophaga; specificEpithet: danausophaga; scientificNameAuthorship: Fleming & Wood, 2018; **Location:** continent: Central America; country: Costa Rica; countryCode: CR; stateProvince: Guanacaste; county: Sector Horizontes; locality: Area de Conservacion Guanacaste; verbatimLocality: Quebrada San Pancho; verbatimElevation: 90; verbatimLatitude: 10.7477; verbatimLongitude: -85.5858; verbatimCoordinateSystem: Decimal; decimalLatitude: 10.7477; decimalLongitude: -85.5858; **Identification:** identifiedBy: AJ Fleming; dateIdentified: 2017; **Event:** samplingProtocol: Reared from the larva of the Nymphalidae, Danaus plexippus; verbatimEventDate: 17-Jul-2007; **Record Level:** language: en; institutionCode: CNC; collectionCode: Insects; basisOfRecord: Pinned Specimen
- **Type status:** Other material. **Occurrence:** occurrenceDetails: http://janzen.sas.upenn.edu; catalogNumber: DHJPAR0030196; recordedBy: D.H. Janzen, W. Hallwachs & Lucia Rios; individualID: DHJPAR0030196; individualCount: 1; sex: female; lifeStage: adult; preparations: pinned; otherCatalogNumbers: ASHYB940-09, 09-SRNP-20077, BOLD:AAA5134; **Taxon:** scientificName: Hyphantrophaga danausophaga; phylum: Arthropoda; class: Insecta; order: Diptera; family: Tachinidae; genus: Hyphantrophaga; specificEpithet: danausophaga; scientificNameAuthorship: Fleming & Wood, 2018; **Location:** continent: Central America; country: Costa Rica; countryCode: CR; stateProvince: Guanacaste; county: Sector Del Oro; locality: Area de Conservacion Guanacaste; verbatimLocality: Jose Leon; verbatimElevation: 400; verbatimLatitude: 11.033; verbatimLongitude: -85.429; verbatimCoordinateSystem: Decimal; decimalLatitude: 11.033; decimalLongitude: -85.429; **Identification:** identifiedBy: AJ Fleming; dateIdentified: 2017; **Event:** samplingProtocol: Reared from the larva of the Nymphalidae, Danaus plexippus; verbatimEventDate: 05-Feb-2009; **Record Level:** language: en; institutionCode: CNC; collectionCode: Insects; basisOfRecord: Pinned Specimen
- **Type status:** Other material. **Occurrence:** occurrenceDetails: http://janzen.sas.upenn.edu; catalogNumber: DHJPAR0030197; recordedBy: D.H. Janzen, W. Hallwachs & Lucia Rios; individualID: DHJPAR0030197; individualCount: 1; sex: female; lifeStage: adult; preparations: pinned; otherCatalogNumbers: ASHYB941-09, 09-SRNP-20033, BOLD:AAA5134; **Taxon:** scientificName: Hyphantrophaga danausophaga; phylum: Arthropoda; class: Insecta; order: Diptera; family: Tachinidae; genus: Hyphantrophaga; specificEpithet: danausophaga; scientificNameAuthorship: Fleming & Wood, 2018; **Location:** continent: Central America; country: Costa Rica; countryCode: CR; stateProvince: Guanacaste; county: Sector Del Oro; locality: Area de Conservacion Guanacaste; verbatimLocality: Jose Leon; verbatimElevation: 400; verbatimLatitude: 11.033; verbatimLongitude: -85.429; verbatimCoordinateSystem: Decimal; decimalLatitude: 11.033; decimalLongitude: -85.429; **Identification:** identifiedBy: AJ Fleming; dateIdentified: 2017; **Event:** samplingProtocol: Reared from the larva of the Nymphalidae, Danaus plexippus; verbatimEventDate: 02-Feb-2009; **Record Level:** language: en; institutionCode: CNC; collectionCode: Insects; basisOfRecord: Pinned Specimen
- **Type status:** Other material. **Occurrence:** occurrenceDetails: http://janzen.sas.upenn.edu; catalogNumber: DHJPAR0034366; recordedBy: D.H. Janzen, W. Hallwachs & Dunia Garcia; individualID: DHJPAR0034366; individualCount: 1; sex: female; lifeStage: adult; preparations: pinned; otherCatalogNumbers: ASHYC1018-09, 09-SRNP-35278, BOLD:AAA5134; **Taxon:** scientificName: Hyphantrophaga danausophaga; phylum: Arthropoda; class: Insecta; order: Diptera; family: Tachinidae; genus: Hyphantrophaga; specificEpithet: danausophaga; scientificNameAuthorship: Fleming & Wood, 2018; **Location:** continent: Central America; country: Costa Rica; countryCode: CR; stateProvince: Guanacaste; county: Sector Cacao; locality: Area de Conservacion Guanacaste; verbatimLocality: Estacion Cacao; verbatimElevation: 1150; verbatimLatitude: 10.9269; verbatimLongitude: -85.4682; verbatimCoordinateSystem: Decimal; decimalLatitude: 10.9269; decimalLongitude: -85.4682; **Identification:** identifiedBy: AJ Fleming; dateIdentified: 2017; **Event:** samplingProtocol: Reared from the larva of the Nymphalidae, Danaus plexippus; verbatimEventDate: 11-May-2009; **Record Level:** language: en; institutionCode: CNC; collectionCode: Insects; basisOfRecord: Pinned Specimen
- **Type status:** Other material. **Occurrence:** occurrenceDetails: http://janzen.sas.upenn.edu; catalogNumber: DHJPAR0034367; recordedBy: D.H. Janzen, W. Hallwachs & Manuel Pereira; individualID: DHJPAR0034367; individualCount: 1; sex: female; lifeStage: adult; preparations: pinned; otherCatalogNumbers: ASHYC1019-09, 09-SRNP-35356, BOLD:AAA5134; **Taxon:** scientificName: Hyphantrophaga danausophaga; phylum: Arthropoda; class: Insecta; order: Diptera; family: Tachinidae; genus: Hyphantrophaga; specificEpithet: danausophaga; scientificNameAuthorship: Fleming & Wood, 2018; **Location:** continent: Central America; country: Costa Rica; countryCode: CR; stateProvince: Guanacaste; county: Sector Cacao; locality: Area de Conservacion Guanacaste; verbatimLocality: Estacion Cacao; verbatimElevation: 1150; verbatimLatitude: 10.9269; verbatimLongitude: -85.4682; verbatimCoordinateSystem: Decimal; decimalLatitude: 10.9269; decimalLongitude: -85.4682; **Identification:** identifiedBy: AJ Fleming; dateIdentified: 2017; **Event:** samplingProtocol: Reared from the larva of the Nymphalidae, Danaus plexippus; verbatimEventDate: 22-May-2009; **Record Level:** language: en; institutionCode: CNC; collectionCode: Insects; basisOfRecord: Pinned Specimen
- **Type status:** Other material. **Occurrence:** occurrenceDetails: http://janzen.sas.upenn.edu; catalogNumber: DHJPAR0034369; recordedBy: D.H. Janzen, W. Hallwachs & Harry Ramirez; individualID: DHJPAR0034369; individualCount: 1; sex: male; lifeStage: adult; preparations: pinned; otherCatalogNumbers: ASHYC1021-09, 09-SRNP-35265, BOLD:AAA5134; **Taxon:** scientificName: Hyphantrophaga danausophaga; phylum: Arthropoda; class: Insecta; order: Diptera; family: Tachinidae; genus: Hyphantrophaga; specificEpithet: danausophaga; scientificNameAuthorship: Fleming & Wood, 2018; **Location:** continent: Central America; country: Costa Rica; countryCode: CR; stateProvince: Guanacaste; county: Sector Cacao; locality: Area de Conservacion Guanacaste; verbatimLocality: Estacion Cacao; verbatimElevation: 1150; verbatimLatitude: 10.9269; verbatimLongitude: -85.4682; verbatimCoordinateSystem: Decimal; decimalLatitude: 10.9269; decimalLongitude: -85.4682; **Identification:** identifiedBy: AJ Fleming; dateIdentified: 2017; **Event:** samplingProtocol: Reared from the larva of the Nymphalidae, Danaus plexippus; verbatimEventDate: 10-May-2009; **Record Level:** language: en; institutionCode: CNC; collectionCode: Insects; basisOfRecord: Pinned Specimen
- **Type status:** Other material. **Occurrence:** occurrenceDetails: http://janzen.sas.upenn.edu; catalogNumber: DHJPAR0034370; recordedBy: D.H. Janzen, W. Hallwachs & Manuel Pereira; individualID: DHJPAR0034370; individualCount: 1; sex: male; lifeStage: adult; preparations: pinned; otherCatalogNumbers: ASHYC1022-09, 09-SRNP-35358, BOLD:AAA5134; **Taxon:** scientificName: Hyphantrophaga danausophaga; phylum: Arthropoda; class: Insecta; order: Diptera; family: Tachinidae; genus: Hyphantrophaga; specificEpithet: danausophaga; scientificNameAuthorship: Fleming & Wood, 2018; **Location:** continent: Central America; country: Costa Rica; countryCode: CR; stateProvince: Guanacaste; county: Sector Cacao; locality: Area de Conservacion Guanacaste; verbatimLocality: Estacion Cacao; verbatimElevation: 1150; verbatimLatitude: 10.9269; verbatimLongitude: -85.4682; verbatimCoordinateSystem: Decimal; decimalLatitude: 10.9269; decimalLongitude: -85.4682; **Identification:** identifiedBy: AJ Fleming; dateIdentified: 2017; **Event:** samplingProtocol: Reared from the larva of the Nymphalidae, Danaus plexippus; verbatimEventDate: 18-May-2009; **Record Level:** language: en; institutionCode: CNC; collectionCode: Insects; basisOfRecord: Pinned Specimen
- **Type status:** Other material. **Occurrence:** occurrenceDetails: http://janzen.sas.upenn.edu; catalogNumber: DHJPAR0034371; recordedBy: D.H. Janzen, W. Hallwachs & Manuel Pereira; individualID: DHJPAR0034371; individualCount: 1; sex: female; lifeStage: adult; preparations: pinned; otherCatalogNumbers: ASHYC1023-09, 09-SRNP-35342, BOLD:AAA5134; **Taxon:** scientificName: Hyphantrophaga danausophaga; phylum: Arthropoda; class: Insecta; order: Diptera; family: Tachinidae; genus: Hyphantrophaga; specificEpithet: danausophaga; scientificNameAuthorship: Fleming & Wood, 2018; **Location:** continent: Central America; country: Costa Rica; countryCode: CR; stateProvince: Guanacaste; county: Sector Cacao; locality: Area de Conservacion Guanacaste; verbatimLocality: Estacion Cacao; verbatimElevation: 1150; verbatimLatitude: 10.9269; verbatimLongitude: -85.4682; verbatimCoordinateSystem: Decimal; decimalLatitude: 10.9269; decimalLongitude: -85.4682; **Identification:** identifiedBy: AJ Fleming; dateIdentified: 2017; **Event:** samplingProtocol: Reared from the larva of the Nymphalidae, Danaus plexippus; verbatimEventDate: 20-May-2009; **Record Level:** language: en; institutionCode: CNC; collectionCode: Insects; basisOfRecord: Pinned Specimen
- **Type status:** Other material. **Occurrence:** occurrenceDetails: http://janzen.sas.upenn.edu; catalogNumber: DHJPAR0034375; recordedBy: D.H. Janzen, W. Hallwachs & Manuel Pereira; individualID: DHJPAR0034375; individualCount: 1; sex: female; lifeStage: adult; preparations: pinned; otherCatalogNumbers: ASHYC1027-09, 09-SRNP-35341, BOLD:AAA5134; **Taxon:** scientificName: Hyphantrophaga danausophaga; phylum: Arthropoda; class: Insecta; order: Diptera; family: Tachinidae; genus: Hyphantrophaga; specificEpithet: danausophaga; scientificNameAuthorship: Fleming & Wood, 2018; **Location:** continent: Central America; country: Costa Rica; countryCode: CR; stateProvince: Guanacaste; county: Sector Cacao; locality: Area de Conservacion Guanacaste; verbatimLocality: Estacion Cacao; verbatimElevation: 1150; verbatimLatitude: 10.9269; verbatimLongitude: -85.4682; verbatimCoordinateSystem: Decimal; decimalLatitude: 10.9269; decimalLongitude: -85.4682; **Identification:** identifiedBy: AJ Fleming; dateIdentified: 2017; **Event:** samplingProtocol: Reared from the larva of the Nymphalidae, Danaus plexippus; verbatimEventDate: 05-May-2009; **Record Level:** language: en; institutionCode: CNC; collectionCode: Insects; basisOfRecord: Pinned Specimen
- **Type status:** Other material. **Occurrence:** occurrenceDetails: http://janzen.sas.upenn.edu; catalogNumber: DHJPAR0034377; recordedBy: D.H. Janzen, W. Hallwachs & Manuel Pereira; individualID: DHJPAR0034377; individualCount: 1; sex: female; lifeStage: adult; preparations: pinned; otherCatalogNumbers: ASHYC1029-09, 09-SRNP-35356, BOLD:AAA5134; **Taxon:** scientificName: Hyphantrophaga danausophaga; phylum: Arthropoda; class: Insecta; order: Diptera; family: Tachinidae; genus: Hyphantrophaga; specificEpithet: danausophaga; scientificNameAuthorship: Fleming & Wood, 2018; **Location:** continent: Central America; country: Costa Rica; countryCode: CR; stateProvince: Guanacaste; county: Sector Cacao; locality: Area de Conservacion Guanacaste; verbatimLocality: Estacion Cacao; verbatimElevation: 1150; verbatimLatitude: 10.9269; verbatimLongitude: -85.4682; verbatimCoordinateSystem: Decimal; decimalLatitude: 10.9269; decimalLongitude: -85.4682; **Identification:** identifiedBy: AJ Fleming; dateIdentified: 2017; **Event:** samplingProtocol: Reared from the larva of the Nymphalidae, Danaus plexippus; verbatimEventDate: 22-May-2009; **Record Level:** language: en; institutionCode: CNC; collectionCode: Insects; basisOfRecord: Pinned Specimen
- **Type status:** Other material. **Occurrence:** occurrenceDetails: http://janzen.sas.upenn.edu; catalogNumber: DHJPAR0034379; recordedBy: D.H. Janzen, W. Hallwachs & Manuel Pereira; individualID: DHJPAR0034379; individualCount: 1; sex: female; lifeStage: adult; preparations: pinned; otherCatalogNumbers: ASHYC1031-09, 09-SRNP-35357, BOLD:AAA5134; **Taxon:** scientificName: Hyphantrophaga danausophaga; phylum: Arthropoda; class: Insecta; order: Diptera; family: Tachinidae; genus: Hyphantrophaga; specificEpithet: danausophaga; scientificNameAuthorship: Fleming & Wood, 2018; **Location:** continent: Central America; country: Costa Rica; countryCode: CR; stateProvince: Guanacaste; county: Sector Cacao; locality: Area de Conservacion Guanacaste; verbatimLocality: Estacion Cacao; verbatimElevation: 1150; verbatimLatitude: 10.9269; verbatimLongitude: -85.4682; verbatimCoordinateSystem: Decimal; decimalLatitude: 10.9269; decimalLongitude: -85.4682; **Identification:** identifiedBy: AJ Fleming; dateIdentified: 2017; **Event:** samplingProtocol: Reared from the larva of the Nymphalidae, Danaus plexippus; verbatimEventDate: 22-May-2009; **Record Level:** language: en; institutionCode: CNC; collectionCode: Insects; basisOfRecord: Pinned Specimen
- **Type status:** Paratype. **Occurrence:** occurrenceDetails: http://janzen.sas.upenn.edu; catalogNumber: DHJPAR0042587; recordedBy: D.H. Janzen, W. Hallwachs & Harry Ramirez; individualID: DHJPAR0042587; individualCount: 1; lifeStage: adult; preparations: pinned; otherCatalogNumbers: ASHYH345-11, 11-SRNP-35111, BOLD:AAA5134; **Taxon:** scientificName: Hyphantrophaga danausophaga; phylum: Arthropoda; class: Insecta; order: Diptera; family: Tachinidae; genus: Hyphantrophaga; specificEpithet: danausophaga; scientificNameAuthorship: Fleming & Wood, 2018; **Location:** continent: Central America; country: Costa Rica; countryCode: CR; stateProvince: Guanacaste; county: Sector Cacao; locality: Area de Conservacion Guanacaste; verbatimLocality: Estacion Cacao; verbatimElevation: 1150; verbatimLatitude: 10.9269; verbatimLongitude: -85.4682; verbatimCoordinateSystem: Decimal; decimalLatitude: 10.9269; decimalLongitude: -85.4682; **Identification:** identifiedBy: AJ Fleming; dateIdentified: 2017; **Event:** samplingProtocol: Reared from the larva of the Nymphalidae, Danaus gilippus; verbatimEventDate: 29-Mar-2011; **Record Level:** language: en; institutionCode: CNC; collectionCode: Insects; basisOfRecord: Pinned Specimen
- **Type status:** Other material. **Occurrence:** occurrenceDetails: http://janzen.sas.upenn.edu; catalogNumber: DHJPAR0045680; recordedBy: D.H. Janzen, W. Hallwachs & Christian Herrera; individualID: DHJPAR0045680; individualCount: 1; sex: female; lifeStage: adult; preparations: pinned; otherCatalogNumbers: ACGAZ869-11, 11-SRNP-3220, BOLD:AAA5134; **Taxon:** scientificName: Hyphantrophaga danausophaga; phylum: Arthropoda; class: Insecta; order: Diptera; family: Tachinidae; genus: Hyphantrophaga; specificEpithet: danausophaga; scientificNameAuthorship: Fleming & Wood, 2018; **Location:** continent: Central America; country: Costa Rica; countryCode: CR; stateProvince: Alajuela; county: Sector San Cristobal; locality: Area de Conservacion Guanacaste; verbatimLocality: Finca San Gabriel; verbatimElevation: 645; verbatimLatitude: 10.8777; verbatimLongitude: -85.3934; verbatimCoordinateSystem: Decimal; decimalLatitude: 10.8777; decimalLongitude: -85.3934; **Identification:** identifiedBy: AJ Fleming; dateIdentified: 2017; **Event:** samplingProtocol: Reared from the larva of the Nymphalidae, Danaus plexippus; verbatimEventDate: 06-Sep-2011; **Record Level:** language: en; institutionCode: CNC; collectionCode: Insects; basisOfRecord: Pinned Specimen
- **Type status:** Other material. **Occurrence:** occurrenceDetails: http://janzen.sas.upenn.edu; catalogNumber: DHJPAR0046595; recordedBy: D.H. Janzen, W. Hallwachs & Manuel Pereira; individualID: DHJPAR0046595; individualCount: 1; sex: female; lifeStage: adult; preparations: pinned; otherCatalogNumbers: ACGBA768-12, 11-SRNP-35602, BOLD:AAA5134; **Taxon:** scientificName: Hyphantrophaga danausophaga; phylum: Arthropoda; class: Insecta; order: Diptera; family: Tachinidae; genus: Hyphantrophaga; specificEpithet: danausophaga; scientificNameAuthorship: Fleming & Wood, 2018; **Location:** continent: Central America; country: Costa Rica; countryCode: CR; stateProvince: Guanacaste; county: Sector Cacao; locality: Area de Conservacion Guanacaste; verbatimLocality: Estacion Cacao; verbatimElevation: 1150; verbatimLatitude: 10.9269; verbatimLongitude: -85.4682; verbatimCoordinateSystem: Decimal; decimalLatitude: 10.9269; decimalLongitude: -85.4682; **Identification:** identifiedBy: AJ Fleming; dateIdentified: 2017; **Event:** samplingProtocol: Reared from the larva of the Nymphalidae, Danaus gilippus; verbatimEventDate: 14-Sep-2011; **Record Level:** language: en; institutionCode: CNC; collectionCode: Insects; basisOfRecord: Pinned Specimen
- **Type status:** Other material. **Occurrence:** occurrenceDetails: http://janzen.sas.upenn.edu; catalogNumber: DHJPAR0046596; recordedBy: D.H. Janzen, W. Hallwachs & Manuel Pereira; individualID: DHJPAR0046596; individualCount: 1; sex: female; lifeStage: adult; preparations: pinned; otherCatalogNumbers: ACGBA769-12, 11-SRNP-35603, BOLD:AAA5134; **Taxon:** scientificName: Hyphantrophaga danausophaga; phylum: Arthropoda; class: Insecta; order: Diptera; family: Tachinidae; genus: Hyphantrophaga; specificEpithet: danausophaga; scientificNameAuthorship: Fleming & Wood, 2018; **Location:** continent: Central America; country: Costa Rica; countryCode: CR; stateProvince: Guanacaste; county: Sector Cacao; locality: Area de Conservacion Guanacaste; verbatimLocality: Estacion Cacao; verbatimElevation: 1150; verbatimLatitude: 10.9269; verbatimLongitude: -85.4682; verbatimCoordinateSystem: Decimal; decimalLatitude: 10.9269; decimalLongitude: -85.4682; **Identification:** identifiedBy: AJ Fleming; dateIdentified: 2017; **Event:** samplingProtocol: Reared from the larva of the Nymphalidae, Danaus gilippus; verbatimEventDate: 16-Sep-2011; **Record Level:** language: en; institutionCode: CNC; collectionCode: Insects; basisOfRecord: Pinned Specimen
- **Type status:** Other material. **Occurrence:** occurrenceDetails: http://janzen.sas.upenn.edu; catalogNumber: DHJPAR0046597; recordedBy: D.H. Janzen, W. Hallwachs & Manuel Pereira; individualID: DHJPAR0046597; individualCount: 1; sex: female; lifeStage: adult; preparations: pinned; otherCatalogNumbers: ACGBA770-12, 11-SRNP-35604, BOLD:AAA5134; **Taxon:** scientificName: Hyphantrophaga danausophaga; phylum: Arthropoda; class: Insecta; order: Diptera; family: Tachinidae; genus: Hyphantrophaga; specificEpithet: danausophaga; scientificNameAuthorship: Fleming & Wood, 2018; **Location:** continent: Central America; country: Costa Rica; countryCode: CR; stateProvince: Guanacaste; county: Sector Cacao; locality: Area de Conservacion Guanacaste; verbatimLocality: Estacion Cacao; verbatimElevation: 1150; verbatimLatitude: 10.9269; verbatimLongitude: -85.4682; verbatimCoordinateSystem: Decimal; decimalLatitude: 10.9269; decimalLongitude: -85.4682; **Identification:** identifiedBy: AJ Fleming; dateIdentified: 2017; **Event:** samplingProtocol: Reared from the larva of the Nymphalidae, Danaus gilippus; verbatimEventDate: 14-Sep-2011; **Record Level:** language: en; institutionCode: CNC; collectionCode: Insects; basisOfRecord: Pinned Specimen
- **Type status:** Other material. **Occurrence:** occurrenceDetails: http://janzen.sas.upenn.edu; catalogNumber: DHJPAR0046598; recordedBy: D.H. Janzen, W. Hallwachs & Manuel Pereira; individualID: DHJPAR0046598; individualCount: 1; sex: female; lifeStage: adult; preparations: pinned; otherCatalogNumbers: ACGBA771-12, 11-SRNP-35605, BOLD:AAA5134; **Taxon:** scientificName: Hyphantrophaga danausophaga; phylum: Arthropoda; class: Insecta; order: Diptera; family: Tachinidae; genus: Hyphantrophaga; specificEpithet: danausophaga; scientificNameAuthorship: Fleming & Wood, 2018; **Location:** continent: Central America; country: Costa Rica; countryCode: CR; stateProvince: Guanacaste; county: Sector Cacao; locality: Area de Conservacion Guanacaste; verbatimLocality: Estacion Cacao; verbatimElevation: 1150; verbatimLatitude: 10.9269; verbatimLongitude: -85.4682; verbatimCoordinateSystem: Decimal; decimalLatitude: 10.9269; decimalLongitude: -85.4682; **Identification:** identifiedBy: AJ Fleming; dateIdentified: 2017; **Event:** samplingProtocol: Reared from the larva of the Nymphalidae, Danaus gilippus; verbatimEventDate: 16-Sep-2011; **Record Level:** language: en; institutionCode: CNC; collectionCode: Insects; basisOfRecord: Pinned Specimen
- **Type status:** Other material. **Occurrence:** occurrenceDetails: http://janzen.sas.upenn.edu; catalogNumber: DHJPAR0050240; recordedBy: D.H. Janzen, W. Hallwachs & Cirilo Umaña; individualID: DHJPAR0050240; individualCount: 1; sex: male; lifeStage: adult; preparations: pinned; otherCatalogNumbers: ACGAZ1554-12, 12-SRNP-75567, BOLD:AAA5134; **Taxon:** scientificName: Hyphantrophaga danausophaga; phylum: Arthropoda; class: Insecta; order: Diptera; family: Tachinidae; genus: Hyphantrophaga; specificEpithet: danausophaga; scientificNameAuthorship: Fleming & Wood, 2018; **Location:** continent: Central America; country: Costa Rica; countryCode: CR; stateProvince: Alajuela; county: Sector Rincon Rain Forest; locality: Area de Conservacion Guanacaste; verbatimLocality: Finca Esmeralda; verbatimElevation: 123; verbatimLatitude: 10.9355; verbatimLongitude: -85.2531; verbatimCoordinateSystem: Decimal; decimalLatitude: 10.9355; decimalLongitude: -85.2531; **Identification:** identifiedBy: AJ Fleming; dateIdentified: 2017; **Event:** samplingProtocol: Reared from the larva of the Nymphalidae, Danaus plexippus; verbatimEventDate: 02-Apr-2012; **Record Level:** language: en; institutionCode: CNC; collectionCode: Insects; basisOfRecord: Pinned Specimen
- **Type status:** Other material. **Occurrence:** occurrenceDetails: http://janzen.sas.upenn.edu; catalogNumber: DHJPAR0050466; recordedBy: D.H. Janzen, W. Hallwachs & Cirilo Umaña; individualID: DHJPAR0050466; individualCount: 1; sex: female; lifeStage: adult; preparations: pinned; otherCatalogNumbers: ACGBA3058-13, 13-SRNP-75000, BOLD:AAA5134; **Taxon:** scientificName: Hyphantrophaga danausophaga; phylum: Arthropoda; class: Insecta; order: Diptera; family: Tachinidae; genus: Hyphantrophaga; specificEpithet: danausophaga; scientificNameAuthorship: Fleming & Wood, 2018; **Location:** continent: Central America; country: Costa Rica; countryCode: CR; stateProvince: Alajuela; county: Sector Rincon Rain Forest; locality: Area de Conservacion Guanacaste; verbatimLocality: Finca Esmeralda; verbatimElevation: 123; verbatimLatitude: 10.9355; verbatimLongitude: -85.2531; verbatimCoordinateSystem: Decimal; decimalLatitude: 10.9355; decimalLongitude: -85.2531; **Identification:** identifiedBy: AJ Fleming; dateIdentified: 2017; **Event:** samplingProtocol: Reared from the larva of the Nymphalidae, Danaus plexippus; verbatimEventDate: 26-Jan-2013; **Record Level:** language: en; institutionCode: CNC; collectionCode: Insects; basisOfRecord: Pinned Specimen
- **Type status:** Other material. **Occurrence:** occurrenceDetails: http://janzen.sas.upenn.edu; catalogNumber: DHJPAR0050476; recordedBy: D.H. Janzen, W. Hallwachs & Cirilo Umaña; individualID: DHJPAR0050476; individualCount: 1; sex: female; lifeStage: adult; preparations: pinned; otherCatalogNumbers: ACGBA3068-13, 12-SRNP-78158, BOLD:AAA5134; **Taxon:** scientificName: Hyphantrophaga danausophaga; phylum: Arthropoda; class: Insecta; order: Diptera; family: Tachinidae; genus: Hyphantrophaga; specificEpithet: danausophaga; scientificNameAuthorship: Fleming & Wood, 2018; **Location:** continent: Central America; country: Costa Rica; countryCode: CR; stateProvince: Alajuela; county: Sector Rincon Rain Forest; locality: Area de Conservacion Guanacaste; verbatimLocality: Finca Esmeralda; verbatimElevation: 123; verbatimLatitude: 10.9355; verbatimLongitude: -85.2531; verbatimCoordinateSystem: Decimal; decimalLatitude: 10.9355; decimalLongitude: -85.2531; **Identification:** identifiedBy: AJ Fleming; dateIdentified: 2017; **Event:** samplingProtocol: Reared from the larva of the Nymphalidae, Danaus gilippus; verbatimEventDate: 17-Jan-2013; **Record Level:** language: en; institutionCode: CNC; collectionCode: Insects; basisOfRecord: Pinned Specimen
- **Type status:** Other material. **Occurrence:** occurrenceDetails: http://janzen.sas.upenn.edu; catalogNumber: DHJPAR0050479; recordedBy: D.H. Janzen, W. Hallwachs & Cirilo Umaña; individualID: DHJPAR0050479; individualCount: 1; sex: female; lifeStage: adult; preparations: pinned; otherCatalogNumbers: ACGBA3071-13, 12-SRNP-78157, BOLD:AAA5134; **Taxon:** scientificName: Hyphantrophaga danausophaga; phylum: Arthropoda; class: Insecta; order: Diptera; family: Tachinidae; genus: Hyphantrophaga; specificEpithet: danausophaga; scientificNameAuthorship: Fleming & Wood, 2018; **Location:** continent: Central America; country: Costa Rica; countryCode: CR; stateProvince: Alajuela; county: Sector Rincon Rain Forest; locality: Area de Conservacion Guanacaste; verbatimLocality: Finca Esmeralda; verbatimElevation: 123; verbatimLatitude: 10.9355; verbatimLongitude: -85.2531; verbatimCoordinateSystem: Decimal; decimalLatitude: 10.9355; decimalLongitude: -85.2531; **Identification:** identifiedBy: AJ Fleming; dateIdentified: 2017; **Event:** samplingProtocol: Reared from the larva of the Nymphalidae, Danaus gilippus; verbatimEventDate: 18-Jan-2013; **Record Level:** language: en; institutionCode: CNC; collectionCode: Insects; basisOfRecord: Pinned Specimen
- **Type status:** Other material. **Occurrence:** occurrenceDetails: http://janzen.sas.upenn.edu; catalogNumber: DHJPAR0050634; recordedBy: D.H. Janzen, W. Hallwachs & Cirilo Umaña; individualID: DHJPAR0050634; individualCount: 1; sex: male; lifeStage: adult; preparations: pinned; otherCatalogNumbers: ACGBA3226-13, 12-SRNP-77661, BOLD:AAA5134; **Taxon:** scientificName: Hyphantrophaga danausophaga; phylum: Arthropoda; class: Insecta; order: Diptera; family: Tachinidae; genus: Hyphantrophaga; specificEpithet: danausophaga; scientificNameAuthorship: Fleming & Wood, 2018; **Location:** continent: Central America; country: Costa Rica; countryCode: CR; stateProvince: Alajuela; county: Sector Rincon Rain Forest; locality: Area de Conservacion Guanacaste; verbatimLocality: Finca Esmeralda; verbatimElevation: 123; verbatimLatitude: 10.9355; verbatimLongitude: -85.2531; verbatimCoordinateSystem: Decimal; decimalLatitude: 10.9355; decimalLongitude: -85.2531; **Identification:** identifiedBy: AJ Fleming; dateIdentified: 2017; **Event:** samplingProtocol: Reared from the larva of the Nymphalidae, Danaus gilippus; verbatimEventDate: 10-Dec-2012; **Record Level:** language: en; institutionCode: CNC; collectionCode: Insects; basisOfRecord: Pinned Specimen
- **Type status:** Other material. **Occurrence:** occurrenceDetails: http://janzen.sas.upenn.edu; catalogNumber: DHJPAR0052440; recordedBy: D.H. Janzen, W. Hallwachs & Lucia Rios; individualID: DHJPAR0052440; individualCount: 1; sex: male; lifeStage: adult; preparations: pinned; otherCatalogNumbers: ASHYM1794-13, 13-SRNP-21416, BOLD:AAA5134; **Taxon:** scientificName: Hyphantrophaga danausophaga; phylum: Arthropoda; class: Insecta; order: Diptera; family: Tachinidae; genus: Hyphantrophaga; specificEpithet: danausophaga; scientificNameAuthorship: Fleming & Wood, 2018; **Location:** continent: Central America; country: Costa Rica; countryCode: CR; stateProvince: Guanacaste; county: Sector El Hacha; locality: Area de Conservacion Guanacaste; verbatimLocality: Estacion Los Almendros; verbatimElevation: 290; verbatimLatitude: 11.0323; verbatimLongitude: -85.5278; verbatimCoordinateSystem: Decimal; decimalLatitude: 11.0323; decimalLongitude: -85.5278; **Identification:** identifiedBy: AJ Fleming; dateIdentified: 2017; **Event:** samplingProtocol: Reared from the larva of the Nymphalidae, Danaus plexippus; verbatimEventDate: 19-Aug-2013; **Record Level:** language: en; institutionCode: CNC; collectionCode: Insects; basisOfRecord: Pinned Specimen
- **Type status:** Other material. **Occurrence:** occurrenceDetails: http://janzen.sas.upenn.edu; catalogNumber: DHJPAR0052445; recordedBy: D.H. Janzen, W. Hallwachs & Lucia Rios; individualID: DHJPAR0052445; individualCount: 1; sex: female; lifeStage: adult; preparations: pinned; otherCatalogNumbers: ASHYM1799-13, 13-SRNP-21291, BOLD:AAA5134; **Taxon:** scientificName: Hyphantrophaga danausophaga; phylum: Arthropoda; class: Insecta; order: Diptera; family: Tachinidae; genus: Hyphantrophaga; specificEpithet: danausophaga; scientificNameAuthorship: Fleming & Wood, 2018; **Location:** continent: Central America; country: Costa Rica; countryCode: CR; stateProvince: Guanacaste; county: Sector El Hacha; locality: Area de Conservacion Guanacaste; verbatimLocality: Los Inocentes; verbatimElevation: 275; verbatimLatitude: 11.0324; verbatimLongitude: -85.4993; verbatimCoordinateSystem: Decimal; decimalLatitude: 11.0324; decimalLongitude: -85.4993; **Identification:** identifiedBy: AJ Fleming; dateIdentified: 2017; **Event:** samplingProtocol: Reared from the larva of the Nymphalidae, Danaus plexippus; verbatimEventDate: 16-Aug-2013; **Record Level:** language: en; institutionCode: CNC; collectionCode: Insects; basisOfRecord: Pinned Specimen
- **Type status:** Other material. **Occurrence:** occurrenceDetails: http://janzen.sas.upenn.edu; catalogNumber: DHJPAR0052447; recordedBy: D.H. Janzen, W. Hallwachs & Lucia Rios; individualID: DHJPAR0052447; individualCount: 1; sex: male; lifeStage: adult; preparations: pinned; otherCatalogNumbers: ASHYM1801-13, 13-SRNP-21325, BOLD:AAA5134; **Taxon:** scientificName: Hyphantrophaga danausophaga; phylum: Arthropoda; class: Insecta; order: Diptera; family: Tachinidae; genus: Hyphantrophaga; specificEpithet: danausophaga; scientificNameAuthorship: Fleming & Wood, 2018; **Location:** continent: Central America; country: Costa Rica; countryCode: CR; stateProvince: Guanacaste; county: Sector El Hacha; locality: Area de Conservacion Guanacaste; verbatimLocality: Los Inocentes; verbatimElevation: 275; verbatimLatitude: 11.0324; verbatimLongitude: -85.4993; verbatimCoordinateSystem: Decimal; decimalLatitude: 11.0324; decimalLongitude: -85.4993; **Identification:** identifiedBy: AJ Fleming; dateIdentified: 2017; **Event:** samplingProtocol: Reared from the larva of the Nymphalidae, Danaus plexippus; verbatimEventDate: 10-Aug-2013; **Record Level:** language: en; institutionCode: CNC; collectionCode: Insects; basisOfRecord: Pinned Specimen
- **Type status:** Other material. **Occurrence:** occurrenceDetails: http://janzen.sas.upenn.edu; catalogNumber: DHJPAR0052448; recordedBy: D.H. Janzen, W. Hallwachs & Lucia Rios; individualID: DHJPAR0052448; individualCount: 1; sex: female; lifeStage: adult; preparations: pinned; otherCatalogNumbers: ASHYM1802-13, 13-SRNP-21279, BOLD:AAA5134; **Taxon:** scientificName: Hyphantrophaga danausophaga; phylum: Arthropoda; class: Insecta; order: Diptera; family: Tachinidae; genus: Hyphantrophaga; specificEpithet: danausophaga; scientificNameAuthorship: Fleming & Wood, 2018; **Location:** continent: Central America; country: Costa Rica; countryCode: CR; stateProvince: Guanacaste; county: Sector El Hacha; locality: Area de Conservacion Guanacaste; verbatimLocality: Los Inocentes; verbatimElevation: 275; verbatimLatitude: 11.0324; verbatimLongitude: -85.4993; verbatimCoordinateSystem: Decimal; decimalLatitude: 11.0324; decimalLongitude: -85.4993; **Identification:** identifiedBy: AJ Fleming; dateIdentified: 2017; **Event:** samplingProtocol: Reared from the larva of the Nymphalidae, Danaus plexippus; verbatimEventDate: 08-Aug-2013; **Record Level:** language: en; institutionCode: CNC; collectionCode: Insects; basisOfRecord: Pinned Specimen
- **Type status:** Other material. **Occurrence:** occurrenceDetails: http://janzen.sas.upenn.edu; catalogNumber: DHJPAR0052451; recordedBy: D.H. Janzen, W. Hallwachs & Lucia Rios; individualID: DHJPAR0052451; individualCount: 1; sex: female; lifeStage: adult; preparations: pinned; otherCatalogNumbers: ASHYM1805-13, 13-SRNP-21288, BOLD:AAA5134; **Taxon:** scientificName: Hyphantrophaga danausophaga; phylum: Arthropoda; class: Insecta; order: Diptera; family: Tachinidae; genus: Hyphantrophaga; specificEpithet: danausophaga; scientificNameAuthorship: Fleming & Wood, 2018; **Location:** continent: Central America; country: Costa Rica; countryCode: CR; stateProvince: Guanacaste; county: Sector El Hacha; locality: Area de Conservacion Guanacaste; verbatimLocality: Los Inocentes; verbatimElevation: 275; verbatimLatitude: 11.0324; verbatimLongitude: -85.4993; verbatimCoordinateSystem: Decimal; decimalLatitude: 11.0324; decimalLongitude: -85.4993; **Identification:** identifiedBy: AJ Fleming; dateIdentified: 2017; **Event:** samplingProtocol: Reared from the larva of the Nymphalidae, Danaus plexippus; verbatimEventDate: 06-Aug-2013; **Record Level:** language: en; institutionCode: CNC; collectionCode: Insects; basisOfRecord: Pinned Specimen
- **Type status:** Other material. **Occurrence:** occurrenceDetails: http://janzen.sas.upenn.edu; catalogNumber: DHJPAR0052453; recordedBy: D.H. Janzen, W. Hallwachs & Lucia Rios; individualID: DHJPAR0052453; individualCount: 1; sex: female; lifeStage: adult; preparations: pinned; otherCatalogNumbers: ASHYM1807-13, 13-SRNP-21283, BOLD:AAA5134; **Taxon:** scientificName: Hyphantrophaga danausophaga; phylum: Arthropoda; class: Insecta; order: Diptera; family: Tachinidae; genus: Hyphantrophaga; specificEpithet: danausophaga; scientificNameAuthorship: Fleming & Wood, 2018; **Location:** continent: Central America; country: Costa Rica; countryCode: CR; stateProvince: Guanacaste; county: Sector El Hacha; locality: Area de Conservacion Guanacaste; verbatimLocality: Los Inocentes; verbatimElevation: 275; verbatimLatitude: 11.0324; verbatimLongitude: -85.4993; verbatimCoordinateSystem: Decimal; decimalLatitude: 11.0324; decimalLongitude: -85.4993; **Identification:** identifiedBy: AJ Fleming; dateIdentified: 2017; **Event:** samplingProtocol: Reared from the larva of the Nymphalidae, Danaus plexippus; verbatimEventDate: 12-Aug-2013; **Record Level:** language: en; institutionCode: CNC; collectionCode: Insects; basisOfRecord: Pinned Specimen
- **Type status:** Other material. **Occurrence:** occurrenceDetails: http://janzen.sas.upenn.edu; catalogNumber: DHJPAR0052483; recordedBy: D.H. Janzen, W. Hallwachs & Elieth Cantillano; individualID: DHJPAR0052483; individualCount: 1; sex: female; lifeStage: adult; preparations: pinned; otherCatalogNumbers: ASHYM1837-13, 13-SRNP-21247, BOLD:AAA5134; **Taxon:** scientificName: Hyphantrophaga danausophaga; phylum: Arthropoda; class: Insecta; order: Diptera; family: Tachinidae; genus: Hyphantrophaga; specificEpithet: danausophaga; scientificNameAuthorship: Fleming & Wood, 2018; **Location:** continent: Central America; country: Costa Rica; countryCode: CR; stateProvince: Guanacaste; county: Sector El Hacha; locality: Area de Conservacion Guanacaste; verbatimLocality: Los Inocentes; verbatimElevation: 275; verbatimLatitude: 11.0324; verbatimLongitude: -85.4993; verbatimCoordinateSystem: Decimal; decimalLatitude: 11.0324; decimalLongitude: -85.4993; **Identification:** identifiedBy: AJ Fleming; dateIdentified: 2017; **Event:** samplingProtocol: Reared from the larva of the Nymphalidae, Danaus plexippus; verbatimEventDate: 02-Aug-2013; **Record Level:** language: en; institutionCode: CNC; collectionCode: Insects; basisOfRecord: Pinned Specimen
- **Type status:** Other material. **Occurrence:** occurrenceDetails: http://janzen.sas.upenn.edu; catalogNumber: DHJPAR0053346; recordedBy: D.H. Janzen, W. Hallwachs & Lucia Rios; individualID: DHJPAR0053346; individualCount: 1; sex: female; lifeStage: adult; preparations: pinned; otherCatalogNumbers: ASHYM2700-13, 13-SRNP-21925, BOLD:AAA5134; **Taxon:** scientificName: Hyphantrophaga danausophaga; phylum: Arthropoda; class: Insecta; order: Diptera; family: Tachinidae; genus: Hyphantrophaga; specificEpithet: danausophaga; scientificNameAuthorship: Fleming & Wood, 2018; **Location:** continent: Central America; country: Costa Rica; countryCode: CR; stateProvince: Guanacaste; county: Sector El Hacha; locality: Area de Conservacion Guanacaste; verbatimLocality: Estacion Los Almendros; verbatimElevation: 290; verbatimLatitude: 11.0323; verbatimLongitude: -85.5278; verbatimCoordinateSystem: Decimal; decimalLatitude: 11.0323; decimalLongitude: -85.5278; **Identification:** identifiedBy: AJ Fleming; dateIdentified: 2017; **Event:** samplingProtocol: Reared from the larva of the Nymphalidae, Danaus plexippus; verbatimEventDate: 16-Sep-2013; **Record Level:** language: en; institutionCode: CNC; collectionCode: Insects; basisOfRecord: Pinned Specimen
- **Type status:** Other material. **Occurrence:** occurrenceDetails: http://janzen.sas.upenn.edu; catalogNumber: DHJPAR0053347; recordedBy: D.H. Janzen, W. Hallwachs & Lucia Rios; individualID: DHJPAR0053347; individualCount: 1; sex: male; lifeStage: adult; preparations: pinned; otherCatalogNumbers: ASHYM2701-13, 13-SRNP-21855, BOLD:AAA5134; **Taxon:** scientificName: Hyphantrophaga danausophaga; phylum: Arthropoda; class: Insecta; order: Diptera; family: Tachinidae; genus: Hyphantrophaga; specificEpithet: danausophaga; scientificNameAuthorship: Fleming & Wood, 2018; **Location:** continent: Central America; country: Costa Rica; countryCode: CR; stateProvince: Guanacaste; county: Sector El Hacha; locality: Area de Conservacion Guanacaste; verbatimLocality: Los Inocentes; verbatimElevation: 275; verbatimLatitude: 11.0324; verbatimLongitude: -85.4993; verbatimCoordinateSystem: Decimal; decimalLatitude: 11.0324; decimalLongitude: -85.4993; **Identification:** identifiedBy: AJ Fleming; dateIdentified: 2017; **Event:** samplingProtocol: Reared from the larva of the Nymphalidae, Danaus plexippus; verbatimEventDate: 11-Sep-2013; **Record Level:** language: en; institutionCode: CNC; collectionCode: Insects; basisOfRecord: Pinned Specimen
- **Type status:** Other material. **Occurrence:** occurrenceDetails: http://janzen.sas.upenn.edu; catalogNumber: DHJPAR0053348; recordedBy: D.H. Janzen, W. Hallwachs & Lucia Rios; individualID: DHJPAR0053348; individualCount: 1; sex: male; lifeStage: adult; preparations: pinned; otherCatalogNumbers: ASHYM2702-13, 13-SRNP-21867, BOLD:AAA5134; **Taxon:** scientificName: Hyphantrophaga danausophaga; phylum: Arthropoda; class: Insecta; order: Diptera; family: Tachinidae; genus: Hyphantrophaga; specificEpithet: danausophaga; scientificNameAuthorship: Fleming & Wood, 2018; **Location:** continent: Central America; country: Costa Rica; countryCode: CR; stateProvince: Guanacaste; county: Sector El Hacha; locality: Area de Conservacion Guanacaste; verbatimLocality: Los Inocentes; verbatimElevation: 275; verbatimLatitude: 11.0324; verbatimLongitude: -85.4993; verbatimCoordinateSystem: Decimal; decimalLatitude: 11.0324; decimalLongitude: -85.4993; **Identification:** identifiedBy: AJ Fleming; dateIdentified: 2017; **Event:** samplingProtocol: Reared from the larva of the Nymphalidae, Danaus plexippus; verbatimEventDate: 14-Sep-2013; **Record Level:** language: en; institutionCode: CNC; collectionCode: Insects; basisOfRecord: Pinned Specimen
- **Type status:** Other material. **Occurrence:** occurrenceDetails: http://janzen.sas.upenn.edu; catalogNumber: DHJPAR0053349; recordedBy: D.H. Janzen, W. Hallwachs & Lucia Rios; individualID: DHJPAR0053349; individualCount: 1; sex: male; lifeStage: adult; preparations: pinned; otherCatalogNumbers: ASHYM2703-13, 13-SRNP-22005, BOLD:AAA5134; **Taxon:** scientificName: Hyphantrophaga danausophaga; phylum: Arthropoda; class: Insecta; order: Diptera; family: Tachinidae; genus: Hyphantrophaga; specificEpithet: danausophaga; scientificNameAuthorship: Fleming & Wood, 2018; **Location:** continent: Central America; country: Costa Rica; countryCode: CR; stateProvince: Guanacaste; county: Sector El Hacha; locality: Area de Conservacion Guanacaste; verbatimLocality: Los Inocentes; verbatimElevation: 275; verbatimLatitude: 11.0324; verbatimLongitude: -85.4993; verbatimCoordinateSystem: Decimal; decimalLatitude: 11.0324; decimalLongitude: -85.4993; **Identification:** identifiedBy: AJ Fleming; dateIdentified: 2017; **Event:** samplingProtocol: Reared from the larva of the Nymphalidae, Danaus plexippus; verbatimEventDate: 17-Sep-2013; **Record Level:** language: en; institutionCode: CNC; collectionCode: Insects; basisOfRecord: Pinned Specimen
- **Type status:** Other material. **Occurrence:** occurrenceDetails: http://janzen.sas.upenn.edu; catalogNumber: DHJPAR0053350; recordedBy: D.H. Janzen, W. Hallwachs & Lucia Rios; individualID: DHJPAR0053350; individualCount: 1; sex: male; lifeStage: adult; preparations: pinned; otherCatalogNumbers: ASHYM2704-13, 13-SRNP-21873, BOLD:AAA5134; **Taxon:** scientificName: Hyphantrophaga danausophaga; phylum: Arthropoda; class: Insecta; order: Diptera; family: Tachinidae; genus: Hyphantrophaga; specificEpithet: danausophaga; scientificNameAuthorship: Fleming & Wood, 2018; **Location:** continent: Central America; country: Costa Rica; countryCode: CR; stateProvince: Guanacaste; county: Sector El Hacha; locality: Area de Conservacion Guanacaste; verbatimLocality: Los Inocentes; verbatimElevation: 275; verbatimLatitude: 11.0324; verbatimLongitude: -85.4993; verbatimCoordinateSystem: Decimal; decimalLatitude: 11.0324; decimalLongitude: -85.4993; **Identification:** identifiedBy: AJ Fleming; dateIdentified: 2017; **Event:** samplingProtocol: Reared from the larva of the Nymphalidae, Danaus plexippus; verbatimEventDate: 20-Sep-2013; **Record Level:** language: en; institutionCode: CNC; collectionCode: Insects; basisOfRecord: Pinned Specimen
- **Type status:** Other material. **Occurrence:** occurrenceDetails: http://janzen.sas.upenn.edu; catalogNumber: DHJPAR0053351; recordedBy: D.H. Janzen, W. Hallwachs & Lucia Rios; individualID: DHJPAR0053351; individualCount: 1; sex: male; lifeStage: adult; preparations: pinned; otherCatalogNumbers: ASHYM2705-13, 13-SRNP-21870, BOLD:AAA5134; **Taxon:** scientificName: Hyphantrophaga danausophaga; phylum: Arthropoda; class: Insecta; order: Diptera; family: Tachinidae; genus: Hyphantrophaga; specificEpithet: danausophaga; scientificNameAuthorship: Fleming & Wood, 2018; **Location:** continent: Central America; country: Costa Rica; countryCode: CR; stateProvince: Guanacaste; county: Sector El Hacha; locality: Area de Conservacion Guanacaste; verbatimLocality: Los Inocentes; verbatimElevation: 275; verbatimLatitude: 11.0324; verbatimLongitude: -85.4993; verbatimCoordinateSystem: Decimal; decimalLatitude: 11.0324; decimalLongitude: -85.4993; **Identification:** identifiedBy: AJ Fleming; dateIdentified: 2017; **Event:** samplingProtocol: Reared from the larva of the Nymphalidae, Danaus plexippus; verbatimEventDate: 14-Sep-2013; **Record Level:** language: en; institutionCode: CNC; collectionCode: Insects; basisOfRecord: Pinned Specimen
- **Type status:** Other material. **Occurrence:** occurrenceDetails: http://janzen.sas.upenn.edu; catalogNumber: DHJPAR0053352; recordedBy: D.H. Janzen, W. Hallwachs & Lucia Rios; individualID: DHJPAR0053352; individualCount: 1; sex: female; lifeStage: adult; preparations: pinned; otherCatalogNumbers: ASHYM2706-13, 13-SRNP-21869, BOLD:AAA5134; **Taxon:** scientificName: Hyphantrophaga danausophaga; phylum: Arthropoda; class: Insecta; order: Diptera; family: Tachinidae; genus: Hyphantrophaga; specificEpithet: danausophaga; scientificNameAuthorship: Fleming & Wood, 2018; **Location:** continent: Central America; country: Costa Rica; countryCode: CR; stateProvince: Guanacaste; county: Sector El Hacha; locality: Area de Conservacion Guanacaste; verbatimLocality: Los Inocentes; verbatimElevation: 275; verbatimLatitude: 11.0324; verbatimLongitude: -85.4993; verbatimCoordinateSystem: Decimal; decimalLatitude: 11.0324; decimalLongitude: -85.4993; **Identification:** identifiedBy: AJ Fleming; dateIdentified: 2017; **Event:** samplingProtocol: Reared from the larva of the Nymphalidae, Danaus plexippus; verbatimEventDate: 11-Sep-2013; **Record Level:** language: en; institutionCode: CNC; collectionCode: Insects; basisOfRecord: Pinned Specimen
- **Type status:** Other material. **Occurrence:** occurrenceDetails: http://janzen.sas.upenn.edu; catalogNumber: DHJPAR0053353; recordedBy: D.H. Janzen, W. Hallwachs & Lucia Rios; individualID: DHJPAR0053353; individualCount: 1; sex: male; lifeStage: adult; preparations: pinned; otherCatalogNumbers: ASHYM2707-13, 13-SRNP-21921, BOLD:AAA5134; **Taxon:** scientificName: Hyphantrophaga danausophaga; phylum: Arthropoda; class: Insecta; order: Diptera; family: Tachinidae; genus: Hyphantrophaga; specificEpithet: danausophaga; scientificNameAuthorship: Fleming & Wood, 2018; **Location:** continent: Central America; country: Costa Rica; countryCode: CR; stateProvince: Guanacaste; county: Sector El Hacha; locality: Area de Conservacion Guanacaste; verbatimLocality: Los Inocentes; verbatimElevation: 275; verbatimLatitude: 11.0324; verbatimLongitude: -85.4993; verbatimCoordinateSystem: Decimal; decimalLatitude: 11.0324; decimalLongitude: -85.4993; **Identification:** identifiedBy: AJ Fleming; dateIdentified: 2017; **Event:** samplingProtocol: Reared from the larva of the Nymphalidae, Danaus plexippus; verbatimEventDate: unavailable, 2013; **Record Level:** language: en; institutionCode: CNC; collectionCode: Insects; basisOfRecord: Pinned Specimen
- **Type status:** Other material. **Occurrence:** occurrenceDetails: http://janzen.sas.upenn.edu; catalogNumber: DHJPAR0053354; recordedBy: D.H. Janzen, W. Hallwachs & Lucia Rios; individualID: DHJPAR0053354; individualCount: 1; sex: male; lifeStage: adult; preparations: pinned; otherCatalogNumbers: ASHYM2708-13, 13-SRNP-21924, BOLD:AAA5134; **Taxon:** scientificName: Hyphantrophaga danausophaga; phylum: Arthropoda; class: Insecta; order: Diptera; family: Tachinidae; genus: Hyphantrophaga; specificEpithet: danausophaga; scientificNameAuthorship: Fleming & Wood, 2018; **Location:** continent: Central America; country: Costa Rica; countryCode: CR; stateProvince: Guanacaste; county: Sector El Hacha; locality: Area de Conservacion Guanacaste; verbatimLocality: Los Inocentes; verbatimElevation: 275; verbatimLatitude: 11.0324; verbatimLongitude: -85.4993; verbatimCoordinateSystem: Decimal; decimalLatitude: 11.0324; decimalLongitude: -85.4993; **Identification:** identifiedBy: AJ Fleming; dateIdentified: 2017; **Event:** samplingProtocol: Reared from the larva of the Nymphalidae, Danaus plexippus; verbatimEventDate: unavailable, 2013; **Record Level:** language: en; institutionCode: CNC; collectionCode: Insects; basisOfRecord: Pinned Specimen
- **Type status:** Other material. **Occurrence:** occurrenceDetails: http://janzen.sas.upenn.edu; catalogNumber: DHJPAR0053355; recordedBy: D.H. Janzen, W. Hallwachs & Lucia Rios; individualID: DHJPAR0053355; individualCount: 1; sex: female; lifeStage: adult; preparations: pinned; otherCatalogNumbers: ASHYM2709-13, 13-SRNP-21859, BOLD:AAA5134; **Taxon:** scientificName: Hyphantrophaga danausophaga; phylum: Arthropoda; class: Insecta; order: Diptera; family: Tachinidae; genus: Hyphantrophaga; specificEpithet: danausophaga; scientificNameAuthorship: Fleming & Wood, 2018; **Location:** continent: Central America; country: Costa Rica; countryCode: CR; stateProvince: Guanacaste; county: Sector El Hacha; locality: Area de Conservacion Guanacaste; verbatimLocality: Los Inocentes; verbatimElevation: 275; verbatimLatitude: 11.0324; verbatimLongitude: -85.4993; verbatimCoordinateSystem: Decimal; decimalLatitude: 11.0324; decimalLongitude: -85.4993; **Identification:** identifiedBy: AJ Fleming; dateIdentified: 2017; **Event:** samplingProtocol: Reared from the larva of the Nymphalidae, Danaus plexippus; verbatimEventDate: 12-Sep-2013; **Record Level:** language: en; institutionCode: CNC; collectionCode: Insects; basisOfRecord: Pinned Specimen
- **Type status:** Other material. **Occurrence:** occurrenceDetails: http://janzen.sas.upenn.edu; catalogNumber: DHJPAR0053356; recordedBy: D.H. Janzen, W. Hallwachs & Lucia Rios; individualID: DHJPAR0053356; individualCount: 1; sex: male; lifeStage: adult; preparations: pinned; otherCatalogNumbers: ASHYM2710-13, 13-SRNP-21854, BOLD:AAA5134; **Taxon:** scientificName: Hyphantrophaga danausophaga; phylum: Arthropoda; class: Insecta; order: Diptera; family: Tachinidae; genus: Hyphantrophaga; specificEpithet: danausophaga; scientificNameAuthorship: Fleming & Wood, 2018; **Location:** continent: Central America; country: Costa Rica; countryCode: CR; stateProvince: Guanacaste; county: Sector El Hacha; locality: Area de Conservacion Guanacaste; verbatimLocality: Los Inocentes; verbatimElevation: 275; verbatimLatitude: 11.0324; verbatimLongitude: -85.4993; verbatimCoordinateSystem: Decimal; decimalLatitude: 11.0324; decimalLongitude: -85.4993; **Identification:** identifiedBy: AJ Fleming; dateIdentified: 2017; **Event:** samplingProtocol: Reared from the larva of the Nymphalidae, Danaus plexippus; verbatimEventDate: 12-Sep-2013; **Record Level:** language: en; institutionCode: CNC; collectionCode: Insects; basisOfRecord: Pinned Specimen
- **Type status:** Other material. **Occurrence:** occurrenceDetails: http://janzen.sas.upenn.edu; catalogNumber: DHJPAR0053359; recordedBy: D.H. Janzen, W. Hallwachs & Lucia Rios; individualID: DHJPAR0053359; individualCount: 1; sex: female; lifeStage: adult; preparations: pinned; otherCatalogNumbers: ASHYM2713-13, 13-SRNP-21995, BOLD:AAA5134; **Taxon:** scientificName: Hyphantrophaga danausophaga; phylum: Arthropoda; class: Insecta; order: Diptera; family: Tachinidae; genus: Hyphantrophaga; specificEpithet: danausophaga; scientificNameAuthorship: Fleming & Wood, 2018; **Location:** continent: Central America; country: Costa Rica; countryCode: CR; stateProvince: Guanacaste; county: Sector El Hacha; locality: Area de Conservacion Guanacaste; verbatimLocality: Los Inocentes; verbatimElevation: 275; verbatimLatitude: 11.0324; verbatimLongitude: -85.4993; verbatimCoordinateSystem: Decimal; decimalLatitude: 11.0324; decimalLongitude: -85.4993; **Identification:** identifiedBy: AJ Fleming; dateIdentified: 2017; **Event:** samplingProtocol: Reared from the larva of the Nymphalidae, Danaus plexippus; verbatimEventDate: 14-Oct-2013; **Record Level:** language: en; institutionCode: CNC; collectionCode: Insects; basisOfRecord: Pinned Specimen
- **Type status:** Other material. **Occurrence:** occurrenceDetails: http://janzen.sas.upenn.edu; catalogNumber: DHJPAR0053360; recordedBy: D.H. Janzen, W. Hallwachs & Lucia Rios; individualID: DHJPAR0053360; individualCount: 1; sex: female; lifeStage: adult; preparations: pinned; otherCatalogNumbers: ASHYM2714-13, 13-SRNP-21853, BOLD:AAA5134; **Taxon:** scientificName: Hyphantrophaga danausophaga; phylum: Arthropoda; class: Insecta; order: Diptera; family: Tachinidae; genus: Hyphantrophaga; specificEpithet: danausophaga; scientificNameAuthorship: Fleming & Wood, 2018; **Location:** continent: Central America; country: Costa Rica; countryCode: CR; stateProvince: Guanacaste; county: Sector El Hacha; locality: Area de Conservacion Guanacaste; verbatimLocality: Los Inocentes; verbatimElevation: 275; verbatimLatitude: 11.0324; verbatimLongitude: -85.4993; verbatimCoordinateSystem: Decimal; decimalLatitude: 11.0324; decimalLongitude: -85.4993; **Identification:** identifiedBy: AJ Fleming; dateIdentified: 2017; **Event:** samplingProtocol: Reared from the larva of the Nymphalidae, Danaus plexippus; verbatimEventDate: 03-Sep-2013; **Record Level:** language: en; institutionCode: CNC; collectionCode: Insects; basisOfRecord: Pinned Specimen
- **Type status:** Other material. **Occurrence:** occurrenceDetails: http://janzen.sas.upenn.edu; catalogNumber: DHJPAR0053361; recordedBy: D.H. Janzen, W. Hallwachs & Lucia Rios; individualID: DHJPAR0053361; individualCount: 1; sex: female; lifeStage: adult; preparations: pinned; otherCatalogNumbers: ASHYM2715-13, 13-SRNP-21852, BOLD:AAA5134; **Taxon:** scientificName: Hyphantrophaga danausophaga; phylum: Arthropoda; class: Insecta; order: Diptera; family: Tachinidae; genus: Hyphantrophaga; specificEpithet: danausophaga; scientificNameAuthorship: Fleming & Wood, 2018; **Location:** continent: Central America; country: Costa Rica; countryCode: CR; stateProvince: Guanacaste; county: Sector El Hacha; locality: Area de Conservacion Guanacaste; verbatimLocality: Los Inocentes; verbatimElevation: 275; verbatimLatitude: 11.0324; verbatimLongitude: -85.4993; verbatimCoordinateSystem: Decimal; decimalLatitude: 11.0324; decimalLongitude: -85.4993; **Identification:** identifiedBy: AJ Fleming; dateIdentified: 2017; **Event:** samplingProtocol: Reared from the larva of the Nymphalidae, Danaus plexippus; verbatimEventDate: 13-Sep-2013; **Record Level:** language: en; institutionCode: CNC; collectionCode: Insects; basisOfRecord: Pinned Specimen
- **Type status:** Other material. **Occurrence:** occurrenceDetails: http://janzen.sas.upenn.edu; catalogNumber: DHJPAR0053363; recordedBy: D.H. Janzen, W. Hallwachs & Lucia Rios; individualID: DHJPAR0053363; individualCount: 1; sex: female; lifeStage: adult; preparations: pinned; otherCatalogNumbers: ASHYM2717-13, 13-SRNP-22002, BOLD:AAA5134; **Taxon:** scientificName: Hyphantrophaga danausophaga; phylum: Arthropoda; class: Insecta; order: Diptera; family: Tachinidae; genus: Hyphantrophaga; specificEpithet: danausophaga; scientificNameAuthorship: Fleming & Wood, 2018; **Location:** continent: Central America; country: Costa Rica; countryCode: CR; stateProvince: Guanacaste; county: Sector El Hacha; locality: Area de Conservacion Guanacaste; verbatimLocality: Los Inocentes; verbatimElevation: 275; verbatimLatitude: 11.0324; verbatimLongitude: -85.4993; verbatimCoordinateSystem: Decimal; decimalLatitude: 11.0324; decimalLongitude: -85.4993; **Identification:** identifiedBy: AJ Fleming; dateIdentified: 2017; **Event:** samplingProtocol: Reared from the larva of the Nymphalidae, Danaus plexippus; verbatimEventDate: 14-Oct-2013; **Record Level:** language: en; institutionCode: CNC; collectionCode: Insects; basisOfRecord: Pinned Specimen
- **Type status:** Other material. **Occurrence:** occurrenceDetails: http://janzen.sas.upenn.edu; catalogNumber: DHJPAR0053370; recordedBy: D.H. Janzen, W. Hallwachs & Elieth Cantillano; individualID: DHJPAR0053370; individualCount: 1; sex: male; lifeStage: adult; preparations: pinned; otherCatalogNumbers: ASHYM2724-13, 13-SRNP-21248, BOLD:AAA5134; **Taxon:** scientificName: Hyphantrophaga danausophaga; phylum: Arthropoda; class: Insecta; order: Diptera; family: Tachinidae; genus: Hyphantrophaga; specificEpithet: danausophaga; scientificNameAuthorship: Fleming & Wood, 2018; **Location:** continent: Central America; country: Costa Rica; countryCode: CR; stateProvince: Guanacaste; county: Sector El Hacha; locality: Area de Conservacion Guanacaste; verbatimLocality: Los Inocentes; verbatimElevation: 275; verbatimLatitude: 11.0324; verbatimLongitude: -85.4993; verbatimCoordinateSystem: Decimal; decimalLatitude: 11.0324; decimalLongitude: -85.4993; **Identification:** identifiedBy: AJ Fleming; dateIdentified: 2017; **Event:** samplingProtocol: Reared from the larva of the Nymphalidae, Danaus plexippus; verbatimEventDate: 10-Aug-2013; **Record Level:** language: en; institutionCode: CNC; collectionCode: Insects; basisOfRecord: Pinned Specimen
- **Type status:** Other material. **Occurrence:** occurrenceDetails: http://janzen.sas.upenn.edu; catalogNumber: DHJPAR0053371; recordedBy: D.H. Janzen, W. Hallwachs & Lucia Rios; individualID: DHJPAR0053371; individualCount: 1; sex: female; lifeStage: adult; preparations: pinned; otherCatalogNumbers: ASHYM2725-13, 13-SRNP-22004, BOLD:AAA5134; **Taxon:** scientificName: Hyphantrophaga danausophaga; phylum: Arthropoda; class: Insecta; order: Diptera; family: Tachinidae; genus: Hyphantrophaga; specificEpithet: danausophaga; scientificNameAuthorship: Fleming & Wood, 2018; **Location:** continent: Central America; country: Costa Rica; countryCode: CR; stateProvince: Guanacaste; county: Sector El Hacha; locality: Area de Conservacion Guanacaste; verbatimLocality: Los Inocentes; verbatimElevation: 275; verbatimLatitude: 11.0324; verbatimLongitude: -85.4993; verbatimCoordinateSystem: Decimal; decimalLatitude: 11.0324; decimalLongitude: -85.4993; **Identification:** identifiedBy: AJ Fleming; dateIdentified: 2017; **Event:** samplingProtocol: Reared from the larva of the Nymphalidae, Danaus plexippus; verbatimEventDate: 23-Sep-2013; **Record Level:** language: en; institutionCode: CNC; collectionCode: Insects; basisOfRecord: Pinned Specimen
- **Type status:** Other material. **Occurrence:** occurrenceDetails: http://janzen.sas.upenn.edu; catalogNumber: DHJPAR0053372; recordedBy: D.H. Janzen, W. Hallwachs & Lucia Rios; individualID: DHJPAR0053372; individualCount: 1; sex: female; lifeStage: adult; preparations: pinned; otherCatalogNumbers: ASHYM2726-13, 13-SRNP-21994, BOLD:AAA5134; **Taxon:** scientificName: Hyphantrophaga danausophaga; phylum: Arthropoda; class: Insecta; order: Diptera; family: Tachinidae; genus: Hyphantrophaga; specificEpithet: danausophaga; scientificNameAuthorship: Fleming & Wood, 2018; **Location:** continent: Central America; country: Costa Rica; countryCode: CR; stateProvince: Guanacaste; county: Sector El Hacha; locality: Area de Conservacion Guanacaste; verbatimLocality: Los Inocentes; verbatimElevation: 275; verbatimLatitude: 11.0324; verbatimLongitude: -85.4993; verbatimCoordinateSystem: Decimal; decimalLatitude: 11.0324; decimalLongitude: -85.4993; **Identification:** identifiedBy: AJ Fleming; dateIdentified: 2017; **Event:** samplingProtocol: Reared from the larva of the Nymphalidae, Danaus plexippus; verbatimEventDate: 23-Sep-2013; **Record Level:** language: en; institutionCode: CNC; collectionCode: Insects; basisOfRecord: Pinned Specimen
- **Type status:** Other material. **Occurrence:** occurrenceDetails: http://janzen.sas.upenn.edu; catalogNumber: DHJPAR0053373; recordedBy: D.H. Janzen, W. Hallwachs & Lucia Rios; individualID: DHJPAR0053373; individualCount: 1; sex: female; lifeStage: adult; preparations: pinned; otherCatalogNumbers: ASHYM2727-13, 13-SRNP-21990, BOLD:AAA5134; **Taxon:** scientificName: Hyphantrophaga danausophaga; phylum: Arthropoda; class: Insecta; order: Diptera; family: Tachinidae; genus: Hyphantrophaga; specificEpithet: danausophaga; scientificNameAuthorship: Fleming & Wood, 2018; **Location:** continent: Central America; country: Costa Rica; countryCode: CR; stateProvince: Guanacaste; county: Sector El Hacha; locality: Area de Conservacion Guanacaste; verbatimLocality: Los Inocentes; verbatimElevation: 275; verbatimLatitude: 11.0324; verbatimLongitude: -85.4993; verbatimCoordinateSystem: Decimal; decimalLatitude: 11.0324; decimalLongitude: -85.4993; **Identification:** identifiedBy: AJ Fleming; dateIdentified: 2017; **Event:** samplingProtocol: Reared from the larva of the Nymphalidae, Danaus plexippus; verbatimEventDate: 17-Sep-2013; **Record Level:** language: en; institutionCode: CNC; collectionCode: Insects; basisOfRecord: Pinned Specimen
- **Type status:** Other material. **Occurrence:** occurrenceDetails: http://janzen.sas.upenn.edu; catalogNumber: DHJPAR0053374; recordedBy: D.H. Janzen, W. Hallwachs & Lucia Rios; individualID: DHJPAR0053374; individualCount: 1; sex: male; lifeStage: adult; preparations: pinned; otherCatalogNumbers: ASHYM2728-13, 13-SRNP-22022, BOLD:AAA5134; **Taxon:** scientificName: Hyphantrophaga danausophaga; phylum: Arthropoda; class: Insecta; order: Diptera; family: Tachinidae; genus: Hyphantrophaga; specificEpithet: danausophaga; scientificNameAuthorship: Fleming & Wood, 2018; **Location:** continent: Central America; country: Costa Rica; countryCode: CR; stateProvince: Guanacaste; county: Sector El Hacha; locality: Area de Conservacion Guanacaste; verbatimLocality: Los Inocentes; verbatimElevation: 275; verbatimLatitude: 11.0324; verbatimLongitude: -85.4993; verbatimCoordinateSystem: Decimal; decimalLatitude: 11.0324; decimalLongitude: -85.4993; **Identification:** identifiedBy: AJ Fleming; dateIdentified: 2017; **Event:** samplingProtocol: Reared from the larva of the Nymphalidae, Danaus plexippus; verbatimEventDate: 23-Sep-2013; **Record Level:** language: en; institutionCode: CNC; collectionCode: Insects; basisOfRecord: Pinned Specimen
- **Type status:** Other material. **Occurrence:** occurrenceDetails: http://janzen.sas.upenn.edu; catalogNumber: DHJPAR0053377; recordedBy: D.H. Janzen, W. Hallwachs & Lucia Rios; individualID: DHJPAR0053377; individualCount: 1; sex: female; lifeStage: adult; preparations: pinned; otherCatalogNumbers: ASHYM2731-13, 13-SRNP-21991, BOLD:AAA5134; **Taxon:** scientificName: Hyphantrophaga danausophaga; phylum: Arthropoda; class: Insecta; order: Diptera; family: Tachinidae; genus: Hyphantrophaga; specificEpithet: danausophaga; scientificNameAuthorship: Fleming & Wood, 2018; **Location:** continent: Central America; country: Costa Rica; countryCode: CR; stateProvince: Guanacaste; county: Sector El Hacha; locality: Area de Conservacion Guanacaste; verbatimLocality: Los Inocentes; verbatimElevation: 275; verbatimLatitude: 11.0324; verbatimLongitude: -85.4993; verbatimCoordinateSystem: Decimal; decimalLatitude: 11.0324; decimalLongitude: -85.4993; **Identification:** identifiedBy: AJ Fleming; dateIdentified: 2017; **Event:** samplingProtocol: Reared from the larva of the Nymphalidae, Danaus plexippus; verbatimEventDate: 17-Sep-2013; **Record Level:** language: en; institutionCode: CNC; collectionCode: Insects; basisOfRecord: Pinned Specimen
- **Type status:** Other material. **Occurrence:** occurrenceDetails: http://janzen.sas.upenn.edu; catalogNumber: DHJPAR0053379; recordedBy: D.H. Janzen, W. Hallwachs & Lucia Rios; individualID: DHJPAR0053379; individualCount: 1; lifeStage: adult; preparations: pinned; otherCatalogNumbers: ASHYM2733-13, 13-SRNP-21866,; **Taxon:** scientificName: Hyphantrophaga danausophaga; phylum: Arthropoda; class: Insecta; order: Diptera; family: Tachinidae; genus: Hyphantrophaga; specificEpithet: danausophaga; scientificNameAuthorship: Fleming & Wood, 2018; **Location:** continent: Central America; country: Costa Rica; countryCode: CR; stateProvince: Guanacaste; county: Sector El Hacha; locality: Area de Conservacion Guanacaste; verbatimLocality: Los Inocentes; verbatimElevation: 275; verbatimLatitude: 11.0324; verbatimLongitude: -85.4993; verbatimCoordinateSystem: Decimal; decimalLatitude: 11.0324; decimalLongitude: -85.4993; **Identification:** identifiedBy: AJ Fleming; dateIdentified: 2017; **Event:** samplingProtocol: Reared from the larva of the Nymphalidae, Danaus plexippus; verbatimEventDate: 11-Sep-2013; **Record Level:** language: en; institutionCode: CNC; collectionCode: Insects; basisOfRecord: Pinned Specimen
- **Type status:** Other material. **Occurrence:** occurrenceDetails: http://janzen.sas.upenn.edu; catalogNumber: DHJPAR0053380; recordedBy: D.H. Janzen, W. Hallwachs & Lucia Rios; individualID: DHJPAR0053380; individualCount: 1; sex: male; lifeStage: adult; preparations: pinned; otherCatalogNumbers: ASHYM2734-13, 13-SRNP-21872, BOLD:AAA5134; **Taxon:** scientificName: Hyphantrophaga danausophaga; phylum: Arthropoda; class: Insecta; order: Diptera; family: Tachinidae; genus: Hyphantrophaga; specificEpithet: danausophaga; scientificNameAuthorship: Fleming & Wood, 2018; **Location:** continent: Central America; country: Costa Rica; countryCode: CR; stateProvince: Guanacaste; county: Sector El Hacha; locality: Area de Conservacion Guanacaste; verbatimLocality: Los Inocentes; verbatimElevation: 275; verbatimLatitude: 11.0324; verbatimLongitude: -85.4993; verbatimCoordinateSystem: Decimal; decimalLatitude: 11.0324; decimalLongitude: -85.4993; **Identification:** identifiedBy: AJ Fleming; dateIdentified: 2017; **Event:** samplingProtocol: Reared from the larva of the Nymphalidae, Danaus plexippus; verbatimEventDate: 11-Sep-2013; **Record Level:** language: en; institutionCode: CNC; collectionCode: Insects; basisOfRecord: Pinned Specimen
- **Type status:** Other material. **Occurrence:** occurrenceDetails: http://janzen.sas.upenn.edu; catalogNumber: DHJPAR0053383; recordedBy: D.H. Janzen, W. Hallwachs & Lucia Rios; individualID: DHJPAR0053383; individualCount: 1; sex: male; lifeStage: adult; preparations: pinned; otherCatalogNumbers: ASHYM2737-13, 13-SRNP-21871, BOLD:AAA5134; **Taxon:** scientificName: Hyphantrophaga danausophaga; phylum: Arthropoda; class: Insecta; order: Diptera; family: Tachinidae; genus: Hyphantrophaga; specificEpithet: danausophaga; scientificNameAuthorship: Fleming & Wood, 2018; **Location:** continent: Central America; country: Costa Rica; countryCode: CR; stateProvince: Guanacaste; county: Sector El Hacha; locality: Area de Conservacion Guanacaste; verbatimLocality: Los Inocentes; verbatimElevation: 275; verbatimLatitude: 11.0324; verbatimLongitude: -85.4993; verbatimCoordinateSystem: Decimal; decimalLatitude: 11.0324; decimalLongitude: -85.4993; **Identification:** identifiedBy: AJ Fleming; dateIdentified: 2017; **Event:** samplingProtocol: Reared from the larva of the Nymphalidae, Danaus plexippus; verbatimEventDate: 13-Sep-2013; **Record Level:** language: en; institutionCode: CNC; collectionCode: Insects; basisOfRecord: Pinned Specimen
- **Type status:** Other material. **Occurrence:** occurrenceDetails: http://janzen.sas.upenn.edu; catalogNumber: DHJPAR0053384; recordedBy: D.H. Janzen, W. Hallwachs & Lucia Rios; individualID: DHJPAR0053384; individualCount: 1; sex: male; lifeStage: adult; preparations: pinned; otherCatalogNumbers: ASHYM2738-13, 13-SRNP-21767, BOLD:AAA5134; **Taxon:** scientificName: Hyphantrophaga danausophaga; phylum: Arthropoda; class: Insecta; order: Diptera; family: Tachinidae; genus: Hyphantrophaga; specificEpithet: danausophaga; scientificNameAuthorship: Fleming & Wood, 2018; **Location:** continent: Central America; country: Costa Rica; countryCode: CR; stateProvince: Guanacaste; county: Sector El Hacha; locality: Area de Conservacion Guanacaste; verbatimLocality: Los Inocentes; verbatimElevation: 275; verbatimLatitude: 11.0324; verbatimLongitude: -85.4993; verbatimCoordinateSystem: Decimal; decimalLatitude: 11.0324; decimalLongitude: -85.4993; **Identification:** identifiedBy: AJ Fleming; dateIdentified: 2017; **Event:** samplingProtocol: Reared from the larva of the Nymphalidae, Danaus plexippus; verbatimEventDate: 01-Sep-2013; **Record Level:** language: en; institutionCode: CNC; collectionCode: Insects; basisOfRecord: Pinned Specimen
- **Type status:** Other material. **Occurrence:** occurrenceDetails: http://janzen.sas.upenn.edu; catalogNumber: DHJPAR0053387; recordedBy: D.H. Janzen, W. Hallwachs & Lucia Rios; individualID: DHJPAR0053387; individualCount: 1; sex: female; lifeStage: adult; preparations: pinned; otherCatalogNumbers: ASHYM2741-13, 13-SRNP-21858, BOLD:AAA5134; **Taxon:** scientificName: Hyphantrophaga danausophaga; phylum: Arthropoda; class: Insecta; order: Diptera; family: Tachinidae; genus: Hyphantrophaga; specificEpithet: danausophaga; scientificNameAuthorship: Fleming & Wood, 2018; **Location:** continent: Central America; country: Costa Rica; countryCode: CR; stateProvince: Guanacaste; county: Sector El Hacha; locality: Area de Conservacion Guanacaste; verbatimLocality: Los Inocentes; verbatimElevation: 275; verbatimLatitude: 11.0324; verbatimLongitude: -85.4993; verbatimCoordinateSystem: Decimal; decimalLatitude: 11.0324; decimalLongitude: -85.4993; **Identification:** identifiedBy: AJ Fleming; dateIdentified: 2017; **Event:** samplingProtocol: Reared from the larva of the Nymphalidae, Danaus plexippus; verbatimEventDate: 14-Sep-2013; **Record Level:** language: en; institutionCode: CNC; collectionCode: Insects; basisOfRecord: Pinned Specimen
- **Type status:** Other material. **Occurrence:** occurrenceDetails: http://janzen.sas.upenn.edu; catalogNumber: DHJPAR0053388; recordedBy: D.H. Janzen, W. Hallwachs & Lucia Rios; individualID: DHJPAR0053388; individualCount: 1; sex: male; lifeStage: adult; preparations: pinned; otherCatalogNumbers: ASHYM2742-13, 13-SRNP-21922, BOLD:AAA5134; **Taxon:** scientificName: Hyphantrophaga danausophaga; phylum: Arthropoda; class: Insecta; order: Diptera; family: Tachinidae; genus: Hyphantrophaga; specificEpithet: danausophaga; scientificNameAuthorship: Fleming & Wood, 2018; **Location:** continent: Central America; country: Costa Rica; countryCode: CR; stateProvince: Guanacaste; county: Sector El Hacha; locality: Area de Conservacion Guanacaste; verbatimLocality: Los Inocentes; verbatimElevation: 275; verbatimLatitude: 11.0324; verbatimLongitude: -85.4993; verbatimCoordinateSystem: Decimal; decimalLatitude: 11.0324; decimalLongitude: -85.4993; **Identification:** identifiedBy: AJ Fleming; dateIdentified: 2017; **Event:** samplingProtocol: Reared from the larva of the Nymphalidae, Danaus plexippus; verbatimEventDate: 16-Sep-2013; **Record Level:** language: en; institutionCode: CNC; collectionCode: Insects; basisOfRecord: Pinned Specimen
- **Type status:** Other material. **Occurrence:** occurrenceDetails: http://janzen.sas.upenn.edu; catalogNumber: DHJPAR0054978; recordedBy: D.H. Janzen, W. Hallwachs & Lucia Rios; individualID: DHJPAR0054978; individualCount: 1; sex: male; lifeStage: adult; preparations: pinned; otherCatalogNumbers: ASHYH1525-14, 13-SRNP-22908, BOLD:AAA5134; **Taxon:** scientificName: Hyphantrophaga danausophaga; phylum: Arthropoda; class: Insecta; order: Diptera; family: Tachinidae; genus: Hyphantrophaga; specificEpithet: danausophaga; scientificNameAuthorship: Fleming & Wood, 2018; **Location:** continent: Central America; country: Costa Rica; countryCode: CR; stateProvince: Guanacaste; county: Sector El Hacha; locality: Area de Conservacion Guanacaste; verbatimLocality: Estacion Los Almendros; verbatimElevation: 290; verbatimLatitude: 11.0323; verbatimLongitude: -85.5278; verbatimCoordinateSystem: Decimal; decimalLatitude: 11.0323; decimalLongitude: -85.5278; **Identification:** identifiedBy: AJ Fleming; dateIdentified: 2017; **Event:** samplingProtocol: Reared from the larva of the Nymphalidae, Danaus plexippus; verbatimEventDate: 07-Jan-2014; **Record Level:** language: en; institutionCode: CNC; collectionCode: Insects; basisOfRecord: Pinned Specimen
- **Type status:** Other material. **Occurrence:** occurrenceDetails: http://janzen.sas.upenn.edu; catalogNumber: DHJPAR0057601; recordedBy: D.H. Janzen, W. Hallwachs & Lucia Rios; individualID: DHJPAR0057601; individualCount: 1; lifeStage: adult; preparations: pinned; otherCatalogNumbers: ASTAX013-15, 15-SRNP-20226,; **Taxon:** scientificName: Hyphantrophaga danausophaga; phylum: Arthropoda; class: Insecta; order: Diptera; family: Tachinidae; genus: Hyphantrophaga; specificEpithet: danausophaga; scientificNameAuthorship: Fleming & Wood, 2018; **Location:** continent: Central America; country: Costa Rica; countryCode: CR; stateProvince: Guanacaste; county: Sector El Hacha; locality: Area de Conservacion Guanacaste; verbatimLocality: Rio Sabalo; verbatimCoordinateSystem: Decimal; **Identification:** identifiedBy: AJ Fleming; dateIdentified: 2017; **Event:** samplingProtocol: Reared from the larva of the Nymphalidae, Danaus plexippus; verbatimEventDate: unavailable, 2016; **Record Level:** language: en; institutionCode: CNC; collectionCode: Insects; basisOfRecord: Pinned Specimen
- **Type status:** Other material. **Occurrence:** occurrenceDetails: http://janzen.sas.upenn.edu; catalogNumber: DHJPAR0060975; recordedBy: D.H. Janzen, W. Hallwachs & Osvaldo Espinoza; individualID: DHJPAR0060975; individualCount: 1; lifeStage: adult; preparations: pinned; otherCatalogNumbers: ACGBA7396-17, 17-SRNP-605, BOLD:AAA5134; **Taxon:** scientificName: Hyphantrophaga danausophaga; phylum: Arthropoda; class: Insecta; order: Diptera; family: Tachinidae; genus: Hyphantrophaga; specificEpithet: danausophaga; scientificNameAuthorship: Fleming & Wood, 2018; **Location:** continent: Central America; country: Costa Rica; countryCode: CR; stateProvince: Guanacaste; county: Sector San Cristobal; locality: Area de Conservacion Guanacaste; verbatimLocality: Finca San Gabriel; verbatimElevation: 645; verbatimLatitude: 10.8777; verbatimLongitude: -85.3934; verbatimCoordinateSystem: Decimal; decimalLatitude: 10.8777; decimalLongitude: -85.3934; **Identification:** identifiedBy: AJ Fleming; dateIdentified: 2017; **Event:** samplingProtocol: Reared from the larva of the Nymphalidae, Danaus plexippus; verbatimEventDate: 13-Apr-2017; **Record Level:** language: en; institutionCode: CNC; collectionCode: Insects; basisOfRecord: Pinned Specimen
- **Type status:** Other material. **Occurrence:** occurrenceDetails: http://janzen.sas.upenn.edu; catalogNumber: DHJPAR0060977; recordedBy: D.H. Janzen, W. Hallwachs & Osvaldo Espinoza; individualID: DHJPAR0060977; individualCount: 1; lifeStage: adult; preparations: pinned; otherCatalogNumbers: ACGBA7398-17, 17-SRNP-602, BOLD:AAA5134; **Taxon:** scientificName: Hyphantrophaga danausophaga; phylum: Arthropoda; class: Insecta; order: Diptera; family: Tachinidae; genus: Hyphantrophaga; specificEpithet: danausophaga; scientificNameAuthorship: Fleming & Wood, 2018; **Location:** continent: Central America; country: Costa Rica; countryCode: CR; stateProvince: Guanacaste; county: Sector San Cristobal; locality: Area de Conservacion Guanacaste; verbatimLocality: Finca San Gabriel; verbatimElevation: 645; verbatimLatitude: 10.8777; verbatimLongitude: -85.3934; verbatimCoordinateSystem: Decimal; decimalLatitude: 10.8777; decimalLongitude: -85.3934; **Identification:** identifiedBy: AJ Fleming; dateIdentified: 2017; **Event:** samplingProtocol: Reared from the larva of the Nymphalidae, Danaus plexippus; verbatimEventDate: 08-Apr-2017; **Record Level:** language: en; institutionCode: CNC; collectionCode: Insects; basisOfRecord: Pinned Specimen
- **Type status:** Other material. **Occurrence:** occurrenceDetails: http://janzen.sas.upenn.edu; catalogNumber: DHJPAR0060978; recordedBy: D.H. Janzen, W. Hallwachs & Osvaldo Espinoza; individualID: DHJPAR0060978; individualCount: 1; lifeStage: adult; preparations: pinned; otherCatalogNumbers: ACGBA7399-17, 17-SRNP-603, BOLD:AAA5134; **Taxon:** scientificName: Hyphantrophaga danausophaga; phylum: Arthropoda; class: Insecta; order: Diptera; family: Tachinidae; genus: Hyphantrophaga; specificEpithet: danausophaga; scientificNameAuthorship: Fleming & Wood, 2018; **Location:** continent: Central America; country: Costa Rica; countryCode: CR; stateProvince: Guanacaste; county: Sector San Cristobal; locality: Area de Conservacion Guanacaste; verbatimLocality: Finca San Gabriel; verbatimElevation: 645; verbatimLatitude: 10.8777; verbatimLongitude: -85.3934; verbatimCoordinateSystem: Decimal; **Identification:** identifiedBy: AJ Fleming; dateIdentified: 2017; **Event:** samplingProtocol: Reared from the larva of the Nymphalidae, Danaus plexippus; verbatimEventDate: 08-Apr-2017; **Record Level:** language: en; institutionCode: CNC; collectionCode: Insects; basisOfRecord: Pinned Specimen

#### Description

**Male** (Fig. 9). Length: 7–11mm. **Head** (Fig. 9b): vertex 1/4 of head width; two reclinate upper orbital setae; ocellar setae arising beside anterior ocellus; ocellar triangle of dark blackened gold colour, with light gold tomentum around margin; fronto-orbital plate dull grey tomentose with slight gold tinge, gold stronger around vertex; eye densely haired; fronto-orbital plate setulose, setulae not extending below lowest frontal seta; fronto-orbital plate shiny silver; parafacial bare; facial ridge bare; pedicel black with a small spot of orange basally, sometimes extending into adjacent region of postpedicel; otherwise concolorous with postpedicel; arista black, very minutely pubescent, gradually tapered apically, beginning on basal 1/3–1/4; palpus ranging from yellow to brown. **Thorax** (Fig. 9a, c): entirely densely hirsute with short black setulae amongst setae; prosternum setose with 1–3 strong setae surrounded by a brush of weaker setulae; four prominent dorsal vittae, outermost two broken across suture, innermost pair unbroken across suture, not reaching beyond 2nd postsutural dorsocentral seta; postpronotum with five setae arranged in a triangle; chaetotaxy: acrostichal setae 3:3; dorsocentral setae 3:4; intra-alar setae 3:3; supra-alar setae 2:3; katepisternum with three setae, basal seta weakest, arising anterior to suture; lateral scutellar setae 1/2 as long as subapical setae, slightly curving inwards medially; subapical setae subequal in length of basal scutellar setae, the latter arising above plane of remaining marginal scutellar setae; apical scutellar setae ranging from 1/2 as long as lateral scutellar setae to subequal in length but 1/2 the diameter; one pair of discal scutellar setae; scutellum gold tomentose along posterior margin, transitioning to grey tomentose along basal edge. **Legs** (Fig. 9c): femora and coxae black in ground colour; tibiae yellow in ground colour, densely covered in short black hairs; appearing darkened almost black; fore femur with dense silver tomentum on posterodorsal surface; hind coxa either bare or with a single seta along posterior margin. **Wing** (Fig. 9a): pale translucent, not distinctly infuscate; vein R_4+5_ with 2–3 (most often 2) setulae at base. **Abdomen** (Fig. 9a, c): ground colour brown; middorsal depression on ST1+2 extending almost to margin; median marginal setae absent on ST1+2 and T3; a complete row of marginal setae present on T4; discal setae only on T5; sex patch covering ventral surfaces of T4–T5 and posterior 1/3 of T3; distinct tomentose bands along anterior 2/3 of T3 and T4, broken medially by a dorsocentral stripe; T5 with silver tomentum over its entirety. **Terminalia**: sternite 5 (Fig. 9f) with a deeply excavated median cleft, smoothly U-shaped, margins covered in dense tomentum. Lateral lobes of sternite rounded apically, with 2–3 strong setae surrounded by many shorter, weaker setulae. Anterior plate of sternite 5 from subequal to slightly longer than apical lobes; unsclerotised "window" appearing blunt convex, with a slightly rectangular base and a wider crown making it appear mushroom-shaped, as wide as median cleft. Cerci in posterior view (Fig. 9d) rectangular and slightly shorter than surstyli, blunt and rounded at apex, completely separate medially, appearing slightly divergent basally, twice as wide as at apex; in lateral view with a slight downward curve in apical 1/3; densely setulose along basal 2/3 dorsally, setulose ventrally along entire length (visible in lateral view). Surstylus in lateral view (Fig. 9e) narrow, almost parallel-sided along its length, ending in a slightly downcurved apex, making the structure appear blade-like; when viewed dorsally surstyli appearing to point outward. Pregonite short and wide, well-developed, subequal in length to postgonite, as long as distiphallus, bare and squared off apically. Postgonite slightly narrow, 1/3 as wide as pregonite, sharply pointed and curved at apex. Distiphallus rectangular, only weakly flaring apically with a slender median longitudinal sclerotised reinforcement on its posterior surface and a broad, anterolateral, apically clubbed sclerotised acrophallus on each side, joining the plate of opposite side on anterior surface near apex.

**Female**. Length: 8–11 mm. As male, differing only by the presence of two pairs of proclinate orbital setae.

#### Diagnosis

To date, *Hyphantrophaga danausophaga* **sp. n.** can only be distinguished from its closest congener, *H. morphophaga* **sp. n.**, by its host selection and habitat, being found parasitising only medium-sized, naked, ringed Nymphalidae larvae in open, heavily insolated grassland and pasture habitats.

#### Etymology

Named after the host genus, *Danaus* and the Greek "phago" meaning "eating", in reference to its primary host species.

#### Distribution

Costa Rica, ACG, Alajuela and Guanacaste Provinces, 90–1150 m elevation.

#### Ecology

*Hyphantrophaga danausophaga* **sp. n.** has been reared 105 times from three species of Lepidoptera in the family Nymphalidae, *Danaus plexippus* (Linnaeus, 1758), *Danaus gilippus* (Cramer, 1775) and *Lycorea atergatis* (Doubleday, 1847), in cloud forest, rain forest, dry forest and dry-rain lowland intergrade.

### Hyphantrophaga diniamartinezae

Fleming & Wood sp. n.

urn:lsid:zoobank.org:act:3D997452-E42A-40CD-80B8-18221259BA18

#### Materials

- **Type status:** Holotype. **Occurrence:** occurrenceDetails: http://janzen.sas.upenn.edu; catalogNumber: DHJPAR0045610; recordedBy: D.H. Janzen, W. Hallwachs & Ricardo Calero; individualID: DHJPAR0045610; individualCount: 1; sex: male; lifeStage: adult; preparations: pinned; otherCatalogNumbers: ACGAZ799-11, 11-SRNP-71939, BOLD:AAM3713; **Taxon:** scientificName: Hyphantrophaga diniamartinezae; phylum: Arthropoda; class: Insecta; order: Diptera; family: Tachinidae; genus: Hyphantrophaga; specificEpithet: diniamartinezae; scientificNameAuthorship: Fleming & Wood, 2018; **Location:** continent: Central America; country: Costa Rica; countryCode: CR; stateProvince: Guanacaste; county: Sector Pitilla; locality: Area de Conservacion Guanacaste; verbatimLocality: Manguera; verbatimElevation: 470; verbatimLatitude: 10.9959; verbatimLongitude: -85.3984; verbatimCoordinateSystem: Decimal; decimalLatitude: 10.9959; decimalLongitude: -85.3984; **Identification:** identifiedBy: AJ Fleming; dateIdentified: 2017; **Event:** samplingProtocol: Reared from the larva of the Sphingidae, Xylophanes ceratomioides; verbatimEventDate: 06-Oct-2011; **Record Level:** language: en; institutionCode: CNC; collectionCode: Insects; basisOfRecord: Pinned Specimen
- **Type status:** Paratype. **Occurrence:** occurrenceDetails: http://janzen.sas.upenn.edu; catalogNumber: DHJPAR0008100; recordedBy: D.H. Janzen, W. Hallwachs & Jose Perez; individualID: DHJPAR0008100; individualCount: 1; sex: female; lifeStage: adult; preparations: pinned; otherCatalogNumbers: ASTAT872-06, 00-SRNP-20702, BOLD:AAM3713; **Taxon:** scientificName: Hyphantrophaga diniamartinezae; phylum: Arthropoda; class: Insecta; order: Diptera; family: Tachinidae; genus: Hyphantrophaga; specificEpithet: diniamartinezae; scientificNameAuthorship: Fleming & Wood, 2018; **Location:** continent: Central America; country: Costa Rica; countryCode: CR; stateProvince: Alajuela; county: Sector Rincon Rain Forest; locality: Area de Conservacion Guanacaste; verbatimLocality: Quebrada Escondida; verbatimElevation: 420; verbatimLatitude: 10.8993; verbatimLongitude: -85.2749; verbatimCoordinateSystem: Decimal; decimalLatitude: 10.8993; decimalLongitude: -85.2749; **Identification:** identifiedBy: AJ Fleming; dateIdentified: 2017; **Event:** samplingProtocol: Reared from the larva of the Sphingidae, Xylophanes chiron; verbatimEventDate: 04-Dec-2000; **Record Level:** language: en; institutionCode: CNC; collectionCode: Insects; basisOfRecord: Pinned Specimen
- **Type status:** Paratype. **Occurrence:** occurrenceDetails: http://janzen.sas.upenn.edu; catalogNumber: DHJPAR0023083; recordedBy: D.H. Janzen, W. Hallwachs & Roster Moraga; individualID: DHJPAR0023083; individualCount: 1; sex: female; lifeStage: adult; preparations: pinned; otherCatalogNumbers: ASTAW244-08, 07-SRNP-24104, BOLD:AAM3713; **Taxon:** scientificName: Hyphantrophaga diniamartinezae; phylum: Arthropoda; class: Insecta; order: Diptera; family: Tachinidae; genus: Hyphantrophaga; specificEpithet: diniamartinezae; scientificNameAuthorship: Fleming & Wood, 2018; **Location:** continent: Central America; country: Costa Rica; countryCode: CR; stateProvince: Guanacaste; county: Sector Del Oro; locality: Area de Conservacion Guanacaste; verbatimLocality: Margarita; verbatimElevation: 380; verbatimLatitude: 11.0323; verbatimLongitude: -85.4395; verbatimCoordinateSystem: Decimal; decimalLatitude: 11.0323; decimalLongitude: -85.4395; **Identification:** identifiedBy: AJ Fleming; dateIdentified: 2017; **Event:** samplingProtocol: Reared from the larva of the Sphingidae, Xylophanes porcus; verbatimEventDate: 27-Nov-2007; **Record Level:** language: en; institutionCode: CNC; collectionCode: Insects; basisOfRecord: Pinned Specimen
- **Type status:** Paratype. **Occurrence:** occurrenceDetails: http://janzen.sas.upenn.edu; catalogNumber: DHJPAR0008093; recordedBy: D.H. Janzen, W. Hallwachs & Freyci Vargas; individualID: DHJPAR0008093; individualCount: 1; sex: male; lifeStage: adult; preparations: pinned; otherCatalogNumbers: ASTAT865-06, 00-SRNP-20696, BOLD:AAM3713; **Taxon:** scientificName: Hyphantrophaga diniamartinezae; phylum: Arthropoda; class: Insecta; order: Diptera; family: Tachinidae; genus: Hyphantrophaga; specificEpithet: diniamartinezae; scientificNameAuthorship: Fleming & Wood, 2018; **Location:** continent: Central America; country: Costa Rica; countryCode: CR; stateProvince: Alajuela; county: Sector Rincon Rain Forest; locality: Area de Conservacion Guanacaste; verbatimLocality: Quebrada Escondida; verbatimElevation: 420; verbatimLatitude: 10.8993; verbatimLongitude: -85.2749; verbatimCoordinateSystem: Decimal; decimalLatitude: 10.8993; decimalLongitude: -85.2749; **Identification:** identifiedBy: AJ Fleming; dateIdentified: 2017; **Event:** samplingProtocol: Reared from the larva of the Sphingidae, Xylophanes chiron; verbatimEventDate: 01-Dec-2000; **Record Level:** language: en; institutionCode: CNC; collectionCode: Insects; basisOfRecord: Pinned Specimen

#### Description

**Male** (Fig. 10). Length: 7–10 mm. **Head** (Fig. 10b): vertex 1/5 of head width; two pairs of reclinate upper orbital setae; ocellar setae arising behind anterior ocellus; ocellar triangle pale brassy; fronto-orbital plate pale brassy on upper 80%, densely setulose, setulae not extending below lowest frontal seta; parafacial silver and bare; eye densely haired; facial ridge bare; pedicel black, concolorous with postpedicel; arista black, very minutely pubescent, distinctly thickened on basal 1/3–1/4; palpus yellow and haired apically, narrow and digitiform. **Thorax** (Fig. 10a, c): dull brassy tomentose dorsally, contrasting with slightly brighter silver tomentose laterally, lateral surfaces with dense dark setulae making it appear darker; thorax with dense dark hairs interspersed amongst setae; four thick dorsal vittae, outermost two broken across suture, innermost pair unbroken, reaching 3rd postsutural dorsocentral seta, both pairs of vittae widening postsuturally and smudging together; postpronotum with 3–4 setae arranged in a triangle; chaetotaxy: acrostichal setae 3:3; dorsocentral setae 3:4; intra-alar setae 3–4:3; supra-alar setae 2:3; three katepisternal setae; basal scutellar setae subequal in length to subapical scutellar setae; lateral scutellar setae less than 2/3 as long as subapical setae, strongly curving inwards medially; apical scutellar setae subequal in length to lateral scutellar setae, crossed apically; one pair of discal scutellar setae more widely set than apical scutellar setae, but more narrowly than subapical scutellar setae; scutellum very slightly darkened across basal 25%, remainder concolorous with scutum. **Legs** (Fig. 10c): black in ground colour; fore femur with dense silver tomentum on posterodorsal surface; hind coxa bare. **Wing** (Fig. 10a): pale translucent, hyaline; vein R_4+5_ with only 2–3 setulae at base. **Abdomen** (Fig. 10a, c): ground colour black dorsally, yellow lateroventrally; middorsal depression on ST1+2 reaching hind margin; median marginal setae present on ST1+2–T3; a complete row of marginal setae present on T4; discal setae only on T5; sex patch covering ventral surfaces of T4–T5; distinct brassy tomentose bands along anterior edge of T3 and T4, broken medially by a dorsocentral stripe and covering almost 80% of tergites; T3 with silver tomentum ventrolaterally over 60% of surface; T5 with brassy tomentum throughout. **Terminalia** (Fig. 10d, e, f): sternite 5 (Fig. 10f) with a deeply excavated median cleft, widely U-shaped, margins covered in dense tomentum, unsclerotised window tri-lobed and "fleur-de-lys"-shaped. Lateral lobes of sternite pointed apically, many short, stout setae throughout. Anterior plate of sternite 5 2X longer than apical lobes. Cerci in posterior view (Fig. 10d) rounded rectangular-shaped, subequal in length to surstyli, blunt and rounded at apex, completely separate medially, parallel; in lateral view (Fig. 10e) with an evenly rounded downward curve throughout; densely setulose along basal 2/3. Surstylus in lateral view round, tapered and straight along bottom edge; opposite edge evenly rounded, giving it a cleaver-blade appearance; when viewed dorsally, surstyli pointing outward, not strongly convergent. Pregonite short, not well-developed, 0.5 times as long as distiphallus, bare and rounded apically, having the appearance of an upside down boot. Postgonite slightly narrow, 1/3 as wide as pregonite, sharply pointed and curved at apex. Epiphallus well-developed and apically hooked. Distiphallus rectangular with a slender median longitudinal sclerotised reinforcement on its posterior surface and a broad, anterolateral, sclerotised acrophallus on each side, joining on anterior surface near apex.

**Female**. Length: 8–10 mm. As male, differing only by the presence of two pairs of proclinate orbital setae.

#### Diagnosis

*Hyphantrophaga diniamartinezae* **sp. n.** can be distinguished from all other *Hyphantrophaga* species by the following combination of traits: thorax with three postsutural acrostichal setae, four postsutural dorsocentral setae, and three katepisternal setae, legs black, hind coxa bare, tomentum covering less than 50% of abdominal T3, ventral edge of T3 darkened up to 10% of tergite and T5 with brassy tomentum throughout.

#### Etymology

*Hyphantrophaga diniamartinezae* **sp. n.** is named in recognition of Dinia Maria Martinez Cheves' dedication and work in finding and rearing the ACG caterpillars that contained tachinid larvae.

#### Distribution

Costa Rica, ACG, Guanacaste Province, 380–420 m elevation.

#### Ecology

*Hyphantrophaga diniamartinezae* **sp. n.** has been reared two times from two species of Lepidoptera in the family Sphingidae, *Xylophanes chiron* (Drury, 1773) and *Xylophanes porcus* (Hübner, 1823), in rain forest and dry-rain lowland intergrades.

### Hyphantrophaga duniagarciae

Fleming & Wood sp. n.

urn:lsid:zoobank.org:act:1F6DC396-3A43-489B-A7BA-BD1B7FDA7B1B

#### Materials

- **Type status:** Holotype. **Occurrence:** occurrenceDetails: http://janzen.sas.upenn.edu; catalogNumber: DHJPAR0011464; recordedBy: D.H. Janzen, W. Hallwachs & Guillermo Pereira; individualID: DHJPAR0011464; individualCount: 1; sex: female; lifeStage: adult; preparations: pinned; otherCatalogNumbers: ASTAQ851-06, 04-SRNP-12099; **Taxon:** scientificName: Hyphantrophaga duniagarciae; phylum: Arthropoda; class: Insecta; order: Diptera; family: Tachinidae; genus: Hyphantrophaga; specificEpithet: duniagarciae; scientificNameAuthorship: Fleming & Wood, 2018; **Location:** continent: Central America; country: Costa Rica; countryCode: CR; stateProvince: Guanacaste; county: Sector Pocosol; locality: Area de Conservacion Guanacaste; verbatimLocality: Casa Centeno; verbatimElevation: 215; verbatimLatitude: 10.8784; verbatimLongitude: -85.5728; verbatimCoordinateSystem: Decimal; decimalLatitude: 10.8784; decimalLongitude: -85.5728; **Identification:** identifiedBy: AJ Fleming; dateIdentified: 2017; **Event:** samplingProtocol: Reared from the larva of the Zygaenidae, Neoilliberis thyesta; verbatimEventDate: 06-Jun-2005; **Record Level:** language: en; institutionCode: CNC; collectionCode: Insects; basisOfRecord: Pinned Specimen

#### Description

**Male**. Not known at this time.

**Female** (Fig. 11). Length: 4 mm. **Head** (Fig. 11b): vertex 1/3 of head width; two pairs of reclinate upper orbital setae and two pairs of proclinate orbital setae; ocellar setae arising behind anterior ocellus; ocellar triangle brilliant silver; fronto-orbital plate pale brassy on upper 50% coded as silver, remainder dull silver, sparsely setulose, only one or two setulae extending below lowest orbital seta; parafacial silver and bare; eye densely setulose; facial ridge bare; pedicel black, concolorous with postpedicel; arista black, very minutely pubescent, distinctly thickened on basal 1/3–1/4; palpus yellow and haired apically, narrow and digitiform. **Thorax** (Fig. 11a, c): dull grey tomentose dorsally, appearing almost brassy when viewed under certain angles, contrasting with slightly more silver-grey tomentose laterally; dark setulae on both dorsal and lateral surfaces; four thick dorsal vittae, outermost two broken across suture, innermost pair unbroken, reaching 2nd postsutural dorsocentral seta; postpronotum with three setae arranged in an obtuse triangle; chaetotaxy: acrostichal setae 2:3; dorsocentral setae 3:4; intra-alar setae 2:3; supra-alar setae 2:3; three katepisternal setae, basal seta extremely weak and anterior to suture; basal scutellar setae subequal in length to subapical scutellar setae; lateral scutellar setae less than 2/3 as long as subapical setae, strongly curving inwards medially; apical scutellar setae 1/2 length of lateral scutellar setae, crossed apically; one pair of discal scutellar setae more widely set than subapical scutellar setae; scutellum very slightly darkened across basal 15%, remainder concolorous with scutum. **Legs** (Fig. 11c): black in ground colour; fore femur with dense silver tomentum on posterodorsal surface; hind coxa setose. **Wing** (Fig. 11a): pale translucent, hyaline; vein R_4+5_ with only one setula at base. **Abdomen** (Fig. 11a, c): ground colour black; middorsal depression on ST1+2 almost reaching hind margin; median marginal setae present on ST1+2–T3; a complete row of marginal setae present on T4–T5; discal setae present on T3–T5; distinct brassy tomentose bands along anterior edge of T3–T5 unbroken medially, covering almost 70% of tergites; T5 with silver tomentum throughout. **Terminalia**: not examined.

#### Diagnosis

*Hyphantrophaga duniagarciae* **sp. n.** can be distinguished from all other *Hyphantrophaga* species by the following combination of traits: fronto-orbital plate pale brassy on upper 50% (so pale coded as silver), remainder dull silver, pedicel black, four postsutural dorsocentral setae, thorax entirely silver/grey tomentose, legs black, hind coxa setose, median marginal setae present on ST1+2, discal setae present T3–T5.

#### Etymology

*Hyphantrophaga duniagarciae* **sp. n.** is named in recognition of Dunia Garcia Garcia's dedication and work in finding and rearing the ACG caterpillars that contained tachinid larvae.

#### Distribution

Costa Rica, ACG, Guanacaste Province, 215 m elevation.

#### Ecology

*Hyphantrophaga duniagarciae* **sp. n.** has been reared six times from one species of Lepidopterain the family Zygaenidae, Neoilliberis thyesta (Druce, 1884), in dry forest.

### Hyphantrophaga edwinapui

Fleming & Wood sp. n.

urn:lsid:zoobank.org:act:F8AEEBB3-E9A6-4067-8F75-F5E2496EFE1B

#### Materials

- **Type status:** Holotype. **Occurrence:** occurrenceDetails: http://janzen.sas.upenn.edu; catalogNumber: DHJPAR0007463; recordedBy: D.H. Janzen, W. Hallwachs & gusaneros; individualID: DHJPAR0007463; individualCount: 1; sex: male; lifeStage: adult; preparations: pinned; otherCatalogNumbers: ASTAT235-06, 01-SRNP-17538, BOLD:AAC5464; **Taxon:** scientificName: Hyphantrophaga edwinapui; phylum: Arthropoda; class: Insecta; order: Diptera; family: Tachinidae; genus: Hyphantrophaga; specificEpithet: edwinapui; scientificNameAuthorship: Fleming & Wood, 2017; **Location:** continent: Central America; country: Costa Rica; countryCode: CR; stateProvince: Guanacaste; county: Sector Santa Rosa; locality: Area de Conservacion Guanacaste; verbatimLocality: Quebrada Cebollines; verbatimElevation: 270; verbatimLatitude: 10.8222; verbatimLongitude: -85.6434; verbatimCoordinateSystem: Decimal; decimalLatitude: 10.8222; decimalLongitude: -85.6434; **Identification:** identifiedBy: AJ Fleming; dateIdentified: 2017; **Event:** samplingProtocol: Reared from the larva of the Doidae, Doa Janzen01; verbatimEventDate: 02-Nov-2001; **Record Level:** language: en; institutionCode: CNC; collectionCode: Insects; basisOfRecord: Pinned Specimen
- **Type status:** Paratype. **Occurrence:** occurrenceDetails: http://janzen.sas.upenn.edu; catalogNumber: DHJPAR0007465; recordedBy: D.H. Janzen, W. Hallwachs & gusaneros; individualID: DHJPAR0007465; individualCount: 1; sex: female; lifeStage: adult; preparations: pinned; otherCatalogNumbers: ASTAT237-06, 01-SRNP-17505,; **Taxon:** scientificName: Hyphantrophaga edwinapui; phylum: Arthropoda; class: Insecta; order: Diptera; family: Tachinidae; genus: Hyphantrophaga; specificEpithet: edwinapui; scientificNameAuthorship: Fleming & Wood, 2017; **Location:** continent: Central America; country: Costa Rica; countryCode: CR; stateProvince: Guanacaste; county: Sector Santa Rosa; locality: Area de Conservacion Guanacaste; verbatimLocality: Quebrada Cebollines; verbatimElevation: 270; verbatimLatitude: 10.8222; verbatimLongitude: -85.6434; verbatimCoordinateSystem: Decimal; decimalLatitude: 10.8222; decimalLongitude: -85.6434; **Identification:** identifiedBy: AJ Fleming; dateIdentified: 2017; **Event:** samplingProtocol: Reared from the larva of the Doidae, Doa Janzen01; verbatimEventDate: 08-Nov-2001; **Record Level:** language: en; institutionCode: CNC; collectionCode: Insects; basisOfRecord: Pinned Specimen
- **Type status:** Paratype. **Occurrence:** occurrenceDetails: http://janzen.sas.upenn.edu; catalogNumber: DHJPAR0007469; recordedBy: D.H. Janzen, W. Hallwachs & gusaneros; individualID: DHJPAR0007469; individualCount: 1; sex: male; lifeStage: adult; preparations: pinned; otherCatalogNumbers: ASTAT241-06, 01-SRNP-17485, BOLD:AAC5464; **Taxon:** scientificName: Hyphantrophaga edwinapui; phylum: Arthropoda; class: Insecta; order: Diptera; family: Tachinidae; genus: Hyphantrophaga; specificEpithet: edwinapui; scientificNameAuthorship: Fleming & Wood, 2017; **Location:** continent: Central America; country: Costa Rica; countryCode: CR; stateProvince: Guanacaste; county: Sector Santa Rosa; locality: Area de Conservacion Guanacaste; verbatimLocality: Quebrada Cebollines; verbatimElevation: 270; verbatimLatitude: 10.8222; verbatimLongitude: -85.6434; verbatimCoordinateSystem: Decimal; decimalLatitude: 10.8222; decimalLongitude: -85.6434; **Identification:** identifiedBy: AJ Fleming; dateIdentified: 2017; **Event:** samplingProtocol: Reared from the larva of the Doidae, Doa Janzen01; verbatimEventDate: 05-Nov-2001; **Record Level:** language: en; institutionCode: CNC; collectionCode: Insects; basisOfRecord: Pinned Specimen
- **Type status:** Paratype. **Occurrence:** occurrenceDetails: http://janzen.sas.upenn.edu; catalogNumber: DHJPAR0007474; recordedBy: D.H. Janzen, W. Hallwachs & gusaneros; individualID: DHJPAR0007474; individualCount: 1; sex: male; lifeStage: adult; preparations: pinned; otherCatalogNumbers: ASTAT246-06, 01-SRNP-17509, BOLD:AAC5464; **Taxon:** scientificName: Hyphantrophaga edwinapui; phylum: Arthropoda; class: Insecta; order: Diptera; family: Tachinidae; genus: Hyphantrophaga; specificEpithet: edwinapui; scientificNameAuthorship: Fleming & Wood, 2017; **Location:** continent: Central America; country: Costa Rica; countryCode: CR; stateProvince: Guanacaste; county: Sector Santa Rosa; locality: Area de Conservacion Guanacaste; verbatimLocality: Quebrada Cebollines; verbatimElevation: 270; verbatimLatitude: 10.8222; verbatimLongitude: -85.6434; verbatimCoordinateSystem: Decimal; decimalLatitude: 10.8222; decimalLongitude: -85.6434; **Identification:** identifiedBy: AJ Fleming; dateIdentified: 2017; **Event:** samplingProtocol: Reared from the larva of the Doidae, Doa Janzen01; verbatimEventDate: 07-Nov-2001; **Record Level:** language: en; institutionCode: CNC; collectionCode: Insects; basisOfRecord: Pinned Specimen
- **Type status:** Paratype. **Occurrence:** occurrenceDetails: http://janzen.sas.upenn.edu; catalogNumber: DHJPAR0007470; recordedBy: D.H. Janzen, W. Hallwachs & gusaneros; individualID: DHJPAR0007470; individualCount: 1; sex: male; lifeStage: adult; preparations: pinned; otherCatalogNumbers: ASTAT242-06, 01-SRNP-17477, BOLD:AAC5464; **Taxon:** scientificName: Hyphantrophaga edwinapui; phylum: Arthropoda; class: Insecta; order: Diptera; family: Tachinidae; genus: Hyphantrophaga; specificEpithet: edwinapui; scientificNameAuthorship: Fleming & Wood, 2017; **Location:** continent: Central America; country: Costa Rica; countryCode: CR; stateProvince: Guanacaste; county: Sector Santa Rosa; locality: Area de Conservacion Guanacaste; verbatimLocality: Quebrada Cebollines; verbatimElevation: 270; verbatimLatitude: 10.8222; verbatimLongitude: -85.6434; verbatimCoordinateSystem: Decimal; decimalLatitude: 10.8222; decimalLongitude: -85.6434; **Identification:** identifiedBy: AJ Fleming; dateIdentified: 2017; **Event:** samplingProtocol: Reared from the larva of the Doidae, Doa Janzen01; verbatimEventDate: 07-Nov-2001; **Record Level:** language: en; institutionCode: CNC; collectionCode: Insects; basisOfRecord: Pinned Specimen
- **Type status:** Paratype. **Occurrence:** occurrenceDetails: http://janzen.sas.upenn.edu; catalogNumber: DHJPAR0007472; recordedBy: D.H. Janzen, W. Hallwachs & gusaneros; individualID: DHJPAR0007472; individualCount: 1; sex: male; lifeStage: adult; preparations: pinned; otherCatalogNumbers: ASTAT244-06, 01-SRNP-17492, BOLD:AAC5464; **Taxon:** scientificName: Hyphantrophaga edwinapui; phylum: Arthropoda; class: Insecta; order: Diptera; family: Tachinidae; genus: Hyphantrophaga; specificEpithet: edwinapui; scientificNameAuthorship: Fleming & Wood, 2017; **Location:** continent: Central America; country: Costa Rica; countryCode: CR; stateProvince: Guanacaste; county: Sector Santa Rosa; locality: Area de Conservacion Guanacaste; verbatimLocality: Quebrada Cebollines; verbatimElevation: 270; verbatimLatitude: 10.8222; verbatimLongitude: -85.6434; verbatimCoordinateSystem: Decimal; decimalLatitude: 10.8222; decimalLongitude: -85.6434; **Identification:** identifiedBy: AJ Fleming; dateIdentified: 2017; **Event:** samplingProtocol: Reared from the larva of the Doidae, Doa Janzen01; verbatimEventDate: 07-Nov-2001; **Record Level:** language: en; institutionCode: CNC; collectionCode: Insects; basisOfRecord: Pinned Specimen
- **Type status:** Paratype. **Occurrence:** occurrenceDetails: http://janzen.sas.upenn.edu; catalogNumber: DHJPAR0007471; recordedBy: D.H. Janzen, W. Hallwachs & gusaneros; individualID: DHJPAR0007471; individualCount: 1; sex: male; lifeStage: adult; preparations: pinned; otherCatalogNumbers: ASTAT243-06, 01-SRNP-17476, BOLD:AAC5464; **Taxon:** scientificName: Hyphantrophaga edwinapui; phylum: Arthropoda; class: Insecta; order: Diptera; family: Tachinidae; genus: Hyphantrophaga; specificEpithet: edwinapui; scientificNameAuthorship: Fleming & Wood, 2017; **Location:** continent: Central America; country: Costa Rica; countryCode: CR; stateProvince: Guanacaste; county: Sector Santa Rosa; locality: Area de Conservacion Guanacaste; verbatimLocality: Quebrada Cebollines; verbatimElevation: 270; verbatimLatitude: 10.8222; verbatimLongitude: -85.6434; verbatimCoordinateSystem: Decimal; decimalLatitude: 10.8222; decimalLongitude: -85.6434; **Identification:** identifiedBy: AJ Fleming; dateIdentified: 2017; **Event:** samplingProtocol: Reared from the larva of the Doidae, Doa Janzen01; verbatimEventDate: 05-Nov-2001; **Record Level:** language: en; institutionCode: CNC; collectionCode: Insects; basisOfRecord: Pinned Specimen
- **Type status:** Paratype. **Occurrence:** occurrenceDetails: http://janzen.sas.upenn.edu; catalogNumber: DHJPAR0007467; recordedBy: D.H. Janzen, W. Hallwachs & gusaneros; individualID: DHJPAR0007467; individualCount: 1; sex: female; lifeStage: adult; preparations: pinned; otherCatalogNumbers: ASTAT239-06, 01-SRNP-17516, BOLD:AAC5464; **Taxon:** scientificName: Hyphantrophaga edwinapui; phylum: Arthropoda; class: Insecta; order: Diptera; family: Tachinidae; genus: Hyphantrophaga; specificEpithet: edwinapui; scientificNameAuthorship: Fleming & Wood, 2017; **Location:** continent: Central America; country: Costa Rica; countryCode: CR; stateProvince: Guanacaste; county: Sector Santa Rosa; locality: Area de Conservacion Guanacaste; verbatimLocality: Quebrada Cebollines; verbatimElevation: 270; verbatimLatitude: 10.8222; verbatimLongitude: -85.6434; verbatimCoordinateSystem: Decimal; decimalLatitude: 10.8222; decimalLongitude: -85.6434; **Identification:** identifiedBy: AJ Fleming; dateIdentified: 2017; **Event:** samplingProtocol: Reared from the larva of the Doidae, Doa Janzen01; verbatimEventDate: 07-Nov-2001; **Record Level:** language: en; institutionCode: CNC; collectionCode: Insects; basisOfRecord: Pinned Specimen
- **Type status:** Paratype. **Occurrence:** occurrenceDetails: http://janzen.sas.upenn.edu; catalogNumber: DHJPAR0007466; recordedBy: D.H. Janzen, W. Hallwachs & gusaneros; individualID: DHJPAR0007466; individualCount: 1; sex: female; lifeStage: adult; preparations: pinned; otherCatalogNumbers: ASTAT238-06, 01-SRNP-17484, BOLD:AAC5464; **Taxon:** scientificName: Hyphantrophaga edwinapui; phylum: Arthropoda; class: Insecta; order: Diptera; family: Tachinidae; genus: Hyphantrophaga; specificEpithet: edwinapui; scientificNameAuthorship: Fleming & Wood, 2017; **Location:** continent: Central America; country: Costa Rica; countryCode: CR; stateProvince: Guanacaste; county: Sector Santa Rosa; locality: Area de Conservacion Guanacaste; verbatimLocality: Quebrada Cebollines; verbatimElevation: 270; verbatimLatitude: 10.8222; verbatimLongitude: -85.6434; verbatimCoordinateSystem: Decimal; decimalLatitude: 10.8222; decimalLongitude: -85.6434; **Identification:** identifiedBy: AJ Fleming; dateIdentified: 2017; **Event:** samplingProtocol: Reared from the larva of the Doidae, Doa Janzen01; verbatimEventDate: 07-Nov-2001; **Record Level:** language: en; institutionCode: CNC; collectionCode: Insects; basisOfRecord: Pinned Specimen
- **Type status:** Paratype. **Occurrence:** occurrenceDetails: http://janzen.sas.upenn.edu; catalogNumber: DHJPAR0007475; recordedBy: D.H. Janzen, W. Hallwachs & gusaneros; individualID: DHJPAR0007475; individualCount: 1; sex: male; lifeStage: adult; preparations: pinned; otherCatalogNumbers: ASTAT247-06, 01-SRNP-17549,; **Taxon:** scientificName: Hyphantrophaga edwinapui; phylum: Arthropoda; class: Insecta; order: Diptera; family: Tachinidae; genus: Hyphantrophaga; specificEpithet: edwinapui; scientificNameAuthorship: Fleming & Wood, 2017; **Location:** continent: Central America; country: Costa Rica; countryCode: CR; stateProvince: Guanacaste; county: Sector Santa Rosa; locality: Area de Conservacion Guanacaste; verbatimLocality: Quebrada Cebollines; verbatimElevation: 270; verbatimLatitude: 10.8222; verbatimLongitude: -85.6434; verbatimCoordinateSystem: Decimal; decimalLatitude: 10.8222; decimalLongitude: -85.6434; **Identification:** identifiedBy: AJ Fleming; dateIdentified: 2017; **Event:** samplingProtocol: Reared from the larva of the Doidae, Doa Janzen01; verbatimEventDate: 05-Nov-2001; **Record Level:** language: en; institutionCode: CNC; collectionCode: Insects; basisOfRecord: Pinned Specimen
- **Type status:** Paratype. **Occurrence:** occurrenceDetails: http://janzen.sas.upenn.edu; catalogNumber: DHJPAR0007468; recordedBy: D.H. Janzen, W. Hallwachs & gusaneros; individualID: DHJPAR0007468; individualCount: 1; sex: female; lifeStage: adult; preparations: pinned; otherCatalogNumbers: ASTAT240-06, 01-SRNP-17495, BOLD:AAC5464; **Taxon:** scientificName: Hyphantrophaga edwinapui; phylum: Arthropoda; class: Insecta; order: Diptera; family: Tachinidae; genus: Hyphantrophaga; specificEpithet: edwinapui; scientificNameAuthorship: Fleming & Wood, 2017; **Location:** continent: Central America; country: Costa Rica; countryCode: CR; stateProvince: Guanacaste; county: Sector Santa Rosa; locality: Area de Conservacion Guanacaste; verbatimLocality: Quebrada Cebollines; verbatimElevation: 270; verbatimLatitude: 10.8222; verbatimLongitude: -85.6434; verbatimCoordinateSystem: Decimal; decimalLatitude: 10.8222; decimalLongitude: -85.6434; **Identification:** identifiedBy: AJ Fleming; dateIdentified: 2017; **Event:** samplingProtocol: Reared from the larva of the Doidae, Doa Janzen01; verbatimEventDate: 05-Nov-2001; **Record Level:** language: en; institutionCode: CNC; collectionCode: Insects; basisOfRecord: Pinned Specimen
- **Type status:** Paratype. **Occurrence:** occurrenceDetails: http://janzen.sas.upenn.edu; catalogNumber: DHJPAR0007473; recordedBy: D.H. Janzen, W. Hallwachs & gusaneros; individualID: DHJPAR0007473; individualCount: 1; sex: female; lifeStage: adult; preparations: pinned; otherCatalogNumbers: ASTAT245-06, 01-SRNP-17536, BOLD:AAC5464; **Taxon:** scientificName: Hyphantrophaga edwinapui; phylum: Arthropoda; class: Insecta; order: Diptera; family: Tachinidae; genus: Hyphantrophaga; specificEpithet: edwinapui; scientificNameAuthorship: Fleming & Wood, 2017; **Location:** continent: Central America; country: Costa Rica; countryCode: CR; stateProvince: Guanacaste; county: Sector Santa Rosa; locality: Area de Conservacion Guanacaste; verbatimLocality: Quebrada Cebollines; verbatimElevation: 270; verbatimLatitude: 10.8222; verbatimLongitude: -85.6434; verbatimCoordinateSystem: Decimal; decimalLatitude: 10.8222; decimalLongitude: -85.6434; **Identification:** identifiedBy: AJ Fleming; dateIdentified: 2017; **Event:** samplingProtocol: Reared from the larva of the Doidae, Doa Janzen01; verbatimEventDate: 07-Nov-2001; **Record Level:** language: en; institutionCode: CNC; collectionCode: Insects; basisOfRecord: Pinned Specimen
- **Type status:** Paratype. **Occurrence:** occurrenceDetails: http://janzen.sas.upenn.edu; catalogNumber: DHJPAR0007464; recordedBy: D.H. Janzen, W. Hallwachs & gusaneros; individualID: DHJPAR0007464; individualCount: 1; sex: female; lifeStage: adult; preparations: pinned; otherCatalogNumbers: ASTAT236-06, 01-SRNP-17527, BOLD:AAC5464; **Taxon:** scientificName: Hyphantrophaga edwinapui; phylum: Arthropoda; class: Insecta; order: Diptera; family: Tachinidae; genus: Hyphantrophaga; specificEpithet: edwinapui; scientificNameAuthorship: Fleming & Wood, 2017; **Location:** continent: Central America; country: Costa Rica; countryCode: CR; stateProvince: Guanacaste; county: Sector Santa Rosa; locality: Area de Conservacion Guanacaste; verbatimLocality: Quebrada Cebollines; verbatimElevation: 270; verbatimLatitude: 10.8222; verbatimLongitude: -85.6434; verbatimCoordinateSystem: Decimal; decimalLatitude: 10.8222; decimalLongitude: -85.6434; **Identification:** identifiedBy: AJ Fleming; dateIdentified: 2017; **Event:** samplingProtocol: Reared from the larva of the Doidae, Doa Janzen01; verbatimEventDate: 02-Nov-2001; **Record Level:** language: en; institutionCode: CNC; collectionCode: Insects; basisOfRecord: Pinned Specimen

#### Description

**Male** (Fig. 12). Length: 7–9 mm. **Head** (Fig. 12b): vertex 1/5 of head width; two pairs of reclinate upper orbital setae; ocellar setae arising behind anterior ocellus; ocellar triangle silver/dull grey; fronto-orbital plate dull silver/grey and densely setulose throughout, setulae not extending beyond level of lowest frontal seta; parafacial concolorous with fronto-orbital plate and bare; eye densely haired; facial ridge bare; pedicel brownish-black (with only a slight light orange fringe at margin between pedicel and postpedicel), concolorous with postpedicel; arista brown, very minutely pubescent, distinctly thickened on basal 1/3–1/4; palpus dark grey/brown, haired apically, slender-digitiform. **Thorax** (Fig. 12a, c): dull brassy tomentose throughout; densely covered on all surfaces, with short black setulae interspersed amongst the setae; four thick dorsal vittae, outermost two broken across suture, innermost pair unbroken, reaching 2nd postsutural dorsocentral seta; postpronotum with 4–5 setae arranged in a triangle; chaetotaxy: acrostichal setae 3:3; dorsocentral setae 3:4; intra-alar setae 3:3; supra-alar setae 2:3; three katepisternal setae; basal scutellar setae subequal to subapical scutellar setae, curving inwards medially; lateral scutellar setae approximately 1/2 as long as subapical setae, curving inwards medially, parallel to basal scutellar setae; subapical scutellar setae straight and strongly divergent; apical scutellar setae subequal in length to lateral scutellar setae, crossed apically; one pair of discal scutellar setae set more narrowly than subapical setae; scutellum very lightly gold tomentose across apical 20%, remainder concolorous with scutum. **Legs** (Fig. 12c): brilliant yellow in ground colour, covered in dark setulae making them appear darker; fore femur with dense silver tomentum on posterodorsal surface; hind coxa bare. **Wing** (Fig. 12a): pale translucent, hyaline; vein R_4+5_ with only 2–3 setulae at base. **Abdomen** (Fig. 12a, c): ground colour yellowish-brown; middorsal depression on ST1+2 almost reaching hind margin; median marginal setae absent on ST1+2, present on T3; a complete row of marginal setae present on T4; discal setae only on T5; sex patch covering ventral surfaces of T4–T5; entire dorsal surface of tergites distinctly brassy tomentose; T5 with brassy tomentum throughout. **Terminalia** (Fig. 12d, e, f): sternite 5 (Fig. 12f) with a deeply excavated median cleft, squared-off wide V-shaped, margins covered in dense tomentum. Lateral lobes of sternite rounded triangular apically, 1–2 strong setae surrounded by many shorter, weaker setulae. Anterior plate of sternite 5 twice as long as apical lobes; unsclerotised window anterior to median cleft only slightly tri-lobed, with arms as wide as median cleft. Cerci in posterior view (Fig. 12d) narrow, subrectangular and slightly shorter than surstyli, ending in a rounded tip; completely separate medially, straight, divergent in apical 1/3; in lateral view weakly clubbed along anterior 1/3 and weakly curved at beginning of club; densely setulose dorsally up to a tapered tip, apparently bare ventrally (visible in lateral view). Surstylus in lateral view (Fig. 12e) almost parallel-sided along its length, with rounded apices, giving it a spatulate appearance; when viewed dorsally, surstyli appearing not divergent. Pregonite broad and well-developed, slightly bent; basal 2/3 slightly cinched, giving it a very slightly clubbed appearance; apically rounded with a few fine setulae along its edge. Postgonite elongate, equally wide along its length with a slight curve at tip, subequal in length to pregonite. Distiphallus rectangular with a very slight apical flare, a slender median longitudinal sclerotised reinforcement on its posterior surface and a broad, anterolateral, sclerotised acrophallus, joined as a plate on anterior surface near apex.

**Female**. Length: 5–7 mm. As male, differing only by the presence of two pairs of proclinate orbital setae.

#### Diagnosis

*Hyphantrophaga edwinapui* **sp. n.** can be distinguished from all other *Hyphantrophaga* species by the following combination of traits: three katepisternal setae, legs yellow, hind coxa bare, abdominal tergites entirely brassy tomentose and median marginal setae absent from ST1+2.

#### Etymology

*Hyphantrophaga edwinapui* **sp. n.** is named in recognition of Edwin Jose Apu Fajardo's dedication and work in finding and rearing the ACG caterpillars that contained tachinid larvae.

#### Distribution

Costa Rica, ACG, Guanacaste Province, 270 m elevation.

#### Ecology

*Hyphantrophaga edwinapui* **sp. n.** has been reared 22 times from a single species of Lepidoptera in the family Doidae, *Doa* Janzen01, in dry forest.

### Hyphantrophaga eldaarayae

Fleming & Wood sp. n.

urn:lsid:zoobank.org:act:625985CF-3D13-4FA1-A1BC-27819EA9D933

#### Materials

- **Type status:** Holotype. **Occurrence:** occurrenceDetails: http://janzen.sas.upenn.edu; catalogNumber: DHJPAR0022963; recordedBy: D.H. Janzen, W. Hallwachs & Gloria Sihezar; individualID: DHJPAR0022963; individualCount: 1; sex: male; lifeStage: adult; preparations: pinned; otherCatalogNumbers: ASTAW127-08, 01-SRNP-3991,; **Taxon:** scientificName: Hyphantrophaga eldaarayae; phylum: Arthropoda; class: Insecta; order: Diptera; family: Tachinidae; genus: Hyphantrophaga; specificEpithet: eldaarayae; scientificNameAuthorship: Fleming & Wood, 2018; **Location:** continent: Central America; country: Costa Rica; countryCode: CR; stateProvince: Alajuela; county: Sector San Cristobal; locality: Area de Conservacion Guanacaste; verbatimLocality: Rio Blanco Abajo; verbatimElevation: 500; verbatimLatitude: 10.9004; verbatimLongitude: -85.3725; verbatimCoordinateSystem: Decimal; decimalLatitude: 10.9004; decimalLongitude: -85.3725; **Identification:** identifiedBy: AJ Fleming; dateIdentified: 2017; **Event:** samplingProtocol: Reared from the larva of the Sphingidae, Xylophanes chiron; verbatimEventDate: 28-Nov-2001; **Record Level:** language: en; institutionCode: CNC; collectionCode: Insects; basisOfRecord: Pinned Specimen
- **Type status:** Paratype. **Occurrence:** occurrenceDetails: http://janzen.sas.upenn.edu; catalogNumber: DHJPAR0005525; recordedBy: D.H. Janzen, W. Hallwachs & Carolina Cano; individualID: DHJPAR0005525; individualCount: 1; sex: male; lifeStage: adult; preparations: pinned; otherCatalogNumbers: ASTA644-06, 05-SRNP-8070,; **Taxon:** scientificName: Hyphantrophaga eldaarayae; phylum: Arthropoda; class: Insecta; order: Diptera; family: Tachinidae; genus: Hyphantrophaga; specificEpithet: eldaarayae; scientificNameAuthorship: Fleming & Wood, 2018; **Location:** continent: Central America; country: Costa Rica; countryCode: CR; stateProvince: Alajuela; county: Sector San Cristobal; locality: Area de Conservacion Guanacaste; verbatimLocality: Rio Blanco Abajo; verbatimElevation: 500; verbatimLatitude: 10.9004; verbatimLongitude: -85.3725; verbatimCoordinateSystem: Decimal; decimalLatitude: 10.9004; decimalLongitude: -85.3725; **Identification:** identifiedBy: AJ Fleming; dateIdentified: 2017; **Event:** samplingProtocol: Reared from the larva of the Sphingidae, Xylophanes chiron; verbatimEventDate: 26-Jan-2006; **Record Level:** language: en; institutionCode: CNC; collectionCode: Insects; basisOfRecord: Pinned Specimen
- **Type status:** Paratype. **Occurrence:** occurrenceDetails: http://janzen.sas.upenn.edu; catalogNumber: DHJPAR0008059; recordedBy: D.H. Janzen, W. Hallwachs & Jose Perez; individualID: DHJPAR0008059; individualCount: 1; sex: male; lifeStage: adult; preparations: pinned; otherCatalogNumbers: ASTAT831-06, 03-SRNP-11664, BOLD:AAB0592; **Taxon:** scientificName: Hyphantrophaga eldaarayae; phylum: Arthropoda; class: Insecta; order: Diptera; family: Tachinidae; genus: Hyphantrophaga; specificEpithet: eldaarayae; scientificNameAuthorship: Fleming & Wood, 2018; **Location:** continent: Central America; country: Costa Rica; countryCode: CR; stateProvince: Alajuela; county: Sector Rincon Rain Forest; locality: Area de Conservacion Guanacaste; verbatimLocality: San Lucas; verbatimElevation: 320; verbatimLatitude: 10.9185; verbatimLongitude: -85.3034; verbatimCoordinateSystem: Decimal; decimalLatitude: 10.9185; decimalLongitude: -85.3034; **Identification:** identifiedBy: AJ Fleming; dateIdentified: 2017; **Event:** samplingProtocol: Reared from the larva of the Sphingidae, Xylophanes chiron; verbatimEventDate: 02-Aug-2003; **Record Level:** language: en; institutionCode: CNC; collectionCode: Insects; basisOfRecord: Pinned Specimen
- **Type status:** Paratype. **Occurrence:** occurrenceDetails: http://janzen.sas.upenn.edu; catalogNumber: DHJPAR0008064; recordedBy: D.H. Janzen, W. Hallwachs & Jose Perez; individualID: DHJPAR0008064; individualCount: 1; sex: male; lifeStage: adult; preparations: pinned; otherCatalogNumbers: ASTAT836-06, 03-SRNP-31066,; **Taxon:** scientificName: Hyphantrophaga eldaarayae; phylum: Arthropoda; class: Insecta; order: Diptera; family: Tachinidae; genus: Hyphantrophaga; specificEpithet: eldaarayae; scientificNameAuthorship: Fleming & Wood, 2018; **Location:** continent: Central America; country: Costa Rica; countryCode: CR; stateProvince: Alajuela; county: Sector Rincon Rain Forest; locality: Area de Conservacion Guanacaste; verbatimLocality: San Lucas; verbatimElevation: 320; verbatimLatitude: 10.9185; verbatimLongitude: -85.3034; verbatimCoordinateSystem: Decimal; decimalLatitude: 10.9185; decimalLongitude: -85.3034; **Identification:** identifiedBy: AJ Fleming; dateIdentified: 2017; **Event:** samplingProtocol: Reared from the larva of the Sphingidae, Xylophanes chiron; verbatimEventDate: 14-Nov-2003; **Record Level:** language: en; institutionCode: CNC; collectionCode: Insects; basisOfRecord: Pinned Specimen
- **Type status:** Paratype. **Occurrence:** occurrenceDetails: http://janzen.sas.upenn.edu; catalogNumber: DHJPAR0008066; recordedBy: D.H. Janzen, W. Hallwachs & Gloria Sihezar; individualID: DHJPAR0008066; individualCount: 1; sex: male; lifeStage: adult; preparations: pinned; otherCatalogNumbers: ASTAT838-06, 98-SRNP-6070,; **Taxon:** scientificName: Hyphantrophaga eldaarayae; phylum: Arthropoda; class: Insecta; order: Diptera; family: Tachinidae; genus: Hyphantrophaga; specificEpithet: eldaarayae; scientificNameAuthorship: Fleming & Wood, 2018; **Location:** continent: Central America; country: Costa Rica; countryCode: CR; stateProvince: Alajuela; county: Sector San Cristobal; locality: Area de Conservacion Guanacaste; verbatimLocality: Quebrada Cementerio; verbatimElevation: 700; verbatimLatitude: 10.8712; verbatimLongitude: -85.3875; verbatimCoordinateSystem: Decimal; decimalLatitude: 10.8712; decimalLongitude: -85.3875; **Identification:** identifiedBy: AJ Fleming; dateIdentified: 2017; **Event:** samplingProtocol: Reared from the larva of the Sphingidae, Xylophanes chiron; verbatimEventDate: 02-Jul-1998; **Record Level:** language: en; institutionCode: CNC; collectionCode: Insects; basisOfRecord: Pinned Specimen
- **Type status:** Paratype. **Occurrence:** occurrenceDetails: http://janzen.sas.upenn.edu; catalogNumber: DHJPAR0008069; recordedBy: D.H. Janzen, W. Hallwachs & Lucia Rios; individualID: DHJPAR0008069; individualCount: 1; sex: male; lifeStage: adult; preparations: pinned; otherCatalogNumbers: ASTAT841-06, 03-SRNP-37359, BOLD:AAB0592; **Taxon:** scientificName: Hyphantrophaga eldaarayae; phylum: Arthropoda; class: Insecta; order: Diptera; family: Tachinidae; genus: Hyphantrophaga; specificEpithet: eldaarayae; scientificNameAuthorship: Fleming & Wood, 2018; **Location:** continent: Central America; country: Costa Rica; countryCode: CR; stateProvince: Guanacaste; county: Sector Pitilla; locality: Area de Conservacion Guanacaste; verbatimLocality: Estacion Pitilla; verbatimElevation: 675; verbatimLatitude: 10.9893; verbatimLongitude: -85.4258; verbatimCoordinateSystem: Decimal; decimalLatitude: 10.9893; decimalLongitude: -85.4258; **Identification:** identifiedBy: AJ Fleming; dateIdentified: 2017; **Event:** samplingProtocol: Reared from the larva of the Sphingidae, Xylophanes chiron; verbatimEventDate: 16-Jan-2004; **Record Level:** language: en; institutionCode: CNC; collectionCode: Insects; basisOfRecord: Pinned Specimen
- **Type status:** Paratype. **Occurrence:** occurrenceDetails: http://janzen.sas.upenn.edu; catalogNumber: DHJPAR0008075; recordedBy: D.H. Janzen, W. Hallwachs & Calixto Moraga; individualID: DHJPAR0008075; individualCount: 1; sex: male; lifeStage: adult; preparations: pinned; otherCatalogNumbers: ASTAT847-06, 04-SRNP-56432, BOLD:AAB0592; **Taxon:** scientificName: Hyphantrophaga eldaarayae; phylum: Arthropoda; class: Insecta; order: Diptera; family: Tachinidae; genus: Hyphantrophaga; specificEpithet: eldaarayae; scientificNameAuthorship: Fleming & Wood, 2018; **Location:** continent: Central America; country: Costa Rica; countryCode: CR; stateProvince: Guanacaste; county: Sector Pitilla; locality: Area de Conservacion Guanacaste; verbatimLocality: Pasmompa; verbatimElevation: 440; verbatimLatitude: 11.0193; verbatimLongitude: -85.41; verbatimCoordinateSystem: Decimal; decimalLatitude: 11.0193; decimalLongitude: -85.41; **Identification:** identifiedBy: AJ Fleming; dateIdentified: 2017; **Event:** samplingProtocol: Reared from the larva of the Sphingidae, Xylophanes chiron; verbatimEventDate: 26-Dec-2004; **Record Level:** language: en; institutionCode: CNC; collectionCode: Insects; basisOfRecord: Pinned Specimen
- **Type status:** Paratype. **Occurrence:** occurrenceDetails: http://janzen.sas.upenn.edu; catalogNumber: DHJPAR0008080; recordedBy: D.H. Janzen, W. Hallwachs & Guillermo Pereira; individualID: DHJPAR0008080; individualCount: 1; sex: male; lifeStage: adult; preparations: pinned; otherCatalogNumbers: ASTAT852-06, 03-SRNP-14320,; **Taxon:** scientificName: Hyphantrophaga eldaarayae; phylum: Arthropoda; class: Insecta; order: Diptera; family: Tachinidae; genus: Hyphantrophaga; specificEpithet: eldaarayae; scientificNameAuthorship: Fleming & Wood, 2018; **Location:** continent: Central America; country: Costa Rica; countryCode: CR; stateProvince: Guanacaste; county: Sector Santa Rosa; locality: Area de Conservacion Guanacaste; verbatimLocality: Sendero Inga Peluda; verbatimElevation: 280; verbatimLatitude: 10.8733; verbatimLongitude: -85.6067; verbatimCoordinateSystem: Decimal; decimalLatitude: 10.8733; decimalLongitude: -85.6067; **Identification:** identifiedBy: AJ Fleming; dateIdentified: 2017; **Event:** samplingProtocol: Reared from the larva of the Sphingidae, Xylophanes chiron; verbatimEventDate: 18-Aug-2003; **Record Level:** language: en; institutionCode: CNC; collectionCode: Insects; basisOfRecord: Pinned Specimen
- **Type status:** Paratype. **Occurrence:** occurrenceDetails: http://janzen.sas.upenn.edu; catalogNumber: DHJPAR0008081; recordedBy: D.H. Janzen, W. Hallwachs & Petrona Rios; individualID: DHJPAR0008081; individualCount: 1; sex: male; lifeStage: adult; preparations: pinned; otherCatalogNumbers: ASTAT853-06, 03-SRNP-20030,; **Taxon:** scientificName: Hyphantrophaga eldaarayae; phylum: Arthropoda; class: Insecta; order: Diptera; family: Tachinidae; genus: Hyphantrophaga; specificEpithet: eldaarayae; scientificNameAuthorship: Fleming & Wood, 2018; **Location:** continent: Central America; country: Costa Rica; countryCode: CR; stateProvince: Guanacaste; county: Sector Pitilla; locality: Area de Conservacion Guanacaste; verbatimLocality: Pasmompa; verbatimElevation: 440; verbatimLatitude: 11.0193; verbatimLongitude: -85.41; verbatimCoordinateSystem: Decimal; decimalLatitude: 11.0193; decimalLongitude: -85.41; **Identification:** identifiedBy: AJ Fleming; dateIdentified: 2017; **Event:** samplingProtocol: Reared from the larva of the Sphingidae, Xylophanes chiron; verbatimEventDate: 04-Aug-2003; **Record Level:** language: en; institutionCode: CNC; collectionCode: Insects; basisOfRecord: Pinned Specimen
- **Type status:** Paratype. **Occurrence:** occurrenceDetails: http://janzen.sas.upenn.edu; catalogNumber: DHJPAR0008082; recordedBy: D.H. Janzen, W. Hallwachs & Anabelle Cordoba; individualID: DHJPAR0008082; individualCount: 1; sex: male; lifeStage: adult; preparations: pinned; otherCatalogNumbers: ASTAT854-06, 05-SRNP-5411, BOLD:AAB0592; **Taxon:** scientificName: Hyphantrophaga eldaarayae; phylum: Arthropoda; class: Insecta; order: Diptera; family: Tachinidae; genus: Hyphantrophaga; specificEpithet: eldaarayae; scientificNameAuthorship: Fleming & Wood, 2018; **Location:** continent: Central America; country: Costa Rica; countryCode: CR; stateProvince: Alajuela; county: Sector San Cristobal; locality: Area de Conservacion Guanacaste; verbatimLocality: Rio Blanco Abajo; verbatimElevation: 500; verbatimLatitude: 10.9004; verbatimLongitude: -85.3725; verbatimCoordinateSystem: Decimal; decimalLatitude: 10.9004; decimalLongitude: -85.3725; **Identification:** identifiedBy: AJ Fleming; dateIdentified: 2017; **Event:** samplingProtocol: Reared from the larva of the Sphingidae, Xylophanes chiron; verbatimEventDate: 03-Oct-2005; **Record Level:** language: en; institutionCode: CNC; collectionCode: Insects; basisOfRecord: Pinned Specimen
- **Type status:** Paratype. **Occurrence:** occurrenceDetails: http://janzen.sas.upenn.edu; catalogNumber: DHJPAR0008083; recordedBy: D.H. Janzen, W. Hallwachs & Petrona Rios; individualID: DHJPAR0008083; individualCount: 1; sex: male; lifeStage: adult; preparations: pinned; otherCatalogNumbers: ASTAT855-06, 04-SRNP-33892, BOLD:AAB0592; **Taxon:** scientificName: Hyphantrophaga eldaarayae; phylum: Arthropoda; class: Insecta; order: Diptera; family: Tachinidae; genus: Hyphantrophaga; specificEpithet: eldaarayae; scientificNameAuthorship: Fleming & Wood, 2018; **Location:** continent: Central America; country: Costa Rica; countryCode: CR; stateProvince: Guanacaste; county: Sector Pitilla; locality: Area de Conservacion Guanacaste; verbatimLocality: Pasmompa; verbatimElevation: 440; verbatimLatitude: 11.0193; verbatimLongitude: -85.41; verbatimCoordinateSystem: Decimal; decimalLatitude: 11.0193; decimalLongitude: -85.41; **Identification:** identifiedBy: AJ Fleming; dateIdentified: 2017; **Event:** samplingProtocol: Reared from the larva of the Sphingidae, Xylophanes chiron; verbatimEventDate: 20-Aug-2004; **Record Level:** language: en; institutionCode: CNC; collectionCode: Insects; basisOfRecord: Pinned Specimen
- **Type status:** Paratype. **Occurrence:** occurrenceDetails: http://janzen.sas.upenn.edu; catalogNumber: DHJPAR0008060; recordedBy: D.H. Janzen, W. Hallwachs & Carolina Cano; individualID: DHJPAR0008060; individualCount: 1; sex: female; lifeStage: adult; preparations: pinned; otherCatalogNumbers: ASTAT832-06, 03-SRNP-8261,; **Taxon:** scientificName: Hyphantrophaga eldaarayae; phylum: Arthropoda; class: Insecta; order: Diptera; family: Tachinidae; genus: Hyphantrophaga; specificEpithet: eldaarayae; scientificNameAuthorship: Fleming & Wood, 2018; **Location:** continent: Central America; country: Costa Rica; countryCode: CR; stateProvince: Alajuela; county: Sector San Cristobal; locality: Area de Conservacion Guanacaste; verbatimLocality: Rio Blanco Abajo; verbatimElevation: 500; verbatimLatitude: 10.9004; verbatimLongitude: -85.3725; verbatimCoordinateSystem: Decimal; decimalLatitude: 10.9004; decimalLongitude: -85.3725; **Identification:** identifiedBy: AJ Fleming; dateIdentified: 2017; **Event:** samplingProtocol: Reared from the larva of the Sphingidae, Xylophanes chiron; verbatimEventDate: 05-Oct-2003; **Record Level:** language: en; institutionCode: CNC; collectionCode: Insects; basisOfRecord: Pinned Specimen
- **Type status:** Paratype. **Occurrence:** occurrenceDetails: http://janzen.sas.upenn.edu; catalogNumber: DHJPAR0008062; recordedBy: D.H. Janzen, W. Hallwachs & Osvaldo Espinoza; individualID: DHJPAR0008062; individualCount: 1; sex: female; lifeStage: adult; preparations: pinned; otherCatalogNumbers: ASTAT834-06, 01-SRNP-22084,; **Taxon:** scientificName: Hyphantrophaga eldaarayae; phylum: Arthropoda; class: Insecta; order: Diptera; family: Tachinidae; genus: Hyphantrophaga; specificEpithet: eldaarayae; scientificNameAuthorship: Fleming & Wood, 2018; **Location:** continent: Central America; country: Costa Rica; countryCode: CR; stateProvince: Alajuela; county: Sector San Cristobal; locality: Area de Conservacion Guanacaste; verbatimLocality: Rio Blanco Abajo; verbatimElevation: 500; verbatimLatitude: 10.9004; verbatimLongitude: -85.3725; verbatimCoordinateSystem: Decimal; decimalLatitude: 10.9004; decimalLongitude: -85.3725; **Identification:** identifiedBy: AJ Fleming; dateIdentified: 2017; **Event:** samplingProtocol: Reared from the larva of the Sphingidae, Xylophanes chiron; verbatimEventDate: 13-Nov-2001; **Record Level:** language: en; institutionCode: CNC; collectionCode: Insects; basisOfRecord: Pinned Specimen
- **Type status:** Paratype. **Occurrence:** occurrenceDetails: http://janzen.sas.upenn.edu; catalogNumber: DHJPAR0008063; recordedBy: D.H. Janzen, W. Hallwachs & Lucia Rios; individualID: DHJPAR0008063; individualCount: 1; sex: female; lifeStage: adult; preparations: pinned; otherCatalogNumbers: ASTAT835-06, 01-SRNP-10457,; **Taxon:** scientificName: Hyphantrophaga eldaarayae; phylum: Arthropoda; class: Insecta; order: Diptera; family: Tachinidae; genus: Hyphantrophaga; specificEpithet: eldaarayae; scientificNameAuthorship: Fleming & Wood, 2018; **Location:** continent: Central America; country: Costa Rica; countryCode: CR; stateProvince: Guanacaste; county: Sector El Hacha; locality: Area de Conservacion Guanacaste; verbatimLocality: Sendero Tigre; verbatimElevation: 280; verbatimLatitude: 11.0317; verbatimLongitude: -85.5262; verbatimCoordinateSystem: Decimal; decimalLatitude: 11.0317; decimalLongitude: -85.5262; **Identification:** identifiedBy: AJ Fleming; dateIdentified: 2017; **Event:** samplingProtocol: Reared from the larva of the Sphingidae, Xylophanes pluto; verbatimEventDate: 04-Sep-2001; **Record Level:** language: en; institutionCode: CNC; collectionCode: Insects; basisOfRecord: Pinned Specimen
- **Type status:** Paratype. **Occurrence:** occurrenceDetails: http://janzen.sas.upenn.edu; catalogNumber: DHJPAR0008065; recordedBy: D.H. Janzen, W. Hallwachs & Gloria Sihezar; individualID: DHJPAR0008065; individualCount: 1; sex: female; lifeStage: adult; preparations: pinned; otherCatalogNumbers: ASTAT837-06, 02-SRNP-2965, BOLD:AAB0592; **Taxon:** scientificName: Hyphantrophaga eldaarayae; phylum: Arthropoda; class: Insecta; order: Diptera; family: Tachinidae; genus: Hyphantrophaga; specificEpithet: eldaarayae; scientificNameAuthorship: Fleming & Wood, 2018; **Location:** continent: Central America; country: Costa Rica; countryCode: CR; stateProvince: Alajuela; county: Sector San Cristobal; locality: Area de Conservacion Guanacaste; verbatimLocality: Rio Blanco Abajo; verbatimElevation: 500; verbatimLatitude: 10.9004; verbatimLongitude: -85.3725; verbatimCoordinateSystem: Decimal; decimalLatitude: 10.9004; decimalLongitude: -85.3725; **Identification:** identifiedBy: AJ Fleming; dateIdentified: 2017; **Event:** samplingProtocol: Reared from the larva of the Sphingidae, Xylophanes chiron; verbatimEventDate: 02-Jun-2002; **Record Level:** language: en; institutionCode: CNC; collectionCode: Insects; basisOfRecord: Pinned Specimen
- **Type status:** Paratype. **Occurrence:** occurrenceDetails: http://janzen.sas.upenn.edu; catalogNumber: DHJPAR0008067; recordedBy: D.H. Janzen, W. Hallwachs & gusaneros; individualID: DHJPAR0008067; individualCount: 1; sex: female; lifeStage: adult; preparations: pinned; otherCatalogNumbers: ASTAT839-06, 98-SRNP-12655,; **Taxon:** scientificName: Hyphantrophaga eldaarayae; phylum: Arthropoda; class: Insecta; order: Diptera; family: Tachinidae; genus: Hyphantrophaga; specificEpithet: eldaarayae; scientificNameAuthorship: Fleming & Wood, 2018; **Location:** continent: Central America; country: Costa Rica; countryCode: CR; stateProvince: Guanacaste; county: Sector Santa Rosa; locality: Area de Conservacion Guanacaste; verbatimLocality: Ojochal; verbatimElevation: 10; verbatimLatitude: 10.7851; verbatimLongitude: -85.6637; verbatimCoordinateSystem: Decimal; decimalLatitude: 10.7851; decimalLongitude: -85.6637; **Identification:** identifiedBy: AJ Fleming; dateIdentified: 2017; **Event:** samplingProtocol: Reared from the larva of the Sphingidae, Xylophanes pluto; verbatimEventDate: 23-Nov-1998; **Record Level:** language: en; institutionCode: CNC; collectionCode: Insects; basisOfRecord: Pinned Specimen
- **Type status:** Paratype. **Occurrence:** occurrenceDetails: http://janzen.sas.upenn.edu; catalogNumber: DHJPAR0008068; recordedBy: D.H. Janzen, W. Hallwachs & Lucia Rios; individualID: DHJPAR0008068; individualCount: 1; sex: female; lifeStage: adult; preparations: pinned; otherCatalogNumbers: ASTAT840-06, 98-SRNP-13992,; **Taxon:** scientificName: Hyphantrophaga eldaarayae; phylum: Arthropoda; class: Insecta; order: Diptera; family: Tachinidae; genus: Hyphantrophaga; specificEpithet: eldaarayae; scientificNameAuthorship: Fleming & Wood, 2018; **Location:** continent: Central America; country: Costa Rica; countryCode: CR; stateProvince: Guanacaste; county: Sector El Hacha; locality: Area de Conservacion Guanacaste; verbatimLocality: Sendero Bejuquilla; verbatimElevation: 280; verbatimLatitude: 11.03; verbatimLongitude: -85.527; verbatimCoordinateSystem: Decimal; decimalLatitude: 11.03; decimalLongitude: -85.527; **Identification:** identifiedBy: AJ Fleming; dateIdentified: 2017; **Event:** samplingProtocol: Reared from the larva of the Sphingidae, Xylophanes tyndarus; verbatimEventDate: 23-Dec-1998; **Record Level:** language: en; institutionCode: CNC; collectionCode: Insects; basisOfRecord: Pinned Specimen
- **Type status:** Paratype. **Occurrence:** occurrenceDetails: http://janzen.sas.upenn.edu; catalogNumber: DHJPAR0008070; recordedBy: D.H. Janzen, W. Hallwachs & Calixto Moraga; individualID: DHJPAR0008070; individualCount: 1; sex: female; lifeStage: adult; preparations: pinned; otherCatalogNumbers: ASTAT842-06, 03-SRNP-20157,; **Taxon:** scientificName: Hyphantrophaga eldaarayae; phylum: Arthropoda; class: Insecta; order: Diptera; family: Tachinidae; genus: Hyphantrophaga; specificEpithet: eldaarayae; scientificNameAuthorship: Fleming & Wood, 2018; **Location:** continent: Central America; country: Costa Rica; countryCode: CR; stateProvince: Guanacaste; county: Sector Pitilla; locality: Area de Conservacion Guanacaste; verbatimLocality: Pasmompa; verbatimElevation: 440; verbatimLatitude: 11.0193; verbatimLongitude: -85.41; verbatimCoordinateSystem: Decimal; decimalLatitude: 11.0193; decimalLongitude: -85.41; **Identification:** identifiedBy: AJ Fleming; dateIdentified: 2017; **Event:** samplingProtocol: Reared from the larva of the Sphingidae, Xylophanes chiron; verbatimEventDate: 11-Sep-2003; **Record Level:** language: en; institutionCode: CNC; collectionCode: Insects; basisOfRecord: Pinned Specimen
- **Type status:** Paratype. **Occurrence:** occurrenceDetails: http://janzen.sas.upenn.edu; catalogNumber: DHJPAR0008071; recordedBy: D.H. Janzen, W. Hallwachs & Jose Perez; individualID: DHJPAR0008071; individualCount: 1; sex: female; lifeStage: adult; preparations: pinned; otherCatalogNumbers: ASTAT843-06, 03-SRNP-31066, BOLD:AAB0592; **Taxon:** scientificName: Hyphantrophaga eldaarayae; phylum: Arthropoda; class: Insecta; order: Diptera; family: Tachinidae; genus: Hyphantrophaga; specificEpithet: eldaarayae; scientificNameAuthorship: Fleming & Wood, 2018; **Location:** continent: Central America; country: Costa Rica; countryCode: CR; stateProvince: Alajuela; county: Sector Rincon Rain Forest; locality: Area de Conservacion Guanacaste; verbatimLocality: San Lucas; verbatimElevation: 320; verbatimLatitude: 10.9185; verbatimLongitude: -85.3034; verbatimCoordinateSystem: Decimal; decimalLatitude: 10.9185; decimalLongitude: -85.3034; **Identification:** identifiedBy: AJ Fleming; dateIdentified: 2017; **Event:** samplingProtocol: Reared from the larva of the Sphingidae, Xylophanes chiron; verbatimEventDate: 14-Nov-2003; **Record Level:** language: en; institutionCode: CNC; collectionCode: Insects; basisOfRecord: Pinned Specimen
- **Type status:** Paratype. **Occurrence:** occurrenceDetails: http://janzen.sas.upenn.edu; catalogNumber: DHJPAR0008072; recordedBy: D.H. Janzen, W. Hallwachs & Carolina Cano; individualID: DHJPAR0008072; individualCount: 1; sex: female; lifeStage: adult; preparations: pinned; otherCatalogNumbers: ASTAT844-06, 02-SRNP-19190,; **Taxon:** scientificName: Hyphantrophaga eldaarayae; phylum: Arthropoda; class: Insecta; order: Diptera; family: Tachinidae; genus: Hyphantrophaga; specificEpithet: eldaarayae; scientificNameAuthorship: Fleming & Wood, 2018; **Location:** continent: Central America; country: Costa Rica; countryCode: CR; stateProvince: Alajuela; county: Sector San Cristobal; locality: Area de Conservacion Guanacaste; verbatimLocality: Rio Blanco Abajo; verbatimElevation: 500; verbatimLatitude: 10.9004; verbatimLongitude: -85.3725; verbatimCoordinateSystem: Decimal; decimalLatitude: 10.9004; decimalLongitude: -85.3725; **Identification:** identifiedBy: AJ Fleming; dateIdentified: 2017; **Event:** samplingProtocol: Reared from the larva of the Sphingidae, Xylophanes chiron; verbatimEventDate: 14-Oct-2002; **Record Level:** language: en; institutionCode: CNC; collectionCode: Insects; basisOfRecord: Pinned Specimen
- **Type status:** Paratype. **Occurrence:** occurrenceDetails: http://janzen.sas.upenn.edu; catalogNumber: DHJPAR0008073; recordedBy: D.H. Janzen, W. Hallwachs & Guillermo Pereira; individualID: DHJPAR0008073; individualCount: 1; sex: female; lifeStage: adult; preparations: pinned; otherCatalogNumbers: ASTAT845-06, 03-SRNP-14322,; **Taxon:** scientificName: Hyphantrophaga eldaarayae; phylum: Arthropoda; class: Insecta; order: Diptera; family: Tachinidae; genus: Hyphantrophaga; specificEpithet: eldaarayae; scientificNameAuthorship: Fleming & Wood, 2018; **Location:** continent: Central America; country: Costa Rica; countryCode: CR; stateProvince: Guanacaste; county: Sector Santa Rosa; locality: Area de Conservacion Guanacaste; verbatimLocality: Sendero Inga Peluda; verbatimElevation: 280; verbatimLatitude: 10.8733; verbatimLongitude: -85.6067; verbatimCoordinateSystem: Decimal; decimalLatitude: 10.8733; decimalLongitude: -85.6067; **Identification:** identifiedBy: AJ Fleming; dateIdentified: 2017; **Event:** samplingProtocol: Reared from the larva of the Sphingidae, Xylophanes chiron; verbatimEventDate: 10-Aug-2003; **Record Level:** language: en; institutionCode: CNC; collectionCode: Insects; basisOfRecord: Pinned Specimen
- **Type status:** Paratype. **Occurrence:** occurrenceDetails: http://janzen.sas.upenn.edu; catalogNumber: DHJPAR0008074; recordedBy: D.H. Janzen, W. Hallwachs & Mariano Pereira; individualID: DHJPAR0008074; individualCount: 1; sex: female; lifeStage: adult; preparations: pinned; otherCatalogNumbers: ASTAT846-06, 03-SRNP-14331,; **Taxon:** scientificName: Hyphantrophaga eldaarayae; phylum: Arthropoda; class: Insecta; order: Diptera; family: Tachinidae; genus: Hyphantrophaga; specificEpithet: eldaarayae; scientificNameAuthorship: Fleming & Wood, 2018; **Location:** continent: Central America; country: Costa Rica; countryCode: CR; stateProvince: Guanacaste; county: Sector Santa Rosa; locality: Area de Conservacion Guanacaste; verbatimLocality: Sendero Inga Peluda; verbatimElevation: 280; verbatimLatitude: 10.8733; verbatimLongitude: -85.6067; verbatimCoordinateSystem: Decimal; decimalLatitude: 10.8733; decimalLongitude: -85.6067; **Identification:** identifiedBy: AJ Fleming; dateIdentified: 2017; **Event:** samplingProtocol: Reared from the larva of the Sphingidae, Xylophanes chiron; verbatimEventDate: 10-Aug-2003; **Record Level:** language: en; institutionCode: CNC; collectionCode: Insects; basisOfRecord: Pinned Specimen
- **Type status:** Paratype. **Occurrence:** occurrenceDetails: http://janzen.sas.upenn.edu; catalogNumber: DHJPAR0008076; recordedBy: D.H. Janzen, W. Hallwachs & Guillermo Pereira; individualID: DHJPAR0008076; individualCount: 1; sex: female; lifeStage: adult; preparations: pinned; otherCatalogNumbers: ASTAT848-06, 03-SRNP-14312,; **Taxon:** scientificName: Hyphantrophaga eldaarayae; phylum: Arthropoda; class: Insecta; order: Diptera; family: Tachinidae; genus: Hyphantrophaga; specificEpithet: eldaarayae; scientificNameAuthorship: Fleming & Wood, 2018; **Location:** continent: Central America; country: Costa Rica; countryCode: CR; stateProvince: Guanacaste; county: Sector Santa Rosa; locality: Area de Conservacion Guanacaste; verbatimLocality: Sendero Inga Peluda; verbatimElevation: 280; verbatimLatitude: 10.8733; verbatimLongitude: -85.6067; verbatimCoordinateSystem: Decimal; decimalLatitude: 10.8733; decimalLongitude: -85.6067; **Identification:** identifiedBy: AJ Fleming; dateIdentified: 2017; **Event:** samplingProtocol: Reared from the larva of the Sphingidae, Xylophanes chiron; verbatimEventDate: 06-Aug-2003; **Record Level:** language: en; institutionCode: CNC; collectionCode: Insects; basisOfRecord: Pinned Specimen
- **Type status:** Paratype. **Occurrence:** occurrenceDetails: http://janzen.sas.upenn.edu; catalogNumber: DHJPAR0008077; recordedBy: D.H. Janzen, W. Hallwachs & Elda Araya; individualID: DHJPAR0008077; individualCount: 1; sex: female; lifeStage: adult; preparations: pinned; otherCatalogNumbers: ASTAT849-06, 04-SRNP-2696, BOLD:AAB0592; **Taxon:** scientificName: Hyphantrophaga eldaarayae; phylum: Arthropoda; class: Insecta; order: Diptera; family: Tachinidae; genus: Hyphantrophaga; specificEpithet: eldaarayae; scientificNameAuthorship: Fleming & Wood, 2018; **Location:** continent: Central America; country: Costa Rica; countryCode: CR; stateProvince: Alajuela; county: Sector San Cristobal; locality: Area de Conservacion Guanacaste; verbatimLocality: Sendero Palo Alto; verbatimElevation: 570; verbatimLatitude: 10.8819; verbatimLongitude: -85.3822; verbatimCoordinateSystem: Decimal; decimalLatitude: 10.8819; decimalLongitude: -85.3822; **Identification:** identifiedBy: AJ Fleming; dateIdentified: 2017; **Event:** samplingProtocol: Reared from the larva of the Sphingidae, Xylophanes chiron; verbatimEventDate: 11-Jul-2004; **Record Level:** language: en; institutionCode: CNC; collectionCode: Insects; basisOfRecord: Pinned Specimen
- **Type status:** Other material. **Occurrence:** occurrenceDetails: http://janzen.sas.upenn.edu; catalogNumber: DHJPAR0008086; recordedBy: D.H. Janzen, W. Hallwachs & Calixto Moraga; individualID: DHJPAR0008086; individualCount: 1; sex: male; lifeStage: adult; preparations: pinned; otherCatalogNumbers: ASTAT858-06, 04-SRNP-33860, BOLD:AAB0592; **Taxon:** scientificName: Hyphantrophaga eldaarayae; phylum: Arthropoda; class: Insecta; order: Diptera; family: Tachinidae; genus: Hyphantrophaga; specificEpithet: eldaarayae; scientificNameAuthorship: Fleming & Wood, 2018; **Location:** continent: Central America; country: Costa Rica; countryCode: CR; stateProvince: Guanacaste; county: Sector Pitilla; locality: Area de Conservacion Guanacaste; verbatimLocality: Estacion Pitilla; verbatimElevation: 675; verbatimLatitude: 10.9893; verbatimLongitude: -85.4258; verbatimCoordinateSystem: Decimal; decimalLatitude: 10.9893; decimalLongitude: -85.4258; **Identification:** identifiedBy: AJ Fleming; dateIdentified: 2017; **Event:** samplingProtocol: Reared from the larva of the Sphingidae, Xylophanes chiron; verbatimEventDate: 15-Aug-2004; **Record Level:** language: en; institutionCode: CNC; collectionCode: Insects; basisOfRecord: Pinned Specimen
- **Type status:** Other material. **Occurrence:** occurrenceDetails: http://janzen.sas.upenn.edu; catalogNumber: DHJPAR0008087; recordedBy: D.H. Janzen, W. Hallwachs & Elda Araya; individualID: DHJPAR0008087; individualCount: 1; sex: male; lifeStage: adult; preparations: pinned; otherCatalogNumbers: ASTAT859-06, 04-SRNP-4398,; **Taxon:** scientificName: Hyphantrophaga eldaarayae; phylum: Arthropoda; class: Insecta; order: Diptera; family: Tachinidae; genus: Hyphantrophaga; specificEpithet: eldaarayae; scientificNameAuthorship: Fleming & Wood, 2018; **Location:** continent: Central America; country: Costa Rica; countryCode: CR; stateProvince: Alajuela; county: Sector San Cristobal; locality: Area de Conservacion Guanacaste; verbatimLocality: Finca San Gabriel; verbatimElevation: 645; verbatimLatitude: 10.8777; verbatimLongitude: -85.3934; verbatimCoordinateSystem: Decimal; decimalLatitude: 10.8777; decimalLongitude: -85.3934; **Identification:** identifiedBy: AJ Fleming; dateIdentified: 2017; **Event:** samplingProtocol: Reared from the larva of the Sphingidae, Xylophanes chiron; verbatimEventDate: 24-Sep-2004; **Record Level:** language: en; institutionCode: CNC; collectionCode: Insects; basisOfRecord: Pinned Specimen
- **Type status:** Other material. **Occurrence:** occurrenceDetails: http://janzen.sas.upenn.edu; catalogNumber: DHJPAR0008088; recordedBy: D.H. Janzen, W. Hallwachs & Lucia Rios; individualID: DHJPAR0008088; individualCount: 1; sex: male; lifeStage: adult; preparations: pinned; otherCatalogNumbers: ASTAT860-06, 00-SRNP-2879.1,; **Taxon:** scientificName: Hyphantrophaga eldaarayae; phylum: Arthropoda; class: Insecta; order: Diptera; family: Tachinidae; genus: Hyphantrophaga; specificEpithet: eldaarayae; scientificNameAuthorship: Fleming & Wood, 2018; **Location:** continent: Central America; country: Costa Rica; countryCode: CR; stateProvince: Guanacaste; county: Sector El Hacha; locality: Area de Conservacion Guanacaste; verbatimLocality: Sendero Tigre; verbatimElevation: 280; verbatimLatitude: 11.0317; verbatimLongitude: -85.5262; verbatimCoordinateSystem: Decimal; decimalLatitude: 11.0317; decimalLongitude: -85.5262; **Identification:** identifiedBy: AJ Fleming; dateIdentified: 2017; **Event:** samplingProtocol: Reared from the larva of the Sphingidae, Xylophanes tyndarus; verbatimEventDate: 09-Oct-2000; **Record Level:** language: en; institutionCode: CNC; collectionCode: Insects; basisOfRecord: Pinned Specimen
- **Type status:** Other material. **Occurrence:** occurrenceDetails: http://janzen.sas.upenn.edu; catalogNumber: DHJPAR0008091; recordedBy: D.H. Janzen, W. Hallwachs & gusaneros; individualID: DHJPAR0008091; individualCount: 1; sex: male; lifeStage: adult; preparations: pinned; otherCatalogNumbers: ASTAT863-06, 98-SRNP-12367, BOLD:AAB0592; **Taxon:** scientificName: Hyphantrophaga eldaarayae; phylum: Arthropoda; class: Insecta; order: Diptera; family: Tachinidae; genus: Hyphantrophaga; specificEpithet: eldaarayae; scientificNameAuthorship: Fleming & Wood, 2018; **Location:** continent: Central America; country: Costa Rica; countryCode: CR; stateProvince: Guanacaste; county: Sector Santa Rosa; locality: Area de Conservacion Guanacaste; verbatimLocality: Bosque Interseccion; verbatimElevation: 15; verbatimLatitude: 10.8005; verbatimLongitude: -85.6492; verbatimCoordinateSystem: Decimal; decimalLatitude: 10.8005; decimalLongitude: -85.6492; **Identification:** identifiedBy: AJ Fleming; dateIdentified: 2017; **Event:** samplingProtocol: Reared from the larva of the Sphingidae, Xylophanes pluto; verbatimEventDate: 22-Nov-1998; **Record Level:** language: en; institutionCode: CNC; collectionCode: Insects; basisOfRecord: Pinned Specimen
- **Type status:** Other material. **Occurrence:** occurrenceDetails: http://janzen.sas.upenn.edu; catalogNumber: DHJPAR0008094; recordedBy: D.H. Janzen, W. Hallwachs & Mariano Pereira; individualID: DHJPAR0008094; individualCount: 1; sex: male; lifeStage: adult; preparations: pinned; otherCatalogNumbers: ASTAT866-06, 97-SRNP-4527, BOLD:AAB0592; **Taxon:** scientificName: Hyphantrophaga eldaarayae; phylum: Arthropoda; class: Insecta; order: Diptera; family: Tachinidae; genus: Hyphantrophaga; specificEpithet: eldaarayae; scientificNameAuthorship: Fleming & Wood, 2018; **Location:** continent: Central America; country: Costa Rica; countryCode: CR; stateProvince: Guanacaste; county: Sector El Hacha; locality: Area de Conservacion Guanacaste; verbatimLocality: Sendero Tigre; verbatimElevation: 280; verbatimLatitude: 11.0317; verbatimLongitude: -85.5262; verbatimCoordinateSystem: Decimal; decimalLatitude: 11.0317; decimalLongitude: -85.5262; **Identification:** identifiedBy: AJ Fleming; dateIdentified: 2017; **Event:** samplingProtocol: Reared from the larva of the Sphingidae, Xylophanes chiron; verbatimEventDate: 23-Jul-1997; **Record Level:** language: en; institutionCode: CNC; collectionCode: Insects; basisOfRecord: Pinned Specimen
- **Type status:** Other material. **Occurrence:** occurrenceDetails: http://janzen.sas.upenn.edu; catalogNumber: DHJPAR0008096; recordedBy: D.H. Janzen, W. Hallwachs & Elieth Cantillano; individualID: DHJPAR0008096; individualCount: 1; sex: male; lifeStage: adult; preparations: pinned; otherCatalogNumbers: ASTAT868-06, 00-SRNP-4448,; **Taxon:** scientificName: Hyphantrophaga eldaarayae; phylum: Arthropoda; class: Insecta; order: Diptera; family: Tachinidae; genus: Hyphantrophaga; specificEpithet: eldaarayae; scientificNameAuthorship: Fleming & Wood, 2018; **Location:** continent: Central America; country: Costa Rica; countryCode: CR; stateProvince: Guanacaste; county: Sector Pitilla; locality: Area de Conservacion Guanacaste; verbatimLocality: Pasmompa; verbatimElevation: 440; verbatimLatitude: 11.0193; verbatimLongitude: -85.41; verbatimCoordinateSystem: Decimal; decimalLatitude: 11.0193; decimalLongitude: -85.41; **Identification:** identifiedBy: AJ Fleming; dateIdentified: 2017; **Event:** samplingProtocol: Reared from the larva of the Sphingidae, Xylophanes maculator; verbatimEventDate: 29-Jan-2001; **Record Level:** language: en; institutionCode: CNC; collectionCode: Insects; basisOfRecord: Pinned Specimen
- **Type status:** Other material. **Occurrence:** occurrenceDetails: http://janzen.sas.upenn.edu; catalogNumber: DHJPAR0008098; recordedBy: D.H. Janzen, W. Hallwachs & gusaneros; individualID: DHJPAR0008098; individualCount: 1; sex: male; lifeStage: adult; preparations: pinned; otherCatalogNumbers: ASTAT870-06, 98-SRNP-12364,; **Taxon:** scientificName: Hyphantrophaga eldaarayae; phylum: Arthropoda; class: Insecta; order: Diptera; family: Tachinidae; genus: Hyphantrophaga; specificEpithet: eldaarayae; scientificNameAuthorship: Fleming & Wood, 2018; **Location:** continent: Central America; country: Costa Rica; countryCode: CR; stateProvince: Guanacaste; county: Sector Santa Rosa; locality: Area de Conservacion Guanacaste; verbatimLocality: Bosque Interseccion; verbatimElevation: 15; verbatimLatitude: 10.8005; verbatimLongitude: -85.6492; verbatimCoordinateSystem: Decimal; decimalLatitude: 10.8005; decimalLongitude: -85.6492; **Identification:** identifiedBy: AJ Fleming; dateIdentified: 2017; **Event:** samplingProtocol: Reared from the larva of the Sphingidae, Xylophanes pluto; verbatimEventDate: 16-Nov-1998; **Record Level:** language: en; institutionCode: CNC; collectionCode: Insects; basisOfRecord: Pinned Specimen
- **Type status:** Other material. **Occurrence:** occurrenceDetails: http://janzen.sas.upenn.edu; catalogNumber: DHJPAR0008099; recordedBy: D.H. Janzen, W. Hallwachs & Lucia Rios; individualID: DHJPAR0008099; individualCount: 1; sex: male; lifeStage: adult; preparations: pinned; otherCatalogNumbers: ASTAT871-06, 98-SRNP-14084, BOLD:AAB0592; **Taxon:** scientificName: Hyphantrophaga eldaarayae; phylum: Arthropoda; class: Insecta; order: Diptera; family: Tachinidae; genus: Hyphantrophaga; specificEpithet: eldaarayae; scientificNameAuthorship: Fleming & Wood, 2018; **Location:** continent: Central America; country: Costa Rica; countryCode: CR; stateProvince: Guanacaste; county: Sector El Hacha; locality: Area de Conservacion Guanacaste; verbatimLocality: Sendero Bejuquilla; verbatimElevation: 280; verbatimLatitude: 11.03; verbatimLongitude: -85.527; verbatimCoordinateSystem: Decimal; decimalLatitude: 11.03; decimalLongitude: -85.527; **Identification:** identifiedBy: AJ Fleming; dateIdentified: 2017; **Event:** samplingProtocol: Reared from the larva of the Sphingidae, Xylophanes chiron; verbatimEventDate: 13-Dec-1998; **Record Level:** language: en; institutionCode: CNC; collectionCode: Insects; basisOfRecord: Pinned Specimen
- **Type status:** Other material. **Occurrence:** occurrenceDetails: http://janzen.sas.upenn.edu; catalogNumber: DHJPAR0008108; recordedBy: D.H. Janzen, W. Hallwachs & Carolina Cano; individualID: DHJPAR0008108; individualCount: 1; sex: male; lifeStage: adult; preparations: pinned; otherCatalogNumbers: ASTAT880-06, 03-SRNP-13019.1, BOLD:AAB0592; **Taxon:** scientificName: Hyphantrophaga eldaarayae; phylum: Arthropoda; class: Insecta; order: Diptera; family: Tachinidae; genus: Hyphantrophaga; specificEpithet: eldaarayae; scientificNameAuthorship: Fleming & Wood, 2018; **Location:** continent: Central America; country: Costa Rica; countryCode: CR; stateProvince: Alajuela; county: Sector Rincon Rain Forest; locality: Area de Conservacion Guanacaste; verbatimLocality: San Lucas; verbatimElevation: 320; verbatimLatitude: 10.9185; verbatimLongitude: -85.3034; verbatimCoordinateSystem: Decimal; decimalLatitude: 10.9185; decimalLongitude: -85.3034; **Identification:** identifiedBy: AJ Fleming; dateIdentified: 2017; **Event:** samplingProtocol: Reared from the larva of the Sphingidae, Xylophanes chiron; verbatimEventDate: 05-Nov-2003; **Record Level:** language: en; institutionCode: CNC; collectionCode: Insects; basisOfRecord: Pinned Specimen
- **Type status:** Other material. **Occurrence:** occurrenceDetails: http://janzen.sas.upenn.edu; catalogNumber: DHJPAR0015261; recordedBy: D.H. Janzen, W. Hallwachs & Roster Moraga; individualID: DHJPAR0015261; individualCount: 1; sex: male; lifeStage: adult; preparations: pinned; otherCatalogNumbers: ASTAS016-06, 06-SRNP-21420, BOLD:AAB0592; **Taxon:** scientificName: Hyphantrophaga eldaarayae; phylum: Arthropoda; class: Insecta; order: Diptera; family: Tachinidae; genus: Hyphantrophaga; specificEpithet: eldaarayae; scientificNameAuthorship: Fleming & Wood, 2018; **Location:** continent: Central America; country: Costa Rica; countryCode: CR; stateProvince: Guanacaste; county: Sector Del Oro; locality: Area de Conservacion Guanacaste; verbatimLocality: Margarita; verbatimElevation: 380; verbatimLatitude: 11.0323; verbatimLongitude: -85.4395; verbatimCoordinateSystem: Decimal; decimalLatitude: 11.0323; decimalLongitude: -85.4395; **Identification:** identifiedBy: AJ Fleming; dateIdentified: 2017; **Event:** samplingProtocol: Reared from the larva of the Sphingidae, Xylophanes chiron; verbatimEventDate: 09-Jul-2006; **Record Level:** language: en; institutionCode: CNC; collectionCode: Insects; basisOfRecord: Pinned Specimen
- **Type status:** Other material. **Occurrence:** occurrenceDetails: http://janzen.sas.upenn.edu; catalogNumber: DHJPAR0015262; recordedBy: D.H. Janzen, W. Hallwachs & Roster Moraga; individualID: DHJPAR0015262; individualCount: 1; sex: male; lifeStage: adult; preparations: pinned; otherCatalogNumbers: ASTAS017-06, 06-SRNP-21347, BOLD:AAB0592; **Taxon:** scientificName: Hyphantrophaga eldaarayae; phylum: Arthropoda; class: Insecta; order: Diptera; family: Tachinidae; genus: Hyphantrophaga; specificEpithet: eldaarayae; scientificNameAuthorship: Fleming & Wood, 2018; **Location:** continent: Central America; country: Costa Rica; countryCode: CR; stateProvince: Guanacaste; county: Sector Del Oro; locality: Area de Conservacion Guanacaste; verbatimLocality: Puente Mena; verbatimElevation: 280; verbatimLatitude: 11.0456; verbatimLongitude: -85.4574; verbatimCoordinateSystem: Decimal; decimalLatitude: 11.0456; decimalLongitude: -85.4574; **Identification:** identifiedBy: AJ Fleming; dateIdentified: 2017; **Event:** samplingProtocol: Reared from the larva of the Sphingidae, Xylophanes chiron; verbatimEventDate: 08-Jul-2006; **Record Level:** language: en; institutionCode: CNC; collectionCode: Insects; basisOfRecord: Pinned Specimen
- **Type status:** Other material. **Occurrence:** occurrenceDetails: http://janzen.sas.upenn.edu; catalogNumber: DHJPAR0015268; recordedBy: D.H. Janzen, W. Hallwachs & Minor Carmona; individualID: DHJPAR0015268; individualCount: 1; sex: male; lifeStage: adult; preparations: pinned; otherCatalogNumbers: ASTAS023-06, 06-SRNP-42101,; **Taxon:** scientificName: Hyphantrophaga eldaarayae; phylum: Arthropoda; class: Insecta; order: Diptera; family: Tachinidae; genus: Hyphantrophaga; specificEpithet: eldaarayae; scientificNameAuthorship: Fleming & Wood, 2018; **Location:** continent: Central America; country: Costa Rica; countryCode: CR; stateProvince: Alajuela; county: Sector Rincon Rain Forest; locality: Area de Conservacion Guanacaste; verbatimLocality: Puente Rio Negro; verbatimElevation: 340; verbatimLatitude: 10.9038; verbatimLongitude: -85.3027; verbatimCoordinateSystem: Decimal; decimalLatitude: 10.9038; decimalLongitude: -85.3027; **Identification:** identifiedBy: AJ Fleming; dateIdentified: 2017; **Event:** samplingProtocol: Reared from the larva of the Sphingidae, Xylophanes chiron; verbatimEventDate: 09-Jul-2006; **Record Level:** language: en; institutionCode: CNC; collectionCode: Insects; basisOfRecord: Pinned Specimen
- **Type status:** Other material. **Occurrence:** occurrenceDetails: http://janzen.sas.upenn.edu; catalogNumber: DHJPAR0016114; recordedBy: D.H. Janzen, W. Hallwachs & Anabelle Cordoba; individualID: DHJPAR0016114; individualCount: 1; sex: male; lifeStage: adult; preparations: pinned; otherCatalogNumbers: ASTAP143-06, 06-SRNP-7629, BOLD:AAB0592; **Taxon:** scientificName: Hyphantrophaga eldaarayae; phylum: Arthropoda; class: Insecta; order: Diptera; family: Tachinidae; genus: Hyphantrophaga; specificEpithet: eldaarayae; scientificNameAuthorship: Fleming & Wood, 2018; **Location:** continent: Central America; country: Costa Rica; countryCode: CR; stateProvince: Alajuela; county: Sector San Cristobal; locality: Area de Conservacion Guanacaste; verbatimLocality: Finca San Gabriel; verbatimElevation: 645; verbatimLatitude: 10.8777; verbatimLongitude: -85.3934; verbatimCoordinateSystem: Decimal; decimalLatitude: 10.8777; decimalLongitude: -85.3934; **Identification:** identifiedBy: AJ Fleming; dateIdentified: 2017; **Event:** samplingProtocol: Reared from the larva of the Sphingidae, Xylophanes chiron; verbatimEventDate: 18-Oct-2006; **Record Level:** language: en; institutionCode: CNC; collectionCode: Insects; basisOfRecord: Pinned Specimen
- **Type status:** Other material. **Occurrence:** occurrenceDetails: http://janzen.sas.upenn.edu; catalogNumber: DHJPAR0022017; recordedBy: D.H. Janzen, W. Hallwachs & Duvalier Briceno; individualID: DHJPAR0022017; individualCount: 1; sex: male; lifeStage: adult; preparations: pinned; otherCatalogNumbers: ASTAT1155-07, 07-SRNP-65374, BOLD:AAB0592; **Taxon:** scientificName: Hyphantrophaga eldaarayae; phylum: Arthropoda; class: Insecta; order: Diptera; family: Tachinidae; genus: Hyphantrophaga; specificEpithet: eldaarayae; scientificNameAuthorship: Fleming & Wood, 2018; **Location:** continent: Central America; country: Costa Rica; countryCode: CR; stateProvince: Alajuela; county: Brasilia; locality: Area de Conservacion Guanacaste; verbatimLocality: Piedrona; verbatimElevation: 340; verbatimLatitude: 11.0162; verbatimLongitude: -85.359; verbatimCoordinateSystem: Decimal; decimalLatitude: 11.0162; decimalLongitude: -85.359; **Identification:** identifiedBy: AJ Fleming; dateIdentified: 2017; **Event:** samplingProtocol: Reared from the larva of the Sphingidae, Xylophanes chiron; verbatimEventDate: 12-Sep-2007; **Record Level:** language: en; institutionCode: CNC; collectionCode: Insects; basisOfRecord: Pinned Specimen
- **Type status:** Other material. **Occurrence:** occurrenceDetails: http://janzen.sas.upenn.edu; catalogNumber: DHJPAR0022961; recordedBy: D.H. Janzen, W. Hallwachs & gusaneros; individualID: DHJPAR0022961; individualCount: 1; sex: male; lifeStage: adult; preparations: pinned; otherCatalogNumbers: ASTAW125-08, 86-SRNP-226, BOLD:AAB0592; **Taxon:** scientificName: Hyphantrophaga eldaarayae; phylum: Arthropoda; class: Insecta; order: Diptera; family: Tachinidae; genus: Hyphantrophaga; specificEpithet: eldaarayae; scientificNameAuthorship: Fleming & Wood, 2018; **Location:** continent: Central America; country: Costa Rica; countryCode: CR; stateProvince: Guanacaste; county: Sector Santa Rosa; locality: Area de Conservacion Guanacaste; verbatimLocality: Bosque Humedo; verbatimElevation: 290; verbatimLatitude: 10.8514; verbatimLongitude: -85.608; verbatimCoordinateSystem: Decimal; decimalLatitude: 10.8514; decimalLongitude: -85.608; **Identification:** identifiedBy: AJ Fleming; dateIdentified: 2017; **Event:** samplingProtocol: Reared from the larva of the Sphingidae, Xylophanes chiron; verbatimEventDate: 24-Jul-1986; **Record Level:** language: en; institutionCode: CNC; collectionCode: Insects; basisOfRecord: Pinned Specimen
- **Type status:** Other material. **Occurrence:** occurrenceDetails: http://janzen.sas.upenn.edu; catalogNumber: DHJPAR0022962; recordedBy: D.H. Janzen, W. Hallwachs & Gloria Sihezar; individualID: DHJPAR0022962; individualCount: 1; sex: male; lifeStage: adult; preparations: pinned; otherCatalogNumbers: ASTAW126-08, 01-SRNP-3991,; **Taxon:** scientificName: Hyphantrophaga eldaarayae; phylum: Arthropoda; class: Insecta; order: Diptera; family: Tachinidae; genus: Hyphantrophaga; specificEpithet: eldaarayae; scientificNameAuthorship: Fleming & Wood, 2018; **Location:** continent: Central America; country: Costa Rica; countryCode: CR; stateProvince: Alajuela; county: Sector San Cristobal; locality: Area de Conservacion Guanacaste; verbatimLocality: Rio Blanco Abajo; verbatimElevation: 500; verbatimLatitude: 10.9004; verbatimLongitude: -85.3725; verbatimCoordinateSystem: Decimal; decimalLatitude: 10.9004; decimalLongitude: -85.3725; **Identification:** identifiedBy: AJ Fleming; dateIdentified: 2017; **Event:** samplingProtocol: Reared from the larva of the Sphingidae, Xylophanes chiron; verbatimEventDate: 28-Nov-2001; **Record Level:** language: en; institutionCode: CNC; collectionCode: Insects; basisOfRecord: Pinned Specimen
- **Type status:** Other material. **Occurrence:** occurrenceDetails: http://janzen.sas.upenn.edu; catalogNumber: DHJPAR0005524; recordedBy: D.H. Janzen, W. Hallwachs & Carolina Cano; individualID: DHJPAR0005524; individualCount: 1; sex: male; lifeStage: adult; preparations: pinned; otherCatalogNumbers: ASTA643-06, 05-SRNP-7881,; **Taxon:** scientificName: Hyphantrophaga eldaarayae; phylum: Arthropoda; class: Insecta; order: Diptera; family: Tachinidae; genus: Hyphantrophaga; specificEpithet: eldaarayae; scientificNameAuthorship: Fleming & Wood, 2018; **Location:** continent: Central America; country: Costa Rica; countryCode: CR; stateProvince: Alajuela; county: Sector San Cristobal; locality: Area de Conservacion Guanacaste; verbatimLocality: Sendero Pinyal; verbatimElevation: 630; verbatimLatitude: 10.8716; verbatimLongitude: -85.3933; verbatimCoordinateSystem: Decimal; decimalLatitude: 10.8716; decimalLongitude: -85.3933; **Identification:** identifiedBy: AJ Fleming; dateIdentified: 2017; **Event:** samplingProtocol: Reared from the larva of the Sphingidae, Xylophanes chiron; verbatimEventDate: 30-Jan-2006; **Record Level:** language: en; institutionCode: CNC; collectionCode: Insects; basisOfRecord: Pinned Specimen
- **Type status:** Other material. **Occurrence:** occurrenceDetails: http://janzen.sas.upenn.edu; catalogNumber: DHJPAR0022964; recordedBy: D.H. Janzen, W. Hallwachs & Gloria Sihezar; individualID: DHJPAR0022964; individualCount: 1; sex: male; lifeStage: adult; preparations: pinned; otherCatalogNumbers: ASTAW128-08, 01-SRNP-3991, BOLD:AAB0592; **Taxon:** scientificName: Hyphantrophaga eldaarayae; phylum: Arthropoda; class: Insecta; order: Diptera; family: Tachinidae; genus: Hyphantrophaga; specificEpithet: eldaarayae; scientificNameAuthorship: Fleming & Wood, 2018; **Location:** continent: Central America; country: Costa Rica; countryCode: CR; stateProvince: Alajuela; county: Sector San Cristobal; locality: Area de Conservacion Guanacaste; verbatimLocality: Rio Blanco Abajo; verbatimElevation: 500; verbatimLatitude: 10.9004; verbatimLongitude: -85.3725; verbatimCoordinateSystem: Decimal; decimalLatitude: 10.9004; decimalLongitude: -85.3725; **Identification:** identifiedBy: AJ Fleming; dateIdentified: 2017; **Event:** samplingProtocol: Reared from the larva of the Sphingidae, Xylophanes chiron; verbatimEventDate: 28-Nov-2001; **Record Level:** language: en; institutionCode: CNC; collectionCode: Insects; basisOfRecord: Pinned Specimen
- **Type status:** Other material. **Occurrence:** occurrenceDetails: http://janzen.sas.upenn.edu; catalogNumber: DHJPAR0024606; recordedBy: D.H. Janzen, W. Hallwachs & Calixto Moraga; individualID: DHJPAR0024606; individualCount: 1; sex: male; lifeStage: adult; preparations: pinned; otherCatalogNumbers: ASTAW716-08, 08-SRNP-30750,; **Taxon:** scientificName: Hyphantrophaga eldaarayae; phylum: Arthropoda; class: Insecta; order: Diptera; family: Tachinidae; genus: Hyphantrophaga; specificEpithet: eldaarayae; scientificNameAuthorship: Fleming & Wood, 2018; **Location:** continent: Central America; country: Costa Rica; countryCode: CR; stateProvince: Guanacaste; county: Sector Pitilla; locality: Area de Conservacion Guanacaste; verbatimLocality: Sendero Mismo; verbatimElevation: 680; verbatimLatitude: 10.9876; verbatimLongitude: -85.4197; verbatimCoordinateSystem: Decimal; decimalLatitude: 10.9876; decimalLongitude: -85.4197; **Identification:** identifiedBy: AJ Fleming; dateIdentified: 2017; **Event:** samplingProtocol: Reared from the larva of the Sphingidae, Xylophanes chiron; verbatimEventDate: 18-Apr-2008; **Record Level:** language: en; institutionCode: CNC; collectionCode: Insects; basisOfRecord: Pinned Specimen
- **Type status:** Other material. **Occurrence:** occurrenceDetails: http://janzen.sas.upenn.edu; catalogNumber: DHJPAR0029703; recordedBy: D.H. Janzen, W. Hallwachs & Ronald Siezar; individualID: DHJPAR0029703; individualCount: 1; sex: male; lifeStage: adult; preparations: pinned; otherCatalogNumbers: ASHYM1124-09, 08-SRNP-71975, BOLD:AAB0592; **Taxon:** scientificName: Hyphantrophaga eldaarayae; phylum: Arthropoda; class: Insecta; order: Diptera; family: Tachinidae; genus: Hyphantrophaga; specificEpithet: eldaarayae; scientificNameAuthorship: Fleming & Wood, 2018; **Location:** continent: Central America; country: Costa Rica; countryCode: CR; stateProvince: Guanacaste; county: Sector Pitilla; locality: Area de Conservacion Guanacaste; verbatimLocality: Quebradona; verbatimElevation: 475; verbatimLatitude: 10.991; verbatimLongitude: -85.3954; verbatimCoordinateSystem: Decimal; decimalLatitude: 10.991; decimalLongitude: -85.3954; **Identification:** identifiedBy: AJ Fleming; dateIdentified: 2017; **Event:** samplingProtocol: Reared from the larva of the Sphingidae, Xylophanes chiron; verbatimEventDate: 08-Sep-2008; **Record Level:** language: en; institutionCode: CNC; collectionCode: Insects; basisOfRecord: Pinned Specimen
- **Type status:** Other material. **Occurrence:** occurrenceDetails: http://janzen.sas.upenn.edu; catalogNumber: DHJPAR0030007; recordedBy: D.H. Janzen, W. Hallwachs & Carolina Cano; individualID: DHJPAR0030007; individualCount: 1; sex: male; lifeStage: adult; preparations: pinned; otherCatalogNumbers: ASHYB751-09, 08-SRNP-6017, BOLD:AAB0592; **Taxon:** scientificName: Hyphantrophaga eldaarayae; phylum: Arthropoda; class: Insecta; order: Diptera; family: Tachinidae; genus: Hyphantrophaga; specificEpithet: eldaarayae; scientificNameAuthorship: Fleming & Wood, 2018; **Location:** continent: Central America; country: Costa Rica; countryCode: CR; stateProvince: Alajuela; county: Sector San Cristobal; locality: Area de Conservacion Guanacaste; verbatimLocality: Tajo Angeles; verbatimElevation: 540; verbatimLatitude: 10.8647; verbatimLongitude: -85.4153; verbatimCoordinateSystem: Decimal; decimalLatitude: 10.8647; decimalLongitude: -85.4153; **Identification:** identifiedBy: AJ Fleming; dateIdentified: 2017; **Event:** samplingProtocol: Reared from the larva of the Sphingidae, Xylophanes chiron; verbatimEventDate: 03-Dec-2007; **Record Level:** language: en; institutionCode: CNC; collectionCode: Insects; basisOfRecord: Pinned Specimen
- **Type status:** Other material. **Occurrence:** occurrenceDetails: http://janzen.sas.upenn.edu; catalogNumber: DHJPAR0030035; recordedBy: D.H. Janzen, W. Hallwachs & Anabelle Cordoba; individualID: DHJPAR0030035; individualCount: 1; sex: male; lifeStage: adult; preparations: pinned; otherCatalogNumbers: ASHYB779-09, 09-SRNP-40036, BOLD:AAB0592; **Taxon:** scientificName: Hyphantrophaga eldaarayae; phylum: Arthropoda; class: Insecta; order: Diptera; family: Tachinidae; genus: Hyphantrophaga; specificEpithet: eldaarayae; scientificNameAuthorship: Fleming & Wood, 2018; **Location:** continent: Central America; country: Costa Rica; countryCode: CR; stateProvince: Alajuela; county: Sector Rincon Rain Forest; locality: Area de Conservacion Guanacaste; verbatimLocality: San Lucas; verbatimElevation: 320; verbatimLatitude: 10.9185; verbatimLongitude: -85.3034; verbatimCoordinateSystem: Decimal; decimalLatitude: 10.9185; decimalLongitude: -85.3034; **Identification:** identifiedBy: AJ Fleming; dateIdentified: 2017; **Event:** samplingProtocol: Reared from the larva of the Sphingidae, Xylophanes porcus; verbatimEventDate: 16-Feb-2009; **Record Level:** language: en; institutionCode: CNC; collectionCode: Insects; basisOfRecord: Pinned Specimen
- **Type status:** Other material. **Occurrence:** occurrenceDetails: http://janzen.sas.upenn.edu; catalogNumber: DHJPAR0036488; recordedBy: D.H. Janzen, W. Hallwachs & Roster Moraga; individualID: DHJPAR0036488; individualCount: 1; sex: male; lifeStage: adult; preparations: pinned; otherCatalogNumbers: ASHYE1399-09, 09-SRNP-21680, BOLD:AAB0592; **Taxon:** scientificName: Hyphantrophaga eldaarayae; phylum: Arthropoda; class: Insecta; order: Diptera; family: Tachinidae; genus: Hyphantrophaga; specificEpithet: eldaarayae; scientificNameAuthorship: Fleming & Wood, 2018; **Location:** continent: Central America; country: Costa Rica; countryCode: CR; stateProvince: Guanacaste; county: Sector Del Oro; locality: Area de Conservacion Guanacaste; verbatimLocality: Rio Chon; verbatimElevation: 320; verbatimLatitude: 11.0412; verbatimLongitude: -85.4417; verbatimCoordinateSystem: Decimal; decimalLatitude: 11.0412; decimalLongitude: -85.4417; **Identification:** identifiedBy: AJ Fleming; dateIdentified: 2017; **Event:** samplingProtocol: Reared from the larva of the Sphingidae, Xylophanes chiron; verbatimEventDate: 31-Jul-2009; **Record Level:** language: en; institutionCode: CNC; collectionCode: Insects; basisOfRecord: Pinned Specimen
- **Type status:** Other material. **Occurrence:** occurrenceDetails: http://janzen.sas.upenn.edu; catalogNumber: DHJPAR0036559; recordedBy: D.H. Janzen, W. Hallwachs & Cirilo Umaña; individualID: DHJPAR0036559; individualCount: 1; sex: male; lifeStage: adult; preparations: pinned; otherCatalogNumbers: ASHYE1470-09, 09-SRNP-75401, BOLD:AAB0592; **Taxon:** scientificName: Hyphantrophaga eldaarayae; phylum: Arthropoda; class: Insecta; order: Diptera; family: Tachinidae; genus: Hyphantrophaga; specificEpithet: eldaarayae; scientificNameAuthorship: Fleming & Wood, 2018; **Location:** continent: Central America; country: Costa Rica; countryCode: CR; stateProvince: Alajuela; county: Sector Rincon Rain Forest; locality: Area de Conservacion Guanacaste; verbatimLocality: Estacion Llanura; verbatimElevation: 135; verbatimLatitude: 10.9333; verbatimLongitude: -85.2533; verbatimCoordinateSystem: Decimal; decimalLatitude: 10.9333; decimalLongitude: -85.2533; **Identification:** identifiedBy: AJ Fleming; dateIdentified: 2017; **Event:** samplingProtocol: Reared from the larva of the Sphingidae, Xylophanes chiron; verbatimEventDate: 21-Sep-2009; **Record Level:** language: en; institutionCode: CNC; collectionCode: Insects; basisOfRecord: Pinned Specimen
- **Type status:** Other material. **Occurrence:** occurrenceDetails: http://janzen.sas.upenn.edu; catalogNumber: DHJPAR0037479; recordedBy: D.H. Janzen, W. Hallwachs & Cirilo Umaña; individualID: DHJPAR0037479; individualCount: 1; sex: male; lifeStage: adult; preparations: pinned; otherCatalogNumbers: ASHYC4224-10, 10-SRNP-75042, BOLD:AAB0592; **Taxon:** scientificName: Hyphantrophaga eldaarayae; phylum: Arthropoda; class: Insecta; order: Diptera; family: Tachinidae; genus: Hyphantrophaga; specificEpithet: eldaarayae; scientificNameAuthorship: Fleming & Wood, 2018; **Location:** continent: Central America; country: Costa Rica; countryCode: CR; stateProvince: Alajuela; county: Sector Rincon Rain Forest; locality: Area de Conservacion Guanacaste; verbatimLocality: Estacion Llanura; verbatimElevation: 135; verbatimLatitude: 10.9333; verbatimLongitude: -85.2533; verbatimCoordinateSystem: Decimal; decimalLatitude: 10.9333; decimalLongitude: -85.2533; **Identification:** identifiedBy: AJ Fleming; dateIdentified: 2017; **Event:** samplingProtocol: Reared from the larva of the Sphingidae, Xylophanes chiron; verbatimEventDate: 09-Feb-2010; **Record Level:** language: en; institutionCode: CNC; collectionCode: Insects; basisOfRecord: Pinned Specimen
- **Type status:** Other material. **Occurrence:** occurrenceDetails: http://janzen.sas.upenn.edu; catalogNumber: DHJPAR0037657; recordedBy: D.H. Janzen, W. Hallwachs & Carolina Cano; individualID: DHJPAR0037657; individualCount: 1; sex: male; lifeStage: adult; preparations: pinned; otherCatalogNumbers: ASHYC4402-10, 07-SRNP-2058, BOLD:AAB0592; **Taxon:** scientificName: Hyphantrophaga eldaarayae; phylum: Arthropoda; class: Insecta; order: Diptera; family: Tachinidae; genus: Hyphantrophaga; specificEpithet: eldaarayae; scientificNameAuthorship: Fleming & Wood, 2018; **Location:** continent: Central America; country: Costa Rica; countryCode: CR; stateProvince: Alajuela; county: Sector San Cristobal; locality: Area de Conservacion Guanacaste; verbatimLocality: Vado Rio Cucaracho; verbatimElevation: 640; verbatimLatitude: 10.8702; verbatimLongitude: -85.3915; verbatimCoordinateSystem: Decimal; decimalLatitude: 10.8702; decimalLongitude: -85.3915; **Identification:** identifiedBy: AJ Fleming; dateIdentified: 2017; **Event:** samplingProtocol: Reared from the larva of the Sphingidae, Xylophanes chiron; verbatimEventDate: 25-Jul-2007; **Record Level:** language: en; institutionCode: CNC; collectionCode: Insects; basisOfRecord: Pinned Specimen
- **Type status:** Other material. **Occurrence:** occurrenceDetails: http://janzen.sas.upenn.edu; catalogNumber: DHJPAR0040181; recordedBy: D.H. Janzen, W. Hallwachs & Dinia Martinez; individualID: DHJPAR0040181; individualCount: 1; sex: male; lifeStage: adult; preparations: pinned; otherCatalogNumbers: ASHYE2348-11, 10-SRNP-72048,; **Taxon:** scientificName: Hyphantrophaga eldaarayae; phylum: Arthropoda; class: Insecta; order: Diptera; family: Tachinidae; genus: Hyphantrophaga; specificEpithet: eldaarayae; scientificNameAuthorship: Fleming & Wood, 2018; **Location:** continent: Central America; country: Costa Rica; countryCode: CR; stateProvince: Guanacaste; county: Sector Pitilla; locality: Area de Conservacion Guanacaste; verbatimLocality: Medrano; verbatimElevation: 380; verbatimLatitude: 11.016; verbatimLongitude: -85.3805; verbatimCoordinateSystem: Decimal; decimalLatitude: 11.016; decimalLongitude: -85.3805; **Identification:** identifiedBy: AJ Fleming; dateIdentified: 2017; **Event:** samplingProtocol: Reared from the larva of the Sphingidae, Xylophanes porcusDHJ04; verbatimEventDate: 16-Aug-2010; **Record Level:** language: en; institutionCode: CNC; collectionCode: Insects; basisOfRecord: Pinned Specimen
- **Type status:** Other material. **Occurrence:** occurrenceDetails: http://janzen.sas.upenn.edu; catalogNumber: DHJPAR0040182; recordedBy: D.H. Janzen, W. Hallwachs & Ricardo Calero; individualID: DHJPAR0040182; individualCount: 1; sex: male; lifeStage: adult; preparations: pinned; otherCatalogNumbers: ASHYE2349-11, 10-SRNP-72047,; **Taxon:** scientificName: Hyphantrophaga eldaarayae; phylum: Arthropoda; class: Insecta; order: Diptera; family: Tachinidae; genus: Hyphantrophaga; specificEpithet: eldaarayae; scientificNameAuthorship: Fleming & Wood, 2018; **Location:** continent: Central America; country: Costa Rica; countryCode: CR; stateProvince: Guanacaste; county: Sector Pitilla; locality: Area de Conservacion Guanacaste; verbatimLocality: Medrano; verbatimElevation: 380; verbatimLatitude: 11.016; verbatimLongitude: -85.3805; verbatimCoordinateSystem: Decimal; decimalLatitude: 11.016; decimalLongitude: -85.3805; **Identification:** identifiedBy: AJ Fleming; dateIdentified: 2017; **Event:** samplingProtocol: Reared from the larva of the Sphingidae, Xylophanes porcusDHJ04; verbatimEventDate: 13-Aug-2010; **Record Level:** language: en; institutionCode: CNC; collectionCode: Insects; basisOfRecord: Pinned Specimen
- **Type status:** Other material. **Occurrence:** occurrenceDetails: http://janzen.sas.upenn.edu; catalogNumber: DHJPAR0046641; recordedBy: D.H. Janzen, W. Hallwachs & Ricardo Calero; individualID: DHJPAR0046641; individualCount: 1; sex: male; lifeStage: adult; preparations: pinned; otherCatalogNumbers: ACGBA814-12, 11-SRNP-72185, BOLD:AAB0592; **Taxon:** scientificName: Hyphantrophaga eldaarayae; phylum: Arthropoda; class: Insecta; order: Diptera; family: Tachinidae; genus: Hyphantrophaga; specificEpithet: eldaarayae; scientificNameAuthorship: Fleming & Wood, 2018; **Location:** continent: Central America; country: Costa Rica; countryCode: CR; stateProvince: Guanacaste; county: Sector Pitilla; locality: Area de Conservacion Guanacaste; verbatimLocality: Manguera; verbatimElevation: 470; verbatimLatitude: 10.9959; verbatimLongitude: -85.3984; verbatimCoordinateSystem: Decimal; decimalLatitude: 10.9959; decimalLongitude: -85.3984; **Identification:** identifiedBy: AJ Fleming; dateIdentified: 2017; **Event:** samplingProtocol: Reared from the larva of the Sphingidae, Xylophanes chiron; verbatimEventDate: 21-Nov-2011; **Record Level:** language: en; institutionCode: CNC; collectionCode: Insects; basisOfRecord: Pinned Specimen
- **Type status:** Other material. **Occurrence:** occurrenceDetails: http://janzen.sas.upenn.edu; catalogNumber: DHJPAR0050202; recordedBy: D.H. Janzen, W. Hallwachs & Cirilo Umaña; individualID: DHJPAR0050202; individualCount: 1; sex: male; lifeStage: adult; preparations: pinned; otherCatalogNumbers: ACGAZ1516-12, 12-SRNP-76615, BOLD:AAB0592; **Taxon:** scientificName: Hyphantrophaga eldaarayae; phylum: Arthropoda; class: Insecta; order: Diptera; family: Tachinidae; genus: Hyphantrophaga; specificEpithet: eldaarayae; scientificNameAuthorship: Fleming & Wood, 2018; **Location:** continent: Central America; country: Costa Rica; countryCode: CR; stateProvince: Alajuela; county: Sector Rincon Rain Forest; locality: Area de Conservacion Guanacaste; verbatimLocality: Quebrada Bambu; verbatimElevation: 109; verbatimLatitude: 10.9301; verbatimLongitude: -85.2521; verbatimCoordinateSystem: Decimal; decimalLatitude: 10.9301; decimalLongitude: -85.2521; **Identification:** identifiedBy: AJ Fleming; dateIdentified: 2017; **Event:** samplingProtocol: Reared from the larva of the Sphingidae, Xylophanes chiron; verbatimEventDate: 16-Sep-2012; **Record Level:** language: en; institutionCode: CNC; collectionCode: Insects; basisOfRecord: Pinned Specimen
- **Type status:** Other material. **Occurrence:** occurrenceDetails: http://janzen.sas.upenn.edu; catalogNumber: DHJPAR0050239; recordedBy: D.H. Janzen, W. Hallwachs & Edwin Apu; individualID: DHJPAR0050239; individualCount: 1; sex: male; lifeStage: adult; preparations: pinned; otherCatalogNumbers: ACGAZ1553-12, 12-SRNP-69449, BOLD:AAB0592; **Taxon:** scientificName: Hyphantrophaga eldaarayae; phylum: Arthropoda; class: Insecta; order: Diptera; family: Tachinidae; genus: Hyphantrophaga; specificEpithet: eldaarayae; scientificNameAuthorship: Fleming & Wood, 2018; **Location:** continent: Central America; country: Costa Rica; countryCode: CR; stateProvince: Alajuela; county: Sector Rincon Rain Forest; locality: Area de Conservacion Guanacaste; verbatimLocality: Selva; verbatimElevation: 410; verbatimLatitude: 10.9229; verbatimLongitude: -85.3188; verbatimCoordinateSystem: Decimal; decimalLatitude: 10.9229; decimalLongitude: -85.3188; **Identification:** identifiedBy: AJ Fleming; dateIdentified: 2017; **Event:** samplingProtocol: Reared from the larva of the Sphingidae, Xylophanes hannemanni; verbatimEventDate: 04-Apr-2012; **Record Level:** language: en; institutionCode: CNC; collectionCode: Insects; basisOfRecord: Pinned Specimen
- **Type status:** Other material. **Occurrence:** occurrenceDetails: http://janzen.sas.upenn.edu; catalogNumber: DHJPAR0050493; recordedBy: D.H. Janzen, W. Hallwachs & Cirilo Umaña; individualID: DHJPAR0050493; individualCount: 1; sex: male; lifeStage: adult; preparations: pinned; otherCatalogNumbers: ACGBA3085-13, 12-SRNP-77956, BOLD:AAB0592; **Taxon:** scientificName: Hyphantrophaga eldaarayae; phylum: Arthropoda; class: Insecta; order: Diptera; family: Tachinidae; genus: Hyphantrophaga; specificEpithet: eldaarayae; scientificNameAuthorship: Fleming & Wood, 2018; **Location:** continent: Central America; country: Costa Rica; countryCode: CR; stateProvince: Alajuela; county: Sector Rincon Rain Forest; locality: Area de Conservacion Guanacaste; verbatimLocality: Finca Esmeralda; verbatimElevation: 123; verbatimLatitude: 10.9355; verbatimLongitude: -85.2531; verbatimCoordinateSystem: Decimal; decimalLatitude: 10.9355; decimalLongitude: -85.2531; **Identification:** identifiedBy: AJ Fleming; dateIdentified: 2017; **Event:** samplingProtocol: Reared from the larva of the Sphingidae, Xylophanes chiron; verbatimEventDate: 04-Jan-2013; **Record Level:** language: en; institutionCode: CNC; collectionCode: Insects; basisOfRecord: Pinned Specimen
- **Type status:** Other material. **Occurrence:** occurrenceDetails: http://janzen.sas.upenn.edu; catalogNumber: DHJPAR0050533; recordedBy: D.H. Janzen, W. Hallwachs & Harry Ramirez; individualID: DHJPAR0050533; individualCount: 1; sex: male; lifeStage: adult; preparations: pinned; otherCatalogNumbers: ACGBA3125-13, 12-SRNP-35511, BOLD:AAB0592; **Taxon:** scientificName: Hyphantrophaga eldaarayae; phylum: Arthropoda; class: Insecta; order: Diptera; family: Tachinidae; genus: Hyphantrophaga; specificEpithet: eldaarayae; scientificNameAuthorship: Fleming & Wood, 2018; **Location:** continent: Central America; country: Costa Rica; countryCode: CR; stateProvince: Guanacaste; county: Sector Cacao; locality: Area de Conservacion Guanacaste; verbatimLocality: Estacion Cacao; verbatimElevation: 1150; verbatimLatitude: 10.9269; verbatimLongitude: -85.4682; verbatimCoordinateSystem: Decimal; decimalLatitude: 10.9269; decimalLongitude: -85.4682; **Identification:** identifiedBy: AJ Fleming; dateIdentified: 2017; **Event:** samplingProtocol: Reared from the larva of the Sphingidae, Xylophanes crotonis; verbatimEventDate: 19-Dec-2012; **Record Level:** language: en; institutionCode: CNC; collectionCode: Insects; basisOfRecord: Pinned Specimen
- **Type status:** Other material. **Occurrence:** occurrenceDetails: http://janzen.sas.upenn.edu; catalogNumber: DHJPAR0050644; recordedBy: D.H. Janzen, W. Hallwachs & Keiner Aragon; individualID: DHJPAR0050644; individualCount: 1; sex: male; lifeStage: adult; preparations: pinned; otherCatalogNumbers: ACGBA3236-13, 12-SRNP-68836,; **Taxon:** scientificName: Hyphantrophaga eldaarayae; phylum: Arthropoda; class: Insecta; order: Diptera; family: Tachinidae; genus: Hyphantrophaga; specificEpithet: eldaarayae; scientificNameAuthorship: Fleming & Wood, 2018; **Location:** continent: Central America; country: Costa Rica; countryCode: CR; stateProvince: Alajuela; county: Sector Rincon Rain Forest; locality: Area de Conservacion Guanacaste; verbatimLocality: Pochote; verbatimElevation: 150; verbatimLatitude: 10.953; verbatimLongitude: -85.2698; verbatimCoordinateSystem: Decimal; decimalLatitude: 10.953; decimalLongitude: -85.2698; **Identification:** identifiedBy: AJ Fleming; dateIdentified: 2017; **Event:** samplingProtocol: Reared from the larva of the Sphingidae, Xylophanes hannemanni; verbatimEventDate: 13-Dec-2012; **Record Level:** language: en; institutionCode: CNC; collectionCode: Insects; basisOfRecord: Pinned Specimen
- **Type status:** Other material. **Occurrence:** occurrenceDetails: http://janzen.sas.upenn.edu; catalogNumber: DHJPAR0053425; recordedBy: D.H. Janzen, W. Hallwachs & Keiner Aragon; individualID: DHJPAR0053425; individualCount: 1; sex: male; lifeStage: adult; preparations: pinned; otherCatalogNumbers: ASHYM2779-13, 13-SRNP-79150, BOLD:AAB0592; **Taxon:** scientificName: Hyphantrophaga eldaarayae; phylum: Arthropoda; class: Insecta; order: Diptera; family: Tachinidae; genus: Hyphantrophaga; specificEpithet: eldaarayae; scientificNameAuthorship: Fleming & Wood, 2018; **Location:** continent: Central America; country: Costa Rica; countryCode: CR; stateProvince: Alajuela; county: Sector Rincon Rain Forest; locality: Area de Conservacion Guanacaste; verbatimLocality: Palomo; verbatimElevation: 96; verbatimLatitude: 10.9619; verbatimLongitude: -85.2804; verbatimCoordinateSystem: Decimal; decimalLatitude: 10.9619; decimalLongitude: -85.2804; **Identification:** identifiedBy: AJ Fleming; dateIdentified: 2017; **Event:** samplingProtocol: Reared from the larva of the Sphingidae, Xylophanes chiron; verbatimEventDate: 05-Sep-2013; **Record Level:** language: en; institutionCode: CNC; collectionCode: Insects; basisOfRecord: Pinned Specimen
- **Type status:** Other material. **Occurrence:** occurrenceDetails: http://janzen.sas.upenn.edu; catalogNumber: DHJPAR0056176; recordedBy: D.H. Janzen, W. Hallwachs & Edwin Apu; individualID: DHJPAR0056176; individualCount: 1; sex: male; lifeStage: adult; preparations: pinned; otherCatalogNumbers: ASHYH2433-14, 14-SRNP-81055,; **Taxon:** scientificName: Hyphantrophaga eldaarayae; phylum: Arthropoda; class: Insecta; order: Diptera; family: Tachinidae; genus: Hyphantrophaga; specificEpithet: eldaarayae; scientificNameAuthorship: Fleming & Wood, 2018; **Location:** continent: Central America; country: Costa Rica; countryCode: CR; stateProvince: Alajuela; county: Sector Rincon Rain Forest; locality: Area de Conservacion Guanacaste; verbatimLocality: Jacobo; verbatimElevation: 461; verbatimLatitude: 10.9408; verbatimLongitude: -85.3177; verbatimCoordinateSystem: Decimal; decimalLatitude: 10.9408; decimalLongitude: -85.3177; **Identification:** identifiedBy: AJ Fleming; dateIdentified: 2017; **Event:** samplingProtocol: Reared from the larva of the Sphingidae, Xylophanes chiron; verbatimEventDate: 21-Sep-2014; **Record Level:** language: en; institutionCode: CNC; collectionCode: Insects; basisOfRecord: Pinned Specimen
- **Type status:** Other material. **Occurrence:** occurrenceDetails: http://janzen.sas.upenn.edu; catalogNumber: DHJPAR0056177; recordedBy: D.H. Janzen, W. Hallwachs & Keiner Aragon; individualID: DHJPAR0056177; individualCount: 1; sex: male; lifeStage: adult; preparations: pinned; otherCatalogNumbers: ASHYH2434-14, 14-SRNP-46592, BOLD:AAB0592; **Taxon:** scientificName: Hyphantrophaga eldaarayae; phylum: Arthropoda; class: Insecta; order: Diptera; family: Tachinidae; genus: Hyphantrophaga; specificEpithet: eldaarayae; scientificNameAuthorship: Fleming & Wood, 2018; **Location:** continent: Central America; country: Costa Rica; countryCode: CR; stateProvince: Alajuela; county: Sector Rincon Rain Forest; locality: Area de Conservacion Guanacaste; verbatimLocality: Palomo; verbatimElevation: 96; verbatimLatitude: 10.9619; verbatimLongitude: -85.2804; verbatimCoordinateSystem: Decimal; decimalLatitude: 10.9619; decimalLongitude: -85.2804; **Identification:** identifiedBy: AJ Fleming; dateIdentified: 2017; **Event:** samplingProtocol: Reared from the larva of the Sphingidae, Xylophanes chiron; verbatimEventDate: 11-Sep-2014; **Record Level:** language: en; institutionCode: CNC; collectionCode: Insects; basisOfRecord: Pinned Specimen
- **Type status:** Other material. **Occurrence:** occurrenceDetails: http://janzen.sas.upenn.edu; catalogNumber: DHJPAR0057150; recordedBy: D.H. Janzen, W. Hallwachs & Minor Carmona; individualID: DHJPAR0057150; individualCount: 1; sex: male; lifeStage: adult; preparations: pinned; otherCatalogNumbers: ACGBA5060-15, 14-SRNP-81304, BOLD:AAB0592; **Taxon:** scientificName: Hyphantrophaga eldaarayae; phylum: Arthropoda; class: Insecta; order: Diptera; family: Tachinidae; genus: Hyphantrophaga; specificEpithet: eldaarayae; scientificNameAuthorship: Fleming & Wood, 2018; **Location:** continent: Central America; country: Costa Rica; countryCode: CR; stateProvince: Alajuela; county: Sector Rincon Rain Forest; locality: Area de Conservacion Guanacaste; verbatimLocality: Chayito; verbatimElevation: 470; verbatimLatitude: 10.9459; verbatimLongitude: -85.3204; verbatimCoordinateSystem: Decimal; decimalLatitude: 10.9459; decimalLongitude: -85.3204; **Identification:** identifiedBy: AJ Fleming; dateIdentified: 2017; **Event:** samplingProtocol: Reared from the larva of the Sphingidae, Xylophanes chiron; verbatimEventDate: 03-Nov-2014; **Record Level:** language: en; institutionCode: CNC; collectionCode: Insects; basisOfRecord: Pinned Specimen
- **Type status:** Other material. **Occurrence:** occurrenceDetails: http://janzen.sas.upenn.edu; catalogNumber: DHJPAR0057226; recordedBy: D.H. Janzen, W. Hallwachs & Edwin Apu; individualID: DHJPAR0057226; individualCount: 1; sex: male; lifeStage: adult; preparations: pinned; otherCatalogNumbers: ACGBA5136-15, 14-SRNP-81650, BOLD:AAB0592; **Taxon:** scientificName: Hyphantrophaga eldaarayae; phylum: Arthropoda; class: Insecta; order: Diptera; family: Tachinidae; genus: Hyphantrophaga; specificEpithet: eldaarayae; scientificNameAuthorship: Fleming & Wood, 2018; **Location:** continent: Central America; country: Costa Rica; countryCode: CR; stateProvince: Alajuela; county: Sector Rincon Rain Forest; locality: Area de Conservacion Guanacaste; verbatimLocality: Jacobo; verbatimElevation: 461; verbatimLatitude: 10.9408; verbatimLongitude: -85.3177; verbatimCoordinateSystem: Decimal; decimalLatitude: 10.9408; decimalLongitude: -85.3177; **Identification:** identifiedBy: AJ Fleming; dateIdentified: 2017; **Event:** samplingProtocol: Reared from the larva of the Sphingidae, Xylophanes chiron; verbatimEventDate: 27-Dec-2014; **Record Level:** language: en; institutionCode: CNC; collectionCode: Insects; basisOfRecord: Pinned Specimen
- **Type status:** Other material. **Occurrence:** occurrenceDetails: http://janzen.sas.upenn.edu; catalogNumber: DHJPAR0057245; recordedBy: D.H. Janzen, W. Hallwachs & Cirilo Umaña; individualID: DHJPAR0057245; individualCount: 1; sex: male; lifeStage: adult; preparations: pinned; otherCatalogNumbers: ACGBA5155-15, 15-SRNP-75028,; **Taxon:** scientificName: Hyphantrophaga eldaarayae; phylum: Arthropoda; class: Insecta; order: Diptera; family: Tachinidae; genus: Hyphantrophaga; specificEpithet: eldaarayae; scientificNameAuthorship: Fleming & Wood, 2018; **Location:** continent: Central America; country: Costa Rica; countryCode: CR; stateProvince: Alajuela; county: Sector Rincon Rain Forest; locality: Area de Conservacion Guanacaste; verbatimLocality: Finca Esmeralda; verbatimElevation: 123; verbatimLatitude: 10.9355; verbatimLongitude: -85.2531; verbatimCoordinateSystem: Decimal; decimalLatitude: 10.9355; decimalLongitude: -85.2531; **Identification:** identifiedBy: AJ Fleming; dateIdentified: 2017; **Event:** samplingProtocol: Reared from the larva of the Sphingidae, Xylophanes cthulhu; verbatimEventDate: 06-Feb-2015; **Record Level:** language: en; institutionCode: CNC; collectionCode: Insects; basisOfRecord: Pinned Specimen
- **Type status:** Other material. **Occurrence:** occurrenceDetails: http://janzen.sas.upenn.edu; catalogNumber: DHJPAR0058314; recordedBy: D.H. Janzen, W. Hallwachs & Keiner Aragon; individualID: DHJPAR0058314; individualCount: 1; sex: male; lifeStage: adult; preparations: pinned; otherCatalogNumbers: MHMYN7914-16, 15-SRNP-46229, BOLD:AAB0592; **Taxon:** scientificName: Hyphantrophaga eldaarayae; phylum: Arthropoda; class: Insecta; order: Diptera; family: Tachinidae; genus: Hyphantrophaga; specificEpithet: eldaarayae; scientificNameAuthorship: Fleming & Wood, 2018; **Location:** continent: Central America; country: Costa Rica; countryCode: CR; stateProvince: Alajuela; county: Sector Rincon Rain Forest; locality: Area de Conservacion Guanacaste; verbatimLocality: Palomo; verbatimElevation: 96; verbatimLatitude: 10.9619; verbatimLongitude: -85.2804; verbatimCoordinateSystem: Decimal; decimalLatitude: 10.9619; decimalLongitude: -85.2804; **Identification:** identifiedBy: AJ Fleming; dateIdentified: 2017; **Event:** samplingProtocol: Reared from the larva of the Sphingidae, Xylophanes germen; verbatimEventDate: 28-Oct-2015; **Record Level:** language: en; institutionCode: CNC; collectionCode: Insects; basisOfRecord: Pinned Specimen
- **Type status:** Other material. **Occurrence:** occurrenceDetails: http://janzen.sas.upenn.edu; catalogNumber: DHJPAR0058415; recordedBy: D.H. Janzen, W. Hallwachs & Keiner Aragon; individualID: DHJPAR0058415; individualCount: 1; sex: male; lifeStage: adult; preparations: pinned; otherCatalogNumbers: MHMYN8015-16, 15-SRNP-46524, BOLD:AAB0592; **Taxon:** scientificName: Hyphantrophaga eldaarayae; phylum: Arthropoda; class: Insecta; order: Diptera; family: Tachinidae; genus: Hyphantrophaga; specificEpithet: eldaarayae; scientificNameAuthorship: Fleming & Wood, 2018; **Location:** continent: Central America; country: Costa Rica; countryCode: CR; stateProvince: Alajuela; county: Sector Rincon Rain Forest; locality: Area de Conservacion Guanacaste; verbatimLocality: Casa Keyner; verbatimElevation: 121; verbatimLatitude: 10.9564; verbatimLongitude: -85.2661; verbatimCoordinateSystem: Decimal; decimalLatitude: 10.9564; decimalLongitude: -85.2661; **Identification:** identifiedBy: AJ Fleming; dateIdentified: 2017; **Event:** samplingProtocol: Reared from the larva of the Sphingidae, Xylophanes guianensis; verbatimEventDate: 05-Jan-2016; **Record Level:** language: en; institutionCode: CNC; collectionCode: Insects; basisOfRecord: Pinned Specimen
- **Type status:** Other material. **Occurrence:** occurrenceDetails: http://janzen.sas.upenn.edu; catalogNumber: DHJPAR0008079; recordedBy: D.H. Janzen, W. Hallwachs & Roster Moraga; individualID: DHJPAR0008079; individualCount: 1; sex: female; lifeStage: adult; preparations: pinned; otherCatalogNumbers: ASTAT851-06, 03-SRNP-17832,; **Taxon:** scientificName: Hyphantrophaga eldaarayae; phylum: Arthropoda; class: Insecta; order: Diptera; family: Tachinidae; genus: Hyphantrophaga; specificEpithet: eldaarayae; scientificNameAuthorship: Fleming & Wood, 2018; **Location:** continent: Central America; country: Costa Rica; countryCode: CR; stateProvince: Guanacaste; county: Sector Del Oro; locality: Area de Conservacion Guanacaste; verbatimLocality: Sendero Puertas; verbatimElevation: 400; verbatimLatitude: 11.0109; verbatimLongitude: -85.4882; verbatimCoordinateSystem: Decimal; decimalLatitude: 11.0109; decimalLongitude: -85.4882; **Identification:** identifiedBy: AJ Fleming; dateIdentified: 2017; **Event:** samplingProtocol: Reared from the larva of the Sphingidae, Xylophanes chiron; verbatimEventDate: 12-Aug-2003; **Record Level:** language: en; institutionCode: CNC; collectionCode: Insects; basisOfRecord: Pinned Specimen
- **Type status:** Other material. **Occurrence:** occurrenceDetails: http://janzen.sas.upenn.edu; catalogNumber: DHJPAR0008085; recordedBy: D.H. Janzen, W. Hallwachs & Guillermo Pereira; individualID: DHJPAR0008085; individualCount: 1; sex: female; lifeStage: adult; preparations: pinned; otherCatalogNumbers: ASTAT857-06, 05-SRNP-61306,; **Taxon:** scientificName: Hyphantrophaga eldaarayae; phylum: Arthropoda; class: Insecta; order: Diptera; family: Tachinidae; genus: Hyphantrophaga; specificEpithet: eldaarayae; scientificNameAuthorship: Fleming & Wood, 2018; **Location:** continent: Central America; country: Costa Rica; countryCode: CR; stateProvince: Guanacaste; county: Sector Santa Rosa; locality: Area de Conservacion Guanacaste; verbatimLocality: Quebrada Puercos; verbatimElevation: 155; verbatimLatitude: 10.8591; verbatimLongitude: -85.5709; verbatimCoordinateSystem: Decimal; decimalLatitude: 10.8591; decimalLongitude: -85.5709; **Identification:** identifiedBy: AJ Fleming; dateIdentified: 2017; **Event:** samplingProtocol: Reared from the larva of the Sphingidae, Xylophanes pluto; verbatimEventDate: 19-Oct-2005; **Record Level:** language: en; institutionCode: CNC; collectionCode: Insects; basisOfRecord: Pinned Specimen
- **Type status:** Other material. **Occurrence:** occurrenceDetails: http://janzen.sas.upenn.edu; catalogNumber: DHJPAR0008089; recordedBy: D.H. Janzen, W. Hallwachs & Lucia Rios; individualID: DHJPAR0008089; individualCount: 1; sex: female; lifeStage: adult; preparations: pinned; otherCatalogNumbers: ASTAT861-06, 00-SRNP-2778, BOLD:AAB0592; **Taxon:** scientificName: Hyphantrophaga eldaarayae; phylum: Arthropoda; class: Insecta; order: Diptera; family: Tachinidae; genus: Hyphantrophaga; specificEpithet: eldaarayae; scientificNameAuthorship: Fleming & Wood, 2018; **Location:** continent: Central America; country: Costa Rica; countryCode: CR; stateProvince: Guanacaste; county: Sector El Hacha; locality: Area de Conservacion Guanacaste; verbatimLocality: Sendero Tigre; verbatimElevation: 280; verbatimLatitude: 11.0317; verbatimLongitude: -85.5262; verbatimCoordinateSystem: Decimal; decimalLatitude: 11.0317; decimalLongitude: -85.5262; **Identification:** identifiedBy: AJ Fleming; dateIdentified: 2017; **Event:** samplingProtocol: Reared from the larva of the Sphingidae, Xylophanes tyndarus; verbatimEventDate: 17-Jul-2000; **Record Level:** language: en; institutionCode: CNC; collectionCode: Insects; basisOfRecord: Pinned Specimen
- **Type status:** Other material. **Occurrence:** occurrenceDetails: http://janzen.sas.upenn.edu; catalogNumber: DHJPAR0008090; recordedBy: D.H. Janzen, W. Hallwachs & Mariano Pereira; individualID: DHJPAR0008090; individualCount: 1; sex: female; lifeStage: adult; preparations: pinned; otherCatalogNumbers: ASTAT862-06, 97-SRNP-4524,; **Taxon:** scientificName: Hyphantrophaga eldaarayae; phylum: Arthropoda; class: Insecta; order: Diptera; family: Tachinidae; genus: Hyphantrophaga; specificEpithet: eldaarayae; scientificNameAuthorship: Fleming & Wood, 2018; **Location:** continent: Central America; country: Costa Rica; countryCode: CR; stateProvince: Guanacaste; county: Sector El Hacha; locality: Area de Conservacion Guanacaste; verbatimLocality: Sendero Tigre; verbatimElevation: 280; verbatimLatitude: 11.0317; verbatimLongitude: -85.5262; verbatimCoordinateSystem: Decimal; decimalLatitude: 11.0317; decimalLongitude: -85.5262; **Identification:** identifiedBy: AJ Fleming; dateIdentified: 2017; **Event:** samplingProtocol: Reared from the larva of the Sphingidae, Xylophanes chiron; verbatimEventDate: 23-Jul-1997; **Record Level:** language: en; institutionCode: CNC; collectionCode: Insects; basisOfRecord: Pinned Specimen
- **Type status:** Other material. **Occurrence:** occurrenceDetails: http://janzen.sas.upenn.edu; catalogNumber: DHJPAR0008092; recordedBy: D.H. Janzen, W. Hallwachs & Jose Perez; individualID: DHJPAR0008092; individualCount: 1; sex: female; lifeStage: adult; preparations: pinned; otherCatalogNumbers: ASTAT864-06, 04-SRNP-40099, BOLD:AAB0592; **Taxon:** scientificName: Hyphantrophaga eldaarayae; phylum: Arthropoda; class: Insecta; order: Diptera; family: Tachinidae; genus: Hyphantrophaga; specificEpithet: eldaarayae; scientificNameAuthorship: Fleming & Wood, 2018; **Location:** continent: Central America; country: Costa Rica; countryCode: CR; stateProvince: Alajuela; county: Sector Rincon Rain Forest; locality: Area de Conservacion Guanacaste; verbatimLocality: Camino Porvenir; verbatimElevation: 383; verbatimLatitude: 10.9038; verbatimLongitude: -85.2596; verbatimCoordinateSystem: Decimal; decimalLatitude: 10.9038; decimalLongitude: -85.2596; **Identification:** identifiedBy: AJ Fleming; dateIdentified: 2017; **Event:** samplingProtocol: Reared from the larva of the Sphingidae, Xylophanes chiron; verbatimEventDate: 13-Feb-2004; **Record Level:** language: en; institutionCode: CNC; collectionCode: Insects; basisOfRecord: Pinned Specimen
- **Type status:** Other material. **Occurrence:** occurrenceDetails: http://janzen.sas.upenn.edu; catalogNumber: DHJPAR0008101; recordedBy: D.H. Janzen, W. Hallwachs & Lucia Rios; individualID: DHJPAR0008101; individualCount: 1; sex: female; lifeStage: adult; preparations: pinned; otherCatalogNumbers: ASTAT873-06, 99-SRNP-2043, BOLD:AAB0592; **Taxon:** scientificName: Hyphantrophaga eldaarayae; phylum: Arthropoda; class: Insecta; order: Diptera; family: Tachinidae; genus: Hyphantrophaga; specificEpithet: eldaarayae; scientificNameAuthorship: Fleming & Wood, 2018; **Location:** continent: Central America; country: Costa Rica; countryCode: CR; stateProvince: Guanacaste; county: Sector El Hacha; locality: Area de Conservacion Guanacaste; verbatimLocality: Sendero Tigre; verbatimElevation: 280; verbatimLatitude: 11.0317; verbatimLongitude: -85.5262; verbatimCoordinateSystem: Decimal; decimalLatitude: 11.0317; decimalLongitude: -85.5262; **Identification:** identifiedBy: AJ Fleming; dateIdentified: 2017; **Event:** samplingProtocol: Reared from the larva of the Sphingidae, Xylophanes chiron; verbatimEventDate: 02-Sep-1999; **Record Level:** language: en; institutionCode: CNC; collectionCode: Insects; basisOfRecord: Pinned Specimen
- **Type status:** Other material. **Occurrence:** occurrenceDetails: http://janzen.sas.upenn.edu; catalogNumber: DHJPAR0008102; recordedBy: D.H. Janzen, W. Hallwachs & Lucia Rios; individualID: DHJPAR0008102; individualCount: 1; sex: female; lifeStage: adult; preparations: pinned; otherCatalogNumbers: ASTAT874-06, 97-SRNP-4646, BOLD:AAB0592; **Taxon:** scientificName: Hyphantrophaga eldaarayae; phylum: Arthropoda; class: Insecta; order: Diptera; family: Tachinidae; genus: Hyphantrophaga; specificEpithet: eldaarayae; scientificNameAuthorship: Fleming & Wood, 2018; **Location:** continent: Central America; country: Costa Rica; countryCode: CR; stateProvince: Guanacaste; county: Sector El Hacha; locality: Area de Conservacion Guanacaste; verbatimLocality: Sendero Bejuquilla; verbatimElevation: 280; verbatimLatitude: 11.03; verbatimLongitude: -85.527; verbatimCoordinateSystem: Decimal; decimalLatitude: 11.03; decimalLongitude: -85.527; **Identification:** identifiedBy: AJ Fleming; dateIdentified: 2017; **Event:** samplingProtocol: Reared from the larva of the Sphingidae, Xylophanes chiron; verbatimEventDate: 08-May-1997; **Record Level:** language: en; institutionCode: CNC; collectionCode: Insects; basisOfRecord: Pinned Specimen
- **Type status:** Other material. **Occurrence:** occurrenceDetails: http://janzen.sas.upenn.edu; catalogNumber: DHJPAR0008103; recordedBy: D.H. Janzen, W. Hallwachs & gusaneros; individualID: DHJPAR0008103; individualCount: 1; sex: female; lifeStage: adult; preparations: pinned; otherCatalogNumbers: ASTAT875-06, 91-SRNP-3000, BOLD:AAB0592; **Taxon:** scientificName: Hyphantrophaga eldaarayae; phylum: Arthropoda; class: Insecta; order: Diptera; family: Tachinidae; genus: Hyphantrophaga; specificEpithet: eldaarayae; scientificNameAuthorship: Fleming & Wood, 2018; **Location:** continent: Central America; country: Costa Rica; countryCode: CR; stateProvince: Guanacaste; county: Sector Santa Rosa; locality: Area de Conservacion Guanacaste; verbatimLocality: Bosque Humedo; verbatimElevation: 290; verbatimLatitude: 10.8514; verbatimLongitude: -85.608; verbatimCoordinateSystem: Decimal; decimalLatitude: 10.8514; decimalLongitude: -85.608; **Identification:** identifiedBy: AJ Fleming; dateIdentified: 2017; **Event:** samplingProtocol: Reared from the larva of the Sphingidae, Xylophanes anubus; verbatimEventDate: 18-Sep-1991; **Record Level:** language: en; institutionCode: CNC; collectionCode: Insects; basisOfRecord: Pinned Specimen
- **Type status:** Other material. **Occurrence:** occurrenceDetails: http://janzen.sas.upenn.edu; catalogNumber: DHJPAR0008104; recordedBy: D.H. Janzen, W. Hallwachs & Mariano Pereira; individualID: DHJPAR0008104; individualCount: 1; sex: female; lifeStage: adult; preparations: pinned; otherCatalogNumbers: ASTAT876-06, 99-SRNP-1301, BOLD:AAB0592; **Taxon:** scientificName: Hyphantrophaga eldaarayae; phylum: Arthropoda; class: Insecta; order: Diptera; family: Tachinidae; genus: Hyphantrophaga; specificEpithet: eldaarayae; scientificNameAuthorship: Fleming & Wood, 2018; **Location:** continent: Central America; country: Costa Rica; countryCode: CR; stateProvince: Guanacaste; county: Sector Cacao; locality: Area de Conservacion Guanacaste; verbatimLocality: Sendero Nayo; verbatimElevation: 1090; verbatimLatitude: 10.9245; verbatimLongitude: -85.4695; verbatimCoordinateSystem: Decimal; decimalLatitude: 10.9245; decimalLongitude: -85.4695; **Identification:** identifiedBy: AJ Fleming; dateIdentified: 2017; **Event:** samplingProtocol: Reared from the larva of the Sphingidae, Xylophanes crotonis; verbatimEventDate: 09-Jul-1999; **Record Level:** language: en; institutionCode: CNC; collectionCode: Insects; basisOfRecord: Pinned Specimen
- **Type status:** Other material. **Occurrence:** occurrenceDetails: http://janzen.sas.upenn.edu; catalogNumber: DHJPAR0008105; recordedBy: D.H. Janzen, W. Hallwachs & Lucia Rios; individualID: DHJPAR0008105; individualCount: 1; sex: female; lifeStage: adult; preparations: pinned; otherCatalogNumbers: ASTAT877-06, 98-SRNP-13639, BOLD:AAB0592; **Taxon:** scientificName: Hyphantrophaga eldaarayae; phylum: Arthropoda; class: Insecta; order: Diptera; family: Tachinidae; genus: Hyphantrophaga; specificEpithet: eldaarayae; scientificNameAuthorship: Fleming & Wood, 2018; **Location:** continent: Central America; country: Costa Rica; countryCode: CR; stateProvince: Guanacaste; county: Sector El Hacha; locality: Area de Conservacion Guanacaste; verbatimLocality: Sendero Bejuquilla; verbatimElevation: 280; verbatimLatitude: 11.03; verbatimLongitude: -85.527; verbatimCoordinateSystem: Decimal; decimalLatitude: 11.03; decimalLongitude: -85.527; **Identification:** identifiedBy: AJ Fleming; dateIdentified: 2017; **Event:** samplingProtocol: Reared from the larva of the Sphingidae, Xylophanes chiron; verbatimEventDate: 27-Oct-1998; **Record Level:** language: en; institutionCode: CNC; collectionCode: Insects; basisOfRecord: Pinned Specimen
- **Type status:** Other material. **Occurrence:** occurrenceDetails: http://janzen.sas.upenn.edu; catalogNumber: DHJPAR0008107; recordedBy: D.H. Janzen, W. Hallwachs & Jose Perez; individualID: DHJPAR0008107; individualCount: 1; sex: female; lifeStage: adult; preparations: pinned; otherCatalogNumbers: ASTAT879-06, 03-SRNP-11998,; **Taxon:** scientificName: Hyphantrophaga eldaarayae; phylum: Arthropoda; class: Insecta; order: Diptera; family: Tachinidae; genus: Hyphantrophaga; specificEpithet: eldaarayae; scientificNameAuthorship: Fleming & Wood, 2018; **Location:** continent: Central America; country: Costa Rica; countryCode: CR; stateProvince: Alajuela; county: Sector Rincon Rain Forest; locality: Area de Conservacion Guanacaste; verbatimLocality: San Lucas; verbatimElevation: 320; verbatimLatitude: 10.9185; verbatimLongitude: -85.3034; verbatimCoordinateSystem: Decimal; decimalLatitude: 10.9185; decimalLongitude: -85.3034; **Identification:** identifiedBy: AJ Fleming; dateIdentified: 2017; **Event:** samplingProtocol: Reared from the larva of the Sphingidae, Xylophanes chiron; verbatimEventDate: 02-Sep-2003; **Record Level:** language: en; institutionCode: CNC; collectionCode: Insects; basisOfRecord: Pinned Specimen
- **Type status:** Other material. **Occurrence:** occurrenceDetails: http://janzen.sas.upenn.edu; catalogNumber: DHJPAR0008109; recordedBy: D.H. Janzen, W. Hallwachs & gusaneros; individualID: DHJPAR0008109; individualCount: 1; sex: female; lifeStage: adult; preparations: pinned; otherCatalogNumbers: ASTAT881-06, 98-SRNP-12603, BOLD:AAB0592; **Taxon:** scientificName: Hyphantrophaga eldaarayae; phylum: Arthropoda; class: Insecta; order: Diptera; family: Tachinidae; genus: Hyphantrophaga; specificEpithet: eldaarayae; scientificNameAuthorship: Fleming & Wood, 2018; **Location:** continent: Central America; country: Costa Rica; countryCode: CR; stateProvince: Guanacaste; county: Sector Santa Rosa; locality: Area de Conservacion Guanacaste; verbatimLocality: Tanquetas; verbatimElevation: 295; verbatimLatitude: 10.8708; verbatimLongitude: -85.6053; verbatimCoordinateSystem: Decimal; decimalLatitude: 10.8708; decimalLongitude: -85.6053; **Identification:** identifiedBy: AJ Fleming; dateIdentified: 2017; **Event:** samplingProtocol: Reared from the larva of the Sphingidae, Xylophanes libyaDHJ02; verbatimEventDate: 27-Nov-1998; **Record Level:** language: en; institutionCode: CNC; collectionCode: Insects; basisOfRecord: Pinned Specimen
- **Type status:** Other material. **Occurrence:** occurrenceDetails: http://janzen.sas.upenn.edu; catalogNumber: DHJPAR0008110; recordedBy: D.H. Janzen, W. Hallwachs & Lucia Rios; individualID: DHJPAR0008110; individualCount: 1; sex: female; lifeStage: adult; preparations: pinned; otherCatalogNumbers: ASTAT882-06, 98-SRNP-14083,; **Taxon:** scientificName: Hyphantrophaga eldaarayae; phylum: Arthropoda; class: Insecta; order: Diptera; family: Tachinidae; genus: Hyphantrophaga; specificEpithet: eldaarayae; scientificNameAuthorship: Fleming & Wood, 2018; **Location:** continent: Central America; country: Costa Rica; countryCode: CR; stateProvince: Guanacaste; county: Sector El Hacha; locality: Area de Conservacion Guanacaste; verbatimLocality: Sendero Bejuquilla; verbatimElevation: 280; verbatimLatitude: 11.03; verbatimLongitude: -85.527; verbatimCoordinateSystem: Decimal; decimalLatitude: 11.03; decimalLongitude: -85.527; **Identification:** identifiedBy: AJ Fleming; dateIdentified: 2017; **Event:** samplingProtocol: Reared from the larva of the Sphingidae, Xylophanes chiron; verbatimEventDate: 13-Dec-1998; **Record Level:** language: en; institutionCode: CNC; collectionCode: Insects; basisOfRecord: Pinned Specimen
- **Type status:** Other material. **Occurrence:** occurrenceDetails: http://janzen.sas.upenn.edu; catalogNumber: DHJPAR0015010; recordedBy: D.H. Janzen, W. Hallwachs & Roster Moraga; individualID: DHJPAR0015010; individualCount: 1; sex: female; lifeStage: adult; preparations: pinned; otherCatalogNumbers: ASTAV701-06, 06-SRNP-21373,; **Taxon:** scientificName: Hyphantrophaga eldaarayae; phylum: Arthropoda; class: Insecta; order: Diptera; family: Tachinidae; genus: Hyphantrophaga; specificEpithet: eldaarayae; scientificNameAuthorship: Fleming & Wood, 2018; **Location:** continent: Central America; country: Costa Rica; countryCode: CR; stateProvince: Guanacaste; county: Sector Del Oro; locality: Area de Conservacion Guanacaste; verbatimLocality: San Antonio; verbatimElevation: 335; verbatimLatitude: 11.0353; verbatimLongitude: -85.4453; verbatimCoordinateSystem: Decimal; decimalLatitude: 11.0353; decimalLongitude: -85.4453; **Identification:** identifiedBy: AJ Fleming; dateIdentified: 2017; **Event:** samplingProtocol: Reared from the larva of the Sphingidae, Xylophanes chiron; verbatimEventDate: 05-Jul-2006; **Record Level:** language: en; institutionCode: CNC; collectionCode: Insects; basisOfRecord: Pinned Specimen
- **Type status:** Other material. **Occurrence:** occurrenceDetails: http://janzen.sas.upenn.edu; catalogNumber: DHJPAR0015260; recordedBy: D.H. Janzen, W. Hallwachs & Roster Moraga; individualID: DHJPAR0015260; individualCount: 1; sex: female; lifeStage: adult; preparations: pinned; otherCatalogNumbers: ASTAS015-06, 06-SRNP-21415, BOLD:AAB0592; **Taxon:** scientificName: Hyphantrophaga eldaarayae; phylum: Arthropoda; class: Insecta; order: Diptera; family: Tachinidae; genus: Hyphantrophaga; specificEpithet: eldaarayae; scientificNameAuthorship: Fleming & Wood, 2018; **Location:** continent: Central America; country: Costa Rica; countryCode: CR; stateProvince: Guanacaste; county: Sector Del Oro; locality: Area de Conservacion Guanacaste; verbatimLocality: Margarita; verbatimElevation: 380; verbatimLatitude: 11.0323; verbatimLongitude: -85.4395; verbatimCoordinateSystem: Decimal; decimalLatitude: 11.0323; decimalLongitude: -85.4395; **Identification:** identifiedBy: AJ Fleming; dateIdentified: 2017; **Event:** samplingProtocol: Reared from the larva of the Sphingidae, Xylophanes chiron; verbatimEventDate: 10-Jul-2006; **Record Level:** language: en; institutionCode: CNC; collectionCode: Insects; basisOfRecord: Pinned Specimen
- **Type status:** Other material. **Occurrence:** occurrenceDetails: http://janzen.sas.upenn.edu; catalogNumber: DHJPAR0010343; recordedBy: D.H. Janzen, W. Hallwachs & Carolina Cano; individualID: DHJPAR0010343; individualCount: 1; sex: female; lifeStage: adult; preparations: pinned; otherCatalogNumbers: ASTAS174-06, 06-SRNP-5715, BOLD:AAB0592; **Taxon:** scientificName: Hyphantrophaga eldaarayae; phylum: Arthropoda; class: Insecta; order: Diptera; family: Tachinidae; genus: Hyphantrophaga; specificEpithet: eldaarayae; scientificNameAuthorship: Fleming & Wood, 2018; **Location:** continent: Central America; country: Costa Rica; countryCode: CR; stateProvince: Alajuela; county: Sector San Cristobal; locality: Area de Conservacion Guanacaste; verbatimLocality: Vado Rio Cucaracho; verbatimElevation: 640; verbatimLatitude: 10.8702; verbatimLongitude: -85.3915; verbatimCoordinateSystem: Decimal; decimalLatitude: 10.8702; decimalLongitude: -85.3915; **Identification:** identifiedBy: AJ Fleming; dateIdentified: 2017; **Event:** samplingProtocol: Reared from the larva of the Sphingidae, Xylophanes jocasta; verbatimEventDate: 16-Aug-2006; **Record Level:** language: en; institutionCode: CNC; collectionCode: Insects; basisOfRecord: Pinned Specimen
- **Type status:** Other material. **Occurrence:** occurrenceDetails: http://janzen.sas.upenn.edu; catalogNumber: DHJPAR0016596; recordedBy: D.H. Janzen, W. Hallwachs & Jose Perez; individualID: DHJPAR0016596; individualCount: 1; sex: female; lifeStage: adult; preparations: pinned; otherCatalogNumbers: ASTAP800-07, 06-SRNP-43632, BOLD:AAB0592; **Taxon:** scientificName: Hyphantrophaga eldaarayae; phylum: Arthropoda; class: Insecta; order: Diptera; family: Tachinidae; genus: Hyphantrophaga; specificEpithet: eldaarayae; scientificNameAuthorship: Fleming & Wood, 2018; **Location:** continent: Central America; country: Costa Rica; countryCode: CR; stateProvince: Alajuela; county: Sector Rincon Rain Forest; locality: Area de Conservacion Guanacaste; verbatimLocality: Puente Rio Negro; verbatimElevation: 340; verbatimLatitude: 10.9038; verbatimLongitude: -85.3027; verbatimCoordinateSystem: Decimal; decimalLatitude: 10.9038; decimalLongitude: -85.3027; **Identification:** identifiedBy: AJ Fleming; dateIdentified: 2017; **Event:** samplingProtocol: Reared from the larva of the Sphingidae, Xylophanes chiron; verbatimEventDate: 22-Oct-2006; **Record Level:** language: en; institutionCode: CNC; collectionCode: Insects; basisOfRecord: Pinned Specimen
- **Type status:** Other material. **Occurrence:** occurrenceDetails: http://janzen.sas.upenn.edu; catalogNumber: DHJPAR0022019; recordedBy: D.H. Janzen, W. Hallwachs & Duvalier Briceno; individualID: DHJPAR0022019; individualCount: 1; sex: female; lifeStage: adult; preparations: pinned; otherCatalogNumbers: ASTAT1157-07, 07-SRNP-65316, BOLD:AAB0592; **Taxon:** scientificName: Hyphantrophaga eldaarayae; phylum: Arthropoda; class: Insecta; order: Diptera; family: Tachinidae; genus: Hyphantrophaga; specificEpithet: eldaarayae; scientificNameAuthorship: Fleming & Wood, 2018; **Location:** continent: Central America; country: Costa Rica; countryCode: CR; stateProvince: Alajuela; county: Brasilia; locality: Area de Conservacion Guanacaste; verbatimLocality: Piedrona; verbatimElevation: 340; verbatimLatitude: 11.0162; verbatimLongitude: -85.359; verbatimCoordinateSystem: Decimal; decimalLatitude: 11.0162; decimalLongitude: -85.359; **Identification:** identifiedBy: AJ Fleming; dateIdentified: 2017; **Event:** samplingProtocol: Reared from the larva of the Sphingidae, Xylophanes chiron; verbatimEventDate: 05-Sep-2007; **Record Level:** language: en; institutionCode: CNC; collectionCode: Insects; basisOfRecord: Pinned Specimen
- **Type status:** Other material. **Occurrence:** occurrenceDetails: http://janzen.sas.upenn.edu; catalogNumber: DHJPAR0022960; recordedBy: D.H. Janzen, W. Hallwachs & gusaneros; individualID: DHJPAR0022960; individualCount: 1; sex: female; lifeStage: adult; preparations: pinned; otherCatalogNumbers: ASTAW124-08, 86-SRNP-226,; **Taxon:** scientificName: Hyphantrophaga eldaarayae; phylum: Arthropoda; class: Insecta; order: Diptera; family: Tachinidae; genus: Hyphantrophaga; specificEpithet: eldaarayae; scientificNameAuthorship: Fleming & Wood, 2018; **Location:** continent: Central America; country: Costa Rica; countryCode: CR; stateProvince: Guanacaste; county: Sector Santa Rosa; locality: Area de Conservacion Guanacaste; verbatimLocality: Bosque Humedo; verbatimElevation: 290; verbatimLatitude: 10.8514; verbatimLongitude: -85.608; verbatimCoordinateSystem: Decimal; decimalLatitude: 10.8514; decimalLongitude: -85.608; **Identification:** identifiedBy: AJ Fleming; dateIdentified: 2017; **Event:** samplingProtocol: Reared from the larva of the Sphingidae, Xylophanes chiron; verbatimEventDate: 24-Jul-1986; **Record Level:** language: en; institutionCode: CNC; collectionCode: Insects; basisOfRecord: Pinned Specimen
- **Type status:** Other material. **Occurrence:** occurrenceDetails: http://janzen.sas.upenn.edu; catalogNumber: DHJPAR0022965; recordedBy: D.H. Janzen, W. Hallwachs & Osvaldo Espinoza; individualID: DHJPAR0022965; individualCount: 1; sex: female; lifeStage: adult; preparations: pinned; otherCatalogNumbers: ASTAW129-08, 01-SRNP-22178, BOLD:AAB0592; **Taxon:** scientificName: Hyphantrophaga eldaarayae; phylum: Arthropoda; class: Insecta; order: Diptera; family: Tachinidae; genus: Hyphantrophaga; specificEpithet: eldaarayae; scientificNameAuthorship: Fleming & Wood, 2018; **Location:** continent: Central America; country: Costa Rica; countryCode: CR; stateProvince: Alajuela; county: Sector San Cristobal; locality: Area de Conservacion Guanacaste; verbatimLocality: Rio Blanco Abajo; verbatimElevation: 500; verbatimLatitude: 10.9004; verbatimLongitude: -85.3725; verbatimCoordinateSystem: Decimal; decimalLatitude: 10.9004; decimalLongitude: -85.3725; **Identification:** identifiedBy: AJ Fleming; dateIdentified: 2017; **Event:** samplingProtocol: Reared from the larva of the Sphingidae, Xylophanes chiron; verbatimEventDate: 14-Dec-2001; **Record Level:** language: en; institutionCode: CNC; collectionCode: Insects; basisOfRecord: Pinned Specimen
- **Type status:** Other material. **Occurrence:** occurrenceDetails: http://janzen.sas.upenn.edu; catalogNumber: DHJPAR0022966; recordedBy: D.H. Janzen, W. Hallwachs & Osvaldo Espinoza; individualID: DHJPAR0022966; individualCount: 1; sex: female; lifeStage: adult; preparations: pinned; otherCatalogNumbers: ASTAW130-08, 01-SRNP-22178, BOLD:AAB0592; **Taxon:** scientificName: Hyphantrophaga eldaarayae; phylum: Arthropoda; class: Insecta; order: Diptera; family: Tachinidae; genus: Hyphantrophaga; specificEpithet: eldaarayae; scientificNameAuthorship: Fleming & Wood, 2018; **Location:** continent: Central America; country: Costa Rica; countryCode: CR; stateProvince: Alajuela; county: Sector San Cristobal; locality: Area de Conservacion Guanacaste; verbatimLocality: Rio Blanco Abajo; verbatimElevation: 500; verbatimLatitude: 10.9004; verbatimLongitude: -85.3725; verbatimCoordinateSystem: Decimal; decimalLatitude: 10.9004; decimalLongitude: -85.3725; **Identification:** identifiedBy: AJ Fleming; dateIdentified: 2017; **Event:** samplingProtocol: Reared from the larva of the Sphingidae, Xylophanes chiron; verbatimEventDate: 14-Dec-2001; **Record Level:** language: en; institutionCode: CNC; collectionCode: Insects; basisOfRecord: Pinned Specimen
- **Type status:** Other material. **Occurrence:** occurrenceDetails: http://janzen.sas.upenn.edu; catalogNumber: DHJPAR0027792; recordedBy: D.H. Janzen, W. Hallwachs & Lucia Vargas; individualID: DHJPAR0027792; individualCount: 1; sex: female; lifeStage: adult; preparations: pinned; otherCatalogNumbers: ASHYE029-08, 08-SRNP-13448, BOLD:AAB0592; **Taxon:** scientificName: Hyphantrophaga eldaarayae; phylum: Arthropoda; class: Insecta; order: Diptera; family: Tachinidae; genus: Hyphantrophaga; specificEpithet: eldaarayae; scientificNameAuthorship: Fleming & Wood, 2018; **Location:** continent: Central America; country: Costa Rica; countryCode: CR; stateProvince: Guanacaste; county: Sector Santa Rosa; locality: Area de Conservacion Guanacaste; verbatimLocality: Bosque San Emilio; verbatimElevation: 300; verbatimLatitude: 10.8439; verbatimLongitude: -85.6138; verbatimCoordinateSystem: Decimal; decimalLatitude: 10.8439; decimalLongitude: -85.6138; **Identification:** identifiedBy: AJ Fleming; dateIdentified: 2017; **Event:** samplingProtocol: Reared from the larva of the Sphingidae, Xylophanes chiron; verbatimEventDate: 12-Jun-2008; **Record Level:** language: en; institutionCode: CNC; collectionCode: Insects; basisOfRecord: Pinned Specimen
- **Type status:** Other material. **Occurrence:** occurrenceDetails: http://janzen.sas.upenn.edu; catalogNumber: DHJPAR0037476; recordedBy: D.H. Janzen, W. Hallwachs & Cirilo Umaña; individualID: DHJPAR0037476; individualCount: 1; sex: female; lifeStage: adult; preparations: pinned; otherCatalogNumbers: ASHYC4221-10, 10-SRNP-75043, BOLD:AAB0592; **Taxon:** scientificName: Hyphantrophaga eldaarayae; phylum: Arthropoda; class: Insecta; order: Diptera; family: Tachinidae; genus: Hyphantrophaga; specificEpithet: eldaarayae; scientificNameAuthorship: Fleming & Wood, 2018; **Location:** continent: Central America; country: Costa Rica; countryCode: CR; stateProvince: Alajuela; county: Sector Rincon Rain Forest; locality: Area de Conservacion Guanacaste; verbatimLocality: Estacion Llanura; verbatimElevation: 135; verbatimLatitude: 10.9333; verbatimLongitude: -85.2533; verbatimCoordinateSystem: Decimal; decimalLatitude: 10.9333; decimalLongitude: -85.2533; **Identification:** identifiedBy: AJ Fleming; dateIdentified: 2017; **Event:** samplingProtocol: Reared from the larva of the Sphingidae, Xylophanes chiron; verbatimEventDate: 13-Feb-2010; **Record Level:** language: en; institutionCode: CNC; collectionCode: Insects; basisOfRecord: Pinned Specimen
- **Type status:** Other material. **Occurrence:** occurrenceDetails: http://janzen.sas.upenn.edu; catalogNumber: DHJPAR0037658; recordedBy: D.H. Janzen, W. Hallwachs & Carolina Cano; individualID: DHJPAR0037658; individualCount: 1; sex: female; lifeStage: adult; preparations: pinned; otherCatalogNumbers: ASHYC4403-10, 07-SRNP-2058,; **Taxon:** scientificName: Hyphantrophaga eldaarayae; phylum: Arthropoda; class: Insecta; order: Diptera; family: Tachinidae; genus: Hyphantrophaga; specificEpithet: eldaarayae; scientificNameAuthorship: Fleming & Wood, 2018; **Location:** continent: Central America; country: Costa Rica; countryCode: CR; stateProvince: Alajuela; county: Sector San Cristobal; locality: Area de Conservacion Guanacaste; verbatimLocality: Vado Rio Cucaracho; verbatimElevation: 640; verbatimLatitude: 10.8702; verbatimLongitude: -85.3915; verbatimCoordinateSystem: Decimal; decimalLatitude: 10.8702; decimalLongitude: -85.3915; **Identification:** identifiedBy: AJ Fleming; dateIdentified: 2017; **Event:** samplingProtocol: Reared from the larva of the Sphingidae, Xylophanes chiron; verbatimEventDate: 25-Jul-2007; **Record Level:** language: en; institutionCode: CNC; collectionCode: Insects; basisOfRecord: Pinned Specimen
- **Type status:** Other material. **Occurrence:** occurrenceDetails: http://janzen.sas.upenn.edu; catalogNumber: DHJPAR0046567; recordedBy: D.H. Janzen, W. Hallwachs & Jose Cortez; individualID: DHJPAR0046567; individualCount: 1; sex: female; lifeStage: adult; preparations: pinned; otherCatalogNumbers: ACGBA740-12, 11-SRNP-57619,; **Taxon:** scientificName: Hyphantrophaga eldaarayae; phylum: Arthropoda; class: Insecta; order: Diptera; family: Tachinidae; genus: Hyphantrophaga; specificEpithet: eldaarayae; scientificNameAuthorship: Fleming & Wood, 2018; **Location:** continent: Central America; country: Costa Rica; countryCode: CR; stateProvince: Guanacaste; county: Sector Mundo Nuevo; locality: Area de Conservacion Guanacaste; verbatimLocality: Quebrada Tibio Perla; verbatimElevation: 330; verbatimLatitude: 10.7626; verbatimLongitude: -85.4298; verbatimCoordinateSystem: Decimal; decimalLatitude: 10.7626; decimalLongitude: -85.4298; **Identification:** identifiedBy: AJ Fleming; dateIdentified: 2017; **Event:** samplingProtocol: Reared from the larva of the Sphingidae, Xylophanes pluto; verbatimEventDate: 31-Jan-2012; **Record Level:** language: en; institutionCode: CNC; collectionCode: Insects; basisOfRecord: Pinned Specimen
- **Type status:** Other material. **Occurrence:** occurrenceDetails: http://janzen.sas.upenn.edu; catalogNumber: DHJPAR0048429; recordedBy: D.H. Janzen, W. Hallwachs & Anabelle Cordoba; individualID: DHJPAR0048429; individualCount: 1; sex: female; lifeStage: adult; preparations: pinned; otherCatalogNumbers: ACGBA1971-12, 12-SRNP-40735, BOLD:AAB0592; **Taxon:** scientificName: Hyphantrophaga eldaarayae; phylum: Arthropoda; class: Insecta; order: Diptera; family: Tachinidae; genus: Hyphantrophaga; specificEpithet: eldaarayae; scientificNameAuthorship: Fleming & Wood, 2018; **Location:** continent: Central America; country: Costa Rica; countryCode: CR; stateProvince: Alajuela; county: Sector Rincon Rain Forest; locality: Area de Conservacion Guanacaste; verbatimLocality: San Lucas; verbatimElevation: 320; verbatimLatitude: 10.9185; verbatimLongitude: -85.3034; verbatimCoordinateSystem: Decimal; decimalLatitude: 10.9185; decimalLongitude: -85.3034; **Identification:** identifiedBy: AJ Fleming; dateIdentified: 2017; **Event:** samplingProtocol: Reared from the larva of the Sphingidae, Xylophanes chiron; verbatimEventDate: 26-Mar-2012; **Record Level:** language: en; institutionCode: CNC; collectionCode: Insects; basisOfRecord: Pinned Specimen
- **Type status:** Other material. **Occurrence:** occurrenceDetails: http://janzen.sas.upenn.edu; catalogNumber: DHJPAR0050186; recordedBy: D.H. Janzen, W. Hallwachs & Ricardo Calero; individualID: DHJPAR0050186; individualCount: 1; sex: female; lifeStage: adult; preparations: pinned; otherCatalogNumbers: ACGAZ1500-12, 12-SRNP-71976, BOLD:AAB0592; **Taxon:** scientificName: Hyphantrophaga eldaarayae; phylum: Arthropoda; class: Insecta; order: Diptera; family: Tachinidae; genus: Hyphantrophaga; specificEpithet: eldaarayae; scientificNameAuthorship: Fleming & Wood, 2018; **Location:** continent: Central America; country: Costa Rica; countryCode: CR; stateProvince: Guanacaste; county: Sector Pitilla; locality: Area de Conservacion Guanacaste; verbatimLocality: Medrano; verbatimElevation: 380; verbatimLatitude: 11.016; verbatimLongitude: -85.3805; verbatimCoordinateSystem: Decimal; decimalLatitude: 11.016; decimalLongitude: -85.3805; **Identification:** identifiedBy: AJ Fleming; dateIdentified: 2017; **Event:** samplingProtocol: Reared from the larva of the Sphingidae, Xylophanes porcusDHJ03; verbatimEventDate: 13-Sep-2012; **Record Level:** language: en; institutionCode: CNC; collectionCode: Insects; basisOfRecord: Pinned Specimen
- **Type status:** Other material. **Occurrence:** occurrenceDetails: http://janzen.sas.upenn.edu; catalogNumber: DHJPAR0050631; recordedBy: D.H. Janzen, W. Hallwachs & Keiner Aragon; individualID: DHJPAR0050631; individualCount: 1; sex: female; lifeStage: adult; preparations: pinned; otherCatalogNumbers: ACGBA3223-13, 12-SRNP-68832, BOLD:AAB0592; **Taxon:** scientificName: Hyphantrophaga eldaarayae; phylum: Arthropoda; class: Insecta; order: Diptera; family: Tachinidae; genus: Hyphantrophaga; specificEpithet: eldaarayae; scientificNameAuthorship: Fleming & Wood, 2018; **Location:** continent: Central America; country: Costa Rica; countryCode: CR; stateProvince: Alajuela; county: Sector Rincon Rain Forest; locality: Area de Conservacion Guanacaste; verbatimLocality: Malaguenya; verbatimElevation: 221; verbatimLatitude: 10.9555; verbatimLongitude: -85.2838; verbatimCoordinateSystem: Decimal; decimalLatitude: 10.9555; decimalLongitude: -85.2838; **Identification:** identifiedBy: AJ Fleming; dateIdentified: 2017; **Event:** samplingProtocol: Reared from the larva of the Sphingidae, Xylophanes guianensis; verbatimEventDate: 20-Dec-2012; **Record Level:** language: en; institutionCode: CNC; collectionCode: Insects; basisOfRecord: Pinned Specimen
- **Type status:** Other material. **Occurrence:** occurrenceDetails: http://janzen.sas.upenn.edu; catalogNumber: DHJPAR0053416; recordedBy: D.H. Janzen, W. Hallwachs & Cirilo Umaña; individualID: DHJPAR0053416; individualCount: 1; sex: female; lifeStage: adult; preparations: pinned; otherCatalogNumbers: ASHYM2770-13, 13-SRNP-77374, BOLD:AAB0592; **Taxon:** scientificName: Hyphantrophaga eldaarayae; phylum: Arthropoda; class: Insecta; order: Diptera; family: Tachinidae; genus: Hyphantrophaga; specificEpithet: eldaarayae; scientificNameAuthorship: Fleming & Wood, 2018; **Location:** continent: Central America; country: Costa Rica; countryCode: CR; stateProvince: Alajuela; county: Sector Rincon Rain Forest; locality: Area de Conservacion Guanacaste; verbatimLocality: Finca Esmeralda; verbatimElevation: 123; verbatimLatitude: 10.9355; verbatimLongitude: -85.2531; verbatimCoordinateSystem: Decimal; decimalLatitude: 10.9355; decimalLongitude: -85.2531; **Identification:** identifiedBy: AJ Fleming; dateIdentified: 2017; **Event:** samplingProtocol: Reared from the larva of the Sphingidae, Xylophanes chiron; verbatimEventDate: 21-Sep-2013; **Record Level:** language: en; institutionCode: CNC; collectionCode: Insects; basisOfRecord: Pinned Specimen
- **Type status:** Other material. **Occurrence:** occurrenceDetails: http://janzen.sas.upenn.edu; catalogNumber: DHJPAR0056192; recordedBy: D.H. Janzen, W. Hallwachs & Pablo Umaña Calderon; individualID: DHJPAR0056192; individualCount: 1; sex: female; lifeStage: adult; preparations: pinned; otherCatalogNumbers: ASHYH2449-14, 14-SRNP-43786,; **Taxon:** scientificName: Hyphantrophaga eldaarayae; phylum: Arthropoda; class: Insecta; order: Diptera; family: Tachinidae; genus: Hyphantrophaga; specificEpithet: eldaarayae; scientificNameAuthorship: Fleming & Wood, 2018; **Location:** continent: Central America; country: Costa Rica; countryCode: CR; stateProvince: Alajuela; county: Sector Rincon Rain Forest; locality: Area de Conservacion Guanacaste; verbatimLocality: Sendero Venado; verbatimElevation: 420; verbatimLatitude: 10.8968; verbatimLongitude: -85.27; verbatimCoordinateSystem: Decimal; decimalLatitude: 10.8968; decimalLongitude: -85.27; **Identification:** identifiedBy: AJ Fleming; dateIdentified: 2017; **Event:** samplingProtocol: Reared from the larva of the Sphingidae, Xylophanes chiron; verbatimEventDate: 26-Sep-2014; **Record Level:** language: en; institutionCode: CNC; collectionCode: Insects; basisOfRecord: Pinned Specimen
- **Type status:** Other material. **Occurrence:** occurrenceDetails: http://janzen.sas.upenn.edu; catalogNumber: DHJPAR0057035; recordedBy: D.H. Janzen, W. Hallwachs & Manuel Rios; individualID: DHJPAR0057035; individualCount: 1; sex: female; lifeStage: adult; preparations: pinned; otherCatalogNumbers: ACGBA4945-15, 15-SRNP-30018, BOLD:AAB0592; **Taxon:** scientificName: Hyphantrophaga eldaarayae; phylum: Arthropoda; class: Insecta; order: Diptera; family: Tachinidae; genus: Hyphantrophaga; specificEpithet: eldaarayae; scientificNameAuthorship: Fleming & Wood, 2018; **Location:** continent: Central America; country: Costa Rica; countryCode: CR; stateProvince: Guanacaste; county: Sector Pitilla; locality: Area de Conservacion Guanacaste; verbatimLocality: Pasmompa; verbatimElevation: 440; verbatimLatitude: 11.0193; verbatimLongitude: -85.41; verbatimCoordinateSystem: Decimal; decimalLatitude: 11.0193; decimalLongitude: -85.41; **Identification:** identifiedBy: AJ Fleming; dateIdentified: 2017; **Event:** samplingProtocol: Reared from the larva of the Sphingidae, Xylophanes loelia; verbatimEventDate: 20-Feb-2015; **Record Level:** language: en; institutionCode: CNC; collectionCode: Insects; basisOfRecord: Pinned Specimen
- **Type status:** Other material. **Occurrence:** occurrenceDetails: http://janzen.sas.upenn.edu; catalogNumber: DHJPAR0057248; recordedBy: D.H. Janzen, W. Hallwachs & Cirilo Umaña; individualID: DHJPAR0057248; individualCount: 1; sex: female; lifeStage: adult; preparations: pinned; otherCatalogNumbers: ACGBA5158-15, 15-SRNP-75070, BOLD:AAB0592; **Taxon:** scientificName: Hyphantrophaga eldaarayae; phylum: Arthropoda; class: Insecta; order: Diptera; family: Tachinidae; genus: Hyphantrophaga; specificEpithet: eldaarayae; scientificNameAuthorship: Fleming & Wood, 2018; **Location:** continent: Central America; country: Costa Rica; countryCode: CR; stateProvince: Alajuela; county: Sector Rincon Rain Forest; locality: Area de Conservacion Guanacaste; verbatimLocality: Finca Esmeralda; verbatimElevation: 123; verbatimLatitude: 10.9355; verbatimLongitude: -85.2531; verbatimCoordinateSystem: Decimal; decimalLatitude: 10.9355; decimalLongitude: -85.2531; **Identification:** identifiedBy: AJ Fleming; dateIdentified: 2017; **Event:** samplingProtocol: Reared from the larva of the Sphingidae, Xylophanes chiron; verbatimEventDate: 03-Feb-2015; **Record Level:** language: en; institutionCode: CNC; collectionCode: Insects; basisOfRecord: Pinned Specimen
- **Type status:** Other material. **Occurrence:** occurrenceDetails: http://janzen.sas.upenn.edu; catalogNumber: DHJPAR0008078; recordedBy: D.H. Janzen, W. Hallwachs & Minor Carmona; individualID: DHJPAR0008078; individualCount: 1; lifeStage: adult; preparations: pinned; otherCatalogNumbers: ASTAT850-06, 03-SRNP-11723, BOLD:AAB0592; **Taxon:** scientificName: Hyphantrophaga eldaarayae; phylum: Arthropoda; class: Insecta; order: Diptera; family: Tachinidae; genus: Hyphantrophaga; specificEpithet: eldaarayae; scientificNameAuthorship: Fleming & Wood, 2018; **Location:** continent: Central America; country: Costa Rica; countryCode: CR; stateProvince: Alajuela; county: Sector Rincon Rain Forest; locality: Area de Conservacion Guanacaste; verbatimLocality: San Lucas; verbatimElevation: 320; verbatimLatitude: 10.9185; verbatimLongitude: -85.3034; verbatimCoordinateSystem: Decimal; decimalLatitude: 10.9185; decimalLongitude: -85.3034; **Identification:** identifiedBy: AJ Fleming; dateIdentified: 2017; **Event:** samplingProtocol: Reared from the larva of the Sphingidae, Xylophanes chiron; verbatimEventDate: 18-Aug-2003; **Record Level:** language: en; institutionCode: CNC; collectionCode: Insects; basisOfRecord: Pinned Specimen
- **Type status:** Other material. **Occurrence:** occurrenceDetails: http://janzen.sas.upenn.edu; catalogNumber: DHJPAR0008106; recordedBy: D.H. Janzen, W. Hallwachs & Lucia Rios; individualID: DHJPAR0008106; individualCount: 1; lifeStage: adult; preparations: pinned; otherCatalogNumbers: ASTAT878-06, 98-SRNP-13986, BOLD:AAB0592; **Taxon:** scientificName: Hyphantrophaga eldaarayae; phylum: Arthropoda; class: Insecta; order: Diptera; family: Tachinidae; genus: Hyphantrophaga; specificEpithet: eldaarayae; scientificNameAuthorship: Fleming & Wood, 2018; **Location:** continent: Central America; country: Costa Rica; countryCode: CR; stateProvince: Guanacaste; county: Sector El Hacha; locality: Area de Conservacion Guanacaste; verbatimLocality: Sendero Bejuquilla; verbatimElevation: 280; verbatimLatitude: 11.03; verbatimLongitude: -85.527; verbatimCoordinateSystem: Decimal; decimalLatitude: 11.03; decimalLongitude: -85.527; **Identification:** identifiedBy: AJ Fleming; dateIdentified: 2017; **Event:** samplingProtocol: Reared from the larva of the Sphingidae, Xylophanes chiron; verbatimEventDate: 12-Mar-1998; **Record Level:** language: en; institutionCode: CNC; collectionCode: Insects; basisOfRecord: Pinned Specimen
- **Type status:** Other material. **Occurrence:** occurrenceDetails: http://janzen.sas.upenn.edu; catalogNumber: DHJPAR0006956; recordedBy: D.H. Janzen, W. Hallwachs & Minor Carmona; individualID: DHJPAR0006956; individualCount: 1; lifeStage: adult; preparations: pinned; otherCatalogNumbers: ASTAV198-06, 06-SRNP-40507,; **Taxon:** scientificName: Hyphantrophaga eldaarayae; phylum: Arthropoda; class: Insecta; order: Diptera; family: Tachinidae; genus: Hyphantrophaga; specificEpithet: eldaarayae; scientificNameAuthorship: Fleming & Wood, 2018; **Location:** continent: Central America; country: Costa Rica; countryCode: CR; stateProvince: Alajuela; county: Sector Rincon Rain Forest; locality: Area de Conservacion Guanacaste; verbatimLocality: Finca Aurita; verbatimElevation: 460; verbatimLatitude: 10.8841; verbatimLongitude: -85.2573; verbatimCoordinateSystem: Decimal; decimalLatitude: 10.8841; decimalLongitude: -85.2573; **Identification:** identifiedBy: AJ Fleming; dateIdentified: 2017; **Event:** samplingProtocol: Reared from the larva of the Sphingidae, Xylophanes chiron; verbatimEventDate: 17-Mar-2006; **Record Level:** language: en; institutionCode: CNC; collectionCode: Insects; basisOfRecord: Pinned Specimen
- **Type status:** Other material. **Occurrence:** occurrenceDetails: http://janzen.sas.upenn.edu; catalogNumber: DHJPAR0019719; recordedBy: D.H. Janzen, W. Hallwachs & Carolina Cano; individualID: DHJPAR0019719; individualCount: 1; lifeStage: adult; preparations: pinned; otherCatalogNumbers: ASTAB267-07, 07-SRNP-2058,; **Taxon:** scientificName: Hyphantrophaga eldaarayae; phylum: Arthropoda; class: Insecta; order: Diptera; family: Tachinidae; genus: Hyphantrophaga; specificEpithet: eldaarayae; scientificNameAuthorship: Fleming & Wood, 2018; **Location:** continent: Central America; country: Costa Rica; countryCode: CR; stateProvince: Alajuela; county: Sector San Cristobal; locality: Area de Conservacion Guanacaste; verbatimLocality: Vado Rio Cucaracho; verbatimElevation: 640; verbatimLatitude: 10.8702; verbatimLongitude: -85.3915; verbatimCoordinateSystem: Decimal; decimalLatitude: 10.8702; decimalLongitude: -85.3915; **Identification:** identifiedBy: AJ Fleming; dateIdentified: 2017; **Event:** samplingProtocol: Reared from the larva of the Sphingidae, Xylophanes chiron; verbatimEventDate: 25-Jul-2007; **Record Level:** language: en; institutionCode: CNC; collectionCode: Insects; basisOfRecord: Pinned Specimen
- **Type status:** Other material. **Occurrence:** occurrenceDetails: http://janzen.sas.upenn.edu; catalogNumber: DHJPAR0029647; recordedBy: D.H. Janzen, W. Hallwachs & Elda Araya; individualID: DHJPAR0029647; individualCount: 1; lifeStage: adult; preparations: pinned; otherCatalogNumbers: ASHYM1068-09, 08-SRNP-5331,; **Taxon:** scientificName: Hyphantrophaga eldaarayae; phylum: Arthropoda; class: Insecta; order: Diptera; family: Tachinidae; genus: Hyphantrophaga; specificEpithet: eldaarayae; scientificNameAuthorship: Fleming & Wood, 2018; **Location:** continent: Central America; country: Costa Rica; countryCode: CR; stateProvince: Alajuela; county: Buenos Aires; locality: Area de Conservacion Guanacaste; verbatimLocality: Finca Tomate; verbatimElevation: 360; verbatimLatitude: 10.9035; verbatimLongitude: -85.3092; verbatimCoordinateSystem: Decimal; decimalLatitude: 10.9035; decimalLongitude: -85.3092; **Identification:** identifiedBy: AJ Fleming; dateIdentified: 2017; **Event:** samplingProtocol: Reared from the larva of the Sphingidae, Xylophanes chiron; verbatimEventDate: 19-Oct-2008; **Record Level:** language: en; institutionCode: CNC; collectionCode: Insects; basisOfRecord: Pinned Specimen
- **Type status:** Other material. **Occurrence:** occurrenceDetails: http://janzen.sas.upenn.edu; catalogNumber: DHJPAR0040932; recordedBy: D.H. Janzen, W. Hallwachs & Gloria Sihezar; individualID: DHJPAR0040932; individualCount: 1; lifeStage: adult; preparations: pinned; otherCatalogNumbers: ASHYF847-11, 10-SRNP-6265, BOLD:AAB0592; **Taxon:** scientificName: Hyphantrophaga eldaarayae; phylum: Arthropoda; class: Insecta; order: Diptera; family: Tachinidae; genus: Hyphantrophaga; specificEpithet: eldaarayae; scientificNameAuthorship: Fleming & Wood, 2018; **Location:** continent: Central America; country: Costa Rica; countryCode: CR; stateProvince: Alajuela; county: Sector San Cristobal; locality: Area de Conservacion Guanacaste; verbatimLocality: Tajo Angeles; verbatimElevation: 540; verbatimLatitude: 10.8647; verbatimLongitude: -85.4153; verbatimCoordinateSystem: Decimal; decimalLatitude: 10.8647; decimalLongitude: -85.4153; **Identification:** identifiedBy: AJ Fleming; dateIdentified: 2017; **Event:** samplingProtocol: Reared from the larva of the Sphingidae, Xylophanes chiron; verbatimEventDate: 26-Nov-2010; **Record Level:** language: en; institutionCode: CNC; collectionCode: Insects; basisOfRecord: Pinned Specimen
- **Type status:** Other material. **Occurrence:** occurrenceDetails: http://janzen.sas.upenn.edu; catalogNumber: DHJPAR0042146; recordedBy: D.H. Janzen, W. Hallwachs & Gloria Sihezar; individualID: DHJPAR0042146; individualCount: 1; lifeStage: adult; preparations: pinned; otherCatalogNumbers: ASHYF2034-11, 10-SRNP-6265, BOLD:AAB0592; **Taxon:** scientificName: Hyphantrophaga eldaarayae; phylum: Arthropoda; class: Insecta; order: Diptera; family: Tachinidae; genus: Hyphantrophaga; specificEpithet: eldaarayae; scientificNameAuthorship: Fleming & Wood, 2018; **Location:** continent: Central America; country: Costa Rica; countryCode: CR; stateProvince: Alajuela; county: Sector San Cristobal; locality: Area de Conservacion Guanacaste; verbatimLocality: Tajo Angeles; verbatimElevation: 540; verbatimLatitude: 10.8647; verbatimLongitude: -85.4153; verbatimCoordinateSystem: Decimal; decimalLatitude: 10.8647; decimalLongitude: -85.4153; **Identification:** identifiedBy: AJ Fleming; dateIdentified: 2017; **Event:** samplingProtocol: Reared from the larva of the Sphingidae, Xylophanes chiron; verbatimEventDate: 26-Nov-2010; **Record Level:** language: en; institutionCode: CNC; collectionCode: Insects; basisOfRecord: Pinned Specimen
- **Type status:** Other material. **Occurrence:** occurrenceDetails: http://janzen.sas.upenn.edu; catalogNumber: DHJPAR0055641; recordedBy: D.H. Janzen, W. Hallwachs & Manuel Rios; individualID: DHJPAR0055641; individualCount: 1; lifeStage: adult; preparations: pinned; otherCatalogNumbers: ASHYH2188-14, 14-SRNP-30344, BOLD:AAB0592; **Taxon:** scientificName: Hyphantrophaga eldaarayae; phylum: Arthropoda; class: Insecta; order: Diptera; family: Tachinidae; genus: Hyphantrophaga; specificEpithet: eldaarayae; scientificNameAuthorship: Fleming & Wood, 2018; **Location:** continent: Central America; country: Costa Rica; countryCode: CR; stateProvince: Guanacaste; county: Sector Pitilla; locality: Area de Conservacion Guanacaste; verbatimLocality: Sendero Naciente; verbatimElevation: 700; verbatimLatitude: 10.9871; verbatimLongitude: -85.4282; verbatimCoordinateSystem: Decimal; decimalLatitude: 10.9871; decimalLongitude: -85.4282; **Identification:** identifiedBy: AJ Fleming; dateIdentified: 2017; **Event:** samplingProtocol: Reared from the larva of the Sphingidae, Xylophanes germen; verbatimEventDate: 04-Apr-2014; **Record Level:** language: en; institutionCode: CNC; collectionCode: Insects; basisOfRecord: Pinned Specimen
- **Type status:** Other material. **Occurrence:** occurrenceDetails: http://janzen.sas.upenn.edu; catalogNumber: DHJPAR0061283; recordedBy: D.H. Janzen, W. Hallwachs & Ricardo Calero; individualID: DHJPAR0061283; individualCount: 1; lifeStage: adult; preparations: pinned; otherCatalogNumbers: ACGBA7666-17, 17-SRNP-72587, BOLD:AAB0592; **Taxon:** scientificName: Hyphantrophaga eldaarayae; phylum: Arthropoda; class: Insecta; order: Diptera; family: Tachinidae; genus: Hyphantrophaga; specificEpithet: eldaarayae; scientificNameAuthorship: Fleming & Wood, 2018; **Location:** continent: Central America; country: Costa Rica; countryCode: CR; stateProvince: Guanacaste; county: Sector Pitilla; locality: Area de Conservacion Guanacaste; verbatimLocality: Medrano; verbatimElevation: 380; verbatimLatitude: 11.016; verbatimLongitude: -85.3805; verbatimCoordinateSystem: Decimal; decimalLatitude: 11.016; decimalLongitude: -85.3805; **Identification:** identifiedBy: AJ Fleming; dateIdentified: 2017; **Event:** samplingProtocol: Reared from the larva of the Sphingidae, Xylophanes chiron; verbatimEventDate: 21-Aug-2017; **Record Level:** language: en; institutionCode: CNC; collectionCode: Insects; basisOfRecord: Pinned Specimen

#### Description

**Male** (Fig. 13). Length: 9–12 mm. **Head** (Fig. 13b): vertex 1/5 of head width; two pairs of reclinate upper orbital setae; ocellar setae arising behind anterior ocellus; ocellar triangle pale brassy; fronto-orbital plate pale brassy on upper 80%, densely setulose, setulae not extending below lowest frontal seta; parafacial silver and bare; eye densely haired; facial ridge bare; pedicel black, concolorous with postpedicel; arista black, very minutely pubescent, distinctly thickened on basal 1/3–1/4; palpus yellow and haired apically, narrow and digitiform, apically pointed. **Thorax** (Fig. 13a, c): dull brassy tomentose dorsally, contrasting with slightly brighter silver tomentose laterally, lateral surfaces with dense dark setulae making it appear darker; four thick dorsal vittae, outermost two broken across suture, innermost pair unbroken, reaching 3rd postsutural dorsocentral seta, both pairs of vittae not widening postsuturally; postpronotum with 3–5 setae arranged in a triangle; chaetotaxy: acrostichal setae 3:3; dorsocentral setae 3:4; intra-alar setae 3–4:3; supra-alar setae 2:3; three katepisternal setae; basal scutellar setae as long as subapical scutellar setae, curving slightly inwards medially; lateral scutellar setae less than 1/2 as long as subapical setae, curving inwards medially; apical scutellar setae subequal in length to lateral scutellar setae, crossed apically, angled slightly above plane of remaining marginal scutellar setae; one pair of discal scutellar setae more widely set than apical setae; scutellum very slightly darkened across basal 15%, shaped as two adjacent crescents, remainder concolorous with scutum. **Legs** (Fig. 13c): black in ground colour; fore femur with dense silver tomentum on posterodorsal surface; hind coxa bare. **Wing** (Fig. 13a): pale translucent, hyaline, not distinctly infuscate; vein R_4+5_ with only 2–3 setulae at base. **Abdomen** (Fig. 13a, c): ground colour black dorsally, yellow lateroventrally; mid-dorsal depression on ST1+2 reaching hind margin; median marginal setae present on ST1+2–T3; a complete row of marginal setae present on T4; discal setae only on T5; sex patch covering ventral surfaces of T4–T5; distinct brassy tomentose bands along anterior edge of T3 and T4, broken medially by a dorsocentral stripe and covering almost 80% of tergites; T3 with silver tomentum ventrolaterally over 80% of surface; T5 with brassy tomentum throughout. **Terminalia**: sternite 5 (Fig. 13f) with a deeply excavated median cleft, widely U-shaped, margins covered in dense tomentum. Lateral lobes of sternite rounded apically, many stout setae confined to lobe margins. Anterior plate of sternite 5 subequal to length of apical lobes; unsclerotised "window" anterior to median cleft reduced to a narrow strip as wide as median cleft. Cerci in posterior view (Fig. 13d) spatulate and rounded apically, slightly longer than surstyli, completely separate medially, slightly divergent; in lateral view with an evenly rounded downward curve throughout; densely setulose along basal 2/3. Surstylus in lateral view (Fig. 13e) round, tapered along its length and straight along bottom edge, opposite edge evenly rounded giving it a cleaver blade appearance; when viewed dorsally, surstyli pointing outwards and not strongly convergent. Pregonite short, not well developed, 1/2 as long as distiphallus, bare and rounded apically, having the appearance of an upside-down boot. Postgonite slightly narrow, 1/3 as wide as pregonite, sharply pointed and curved at apex. Epiphallus well-developed and apically hooked. Distiphallus rectangular with a slender median longitudinal sclerotised reinforcement on its posterior surface and a broad, anterolateral, sclerotised acrophallus on each side, joining on anterior surface near apex.

**Female**. Length: 8–11 mm. As male, differing only by the presence of two pairs of proclinate orbital setae.

#### Diagnosis

*Hyphantrophaga eldaarayae* **sp. n.** can be distinguished from all other *Hyphantrophaga* species by the following combination of traits: thorax with three postsutural acrostichal setae, four postsutural dorsocentral setae, and three katepisternal setae, legs black, hind coxa bare, silver tomentum covering more than 80% of ventrolateral surfaces of T3, with silver tomentum reaching edge of tergite ventrally.

#### Etymology

*Hyphantrophaga eldaarayae* **sp. n.** is named in recognition of Elda Araya Martinez's dedication and work in finding and rearing the ACG caterpillars that contained tachinid larvae.

#### Distribution

Costa Rica, ACG, Guanacaste Province, 380–420 m elevation.

#### Ecology

*Hyphantrophaga eldaarayae* **sp. n.** has been reared 116 times from 15 species of Lepidoptera in the family Sphingidae, *Xylophanes chiron* (Drury, 1773), *Xylophanes anubus* (Cramer, 1777), *Xylophanes tyndarus* (Boisduval, 1875), *Xylophanes* libyaDHJ02, *Xylophanes pluto* (Fabricius, 1777), *Xylophanes crotonis* (Walker, 1856), *Xylophanes maculator* (Boisduval, 1875), *Xylophanes jocasta* (Druce, 1888), *Xylophanes guianensis* (Rothschild, 1894), *Xylophanes hannemanni* Closs, 1917, *Xylophanes* porcusDHJ03, *Xylophanes germen* (Schaus, 1890), *Xylophanes cthulhu* Haxaire & Vaglia, 2008, *Xylophanes loelia* (Druce, 1878) and *Xylophanes porcus* (Hübner, 1823); in rain forest and dry-rain lowland intergrades.

### Hyphantrophaga eliethcantillanoae

Fleming & Wood sp. n.

urn:lsid:zoobank.org:act:2DB211E5-CB8B-4821-9DF6-4341A2E13252

#### Materials

- **Type status:** Holotype. **Occurrence:** occurrenceDetails: http://janzen.sas.upenn.edu; catalogNumber: DHJPAR0015985; recordedBy: D.H. Janzen, W. Hallwachs & Mariano Pereira; individualID: DHJPAR0015985; individualCount: 1; sex: male; lifeStage: adult; preparations: pinned; otherCatalogNumbers: ASTAP014-06, 06-SRNP-57540, BOLD:AAA1698; **Taxon:** scientificName: Hyphantrophaga eliethcantillanoae; phylum: Arthropoda; class: Insecta; order: Diptera; family: Tachinidae; genus: Hyphantrophaga; specificEpithet: eliethcantillanoae; scientificNameAuthorship: Fleming & Wood, 2017; **Location:** continent: Central America; country: Costa Rica; countryCode: CR; stateProvince: Guanacaste; county: Sector Mundo Nuevo; locality: Area de Conservacion Guanacaste; verbatimLocality: Quebrada Tibio Perla; verbatimElevation: 330; verbatimLatitude: 10.7626; verbatimLongitude: -85.4298; verbatimCoordinateSystem: Decimal; decimalLatitude: 10.7626; decimalLongitude: -85.4298; **Identification:** identifiedBy: AJ Fleming; dateIdentified: 2017; **Event:** samplingProtocol: Reared from the larva of the Crambidae, Omiodes cuniculalis; verbatimEventDate: 02-Sep-2006; **Record Level:** language: en; institutionCode: CNC; collectionCode: Insects; basisOfRecord: Pinned Specimen
- **Type status:** Paratype. **Occurrence:** occurrenceDetails: http://janzen.sas.upenn.edu; catalogNumber: DHJPAR0016011; recordedBy: D.H. Janzen, W. Hallwachs & Mariano Pereira; individualID: DHJPAR0016011; individualCount: 1; sex: male; lifeStage: adult; preparations: pinned; otherCatalogNumbers: ASTAP040-06, 06-SRNP-57589, BOLD:AAA1698; **Taxon:** scientificName: Hyphantrophaga eliethcantillanoae; phylum: Arthropoda; class: Insecta; order: Diptera; family: Tachinidae; genus: Hyphantrophaga; specificEpithet: eliethcantillanoae; scientificNameAuthorship: Fleming & Wood, 2017; **Location:** continent: Central America; country: Costa Rica; countryCode: CR; stateProvince: Guanacaste; county: Sector Mundo Nuevo; locality: Area de Conservacion Guanacaste; verbatimLocality: Quebrada Tibio Perla; verbatimElevation: 330; verbatimLatitude: 10.7626; verbatimLongitude: -85.4298; verbatimCoordinateSystem: Decimal; decimalLatitude: 10.7626; decimalLongitude: -85.4298; **Identification:** identifiedBy: AJ Fleming; dateIdentified: 2017; **Event:** samplingProtocol: Reared from the larva of the Crambidae, Omiodes cuniculalis; verbatimEventDate: 31-Aug-2006; **Record Level:** language: en; institutionCode: CNC; collectionCode: Insects; basisOfRecord: Pinned Specimen
- **Type status:** Paratype. **Occurrence:** occurrenceDetails: http://janzen.sas.upenn.edu; catalogNumber: DHJPAR0048627; recordedBy: D.H. Janzen, W. Hallwachs & Freddy Quesada; individualID: DHJPAR0048627; individualCount: 1; sex: male; lifeStage: adult; preparations: pinned; otherCatalogNumbers: ACGBA2169-12, 12-SRNP-70590,; **Taxon:** scientificName: Hyphantrophaga eliethcantillanoae; phylum: Arthropoda; class: Insecta; order: Diptera; family: Tachinidae; genus: Hyphantrophaga; specificEpithet: eliethcantillanoae; scientificNameAuthorship: Fleming & Wood, 2017; **Location:** continent: Central America; country: Costa Rica; countryCode: CR; stateProvince: Guanacaste; county: Sector Pitilla; locality: Area de Conservacion Guanacaste; verbatimLocality: Quebradona; verbatimElevation: 475; verbatimLatitude: 10.991; verbatimLongitude: -85.3954; verbatimCoordinateSystem: Decimal; decimalLatitude: 10.991; decimalLongitude: -85.3954; **Identification:** identifiedBy: AJ Fleming; dateIdentified: 2017; **Event:** samplingProtocol: Reared from the larva of the Crambidae, Omiodes fulvicauda; verbatimEventDate: 12-Apr-2012; **Record Level:** language: en; institutionCode: CNC; collectionCode: Insects; basisOfRecord: Pinned Specimen
- **Type status:** Paratype. **Occurrence:** occurrenceDetails: http://janzen.sas.upenn.edu; catalogNumber: DHJPAR0015991; recordedBy: D.H. Janzen, W. Hallwachs & Mariano Pereira; individualID: DHJPAR0015991; individualCount: 1; sex: female; lifeStage: adult; preparations: pinned; otherCatalogNumbers: ASTAP020-06, 06-SRNP-57542, BOLD:AAA1698; **Taxon:** scientificName: Hyphantrophaga eliethcantillanoae; phylum: Arthropoda; class: Insecta; order: Diptera; family: Tachinidae; genus: Hyphantrophaga; specificEpithet: eliethcantillanoae; scientificNameAuthorship: Fleming & Wood, 2017; **Location:** continent: Central America; country: Costa Rica; countryCode: CR; stateProvince: Guanacaste; county: Sector Mundo Nuevo; locality: Area de Conservacion Guanacaste; verbatimLocality: Quebrada Tibio Perla; verbatimElevation: 330; verbatimLatitude: 10.7626; verbatimLongitude: -85.4298; verbatimCoordinateSystem: Decimal; decimalLatitude: 10.7626; decimalLongitude: -85.4298; **Identification:** identifiedBy: AJ Fleming; dateIdentified: 2017; **Event:** samplingProtocol: Reared from the larva of the Crambidae, Omiodes cuniculalis; verbatimEventDate: 02-Sep-2006; **Record Level:** language: en; institutionCode: CNC; collectionCode: Insects; basisOfRecord: Pinned Specimen
- **Type status:** Paratype. **Occurrence:** occurrenceDetails: http://janzen.sas.upenn.edu; catalogNumber: DHJPAR0015979; recordedBy: D.H. Janzen, W. Hallwachs & Mariano Pereira; individualID: DHJPAR0015979; individualCount: 1; sex: female; lifeStage: adult; preparations: pinned; otherCatalogNumbers: ASTAP008-06, 06-SRNP-57601, BOLD:AAA1698; **Taxon:** scientificName: Hyphantrophaga eliethcantillanoae; phylum: Arthropoda; class: Insecta; order: Diptera; family: Tachinidae; genus: Hyphantrophaga; specificEpithet: eliethcantillanoae; scientificNameAuthorship: Fleming & Wood, 2017; **Location:** continent: Central America; country: Costa Rica; countryCode: CR; stateProvince: Guanacaste; county: Sector Mundo Nuevo; locality: Area de Conservacion Guanacaste; verbatimLocality: Quebrada Tibio Perla; verbatimElevation: 330; verbatimLatitude: 10.7626; verbatimLongitude: -85.4298; verbatimCoordinateSystem: Decimal; decimalLatitude: 10.7626; decimalLongitude: -85.4298; **Identification:** identifiedBy: AJ Fleming; dateIdentified: 2017; **Event:** samplingProtocol: Reared from the larva of the Crambidae, Omiodes cuniculalis; verbatimEventDate: 02-Sep-2006; **Record Level:** language: en; institutionCode: CNC; collectionCode: Insects; basisOfRecord: Pinned Specimen
- **Type status:** Paratype. **Occurrence:** occurrenceDetails: http://janzen.sas.upenn.edu; catalogNumber: DHJPAR0016002; recordedBy: D.H. Janzen, W. Hallwachs & Mariano Pereira; individualID: DHJPAR0016002; individualCount: 1; sex: female; lifeStage: adult; preparations: pinned; otherCatalogNumbers: ASTAP031-06, 06-SRNP-57575, BOLD:AAA1698; **Taxon:** scientificName: Hyphantrophaga eliethcantillanoae; phylum: Arthropoda; class: Insecta; order: Diptera; family: Tachinidae; genus: Hyphantrophaga; specificEpithet: eliethcantillanoae; scientificNameAuthorship: Fleming & Wood, 2017; **Location:** continent: Central America; country: Costa Rica; countryCode: CR; stateProvince: Guanacaste; county: Sector Mundo Nuevo; locality: Area de Conservacion Guanacaste; verbatimLocality: Quebrada Tibio Perla; verbatimElevation: 330; verbatimLatitude: 10.7626; verbatimLongitude: -85.4298; verbatimCoordinateSystem: Decimal; decimalLatitude: 10.7626; decimalLongitude: -85.4298; **Identification:** identifiedBy: AJ Fleming; dateIdentified: 2017; **Event:** samplingProtocol: Reared from the larva of the Crambidae, Omiodes cuniculalis; verbatimEventDate: 01-Sep-2006; **Record Level:** language: en; institutionCode: CNC; collectionCode: Insects; basisOfRecord: Pinned Specimen

#### Description

**Male** (Fig. 14). Length: 5–9 mm. **Head** (Fig. 14b): vertex 1/4 of head width; two reclinate upper orbital setae; ocellar setae arising behind anterior ocellus; ocellar triangle gold, concolorous with fronto-orbital plate; fronto-orbital plate gold over 75% of surface, densely setulose, setulae not extending beyond lowest frontal seta; parafacial shiny silver and bare; facial ridge bare; pedicel black, concolorous with postpedicel; arista black, very minutely pubescent, distinctly thickened on basal 1/3–1/4; palpus yellow and haired. **Thorax** (Fig. 14a, c): thorax gold tomentose dorsally, grey tomentose laterally; four narrow yet prominent dorsal vittae, outermost two broken across suture, innermost pair unbroken, reaching just beyond 1st postsutural dorsocentral seta; postpronotum with 3–5 setae arranged in a triangle; chaetotaxy: acrostichal setae 4:3; dorsocentral setae 3:4; intra-alar setae 3:3; supra-alar setae 2:3; three katepisternal setae; basal scutellar setae subequal in length to subapical scutellar setae; lateral scutellar setae 4/5 length of basal setae; subapical setae straight and divergent; apical scutellar short and weak, 1/3 length of subapical scutellar setae, erect and crossed midway along length; one pair of discal scutellar setae more widely set than apical scutellar setae, but only slightly narrower than subapical scutellar setae; scutellum concolorous with scutum. **Legs** (Fig. 14c): black throughout; fore femur with dense silver tomentum on posterodorsal surface; hind coxa setose. **Wing** (Fig. 14a): clear translucent, not infuscate; vein R_4+5_ with two setulae at base. **Abdomen** (Fig. 14a, c): ground colour black; middorsal depression on ST1+2 reaching hind margin; abdominal tomentum present as gold bands spanning anterior 70% of T3–T4; T5 entirely gold tomentose; median marginal setae present on ST1+2 and T3; a complete row of marginal setae present on T4; discal setae present on T3–T5; sex patch absent. **Terminalia** (Fig. 14d, e, f): anterior margin of sternite 5 (Fig. 14f) with a slight curved medial depression, posterior margin with a deeply excavated median cleft, smoothly U-shaped; posterior lobes of sternite rounded, toothed apically and completely devoid of any setae; anterior plate of sternite 5 1/5 as long as apical lobes, slightly translucent throughout, not as sclerotised as lobes. Unsclerotised "window" anterior to median cleft umbonate, central portion of "window" 2X as long as median cleft, "arms" extending 2X as wide as median cleft. Cerci (Fig. 14d) in posterior view subrectangular and slightly shorter than surstyli, blunt and rounded off towards apex, completely separate medially, diverging slightly at tips; in lateral view (Fig. 14e) strongly tapered along anterior 1/3 and weakly curved at beginning of taper; densely setulose dorsally up to taper point, apparently bare ventrally (visible in lateral view). Surstylus in lateral view slightly swollen basally, otherwise almost parallel-sided with rounded ends; when viewed dorsally, surstyli appearing weakly divergent and slightly open at tips. Pregonite broad and well-developed, slightly bent; basal 2/3 slightly cinched, giving it a very slightly clubbed appearance, apically rounded, devoid of setulae. Postgonite elongate and slender, horn-shaped, subequal in length to pregonite. Distiphallus sail-shaped, apically flared, with a slender median longitudinal sclerotised reinforcement on its posterior surface and a broad, anterolateral, sclerotised acrophallus on each side, joining the plate of opposite side on anterior surface near apex.

**Female**. Length: 6–9 mm. As male, differing only by the presence of two pairs of proclinate orbital setae.

#### Diagnosis

*Hyphantrophaga eliethcantillanoae* **sp. n.** can be distinguished from all other *Hyphantrophaga* species by the following combination of traits: pedicel and arista concolorous black, four postsutural dorsocentral setae, four thoracic vittae with inner pair solid across suture, hind coxa setose, discal setae present only on T3–T5 and T5 entirely gold tomentose.

#### Etymology

*Hyphantrophaga eliethcantillanoae* **sp. n.** is named in recognition of Elieth Cantillano Espinoza's dedication and work in finding and rearing the ACG caterpillars that contained tachinid larvae.

#### Distribution

Costa Rica, ACG, Alajuela and Guanacaste Provinces, 10–470 m elevation.

#### Ecology

*Hyphantrophaga eliethcantillanoae* **sp. n.** has been reared 386 times from two species of Lepidoptera in the family Crambidae, *Omiodes cuniculalis* Guenée, 1854 and *Omiodes fulvicauda* (Hampson, 1898); in rain forest, dry forest and dry-rain lowland intergrade.

### Hyphantrophaga gilberthampiei

Fleming & Wood sp. n.

urn:lsid:zoobank.org:act:7E9DB836-E9BD-446B-8AFA-3F0CD45325F7

#### Materials

- **Type status:** Holotype. **Occurrence:** occurrenceDetails: http://janzen.sas.upenn.edu; catalogNumber: DHJPAR0059246; recordedBy: D.H. Janzen, W. Hallwachs & Manuel Rios; individualID: DHJPAR0059246; individualCount: 1; sex: female; lifeStage: adult; preparations: pinned; otherCatalogNumbers: ACGBA5663-16, 16-SRNP-30146, BOLD:ADE0400; **Taxon:** scientificName: Hyphantrophaga gilberthampiei; phylum: Arthropoda; class: Insecta; order: Diptera; family: Tachinidae; genus: Hyphantrophaga; specificEpithet: gilberthampiei; scientificNameAuthorship: Fleming & Wood, 2017; **Location:** continent: Central America; country: Costa Rica; countryCode: CR; stateProvince: Guanacaste; county: Sector Pitilla; locality: Area de Conservacion Guanacaste; verbatimLocality: Sendero Orosilito; verbatimElevation: 900; verbatimLatitude: 10.98332; verbatimLongitude: -85.43623; verbatimCoordinateSystem: Decimal; decimalLatitude: 10.98332; decimalLongitude: -85.43623; **Identification:** identifiedBy: AJ Fleming; dateIdentified: 2017; **Event:** samplingProtocol: Reared from the larva of the Hesperiidae, Cynea megalops; verbatimEventDate: 10-Mar-2016; **Record Level:** language: en; institutionCode: CNC; collectionCode: Insects; basisOfRecord: Pinned Specimen

#### Description

**Male.** Not known at this time.

**Female** (Fig. 15). Length: 8–11 mm . **Head** (Fig. 15b): vertex 1/4 of head width; two reclinate upper orbital setae and two pairs of proclinate orbital setae; ocellar setae arising beside anterior ocellus; ocellar triangle gold; fronto-orbital plate gold throughout, sparse short setulae interspersed amongst frontal setae; parafacial shiny silver and bare; facial ridge bare; eye with short sparse ommatrichia up to 2X as long as one ommatidium; pedicel black, concolorous with postpedicel; arista brown, very minutely pubescent, distinctly thickened on basal 1/3–1/4; palpus orange, sparsely haired and oar-shaped. **Thorax** (Fig. 15a, c): brilliant gold tomentose dorsally, contrasting with silver grey laterally; with black setulae along both dorsal and lateral surfaces; thoracic vittae fused into two prominent stripes spanning entire thorax, unbroken along suture; postpronotum with five setae arranged in a triangle; chaetotaxy: acrostichal setae 3:3; dorsocentral setae 3:4; intra-alar setae 2:3; supra-alar setae 2:3; two katepisternal setae; basal scutellar setae 1/2 as long as subapical scutellar setae and slightly curving inwards medially; lateral scutellar setae subequal to basal scutellar setae; apical scutellar setae 1/3 length of subapical setae and straight; one pair of discal scutellar setae; scutellum concolorous with scutum, black over 50% of its surface with gold tomentum only along outer margin. **Legs** (Fig. 15c): black in ground colour; fore femur with dense silver tomentum on posterodorsal surface; hind coxa bare. **Wing** (Fig. 15a): pale translucent, hyaline, not distinctly infuscate; vein R_4+5_ with only 2–3 setulae at base. **Abdomen** (Fig. 15a, c): ground colour black; middorsal depression on ST1+2 reaching hind margin; median marginal setae present on T3; a complete row of marginal setae present on T4; discal setae only on T5; light silver tomentose bands along anterior edge extending over 20% of T3 and T4, broken medially by a dorsocentral stripe; T5 with silver tomentum covering anterior half. **Terminalia**: not examined.

#### Diagnosis

*Hyphantrophaga gilberthampiei* **sp. n.** can be distinguished from all other *Hyphantrophaga* species by the following combination of traits: legs entirely black; dorsum of thorax gold tomentose with four dorsal vittae fused into two prominent dark lines extending across thoracic suture; scutellum black over 50% of its surface, with gold tomentum only along margin; ground colour of abdomen black, T4 with a light dusting of silver tomentum covering approximately 20% of dorsal surface of tergite; whole abdomen with a velvet black texture and colour.

#### Etymology

*Hyphantrophaga gilberthampiei* **sp. n.** is named in recognition of Gilberth Ampie Cruz's dedication and work in finding and rearing the ACG caterpillars that contained tachinid larvae.

#### Distribution

Costa Rica, ACG, Guanacaste Province, 900 m elevation.

#### Ecology

*Hyphantrophaga gilberthampiei* **sp. n.** has been reared once from one species of Lepidoptera in the family Hesperiidae, *Cynea megalops* (Godman, 1900), in dry forest.

### Hyphantrophaga guillermopereirai

Fleming & Wood sp. n.

urn:lsid:zoobank.org:act:D6D2B422-709C-4B56-A412-6720D7F9AE6D

#### Materials

- **Type status:** Holotype. **Occurrence:** occurrenceDetails: http://janzen.sas.upenn.edu; catalogNumber: DHJPAR0007280; recordedBy: D.H. Janzen, W. Hallwachs & Manuel Pereira; individualID: DHJPAR0007280; individualCount: 1; sex: male; lifeStage: adult; preparations: pinned; otherCatalogNumbers: ASTAT052-06, 00-SRNP-16230, BOLD:AAA1953; **Taxon:** scientificName: Hyphantrophaga guillermopereirai; phylum: Arthropoda; class: Insecta; order: Diptera; family: Tachinidae; genus: Hyphantrophaga; specificEpithet: guillermopereirai; scientificNameAuthorship: Fleming & Wood, 2017; **Location:** continent: Central America; country: Costa Rica; countryCode: CR; stateProvince: Guanacaste; county: Potrerillos; locality: Area de Conservacion Guanacaste; verbatimLocality: Rio Azufrado; verbatimElevation: 95; verbatimLatitude: 10.8122; verbatimLongitude: -85.5444; verbatimCoordinateSystem: Decimal; decimalLatitude: 10.8122; decimalLongitude: -85.5444; **Identification:** identifiedBy: AJ Fleming; dateIdentified: 2017; **Event:** samplingProtocol: Reared from the larva of the Nymphalidae, Agraulis vanillae; verbatimEventDate: 22-Aug-2000; **Record Level:** language: en; institutionCode: CNC; collectionCode: Insects; basisOfRecord: Pinned Specimen
- **Type status:** Paratype. **Occurrence:** occurrenceDetails: http://janzen.sas.upenn.edu; catalogNumber: DHJPAR0007277; recordedBy: D.H. Janzen, W. Hallwachs & gusaneros; individualID: DHJPAR0007277; individualCount: 1; sex: male; lifeStage: adult; preparations: pinned; otherCatalogNumbers: ASTAT049-06, 92-SRNP-5826, BOLD:AAA1953; **Taxon:** scientificName: Hyphantrophaga guillermopereirai; phylum: Arthropoda; class: Insecta; order: Diptera; family: Tachinidae; genus: Hyphantrophaga; specificEpithet: guillermopereirai; scientificNameAuthorship: Fleming & Wood, 2017; **Location:** continent: Central America; country: Costa Rica; countryCode: CR; stateProvince: Guanacaste; county: Sector Santa Rosa; locality: Area de Conservacion Guanacaste; verbatimLocality: Bosque Humedo; verbatimElevation: 290; verbatimLatitude: 10.8514; verbatimLongitude: -85.608; verbatimCoordinateSystem: Decimal; decimalLatitude: 10.8514; decimalLongitude: -85.608; **Identification:** identifiedBy: AJ Fleming; dateIdentified: 2017; **Event:** samplingProtocol: Reared from the larva of the Hesperiidae, Polyctor cleta; verbatimEventDate: 30-Nov-1992; **Record Level:** language: en; institutionCode: CNC; collectionCode: Insects; basisOfRecord: Pinned Specimen
- **Type status:** Paratype. **Occurrence:** occurrenceDetails: http://janzen.sas.upenn.edu; catalogNumber: DHJPAR0048680; recordedBy: D.H. Janzen, W. Hallwachs & Lucia Rios; individualID: DHJPAR0048680; individualCount: 1; sex: male; lifeStage: adult; preparations: pinned; otherCatalogNumbers: ACGBA2222-12, 12-SRNP-20677, BOLD:AAA1953; **Taxon:** scientificName: Hyphantrophaga guillermopereirai; phylum: Arthropoda; class: Insecta; order: Diptera; family: Tachinidae; genus: Hyphantrophaga; specificEpithet: guillermopereirai; scientificNameAuthorship: Fleming & Wood, 2017; **Location:** continent: Central America; country: Costa Rica; countryCode: CR; stateProvince: Guanacaste; county: Sector El Hacha; locality: Area de Conservacion Guanacaste; verbatimLocality: Estacion Los Almendros; verbatimElevation: 290; verbatimLatitude: 11.0323; verbatimLongitude: -85.5278; verbatimCoordinateSystem: Decimal; decimalLatitude: 11.0323; decimalLongitude: -85.5278; **Identification:** identifiedBy: AJ Fleming; dateIdentified: 2017; **Event:** samplingProtocol: Reared from the larva of the Erebidae, Renodes curviluna; verbatimEventDate: 12-Apr-2012; **Record Level:** language: en; institutionCode: CNC; collectionCode: Insects; basisOfRecord: Pinned Specimen
- **Type status:** Paratype. **Occurrence:** occurrenceDetails: http://janzen.sas.upenn.edu; catalogNumber: DHJPAR0007303; recordedBy: D.H. Janzen, W. Hallwachs & gusaneros; individualID: DHJPAR0007303; individualCount: 1; sex: male; lifeStage: adult; preparations: pinned; otherCatalogNumbers: ASTAT075-06, 93-SRNP-7336, BOLD:AAA1953; **Taxon:** scientificName: Hyphantrophaga guillermopereirai; phylum: Arthropoda; class: Insecta; order: Diptera; family: Tachinidae; genus: Hyphantrophaga; specificEpithet: guillermopereirai; scientificNameAuthorship: Fleming & Wood, 2017; **Location:** continent: Central America; country: Costa Rica; countryCode: CR; stateProvince: Guanacaste; county: Sector Santa Rosa; locality: Area de Conservacion Guanacaste; verbatimLocality: Bosque Humedo; verbatimElevation: 290; verbatimLatitude: 10.8514; verbatimLongitude: -85.608; verbatimCoordinateSystem: Decimal; decimalLatitude: 10.8514; decimalLongitude: -85.608; **Identification:** identifiedBy: AJ Fleming; dateIdentified: 2017; **Event:** samplingProtocol: Reared from the larva of the Erebidae, Glaucostola romula; verbatimEventDate: 12-Jan-1993; **Record Level:** language: en; institutionCode: CNC; collectionCode: Insects; basisOfRecord: Pinned Specimen
- **Type status:** Paratype. **Occurrence:** occurrenceDetails: http://janzen.sas.upenn.edu; catalogNumber: DHJPAR0007029; recordedBy: D.H. Janzen, W. Hallwachs & Jose Cortez; individualID: DHJPAR0007029; individualCount: 1; sex: female; lifeStage: adult; preparations: pinned; otherCatalogNumbers: ASTAV271-06, 05-SRNP-66375, BOLD:AAA1953; **Taxon:** scientificName: Hyphantrophaga guillermopereirai; phylum: Arthropoda; class: Insecta; order: Diptera; family: Tachinidae; genus: Hyphantrophaga; specificEpithet: guillermopereirai; scientificNameAuthorship: Fleming & Wood, 2017; **Location:** continent: Central America; country: Costa Rica; countryCode: CR; stateProvince: Guanacaste; county: Sector Mundo Nuevo; locality: Area de Conservacion Guanacaste; verbatimLocality: Sendero Mora; verbatimElevation: 480; verbatimLatitude: 10.7683; verbatimLongitude: -85.4257; verbatimCoordinateSystem: Decimal; decimalLatitude: 10.7683; decimalLongitude: -85.4257; **Identification:** identifiedBy: AJ Fleming; dateIdentified: 2017; **Event:** samplingProtocol: Reared from the larva of the Hesperiidae, Chiomara georgina; verbatimEventDate: 15-Jan-2006; **Record Level:** language: en; institutionCode: CNC; collectionCode: Insects; basisOfRecord: Pinned Specimen
- **Type status:** Paratype. **Occurrence:** occurrenceDetails: http://janzen.sas.upenn.edu; catalogNumber: DHJPAR0007244; recordedBy: D.H. Janzen, W. Hallwachs & Elieth Cantillano; individualID: DHJPAR0007244; individualCount: 1; sex: female; lifeStage: adult; preparations: pinned; otherCatalogNumbers: ASTAT016-06, 04-SRNP-20122, BOLD:AAA1953; **Taxon:** scientificName: Hyphantrophaga guillermopereirai; phylum: Arthropoda; class: Insecta; order: Diptera; family: Tachinidae; genus: Hyphantrophaga; specificEpithet: guillermopereirai; scientificNameAuthorship: Fleming & Wood, 2017; **Location:** continent: Central America; country: Costa Rica; countryCode: CR; stateProvince: Guanacaste; county: Sector El Hacha; locality: Area de Conservacion Guanacaste; verbatimLocality: Sendero Bejuquilla; verbatimElevation: 280; verbatimLatitude: 11.03; verbatimLongitude: -85.527; verbatimCoordinateSystem: Decimal; decimalLatitude: 11.03; decimalLongitude: -85.527; **Identification:** identifiedBy: AJ Fleming; dateIdentified: 2017; **Event:** samplingProtocol: Reared from the larva of the Hesperiidae, Tosta gorgus; verbatimEventDate: 14-Feb-2004; **Record Level:** language: en; institutionCode: CNC; collectionCode: Insects; basisOfRecord: Pinned Specimen

#### Description

**Male** (Fig. 16). Length: 5–8 mm. **Head** (Fig. 16b): vertex 1/5 of head width; two reclinate upper orbital setae; ocellar setae arising beside anterior ocellus; ocellar triangle gold, concolorous with fronto-orbital plate; fronto-orbital plate gold over 75% of surface, sparsely setulose, setulae not extending beyond lowest frontal seta; parafacial shiny silver and bare; facial ridge bare; pedicel dark brown, slightly lighter than postpedicel; arista brown, very minutely pubescent, distinctly thickened on basal 1/3–1/4; palpus yellow and haired. **Thorax** (Fig. 16a, c): pale brassy-gold tomentose dorsally, gray tomentose laterally; four prominent dorsal vittae, outermost two broken across suture, innermost pair slightly broken across suture, not reaching beyond 2nd postsutural dorsocentral seta; postpronotum with three setae arranged in a triangle; chaetotaxy: acrostichal setae 3:3; dorsocentral setae 3–4:4; intra-alar setae 2–3:3; supra-alar setae 2:3; three katepisternal setae, basal seta extremely weak and anterior to suture; basal scutellar setae 3/4 length of subapical scutellar setae; lateral scutellar setae 2/3 as long as basal scutellar setae, lateral scutellar setae 1/2 as thick as both basal and subapical scutellar setae; subapical scutellar setae straight and divergent, 2X as thick as lateral scutellar setae; apical scutellar setae 1/3 basal scutellar setae crossed apically; one pair of discal scutellar setae more widely set than subapical scutellar setae; scutellum concolorous with scutum. **Legs** (Fig. 16c): yellow in ground colour with dense covering of black hairs, making them appear darker; fore femur with dense silver tomentum on posterodorsal surface; hind coxa setose. **Wing** (Fig. 16a): pale translucent; vein R_4+5_ with 2–3 setulae at base. **Abdomen** (Fig. 16a, c): ground colour black; mid-dorsal depression on ST1+2 not reaching hind margin; marginal setae ST1+2–T5: median marginal setae present on ST1+2 and T3; a complete row of marginal setae present on T4 and T5; discal setae on T3–T5; sex patch absent; distinct brassy-silver tomentose bands along anterior 2/3 of T3 and T4; T5 with brassy-silver tomentum covering entire tergite. **Terminalia** (Fig. 16d, e, f): anterior margin of sternite 5 (Fig. 16f) with a shallow curved medial depression, posterior margin with a deeply excavated median cleft, rectangular-shaped; posterior lobes of sternite rounded, toothed apically and completely devoid of setae, inner margins of lobes sinusoidal undulated; unsclerotised "window" anterior to median cleft convex, arced, "arms" extending 3X as wide as median cleft. Cerci (Fig. 16d) in posterior view subrectangular and slightly shorter than surstyli, blunt and rounded off towards apices, completely separate medially, straight, not obviously divergent from each other; in lateral view (Fig. 16e) strongly tapered along apical 1/3 and weakly curved at beginning of taper; densely setulose dorsally up to taper point, apparently bare ventrally (visible in lateral view). Surstyli in lateral view slightly swollen basally, otherwise almost parallel sided, with rounded apices; when viewed dorsally, surstyli appearing not divergent. Pregonite broad and well-developed, slightly bent; basal 2/3 slightly cinched, giving it a very slightly clubbed appearance, apically rounded, few fine setulae along its edge. Postgonite elongate, parallel-sided along its length, with a slight curve at tip, subequal in length to pregonite. Distiphallus rectangular with a very slight apical flare, with a slender median longitudinal sclerotised reinforcement on its posterior surface and a broad, anterolateral, sclerotised acrophallus, joined as a plate on anterior surface near apex.

**Female**. Length 6–8 mm. As male, differing only by the presence of two pairs of proclinate orbital setae.

#### Diagnosis

*Hyphantrophaga guillermopereirai* **sp. n.** can be distinguished from all other *Hyphantrophaga* species by the following combination of traits: fronto-orbital plate gold over most of its surface and bare, except at level of vertex, pedicel and arista brown (slightly lighter than postpedicel), thorax with both silver and gold tomentum, four postsutural dorsocentral setae, hind coxa setose, marginal setae present on ST1+2–T5 and discal setae present on T3–T5.

#### Etymology

*Hyphantrophaga guillermopereirai* **sp. n.** is named in recognition of Guillermo Pereira Espinoza's dedication and work in finding and rearing the ACG caterpillars that contained tachinid larvae.

#### Distribution

Costa Rica, ACG, Alajuela and Guanacaste Provinces, 10–752 m elevation.

#### Ecology

*Hyphantrophaga guillermopereirai* **sp. n.** has been reared 93 times from 56 species of Lepidoptera spanning across 10 families (Lepidoptera: Crambidae; Erebidae; Geometridae; Hesperiidae; Noctuidae; Notodontidae; Nolidae; Nymphalidae; Riodinidae; Saturniidae), in rain forest, dry forest and dry-rain lowland intergrade.

### Hyphantrophaga hazelcambroneroae

Fleming & Wood sp. n.

urn:lsid:zoobank.org:act:3C8894B8-AAB7-46E2-BF26-AEAC3C3A0169

#### Materials

- **Type status:** Holotype. **Occurrence:** occurrenceDetails: http://janzen.sas.upenn.edu; catalogNumber: DHJPAR0007278; recordedBy: D.H. Janzen, W. Hallwachs and gusaneros; individualID: DHJPAR0007278; individualCount: 1; sex: male; lifeStage: adult; preparations: pinned; otherCatalogNumbers: ASTAT050-06, 93-SRNP-6894,; **Taxon:** scientificName: Hyphantrophaga hazelcambroneroae; phylum: Arthropoda; class: Insecta; order: Diptera; family: Tachinidae; genus: Hyphantrophaga; specificEpithet: hazelcambroneroae; scientificNameAuthorship: Fleming & Wood, 2017; **Location:** continent: Central America; country: Costa Rica; countryCode: CR; stateProvince: Guanacaste; county: Sector Santa Rosa; locality: Area de Conservacion Guanacaste; verbatimLocality: Bosque San Emilio; verbatimElevation: 300; verbatimLatitude: 10.8439; verbatimLongitude: -85.6138; verbatimCoordinateSystem: Decimal; decimalLatitude: 10.8439; decimalLongitude: -85.6138; **Identification:** identifiedBy: AJ Fleming; dateIdentified: 2017; **Event:** samplingProtocol: Reared from the larva of the Crambidae, Hoterodes ausonia; verbatimEventDate: 11-Jul-1993; **Record Level:** language: en; institutionCode: CNC; collectionCode: Insects; basisOfRecord: Pinned Specimen
- **Type status:** Paratype. **Occurrence:** occurrenceDetails: http://janzen.sas.upenn.edu; catalogNumber: DHJPAR0052525; recordedBy: D.H. Janzen, W. Hallwachs and Guillermo Pereira; individualID: DHJPAR0052525; individualCount: 1; sex: male; lifeStage: adult; preparations: pinned; otherCatalogNumbers: ASHYM1879-13, 13-SRNP-19665, BOLD:ACJ4813; **Taxon:** scientificName: Hyphantrophaga hazelcambroneroae; phylum: Arthropoda; class: Insecta; order: Diptera; family: Tachinidae; genus: Hyphantrophaga; specificEpithet: hazelcambroneroae; scientificNameAuthorship: Fleming & Wood, 2017; **Location:** continent: Central America; country: Costa Rica; countryCode: CR; stateProvince: Guanacaste; county: Sector Santa Rosa; locality: Area de Conservacion Guanacaste; verbatimLocality: Area Administrativa; verbatimElevation: 295; verbatimLatitude: 10.8376; verbatimLongitude: -85.6187; verbatimCoordinateSystem: Decimal; decimalLatitude: 10.8376; decimalLongitude: -85.6187; **Identification:** identifiedBy: AJ Fleming; dateIdentified: 2017; **Event:** samplingProtocol: Reared from the larva of the Crambidae, Portentomorpha xanthialis; verbatimEventDate: 20-Aug-2013; **Record Level:** language: en; institutionCode: CNC; collectionCode: Insects; basisOfRecord: Pinned Specimen
- **Type status:** Paratype. **Occurrence:** occurrenceDetails: http://janzen.sas.upenn.edu; catalogNumber: DHJPAR0052653; recordedBy: D.H. Janzen, W. Hallwachs and Johan Vargas; individualID: DHJPAR0052653; individualCount: 1; sex: male; lifeStage: adult; preparations: pinned; otherCatalogNumbers: ASHYM2007-13, 13-SRNP-19649, BOLD:ACJ4813; **Taxon:** scientificName: Hyphantrophaga hazelcambroneroae; phylum: Arthropoda; class: Insecta; order: Diptera; family: Tachinidae; genus: Hyphantrophaga; specificEpithet: hazelcambroneroae; scientificNameAuthorship: Fleming & Wood, 2017; **Location:** continent: Central America; country: Costa Rica; countryCode: CR; stateProvince: Guanacaste; county: Sector Santa Rosa; locality: Area de Conservacion Guanacaste; verbatimLocality: Area Administrativa; verbatimElevation: 295; verbatimLatitude: 10.8376; verbatimLongitude: -85.6187; verbatimCoordinateSystem: Decimal; decimalLatitude: 10.8376; decimalLongitude: -85.6187; **Identification:** identifiedBy: AJ Fleming; dateIdentified: 2017; **Event:** samplingProtocol: Reared from the larva of the Crambidae, Portentomorpha xanthialis; verbatimEventDate: 14-Aug-2013; **Record Level:** language: en; institutionCode: CNC; collectionCode: Insects; basisOfRecord: Pinned Specimen
- **Type status:** Paratype. **Occurrence:** occurrenceDetails: http://janzen.sas.upenn.edu; catalogNumber: DHJPAR0052531; recordedBy: D.H. Janzen, W. Hallwachs and Guillermo Pereira; individualID: DHJPAR0052531; individualCount: 1; sex: male; lifeStage: adult; preparations: pinned; otherCatalogNumbers: ASHYM1885-13, 13-SRNP-19667, BOLD:ACJ4813; **Taxon:** scientificName: Hyphantrophaga hazelcambroneroae; phylum: Arthropoda; class: Insecta; order: Diptera; family: Tachinidae; genus: Hyphantrophaga; specificEpithet: hazelcambroneroae; scientificNameAuthorship: Fleming & Wood, 2017; **Location:** continent: Central America; country: Costa Rica; countryCode: CR; stateProvince: Guanacaste; county: Sector Santa Rosa; locality: Area de Conservacion Guanacaste; verbatimLocality: Area Administrativa; verbatimElevation: 295; verbatimLatitude: 10.8376; verbatimLongitude: -85.6187; verbatimCoordinateSystem: Decimal; decimalLatitude: 10.8376; decimalLongitude: -85.6187; **Identification:** identifiedBy: AJ Fleming; dateIdentified: 2017; **Event:** samplingProtocol: Reared from the larva of the Crambidae, Portentomorpha xanthialis; verbatimEventDate: 20-Aug-2013; **Record Level:** language: en; institutionCode: CNC; collectionCode: Insects; basisOfRecord: Pinned Specimen
- **Type status:** Paratype. **Occurrence:** occurrenceDetails: http://janzen.sas.upenn.edu; catalogNumber: DHJPAR0052540; recordedBy: D.H. Janzen, W. Hallwachs and Guillermo Pereira; individualID: DHJPAR0052540; individualCount: 1; sex: male; lifeStage: adult; preparations: pinned; otherCatalogNumbers: ASHYM1894-13, 13-SRNP-19685, BOLD:ACJ4813; **Taxon:** scientificName: Hyphantrophaga hazelcambroneroae; phylum: Arthropoda; class: Insecta; order: Diptera; family: Tachinidae; genus: Hyphantrophaga; specificEpithet: hazelcambroneroae; scientificNameAuthorship: Fleming & Wood, 2017; **Location:** continent: Central America; country: Costa Rica; countryCode: CR; stateProvince: Guanacaste; county: Sector Santa Rosa; locality: Area de Conservacion Guanacaste; verbatimLocality: Area Administrativa; verbatimElevation: 295; verbatimLatitude: 10.8376; verbatimLongitude: -85.6187; verbatimCoordinateSystem: Decimal; decimalLatitude: 10.8376; decimalLongitude: -85.6187; **Identification:** identifiedBy: AJ Fleming; dateIdentified: 2017; **Event:** samplingProtocol: Reared from the larva of the Crambidae, Portentomorpha xanthialis; verbatimEventDate: 20-Aug-2013; **Record Level:** language: en; institutionCode: CNC; collectionCode: Insects; basisOfRecord: Pinned Specimen

#### Description

**Male** (Fig. 17). Length: 5–8 mm. **Head** (Fig. 17b): vertex 1/4 of head width; two strong reclinate upper orbital setae, these not in line with frontal row (one aberrant male displaying one proclinate orbital seta in addition to the two reclinate pairs, was initially confused as female); ocellar setae arising behind anterior ocellus; ocellar triangle gold, concolorous with upper half of fronto-orbital plate; fronto-orbital plate gold over 50% of surface, sparsely setulose, setulae not extending beyond lowest frontal seta; parafacial shiny silver and bare; facial ridge haired up to 1/2 its surface, these being strong and widely spaced; pedicel dark brown, slightly lighter than postpedicel; arista brown, very minutely pubescent, distinctly thickened on basal 1/3–1/4; palpus yellow and haired, not clubbed. **Thorax** (Fig. 17a, c): brassy-gold tomentose dorsally, grey tomentose laterally; four prominent dorsal vittae, outermost two broken across suture, innermost pair slightly broken across suture, not reaching beyond 2nd postsutural dorsocentral seta; postpronotum with three setae arranged in a triangle; chaetotaxy: acrostichal setae 3:3; dorsocentral setae 3:4 (second postsutural seta often reduced and weaker than the other three); intra-alar setae 2:3; supra-alar setae 2:3; two katepisternal setae; lateral scutellar setae 2/3 as long as basal scutellar setae, lateral scutellar setae 1/2 as thick as both basal and subapical scutellar setae; subapical scutellar setae strongest and longest of marginal scutellar setae, strongly divergent; apical scutellar setae shorter than basal scutellar setae crossed apically; one pair of discal scutellar setae; scutellum concolorous with scutum, very slightly darker than thorax along basal 50%. **Legs** (Fig. 17c): reddish-black in ground colour with dense covering of black hairs, making them appear darker; fore femur with dense silver tomentum on posterodorsal surface; hind coxa bare. **Wing** (Fig. 17a): pale translucent; vein R_4+5_ with 2–3 setulae at base. **Abdomen** (Fig. 17a, c): ground colour black; middorsal depression on ST1+2 reaching hind margin; median marginal setae present on ST1+2 and T3; a complete row of marginal setae present on T4; discal setae on T3–T5; sex patch absent; distinct brassy-gold tomentose bands dorsally along anterior 2/3 of T3 and T4; T5 with brassy-gold tomentum covering entire tergite; T3–T5 silver tomentose ventrally. **Terminalia**: anterior margin of sternite 5 (Fig. 17f) with a deep curved medial depression, posterior margin with a deeply excavated median cleft, two pyramid-shaped lobes; posterior lobes of sternite slightly pointed apically, 1–2 longer setae surrounded by shorter setulae, inner margins of cleft straight with a slight undulation, round cylindrical basally; unsclerotised "window" anterior to median cleft convex, with slender "arms" extending 3X as wide as median cleft with a short rectangular stalk. Cerci (Fig. 17d) in posterior view almost rectangular and subequal in length to surstyli, blunt and rounded off towards apices, separate medially on anterior 1/3, straight and slightly divergent when viewed dorsally, remaining 2/3 fused medially; in lateral view (Fig. 17e), almost parallel-sided along entire length, anterior 1/3 with a strong downward curve; densely setulose dorsally up to curve point, apparently bare ventrally (visible in lateral view). Surstyli in lateral view parallel-sided along their entire length, with rounded apices, strongly curved along anterior 1/3; when viewed dorsally, surstyli appearing not divergent. Pregonite broad and well-developed, slightly bent; basal 2/3 slightly cinched, giving it a very slightly clubbed appearance, apically rounded, few fine setulae along its edge. Postgonite elongate, parallel-sided along its length, with a slight curve at tip, subequal in length to pregonite. Distiphallus sail-shaped, with a very strong apical flare, a slender median longitudinal sclerotised reinforcement on its posterior surface and a broad, anterolateral, sclerotised acrophallus, joined as a plate on anterior surface near apex.

**Female**. Unknown at this time.

#### Diagnosis

*Hyphantrophaga hazelcambroneroae* **sp. n.** can be distinguished from all other *Hyphantrophaga* species by the following combination of traits: facial ridge with setae along half its length, four dorsocentral setae, two katepisternal setae, hind coxa bare, both abdomen and thorax brassy tomentose throughout, abdominal ground color black and median marginal setae present on ST1+2.

#### Etymology

*Hyphantrophaga hazelcambroneroae* **sp. n.** is named in recognition of Hazel Cambronero Romero's dedication and work in finding and rearing the ACG caterpillars that contained tachinid larvae.

#### Distribution

Costa Rica, ACG, Guanacaste Province, 295–300 m elevation.

#### Ecology

*Hyphantrophaga hazelcambroneroae* **sp. n.** has been reared five times from two species of Lepidoptera in the family Crambidae, *Hoterodes ausonia* Cramer, 1777 and *Portentomorpha xanthialis* (Guenée, 1854), in dry forest.

### Hyphantrophaga luciariosae

Fleming & Wood sp. n.

urn:lsid:zoobank.org:act:1DBE5C00-14E2-41B9-B9CF-7E7ED59CD497

#### Materials

- **Type status:** Holotype. **Occurrence:** occurrenceDetails: http://janzen.sas.upenn.edu; catalogNumber: DHJPAR0008095; recordedBy: D.H. Janzen, W. Hallwachs & Roster Moraga; individualID: DHJPAR0008095; individualCount: 1; sex: male; lifeStage: adult; preparations: pinned; otherCatalogNumbers: ASTAT867-06, 01-SRNP-9682, BOLD:AAF3892; **Taxon:** scientificName: Hyphantrophaga luciariosae; phylum: Arthropoda; class: Insecta; order: Diptera; family: Tachinidae; genus: Hyphantrophaga; specificEpithet: luciariosae; scientificNameAuthorship: Fleming & Wood, 2018; **Location:** continent: Central America; country: Costa Rica; countryCode: CR; stateProvince: Guanacaste; county: Sector Orosi; locality: Area de Conservacion Guanacaste; verbatimLocality: Sendero Orosi; verbatimElevation: 620; verbatimLatitude: 10.9937; verbatimLongitude: -85.4639; verbatimCoordinateSystem: Decimal; decimalLatitude: 10.9937; decimalLongitude: -85.4639; **Identification:** identifiedBy: AJ Fleming; dateIdentified: 2017; **Event:** samplingProtocol: Reared from the larva of the Sphingidae, Xylophanes juanita; verbatimEventDate: 26-Jul-2001; **Record Level:** language: en; institutionCode: CNC; collectionCode: Insects; basisOfRecord: Pinned Specimen
- **Type status:** Paratype. **Occurrence:** occurrenceDetails: http://janzen.sas.upenn.edu; catalogNumber: DHJPAR0008061; recordedBy: D.H. Janzen, W. Hallwachs & Roster Moraga; individualID: DHJPAR0008061; individualCount: 1; sex: female; lifeStage: adult; preparations: pinned; otherCatalogNumbers: ASTAT833-06, 01-SRNP-9654, BOLD:AAF3892; **Taxon:** scientificName: Hyphantrophaga luciariosae; phylum: Arthropoda; class: Insecta; order: Diptera; family: Tachinidae; genus: Hyphantrophaga; specificEpithet: luciariosae; scientificNameAuthorship: Fleming & Wood, 2018; **Location:** continent: Central America; country: Costa Rica; countryCode: CR; stateProvince: Guanacaste; county: Sector Orosi; locality: Area de Conservacion Guanacaste; verbatimLocality: Sendero Orosi; verbatimElevation: 620; verbatimLatitude: 10.9937; verbatimLongitude: -85.4639; verbatimCoordinateSystem: Decimal; decimalLatitude: 10.9937; decimalLongitude: -85.4639; **Identification:** identifiedBy: AJ Fleming; dateIdentified: 2017; **Event:** samplingProtocol: Reared from the larva of the Sphingidae, Xylophanes juanita; verbatimEventDate: 29-Jul-2001; **Record Level:** language: en; institutionCode: CNC; collectionCode: Insects; basisOfRecord: Pinned Specimen
- **Type status:** Paratype. **Occurrence:** occurrenceDetails: http://janzen.sas.upenn.edu; catalogNumber: DHJPAR0008097; recordedBy: D.H. Janzen, W. Hallwachs & Roster Moraga; individualID: DHJPAR0008097; individualCount: 1; sex: male; lifeStage: adult; preparations: pinned; otherCatalogNumbers: ASTAT869-06, 01-SRNP-9680, BOLD:AAF3892; **Taxon:** scientificName: Hyphantrophaga luciariosae; phylum: Arthropoda; class: Insecta; order: Diptera; family: Tachinidae; genus: Hyphantrophaga; specificEpithet: luciariosae; scientificNameAuthorship: Fleming & Wood, 2018; **Location:** continent: Central America; country: Costa Rica; countryCode: CR; stateProvince: Guanacaste; county: Sector Orosi; locality: Area de Conservacion Guanacaste; verbatimLocality: Sendero Orosi; verbatimElevation: 620; verbatimLatitude: 10.9937; verbatimLongitude: -85.4639; verbatimCoordinateSystem: Decimal; decimalLatitude: 10.9937; decimalLongitude: -85.4639; **Identification:** identifiedBy: AJ Fleming; dateIdentified: 2017; **Event:** samplingProtocol: Reared from the larva of the Sphingidae, Xylophanes juanita; verbatimEventDate: 16-Jul-2001; **Record Level:** language: en; institutionCode: CNC; collectionCode: Insects; basisOfRecord: Pinned Specimen
- **Type status:** Paratype. **Occurrence:** occurrenceDetails: http://janzen.sas.upenn.edu; catalogNumber: DHJPAR0058396; recordedBy: D.H. Janzen, W. Hallwachs & Kemberly Villalobos; individualID: DHJPAR0058396; individualCount: 1; sex: male; lifeStage: adult; preparations: pinned; otherCatalogNumbers: MHMYN7996-16, 16-SRNP-45023, BOLD:AAF3892; **Taxon:** scientificName: Hyphantrophaga luciariosae; phylum: Arthropoda; class: Insecta; order: Diptera; family: Tachinidae; genus: Hyphantrophaga; specificEpithet: luciariosae; scientificNameAuthorship: Fleming & Wood, 2018; **Location:** continent: Central America; country: Costa Rica; countryCode: CR; stateProvince: Guanacaste; county: Sector Rincon Rain Forest; locality: Area de Conservacion Guanacaste; verbatimLocality: Malaguenya; verbatimElevation: 221; verbatimLatitude: 10.9555; verbatimLongitude: -85.2838; verbatimCoordinateSystem: Decimal; decimalLatitude: 10.9555; decimalLongitude: -85.2838; **Identification:** identifiedBy: AJ Fleming; dateIdentified: 2017; **Event:** samplingProtocol: Reared from the larva of the Sphingidae, Xylophanes adalia; verbatimEventDate: 04-Feb-2016; **Record Level:** language: en; institutionCode: CNC; collectionCode: Insects; basisOfRecord: Pinned Specimen
- **Type status:** Paratype. **Occurrence:** occurrenceDetails: http://janzen.sas.upenn.edu; catalogNumber: DHJPAR0054189; recordedBy: D.H. Janzen, W. Hallwachs & Freddy Quesada; individualID: DHJPAR0054189; individualCount: 1; sex: male; lifeStage: adult; preparations: pinned; **Taxon:** scientificName: Hyphantrophaga luciariosae; phylum: Arthropoda; class: Insecta; order: Diptera; family: Tachinidae; genus: Hyphantrophaga; specificEpithet: luciariosae; scientificNameAuthorship: Fleming & Wood, 2018; **Location:** continent: Central America; country: Costa Rica; countryCode: CR; stateProvince: Guanacaste; county: Sector Pitilla; locality: Area de Conservacion Guanacaste; verbatimLocality: Sendero Naciente; verbatimElevation: 700; verbatimLatitude: 10.9871; verbatimLongitude: -85.4282; verbatimCoordinateSystem: Decimal; decimalLatitude: 10.9871; decimalLongitude: -85.4282; **Identification:** identifiedBy: AJ Fleming; dateIdentified: 2017; **Event:** samplingProtocol: Reared from the larva of the Sphingidae, Xylophanes adalia; verbatimEventDate: Jan-19-2014; **Record Level:** language: en; institutionCode: CNC; collectionCode: Insects; basisOfRecord: Pinned Specimen

#### Description

**Male** (Fig. 18). Length: 7–10 mm. **Head** (Fig. 18b): vertex 1/4 of head width; two reclinate upper orbital setae; ocellar setae arising beside anterior ocellus; ocellar triangle slightly gold, changing to concolorous with fronto-orbital plate; fronto-orbital plate dark brassy-silver over 90% of surface, densely setulose, setulae not extending beyond lowest frontal seta; parafacial dull silver and bare; facial ridge bare; pedicel dark brown, slightly lighter than postpedicel; arista brown, very minutely pubescent, distinctly thickened on basal 1/3–1/4; palpus yellow, haired and oar-shaped. **Thorax** (Fig. 18a, c): pale brassy-gold tomentose dorsally, grey tomentose laterally; four prominent dorsal vittae, outermost two broken across suture, innermost pair unbroken across suture, reaching 3rd postsutural dorsocentral seta; postpronotum with five setae arranged in a triangle; chaetotaxy: acrostichal setae 3:3; dorsocentral setae 3:4; intra-alar setae 3:3; supra-alar setae 2:3; two katepisternal setae; basal scutellar setae as long as scutellar setae; two pairs of lateral scutellar setae, 1/2 as long as basal scutellar setae; subapical scutellar setae straight and divergent, 2X as thick as lateral scutellar setae; apical scutellar setae 1/3 basal scutellar setae, slightly shorter than lateral scutellar setae, crossed apically; one pair of discal scutellar setae more widely set than subapical scutellar setae; scutellum darkened over basal 1/3, remainder concolorous with scutum. **Legs** (Fig. 18c): reddish-brown ground colour; fore femur with dense silver tomentum on posterodorsal surface; hind coxa setose. **Wing** (Fig. 18a): pale translucent, hyaline, not distinctly infuscate; veins reddish; vein R_4+5_ with only 2–3 setulae at base. **Abdomen** (Fig. 18a, c): ground colour reddish-brown; mid-dorsal depression on ST1+2 almost reaching hind margin; median marginal setae present on ST1+2–T3; a complete row of marginal setae present on T4; discal setae only on T5; sex patch covering ventrolateral surfaces of T4–T5; distinct brassy tomentose bands along anterior edge of T3 and T4, broken medially by a dorsocentral stripe and covering almost 80% of tergites; T5 with brassy tomentum throughout. **Terminalia**: sternite 5 (Fig. 18f) with a deeply excavated median cleft, slightly rounded V-shaped, margins covered in dense tomentum. Lateral lobes of sternite rounded apically, with 2–3 strong setae surrounded by many shorter, weaker setulae. Anterior plate of sternite 5 from subequal to slightly shorter than apical lobes, unsclerotised "window" weak, only very slightly unsclerotised, as wide as median cleft and flat. Cerci in posterior view (Fig. 18d) subrectangular, duck-billed and slightly longer than surstyli, blunt and rounded at apex, completely separate medially, appearing slightly divergent; in lateral view with a strong downward curve in apical 1/3; densely setulose along basal 2/3, setulose ventrally along entire length. Surstylus in lateral view (Fig. 18e) almost parallel-sided along its entire length, ending in a slightly downcurved apex, making the structure appear slightly bladelike; surstylus appearing to be fused with epandrium; when viewed dorsally, surstyli appearing to point inward and strongly convergent, laterally covered in short stout setulae. Pregonite short and stout, well-developed, 1/3 times as long as distiphallus, squared-off apically, with a few short marginal setulae. Postgonite slightly narrow, 1/3 as wide as pregonite, sharply pointed and curved at apex. Distiphallus rectangular, with a very slight apical flare, a slender median longitudinal sclerotised reinforcement on its posterior surface and a broad, anterolateral, sclerotised acrophallus, joined as a plate on anterior surface near apex.

**Female**. Length: 8–11 mm. As male, differing only by the presence of two pairs of proclinate orbital setae.

#### Diagnosis

*Hyphantrophaga luciariosae* **sp. n.** can be distinguished from all other *Hyphantrophaga* species by the following combination of traits: thorax with four postsutural dorsocentral setae and two katepisternal setae, hind coxa setose, median marginal setae present on ST1+2, discal setae absent from T3 and T4.

#### Etymology

*Hyphantrophaga luciariosae* **sp. n.** is named in recognition of Lucia Rios Castro's dedication and work in finding and rearing the ACG caterpillars that contained tachinid larvae.

#### Distribution

Costa Rica, ACG, Guanacaste Province, 10–480 m elevation.

#### Ecology

*Hyphantrophaga luciariosae* **sp. n.** has been reared four times from two species of Lepidoptera in the family Sphingidae, *Xylophanes juanita* Rothschild & Jordan, 1903 and *Xylophanes adalia* (Druce, 1881), in dry forest and dry-rain lowland intergrades.

### Hyphantrophaga manuelriosi

Fleming & Wood sp. n.

urn:lsid:zoobank.org:act:5ED9EBB0-36C2-4227-81CD-3712EA8697BB

#### Materials

- **Type status:** Holotype. **Occurrence:** occurrenceDetails: http://janzen.sas.upenn.edu; catalogNumber: DHJPAR0007340; recordedBy: D.H. Janzen, W. Hallwachs & gusaneros; individualID: DHJPAR0007340; individualCount: 1; sex: male; lifeStage: adult; preparations: pinned; otherCatalogNumbers: ASTAT112-06, 94-SRNP-2057, BOLD:AAA8930; **Taxon:** scientificName: Hyphantrophaga manuelriosi; phylum: Arthropoda; class: Insecta; order: Diptera; family: Tachinidae; genus: Hyphantrophaga; specificEpithet: manuelriosi; scientificNameAuthorship: Fleming & Wood, 2018; **Location:** continent: Central America; country: Costa Rica; countryCode: CR; stateProvince: Guanacaste; county: Sector Santa Rosa; locality: Area de Conservacion Guanacaste; verbatimLocality: Cafetal; verbatimElevation: 280; verbatimLatitude: 10.8583; verbatimLongitude: -85.6109; verbatimCoordinateSystem: Decimal; decimalLatitude: 10.8583; decimalLongitude: -85.6109; **Identification:** identifiedBy: AJ Fleming; dateIdentified: 2017; **Event:** samplingProtocol: Reared from the larva of the Crambidae, Syllepte belialis; verbatimEventDate: 06-Jul-1994; **Record Level:** language: en; institutionCode: CNC; collectionCode: Insects; basisOfRecord: Pinned Specimen
- **Type status:** Paratype. **Occurrence:** occurrenceDetails: http://janzen.sas.upenn.edu; catalogNumber: DHJPAR0007331; recordedBy: D.H. Janzen, W. Hallwachs & gusaneros; individualID: DHJPAR0007331; individualCount: 1; sex: male; lifeStage: adult; preparations: pinned; otherCatalogNumbers: ASTAT103-06, 01-SRNP-13266, BOLD:AAA8930; **Taxon:** scientificName: Hyphantrophaga manuelriosi; phylum: Arthropoda; class: Insecta; order: Diptera; family: Tachinidae; genus: Hyphantrophaga; specificEpithet: manuelriosi; scientificNameAuthorship: Fleming & Wood, 2018; **Location:** continent: Central America; country: Costa Rica; countryCode: CR; stateProvince: Guanacaste; county: Sector Santa Rosa; locality: Area de Conservacion Guanacaste; verbatimLocality: Sendero Natural; verbatimElevation: 290; verbatimLatitude: 10.8357; verbatimLongitude: -85.6125; verbatimCoordinateSystem: Decimal; decimalLatitude: 10.8357; decimalLongitude: -85.6125; **Identification:** identifiedBy: AJ Fleming; dateIdentified: 2017; **Event:** samplingProtocol: Reared from the larva of the Crambidae, Syllepte belialis; verbatimEventDate: 10-May-2002; **Record Level:** language: en; institutionCode: CNC; collectionCode: Insects; basisOfRecord: Pinned Specimen
- **Type status:** Paratype. **Occurrence:** occurrenceDetails: http://janzen.sas.upenn.edu; catalogNumber: DHJPAR0007332; recordedBy: D.H. Janzen, W. Hallwachs & gusaneros; individualID: DHJPAR0007332; individualCount: 1; sex: male; lifeStage: adult; preparations: pinned; otherCatalogNumbers: ASTAT104-06, 01-SRNP-13278, BOLD:AAA8930; **Taxon:** scientificName: Hyphantrophaga manuelriosi; phylum: Arthropoda; class: Insecta; order: Diptera; family: Tachinidae; genus: Hyphantrophaga; specificEpithet: manuelriosi; scientificNameAuthorship: Fleming & Wood, 2018; **Location:** continent: Central America; country: Costa Rica; countryCode: CR; stateProvince: Guanacaste; county: Sector Santa Rosa; locality: Area de Conservacion Guanacaste; verbatimLocality: Sendero Natural; verbatimElevation: 290; verbatimLatitude: 10.8357; verbatimLongitude: -85.6125; verbatimCoordinateSystem: Decimal; decimalLatitude: 10.8357; decimalLongitude: -85.6125; **Identification:** identifiedBy: AJ Fleming; dateIdentified: 2017; **Event:** samplingProtocol: Reared from the larva of the Crambidae, Syllepte belialis; verbatimEventDate: 01-May-2002; **Record Level:** language: en; institutionCode: CNC; collectionCode: Insects; basisOfRecord: Pinned Specimen
- **Type status:** Paratype. **Occurrence:** occurrenceDetails: http://janzen.sas.upenn.edu; catalogNumber: DHJPAR0007333; recordedBy: D.H. Janzen, W. Hallwachs & gusaneros; individualID: DHJPAR0007333; individualCount: 1; sex: male; lifeStage: adult; preparations: pinned; otherCatalogNumbers: ASTAT105-06, 01-SRNP-13253, BOLD:AAA8930; **Taxon:** scientificName: Hyphantrophaga manuelriosi; phylum: Arthropoda; class: Insecta; order: Diptera; family: Tachinidae; genus: Hyphantrophaga; specificEpithet: manuelriosi; scientificNameAuthorship: Fleming & Wood, 2018; **Location:** continent: Central America; country: Costa Rica; countryCode: CR; stateProvince: Guanacaste; county: Sector Santa Rosa; locality: Area de Conservacion Guanacaste; verbatimLocality: Sendero Natural; verbatimElevation: 290; verbatimLatitude: 10.8357; verbatimLongitude: -85.6125; verbatimCoordinateSystem: Decimal; decimalLatitude: 10.8357; decimalLongitude: -85.6125; **Identification:** identifiedBy: AJ Fleming; dateIdentified: 2017; **Event:** samplingProtocol: Reared from the larva of the Crambidae, Syllepte belialis; verbatimEventDate: 01-May-2002; **Record Level:** language: en; institutionCode: CNC; collectionCode: Insects; basisOfRecord: Pinned Specimen
- **Type status:** Paratype. **Occurrence:** occurrenceDetails: http://janzen.sas.upenn.edu; catalogNumber: DHJPAR0007334; recordedBy: D.H. Janzen, W. Hallwachs & gusaneros; individualID: DHJPAR0007334; individualCount: 1; sex: male; lifeStage: adult; preparations: pinned; otherCatalogNumbers: ASTAT106-06, 93-SRNP-69, BOLD:AAA8930; **Taxon:** scientificName: Hyphantrophaga manuelriosi; phylum: Arthropoda; class: Insecta; order: Diptera; family: Tachinidae; genus: Hyphantrophaga; specificEpithet: manuelriosi; scientificNameAuthorship: Fleming & Wood, 2018; **Location:** continent: Central America; country: Costa Rica; countryCode: CR; stateProvince: Guanacaste; county: Sector Santa Rosa; locality: Area de Conservacion Guanacaste; verbatimLocality: Bosque Humedo; verbatimElevation: 290; verbatimLatitude: 10.8514; verbatimLongitude: -85.608; verbatimCoordinateSystem: Decimal; decimalLatitude: 10.8514; decimalLongitude: -85.608; **Identification:** identifiedBy: AJ Fleming; dateIdentified: 2017; **Event:** samplingProtocol: Reared from the larva of the Crambidae, Syllepte belialis; verbatimEventDate: 28-May-1993; **Record Level:** language: en; institutionCode: CNC; collectionCode: Insects; basisOfRecord: Pinned Specimen
- **Type status:** Paratype. **Occurrence:** occurrenceDetails: http://janzen.sas.upenn.edu; catalogNumber: DHJPAR0007336; recordedBy: D.H. Janzen, W. Hallwachs & gusaneros; individualID: DHJPAR0007336; individualCount: 1; sex: male; lifeStage: adult; preparations: pinned; otherCatalogNumbers: ASTAT108-06, 94-SRNP-2061, BOLD:AAA8930; **Taxon:** scientificName: Hyphantrophaga manuelriosi; phylum: Arthropoda; class: Insecta; order: Diptera; family: Tachinidae; genus: Hyphantrophaga; specificEpithet: manuelriosi; scientificNameAuthorship: Fleming & Wood, 2018; **Location:** continent: Central America; country: Costa Rica; countryCode: CR; stateProvince: Guanacaste; county: Sector Santa Rosa; locality: Area de Conservacion Guanacaste; verbatimLocality: Cafetal; verbatimElevation: 280; verbatimLatitude: 10.8583; verbatimLongitude: -85.6109; verbatimCoordinateSystem: Decimal; decimalLatitude: 10.8583; decimalLongitude: -85.6109; **Identification:** identifiedBy: AJ Fleming; dateIdentified: 2017; **Event:** samplingProtocol: Reared from the larva of the Crambidae, Syllepte belialis; verbatimEventDate: 06-Aug-1994; **Record Level:** language: en; institutionCode: CNC; collectionCode: Insects; basisOfRecord: Pinned Specimen
- **Type status:** Paratype. **Occurrence:** occurrenceDetails: http://janzen.sas.upenn.edu; catalogNumber: DHJPAR0007337; recordedBy: D.H. Janzen, W. Hallwachs & gusaneros; individualID: DHJPAR0007337; individualCount: 1; sex: female; lifeStage: adult; preparations: pinned; otherCatalogNumbers: ASTAT109-06, 93-SRNP-71, BOLD:AAA8930; **Taxon:** scientificName: Hyphantrophaga manuelriosi; phylum: Arthropoda; class: Insecta; order: Diptera; family: Tachinidae; genus: Hyphantrophaga; specificEpithet: manuelriosi; scientificNameAuthorship: Fleming & Wood, 2018; **Location:** continent: Central America; country: Costa Rica; countryCode: CR; stateProvince: Guanacaste; county: Sector Santa Rosa; locality: Area de Conservacion Guanacaste; verbatimLocality: Bosque Humedo; verbatimElevation: 290; verbatimLatitude: 10.8514; verbatimLongitude: -85.608; verbatimCoordinateSystem: Decimal; decimalLatitude: 10.8514; decimalLongitude: -85.608; **Identification:** identifiedBy: AJ Fleming; dateIdentified: 2017; **Event:** samplingProtocol: Reared from the larva of the Crambidae, Syllepte belialis; verbatimEventDate: 06-May-1993; **Record Level:** language: en; institutionCode: CNC; collectionCode: Insects; basisOfRecord: Pinned Specimen
- **Type status:** Paratype. **Occurrence:** occurrenceDetails: http://janzen.sas.upenn.edu; catalogNumber: DHJPAR0007338; recordedBy: D.H. Janzen, W. Hallwachs & gusaneros; individualID: DHJPAR0007338; individualCount: 1; sex: male; lifeStage: adult; preparations: pinned; otherCatalogNumbers: ASTAT110-06, 01-SRNP-13021, BOLD:AAA8930; **Taxon:** scientificName: Hyphantrophaga manuelriosi; phylum: Arthropoda; class: Insecta; order: Diptera; family: Tachinidae; genus: Hyphantrophaga; specificEpithet: manuelriosi; scientificNameAuthorship: Fleming & Wood, 2018; **Location:** continent: Central America; country: Costa Rica; countryCode: CR; stateProvince: Guanacaste; county: Sector Santa Rosa; locality: Area de Conservacion Guanacaste; verbatimLocality: Sendero Natural; verbatimElevation: 290; verbatimLatitude: 10.8357; verbatimLongitude: -85.6125; verbatimCoordinateSystem: Decimal; decimalLatitude: 10.8357; decimalLongitude: -85.6125; **Identification:** identifiedBy: AJ Fleming; dateIdentified: 2017; **Event:** samplingProtocol: Reared from the larva of the Crambidae, Syllepte belialis; verbatimEventDate: 02-May-2002; **Record Level:** language: en; institutionCode: CNC; collectionCode: Insects; basisOfRecord: Pinned Specimen
- **Type status:** Paratype. **Occurrence:** occurrenceDetails: http://janzen.sas.upenn.edu; catalogNumber: DHJPAR0007339; recordedBy: D.H. Janzen, W. Hallwachs & gusaneros; individualID: DHJPAR0007339; individualCount: 1; sex: male; lifeStage: adult; preparations: pinned; otherCatalogNumbers: ASTAT111-06, 88-SRNP-58.2, BOLD:AAA8930; **Taxon:** scientificName: Hyphantrophaga manuelriosi; phylum: Arthropoda; class: Insecta; order: Diptera; family: Tachinidae; genus: Hyphantrophaga; specificEpithet: manuelriosi; scientificNameAuthorship: Fleming & Wood, 2018; **Location:** continent: Central America; country: Costa Rica; countryCode: CR; stateProvince: Guanacaste; county: Sector Santa Rosa; locality: Area de Conservacion Guanacaste; verbatimLocality: Cafetal; verbatimElevation: 280; verbatimLatitude: 10.8583; verbatimLongitude: -85.6109; verbatimCoordinateSystem: Decimal; decimalLatitude: 10.8583; decimalLongitude: -85.6109; **Identification:** identifiedBy: AJ Fleming; dateIdentified: 2017; **Event:** samplingProtocol: Reared from the larva of the Crambidae, Syllepte belialis; verbatimEventDate: 17-Mar-1989; **Record Level:** language: en; institutionCode: CNC; collectionCode: Insects; basisOfRecord: Pinned Specimen
- **Type status:** Paratype. **Occurrence:** occurrenceDetails: http://janzen.sas.upenn.edu; catalogNumber: DHJPAR0007330; recordedBy: D.H. Janzen, W. Hallwachs & gusaneros; individualID: DHJPAR0007330; individualCount: 1; sex: male; lifeStage: adult; preparations: pinned; otherCatalogNumbers: ASTAT102-06, 01-SRNP-13276, BOLD:AAA8930; **Taxon:** scientificName: Hyphantrophaga manuelriosi; phylum: Arthropoda; class: Insecta; order: Diptera; family: Tachinidae; genus: Hyphantrophaga; specificEpithet: manuelriosi; scientificNameAuthorship: Fleming & Wood, 2018; **Location:** continent: Central America; country: Costa Rica; countryCode: CR; stateProvince: Guanacaste; county: Sector Santa Rosa; locality: Area de Conservacion Guanacaste; verbatimLocality: Sendero Natural; verbatimElevation: 290; verbatimLatitude: 10.8357; verbatimLongitude: -85.6125; verbatimCoordinateSystem: Decimal; decimalLatitude: 10.8357; decimalLongitude: -85.6125; **Identification:** identifiedBy: AJ Fleming; dateIdentified: 2017; **Event:** samplingProtocol: Reared from the larva of the Crambidae, Syllepte belialis; verbatimEventDate: 28-Apr-2002; **Record Level:** language: en; institutionCode: CNC; collectionCode: Insects; basisOfRecord: Pinned Specimen
- **Type status:** Paratype. **Occurrence:** occurrenceDetails: http://janzen.sas.upenn.edu; catalogNumber: DHJPAR0007341; recordedBy: D.H. Janzen, W. Hallwachs & gusaneros; individualID: DHJPAR0007341; individualCount: 1; sex: female; lifeStage: adult; preparations: pinned; otherCatalogNumbers: ASTAT113-06, 94-SRNP-1892, BOLD:AAA8930; **Taxon:** scientificName: Hyphantrophaga manuelriosi; phylum: Arthropoda; class: Insecta; order: Diptera; family: Tachinidae; genus: Hyphantrophaga; specificEpithet: manuelriosi; scientificNameAuthorship: Fleming & Wood, 2018; **Location:** continent: Central America; country: Costa Rica; countryCode: CR; stateProvince: Guanacaste; county: Sector Santa Rosa; locality: Area de Conservacion Guanacaste; verbatimLocality: Cafetal; verbatimElevation: 280; verbatimLatitude: 10.8583; verbatimLongitude: -85.6109; verbatimCoordinateSystem: Decimal; decimalLatitude: 10.8583; decimalLongitude: -85.6109; **Identification:** identifiedBy: AJ Fleming; dateIdentified: 2017; **Event:** samplingProtocol: Reared from the larva of the Crambidae, Syllepte belialis; verbatimEventDate: 24-Apr-1995; **Record Level:** language: en; institutionCode: CNC; collectionCode: Insects; basisOfRecord: Pinned Specimen
- **Type status:** Paratype. **Occurrence:** occurrenceDetails: http://janzen.sas.upenn.edu; catalogNumber: DHJPAR0007344; recordedBy: D.H. Janzen, W. Hallwachs & gusaneros; individualID: DHJPAR0007344; individualCount: 1; sex: male; lifeStage: adult; preparations: pinned; otherCatalogNumbers: ASTAT116-06, 94-SRNP-2054, BOLD:AAA8930; **Taxon:** scientificName: Hyphantrophaga manuelriosi; phylum: Arthropoda; class: Insecta; order: Diptera; family: Tachinidae; genus: Hyphantrophaga; specificEpithet: manuelriosi; scientificNameAuthorship: Fleming & Wood, 2018; **Location:** continent: Central America; country: Costa Rica; countryCode: CR; stateProvince: Guanacaste; county: Sector Santa Rosa; locality: Area de Conservacion Guanacaste; verbatimLocality: Cafetal; verbatimElevation: 280; verbatimLatitude: 10.8583; verbatimLongitude: -85.6109; verbatimCoordinateSystem: Decimal; decimalLatitude: 10.8583; decimalLongitude: -85.6109; **Identification:** identifiedBy: AJ Fleming; dateIdentified: 2017; **Event:** samplingProtocol: Reared from the larva of the Crambidae, Syllepte belialis; verbatimEventDate: 06-Sep-1994; **Record Level:** language: en; institutionCode: CNC; collectionCode: Insects; basisOfRecord: Pinned Specimen
- **Type status:** Paratype. **Occurrence:** occurrenceDetails: http://janzen.sas.upenn.edu; catalogNumber: DHJPAR0007345; recordedBy: D.H. Janzen, W. Hallwachs & gusaneros; individualID: DHJPAR0007345; individualCount: 1; sex: male; lifeStage: adult; preparations: pinned; otherCatalogNumbers: ASTAT117-06, 94-SRNP-1890, BOLD:AAA8930; **Taxon:** scientificName: Hyphantrophaga manuelriosi; phylum: Arthropoda; class: Insecta; order: Diptera; family: Tachinidae; genus: Hyphantrophaga; specificEpithet: manuelriosi; scientificNameAuthorship: Fleming & Wood, 2018; **Location:** continent: Central America; country: Costa Rica; countryCode: CR; stateProvince: Guanacaste; county: Sector Santa Rosa; locality: Area de Conservacion Guanacaste; verbatimLocality: Cafetal; verbatimElevation: 280; verbatimLatitude: 10.8583; verbatimLongitude: -85.6109; verbatimCoordinateSystem: Decimal; decimalLatitude: 10.8583; decimalLongitude: -85.6109; **Identification:** identifiedBy: AJ Fleming; dateIdentified: 2017; **Event:** samplingProtocol: Reared from the larva of the Crambidae, Syllepte belialis; verbatimEventDate: 16-Apr-1995; **Record Level:** language: en; institutionCode: CNC; collectionCode: Insects; basisOfRecord: Pinned Specimen
- **Type status:** Paratype. **Occurrence:** occurrenceDetails: http://janzen.sas.upenn.edu; catalogNumber: DHJPAR0007346; recordedBy: D.H. Janzen, W. Hallwachs & Freddy Quesada; individualID: DHJPAR0007346; individualCount: 1; sex: male; lifeStage: adult; preparations: pinned; otherCatalogNumbers: ASTAT118-06, 04-SRNP-10778, BOLD:AAA8930; **Taxon:** scientificName: Hyphantrophaga manuelriosi; phylum: Arthropoda; class: Insecta; order: Diptera; family: Tachinidae; genus: Hyphantrophaga; specificEpithet: manuelriosi; scientificNameAuthorship: Fleming & Wood, 2018; **Location:** continent: Central America; country: Costa Rica; countryCode: CR; stateProvince: Guanacaste; county: Sector Santa Rosa; locality: Area de Conservacion Guanacaste; verbatimLocality: Mirador Naranjo; verbatimElevation: 240; verbatimLatitude: 10.8064; verbatimLongitude: -85.6433; verbatimCoordinateSystem: Decimal; decimalLatitude: 10.8064; decimalLongitude: -85.6433; **Identification:** identifiedBy: AJ Fleming; dateIdentified: 2017; **Event:** samplingProtocol: Reared from the larva of the Crambidae, Syllepte belialis; verbatimEventDate: 25-Apr-2005; **Record Level:** language: en; institutionCode: CNC; collectionCode: Insects; basisOfRecord: Pinned Specimen
- **Type status:** Paratype. **Occurrence:** occurrenceDetails: http://janzen.sas.upenn.edu; catalogNumber: DHJPAR0007347; recordedBy: D.H. Janzen, W. Hallwachs & gusaneros; individualID: DHJPAR0007347; individualCount: 1; sex: male; lifeStage: adult; preparations: pinned; otherCatalogNumbers: ASTAT119-06, 94-SRNP-2059, BOLD:AAA8930; **Taxon:** scientificName: Hyphantrophaga manuelriosi; phylum: Arthropoda; class: Insecta; order: Diptera; family: Tachinidae; genus: Hyphantrophaga; specificEpithet: manuelriosi; scientificNameAuthorship: Fleming & Wood, 2018; **Location:** continent: Central America; country: Costa Rica; countryCode: CR; stateProvince: Guanacaste; county: Sector Santa Rosa; locality: Area de Conservacion Guanacaste; verbatimLocality: Cafetal; verbatimElevation: 280; verbatimLatitude: 10.8583; verbatimLongitude: -85.6109; verbatimCoordinateSystem: Decimal; decimalLatitude: 10.8583; decimalLongitude: -85.6109; **Identification:** identifiedBy: AJ Fleming; dateIdentified: 2017; **Event:** samplingProtocol: Reared from the larva of the Crambidae, Syllepte belialis; verbatimEventDate: 19-Apr-1995; **Record Level:** language: en; institutionCode: CNC; collectionCode: Insects; basisOfRecord: Pinned Specimen
- **Type status:** Paratype. **Occurrence:** occurrenceDetails: http://janzen.sas.upenn.edu; catalogNumber: DHJPAR0007348; recordedBy: D.H. Janzen, W. Hallwachs & gusaneros; individualID: DHJPAR0007348; individualCount: 1; sex: female; lifeStage: adult; preparations: pinned; otherCatalogNumbers: ASTAT120-06, 94-SRNP-2044, BOLD:AAA8930; **Taxon:** scientificName: Hyphantrophaga manuelriosi; phylum: Arthropoda; class: Insecta; order: Diptera; family: Tachinidae; genus: Hyphantrophaga; specificEpithet: manuelriosi; scientificNameAuthorship: Fleming & Wood, 2018; **Location:** continent: Central America; country: Costa Rica; countryCode: CR; stateProvince: Guanacaste; county: Sector Santa Rosa; locality: Area de Conservacion Guanacaste; verbatimLocality: Cafetal; verbatimElevation: 280; verbatimLatitude: 10.8583; verbatimLongitude: -85.6109; verbatimCoordinateSystem: Decimal; decimalLatitude: 10.8583; decimalLongitude: -85.6109; **Identification:** identifiedBy: AJ Fleming; dateIdentified: 2017; **Event:** samplingProtocol: Reared from the larva of the Crambidae, Syllepte belialis; verbatimEventDate: 04-Aug-1995; **Record Level:** language: en; institutionCode: CNC; collectionCode: Insects; basisOfRecord: Pinned Specimen
- **Type status:** Paratype. **Occurrence:** occurrenceDetails: http://janzen.sas.upenn.edu; catalogNumber: DHJPAR0019677; recordedBy: D.H. Janzen, W. Hallwachs & Jose Alberto Sanchez; individualID: DHJPAR0019677; individualCount: 1; sex: male; lifeStage: adult; preparations: pinned; otherCatalogNumbers: ASTAB225-07, 07-SRNP-56154, BOLD:AAA8930; **Taxon:** scientificName: Hyphantrophaga manuelriosi; phylum: Arthropoda; class: Insecta; order: Diptera; family: Tachinidae; genus: Hyphantrophaga; specificEpithet: manuelriosi; scientificNameAuthorship: Fleming & Wood, 2018; **Location:** continent: Central America; country: Costa Rica; countryCode: CR; stateProvince: Guanacaste; county: Sector Mundo Nuevo; locality: Area de Conservacion Guanacaste; verbatimLocality: Sendero Mora; verbatimElevation: 480; verbatimLatitude: 10.7683; verbatimLongitude: -85.4257; verbatimCoordinateSystem: Decimal; decimalLatitude: 10.7683; decimalLongitude: -85.4257; **Identification:** identifiedBy: AJ Fleming; dateIdentified: 2017; **Event:** samplingProtocol: Reared from the larva of the Crambidae, Syllepte belialis; verbatimEventDate: 28-May-2007; **Record Level:** language: en; institutionCode: CNC; collectionCode: Insects; basisOfRecord: Pinned Specimen
- **Type status:** Paratype. **Occurrence:** occurrenceDetails: http://janzen.sas.upenn.edu; catalogNumber: DHJPAR0019679; recordedBy: D.H. Janzen, W. Hallwachs & Jose Alberto Sanchez; individualID: DHJPAR0019679; individualCount: 1; sex: male; lifeStage: adult; preparations: pinned; otherCatalogNumbers: ASTAB227-07, 07-SRNP-56157, BOLD:AAA8930; **Taxon:** scientificName: Hyphantrophaga manuelriosi; phylum: Arthropoda; class: Insecta; order: Diptera; family: Tachinidae; genus: Hyphantrophaga; specificEpithet: manuelriosi; scientificNameAuthorship: Fleming & Wood, 2018; **Location:** continent: Central America; country: Costa Rica; countryCode: CR; stateProvince: Guanacaste; county: Sector Mundo Nuevo; locality: Area de Conservacion Guanacaste; verbatimLocality: Sendero Mora; verbatimElevation: 480; verbatimLatitude: 10.7683; verbatimLongitude: -85.4257; verbatimCoordinateSystem: Decimal; decimalLatitude: 10.7683; decimalLongitude: -85.4257; **Identification:** identifiedBy: AJ Fleming; dateIdentified: 2017; **Event:** samplingProtocol: Reared from the larva of the Crambidae, Syllepte belialis; verbatimEventDate: 25-May-2007; **Record Level:** language: en; institutionCode: CNC; collectionCode: Insects; basisOfRecord: Pinned Specimen
- **Type status:** Paratype. **Occurrence:** occurrenceDetails: http://janzen.sas.upenn.edu; catalogNumber: DHJPAR0030460; recordedBy: D.H. Janzen, W. Hallwachs & Lucia Vargas; individualID: DHJPAR0030460; individualCount: 1; sex: male; lifeStage: adult; preparations: pinned; otherCatalogNumbers: ASHYB1203-09, 08-SRNP-12948, BOLD:AAA8930; **Taxon:** scientificName: Hyphantrophaga manuelriosi; phylum: Arthropoda; class: Insecta; order: Diptera; family: Tachinidae; genus: Hyphantrophaga; specificEpithet: manuelriosi; scientificNameAuthorship: Fleming & Wood, 2018; **Location:** continent: Central America; country: Costa Rica; countryCode: CR; stateProvince: Guanacaste; county: Sector Santa Rosa; locality: Area de Conservacion Guanacaste; verbatimLocality: Sendero Natural; verbatimElevation: 290; verbatimLatitude: 10.8357; verbatimLongitude: -85.6125; verbatimCoordinateSystem: Decimal; decimalLatitude: 10.8357; decimalLongitude: -85.6125; **Identification:** identifiedBy: AJ Fleming; dateIdentified: 2017; **Event:** samplingProtocol: Reared from the larva of the Crambidae, Syllepte belialis; verbatimEventDate: 09-Apr-2009; **Record Level:** language: en; institutionCode: CNC; collectionCode: Insects; basisOfRecord: Pinned Specimen
- **Type status:** Paratype. **Occurrence:** occurrenceDetails: http://janzen.sas.upenn.edu; catalogNumber: DHJPAR0039306; recordedBy: D.H. Janzen, W. Hallwachs & Mariano Pereira; individualID: DHJPAR0039306; individualCount: 1; sex: female; lifeStage: adult; preparations: pinned; otherCatalogNumbers: ASTAV869-10, 10-SRNP-55351, BOLD:AAA8930; **Taxon:** scientificName: Hyphantrophaga manuelriosi; phylum: Arthropoda; class: Insecta; order: Diptera; family: Tachinidae; genus: Hyphantrophaga; specificEpithet: manuelriosi; scientificNameAuthorship: Fleming & Wood, 2018; **Location:** continent: Central America; country: Costa Rica; countryCode: CR; stateProvince: Guanacaste; county: Sector Mundo Nuevo; locality: Area de Conservacion Guanacaste; verbatimLocality: Estacion La Perla; verbatimElevation: 325; verbatimLatitude: 10.7674; verbatimLongitude: -85.4331; verbatimCoordinateSystem: Decimal; decimalLatitude: 10.7674; decimalLongitude: -85.4331; **Identification:** identifiedBy: AJ Fleming; dateIdentified: 2017; **Event:** samplingProtocol: Reared from the larva of the Crambidae, Syllepte belialis; verbatimEventDate: 13-Jun-2010; **Record Level:** language: en; institutionCode: CNC; collectionCode: Insects; basisOfRecord: Pinned Specimen
- **Type status:** Paratype. **Occurrence:** occurrenceDetails: http://janzen.sas.upenn.edu; catalogNumber: DHJPAR0056209; recordedBy: D.H. Janzen, W. Hallwachs & Jose Cortez; individualID: DHJPAR0056209; individualCount: 1; sex: male; lifeStage: adult; preparations: pinned; otherCatalogNumbers: ASHYH2466-14, 14-SRNP-55897, BOLD:AAA8930; **Taxon:** scientificName: Hyphantrophaga manuelriosi; phylum: Arthropoda; class: Insecta; order: Diptera; family: Tachinidae; genus: Hyphantrophaga; specificEpithet: manuelriosi; scientificNameAuthorship: Fleming & Wood, 2018; **Location:** continent: Central America; country: Costa Rica; countryCode: CR; stateProvince: Guanacaste; county: Sector Mundo Nuevo; locality: Area de Conservacion Guanacaste; verbatimLocality: Sendero Aguacate; verbatimElevation: 335; verbatimLatitude: 10.769; verbatimLongitude: -85.4346; verbatimCoordinateSystem: Decimal; decimalLatitude: 10.769; decimalLongitude: -85.4346; **Identification:** identifiedBy: AJ Fleming; dateIdentified: 2017; **Event:** samplingProtocol: Reared from the larva of the Crambidae, Syllepte belialis; verbatimEventDate: 11-Sep-2014; **Record Level:** language: en; institutionCode: CNC; collectionCode: Insects; basisOfRecord: Pinned Specimen
- **Type status:** Paratype. **Occurrence:** occurrenceDetails: http://janzen.sas.upenn.edu; catalogNumber: DHJPAR0056220; recordedBy: D.H. Janzen, W. Hallwachs & Jose Cortez; individualID: DHJPAR0056220; individualCount: 1; sex: female; lifeStage: adult; preparations: pinned; otherCatalogNumbers: ASHYH2477-14, 14-SRNP-55899, BOLD:AAA8930; **Taxon:** scientificName: Hyphantrophaga manuelriosi; phylum: Arthropoda; class: Insecta; order: Diptera; family: Tachinidae; genus: Hyphantrophaga; specificEpithet: manuelriosi; scientificNameAuthorship: Fleming & Wood, 2018; **Location:** continent: Central America; country: Costa Rica; countryCode: CR; stateProvince: Guanacaste; county: Sector Mundo Nuevo; locality: Area de Conservacion Guanacaste; verbatimLocality: Sendero Aguacate; verbatimElevation: 335; verbatimLatitude: 10.769; verbatimLongitude: -85.4346; verbatimCoordinateSystem: Decimal; decimalLatitude: 10.769; decimalLongitude: -85.4346; **Identification:** identifiedBy: AJ Fleming; dateIdentified: 2017; **Event:** samplingProtocol: Reared from the larva of the Crambidae, Syllepte belialis; verbatimEventDate: 10-Sep-2014; **Record Level:** language: en; institutionCode: CNC; collectionCode: Insects; basisOfRecord: Pinned Specimen
- **Type status:** Paratype. **Occurrence:** occurrenceDetails: http://janzen.sas.upenn.edu; catalogNumber: DHJPAR0055853; recordedBy: D.H. Janzen, W. Hallwachs & Jose Cortez; individualID: DHJPAR0055853; individualCount: 1; sex: female; lifeStage: adult; preparations: pinned; otherCatalogNumbers: ASHYH2585-14, 14-SRNP-55853, BOLD:AAA8930; **Taxon:** scientificName: Hyphantrophaga manuelriosi; phylum: Arthropoda; class: Insecta; order: Diptera; family: Tachinidae; genus: Hyphantrophaga; specificEpithet: manuelriosi; scientificNameAuthorship: Fleming & Wood, 2018; **Location:** continent: Central America; country: Costa Rica; countryCode: CR; stateProvince: Guanacaste; county: Sector Mundo Nuevo; locality: Area de Conservacion Guanacaste; verbatimLocality: Punta Plancha; verbatimElevation: 420; verbatimLatitude: 10.7416; verbatimLongitude: -85.4273; verbatimCoordinateSystem: Decimal; decimalLatitude: 10.7416; decimalLongitude: -85.4273; **Identification:** identifiedBy: AJ Fleming; dateIdentified: 2017; **Event:** samplingProtocol: Reared from the larva of the Crambidae, Syllepte belialis; verbatimEventDate: 05-Aug-2014; **Record Level:** language: en; institutionCode: CNC; collectionCode: Insects; basisOfRecord: Pinned Specimen
- **Type status:** Paratype. **Occurrence:** occurrenceDetails: http://janzen.sas.upenn.edu; catalogNumber: DHJPAR0055855; recordedBy: D.H. Janzen, W. Hallwachs & Jose Cortez; individualID: DHJPAR0055855; individualCount: 1; sex: male; lifeStage: adult; preparations: pinned; otherCatalogNumbers: ASHYH2587-14, 14-SRNP-55890, BOLD:AAA8930; **Taxon:** scientificName: Hyphantrophaga manuelriosi; phylum: Arthropoda; class: Insecta; order: Diptera; family: Tachinidae; genus: Hyphantrophaga; specificEpithet: manuelriosi; scientificNameAuthorship: Fleming & Wood, 2018; **Location:** continent: Central America; country: Costa Rica; countryCode: CR; stateProvince: Guanacaste; county: Sector Mundo Nuevo; locality: Area de Conservacion Guanacaste; verbatimLocality: Sendero Aguacate; verbatimElevation: 335; verbatimLatitude: 10.769; verbatimLongitude: -85.4346; verbatimCoordinateSystem: Decimal; decimalLatitude: 10.769; decimalLongitude: -85.4346; **Identification:** identifiedBy: AJ Fleming; dateIdentified: 2017; **Event:** samplingProtocol: Reared from the larva of the Crambidae, Syllepte belialis; verbatimEventDate: 26-Jul-2014; **Record Level:** language: en; institutionCode: CNC; collectionCode: Insects; basisOfRecord: Pinned Specimen
- **Type status:** Paratype. **Occurrence:** occurrenceDetails: http://janzen.sas.upenn.edu; catalogNumber: DHJPAR0055857; recordedBy: D.H. Janzen, W. Hallwachs & Jose Cortez; individualID: DHJPAR0055857; individualCount: 1; sex: male; lifeStage: adult; preparations: pinned; otherCatalogNumbers: ASHYH2589-14, 14-SRNP-55898, BOLD:AAA8930; **Taxon:** scientificName: Hyphantrophaga manuelriosi; phylum: Arthropoda; class: Insecta; order: Diptera; family: Tachinidae; genus: Hyphantrophaga; specificEpithet: manuelriosi; scientificNameAuthorship: Fleming & Wood, 2018; **Location:** continent: Central America; country: Costa Rica; countryCode: CR; stateProvince: Guanacaste; county: Sector Mundo Nuevo; locality: Area de Conservacion Guanacaste; verbatimLocality: Sendero Aguacate; verbatimElevation: 335; verbatimLatitude: 10.769; verbatimLongitude: -85.4346; verbatimCoordinateSystem: Decimal; decimalLatitude: 10.769; decimalLongitude: -85.4346; **Identification:** identifiedBy: AJ Fleming; dateIdentified: 2017; **Event:** samplingProtocol: Reared from the larva of the Crambidae, Syllepte belialis; verbatimEventDate: 15-Jul-2014; **Record Level:** language: en; institutionCode: CNC; collectionCode: Insects; basisOfRecord: Pinned Specimen
- **Type status:** Other material. **Occurrence:** occurrenceDetails: http://janzen.sas.upenn.edu; catalogNumber: DHJPAR0034334; recordedBy: D.H. Janzen, W. Hallwachs & Lucia Vargas; individualID: DHJPAR0034334; individualCount: 1; lifeStage: adult; preparations: pinned; otherCatalogNumbers: ASHYB1627-09, 08-SRNP-12941, BOLD:AAA8930; **Taxon:** scientificName: Hyphantrophaga manuelriosi; phylum: Arthropoda; class: Insecta; order: Diptera; family: Tachinidae; genus: Hyphantrophaga; specificEpithet: manuelriosi; scientificNameAuthorship: Fleming & Wood, 2018; **Location:** continent: Central America; country: Costa Rica; countryCode: CR; stateProvince: Guanacaste; county: Sector Santa Rosa; locality: Area de Conservacion Guanacaste; verbatimLocality: Sendero Natural; verbatimElevation: 290; verbatimLatitude: 10.8357; verbatimLongitude: -85.6125; verbatimCoordinateSystem: Decimal; decimalLatitude: 10.8357; decimalLongitude: -85.6125; **Identification:** identifiedBy: AJ Fleming; dateIdentified: 2017; **Event:** samplingProtocol: Reared from the larva of the Crambidae, Syllepte belialis; verbatimEventDate: 09-Apr-2009; **Record Level:** language: en; institutionCode: CNC; collectionCode: Insects; basisOfRecord: Pinned Specimen
- **Type status:** Other material. **Occurrence:** occurrenceDetails: http://janzen.sas.upenn.edu; catalogNumber: DHJPAR0035565; recordedBy: D.H. Janzen, W. Hallwachs & Lucia Vargas; individualID: DHJPAR0035565; individualCount: 1; lifeStage: adult; preparations: pinned; otherCatalogNumbers: ASHYC1770-09, 08-SRNP-12897, BOLD:AAA8930; **Taxon:** scientificName: Hyphantrophaga manuelriosi; phylum: Arthropoda; class: Insecta; order: Diptera; family: Tachinidae; genus: Hyphantrophaga; specificEpithet: manuelriosi; scientificNameAuthorship: Fleming & Wood, 2018; **Location:** continent: Central America; country: Costa Rica; countryCode: CR; stateProvince: Guanacaste; county: Sector Santa Rosa; locality: Area de Conservacion Guanacaste; verbatimLocality: Area Administrativa; verbatimElevation: 295; verbatimLatitude: 10.8376; verbatimLongitude: -85.6187; verbatimCoordinateSystem: Decimal; decimalLatitude: 10.8376; decimalLongitude: -85.6187; **Identification:** identifiedBy: AJ Fleming; dateIdentified: 2017; **Event:** samplingProtocol: Reared from the larva of the Crambidae, Syllepte belialis; verbatimEventDate: 17-May-2009; **Record Level:** language: en; institutionCode: CNC; collectionCode: Insects; basisOfRecord: Pinned Specimen
- **Type status:** Other material. **Occurrence:** occurrenceDetails: http://janzen.sas.upenn.edu; catalogNumber: DHJPAR0035567; recordedBy: D.H. Janzen, W. Hallwachs & Lucia Vargas; individualID: DHJPAR0035567; individualCount: 1; lifeStage: adult; preparations: pinned; otherCatalogNumbers: ASHYC1772-09, 08-SRNP-12900, BOLD:AAA8930; **Taxon:** scientificName: Hyphantrophaga manuelriosi; phylum: Arthropoda; class: Insecta; order: Diptera; family: Tachinidae; genus: Hyphantrophaga; specificEpithet: manuelriosi; scientificNameAuthorship: Fleming & Wood, 2018; **Location:** continent: Central America; country: Costa Rica; countryCode: CR; stateProvince: Guanacaste; county: Sector Santa Rosa; locality: Area de Conservacion Guanacaste; verbatimLocality: Area Administrativa; verbatimElevation: 295; verbatimLatitude: 10.8376; verbatimLongitude: -85.6187; verbatimCoordinateSystem: Decimal; decimalLatitude: 10.8376; decimalLongitude: -85.6187; **Identification:** identifiedBy: AJ Fleming; dateIdentified: 2017; **Event:** samplingProtocol: Reared from the larva of the Crambidae, Syllepte belialis; verbatimEventDate: 29-Apr-2009; **Record Level:** language: en; institutionCode: CNC; collectionCode: Insects; basisOfRecord: Pinned Specimen
- **Type status:** Other material. **Occurrence:** occurrenceDetails: http://janzen.sas.upenn.edu; catalogNumber: DHJPAR0035568; recordedBy: D.H. Janzen, W. Hallwachs & Lucia Vargas; individualID: DHJPAR0035568; individualCount: 1; lifeStage: adult; preparations: pinned; otherCatalogNumbers: ASHYC1773-09, 08-SRNP-12905, BOLD:AAA8930; **Taxon:** scientificName: Hyphantrophaga manuelriosi; phylum: Arthropoda; class: Insecta; order: Diptera; family: Tachinidae; genus: Hyphantrophaga; specificEpithet: manuelriosi; scientificNameAuthorship: Fleming & Wood, 2018; **Location:** continent: Central America; country: Costa Rica; countryCode: CR; stateProvince: Guanacaste; county: Sector Santa Rosa; locality: Area de Conservacion Guanacaste; verbatimLocality: Area Administrativa; verbatimElevation: 295; verbatimLatitude: 10.8376; verbatimLongitude: -85.6187; verbatimCoordinateSystem: Decimal; decimalLatitude: 10.8376; decimalLongitude: -85.6187; **Identification:** identifiedBy: AJ Fleming; dateIdentified: 2017; **Event:** samplingProtocol: Reared from the larva of the Crambidae, Syllepte belialis; verbatimEventDate: 14-Apr-2009; **Record Level:** language: en; institutionCode: CNC; collectionCode: Insects; basisOfRecord: Pinned Specimen
- **Type status:** Other material. **Occurrence:** occurrenceDetails: http://janzen.sas.upenn.edu; catalogNumber: DHJPAR0035570; recordedBy: D.H. Janzen, W. Hallwachs & Lucia Vargas; individualID: DHJPAR0035570; individualCount: 1; lifeStage: adult; preparations: pinned; otherCatalogNumbers: ASHYC1775-09, 08-SRNP-12940, BOLD:AAA8930; **Taxon:** scientificName: Hyphantrophaga manuelriosi; phylum: Arthropoda; class: Insecta; order: Diptera; family: Tachinidae; genus: Hyphantrophaga; specificEpithet: manuelriosi; scientificNameAuthorship: Fleming & Wood, 2018; **Location:** continent: Central America; country: Costa Rica; countryCode: CR; stateProvince: Guanacaste; county: Sector Santa Rosa; locality: Area de Conservacion Guanacaste; verbatimLocality: Sendero Natural; verbatimElevation: 290; verbatimLatitude: 10.8357; verbatimLongitude: -85.6125; verbatimCoordinateSystem: Decimal; decimalLatitude: 10.8357; decimalLongitude: -85.6125; **Identification:** identifiedBy: AJ Fleming; dateIdentified: 2017; **Event:** samplingProtocol: Reared from the larva of the Crambidae, Syllepte belialis; verbatimEventDate: 14-May-2009; **Record Level:** language: en; institutionCode: CNC; collectionCode: Insects; basisOfRecord: Pinned Specimen
- **Type status:** Other material. **Occurrence:** occurrenceDetails: http://janzen.sas.upenn.edu; catalogNumber: DHJPAR0035571; recordedBy: D.H. Janzen, W. Hallwachs & Guillermo Pereira; individualID: DHJPAR0035571; individualCount: 1; lifeStage: adult; preparations: pinned; otherCatalogNumbers: ASHYC1776-09, 08-SRNP-13153, BOLD:AAA8930; **Taxon:** scientificName: Hyphantrophaga manuelriosi; phylum: Arthropoda; class: Insecta; order: Diptera; family: Tachinidae; genus: Hyphantrophaga; specificEpithet: manuelriosi; scientificNameAuthorship: Fleming & Wood, 2018; **Location:** continent: Central America; country: Costa Rica; countryCode: CR; stateProvince: Guanacaste; county: Sector Santa Rosa; locality: Area de Conservacion Guanacaste; verbatimLocality: Sendero Natural; verbatimElevation: 290; verbatimLatitude: 10.8357; verbatimLongitude: -85.6125; verbatimCoordinateSystem: Decimal; decimalLatitude: 10.8357; decimalLongitude: -85.6125; **Identification:** identifiedBy: AJ Fleming; dateIdentified: 2017; **Event:** samplingProtocol: Reared from the larva of the Crambidae, Syllepte belialis; verbatimEventDate: 12-May-2009; **Record Level:** language: en; institutionCode: CNC; collectionCode: Insects; basisOfRecord: Pinned Specimen
- **Type status:** Other material. **Occurrence:** occurrenceDetails: http://janzen.sas.upenn.edu; catalogNumber: DHJPAR0035572; recordedBy: D.H. Janzen, W. Hallwachs & Guillermo Pereira; individualID: DHJPAR0035572; individualCount: 1; lifeStage: adult; preparations: pinned; otherCatalogNumbers: ASHYC1777-09, 08-SRNP-13170, BOLD:AAA8930; **Taxon:** scientificName: Hyphantrophaga manuelriosi; phylum: Arthropoda; class: Insecta; order: Diptera; family: Tachinidae; genus: Hyphantrophaga; specificEpithet: manuelriosi; scientificNameAuthorship: Fleming & Wood, 2018; **Location:** continent: Central America; country: Costa Rica; countryCode: CR; stateProvince: Guanacaste; county: Sector Santa Rosa; locality: Area de Conservacion Guanacaste; verbatimLocality: Sendero Natural; verbatimElevation: 290; verbatimLatitude: 10.8357; verbatimLongitude: -85.6125; verbatimCoordinateSystem: Decimal; decimalLatitude: 10.8357; decimalLongitude: -85.6125; **Identification:** identifiedBy: AJ Fleming; dateIdentified: 2017; **Event:** samplingProtocol: Reared from the larva of the Crambidae, Syllepte belialis; verbatimEventDate: 09-May-2009; **Record Level:** language: en; institutionCode: CNC; collectionCode: Insects; basisOfRecord: Pinned Specimen
- **Type status:** Other material. **Occurrence:** occurrenceDetails: http://janzen.sas.upenn.edu; catalogNumber: DHJPAR0035573; recordedBy: D.H. Janzen, W. Hallwachs & Lucia Vargas; individualID: DHJPAR0035573; individualCount: 1; lifeStage: adult; preparations: pinned; otherCatalogNumbers: ASHYC1778-09, 08-SRNP-12902, BOLD:AAA8930; **Taxon:** scientificName: Hyphantrophaga manuelriosi; phylum: Arthropoda; class: Insecta; order: Diptera; family: Tachinidae; genus: Hyphantrophaga; specificEpithet: manuelriosi; scientificNameAuthorship: Fleming & Wood, 2018; **Location:** continent: Central America; country: Costa Rica; countryCode: CR; stateProvince: Guanacaste; county: Sector Santa Rosa; locality: Area de Conservacion Guanacaste; verbatimLocality: Area Administrativa; verbatimElevation: 295; verbatimLatitude: 10.8376; verbatimLongitude: -85.6187; verbatimCoordinateSystem: Decimal; decimalLatitude: 10.8376; decimalLongitude: -85.6187; **Identification:** identifiedBy: AJ Fleming; dateIdentified: 2017; **Event:** samplingProtocol: Reared from the larva of the Crambidae, Syllepte belialis; verbatimEventDate: 14-May-2009; **Record Level:** language: en; institutionCode: CNC; collectionCode: Insects; basisOfRecord: Pinned Specimen
- **Type status:** Other material. **Occurrence:** occurrenceDetails: http://janzen.sas.upenn.edu; catalogNumber: DHJPAR0035576; recordedBy: D.H. Janzen, W. Hallwachs & Lucia Vargas; individualID: DHJPAR0035576; individualCount: 1; lifeStage: adult; preparations: pinned; otherCatalogNumbers: ASHYC1781-09, 08-SRNP-12906, BOLD:AAA8930; **Taxon:** scientificName: Hyphantrophaga manuelriosi; phylum: Arthropoda; class: Insecta; order: Diptera; family: Tachinidae; genus: Hyphantrophaga; specificEpithet: manuelriosi; scientificNameAuthorship: Fleming & Wood, 2018; **Location:** continent: Central America; country: Costa Rica; countryCode: CR; stateProvince: Guanacaste; county: Sector Santa Rosa; locality: Area de Conservacion Guanacaste; verbatimLocality: Area Administrativa; verbatimElevation: 295; verbatimLatitude: 10.8376; verbatimLongitude: -85.6187; verbatimCoordinateSystem: Decimal; decimalLatitude: 10.8376; decimalLongitude: -85.6187; **Identification:** identifiedBy: AJ Fleming; dateIdentified: 2017; **Event:** samplingProtocol: Reared from the larva of the Crambidae, Syllepte belialis; verbatimEventDate: 14-Apr-2009; **Record Level:** language: en; institutionCode: CNC; collectionCode: Insects; basisOfRecord: Pinned Specimen
- **Type status:** Other material. **Occurrence:** occurrenceDetails: http://janzen.sas.upenn.edu; catalogNumber: DHJPAR0035577; recordedBy: D.H. Janzen, W. Hallwachs & Lucia Vargas; individualID: DHJPAR0035577; individualCount: 1; lifeStage: adult; preparations: pinned; otherCatalogNumbers: ASHYC1782-09, 08-SRNP-12912, BOLD:AAA8930; **Taxon:** scientificName: Hyphantrophaga manuelriosi; phylum: Arthropoda; class: Insecta; order: Diptera; family: Tachinidae; genus: Hyphantrophaga; specificEpithet: manuelriosi; scientificNameAuthorship: Fleming & Wood, 2018; **Location:** continent: Central America; country: Costa Rica; countryCode: CR; stateProvince: Guanacaste; county: Sector Santa Rosa; locality: Area de Conservacion Guanacaste; verbatimLocality: Area Administrativa; verbatimElevation: 295; verbatimLatitude: 10.8376; verbatimLongitude: -85.6187; verbatimCoordinateSystem: Decimal; decimalLatitude: 10.8376; decimalLongitude: -85.6187; **Identification:** identifiedBy: AJ Fleming; dateIdentified: 2017; **Event:** samplingProtocol: Reared from the larva of the Crambidae, Syllepte belialis; verbatimEventDate: 17-May-2009; **Record Level:** language: en; institutionCode: CNC; collectionCode: Insects; basisOfRecord: Pinned Specimen
- **Type status:** Other material. **Occurrence:** occurrenceDetails: http://janzen.sas.upenn.edu; catalogNumber: DHJPAR0035578; recordedBy: D.H. Janzen, W. Hallwachs & Lucia Vargas; individualID: DHJPAR0035578; individualCount: 1; lifeStage: adult; preparations: pinned; otherCatalogNumbers: ASHYC1783-09, 08-SRNP-12915, BOLD:AAA8930; **Taxon:** scientificName: Hyphantrophaga manuelriosi; phylum: Arthropoda; class: Insecta; order: Diptera; family: Tachinidae; genus: Hyphantrophaga; specificEpithet: manuelriosi; scientificNameAuthorship: Fleming & Wood, 2018; **Location:** continent: Central America; country: Costa Rica; countryCode: CR; stateProvince: Guanacaste; county: Sector Santa Rosa; locality: Area de Conservacion Guanacaste; verbatimLocality: Area Administrativa; verbatimElevation: 295; verbatimLatitude: 10.8376; verbatimLongitude: -85.6187; verbatimCoordinateSystem: Decimal; decimalLatitude: 10.8376; decimalLongitude: -85.6187; **Identification:** identifiedBy: AJ Fleming; dateIdentified: 2017; **Event:** samplingProtocol: Reared from the larva of the Crambidae, Syllepte belialis; verbatimEventDate: 17-Apr-2009; **Record Level:** language: en; institutionCode: CNC; collectionCode: Insects; basisOfRecord: Pinned Specimen
- **Type status:** Other material. **Occurrence:** occurrenceDetails: http://janzen.sas.upenn.edu; catalogNumber: DHJPAR0036440; recordedBy: D.H. Janzen, W. Hallwachs & Lucia Vargas; individualID: DHJPAR0036440; individualCount: 1; lifeStage: adult; preparations: pinned; otherCatalogNumbers: ASHYD1631-09, 08-SRNP-12914, BOLD:AAA8930; **Taxon:** scientificName: Hyphantrophaga manuelriosi; phylum: Arthropoda; class: Insecta; order: Diptera; family: Tachinidae; genus: Hyphantrophaga; specificEpithet: manuelriosi; scientificNameAuthorship: Fleming & Wood, 2018; **Location:** continent: Central America; country: Costa Rica; countryCode: CR; stateProvince: Guanacaste; county: Sector Santa Rosa; locality: Area de Conservacion Guanacaste; verbatimLocality: Area Administrativa; verbatimElevation: 295; verbatimLatitude: 10.8376; verbatimLongitude: -85.6187; verbatimCoordinateSystem: Decimal; decimalLatitude: 10.8376; decimalLongitude: -85.6187; **Identification:** identifiedBy: AJ Fleming; dateIdentified: 2017; **Event:** samplingProtocol: Reared from the larva of the Crambidae, Syllepte belialis; verbatimEventDate: 14-May-2009; **Record Level:** language: en; institutionCode: CNC; collectionCode: Insects; basisOfRecord: Pinned Specimen
- **Type status:** Other material. **Occurrence:** occurrenceDetails: http://janzen.sas.upenn.edu; catalogNumber: DHJPAR0036445; recordedBy: D.H. Janzen, W. Hallwachs & Guillermo Pereira; individualID: DHJPAR0036445; individualCount: 1; lifeStage: adult; preparations: pinned; otherCatalogNumbers: ASHYD1636-09, 08-SRNP-13159, BOLD:AAA8930; **Taxon:** scientificName: Hyphantrophaga manuelriosi; phylum: Arthropoda; class: Insecta; order: Diptera; family: Tachinidae; genus: Hyphantrophaga; specificEpithet: manuelriosi; scientificNameAuthorship: Fleming & Wood, 2018; **Location:** continent: Central America; country: Costa Rica; countryCode: CR; stateProvince: Guanacaste; county: Sector Santa Rosa; locality: Area de Conservacion Guanacaste; verbatimLocality: Sendero Natural; verbatimElevation: 290; verbatimLatitude: 10.8357; verbatimLongitude: -85.6125; verbatimCoordinateSystem: Decimal; decimalLatitude: 10.8357; decimalLongitude: -85.6125; **Identification:** identifiedBy: AJ Fleming; dateIdentified: 2017; **Event:** samplingProtocol: Reared from the larva of the Crambidae, Syllepte belialis; verbatimEventDate: 09-May-2009; **Record Level:** language: en; institutionCode: CNC; collectionCode: Insects; basisOfRecord: Pinned Specimen
- **Type status:** Other material. **Occurrence:** occurrenceDetails: http://janzen.sas.upenn.edu; catalogNumber: DHJPAR0036447; recordedBy: D.H. Janzen, W. Hallwachs & Guillermo Pereira; individualID: DHJPAR0036447; individualCount: 1; lifeStage: adult; preparations: pinned; otherCatalogNumbers: ASHYD1638-09, 08-SRNP-13162, BOLD:AAA8930; **Taxon:** scientificName: Hyphantrophaga manuelriosi; phylum: Arthropoda; class: Insecta; order: Diptera; family: Tachinidae; genus: Hyphantrophaga; specificEpithet: manuelriosi; scientificNameAuthorship: Fleming & Wood, 2018; **Location:** continent: Central America; country: Costa Rica; countryCode: CR; stateProvince: Guanacaste; county: Sector Santa Rosa; locality: Area de Conservacion Guanacaste; verbatimLocality: Sendero Natural; verbatimElevation: 290; verbatimLatitude: 10.8357; verbatimLongitude: -85.6125; verbatimCoordinateSystem: Decimal; decimalLatitude: 10.8357; decimalLongitude: -85.6125; **Identification:** identifiedBy: AJ Fleming; dateIdentified: 2017; **Event:** samplingProtocol: Reared from the larva of the Crambidae, Syllepte belialis; verbatimEventDate: 24-Apr-2009; **Record Level:** language: en; institutionCode: CNC; collectionCode: Insects; basisOfRecord: Pinned Specimen
- **Type status:** Other material. **Occurrence:** occurrenceDetails: http://janzen.sas.upenn.edu; catalogNumber: DHJPAR0036448; recordedBy: D.H. Janzen, W. Hallwachs & Lucia Vargas; individualID: DHJPAR0036448; individualCount: 1; lifeStage: adult; preparations: pinned; otherCatalogNumbers: ASHYD1639-09, 08-SRNP-12928, BOLD:AAA8930; **Taxon:** scientificName: Hyphantrophaga manuelriosi; phylum: Arthropoda; class: Insecta; order: Diptera; family: Tachinidae; genus: Hyphantrophaga; specificEpithet: manuelriosi; scientificNameAuthorship: Fleming & Wood, 2018; **Location:** continent: Central America; country: Costa Rica; countryCode: CR; stateProvince: Guanacaste; county: Sector Santa Rosa; locality: Area de Conservacion Guanacaste; verbatimLocality: Area Administrativa; verbatimElevation: 295; verbatimLatitude: 10.8376; verbatimLongitude: -85.6187; verbatimCoordinateSystem: Decimal; decimalLatitude: 10.8376; decimalLongitude: -85.6187; **Identification:** identifiedBy: AJ Fleming; dateIdentified: 2017; **Event:** samplingProtocol: Reared from the larva of the Crambidae, Syllepte belialis; verbatimEventDate: 17-May-2009; **Record Level:** language: en; institutionCode: CNC; collectionCode: Insects; basisOfRecord: Pinned Specimen
- **Type status:** Other material. **Occurrence:** occurrenceDetails: http://janzen.sas.upenn.edu; catalogNumber: DHJPAR0036449; recordedBy: D.H. Janzen, W. Hallwachs & Lucia Vargas; individualID: DHJPAR0036449; individualCount: 1; lifeStage: adult; preparations: pinned; otherCatalogNumbers: ASHYD1640-09, 08-SRNP-12916, BOLD:AAA8930; **Taxon:** scientificName: Hyphantrophaga manuelriosi; phylum: Arthropoda; class: Insecta; order: Diptera; family: Tachinidae; genus: Hyphantrophaga; specificEpithet: manuelriosi; scientificNameAuthorship: Fleming & Wood, 2018; **Location:** continent: Central America; country: Costa Rica; countryCode: CR; stateProvince: Guanacaste; county: Sector Santa Rosa; locality: Area de Conservacion Guanacaste; verbatimLocality: Area Administrativa; verbatimElevation: 295; verbatimLatitude: 10.8376; verbatimLongitude: -85.6187; verbatimCoordinateSystem: Decimal; decimalLatitude: 10.8376; decimalLongitude: -85.6187; **Identification:** identifiedBy: AJ Fleming; dateIdentified: 2017; **Event:** samplingProtocol: Reared from the larva of the Crambidae, Syllepte belialis; verbatimEventDate: 15-Apr-2009; **Record Level:** language: en; institutionCode: CNC; collectionCode: Insects; basisOfRecord: Pinned Specimen
- **Type status:** Other material. **Occurrence:** occurrenceDetails: http://janzen.sas.upenn.edu; catalogNumber: DHJPAR0036450; recordedBy: D.H. Janzen, W. Hallwachs & Lucia Vargas; individualID: DHJPAR0036450; individualCount: 1; lifeStage: adult; preparations: pinned; otherCatalogNumbers: ASHYD1641-09, 08-SRNP-12929, BOLD:AAA8930; **Taxon:** scientificName: Hyphantrophaga manuelriosi; phylum: Arthropoda; class: Insecta; order: Diptera; family: Tachinidae; genus: Hyphantrophaga; specificEpithet: manuelriosi; scientificNameAuthorship: Fleming & Wood, 2018; **Location:** continent: Central America; country: Costa Rica; countryCode: CR; stateProvince: Guanacaste; county: Sector Santa Rosa; locality: Area de Conservacion Guanacaste; verbatimLocality: Area Administrativa; verbatimElevation: 295; verbatimLatitude: 10.8376; verbatimLongitude: -85.6187; verbatimCoordinateSystem: Decimal; decimalLatitude: 10.8376; decimalLongitude: -85.6187; **Identification:** identifiedBy: AJ Fleming; dateIdentified: 2017; **Event:** samplingProtocol: Reared from the larva of the Crambidae, Syllepte belialis; verbatimEventDate: 21-Apr-2009; **Record Level:** language: en; institutionCode: CNC; collectionCode: Insects; basisOfRecord: Pinned Specimen
- **Type status:** Other material. **Occurrence:** occurrenceDetails: http://janzen.sas.upenn.edu; catalogNumber: DHJPAR0036452; recordedBy: D.H. Janzen, W. Hallwachs & Guillermo Pereira; individualID: DHJPAR0036452; individualCount: 1; lifeStage: adult; preparations: pinned; otherCatalogNumbers: ASHYD1643-09, 08-SRNP-13158, BOLD:AAA8930; **Taxon:** scientificName: Hyphantrophaga manuelriosi; phylum: Arthropoda; class: Insecta; order: Diptera; family: Tachinidae; genus: Hyphantrophaga; specificEpithet: manuelriosi; scientificNameAuthorship: Fleming & Wood, 2018; **Location:** continent: Central America; country: Costa Rica; countryCode: CR; stateProvince: Guanacaste; county: Sector Santa Rosa; locality: Area de Conservacion Guanacaste; verbatimLocality: Sendero Natural; verbatimElevation: 290; verbatimLatitude: 10.8357; verbatimLongitude: -85.6125; verbatimCoordinateSystem: Decimal; decimalLatitude: 10.8357; decimalLongitude: -85.6125; **Identification:** identifiedBy: AJ Fleming; dateIdentified: 2017; **Event:** samplingProtocol: Reared from the larva of the Crambidae, Syllepte belialis; verbatimEventDate: 24-Apr-2009; **Record Level:** language: en; institutionCode: CNC; collectionCode: Insects; basisOfRecord: Pinned Specimen
- **Type status:** Other material. **Occurrence:** occurrenceDetails: http://janzen.sas.upenn.edu; catalogNumber: DHJPAR0036453; recordedBy: D.H. Janzen, W. Hallwachs & Guillermo Pereira; individualID: DHJPAR0036453; individualCount: 1; lifeStage: adult; preparations: pinned; otherCatalogNumbers: ASHYD1644-09, 08-SRNP-13157, BOLD:AAA8930; **Taxon:** scientificName: Hyphantrophaga manuelriosi; phylum: Arthropoda; class: Insecta; order: Diptera; family: Tachinidae; genus: Hyphantrophaga; specificEpithet: manuelriosi; scientificNameAuthorship: Fleming & Wood, 2018; **Location:** continent: Central America; country: Costa Rica; countryCode: CR; stateProvince: Guanacaste; county: Sector Santa Rosa; locality: Area de Conservacion Guanacaste; verbatimLocality: Sendero Natural; verbatimElevation: 290; verbatimLatitude: 10.8357; verbatimLongitude: -85.6125; verbatimCoordinateSystem: Decimal; decimalLatitude: 10.8357; decimalLongitude: -85.6125; **Identification:** identifiedBy: AJ Fleming; dateIdentified: 2017; **Event:** samplingProtocol: Reared from the larva of the Crambidae, Syllepte belialis; verbatimEventDate: 24-Apr-2009; **Record Level:** language: en; institutionCode: CNC; collectionCode: Insects; basisOfRecord: Pinned Specimen

#### Description

**Male** (Fig. 19). Length: 5–8 mm. **Head** (Fig. 19b): vertex 1/3 of head width; two pairs of reclinate upper orbital setae; ocellar setae arising beside anterior ocellus; ocellar triangle silver (slight gold tinge present but overall concolorous with fronto-orbital plate); eye densely haired; parafacial bare; fronto-orbital plate sparsely setulose, hairs not extending below upper 1/3; fronto-orbital plate shiny silver; facial ridge bare; pedicel light brown-orange, lighter than black postpedicel; arista brown, very minutely pubescent, distinctly thickened on basal 1/3–1/4. **Thorax** (Fig. 19a, c): pale grey tomentose dorsally and laterally; four prominent dorsal vittae broken across suture, innermost pair not reaching 2nd postsutural dorsocentral seta; postpronotum with three setae arranged in a triangle; chaetotaxy: acrostichal setae 3:3; dorsocentral setae 3:4; intra-alar setae 2:3; supra-alar setae 2:3; three katepisternal setae; basal scutellar setae as long as scutellar setae; two pairs of lateral scutellar setae 2/3 as long as basal scutellar setae; subapical scutellar setae straight and divergent, 2X as thick as lateral scutellar setae; apical scutellar setae 1/4 length of basal scutellar setae crossed apically; one pair of discal scutellar setae set as widely apart as subapical scutellar setae; scutellum concolorous with scutum. **Legs** (Fig. 19c): yellow in ground colour, densely covered in black hairs making them appear almost black; fore femur with dense silver tomentum on posterodorsal surface; hind coxa setose. **Wing** (Fig. 19a, c): pale translucent, hyaline, not distinctly infuscate; vein R_4+5_ with only 2–3 setulae at base. **Abdomen** (Fig. 19a, c): ground colour black; mid-dorsal depression on ST1+2 almost reaching hind margin; entire abdomen covered in silver tomentum with brown tones towards tergal edges; median marginal setae present on ST1+2–T3; a complete row of marginal setae present on T4; discal setae only on T5; sex patch absent. **Terminalia**: sternite 5 (Fig. 19f) with a deeply excavated median cleft, slightly rounded V-shaped, margins covered in dense tomentum. Lateral lobes of sternite rounded apically, with 2–3 strong setae surrounded by many shorter, weaker setulae. Anterior plate of sternite 5 from subequal to slightly shorter than apical lobes, unsclerotised "window" weak, only very slightly unsclerotised, as wide as median cleft and flat. Cerci in posterior view (Fig. 19d) subrectangular, duck-billed and slightly longer than surstyli, blunt and rounded at apex, slightly divergent over apical 1/3; basal 2/3 fused; in lateral view rounded at tip, making them appear slightly downturned; densely setulose along basal 2/3, setulose ventrally along entire length. Surstylus in lateral view (Fig. 19e) almost parallel-sided along its entire length, ending in a slightly downcurved apex, making the structure appear slightly blade-like; surstylus appearing to be fused with epandrium; when viewed dorsally, surstyli appearing to point inwards and strongly convergent, laterally covered in short stout setulae. Pregonite short and stout, well-developed, 0.3 times as long as distiphallus, squared-off apically, with a few short marginal setulae. Postgonite slightly narrow, 1/3 as wide as pregonite, sharply pointed and curved at apex. Distiphallus rectangular, with a very slight apical flare, a slender median longitudinal sclerotised reinforcement on its posterior surface and a broad, anterolateral, sclerotised acrophallus, joined as a plate on anterior surface near apex.

**Female**. Length: 5–7 mm. As male, differing only by the presence of two pairs of proclinate orbital setae.

#### Diagnosis

*Hyphantrophaga manuelriosi* **sp. n.** can be distinguished from all other *Hyphantrophaga* species by the following combination of traits: ocellar triangle silver, fronto-orbital plate setulose throughout, pedicel light brown-orange, three katepisternal setae, hind coxa setose, median marginal setae present on ST1+2, discal setae absent from T3 and T4, sex patch absent, abdominal tomentum silver with brown tones at tergal edges.

#### Etymology

*Hyphantrophaga manuelriosi* **sp. n.** is named in recognition of Manuel Rios Castro's dedication and work in finding and rearing the ACG caterpillars that contained tachinid larvae.

#### Distribution

Costa Rica, ACG, Guanacaste Province, 10–480 m elevation.

#### Ecology

*Hyphantrophaga manuelriosi* **sp. n.** has been reared 58 times from one species of Lepidoptera in the family Crambidae, *Syllepte belialis* (Walker, 1859), in dry forest and dry-rain lowland intergrades.

### Hyphantrophaga morphophaga

Fleming & Wood sp. n.

urn:lsid:zoobank.org:act:F3E79C06-F806-4C34-A03B-58D7163280E8

#### Materials

- **Type status:** Holotype. **Occurrence:** occurrenceDetails: http://janzen.sas.upenn.edu; catalogNumber: DHJPAR0007354; recordedBy: D.H. Janzen, W. Hallwachs & gusaneros; individualID: DHJPAR0007354; individualCount: 1; sex: male; lifeStage: adult; preparations: pinned; otherCatalogNumbers: ASTAT126-06, 95-SRNP-4612, BOLD:AAA5134; **Taxon:** scientificName: Hyphantrophaga morphophaga; phylum: Arthropoda; class: Insecta; order: Diptera; family: Tachinidae; genus: Hyphantrophaga; specificEpithet: morphophaga; scientificNameAuthorship: Fleming & Wood, 2018; **Location:** continent: Central America; country: Costa Rica; countryCode: CR; stateProvince: Guanacaste; county: Sector Pailas; locality: Area de Conservacion Guanacaste; verbatimLocality: Gemelos; verbatimElevation: 1276; verbatimLatitude: 10.7693; verbatimLongitude: -85.3466; verbatimCoordinateSystem: Decimal; decimalLatitude: 10.7693; decimalLongitude: -85.3466; **Identification:** identifiedBy: AJ Fleming; dateIdentified: 2017; **Event:** samplingProtocol: Reared from the larva of the Nymphalidae, Morpho catalina; verbatimEventDate: 07-Jan-1995; **Record Level:** language: en; institutionCode: CNC; collectionCode: Insects; basisOfRecord: Pinned Specimen
- **Type status:** Paratype. **Occurrence:** occurrenceDetails: http://janzen.sas.upenn.edu; catalogNumber: DHJPAR0005457; recordedBy: D.H. Janzen, W. Hallwachs & Manuel Pereira; individualID: DHJPAR0005457; individualCount: 1; sex: male; lifeStage: adult; preparations: pinned; otherCatalogNumbers: ASTA577-06, 05-SRNP-47682,; **Taxon:** scientificName: Hyphantrophaga morphophaga; phylum: Arthropoda; class: Insecta; order: Diptera; family: Tachinidae; genus: Hyphantrophaga; specificEpithet: morphophaga; scientificNameAuthorship: Fleming & Wood, 2018; **Location:** continent: Central America; country: Costa Rica; countryCode: CR; stateProvince: Guanacaste; county: Sector Cacao; locality: Area de Conservacion Guanacaste; verbatimLocality: Estacion Gongora; verbatimElevation: 570; verbatimLatitude: 10.887; verbatimLongitude: -85.4744; verbatimCoordinateSystem: Decimal; decimalLatitude: 10.887; decimalLongitude: -85.4744; **Identification:** identifiedBy: AJ Fleming; dateIdentified: 2017; **Event:** samplingProtocol: Reared from the larva of the Nymphalidae, Morpho helenor; verbatimEventDate: 15-Nov-2005; **Record Level:** language: en; institutionCode: CNC; collectionCode: Insects; basisOfRecord: Pinned Specimen
- **Type status:** Paratype. **Occurrence:** occurrenceDetails: http://janzen.sas.upenn.edu; catalogNumber: DHJPAR0007350; recordedBy: D.H. Janzen, W. Hallwachs & Daniel H. Janzen; individualID: DHJPAR0007350; individualCount: 1; sex: male; lifeStage: adult; preparations: pinned; otherCatalogNumbers: ASTAT122-06, 84-SRNP-837, BOLD:AAA5134; **Taxon:** scientificName: Hyphantrophaga morphophaga; phylum: Arthropoda; class: Insecta; order: Diptera; family: Tachinidae; genus: Hyphantrophaga; specificEpithet: morphophaga; scientificNameAuthorship: Fleming & Wood, 2018; **Location:** continent: Central America; country: Costa Rica; countryCode: CR; stateProvince: Guanacaste; county: Sector Santa Rosa; locality: Area de Conservacion Guanacaste; verbatimLocality: Bosque San Emilio; verbatimElevation: 300; verbatimLatitude: 10.8439; verbatimLongitude: -85.6138; verbatimCoordinateSystem: Decimal; decimalLatitude: 10.8439; decimalLongitude: -85.6138; **Identification:** identifiedBy: AJ Fleming; dateIdentified: 2017; **Event:** samplingProtocol: Reared from the larva of the Nymphalidae, Morpho helenor; verbatimEventDate: Unavailable, 1984; **Record Level:** language: en; institutionCode: CNC; collectionCode: Insects; basisOfRecord: Pinned Specimen
- **Type status:** Paratype. **Occurrence:** occurrenceDetails: http://janzen.sas.upenn.edu; catalogNumber: DHJPAR0007351; recordedBy: D.H. Janzen, W. Hallwachs & Ruth Franco; individualID: DHJPAR0007351; individualCount: 1; sex: male; lifeStage: adult; preparations: pinned; otherCatalogNumbers: ASTAT123-06, 97-SRNP-11139, BOLD:AAA5134; **Taxon:** scientificName: Hyphantrophaga morphophaga; phylum: Arthropoda; class: Insecta; order: Diptera; family: Tachinidae; genus: Hyphantrophaga; specificEpithet: morphophaga; scientificNameAuthorship: Fleming & Wood, 2018; **Location:** continent: Central America; country: Costa Rica; countryCode: CR; stateProvince: Guanacaste; county: Sector Cacao; locality: Area de Conservacion Guanacaste; verbatimLocality: Estacion Cacao; verbatimElevation: 1150; verbatimLatitude: 10.9269; verbatimLongitude: -85.4682; verbatimCoordinateSystem: Decimal; decimalLatitude: 10.9269; decimalLongitude: -85.4682; **Identification:** identifiedBy: AJ Fleming; dateIdentified: 2017; **Event:** samplingProtocol: Reared from the larva of the Nymphalidae, Morpho helenor; verbatimEventDate: 27-Jan-1998; **Record Level:** language: en; institutionCode: CNC; collectionCode: Insects; basisOfRecord: Pinned Specimen
- **Type status:** Paratype. **Occurrence:** occurrenceDetails: http://janzen.sas.upenn.edu; catalogNumber: DHJPAR0007352; recordedBy: D.H. Janzen, W. Hallwachs & Harry Ramirez; individualID: DHJPAR0007352; individualCount: 1; sex: male; lifeStage: adult; preparations: pinned; otherCatalogNumbers: ASTAT124-06, 02-SRNP-23408, BOLD:AAA5134; **Taxon:** scientificName: Hyphantrophaga morphophaga; phylum: Arthropoda; class: Insecta; order: Diptera; family: Tachinidae; genus: Hyphantrophaga; specificEpithet: morphophaga; scientificNameAuthorship: Fleming & Wood, 2018; **Location:** continent: Central America; country: Costa Rica; countryCode: CR; stateProvince: Guanacaste; county: Sector Cacao; locality: Area de Conservacion Guanacaste; verbatimLocality: Sendero Arenales; verbatimElevation: 1080; verbatimLatitude: 10.9247; verbatimLongitude: -85.4674; verbatimCoordinateSystem: Decimal; decimalLatitude: 10.9247; decimalLongitude: -85.4674; **Identification:** identifiedBy: AJ Fleming; dateIdentified: 2017; **Event:** samplingProtocol: Reared from the larva of the Nymphalidae, Morpho helenor; verbatimEventDate: 08-Sep-2002; **Record Level:** language: en; institutionCode: CNC; collectionCode: Insects; basisOfRecord: Pinned Specimen
- **Type status:** Paratype. **Occurrence:** occurrenceDetails: http://janzen.sas.upenn.edu; catalogNumber: DHJPAR0007353; recordedBy: D.H. Janzen, W. Hallwachs & gusaneros; individualID: DHJPAR0007353; individualCount: 1; sex: male; lifeStage: adult; preparations: pinned; otherCatalogNumbers: ASTAT125-06, 95-SRNP-4613, BOLD:AAA5134; **Taxon:** scientificName: Hyphantrophaga morphophaga; phylum: Arthropoda; class: Insecta; order: Diptera; family: Tachinidae; genus: Hyphantrophaga; specificEpithet: morphophaga; scientificNameAuthorship: Fleming & Wood, 2018; **Location:** continent: Central America; country: Costa Rica; countryCode: CR; stateProvince: Guanacaste; county: Sector Pailas; locality: Area de Conservacion Guanacaste; verbatimLocality: Gemelos; verbatimElevation: 1276; verbatimLatitude: 10.7693; verbatimLongitude: -85.3466; verbatimCoordinateSystem: Decimal; decimalLatitude: 10.7693; decimalLongitude: -85.3466; **Identification:** identifiedBy: AJ Fleming; dateIdentified: 2017; **Event:** samplingProtocol: Reared from the larva of the Nymphalidae, Morpho catalina; verbatimEventDate: 07-Jan-1995; **Record Level:** language: en; institutionCode: CNC; collectionCode: Insects; basisOfRecord: Pinned Specimen
- **Type status:** Paratype. **Occurrence:** occurrenceDetails: http://janzen.sas.upenn.edu; catalogNumber: DHJPAR0007357; recordedBy: D.H. Janzen, W. Hallwachs & Mariano Pereira; individualID: DHJPAR0007357; individualCount: 1; sex: male; lifeStage: adult; preparations: pinned; otherCatalogNumbers: ASTAT129-06, 04-SRNP-35010, BOLD:AAA5134; **Taxon:** scientificName: Hyphantrophaga morphophaga; phylum: Arthropoda; class: Insecta; order: Diptera; family: Tachinidae; genus: Hyphantrophaga; specificEpithet: morphophaga; scientificNameAuthorship: Fleming & Wood, 2018; **Location:** continent: Central America; country: Costa Rica; countryCode: CR; stateProvince: Guanacaste; county: Sector Cacao; locality: Area de Conservacion Guanacaste; verbatimLocality: Sendero Abajo; verbatimElevation: 1020; verbatimLatitude: 10.9255; verbatimLongitude: -85.4716; verbatimCoordinateSystem: Decimal; decimalLatitude: 10.9255; decimalLongitude: -85.4716; **Identification:** identifiedBy: AJ Fleming; dateIdentified: 2017; **Event:** samplingProtocol: Reared from the larva of the Nymphalidae, Morpho catalina; verbatimEventDate: 25-Feb-2004; **Record Level:** language: en; institutionCode: CNC; collectionCode: Insects; basisOfRecord: Pinned Specimen
- **Type status:** Paratype. **Occurrence:** occurrenceDetails: http://janzen.sas.upenn.edu; catalogNumber: DHJPAR0007361; recordedBy: D.H. Janzen, W. Hallwachs & Elieth Cantillano; individualID: DHJPAR0007361; individualCount: 1; sex: male; lifeStage: adult; preparations: pinned; otherCatalogNumbers: ASTAT133-06, 05-SRNP-23929, BOLD:AAA5134; **Taxon:** scientificName: Hyphantrophaga morphophaga; phylum: Arthropoda; class: Insecta; order: Diptera; family: Tachinidae; genus: Hyphantrophaga; specificEpithet: morphophaga; scientificNameAuthorship: Fleming & Wood, 2018; **Location:** continent: Central America; country: Costa Rica; countryCode: CR; stateProvince: Guanacaste; county: Sector Del Oro; locality: Area de Conservacion Guanacaste; verbatimLocality: Quebrada Romero; verbatimElevation: 490; verbatimLatitude: 11.0052; verbatimLongitude: -85.474; verbatimCoordinateSystem: Decimal; decimalLatitude: 11.0052; decimalLongitude: -85.474; **Identification:** identifiedBy: AJ Fleming; dateIdentified: 2017; **Event:** samplingProtocol: Reared from the larva of the Nymphalidae, Morpho helenor; verbatimEventDate: 22-Oct-2005; **Record Level:** language: en; institutionCode: CNC; collectionCode: Insects; basisOfRecord: Pinned Specimen
- **Type status:** Paratype. **Occurrence:** occurrenceDetails: http://janzen.sas.upenn.edu; catalogNumber: DHJPAR0007364; recordedBy: D.H. Janzen, W. Hallwachs & Lucia Rios; individualID: DHJPAR0007364; individualCount: 1; sex: male; lifeStage: adult; preparations: pinned; otherCatalogNumbers: ASTAT136-06, 05-SRNP-24089, BOLD:AAA5134; **Taxon:** scientificName: Hyphantrophaga morphophaga; phylum: Arthropoda; class: Insecta; order: Diptera; family: Tachinidae; genus: Hyphantrophaga; specificEpithet: morphophaga; scientificNameAuthorship: Fleming & Wood, 2018; **Location:** continent: Central America; country: Costa Rica; countryCode: CR; stateProvince: Guanacaste; county: Sector Del Oro; locality: Area de Conservacion Guanacaste; verbatimLocality: Quebrada Trigal; verbatimElevation: 290; verbatimLatitude: 11.0268; verbatimLongitude: -85.4955; verbatimCoordinateSystem: Decimal; decimalLatitude: 11.0268; decimalLongitude: -85.4955; **Identification:** identifiedBy: AJ Fleming; dateIdentified: 2017; **Event:** samplingProtocol: Reared from the larva of the Nymphalidae, Morpho helenor; verbatimEventDate: 25-Oct-2005; **Record Level:** language: en; institutionCode: CNC; collectionCode: Insects; basisOfRecord: Pinned Specimen
- **Type status:** Paratype. **Occurrence:** occurrenceDetails: http://janzen.sas.upenn.edu; catalogNumber: DHJPAR0007366; recordedBy: D.H. Janzen, W. Hallwachs & Freddy Quesada; individualID: DHJPAR0007366; individualCount: 1; sex: male; lifeStage: adult; preparations: pinned; otherCatalogNumbers: ASTAT138-06, 02-SRNP-24447, BOLD:AAA5134; **Taxon:** scientificName: Hyphantrophaga morphophaga; phylum: Arthropoda; class: Insecta; order: Diptera; family: Tachinidae; genus: Hyphantrophaga; specificEpithet: morphophaga; scientificNameAuthorship: Fleming & Wood, 2018; **Location:** continent: Central America; country: Costa Rica; countryCode: CR; stateProvince: Guanacaste; county: Sector Cacao; locality: Area de Conservacion Guanacaste; verbatimLocality: Sendero Circular; verbatimElevation: 1185; verbatimLatitude: 10.9271; verbatimLongitude: -85.4668; verbatimCoordinateSystem: Decimal; decimalLatitude: 10.9271; decimalLongitude: -85.4668; **Identification:** identifiedBy: AJ Fleming; dateIdentified: 2017; **Event:** samplingProtocol: Reared from the larva of the Nymphalidae, Morpho helenor; verbatimEventDate: 29-Dec-2002; **Record Level:** language: en; institutionCode: CNC; collectionCode: Insects; basisOfRecord: Pinned Specimen
- **Type status:** Paratype. **Occurrence:** occurrenceDetails: http://janzen.sas.upenn.edu; catalogNumber: DHJPAR0007368; recordedBy: D.H. Janzen, W. Hallwachs & Manuel Pereira; individualID: DHJPAR0007368; individualCount: 1; sex: male; lifeStage: adult; preparations: pinned; otherCatalogNumbers: ASTAT140-06, 02-SRNP-29899, BOLD:AAA5134; **Taxon:** scientificName: Hyphantrophaga morphophaga; phylum: Arthropoda; class: Insecta; order: Diptera; family: Tachinidae; genus: Hyphantrophaga; specificEpithet: morphophaga; scientificNameAuthorship: Fleming & Wood, 2018; **Location:** continent: Central America; country: Costa Rica; countryCode: CR; stateProvince: Guanacaste; county: Sector Del Oro; locality: Area de Conservacion Guanacaste; verbatimLocality: Quebrada Raiz; verbatimElevation: 280; verbatimLatitude: 11.0287; verbatimLongitude: -85.4867; verbatimCoordinateSystem: Decimal; decimalLatitude: 11.0287; decimalLongitude: -85.4867; **Identification:** identifiedBy: AJ Fleming; dateIdentified: 2017; **Event:** samplingProtocol: Reared from the larva of the Nymphalidae, Morpho helenor; verbatimEventDate: 20-Oct-2002; **Record Level:** language: en; institutionCode: CNC; collectionCode: Insects; basisOfRecord: Pinned Specimen
- **Type status:** Paratype. **Occurrence:** occurrenceDetails: http://janzen.sas.upenn.edu; catalogNumber: DHJPAR0021984; recordedBy: D.H. Janzen, W. Hallwachs & Lucia Rios; individualID: DHJPAR0021984; individualCount: 1; sex: male; lifeStage: adult; preparations: pinned; otherCatalogNumbers: ASTAT1122-07, 07-SRNP-23642, BOLD:AAA5134; **Taxon:** scientificName: Hyphantrophaga morphophaga; phylum: Arthropoda; class: Insecta; order: Diptera; family: Tachinidae; genus: Hyphantrophaga; specificEpithet: morphophaga; scientificNameAuthorship: Fleming & Wood, 2018; **Location:** continent: Central America; country: Costa Rica; countryCode: CR; stateProvince: Guanacaste; county: Sector Del Oro; locality: Area de Conservacion Guanacaste; verbatimLocality: Quebrada Salazar; verbatimElevation: 560; verbatimLatitude: 11.0022; verbatimLongitude: -85.4634; verbatimCoordinateSystem: Decimal; decimalLatitude: 11.0022; decimalLongitude: -85.4634; **Identification:** identifiedBy: AJ Fleming; dateIdentified: 2017; **Event:** samplingProtocol: Reared from the larva of the Nymphalidae, Morpho helenor; verbatimEventDate: 02-Oct-2007; **Record Level:** language: en; institutionCode: CNC; collectionCode: Insects; basisOfRecord: Pinned Specimen
- **Type status:** Paratype. **Occurrence:** occurrenceDetails: http://janzen.sas.upenn.edu; catalogNumber: DHJPAR0006269; recordedBy: D.H. Janzen, W. Hallwachs & Lucia Rios; individualID: DHJPAR0006269; individualCount: 1; sex: female; lifeStage: adult; preparations: pinned; otherCatalogNumbers: ASTAI697-06, 04-SRNP-22553, BOLD:AAA5134; **Taxon:** scientificName: Hyphantrophaga morphophaga; phylum: Arthropoda; class: Insecta; order: Diptera; family: Tachinidae; genus: Hyphantrophaga; specificEpithet: morphophaga; scientificNameAuthorship: Fleming & Wood, 2018; **Location:** continent: Central America; country: Costa Rica; countryCode: CR; stateProvince: Guanacaste; county: Sector Del Oro; locality: Area de Conservacion Guanacaste; verbatimLocality: Quebrada Trigal; verbatimElevation: 290; verbatimLatitude: 11.0268; verbatimLongitude: -85.4955; verbatimCoordinateSystem: Decimal; decimalLatitude: 11.0268; decimalLongitude: -85.4955; **Identification:** identifiedBy: AJ Fleming; dateIdentified: 2017; **Event:** samplingProtocol: Reared from the larva of the Nymphalidae, Morpho helenor; verbatimEventDate: 02-Aug-2004; **Record Level:** language: en; institutionCode: CNC; collectionCode: Insects; basisOfRecord: Pinned Specimen
- **Type status:** Paratype. **Occurrence:** occurrenceDetails: http://janzen.sas.upenn.edu; catalogNumber: DHJPAR0059547; recordedBy: D.H. Janzen, W. Hallwachs & Lucia Rios; individualID: DHJPAR0059547; individualCount: 1; sex: female; lifeStage: adult; preparations: pinned; otherCatalogNumbers: ACGBA5964-16, 16-SRNP-20807, BOLD:AAA5134; **Taxon:** scientificName: Hyphantrophaga morphophaga; phylum: Arthropoda; class: Insecta; order: Diptera; family: Tachinidae; genus: Hyphantrophaga; specificEpithet: morphophaga; scientificNameAuthorship: Fleming & Wood, 2018; **Location:** continent: Central America; country: Costa Rica; countryCode: CR; stateProvince: Guanacaste; county: Sector Del Oro; locality: Area de Conservacion Guanacaste; verbatimLocality: Quebrada Raiz; verbatimElevation: 280; verbatimLatitude: 11.0287; verbatimLongitude: -85.4867; verbatimCoordinateSystem: Decimal; decimalLatitude: 11.0287; decimalLongitude: -85.4867; **Identification:** identifiedBy: AJ Fleming; dateIdentified: 2017; **Event:** samplingProtocol: Reared from the larva of the Nymphalidae, Morpho helenor; verbatimEventDate: 30-Jul-2016; **Record Level:** language: en; institutionCode: CNC; collectionCode: Insects; basisOfRecord: Pinned Specimen
- **Type status:** Paratype. **Occurrence:** occurrenceDetails: http://janzen.sas.upenn.edu; catalogNumber: DHJPAR0005521; recordedBy: D.H. Janzen, W. Hallwachs & Elieth Cantillano; individualID: DHJPAR0005521; individualCount: 1; sex: female; lifeStage: adult; preparations: pinned; otherCatalogNumbers: ASTA640-06, 05-SRNP-24423, BOLD:AAA5134; **Taxon:** scientificName: Hyphantrophaga morphophaga; phylum: Arthropoda; class: Insecta; order: Diptera; family: Tachinidae; genus: Hyphantrophaga; specificEpithet: morphophaga; scientificNameAuthorship: Fleming & Wood, 2018; **Location:** continent: Central America; country: Costa Rica; countryCode: CR; stateProvince: Guanacaste; county: Sector Del Oro; locality: Area de Conservacion Guanacaste; verbatimLocality: Quebrada Trigal; verbatimElevation: 290; verbatimLatitude: 11.0268; verbatimLongitude: -85.4955; verbatimCoordinateSystem: Decimal; decimalLatitude: 11.0268; decimalLongitude: -85.4955; **Identification:** identifiedBy: AJ Fleming; dateIdentified: 2017; **Event:** samplingProtocol: Reared from the larva of the Nymphalidae, Morpho helenor; verbatimEventDate: 17-Nov-2005; **Record Level:** language: en; institutionCode: CNC; collectionCode: Insects; basisOfRecord: Pinned Specimen
- **Type status:** Paratype. **Occurrence:** occurrenceDetails: http://janzen.sas.upenn.edu; catalogNumber: DHJPAR0007365; recordedBy: D.H. Janzen, W. Hallwachs & Manuel Pereira; individualID: DHJPAR0007365; individualCount: 1; sex: female; lifeStage: adult; preparations: pinned; otherCatalogNumbers: ASTAT137-06, 02-SRNP-30058, BOLD:AAA5134; **Taxon:** scientificName: Hyphantrophaga morphophaga; phylum: Arthropoda; class: Insecta; order: Diptera; family: Tachinidae; genus: Hyphantrophaga; specificEpithet: morphophaga; scientificNameAuthorship: Fleming & Wood, 2018; **Location:** continent: Central America; country: Costa Rica; countryCode: CR; stateProvince: Guanacaste; county: Sector El Hacha; locality: Area de Conservacion Guanacaste; verbatimLocality: Finca Araya; verbatimElevation: 295; verbatimLatitude: 11.0154; verbatimLongitude: -85.5113; verbatimCoordinateSystem: Decimal; decimalLatitude: 11.0154; decimalLongitude: -85.5113; **Identification:** identifiedBy: AJ Fleming; dateIdentified: 2017; **Event:** samplingProtocol: Reared from the larva of the Nymphalidae, Morpho helenor; verbatimEventDate: 14-Nov-2002; **Record Level:** language: en; institutionCode: CNC; collectionCode: Insects; basisOfRecord: Pinned Specimen
- **Type status:** Paratype. **Occurrence:** occurrenceDetails: http://janzen.sas.upenn.edu; catalogNumber: DHJPAR0011459; recordedBy: D.H. Janzen, W. Hallwachs & Guillermo Pereira; individualID: DHJPAR0011459; individualCount: 1; sex: female; lifeStage: adult; preparations: pinned; otherCatalogNumbers: ASTAQ846-06, 04-SRNP-14345,; **Taxon:** scientificName: Hyphantrophaga morphophaga; phylum: Arthropoda; class: Insecta; order: Diptera; family: Tachinidae; genus: Hyphantrophaga; specificEpithet: morphophaga; scientificNameAuthorship: Fleming & Wood, 2018; **Location:** continent: Central America; country: Costa Rica; countryCode: CR; stateProvince: Guanacaste; county: Sector Santa Elena; locality: Area de Conservacion Guanacaste; verbatimLocality: Casa Potrero Grande; verbatimElevation: 17; verbatimLatitude: 10.8492; verbatimLongitude: -85.7731; verbatimCoordinateSystem: Decimal; decimalLatitude: 10.8492; decimalLongitude: -85.7731; **Identification:** identifiedBy: AJ Fleming; dateIdentified: 2017; **Event:** samplingProtocol: Reared from the larva of the Nymphalidae, Morpho helenor; verbatimEventDate: 31-Oct-2004; **Record Level:** language: en; institutionCode: CNC; collectionCode: Insects; basisOfRecord: Pinned Specimen
- **Type status:** Paratype. **Occurrence:** occurrenceDetails: http://janzen.sas.upenn.edu; catalogNumber: DHJPAR0023229; recordedBy: D.H. Janzen, W. Hallwachs & Jose Cortez; individualID: DHJPAR0023229; individualCount: 1; sex: female; lifeStage: adult; preparations: pinned; otherCatalogNumbers: ASTAW390-08, 07-SRNP-60840, BOLD:AAA5134; **Taxon:** scientificName: Hyphantrophaga morphophaga; phylum: Arthropoda; class: Insecta; order: Diptera; family: Tachinidae; genus: Hyphantrophaga; specificEpithet: morphophaga; scientificNameAuthorship: Fleming & Wood, 2018; **Location:** continent: Central America; country: Costa Rica; countryCode: CR; stateProvince: Guanacaste; county: Sector Mundo Nuevo; locality: Area de Conservacion Guanacaste; verbatimLocality: Vado Miramonte; verbatimElevation: 305; verbatimLatitude: 10.7718; verbatimLongitude: -85.434; verbatimCoordinateSystem: Decimal; decimalLatitude: 10.7718; decimalLongitude: -85.434; **Identification:** identifiedBy: AJ Fleming; dateIdentified: 2017; **Event:** samplingProtocol: Reared from the larva of the Nymphalidae, Morpho helenor; verbatimEventDate: 27-Dec-2007; **Record Level:** language: en; institutionCode: CNC; collectionCode: Insects; basisOfRecord: Pinned Specimen
- **Type status:** Paratype. **Occurrence:** occurrenceDetails: http://janzen.sas.upenn.edu; catalogNumber: DHJPAR0006278; recordedBy: D.H. Janzen, W. Hallwachs & Manuel Pereira; individualID: DHJPAR0006278; individualCount: 1; sex: female; lifeStage: adult; preparations: pinned; otherCatalogNumbers: ASTAI706-06, 02-SRNP-29986, BOLD:AAA5134; **Taxon:** scientificName: Hyphantrophaga morphophaga; phylum: Arthropoda; class: Insecta; order: Diptera; family: Tachinidae; genus: Hyphantrophaga; specificEpithet: morphophaga; scientificNameAuthorship: Fleming & Wood, 2018; **Location:** continent: Central America; country: Costa Rica; countryCode: CR; stateProvince: Guanacaste; county: Sector El Hacha; locality: Area de Conservacion Guanacaste; verbatimLocality: Finca Araya; verbatimElevation: 295; verbatimLatitude: 11.0154; verbatimLongitude: -85.5113; verbatimCoordinateSystem: Decimal; decimalLatitude: 11.0154; decimalLongitude: -85.5113; **Identification:** identifiedBy: AJ Fleming; dateIdentified: 2017; **Event:** samplingProtocol: Reared from the larva of the Nymphalidae, Morpho helenor; verbatimEventDate: 24-Oct-2002; **Record Level:** language: en; institutionCode: CNC; collectionCode: Insects; basisOfRecord: Pinned Specimen
- **Type status:** Paratype. **Occurrence:** occurrenceDetails: http://janzen.sas.upenn.edu; catalogNumber: DHJPAR0006289; recordedBy: D.H. Janzen, W. Hallwachs & Harry Ramirez; individualID: DHJPAR0006289; individualCount: 1; sex: female; lifeStage: adult; preparations: pinned; otherCatalogNumbers: ASTAI717-06, 04-SRNP-35577, BOLD:AAA5134; **Taxon:** scientificName: Hyphantrophaga morphophaga; phylum: Arthropoda; class: Insecta; order: Diptera; family: Tachinidae; genus: Hyphantrophaga; specificEpithet: morphophaga; scientificNameAuthorship: Fleming & Wood, 2018; **Location:** continent: Central America; country: Costa Rica; countryCode: CR; stateProvince: Guanacaste; county: Sector Cacao; locality: Area de Conservacion Guanacaste; verbatimLocality: Sendero Segundo; verbatimElevation: 1180; verbatimLatitude: 10.9268; verbatimLongitude: -85.4533; verbatimCoordinateSystem: Decimal; decimalLatitude: 10.9268; decimalLongitude: -85.4533; **Identification:** identifiedBy: AJ Fleming; dateIdentified: 2017; **Event:** samplingProtocol: Reared from the larva of the Nymphalidae, Morpho helenor; verbatimEventDate: 25-Jul-2004; **Record Level:** language: en; institutionCode: CNC; collectionCode: Insects; basisOfRecord: Pinned Specimen
- **Type status:** Paratype. **Occurrence:** occurrenceDetails: http://janzen.sas.upenn.edu; catalogNumber: DHJPAR0005474; recordedBy: D.H. Janzen, W. Hallwachs & Manuel Pereira; individualID: DHJPAR0005474; individualCount: 1; sex: female; lifeStage: adult; preparations: pinned; otherCatalogNumbers: ASTA594-06, 05-SRNP-47688, BOLD:AAA5134; **Taxon:** scientificName: Hyphantrophaga morphophaga; phylum: Arthropoda; class: Insecta; order: Diptera; family: Tachinidae; genus: Hyphantrophaga; specificEpithet: morphophaga; scientificNameAuthorship: Fleming & Wood, 2018; **Location:** continent: Central America; country: Costa Rica; countryCode: CR; stateProvince: Guanacaste; county: Sector Cacao; locality: Area de Conservacion Guanacaste; verbatimLocality: Estacion Gongora; verbatimElevation: 570; verbatimLatitude: 10.887; verbatimLongitude: -85.4744; verbatimCoordinateSystem: Decimal; decimalLatitude: 10.887; decimalLongitude: -85.4744; **Identification:** identifiedBy: AJ Fleming; dateIdentified: 2017; **Event:** samplingProtocol: Reared from the larva of the Nymphalidae, Morpho helenor; verbatimEventDate: 02-Nov-2005; **Record Level:** language: en; institutionCode: CNC; collectionCode: Insects; basisOfRecord: Pinned Specimen
- **Type status:** Paratype. **Occurrence:** occurrenceDetails: http://janzen.sas.upenn.edu; catalogNumber: DHJPAR0007356; recordedBy: D.H. Janzen, W. Hallwachs & Harry Ramirez; individualID: DHJPAR0007356; individualCount: 1; sex: female; lifeStage: adult; preparations: pinned; otherCatalogNumbers: ASTAT128-06, 02-SRNP-8033, BOLD:AAA5134; **Taxon:** scientificName: Hyphantrophaga morphophaga; phylum: Arthropoda; class: Insecta; order: Diptera; family: Tachinidae; genus: Hyphantrophaga; specificEpithet: morphophaga; scientificNameAuthorship: Fleming & Wood, 2018; **Location:** continent: Central America; country: Costa Rica; countryCode: CR; stateProvince: Guanacaste; county: Sector Cacao; locality: Area de Conservacion Guanacaste; verbatimLocality: Sendero Arenales; verbatimElevation: 1080; verbatimLatitude: 10.9247; verbatimLongitude: -85.4674; verbatimCoordinateSystem: Decimal; decimalLatitude: 10.9247; decimalLongitude: -85.4674; **Identification:** identifiedBy: AJ Fleming; dateIdentified: 2017; **Event:** samplingProtocol: Reared from the larva of the Nymphalidae, Morpho catalina; verbatimEventDate: 22-Mar-2002; **Record Level:** language: en; institutionCode: CNC; collectionCode: Insects; basisOfRecord: Pinned Specimen
- **Type status:** Paratype. **Occurrence:** occurrenceDetails: http://janzen.sas.upenn.edu; catalogNumber: DHJPAR0007358; recordedBy: D.H. Janzen, W. Hallwachs & Elieth Cantillano; individualID: DHJPAR0007358; individualCount: 1; sex: female; lifeStage: adult; preparations: pinned; otherCatalogNumbers: ASTAT130-06, 05-SRNP-23929, BOLD:AAA5134; **Taxon:** scientificName: Hyphantrophaga morphophaga; phylum: Arthropoda; class: Insecta; order: Diptera; family: Tachinidae; genus: Hyphantrophaga; specificEpithet: morphophaga; scientificNameAuthorship: Fleming & Wood, 2018; **Location:** continent: Central America; country: Costa Rica; countryCode: CR; stateProvince: Guanacaste; county: Sector Del Oro; locality: Area de Conservacion Guanacaste; verbatimLocality: Quebrada Romero; verbatimElevation: 490; verbatimLatitude: 11.0052; verbatimLongitude: -85.474; verbatimCoordinateSystem: Decimal; decimalLatitude: 11.0052; decimalLongitude: -85.474; **Identification:** identifiedBy: AJ Fleming; dateIdentified: 2017; **Event:** samplingProtocol: Reared from the larva of the Nymphalidae, Morpho helenor; verbatimEventDate: 22-Oct-2005; **Record Level:** language: en; institutionCode: CNC; collectionCode: Insects; basisOfRecord: Pinned Specimen
- **Type status:** Paratype. **Occurrence:** occurrenceDetails: http://janzen.sas.upenn.edu; catalogNumber: DHJPAR0007359; recordedBy: D.H. Janzen, W. Hallwachs & Dunia Garcia; individualID: DHJPAR0007359; individualCount: 1; sex: female; lifeStage: adult; preparations: pinned; otherCatalogNumbers: ASTAT131-06, 03-SRNP-392, BOLD:AAA5134; **Taxon:** scientificName: Hyphantrophaga morphophaga; phylum: Arthropoda; class: Insecta; order: Diptera; family: Tachinidae; genus: Hyphantrophaga; specificEpithet: morphophaga; scientificNameAuthorship: Fleming & Wood, 2018; **Location:** continent: Central America; country: Costa Rica; countryCode: CR; stateProvince: Guanacaste; county: Sector Santa Elena; locality: Area de Conservacion Guanacaste; verbatimLocality: Quebrada Megalopyge; verbatimElevation: 20; verbatimLatitude: 10.8471; verbatimLongitude: -85.7714; verbatimCoordinateSystem: Decimal; decimalLatitude: 10.8471; decimalLongitude: -85.7714; **Identification:** identifiedBy: AJ Fleming; dateIdentified: 2017; **Event:** samplingProtocol: Reared from the larva of the Nymphalidae, Morpho helenor; verbatimEventDate: 13-Mar-2003; **Record Level:** language: en; institutionCode: CNC; collectionCode: Insects; basisOfRecord: Pinned Specimen
- **Type status:** Paratype. **Occurrence:** occurrenceDetails: http://janzen.sas.upenn.edu; catalogNumber: DHJPAR0007360; recordedBy: D.H. Janzen, W. Hallwachs & Ruth Franco; individualID: DHJPAR0007360; individualCount: 1; sex: female; lifeStage: adult; preparations: pinned; otherCatalogNumbers: ASTAT132-06, 02-SRNP-30652, BOLD:AAA5134; **Taxon:** scientificName: Hyphantrophaga morphophaga; phylum: Arthropoda; class: Insecta; order: Diptera; family: Tachinidae; genus: Hyphantrophaga; specificEpithet: morphophaga; scientificNameAuthorship: Fleming & Wood, 2018; **Location:** continent: Central America; country: Costa Rica; countryCode: CR; stateProvince: Guanacaste; county: Sector El Hacha; locality: Area de Conservacion Guanacaste; verbatimLocality: Finca Araya; verbatimElevation: 295; verbatimLatitude: 11.0154; verbatimLongitude: -85.5113; verbatimCoordinateSystem: Decimal; decimalLatitude: 11.0154; decimalLongitude: -85.5113; **Identification:** identifiedBy: AJ Fleming; dateIdentified: 2017; **Event:** samplingProtocol: Reared from the larva of the Nymphalidae, Morpho helenor; verbatimEventDate: 04-Nov-2002; **Record Level:** language: en; institutionCode: CNC; collectionCode: Insects; basisOfRecord: Pinned Specimen
- **Type status:** Paratype. **Occurrence:** occurrenceDetails: http://janzen.sas.upenn.edu; catalogNumber: DHJPAR0007362; recordedBy: D.H. Janzen, W. Hallwachs & Harry Ramirez; individualID: DHJPAR0007362; individualCount: 1; sex: female; lifeStage: adult; preparations: pinned; otherCatalogNumbers: ASTAT134-06, 05-SRNP-35404, BOLD:AAA5134; **Taxon:** scientificName: Hyphantrophaga morphophaga; phylum: Arthropoda; class: Insecta; order: Diptera; family: Tachinidae; genus: Hyphantrophaga; specificEpithet: morphophaga; scientificNameAuthorship: Fleming & Wood, 2018; **Location:** continent: Central America; country: Costa Rica; countryCode: CR; stateProvince: Guanacaste; county: Sector Cacao; locality: Area de Conservacion Guanacaste; verbatimLocality: Sendero Abajo; verbatimElevation: 1020; verbatimLatitude: 10.9255; verbatimLongitude: -85.4716; verbatimCoordinateSystem: Decimal; decimalLatitude: 10.9255; decimalLongitude: -85.4716; **Identification:** identifiedBy: AJ Fleming; dateIdentified: 2017; **Event:** samplingProtocol: Reared from the larva of the Nymphalidae, Morpho helenor; verbatimEventDate: 06-Aug-2005; **Record Level:** language: en; institutionCode: CNC; collectionCode: Insects; basisOfRecord: Pinned Specimen
- **Type status:** Paratype. **Occurrence:** occurrenceDetails: http://janzen.sas.upenn.edu; catalogNumber: DHJPAR0007363; recordedBy: D.H. Janzen, W. Hallwachs & Elieth Cantillano; individualID: DHJPAR0007363; individualCount: 1; sex: female; lifeStage: adult; preparations: pinned; otherCatalogNumbers: ASTAT135-06, 05-SRNP-23927, BOLD:AAA5134; **Taxon:** scientificName: Hyphantrophaga morphophaga; phylum: Arthropoda; class: Insecta; order: Diptera; family: Tachinidae; genus: Hyphantrophaga; specificEpithet: morphophaga; scientificNameAuthorship: Fleming & Wood, 2018; **Location:** continent: Central America; country: Costa Rica; countryCode: CR; stateProvince: Guanacaste; county: Sector Del Oro; locality: Area de Conservacion Guanacaste; verbatimLocality: Quebrada Romero; verbatimElevation: 490; verbatimLatitude: 11.0052; verbatimLongitude: -85.474; verbatimCoordinateSystem: Decimal; decimalLatitude: 11.0052; decimalLongitude: -85.474; **Identification:** identifiedBy: AJ Fleming; dateIdentified: 2017; **Event:** samplingProtocol: Reared from the larva of the Nymphalidae, Morpho helenor; verbatimEventDate: 27-Oct-2005; **Record Level:** language: en; institutionCode: CNC; collectionCode: Insects; basisOfRecord: Pinned Specimen
- **Type status:** Paratype. **Occurrence:** occurrenceDetails: http://janzen.sas.upenn.edu; catalogNumber: DHJPAR0007367; recordedBy: D.H. Janzen, W. Hallwachs & Lucia Rios; individualID: DHJPAR0007367; individualCount: 1; sex: female; lifeStage: adult; preparations: pinned; otherCatalogNumbers: ASTAT139-06, 02-SRNP-31434, BOLD:AAA5134; **Taxon:** scientificName: Hyphantrophaga morphophaga; phylum: Arthropoda; class: Insecta; order: Diptera; family: Tachinidae; genus: Hyphantrophaga; specificEpithet: morphophaga; scientificNameAuthorship: Fleming & Wood, 2018; **Location:** continent: Central America; country: Costa Rica; countryCode: CR; stateProvince: Guanacaste; county: Sector El Hacha; locality: Area de Conservacion Guanacaste; verbatimLocality: Finca Araya; verbatimElevation: 295; verbatimLatitude: 11.0154; verbatimLongitude: -85.5113; verbatimCoordinateSystem: Decimal; decimalLatitude: 11.0154; decimalLongitude: -85.5113; **Identification:** identifiedBy: AJ Fleming; dateIdentified: 2017; **Event:** samplingProtocol: Reared from the larva of the Nymphalidae, Morpho helenor; verbatimEventDate: 22-Nov-2002; **Record Level:** language: en; institutionCode: CNC; collectionCode: Insects; basisOfRecord: Pinned Specimen
- **Type status:** Other material. **Occurrence:** occurrenceDetails: http://janzen.sas.upenn.edu; catalogNumber: DHJPAR0029643; recordedBy: D.H. Janzen, W. Hallwachs & Elda Araya; individualID: DHJPAR0029643; individualCount: 1; sex: male; lifeStage: adult; preparations: pinned; otherCatalogNumbers: ASHYM1064-09, 08-SRNP-5341, BOLD:AAA5134; **Taxon:** scientificName: Hyphantrophaga morphophaga; phylum: Arthropoda; class: Insecta; order: Diptera; family: Tachinidae; genus: Hyphantrophaga; specificEpithet: morphophaga; scientificNameAuthorship: Fleming & Wood, 2018; **Location:** continent: Central America; country: Costa Rica; countryCode: CR; stateProvince: Alajuela; county: Sector San Cristobal; locality: Area de Conservacion Guanacaste; verbatimLocality: Quebrada Garcia; verbatimElevation: 495; verbatimLatitude: 10.8607; verbatimLongitude: -85.4256; verbatimCoordinateSystem: Decimal; decimalLatitude: 10.8607; decimalLongitude: -85.4256; **Identification:** identifiedBy: AJ Fleming; dateIdentified: 2017; **Event:** samplingProtocol: Reared from the larva of the Nymphalidae, Morpho helenor; verbatimEventDate: 20-Oct-2008; **Record Level:** language: en; institutionCode: CNC; collectionCode: Insects; basisOfRecord: Pinned Specimen
- **Type status:** Other material. **Occurrence:** occurrenceDetails: http://janzen.sas.upenn.edu; catalogNumber: DHJPAR0030122; recordedBy: D.H. Janzen, W. Hallwachs & Jose Cortez; individualID: DHJPAR0030122; individualCount: 1; sex: male; lifeStage: adult; preparations: pinned; otherCatalogNumbers: ASHYB866-09, 08-SRNP-58574, BOLD:AAA5134; **Taxon:** scientificName: Hyphantrophaga morphophaga; phylum: Arthropoda; class: Insecta; order: Diptera; family: Tachinidae; genus: Hyphantrophaga; specificEpithet: morphophaga; scientificNameAuthorship: Fleming & Wood, 2018; **Location:** continent: Central America; country: Costa Rica; countryCode: CR; stateProvince: Guanacaste; county: Sector Mundo Nuevo; locality: Area de Conservacion Guanacaste; verbatimLocality: Vado Zanja Tapada; verbatimElevation: 550; verbatimLatitude: 10.7648; verbatimLongitude: -85.3845; verbatimCoordinateSystem: Decimal; decimalLatitude: 10.7648; decimalLongitude: -85.3845; **Identification:** identifiedBy: AJ Fleming; dateIdentified: 2017; **Event:** samplingProtocol: Reared from the larva of the Nymphalidae, Morpho helenor; verbatimEventDate: 02-Jan-2009; **Record Level:** language: en; institutionCode: CNC; collectionCode: Insects; basisOfRecord: Pinned Specimen
- **Type status:** Other material. **Occurrence:** occurrenceDetails: http://janzen.sas.upenn.edu; catalogNumber: DHJPAR0050520; recordedBy: D.H. Janzen, W. Hallwachs & Lucia Rios; individualID: DHJPAR0050520; individualCount: 1; sex: male; lifeStage: adult; preparations: pinned; otherCatalogNumbers: ACGBA3112-13, 12-SRNP-21848, BOLD:AAA5134; **Taxon:** scientificName: Hyphantrophaga morphophaga; phylum: Arthropoda; class: Insecta; order: Diptera; family: Tachinidae; genus: Hyphantrophaga; specificEpithet: morphophaga; scientificNameAuthorship: Fleming & Wood, 2018; **Location:** continent: Central America; country: Costa Rica; countryCode: CR; stateProvince: Guanacaste; county: Sector Del Oro; locality: Area de Conservacion Guanacaste; verbatimLocality: Quebrada Trigal; verbatimElevation: 290; verbatimLatitude: 11.0268; verbatimLongitude: -85.4955; verbatimCoordinateSystem: Decimal; decimalLatitude: 11.0268; decimalLongitude: -85.4955; **Identification:** identifiedBy: AJ Fleming; dateIdentified: 2017; **Event:** samplingProtocol: Reared from the larva of the Nymphalidae, Morpho helenor; verbatimEventDate: 24-Dec-2012; **Record Level:** language: en; institutionCode: CNC; collectionCode: Insects; basisOfRecord: Pinned Specimen
- **Type status:** Other material. **Occurrence:** occurrenceDetails: http://janzen.sas.upenn.edu; catalogNumber: DHJPAR0007355; recordedBy: D.H. Janzen, W. Hallwachs & Harry Ramirez; individualID: DHJPAR0007355; individualCount: 1; sex: male; lifeStage: adult; preparations: pinned; otherCatalogNumbers: ASTAT127-06, 01-SRNP-7320,; **Taxon:** scientificName: Hyphantrophaga morphophaga; phylum: Arthropoda; class: Insecta; order: Diptera; family: Tachinidae; genus: Hyphantrophaga; specificEpithet: morphophaga; scientificNameAuthorship: Fleming & Wood, 2018; **Location:** continent: Central America; country: Costa Rica; countryCode: CR; stateProvince: Guanacaste; county: Sector Cacao; locality: Area de Conservacion Guanacaste; verbatimLocality: Sendero Abajo; verbatimElevation: 1020; verbatimLatitude: 10.9255; verbatimLongitude: -85.4716; verbatimCoordinateSystem: Decimal; decimalLatitude: 10.9255; decimalLongitude: -85.4716; **Identification:** identifiedBy: AJ Fleming; dateIdentified: 2017; **Event:** samplingProtocol: Reared from the larva of the Nymphalidae, Morpho catalina; verbatimEventDate: 13-Sep-2001; **Record Level:** language: en; institutionCode: CNC; collectionCode: Insects; basisOfRecord: Pinned Specimen
- **Type status:** Other material. **Occurrence:** occurrenceDetails: http://janzen.sas.upenn.edu; catalogNumber: DHJPAR0057239; recordedBy: D.H. Janzen, W. Hallwachs & Manuel Rios; individualID: DHJPAR0057239; individualCount: 1; sex: male; lifeStage: adult; preparations: pinned; otherCatalogNumbers: ACGBA5149-15, 14-SRNP-20990, BOLD:AAA5134; **Taxon:** scientificName: Hyphantrophaga morphophaga; phylum: Arthropoda; class: Insecta; order: Diptera; family: Tachinidae; genus: Hyphantrophaga; specificEpithet: morphophaga; scientificNameAuthorship: Fleming & Wood, 2018; **Location:** continent: Central America; country: Costa Rica; countryCode: CR; stateProvince: Guanacaste; county: Sector Del Oro; locality: Area de Conservacion Guanacaste; verbatimLocality: Finca Araya; verbatimElevation: 295; verbatimLatitude: 11.0154; verbatimLongitude: -85.5113; verbatimCoordinateSystem: Decimal; decimalLatitude: 11.0154; decimalLongitude: -85.5113; **Identification:** identifiedBy: AJ Fleming; dateIdentified: 2017; **Event:** samplingProtocol: Reared from the larva of the Nymphalidae, Morpho helenor; verbatimEventDate: 27-Oct-2014; **Record Level:** language: en; institutionCode: CNC; collectionCode: Insects; basisOfRecord: Pinned Specimen
- **Type status:** Other material. **Occurrence:** occurrenceDetails: http://janzen.sas.upenn.edu; catalogNumber: DHJPAR0007370; recordedBy: D.H. Janzen, W. Hallwachs & Freddy Quesada; individualID: DHJPAR0007370; individualCount: 1; sex: male; lifeStage: adult; preparations: pinned; otherCatalogNumbers: ASTAT142-06, 04-SRNP-14946, BOLD:AAA5134; **Taxon:** scientificName: Hyphantrophaga morphophaga; phylum: Arthropoda; class: Insecta; order: Diptera; family: Tachinidae; genus: Hyphantrophaga; specificEpithet: morphophaga; scientificNameAuthorship: Fleming & Wood, 2018; **Location:** continent: Central America; country: Costa Rica; countryCode: CR; stateProvince: Guanacaste; county: Sector Santa Elena; locality: Area de Conservacion Guanacaste; verbatimLocality: Casa Potrero Grande; verbatimElevation: 17; verbatimLatitude: 10.8492; verbatimLongitude: -85.7731; verbatimCoordinateSystem: Decimal; decimalLatitude: 10.8492; decimalLongitude: -85.7731; **Identification:** identifiedBy: AJ Fleming; dateIdentified: 2017; **Event:** samplingProtocol: Reared from the larva of the Nymphalidae, Morpho helenor; verbatimEventDate: 11-Nov-2004; **Record Level:** language: en; institutionCode: CNC; collectionCode: Insects; basisOfRecord: Pinned Specimen
- **Type status:** Other material. **Occurrence:** occurrenceDetails: http://janzen.sas.upenn.edu; catalogNumber: DHJPAR0007371; recordedBy: D.H. Janzen, W. Hallwachs & Manuel Pereira; individualID: DHJPAR0007371; individualCount: 1; sex: male; lifeStage: adult; preparations: pinned; otherCatalogNumbers: ASTAT143-06, 04-SRNP-49474, BOLD:AAA5134; **Taxon:** scientificName: Hyphantrophaga morphophaga; phylum: Arthropoda; class: Insecta; order: Diptera; family: Tachinidae; genus: Hyphantrophaga; specificEpithet: morphophaga; scientificNameAuthorship: Fleming & Wood, 2018; **Location:** continent: Central America; country: Costa Rica; countryCode: CR; stateProvince: Guanacaste; county: Sector Cacao; locality: Area de Conservacion Guanacaste; verbatimLocality: Quebrada Otilio; verbatimElevation: 550; verbatimLatitude: 10.89; verbatimLongitude: -85.4797; verbatimCoordinateSystem: Decimal; decimalLatitude: 10.89; decimalLongitude: -85.4797; **Identification:** identifiedBy: AJ Fleming; dateIdentified: 2017; **Event:** samplingProtocol: Reared from the larva of the Nymphalidae, Morpho helenor; verbatimEventDate: 23-Nov-2004; **Record Level:** language: en; institutionCode: CNC; collectionCode: Insects; basisOfRecord: Pinned Specimen
- **Type status:** Other material. **Occurrence:** occurrenceDetails: http://janzen.sas.upenn.edu; catalogNumber: DHJPAR0007373; recordedBy: D.H. Janzen, W. Hallwachs & Mariano Pereira; individualID: DHJPAR0007373; individualCount: 1; sex: male; lifeStage: adult; preparations: pinned; otherCatalogNumbers: ASTAT145-06, 04-SRNP-35959, BOLD:AAA5134; **Taxon:** scientificName: Hyphantrophaga morphophaga; phylum: Arthropoda; class: Insecta; order: Diptera; family: Tachinidae; genus: Hyphantrophaga; specificEpithet: morphophaga; scientificNameAuthorship: Fleming & Wood, 2018; **Location:** continent: Central America; country: Costa Rica; countryCode: CR; stateProvince: Guanacaste; county: Sector Cacao; locality: Area de Conservacion Guanacaste; verbatimLocality: Sendero Abajo; verbatimElevation: 1020; verbatimLatitude: 10.9255; verbatimLongitude: -85.4716; verbatimCoordinateSystem: Decimal; decimalLatitude: 10.9255; decimalLongitude: -85.4716; **Identification:** identifiedBy: AJ Fleming; dateIdentified: 2017; **Event:** samplingProtocol: Reared from the larva of the Nymphalidae, Morpho helenor; verbatimEventDate: 17-Dec-2004; **Record Level:** language: en; institutionCode: CNC; collectionCode: Insects; basisOfRecord: Pinned Specimen
- **Type status:** Other material. **Occurrence:** occurrenceDetails: http://janzen.sas.upenn.edu; catalogNumber: DHJPAR0007377; recordedBy: D.H. Janzen, W. Hallwachs & Freddy Quesada; individualID: DHJPAR0007377; individualCount: 1; sex: male; lifeStage: adult; preparations: pinned; otherCatalogNumbers: ASTAT149-06, 04-SRNP-14354, BOLD:AAA5134; **Taxon:** scientificName: Hyphantrophaga morphophaga; phylum: Arthropoda; class: Insecta; order: Diptera; family: Tachinidae; genus: Hyphantrophaga; specificEpithet: morphophaga; scientificNameAuthorship: Fleming & Wood, 2018; **Location:** continent: Central America; country: Costa Rica; countryCode: CR; stateProvince: Guanacaste; county: Sector Santa Elena; locality: Area de Conservacion Guanacaste; verbatimLocality: Casa Potrero Grande; verbatimElevation: 17; verbatimLatitude: 10.8492; verbatimLongitude: -85.7731; verbatimCoordinateSystem: Decimal; decimalLatitude: 10.8492; decimalLongitude: -85.7731; **Identification:** identifiedBy: AJ Fleming; dateIdentified: 2017; **Event:** samplingProtocol: Reared from the larva of the Nymphalidae, Morpho helenor; verbatimEventDate: 05-Oct-2004; **Record Level:** language: en; institutionCode: CNC; collectionCode: Insects; basisOfRecord: Pinned Specimen
- **Type status:** Other material. **Occurrence:** occurrenceDetails: http://janzen.sas.upenn.edu; catalogNumber: DHJPAR0007383; recordedBy: D.H. Janzen, W. Hallwachs & Ruth Franco; individualID: DHJPAR0007383; individualCount: 1; sex: male; lifeStage: adult; preparations: pinned; otherCatalogNumbers: ASTAT155-06, 03-SRNP-27782, BOLD:AAA5134; **Taxon:** scientificName: Hyphantrophaga morphophaga; phylum: Arthropoda; class: Insecta; order: Diptera; family: Tachinidae; genus: Hyphantrophaga; specificEpithet: morphophaga; scientificNameAuthorship: Fleming & Wood, 2018; **Location:** continent: Central America; country: Costa Rica; countryCode: CR; stateProvince: Guanacaste; county: Sector Santa Rosa; locality: Area de Conservacion Guanacaste; verbatimLocality: Bosque Humedo; verbatimElevation: 290; verbatimLatitude: 10.8514; verbatimLongitude: -85.608; verbatimCoordinateSystem: Decimal; decimalLatitude: 10.8514; decimalLongitude: -85.608; **Identification:** identifiedBy: AJ Fleming; dateIdentified: 2017; **Event:** samplingProtocol: Reared from the larva of the Nymphalidae, Morpho helenor; verbatimEventDate: 28-Dec-2003; **Record Level:** language: en; institutionCode: CNC; collectionCode: Insects; basisOfRecord: Pinned Specimen
- **Type status:** Other material. **Occurrence:** occurrenceDetails: http://janzen.sas.upenn.edu; catalogNumber: DHJPAR0007384; recordedBy: D.H. Janzen, W. Hallwachs & Dunia Garcia; individualID: DHJPAR0007384; individualCount: 1; sex: male; lifeStage: adult; preparations: pinned; otherCatalogNumbers: ASTAT156-06, 04-SRNP-35102, BOLD:AAA5134; **Taxon:** scientificName: Hyphantrophaga morphophaga; phylum: Arthropoda; class: Insecta; order: Diptera; family: Tachinidae; genus: Hyphantrophaga; specificEpithet: morphophaga; scientificNameAuthorship: Fleming & Wood, 2018; **Location:** continent: Central America; country: Costa Rica; countryCode: CR; stateProvince: Guanacaste; county: Sector Cacao; locality: Area de Conservacion Guanacaste; verbatimLocality: Sendero Segundo; verbatimElevation: 1180; verbatimLatitude: 10.9268; verbatimLongitude: -85.4533; verbatimCoordinateSystem: Decimal; decimalLatitude: 10.9268; decimalLongitude: -85.4533; **Identification:** identifiedBy: AJ Fleming; dateIdentified: 2017; **Event:** samplingProtocol: Reared from the larva of the Nymphalidae, Morpho helenor; verbatimEventDate: 06-Mar-2004; **Record Level:** language: en; institutionCode: CNC; collectionCode: Insects; basisOfRecord: Pinned Specimen
- **Type status:** Other material. **Occurrence:** occurrenceDetails: http://janzen.sas.upenn.edu; catalogNumber: DHJPAR0007385; recordedBy: D.H. Janzen, W. Hallwachs & Harry Ramirez; individualID: DHJPAR0007385; individualCount: 1; sex: male; lifeStage: adult; preparations: pinned; otherCatalogNumbers: ASTAT157-06, 03-SRNP-22589,; **Taxon:** scientificName: Hyphantrophaga morphophaga; phylum: Arthropoda; class: Insecta; order: Diptera; family: Tachinidae; genus: Hyphantrophaga; specificEpithet: morphophaga; scientificNameAuthorship: Fleming & Wood, 2018; **Location:** continent: Central America; country: Costa Rica; countryCode: CR; stateProvince: Guanacaste; county: Sector Cacao; locality: Area de Conservacion Guanacaste; verbatimLocality: Estacion Cacao; verbatimElevation: 1150; verbatimLatitude: 10.9269; verbatimLongitude: -85.4682; verbatimCoordinateSystem: Decimal; decimalLatitude: 10.9269; decimalLongitude: -85.4682; **Identification:** identifiedBy: AJ Fleming; dateIdentified: 2017; **Event:** samplingProtocol: Reared from the larva of the Nymphalidae, Morpho helenor; verbatimEventDate: 23-Nov-2003; **Record Level:** language: en; institutionCode: CNC; collectionCode: Insects; basisOfRecord: Pinned Specimen
- **Type status:** Other material. **Occurrence:** occurrenceDetails: http://janzen.sas.upenn.edu; catalogNumber: DHJPAR0007386; recordedBy: D.H. Janzen, W. Hallwachs & Freddy Quesada; individualID: DHJPAR0007386; individualCount: 1; sex: male; lifeStage: adult; preparations: pinned; otherCatalogNumbers: ASTAT158-06, 02-SRNP-24360, BOLD:AAA5134; **Taxon:** scientificName: Hyphantrophaga morphophaga; phylum: Arthropoda; class: Insecta; order: Diptera; family: Tachinidae; genus: Hyphantrophaga; specificEpithet: morphophaga; scientificNameAuthorship: Fleming & Wood, 2018; **Location:** continent: Central America; country: Costa Rica; countryCode: CR; stateProvince: Guanacaste; county: Sector Cacao; locality: Area de Conservacion Guanacaste; verbatimLocality: Cuesta Caimito; verbatimElevation: 640; verbatimLatitude: 10.8908; verbatimLongitude: -85.4719; verbatimCoordinateSystem: Decimal; decimalLatitude: 10.8908; decimalLongitude: -85.4719; **Identification:** identifiedBy: AJ Fleming; dateIdentified: 2017; **Event:** samplingProtocol: Reared from the larva of the Nymphalidae, Morpho helenor; verbatimEventDate: 10-Dec-2002; **Record Level:** language: en; institutionCode: CNC; collectionCode: Insects; basisOfRecord: Pinned Specimen
- **Type status:** Other material. **Occurrence:** occurrenceDetails: http://janzen.sas.upenn.edu; catalogNumber: DHJPAR0007389; recordedBy: D.H. Janzen, W. Hallwachs & Harry Ramirez; individualID: DHJPAR0007389; individualCount: 1; sex: male; lifeStage: adult; preparations: pinned; otherCatalogNumbers: ASTAT161-06, 03-SRNP-23510, BOLD:AAA5134; **Taxon:** scientificName: Hyphantrophaga morphophaga; phylum: Arthropoda; class: Insecta; order: Diptera; family: Tachinidae; genus: Hyphantrophaga; specificEpithet: morphophaga; scientificNameAuthorship: Fleming & Wood, 2018; **Location:** continent: Central America; country: Costa Rica; countryCode: CR; stateProvince: Guanacaste; county: Sector Cacao; locality: Area de Conservacion Guanacaste; verbatimLocality: Sendero Arenales; verbatimElevation: 1080; verbatimLatitude: 10.9247; verbatimLongitude: -85.4674; verbatimCoordinateSystem: Decimal; decimalLatitude: 10.9247; decimalLongitude: -85.4674; **Identification:** identifiedBy: AJ Fleming; dateIdentified: 2017; **Event:** samplingProtocol: Reared from the larva of the Nymphalidae, Morpho helenor; verbatimEventDate: 04-Dec-2003; **Record Level:** language: en; institutionCode: CNC; collectionCode: Insects; basisOfRecord: Pinned Specimen
- **Type status:** Other material. **Occurrence:** occurrenceDetails: http://janzen.sas.upenn.edu; catalogNumber: DHJPAR0007391; recordedBy: D.H. Janzen, W. Hallwachs & Dunia Garcia; individualID: DHJPAR0007391; individualCount: 1; sex: male; lifeStage: adult; preparations: pinned; otherCatalogNumbers: ASTAT163-06, 03-SRNP-19784, BOLD:AAA5134; **Taxon:** scientificName: Hyphantrophaga morphophaga; phylum: Arthropoda; class: Insecta; order: Diptera; family: Tachinidae; genus: Hyphantrophaga; specificEpithet: morphophaga; scientificNameAuthorship: Fleming & Wood, 2018; **Location:** continent: Central America; country: Costa Rica; countryCode: CR; stateProvince: Guanacaste; county: Sector Del Oro; locality: Area de Conservacion Guanacaste; verbatimLocality: Quebrada Trigal; verbatimElevation: 290; verbatimLatitude: 11.0268; verbatimLongitude: -85.4955; verbatimCoordinateSystem: Decimal; decimalLatitude: 11.0268; decimalLongitude: -85.4955; **Identification:** identifiedBy: AJ Fleming; dateIdentified: 2017; **Event:** samplingProtocol: Reared from the larva of the Nymphalidae, Morpho helenor; verbatimEventDate: 21-Oct-2003; **Record Level:** language: en; institutionCode: CNC; collectionCode: Insects; basisOfRecord: Pinned Specimen
- **Type status:** Other material. **Occurrence:** occurrenceDetails: http://janzen.sas.upenn.edu; catalogNumber: DHJPAR0007392; recordedBy: D.H. Janzen, W. Hallwachs & Mariano Pereira; individualID: DHJPAR0007392; individualCount: 1; sex: male; lifeStage: adult; preparations: pinned; otherCatalogNumbers: ASTAT164-06, 03-SRNP-3015, BOLD:AAA5134; **Taxon:** scientificName: Hyphantrophaga morphophaga; phylum: Arthropoda; class: Insecta; order: Diptera; family: Tachinidae; genus: Hyphantrophaga; specificEpithet: morphophaga; scientificNameAuthorship: Fleming & Wood, 2018; **Location:** continent: Central America; country: Costa Rica; countryCode: CR; stateProvince: Guanacaste; county: Sector Cacao; locality: Area de Conservacion Guanacaste; verbatimLocality: Sendero Ponderosa; verbatimElevation: 1060; verbatimLatitude: 10.9146; verbatimLongitude: -85.4626; verbatimCoordinateSystem: Decimal; decimalLatitude: 10.9146; decimalLongitude: -85.4626; **Identification:** identifiedBy: AJ Fleming; dateIdentified: 2017; **Event:** samplingProtocol: Reared from the larva of the Nymphalidae, Morpho helenor; verbatimEventDate: 01-Apr-2003; **Record Level:** language: en; institutionCode: CNC; collectionCode: Insects; basisOfRecord: Pinned Specimen
- **Type status:** Other material. **Occurrence:** occurrenceDetails: http://janzen.sas.upenn.edu; catalogNumber: DHJPAR0007395; recordedBy: D.H. Janzen, W. Hallwachs & Harry Ramirez; individualID: DHJPAR0007395; individualCount: 1; sex: male; lifeStage: adult; preparations: pinned; otherCatalogNumbers: ASTAT167-06, 01-SRNP-7320,; **Taxon:** scientificName: Hyphantrophaga morphophaga; phylum: Arthropoda; class: Insecta; order: Diptera; family: Tachinidae; genus: Hyphantrophaga; specificEpithet: morphophaga; scientificNameAuthorship: Fleming & Wood, 2018; **Location:** continent: Central America; country: Costa Rica; countryCode: CR; stateProvince: Guanacaste; county: Sector Cacao; locality: Area de Conservacion Guanacaste; verbatimLocality: Sendero Abajo; verbatimElevation: 1020; verbatimLatitude: 10.9255; verbatimLongitude: -85.4716; verbatimCoordinateSystem: Decimal; decimalLatitude: 10.9255; decimalLongitude: -85.4716; **Identification:** identifiedBy: AJ Fleming; dateIdentified: 2017; **Event:** samplingProtocol: Reared from the larva of the Nymphalidae, Morpho catalina; verbatimEventDate: 13-Sep-2001; **Record Level:** language: en; institutionCode: CNC; collectionCode: Insects; basisOfRecord: Pinned Specimen
- **Type status:** Other material. **Occurrence:** occurrenceDetails: http://janzen.sas.upenn.edu; catalogNumber: DHJPAR0007399; recordedBy: D.H. Janzen, W. Hallwachs & Mariano Pereira; individualID: DHJPAR0007399; individualCount: 1; sex: male; lifeStage: adult; preparations: pinned; otherCatalogNumbers: ASTAT171-06, 98-SRNP-15802, BOLD:AAA5134; **Taxon:** scientificName: Hyphantrophaga morphophaga; phylum: Arthropoda; class: Insecta; order: Diptera; family: Tachinidae; genus: Hyphantrophaga; specificEpithet: morphophaga; scientificNameAuthorship: Fleming & Wood, 2018; **Location:** continent: Central America; country: Costa Rica; countryCode: CR; stateProvince: Guanacaste; county: Sector Cacao; locality: Area de Conservacion Guanacaste; verbatimLocality: Sendero Circular; verbatimElevation: 1185; verbatimLatitude: 10.9271; verbatimLongitude: -85.4668; verbatimCoordinateSystem: Decimal; decimalLatitude: 10.9271; decimalLongitude: -85.4668; **Identification:** identifiedBy: AJ Fleming; dateIdentified: 2017; **Event:** samplingProtocol: Reared from the larva of the Nymphalidae, Morpho helenor; verbatimEventDate: 01-Jun-1999; **Record Level:** language: en; institutionCode: CNC; collectionCode: Insects; basisOfRecord: Pinned Specimen
- **Type status:** Other material. **Occurrence:** occurrenceDetails: http://janzen.sas.upenn.edu; catalogNumber: DHJPAR0007405; recordedBy: D.H. Janzen, W. Hallwachs & Mariano Pereira; individualID: DHJPAR0007405; individualCount: 1; sex: male; lifeStage: adult; preparations: pinned; otherCatalogNumbers: ASTAT177-06, 02-SRNP-23914, BOLD:AAA5134; **Taxon:** scientificName: Hyphantrophaga morphophaga; phylum: Arthropoda; class: Insecta; order: Diptera; family: Tachinidae; genus: Hyphantrophaga; specificEpithet: morphophaga; scientificNameAuthorship: Fleming & Wood, 2018; **Location:** continent: Central America; country: Costa Rica; countryCode: CR; stateProvince: Guanacaste; county: Sector Cacao; locality: Area de Conservacion Guanacaste; verbatimLocality: Sendero Arenales; verbatimElevation: 1080; verbatimLatitude: 10.9247; verbatimLongitude: -85.4674; verbatimCoordinateSystem: Decimal; decimalLatitude: 10.9247; decimalLongitude: -85.4674; **Identification:** identifiedBy: AJ Fleming; dateIdentified: 2017; **Event:** samplingProtocol: Reared from the larva of the Nymphalidae, Morpho helenor; verbatimEventDate: 09-Nov-2002; **Record Level:** language: en; institutionCode: CNC; collectionCode: Insects; basisOfRecord: Pinned Specimen
- **Type status:** Other material. **Occurrence:** occurrenceDetails: http://janzen.sas.upenn.edu; catalogNumber: DHJPAR0007406; recordedBy: D.H. Janzen, W. Hallwachs & Mariano Pereira; individualID: DHJPAR0007406; individualCount: 1; sex: male; lifeStage: adult; preparations: pinned; otherCatalogNumbers: ASTAT178-06, 02-SRNP-23915, BOLD:AAA5134; **Taxon:** scientificName: Hyphantrophaga morphophaga; phylum: Arthropoda; class: Insecta; order: Diptera; family: Tachinidae; genus: Hyphantrophaga; specificEpithet: morphophaga; scientificNameAuthorship: Fleming & Wood, 2018; **Location:** continent: Central America; country: Costa Rica; countryCode: CR; stateProvince: Guanacaste; county: Sector Cacao; locality: Area de Conservacion Guanacaste; verbatimLocality: Sendero Arenales; verbatimElevation: 1080; verbatimLatitude: 10.9247; verbatimLongitude: -85.4674; verbatimCoordinateSystem: Decimal; decimalLatitude: 10.9247; decimalLongitude: -85.4674; **Identification:** identifiedBy: AJ Fleming; dateIdentified: 2017; **Event:** samplingProtocol: Reared from the larva of the Nymphalidae, Morpho helenor; verbatimEventDate: 09-Nov-2002; **Record Level:** language: en; institutionCode: CNC; collectionCode: Insects; basisOfRecord: Pinned Specimen
- **Type status:** Other material. **Occurrence:** occurrenceDetails: http://janzen.sas.upenn.edu; catalogNumber: DHJPAR0007407; recordedBy: D.H. Janzen, W. Hallwachs & Freddy Quesada; individualID: DHJPAR0007407; individualCount: 1; sex: male; lifeStage: adult; preparations: pinned; otherCatalogNumbers: ASTAT179-06, 02-SRNP-24432, BOLD:AAA5134; **Taxon:** scientificName: Hyphantrophaga morphophaga; phylum: Arthropoda; class: Insecta; order: Diptera; family: Tachinidae; genus: Hyphantrophaga; specificEpithet: morphophaga; scientificNameAuthorship: Fleming & Wood, 2018; **Location:** continent: Central America; country: Costa Rica; countryCode: CR; stateProvince: Guanacaste; county: Sector Cacao; locality: Area de Conservacion Guanacaste; verbatimLocality: Sendero Nayo; verbatimElevation: 1090; verbatimLatitude: 10.9245; verbatimLongitude: -85.4695; verbatimCoordinateSystem: Decimal; decimalLatitude: 10.9245; decimalLongitude: -85.4695; **Identification:** identifiedBy: AJ Fleming; dateIdentified: 2017; **Event:** samplingProtocol: Reared from the larva of the Nymphalidae, Morpho helenor; verbatimEventDate: 19-Dec-2002; **Record Level:** language: en; institutionCode: CNC; collectionCode: Insects; basisOfRecord: Pinned Specimen
- **Type status:** Other material. **Occurrence:** occurrenceDetails: http://janzen.sas.upenn.edu; catalogNumber: DHJPAR0007408; recordedBy: D.H. Janzen, W. Hallwachs & Lucia Rios; individualID: DHJPAR0007408; individualCount: 1; sex: male; lifeStage: adult; preparations: pinned; otherCatalogNumbers: ASTAT180-06, 02-SRNP-29406, BOLD:AAA5134; **Taxon:** scientificName: Hyphantrophaga morphophaga; phylum: Arthropoda; class: Insecta; order: Diptera; family: Tachinidae; genus: Hyphantrophaga; specificEpithet: morphophaga; scientificNameAuthorship: Fleming & Wood, 2018; **Location:** continent: Central America; country: Costa Rica; countryCode: CR; stateProvince: Guanacaste; county: Sector Del Oro; locality: Area de Conservacion Guanacaste; verbatimLocality: Quebrada Raiz; verbatimElevation: 280; verbatimLatitude: 11.0287; verbatimLongitude: -85.4867; verbatimCoordinateSystem: Decimal; decimalLatitude: 11.0287; decimalLongitude: -85.4867; **Identification:** identifiedBy: AJ Fleming; dateIdentified: 2017; **Event:** samplingProtocol: Reared from the larva of the Nymphalidae, Morpho helenor; verbatimEventDate: 20-Oct-2002; **Record Level:** language: en; institutionCode: CNC; collectionCode: Insects; basisOfRecord: Pinned Specimen
- **Type status:** Other material. **Occurrence:** occurrenceDetails: http://janzen.sas.upenn.edu; catalogNumber: DHJPAR0007409; recordedBy: D.H. Janzen, W. Hallwachs & Bienvenida Chavarria; individualID: DHJPAR0007409; individualCount: 1; sex: male; lifeStage: adult; preparations: pinned; otherCatalogNumbers: ASTAT181-06, 99-SRNP-947, BOLD:AAA5134; **Taxon:** scientificName: Hyphantrophaga morphophaga; phylum: Arthropoda; class: Insecta; order: Diptera; family: Tachinidae; genus: Hyphantrophaga; specificEpithet: morphophaga; scientificNameAuthorship: Fleming & Wood, 2018; **Location:** continent: Central America; country: Costa Rica; countryCode: CR; stateProvince: Guanacaste; county: Sector Cacao; locality: Area de Conservacion Guanacaste; verbatimLocality: Gongora Bananal; verbatimElevation: 600; verbatimLatitude: 10.8892; verbatimLongitude: -85.4761; verbatimCoordinateSystem: Decimal; decimalLatitude: 10.8892; decimalLongitude: -85.4761; **Identification:** identifiedBy: AJ Fleming; dateIdentified: 2017; **Event:** samplingProtocol: Reared from the larva of the Nymphalidae, Morpho helenor; verbatimEventDate: 08-Feb-1999; **Record Level:** language: en; institutionCode: CNC; collectionCode: Insects; basisOfRecord: Pinned Specimen
- **Type status:** Other material. **Occurrence:** occurrenceDetails: http://janzen.sas.upenn.edu; catalogNumber: DHJPAR0007410; recordedBy: D.H. Janzen, W. Hallwachs & Mariano Pereira; individualID: DHJPAR0007410; individualCount: 1; sex: male; lifeStage: adult; preparations: pinned; otherCatalogNumbers: ASTAT182-06, 99-SRNP-863, BOLD:AAA5134; **Taxon:** scientificName: Hyphantrophaga morphophaga; phylum: Arthropoda; class: Insecta; order: Diptera; family: Tachinidae; genus: Hyphantrophaga; specificEpithet: morphophaga; scientificNameAuthorship: Fleming & Wood, 2018; **Location:** continent: Central America; country: Costa Rica; countryCode: CR; stateProvince: Guanacaste; county: Sector Cacao; locality: Area de Conservacion Guanacaste; verbatimLocality: Sendero Circular; verbatimElevation: 1185; verbatimLatitude: 10.9271; verbatimLongitude: -85.4668; verbatimCoordinateSystem: Decimal; decimalLatitude: 10.9271; decimalLongitude: -85.4668; **Identification:** identifiedBy: AJ Fleming; dateIdentified: 2017; **Event:** samplingProtocol: Reared from the larva of the Nymphalidae, Morpho helenor; verbatimEventDate: 08-Mar-1999; **Record Level:** language: en; institutionCode: CNC; collectionCode: Insects; basisOfRecord: Pinned Specimen
- **Type status:** Other material. **Occurrence:** occurrenceDetails: http://janzen.sas.upenn.edu; catalogNumber: DHJPAR0007411; recordedBy: D.H. Janzen, W. Hallwachs & gusaneros; individualID: DHJPAR0007411; individualCount: 1; sex: male; lifeStage: adult; preparations: pinned; otherCatalogNumbers: ASTAT183-06, 96-SRNP-11101, BOLD:AAA5134; **Taxon:** scientificName: Hyphantrophaga morphophaga; phylum: Arthropoda; class: Insecta; order: Diptera; family: Tachinidae; genus: Hyphantrophaga; specificEpithet: morphophaga; scientificNameAuthorship: Fleming & Wood, 2018; **Location:** continent: Central America; country: Costa Rica; countryCode: CR; stateProvince: Guanacaste; county: Sector Cacao; locality: Area de Conservacion Guanacaste; verbatimLocality: Estacion Gongora; verbatimElevation: 570; verbatimLatitude: 10.887; verbatimLongitude: -85.4744; verbatimCoordinateSystem: Decimal; decimalLatitude: 10.887; decimalLongitude: -85.4744; **Identification:** identifiedBy: AJ Fleming; dateIdentified: 2017; **Event:** samplingProtocol: Reared from the larva of the Nymphalidae, Morpho helenor; verbatimEventDate: 11-Nov-1996; **Record Level:** language: en; institutionCode: CNC; collectionCode: Insects; basisOfRecord: Pinned Specimen
- **Type status:** Other material. **Occurrence:** occurrenceDetails: http://janzen.sas.upenn.edu; catalogNumber: DHJPAR0011460; recordedBy: D.H. Janzen, W. Hallwachs & Calixto Moraga; individualID: DHJPAR0011460; individualCount: 1; sex: male; lifeStage: adult; preparations: pinned; otherCatalogNumbers: ASTAQ847-06, 04-SRNP-32851, BOLD:AAA5134; **Taxon:** scientificName: Hyphantrophaga morphophaga; phylum: Arthropoda; class: Insecta; order: Diptera; family: Tachinidae; genus: Hyphantrophaga; specificEpithet: morphophaga; scientificNameAuthorship: Fleming & Wood, 2018; **Location:** continent: Central America; country: Costa Rica; countryCode: CR; stateProvince: Guanacaste; county: Sector Pitilla; locality: Area de Conservacion Guanacaste; verbatimLocality: Sendero Evangelista; verbatimElevation: 660; verbatimLatitude: 10.9868; verbatimLongitude: -85.4208; verbatimCoordinateSystem: Decimal; decimalLatitude: 10.9868; decimalLongitude: -85.4208; **Identification:** identifiedBy: AJ Fleming; dateIdentified: 2017; **Event:** samplingProtocol: Reared from the larva of the Nymphalidae, Morpho amathonte; verbatimEventDate: 12-Aug-2004; **Record Level:** language: en; institutionCode: CNC; collectionCode: Insects; basisOfRecord: Pinned Specimen
- **Type status:** Other material. **Occurrence:** occurrenceDetails: http://janzen.sas.upenn.edu; catalogNumber: DHJPAR0011462; recordedBy: D.H. Janzen, W. Hallwachs & Dunia Garcia; individualID: DHJPAR0011462; individualCount: 1; sex: male; lifeStage: adult; preparations: pinned; otherCatalogNumbers: ASTAQ849-06, 04-SRNP-48895, BOLD:AAA5134; **Taxon:** scientificName: Hyphantrophaga morphophaga; phylum: Arthropoda; class: Insecta; order: Diptera; family: Tachinidae; genus: Hyphantrophaga; specificEpithet: morphophaga; scientificNameAuthorship: Fleming & Wood, 2018; **Location:** continent: Central America; country: Costa Rica; countryCode: CR; stateProvince: Guanacaste; county: Sector Cacao; locality: Area de Conservacion Guanacaste; verbatimLocality: Gongora Bananal; verbatimElevation: 600; verbatimLatitude: 10.8892; verbatimLongitude: -85.4761; verbatimCoordinateSystem: Decimal; decimalLatitude: 10.8892; decimalLongitude: -85.4761; **Identification:** identifiedBy: AJ Fleming; dateIdentified: 2017; **Event:** samplingProtocol: Reared from the larva of the Nymphalidae, Morpho helenor; verbatimEventDate: 22-Oct-2004; **Record Level:** language: en; institutionCode: CNC; collectionCode: Insects; basisOfRecord: Pinned Specimen
- **Type status:** Other material. **Occurrence:** occurrenceDetails: http://janzen.sas.upenn.edu; catalogNumber: DHJPAR0016681; recordedBy: D.H. Janzen, W. Hallwachs & Manuel Pereira; individualID: DHJPAR0016681; individualCount: 1; sex: male; lifeStage: adult; preparations: pinned; otherCatalogNumbers: ASTAP986-07, 06-SRNP-36610, BOLD:AAA5134; **Taxon:** scientificName: Hyphantrophaga morphophaga; phylum: Arthropoda; class: Insecta; order: Diptera; family: Tachinidae; genus: Hyphantrophaga; specificEpithet: morphophaga; scientificNameAuthorship: Fleming & Wood, 2018; **Location:** continent: Central America; country: Costa Rica; countryCode: CR; stateProvince: Guanacaste; county: Sector Cacao; locality: Area de Conservacion Guanacaste; verbatimLocality: Estacion Cacao; verbatimElevation: 1150; verbatimLatitude: 10.9269; verbatimLongitude: -85.4682; verbatimCoordinateSystem: Decimal; decimalLatitude: 10.9269; decimalLongitude: -85.4682; **Identification:** identifiedBy: AJ Fleming; dateIdentified: 2017; **Event:** samplingProtocol: Reared from the larva of the Nymphalidae, Morpho helenor; verbatimEventDate: 28-Nov-2006; **Record Level:** language: en; institutionCode: CNC; collectionCode: Insects; basisOfRecord: Pinned Specimen
- **Type status:** Other material. **Occurrence:** occurrenceDetails: http://janzen.sas.upenn.edu; catalogNumber: DHJPAR0016682; recordedBy: D.H. Janzen, W. Hallwachs & Manuel Pereira; individualID: DHJPAR0016682; individualCount: 1; sex: male; lifeStage: adult; preparations: pinned; otherCatalogNumbers: ASTAP987-07, 06-SRNP-36868, BOLD:AAA5134; **Taxon:** scientificName: Hyphantrophaga morphophaga; phylum: Arthropoda; class: Insecta; order: Diptera; family: Tachinidae; genus: Hyphantrophaga; specificEpithet: morphophaga; scientificNameAuthorship: Fleming & Wood, 2018; **Location:** continent: Central America; country: Costa Rica; countryCode: CR; stateProvince: Guanacaste; county: Sector Cacao; locality: Area de Conservacion Guanacaste; verbatimLocality: Sendero Arenales; verbatimElevation: 1080; verbatimLatitude: 10.9247; verbatimLongitude: -85.4674; verbatimCoordinateSystem: Decimal; decimalLatitude: 10.9247; decimalLongitude: -85.4674; **Identification:** identifiedBy: AJ Fleming; dateIdentified: 2017; **Event:** samplingProtocol: Reared from the larva of the Nymphalidae, Morpho helenor; verbatimEventDate: 27-Dec-2006; **Record Level:** language: en; institutionCode: CNC; collectionCode: Insects; basisOfRecord: Pinned Specimen
- **Type status:** Other material. **Occurrence:** occurrenceDetails: http://janzen.sas.upenn.edu; catalogNumber: DHJPAR0019956; recordedBy: D.H. Janzen, W. Hallwachs & Manuel Pereira; individualID: DHJPAR0019956; individualCount: 1; sex: male; lifeStage: adult; preparations: pinned; otherCatalogNumbers: ASTA1239-07, 07-SRNP-36054, BOLD:AAA5134; **Taxon:** scientificName: Hyphantrophaga morphophaga; phylum: Arthropoda; class: Insecta; order: Diptera; family: Tachinidae; genus: Hyphantrophaga; specificEpithet: morphophaga; scientificNameAuthorship: Fleming & Wood, 2018; **Location:** continent: Central America; country: Costa Rica; countryCode: CR; stateProvince: Guanacaste; county: Sector Cacao; locality: Area de Conservacion Guanacaste; verbatimLocality: Sendero Derrumbe; verbatimElevation: 1220; verbatimLatitude: 10.9292; verbatimLongitude: -85.4643; verbatimCoordinateSystem: Decimal; decimalLatitude: 10.9292; decimalLongitude: -85.4643; **Identification:** identifiedBy: AJ Fleming; dateIdentified: 2017; **Event:** samplingProtocol: Reared from the larva of the Nymphalidae, Morpho helenor; verbatimEventDate: 17-Aug-2007; **Record Level:** language: en; institutionCode: CNC; collectionCode: Insects; basisOfRecord: Pinned Specimen
- **Type status:** Other material. **Occurrence:** occurrenceDetails: http://janzen.sas.upenn.edu; catalogNumber: DHJPAR0007369; recordedBy: D.H. Janzen, W. Hallwachs & Manuel Pereira; individualID: DHJPAR0007369; individualCount: 1; sex: female; lifeStage: adult; preparations: pinned; otherCatalogNumbers: ASTAT141-06, 04-SRNP-35956, BOLD:AAA5134; **Taxon:** scientificName: Hyphantrophaga morphophaga; phylum: Arthropoda; class: Insecta; order: Diptera; family: Tachinidae; genus: Hyphantrophaga; specificEpithet: morphophaga; scientificNameAuthorship: Fleming & Wood, 2018; **Location:** continent: Central America; country: Costa Rica; countryCode: CR; stateProvince: Guanacaste; county: Sector Cacao; locality: Area de Conservacion Guanacaste; verbatimLocality: Sendero Abajo; verbatimElevation: 1020; verbatimLatitude: 10.9255; verbatimLongitude: -85.4716; verbatimCoordinateSystem: Decimal; decimalLatitude: 10.9255; decimalLongitude: -85.4716; **Identification:** identifiedBy: AJ Fleming; dateIdentified: 2017; **Event:** samplingProtocol: Reared from the larva of the Nymphalidae, Morpho helenor; verbatimEventDate: 23-Dec-2004; **Record Level:** language: en; institutionCode: CNC; collectionCode: Insects; basisOfRecord: Pinned Specimen
- **Type status:** Other material. **Occurrence:** occurrenceDetails: http://janzen.sas.upenn.edu; catalogNumber: DHJPAR0007372; recordedBy: D.H. Janzen, W. Hallwachs & Dunia Garcia; individualID: DHJPAR0007372; individualCount: 1; sex: female; lifeStage: adult; preparations: pinned; otherCatalogNumbers: ASTAT144-06, 04-SRNP-48895, BOLD:AAA5134; **Taxon:** scientificName: Hyphantrophaga morphophaga; phylum: Arthropoda; class: Insecta; order: Diptera; family: Tachinidae; genus: Hyphantrophaga; specificEpithet: morphophaga; scientificNameAuthorship: Fleming & Wood, 2018; **Location:** continent: Central America; country: Costa Rica; countryCode: CR; stateProvince: Guanacaste; county: Sector Cacao; locality: Area de Conservacion Guanacaste; verbatimLocality: Gongora Bananal; verbatimElevation: 600; verbatimLatitude: 10.8892; verbatimLongitude: -85.4761; verbatimCoordinateSystem: Decimal; decimalLatitude: 10.8892; decimalLongitude: -85.4761; **Identification:** identifiedBy: AJ Fleming; dateIdentified: 2017; **Event:** samplingProtocol: Reared from the larva of the Nymphalidae, Morpho helenor; verbatimEventDate: 22-Oct-2004; **Record Level:** language: en; institutionCode: CNC; collectionCode: Insects; basisOfRecord: Pinned Specimen
- **Type status:** Other material. **Occurrence:** occurrenceDetails: http://janzen.sas.upenn.edu; catalogNumber: DHJPAR0007375; recordedBy: D.H. Janzen, W. Hallwachs & Harry Ramirez; individualID: DHJPAR0007375; individualCount: 1; sex: female; lifeStage: adult; preparations: pinned; otherCatalogNumbers: ASTAT147-06, 05-SRNP-48498, BOLD:AAA5134; **Taxon:** scientificName: Hyphantrophaga morphophaga; phylum: Arthropoda; class: Insecta; order: Diptera; family: Tachinidae; genus: Hyphantrophaga; specificEpithet: morphophaga; scientificNameAuthorship: Fleming & Wood, 2018; **Location:** continent: Central America; country: Costa Rica; countryCode: CR; stateProvince: Guanacaste; county: Sector Cacao; locality: Area de Conservacion Guanacaste; verbatimLocality: Gongora Bananal; verbatimElevation: 600; verbatimLatitude: 10.8892; verbatimLongitude: -85.4761; verbatimCoordinateSystem: Decimal; decimalLatitude: 10.8892; decimalLongitude: -85.4761; **Identification:** identifiedBy: AJ Fleming; dateIdentified: 2017; **Event:** samplingProtocol: Reared from the larva of the Nymphalidae, Morpho helenor; verbatimEventDate: 30-Oct-2005; **Record Level:** language: en; institutionCode: CNC; collectionCode: Insects; basisOfRecord: Pinned Specimen
- **Type status:** Other material. **Occurrence:** occurrenceDetails: http://janzen.sas.upenn.edu; catalogNumber: DHJPAR0007376; recordedBy: D.H. Janzen, W. Hallwachs & Dunia Garcia; individualID: DHJPAR0007376; individualCount: 1; sex: female; lifeStage: adult; preparations: pinned; otherCatalogNumbers: ASTAT148-06, 05-SRNP-47741, BOLD:AAA5134; **Taxon:** scientificName: Hyphantrophaga morphophaga; phylum: Arthropoda; class: Insecta; order: Diptera; family: Tachinidae; genus: Hyphantrophaga; specificEpithet: morphophaga; scientificNameAuthorship: Fleming & Wood, 2018; **Location:** continent: Central America; country: Costa Rica; countryCode: CR; stateProvince: Guanacaste; county: Sector Cacao; locality: Area de Conservacion Guanacaste; verbatimLocality: Estacion Gongora; verbatimElevation: 570; verbatimLatitude: 10.887; verbatimLongitude: -85.4744; verbatimCoordinateSystem: Decimal; decimalLatitude: 10.887; decimalLongitude: -85.4744; **Identification:** identifiedBy: AJ Fleming; dateIdentified: 2017; **Event:** samplingProtocol: Reared from the larva of the Nymphalidae, Morpho helenor; verbatimEventDate: 28-Oct-2005; **Record Level:** language: en; institutionCode: CNC; collectionCode: Insects; basisOfRecord: Pinned Specimen
- **Type status:** Other material. **Occurrence:** occurrenceDetails: http://janzen.sas.upenn.edu; catalogNumber: DHJPAR0007378; recordedBy: D.H. Janzen, W. Hallwachs & Dunia Garcia; individualID: DHJPAR0007378; individualCount: 1; sex: female; lifeStage: adult; preparations: pinned; otherCatalogNumbers: ASTAT150-06, 04-SRNP-48894, BOLD:AAA5134; **Taxon:** scientificName: Hyphantrophaga morphophaga; phylum: Arthropoda; class: Insecta; order: Diptera; family: Tachinidae; genus: Hyphantrophaga; specificEpithet: morphophaga; scientificNameAuthorship: Fleming & Wood, 2018; **Location:** continent: Central America; country: Costa Rica; countryCode: CR; stateProvince: Guanacaste; county: Sector Cacao; locality: Area de Conservacion Guanacaste; verbatimLocality: Gongora Bananal; verbatimElevation: 600; verbatimLatitude: 10.8892; verbatimLongitude: -85.4761; verbatimCoordinateSystem: Decimal; decimalLatitude: 10.8892; decimalLongitude: -85.4761; **Identification:** identifiedBy: AJ Fleming; dateIdentified: 2017; **Event:** samplingProtocol: Reared from the larva of the Nymphalidae, Morpho helenor; verbatimEventDate: 19-Oct-2004; **Record Level:** language: en; institutionCode: CNC; collectionCode: Insects; basisOfRecord: Pinned Specimen
- **Type status:** Other material. **Occurrence:** occurrenceDetails: http://janzen.sas.upenn.edu; catalogNumber: DHJPAR0007379; recordedBy: D.H. Janzen, W. Hallwachs & Freddy Quesada; individualID: DHJPAR0007379; individualCount: 1; sex: female; lifeStage: adult; preparations: pinned; otherCatalogNumbers: ASTAT151-06, 02-SRNP-24360, BOLD:AAA5134; **Taxon:** scientificName: Hyphantrophaga morphophaga; phylum: Arthropoda; class: Insecta; order: Diptera; family: Tachinidae; genus: Hyphantrophaga; specificEpithet: morphophaga; scientificNameAuthorship: Fleming & Wood, 2018; **Location:** continent: Central America; country: Costa Rica; countryCode: CR; stateProvince: Guanacaste; county: Sector Cacao; locality: Area de Conservacion Guanacaste; verbatimLocality: Cuesta Caimito; verbatimElevation: 640; verbatimLatitude: 10.8908; verbatimLongitude: -85.4719; verbatimCoordinateSystem: Decimal; decimalLatitude: 10.8908; decimalLongitude: -85.4719; **Identification:** identifiedBy: AJ Fleming; dateIdentified: 2017; **Event:** samplingProtocol: Reared from the larva of the Nymphalidae, Morpho helenor; verbatimEventDate: 10-Dec-2002; **Record Level:** language: en; institutionCode: CNC; collectionCode: Insects; basisOfRecord: Pinned Specimen
- **Type status:** Other material. **Occurrence:** occurrenceDetails: http://janzen.sas.upenn.edu; catalogNumber: DHJPAR0007380; recordedBy: D.H. Janzen, W. Hallwachs & Dunia Garcia; individualID: DHJPAR0007380; individualCount: 1; sex: female; lifeStage: adult; preparations: pinned; otherCatalogNumbers: ASTAT152-06, 03-SRNP-23856, BOLD:AAA5134; **Taxon:** scientificName: Hyphantrophaga morphophaga; phylum: Arthropoda; class: Insecta; order: Diptera; family: Tachinidae; genus: Hyphantrophaga; specificEpithet: morphophaga; scientificNameAuthorship: Fleming & Wood, 2018; **Location:** continent: Central America; country: Costa Rica; countryCode: CR; stateProvince: Guanacaste; county: Sector Cacao; locality: Area de Conservacion Guanacaste; verbatimLocality: Sendero Arenales; verbatimElevation: 1080; verbatimLatitude: 10.9247; verbatimLongitude: -85.4674; verbatimCoordinateSystem: Decimal; decimalLatitude: 10.9247; decimalLongitude: -85.4674; **Identification:** identifiedBy: AJ Fleming; dateIdentified: 2017; **Event:** samplingProtocol: Reared from the larva of the Nymphalidae, Morpho helenor; verbatimEventDate: 24-Mar-2004; **Record Level:** language: en; institutionCode: CNC; collectionCode: Insects; basisOfRecord: Pinned Specimen
- **Type status:** Other material. **Occurrence:** occurrenceDetails: http://janzen.sas.upenn.edu; catalogNumber: DHJPAR0007381; recordedBy: D.H. Janzen, W. Hallwachs & Harry Ramirez; individualID: DHJPAR0007381; individualCount: 1; sex: female; lifeStage: adult; preparations: pinned; otherCatalogNumbers: ASTAT153-06, 02-SRNP-24376, BOLD:AAA5134; **Taxon:** scientificName: Hyphantrophaga morphophaga; phylum: Arthropoda; class: Insecta; order: Diptera; family: Tachinidae; genus: Hyphantrophaga; specificEpithet: morphophaga; scientificNameAuthorship: Fleming & Wood, 2018; **Location:** continent: Central America; country: Costa Rica; countryCode: CR; stateProvince: Guanacaste; county: Sector Cacao; locality: Area de Conservacion Guanacaste; verbatimLocality: Sendero Derrumbe; verbatimElevation: 1220; verbatimLatitude: 10.9292; verbatimLongitude: -85.4643; verbatimCoordinateSystem: Decimal; decimalLatitude: 10.9292; decimalLongitude: -85.4643; **Identification:** identifiedBy: AJ Fleming; dateIdentified: 2017; **Event:** samplingProtocol: Reared from the larva of the Nymphalidae, Morpho helenor; verbatimEventDate: 05-Dec-2002; **Record Level:** language: en; institutionCode: CNC; collectionCode: Insects; basisOfRecord: Pinned Specimen
- **Type status:** Other material. **Occurrence:** occurrenceDetails: http://janzen.sas.upenn.edu; catalogNumber: DHJPAR0007387; recordedBy: D.H. Janzen, W. Hallwachs & Roster Moraga; individualID: DHJPAR0007387; individualCount: 1; sex: female; lifeStage: adult; preparations: pinned; otherCatalogNumbers: ASTAT159-06, 97-SRNP-11061, BOLD:AAA5134; **Taxon:** scientificName: Hyphantrophaga morphophaga; phylum: Arthropoda; class: Insecta; order: Diptera; family: Tachinidae; genus: Hyphantrophaga; specificEpithet: morphophaga; scientificNameAuthorship: Fleming & Wood, 2018; **Location:** continent: Central America; country: Costa Rica; countryCode: CR; stateProvince: Guanacaste; county: Sector Cacao; locality: Area de Conservacion Guanacaste; verbatimLocality: Sendero Arenales; verbatimElevation: 1080; verbatimLatitude: 10.9247; verbatimLongitude: -85.4674; verbatimCoordinateSystem: Decimal; decimalLatitude: 10.9247; decimalLongitude: -85.4674; **Identification:** identifiedBy: AJ Fleming; dateIdentified: 2017; **Event:** samplingProtocol: Reared from the larva of the Nymphalidae, Morpho helenor; verbatimEventDate: 15-Jan-1998; **Record Level:** language: en; institutionCode: CNC; collectionCode: Insects; basisOfRecord: Pinned Specimen
- **Type status:** Other material. **Occurrence:** occurrenceDetails: http://janzen.sas.upenn.edu; catalogNumber: DHJPAR0007388; recordedBy: D.H. Janzen, W. Hallwachs & Harry Ramirez; individualID: DHJPAR0007388; individualCount: 1; sex: female; lifeStage: adult; preparations: pinned; otherCatalogNumbers: ASTAT160-06, 03-SRNP-23698, BOLD:AAA5134; **Taxon:** scientificName: Hyphantrophaga morphophaga; phylum: Arthropoda; class: Insecta; order: Diptera; family: Tachinidae; genus: Hyphantrophaga; specificEpithet: morphophaga; scientificNameAuthorship: Fleming & Wood, 2018; **Location:** continent: Central America; country: Costa Rica; countryCode: CR; stateProvince: Guanacaste; county: Sector Cacao; locality: Area de Conservacion Guanacaste; verbatimLocality: Sendero Arenales; verbatimElevation: 1080; verbatimLatitude: 10.9247; verbatimLongitude: -85.4674; verbatimCoordinateSystem: Decimal; decimalLatitude: 10.9247; decimalLongitude: -85.4674; **Identification:** identifiedBy: AJ Fleming; dateIdentified: 2017; **Event:** samplingProtocol: Reared from the larva of the Nymphalidae, Morpho helenor; verbatimEventDate: 18-Nov-2003; **Record Level:** language: en; institutionCode: CNC; collectionCode: Insects; basisOfRecord: Pinned Specimen
- **Type status:** Other material. **Occurrence:** occurrenceDetails: http://janzen.sas.upenn.edu; catalogNumber: DHJPAR0007390; recordedBy: D.H. Janzen, W. Hallwachs & Harry Ramirez; individualID: DHJPAR0007390; individualCount: 1; sex: female; lifeStage: adult; preparations: pinned; otherCatalogNumbers: ASTAT162-06, 03-SRNP-23364, BOLD:AAA5134; **Taxon:** scientificName: Hyphantrophaga morphophaga; phylum: Arthropoda; class: Insecta; order: Diptera; family: Tachinidae; genus: Hyphantrophaga; specificEpithet: morphophaga; scientificNameAuthorship: Fleming & Wood, 2018; **Location:** continent: Central America; country: Costa Rica; countryCode: CR; stateProvince: Guanacaste; county: Sector Cacao; locality: Area de Conservacion Guanacaste; verbatimLocality: Sendero Ponderosa; verbatimElevation: 1060; verbatimLatitude: 10.9146; verbatimLongitude: -85.4626; verbatimCoordinateSystem: Decimal; decimalLatitude: 10.9146; decimalLongitude: -85.4626; **Identification:** identifiedBy: AJ Fleming; dateIdentified: 2017; **Event:** samplingProtocol: Reared from the larva of the Nymphalidae, Morpho helenor; verbatimEventDate: 09-Dec-2003; **Record Level:** language: en; institutionCode: CNC; collectionCode: Insects; basisOfRecord: Pinned Specimen
- **Type status:** Other material. **Occurrence:** occurrenceDetails: http://janzen.sas.upenn.edu; catalogNumber: DHJPAR0007393; recordedBy: D.H. Janzen, W. Hallwachs & Freddy Quesada; individualID: DHJPAR0007393; individualCount: 1; sex: female; lifeStage: adult; preparations: pinned; otherCatalogNumbers: ASTAT165-06, 03-SRNP-3816, BOLD:AAA5134; **Taxon:** scientificName: Hyphantrophaga morphophaga; phylum: Arthropoda; class: Insecta; order: Diptera; family: Tachinidae; genus: Hyphantrophaga; specificEpithet: morphophaga; scientificNameAuthorship: Fleming & Wood, 2018; **Location:** continent: Central America; country: Costa Rica; countryCode: CR; stateProvince: Guanacaste; county: Sector Cacao; locality: Area de Conservacion Guanacaste; verbatimLocality: Sendero Nayo; verbatimElevation: 1090; verbatimLatitude: 10.9245; verbatimLongitude: -85.4695; verbatimCoordinateSystem: Decimal; decimalLatitude: 10.9245; decimalLongitude: -85.4695; **Identification:** identifiedBy: AJ Fleming; dateIdentified: 2017; **Event:** samplingProtocol: Reared from the larva of the Nymphalidae, Morpho helenor; verbatimEventDate: 20-May-2003; **Record Level:** language: en; institutionCode: CNC; collectionCode: Insects; basisOfRecord: Pinned Specimen
- **Type status:** Other material. **Occurrence:** occurrenceDetails: http://janzen.sas.upenn.edu; catalogNumber: DHJPAR0007394; recordedBy: D.H. Janzen, W. Hallwachs & Mariano Pereira; individualID: DHJPAR0007394; individualCount: 1; sex: female; lifeStage: adult; preparations: pinned; otherCatalogNumbers: ASTAT166-06, 01-SRNP-6970, BOLD:AAA5134; **Taxon:** scientificName: Hyphantrophaga morphophaga; phylum: Arthropoda; class: Insecta; order: Diptera; family: Tachinidae; genus: Hyphantrophaga; specificEpithet: morphophaga; scientificNameAuthorship: Fleming & Wood, 2018; **Location:** continent: Central America; country: Costa Rica; countryCode: CR; stateProvince: Guanacaste; county: Sector Cacao; locality: Area de Conservacion Guanacaste; verbatimLocality: Sendero Arenales; verbatimElevation: 1080; verbatimLatitude: 10.9247; verbatimLongitude: -85.4674; verbatimCoordinateSystem: Decimal; decimalLatitude: 10.9247; decimalLongitude: -85.4674; **Identification:** identifiedBy: AJ Fleming; dateIdentified: 2017; **Event:** samplingProtocol: Reared from the larva of the Nymphalidae, Morpho helenor; verbatimEventDate: 07-Aug-2001; **Record Level:** language: en; institutionCode: CNC; collectionCode: Insects; basisOfRecord: Pinned Specimen
- **Type status:** Other material. **Occurrence:** occurrenceDetails: http://janzen.sas.upenn.edu; catalogNumber: DHJPAR0007396; recordedBy: D.H. Janzen, W. Hallwachs & Harry Ramirez; individualID: DHJPAR0007396; individualCount: 1; sex: female; lifeStage: adult; preparations: pinned; otherCatalogNumbers: ASTAT168-06, 98-SRNP-15571, BOLD:AAA5134; **Taxon:** scientificName: Hyphantrophaga morphophaga; phylum: Arthropoda; class: Insecta; order: Diptera; family: Tachinidae; genus: Hyphantrophaga; specificEpithet: morphophaga; scientificNameAuthorship: Fleming & Wood, 2018; **Location:** continent: Central America; country: Costa Rica; countryCode: CR; stateProvince: Guanacaste; county: Sector Cacao; locality: Area de Conservacion Guanacaste; verbatimLocality: Sendero Salto; verbatimElevation: 1000; verbatimLatitude: 10.9302; verbatimLongitude: -85.4694; verbatimCoordinateSystem: Decimal; decimalLatitude: 10.9302; decimalLongitude: -85.4694; **Identification:** identifiedBy: AJ Fleming; dateIdentified: 2017; **Event:** samplingProtocol: Reared from the larva of the Nymphalidae, Morpho helenor; verbatimEventDate: 16-Nov-1998; **Record Level:** language: en; institutionCode: CNC; collectionCode: Insects; basisOfRecord: Pinned Specimen
- **Type status:** Other material. **Occurrence:** occurrenceDetails: http://janzen.sas.upenn.edu; catalogNumber: DHJPAR0007397; recordedBy: D.H. Janzen, W. Hallwachs & Mariano Pereira; individualID: DHJPAR0007397; individualCount: 1; sex: female; lifeStage: adult; preparations: pinned; otherCatalogNumbers: ASTAT169-06, 98-SRNP-15774, BOLD:AAA5134; **Taxon:** scientificName: Hyphantrophaga morphophaga; phylum: Arthropoda; class: Insecta; order: Diptera; family: Tachinidae; genus: Hyphantrophaga; specificEpithet: morphophaga; scientificNameAuthorship: Fleming & Wood, 2018; **Location:** continent: Central America; country: Costa Rica; countryCode: CR; stateProvince: Guanacaste; county: Sector Cacao; locality: Area de Conservacion Guanacaste; verbatimLocality: Sendero Toma Agua; verbatimElevation: 1140; verbatimLatitude: 10.9285; verbatimLongitude: -85.4668; verbatimCoordinateSystem: Decimal; decimalLatitude: 10.9285; decimalLongitude: -85.4668; **Identification:** identifiedBy: AJ Fleming; dateIdentified: 2017; **Event:** samplingProtocol: Reared from the larva of the Nymphalidae, Morpho helenor; verbatimEventDate: 15-Dec-1998; **Record Level:** language: en; institutionCode: CNC; collectionCode: Insects; basisOfRecord: Pinned Specimen
- **Type status:** Other material. **Occurrence:** occurrenceDetails: http://janzen.sas.upenn.edu; catalogNumber: DHJPAR0007398; recordedBy: D.H. Janzen, W. Hallwachs & Harry Ramirez; individualID: DHJPAR0007398; individualCount: 1; sex: female; lifeStage: adult; preparations: pinned; otherCatalogNumbers: ASTAT170-06, 98-SRNP-15738, BOLD:AAA5134; **Taxon:** scientificName: Hyphantrophaga morphophaga; phylum: Arthropoda; class: Insecta; order: Diptera; family: Tachinidae; genus: Hyphantrophaga; specificEpithet: morphophaga; scientificNameAuthorship: Fleming & Wood, 2018; **Location:** continent: Central America; country: Costa Rica; countryCode: CR; stateProvince: Guanacaste; county: Sector Cacao; locality: Area de Conservacion Guanacaste; verbatimLocality: Sendero Arenales; verbatimElevation: 1080; verbatimLatitude: 10.9247; verbatimLongitude: -85.4674; verbatimCoordinateSystem: Decimal; decimalLatitude: 10.9247; decimalLongitude: -85.4674; **Identification:** identifiedBy: AJ Fleming; dateIdentified: 2017; **Event:** samplingProtocol: Reared from the larva of the Nymphalidae, Morpho helenor; verbatimEventDate: 20-Jan-1999; **Record Level:** language: en; institutionCode: CNC; collectionCode: Insects; basisOfRecord: Pinned Specimen
- **Type status:** Other material. **Occurrence:** occurrenceDetails: http://janzen.sas.upenn.edu; catalogNumber: DHJPAR0007400; recordedBy: D.H. Janzen, W. Hallwachs & Freddy Quesada; individualID: DHJPAR0007400; individualCount: 1; sex: female; lifeStage: adult; preparations: pinned; otherCatalogNumbers: ASTAT172-06, 02-SRNP-23313,; **Taxon:** scientificName: Hyphantrophaga morphophaga; phylum: Arthropoda; class: Insecta; order: Diptera; family: Tachinidae; genus: Hyphantrophaga; specificEpithet: morphophaga; scientificNameAuthorship: Fleming & Wood, 2018; **Location:** continent: Central America; country: Costa Rica; countryCode: CR; stateProvince: Guanacaste; county: Sector Cacao; locality: Area de Conservacion Guanacaste; verbatimLocality: Sendero Derrumbe; verbatimElevation: 1220; verbatimLatitude: 10.9292; verbatimLongitude: -85.4643; verbatimCoordinateSystem: Decimal; decimalLatitude: 10.9292; decimalLongitude: -85.4643; **Identification:** identifiedBy: AJ Fleming; dateIdentified: 2017; **Event:** samplingProtocol: Reared from the larva of the Nymphalidae, Morpho helenor; verbatimEventDate: 20-Aug-2002; **Record Level:** language: en; institutionCode: CNC; collectionCode: Insects; basisOfRecord: Pinned Specimen
- **Type status:** Other material. **Occurrence:** occurrenceDetails: http://janzen.sas.upenn.edu; catalogNumber: DHJPAR0007401; recordedBy: D.H. Janzen, W. Hallwachs & Mariano Pereira; individualID: DHJPAR0007401; individualCount: 1; sex: female; lifeStage: adult; preparations: pinned; otherCatalogNumbers: ASTAT173-06, 98-SRNP-15851, BOLD:AAA5134; **Taxon:** scientificName: Hyphantrophaga morphophaga; phylum: Arthropoda; class: Insecta; order: Diptera; family: Tachinidae; genus: Hyphantrophaga; specificEpithet: morphophaga; scientificNameAuthorship: Fleming & Wood, 2018; **Location:** continent: Central America; country: Costa Rica; countryCode: CR; stateProvince: Guanacaste; county: Sector Cacao; locality: Area de Conservacion Guanacaste; verbatimLocality: Sendero Arenales; verbatimElevation: 1080; verbatimLatitude: 10.9247; verbatimLongitude: -85.4674; verbatimCoordinateSystem: Decimal; decimalLatitude: 10.9247; decimalLongitude: -85.4674; **Identification:** identifiedBy: AJ Fleming; dateIdentified: 2017; **Event:** samplingProtocol: Reared from the larva of the Nymphalidae, Morpho helenor; verbatimEventDate: 24-Feb-1999; **Record Level:** language: en; institutionCode: CNC; collectionCode: Insects; basisOfRecord: Pinned Specimen
- **Type status:** Other material. **Occurrence:** occurrenceDetails: http://janzen.sas.upenn.edu; catalogNumber: DHJPAR0007402; recordedBy: D.H. Janzen, W. Hallwachs & Mariano Pereira; individualID: DHJPAR0007402; individualCount: 1; sex: female; lifeStage: adult; preparations: pinned; otherCatalogNumbers: ASTAT174-06, 99-SRNP-568, BOLD:AAA5134; **Taxon:** scientificName: Hyphantrophaga morphophaga; phylum: Arthropoda; class: Insecta; order: Diptera; family: Tachinidae; genus: Hyphantrophaga; specificEpithet: morphophaga; scientificNameAuthorship: Fleming & Wood, 2018; **Location:** continent: Central America; country: Costa Rica; countryCode: CR; stateProvince: Guanacaste; county: Sector Cacao; locality: Area de Conservacion Guanacaste; verbatimLocality: Sendero Arenales; verbatimElevation: 1080; verbatimLatitude: 10.9247; verbatimLongitude: -85.4674; verbatimCoordinateSystem: Decimal; decimalLatitude: 10.9247; decimalLongitude: -85.4674; **Identification:** identifiedBy: AJ Fleming; dateIdentified: 2017; **Event:** samplingProtocol: Reared from the larva of the Nymphalidae, Morpho helenor; verbatimEventDate: 06-Feb-1999; **Record Level:** language: en; institutionCode: CNC; collectionCode: Insects; basisOfRecord: Pinned Specimen
- **Type status:** Other material. **Occurrence:** occurrenceDetails: http://janzen.sas.upenn.edu; catalogNumber: DHJPAR0007403; recordedBy: D.H. Janzen, W. Hallwachs & Lucia Rios; individualID: DHJPAR0007403; individualCount: 1; sex: female; lifeStage: adult; preparations: pinned; otherCatalogNumbers: ASTAT175-06, 02-SRNP-30889, BOLD:AAA5134; **Taxon:** scientificName: Hyphantrophaga morphophaga; phylum: Arthropoda; class: Insecta; order: Diptera; family: Tachinidae; genus: Hyphantrophaga; specificEpithet: morphophaga; scientificNameAuthorship: Fleming & Wood, 2018; **Location:** continent: Central America; country: Costa Rica; countryCode: CR; stateProvince: Guanacaste; county: Sector Del Oro; locality: Area de Conservacion Guanacaste; verbatimLocality: Puente Mena; verbatimElevation: 280; verbatimLatitude: 11.0456; verbatimLongitude: -85.4574; verbatimCoordinateSystem: Decimal; decimalLatitude: 11.0456; decimalLongitude: -85.4574; **Identification:** identifiedBy: AJ Fleming; dateIdentified: 2017; **Event:** samplingProtocol: Reared from the larva of the Nymphalidae, Morpho helenor; verbatimEventDate: 15-Nov-2002; **Record Level:** language: en; institutionCode: CNC; collectionCode: Insects; basisOfRecord: Pinned Specimen
- **Type status:** Other material. **Occurrence:** occurrenceDetails: http://janzen.sas.upenn.edu; catalogNumber: DHJPAR0007404; recordedBy: D.H. Janzen, W. Hallwachs & gusaneros; individualID: DHJPAR0007404; individualCount: 1; sex: female; lifeStage: adult; preparations: pinned; otherCatalogNumbers: ASTAT176-06, 96-SRNP-11367,; **Taxon:** scientificName: Hyphantrophaga morphophaga; phylum: Arthropoda; class: Insecta; order: Diptera; family: Tachinidae; genus: Hyphantrophaga; specificEpithet: morphophaga; scientificNameAuthorship: Fleming & Wood, 2018; **Location:** continent: Central America; country: Costa Rica; countryCode: CR; stateProvince: Guanacaste; county: Sector Santa Rosa; locality: Area de Conservacion Guanacaste; verbatimLocality: Bosque San Emilio; verbatimElevation: 300; verbatimLatitude: 10.8439; verbatimLongitude: -85.6138; verbatimCoordinateSystem: Decimal; decimalLatitude: 10.8439; decimalLongitude: -85.6138; **Identification:** identifiedBy: AJ Fleming; dateIdentified: 2017; **Event:** samplingProtocol: Reared from the larva of the Nymphalidae, Morpho helenor; verbatimEventDate: 11-May-1996; **Record Level:** language: en; institutionCode: CNC; collectionCode: Insects; basisOfRecord: Pinned Specimen
- **Type status:** Other material. **Occurrence:** occurrenceDetails: http://janzen.sas.upenn.edu; catalogNumber: DHJPAR0019953; recordedBy: D.H. Janzen, W. Hallwachs & Jose Cortez; individualID: DHJPAR0019953; individualCount: 1; sex: female; lifeStage: adult; preparations: pinned; otherCatalogNumbers: ASTA1236-07, 07-SRNP-58222, BOLD:AAA5134; **Taxon:** scientificName: Hyphantrophaga morphophaga; phylum: Arthropoda; class: Insecta; order: Diptera; family: Tachinidae; genus: Hyphantrophaga; specificEpithet: morphophaga; scientificNameAuthorship: Fleming & Wood, 2018; **Location:** continent: Central America; country: Costa Rica; countryCode: CR; stateProvince: Guanacaste; county: Sector Mundo Nuevo; locality: Area de Conservacion Guanacaste; verbatimLocality: Sendero Mora; verbatimElevation: 480; verbatimLatitude: 10.7683; verbatimLongitude: -85.4257; verbatimCoordinateSystem: Decimal; decimalLatitude: 10.7683; decimalLongitude: -85.4257; **Identification:** identifiedBy: AJ Fleming; dateIdentified: 2017; **Event:** samplingProtocol: Reared from the larva of the Nymphalidae, Morpho helenor; verbatimEventDate: 17-Aug-2007; **Record Level:** language: en; institutionCode: CNC; collectionCode: Insects; basisOfRecord: Pinned Specimen
- **Type status:** Other material. **Occurrence:** occurrenceDetails: http://janzen.sas.upenn.edu; catalogNumber: DHJPAR0020952; recordedBy: D.H. Janzen, W. Hallwachs & Jose Alberto Sanchez; individualID: DHJPAR0020952; individualCount: 1; sex: female; lifeStage: adult; preparations: pinned; otherCatalogNumbers: ASTA1295-07, 07-SRNP-57297, BOLD:AAA5134; **Taxon:** scientificName: Hyphantrophaga morphophaga; phylum: Arthropoda; class: Insecta; order: Diptera; family: Tachinidae; genus: Hyphantrophaga; specificEpithet: morphophaga; scientificNameAuthorship: Fleming & Wood, 2018; **Location:** continent: Central America; country: Costa Rica; countryCode: CR; stateProvince: Guanacaste; county: Sector Mundo Nuevo; locality: Area de Conservacion Guanacaste; verbatimLocality: Quebrada Tibio Perla; verbatimElevation: 330; verbatimLatitude: 10.7626; verbatimLongitude: -85.4298; verbatimCoordinateSystem: Decimal; decimalLatitude: 10.7626; decimalLongitude: -85.4298; **Identification:** identifiedBy: AJ Fleming; dateIdentified: 2017; **Event:** samplingProtocol: Reared from the larva of the Nymphalidae, Morpho helenor; verbatimEventDate: 29-Jul-2007; **Record Level:** language: en; institutionCode: CNC; collectionCode: Insects; basisOfRecord: Pinned Specimen
- **Type status:** Other material. **Occurrence:** occurrenceDetails: http://janzen.sas.upenn.edu; catalogNumber: DHJPAR0007374; recordedBy: D.H. Janzen, W. Hallwachs & Mariano Pereira; individualID: DHJPAR0007374; individualCount: 1; lifeStage: adult; preparations: pinned; otherCatalogNumbers: ASTAT146-06, 04-SRNP-35960, BOLD:AAA5134; **Taxon:** scientificName: Hyphantrophaga morphophaga; phylum: Arthropoda; class: Insecta; order: Diptera; family: Tachinidae; genus: Hyphantrophaga; specificEpithet: morphophaga; scientificNameAuthorship: Fleming & Wood, 2018; **Location:** continent: Central America; country: Costa Rica; countryCode: CR; stateProvince: Guanacaste; county: Sector Cacao; locality: Area de Conservacion Guanacaste; verbatimLocality: Sendero Abajo; verbatimElevation: 1020; verbatimLatitude: 10.9255; verbatimLongitude: -85.4716; verbatimCoordinateSystem: Decimal; decimalLatitude: 10.9255; decimalLongitude: -85.4716; **Identification:** identifiedBy: AJ Fleming; dateIdentified: 2017; **Event:** samplingProtocol: Reared from the larva of the Nymphalidae, Morpho helenor; verbatimEventDate: 17-Dec-2004; **Record Level:** language: en; institutionCode: CNC; collectionCode: Insects; basisOfRecord: Pinned Specimen
- **Type status:** Other material. **Occurrence:** occurrenceDetails: http://janzen.sas.upenn.edu; catalogNumber: DHJPAR0021975; recordedBy: D.H. Janzen, W. Hallwachs & Dunia Garcia; individualID: DHJPAR0021975; individualCount: 1; lifeStage: adult; preparations: pinned; otherCatalogNumbers: ASTAT1113-07, 07-SRNP-46175, BOLD:AAA5134; **Taxon:** scientificName: Hyphantrophaga morphophaga; phylum: Arthropoda; class: Insecta; order: Diptera; family: Tachinidae; genus: Hyphantrophaga; specificEpithet: morphophaga; scientificNameAuthorship: Fleming & Wood, 2018; **Location:** continent: Central America; country: Costa Rica; countryCode: CR; stateProvince: Guanacaste; county: Sector Cacao; locality: Area de Conservacion Guanacaste; verbatimLocality: Quebrada Otilio; verbatimElevation: 550; verbatimLatitude: 10.89; verbatimLongitude: -85.4797; verbatimCoordinateSystem: Decimal; decimalLatitude: 10.89; decimalLongitude: -85.4797; **Identification:** identifiedBy: AJ Fleming; dateIdentified: 2017; **Event:** samplingProtocol: Reared from the larva of the Nymphalidae, Morpho helenor; verbatimEventDate: 09-Oct-2007; **Record Level:** language: en; institutionCode: CNC; collectionCode: Insects; basisOfRecord: Pinned Specimen
- **Type status:** Other material. **Occurrence:** occurrenceDetails: http://janzen.sas.upenn.edu; catalogNumber: DHJPAR0022286; recordedBy: D.H. Janzen, W. Hallwachs & Jose Cortez; individualID: DHJPAR0022286; individualCount: 1; lifeStage: adult; preparations: pinned; otherCatalogNumbers: ASMGI562-08, 07-SRNP-58222,; **Taxon:** scientificName: Hyphantrophaga morphophaga; phylum: Arthropoda; class: Insecta; order: Diptera; family: Tachinidae; genus: Hyphantrophaga; specificEpithet: morphophaga; scientificNameAuthorship: Fleming & Wood, 2018; **Location:** continent: Central America; country: Costa Rica; countryCode: CR; stateProvince: Guanacaste; county: Sector Mundo Nuevo; locality: Area de Conservacion Guanacaste; verbatimLocality: Sendero Mora; verbatimElevation: 480; verbatimLatitude: 10.7683; verbatimLongitude: -85.4257; verbatimCoordinateSystem: Decimal; decimalLatitude: 10.7683; decimalLongitude: -85.4257; **Identification:** identifiedBy: AJ Fleming; dateIdentified: 2017; **Event:** samplingProtocol: Reared from the larva of the Nymphalidae, Morpho helenor; verbatimEventDate: 17-Aug-2007; **Record Level:** language: en; institutionCode: CNC; collectionCode: Insects; basisOfRecord: Pinned Specimen
- **Type status:** Other material. **Occurrence:** occurrenceDetails: http://janzen.sas.upenn.edu; catalogNumber: DHJPAR0027845; recordedBy: D.H. Janzen, W. Hallwachs & Harry Ramirez; individualID: DHJPAR0027845; individualCount: 1; lifeStage: adult; preparations: pinned; otherCatalogNumbers: ASHYE082-08, 08-SRNP-35746, BOLD:AAA5134; **Taxon:** scientificName: Hyphantrophaga morphophaga; phylum: Arthropoda; class: Insecta; order: Diptera; family: Tachinidae; genus: Hyphantrophaga; specificEpithet: morphophaga; scientificNameAuthorship: Fleming & Wood, 2018; **Location:** continent: Central America; country: Costa Rica; countryCode: CR; stateProvince: Guanacaste; county: Sector Cacao; locality: Area de Conservacion Guanacaste; verbatimLocality: Sendero Derrumbe; verbatimElevation: 1220; verbatimLatitude: 10.9292; verbatimLongitude: -85.4643; verbatimCoordinateSystem: Decimal; decimalLatitude: 10.9292; decimalLongitude: -85.4643; **Identification:** identifiedBy: AJ Fleming; dateIdentified: 2017; **Event:** samplingProtocol: Reared from the larva of the Nymphalidae, Morpho helenor; verbatimEventDate: 14-Aug-2008; **Record Level:** language: en; institutionCode: CNC; collectionCode: Insects; basisOfRecord: Pinned Specimen
- **Type status:** Other material. **Occurrence:** occurrenceDetails: http://janzen.sas.upenn.edu; catalogNumber: DHJPAR0057238; recordedBy: D.H. Janzen, W. Hallwachs & Lucia Rios; individualID: DHJPAR0057238; individualCount: 1; lifeStage: adult; preparations: pinned; otherCatalogNumbers: ACGBA5148-15, 14-SRNP-20989,; **Taxon:** scientificName: Hyphantrophaga morphophaga; phylum: Arthropoda; class: Insecta; order: Diptera; family: Tachinidae; genus: Hyphantrophaga; specificEpithet: morphophaga; scientificNameAuthorship: Fleming & Wood, 2018; **Location:** continent: Central America; country: Costa Rica; countryCode: CR; stateProvince: Guanacaste; county: Sector Del Oro; locality: Area de Conservacion Guanacaste; verbatimLocality: Finca Araya; verbatimElevation: 295; verbatimLatitude: 11.0154; verbatimLongitude: -85.5113; verbatimCoordinateSystem: Decimal; decimalLatitude: 11.0154; decimalLongitude: -85.5113; **Identification:** identifiedBy: AJ Fleming; dateIdentified: 2017; **Event:** samplingProtocol: Reared from the larva of the Nymphalidae, Morpho helenor; verbatimEventDate: 21-Oct-2014; **Record Level:** language: en; institutionCode: CNC; collectionCode: Insects; basisOfRecord: Pinned Specimen
- **Type status:** Other material. **Occurrence:** occurrenceDetails: http://janzen.sas.upenn.edu; catalogNumber: BIOUG28650-B08; recordedBy: D.H. Janzen, W. Hallwachs & D.Janzen, W.Hallwachs; individualID: BIOUG28650-B08; individualCount: 1; lifeStage: adult; preparations: pinned; otherCatalogNumbers: JICAJ024-16, GMP#06717, BOLD:AAA5134; **Taxon:** scientificName: Hyphantrophaga morphophaga; phylum: Arthropoda; class: Insecta; order: Diptera; family: Tachinidae; genus: Hyphantrophaga; specificEpithet: morphophaga; scientificNameAuthorship: Fleming & Wood, 2018; **Location:** continent: Central America; country: Costa Rica; countryCode: CR; stateProvince: Guanacaste; county: Pailas Dos; locality: Area de Conservacion Guanacaste; verbatimLocality: PL12-1; verbatimElevation: 828; verbatimLatitude: 10.7642; verbatimLongitude: -85.335; verbatimCoordinateSystem: Decimal; decimalLatitude: 10.7642; decimalLongitude: -85.335; **Identification:** identifiedBy: AJ Fleming; dateIdentified: 2017; **Event:** samplingProtocol: Reared from the larva of the Malaise trap PL12-1, Malaise trap PL12-1; verbatimEventDate: 05-Dec-2013; **Record Level:** language: en; institutionCode: CNC; collectionCode: Insects; basisOfRecord: Pinned Specimen
- **Type status:** Other material. **Occurrence:** occurrenceDetails: http://janzen.sas.upenn.edu; catalogNumber: DHJPAR0059908; recordedBy: D.H. Janzen, W. Hallwachs & Lucia Rios; individualID: DHJPAR0059908; individualCount: 1; lifeStage: adult; preparations: pinned; otherCatalogNumbers: ACGBA6329-16, 16-SRNP-21403, BOLD:AAA5134; **Taxon:** scientificName: Hyphantrophaga morphophaga; phylum: Arthropoda; class: Insecta; order: Diptera; family: Tachinidae; genus: Hyphantrophaga; specificEpithet: morphophaga; scientificNameAuthorship: Fleming & Wood, 2018; **Location:** continent: Central America; country: Costa Rica; countryCode: CR; stateProvince: Guanacaste; county: Sector Del Oro; locality: Area de Conservacion Guanacaste; verbatimLocality: Sendero Puertas; verbatimElevation: 400; verbatimLatitude: 11.0109; verbatimLongitude: -85.4882; verbatimCoordinateSystem: Decimal; decimalLatitude: 11.0109; decimalLongitude: -85.4882; **Identification:** identifiedBy: AJ Fleming; dateIdentified: 2017; **Event:** samplingProtocol: Reared from the larva of the Nymphalidae, Morpho helenor; verbatimEventDate: 16-Oct-2016; **Record Level:** language: en; institutionCode: CNC; collectionCode: Insects; basisOfRecord: Pinned Specimen
- **Type status:** Other material. **Occurrence:** occurrenceDetails: http://janzen.sas.upenn.edu; catalogNumber: DHJPAR0060314; recordedBy: D.H. Janzen, W. Hallwachs & Lucia Rios; individualID: DHJPAR0060314; individualCount: 1; lifeStage: adult; preparations: pinned; otherCatalogNumbers: ACGBA6735-17, 16-SRNP-21438, BOLD:AAA5134; **Taxon:** scientificName: Hyphantrophaga morphophaga; phylum: Arthropoda; class: Insecta; order: Diptera; family: Tachinidae; genus: Hyphantrophaga; specificEpithet: morphophaga; scientificNameAuthorship: Fleming & Wood, 2018; **Location:** continent: Central America; country: Costa Rica; countryCode: CR; stateProvince: Guanacaste; county: Sector Del Oro; locality: Area de Conservacion Guanacaste; verbatimLocality: Sendero Puertas; verbatimElevation: 400; verbatimLatitude: 11.0109; verbatimLongitude: -85.4882; verbatimCoordinateSystem: Decimal; decimalLatitude: 11.0109; decimalLongitude: -85.4882; **Identification:** identifiedBy: AJ Fleming; dateIdentified: 2017; **Event:** samplingProtocol: Reared from the larva of the Nymphalidae, Morpho helenor; verbatimEventDate: 03-Nov-2016; **Record Level:** language: en; institutionCode: CNC; collectionCode: Insects; basisOfRecord: Pinned Specimen

#### Description

**Male** (Fig. 20). Length: 7–11 mm. **Head** (Fig. 20b): vertex 1/4 of head width; two reclinate upper orbital setae; ocellar setae arising beside anterior ocellus; ocellar triangle dark blackened gold with light gold tomentum around margin; fronto-orbital plate dull grey tomentose with slight gold tinge, stronger around vertex; eye densely haired; fronto-orbital plate setulose, setulae not extending below lowest frontal seta; fronto-orbital plate shiny silver; parafacial bare; facial ridge bare; pedicel black with a small spot of orange basally, sometimes extending into adjacent region of postpedicel; otherwise concolorous with postpedicel; arista black, very minutely pubescent, gradually tapered apically beginning on basal 1/3–1/4; palpus ranging from yellow to brown. **Thorax** (Fig. 20a, c): entire thorax densely hirsute with short black setulae amongst setae; prosternum setose with 1–3 strong setae surrounded by a brush of weaker setulae; four prominent dorsal vittae, outermost pair broken across suture, innermost pair unbroken across suture, not reaching beyond 2nd postsutural dorsocentral seta; postpronotum with five setae arranged in a triangle; chaetotaxy: acrostichal setae 4:3; dorsocentral setae 4:4; intra-alar setae 3:3; supra-alar setae 2:3; katepisternum with three setae, basal seta weakest, arising anterior to suture; lateral scutellar setae 1/2 as long as subapical setae, slightly curving inwards medially; subapical setae subequal in length to basal scutellar setae, the latter arising above plane of remaining marginal scutellar setae; apical scutellar setae ranging from 1/2 as long as lateral scutellar setae to subequal in length but 1/2 the diameter; one pair of discal scutellar setae; scutellum gold tomentose along apical margin, transitioning to grey tomentose basally. **Legs** (Fig. 20c): femora and coxae black in ground colour; tibiae yellow in ground colour, densely covered in short black hairs, appearing darkened, almost black; fore femur with dense silver tomentum on posterodorsal surface; hind coxa either bare or with a single seta along posterior margin. **Wing** (Fig. 20a): pale translucent, not distinctly infuscate; vein R_4+5_ with 2–3 (most often 2) setulae at base. **Abdomen** (Fig. 20a, c): ground colour brown; mid-dorsal depression on ST1+2 extending almost to margin; median marginal setae absent on ST1+2 and T3; a complete row of marginal setae present on T4; discal setae only on T5; sex patch covering ventral surfaces of T4–T5, the posterior 1/3 of T3; distinct tomentose bands along anterior 2/3 of T3 and T4, broken medially by a dorsocentral stripe; T5 with silver tomentum over its entirety. **Terminalia** (Fig. 20d, e, f): sternite 5 (Fig. 20f) with a deeply excavated median cleft, smoothly U-shaped, margins covered in dense tomentum. Lateral lobes of sternite rounded apically, with 2–3 strong setae surrounded by many shorter, weaker setulae. Anterior plate of sternite 5 from subequal to slightly longer than apical lobes; unsclerotised "window" appearing blunt umbonate with a slightly rectangular base and a wider crown making it appear mushroom-shaped, as wide as median cleft. Cerci in posterior view (Fig. 20d) rectangular and slightly shorter than surstyli, blunt and rounded at apex, completely separate medially appearing slightly divergent basally, twice as wide as apex; in lateral view with a slight downward curve in apical 1/3; densely setulose along basal 2/3, setulose ventrally along entire length (visible in lateral view). Surstylus in lateral view (Fig. 20e) narrow, almost parallel-sided along its entire length, ending in a slightly downcurved apex, making the structure appear blade-like; when viewed dorsally, surstyli appearing to point outwards. Pregonite short and wide, well-developed, subequal in length to postgonite, as long as distiphallus, bare and squared-off apically. Postgonite slightly narrow 1/3 as wide as pregonite, sharply pointed and curved at apex. Distiphallus rectangular, only weakly flaring apically with a slender, median longitudinal sclerotised reinforcement on its posterior surface and a broad, anterolateral, apically clubbed sclerotised acrophallus on each side, joining the plate of opposite side on anterior surface near apex.

**Female**. Length: 8–11 mm. As male, differing only by the presence of two pairs of proclinate orbital setae.

#### Diagnosis

To date *Hyphantrophaga morphophaga* **sp. n.** can only be distinguished from its closest congener *H. danausophaga* **sp. n.** by its host selection and habitat, being found parasitising only large, hairy, non-ringed cryptic Nymphalidae larvae in densely shaded habitat within the forest.

#### Etymology

Named after the genus *Morpho* and the Greek "phago" meaning "eating", with reference to its primary hosts.

#### Distribution

Costa Rica, ACG, Alajuela and Guanacaste Provinces, 17–1276 m elevation.

#### Ecology

*Hyphantrophaga morphophaga* **sp. n.** has been reared 130 times from three species of Lepidoptera in the family Nymphalidae, *Morpho amathonte* (Deyrolle, 1860), *Morpho polyphemus catalina* (Corea and Chacon, 1984) and *Morpho helenor* (Catalina, 1776), in cloud forest, rain forest, dry forest and dry-rain lowland intergrade.

### Hyphantrophaga myersi

(Aldrich, 1933)

#### Materials

- **Type status:** Holotype. **Occurrence:** recordedBy: J.G. Myers; individualCount: 1; sex: male; preparations: pinned; otherCatalogNumbers: Cat. No. 49789 U. S. N. M.; **Taxon:** scientificName: Hyphantrophaga myersi; phylum: Arthropoda; class: Insecta; order: Diptera; family: Tachinidae; genus: Prophryno; specificEpithet: myersi; scientificNameAuthorship: (Aldrich, 1933); **Location:** continent: South America; country: Guyana (as British Guiana); stateProvince: Pakaraima (as Pakeraima); verbatimLocality: Upper Ireng River; **Identification:** identifiedBy: M. Wood; **Event:** samplingProtocol: Reared from the larva of the Selenis suere Cramer [sic]; eventDate: March, 1931; **Record Level:** language: en; institutionCode: BMNH; collectionCode: Insects; basisOfRecord: Pinned Specimen
- **Type status:** Other material. **Occurrence:** occurrenceDetails: http://janzen.sas.upenn.edu; catalogNumber: DHJPAR0007460; recordedBy: D.H. Janzen, W. Hallwachs & Osvaldo Espinoza; individualID: DHJPAR0007460; individualCount: 1; lifeStage: adult; preparations: pinned; otherCatalogNumbers: ASTAT232-06, 98-SRNP-6859, BOLD:AAA9858; **Taxon:** scientificName: Hyphantrophaga myersi; phylum: Arthropoda; class: Insecta; order: Diptera; family: Tachinidae; genus: Hyphantrophaga; specificEpithet: myersi; scientificNameAuthorship: (Aldrich, 1933); **Location:** continent: Central America; country: Costa Rica; countryCode: CR; stateProvince: Alajuela; county: Sector San Cristobal; locality: Area de Conservacion Guanacaste; verbatimLocality: Estacion San Cristobal; verbatimElevation: 640; verbatimLatitude: 10.871; verbatimLongitude: -85.3914; verbatimCoordinateSystem: Decimal; decimalLatitude: 10.871; decimalLongitude: -85.3914; **Identification:** identifiedBy: AJ Fleming; dateIdentified: 2017; **Event:** samplingProtocol: Reared from the larva of the Crambidae, Portentomorpha xanthialis; verbatimEventDate: 30-Jul-1998; **Record Level:** language: en; institutionCode: CNC; collectionCode: Insects; basisOfRecord: Pinned Specimen
- **Type status:** Other material. **Occurrence:** occurrenceDetails: http://janzen.sas.upenn.edu; catalogNumber: DHJPAR0007445; recordedBy: D.H. Janzen, W. Hallwachs & Guillermo Pereira; individualID: DHJPAR0007445; individualCount: 1; lifeStage: adult; preparations: pinned; otherCatalogNumbers: ASTAT217-06, 03-SRNP-13817, BOLD:AAA9858; **Taxon:** scientificName: Hyphantrophaga myersi; phylum: Arthropoda; class: Insecta; order: Diptera; family: Tachinidae; genus: Hyphantrophaga; specificEpithet: myersi; scientificNameAuthorship: (Aldrich, 1933); **Location:** continent: Central America; country: Costa Rica; countryCode: CR; stateProvince: Guanacaste; county: Sector Horizontes; locality: Area de Conservacion Guanacaste; verbatimLocality: Bejuco; verbatimElevation: 180; verbatimLatitude: 10.7671; verbatimLongitude: -85.5966; verbatimCoordinateSystem: Decimal; decimalLatitude: 10.7671; decimalLongitude: -85.5966; **Identification:** identifiedBy: AJ Fleming; dateIdentified: 2017; **Event:** samplingProtocol: Reared from the larva of the Crambidae, Portentomorpha xanthialis; verbatimEventDate: 24-Jul-2003; **Record Level:** language: en; institutionCode: CNC; collectionCode: Insects; basisOfRecord: Pinned Specimen
- **Type status:** Other material. **Occurrence:** occurrenceDetails: http://janzen.sas.upenn.edu; catalogNumber: DHJPAR0007447; recordedBy: D.H. Janzen, W. Hallwachs & Jose Cortez; individualID: DHJPAR0007447; individualCount: 1; lifeStage: adult; preparations: pinned; otherCatalogNumbers: ASTAT219-06, 03-SRNP-13919, BOLD:AAA9858; **Taxon:** scientificName: Hyphantrophaga myersi; phylum: Arthropoda; class: Insecta; order: Diptera; family: Tachinidae; genus: Hyphantrophaga; specificEpithet: myersi; scientificNameAuthorship: (Aldrich, 1933); **Location:** continent: Central America; country: Costa Rica; countryCode: CR; stateProvince: Guanacaste; county: Sector Horizontes; locality: Area de Conservacion Guanacaste; verbatimLocality: Bejuco; verbatimElevation: 180; verbatimLatitude: 10.7671; verbatimLongitude: -85.5966; verbatimCoordinateSystem: Decimal; decimalLatitude: 10.7671; decimalLongitude: -85.5966; **Identification:** identifiedBy: AJ Fleming; dateIdentified: 2017; **Event:** samplingProtocol: Reared from the larva of the Crambidae, Portentomorpha xanthialis; verbatimEventDate: 26-Jul-2003; **Record Level:** language: en; institutionCode: CNC; collectionCode: Insects; basisOfRecord: Pinned Specimen
- **Type status:** Other material. **Occurrence:** occurrenceDetails: http://janzen.sas.upenn.edu; catalogNumber: DHJPAR0007461; recordedBy: D.H. Janzen, W. Hallwachs & Osvaldo Espinoza; individualID: DHJPAR0007461; individualCount: 1; lifeStage: adult; preparations: pinned; otherCatalogNumbers: ASTAT233-06, 98-SRNP-6878, BOLD:AAA9858; **Taxon:** scientificName: Hyphantrophaga myersi; phylum: Arthropoda; class: Insecta; order: Diptera; family: Tachinidae; genus: Hyphantrophaga; specificEpithet: myersi; scientificNameAuthorship: (Aldrich, 1933); **Location:** continent: Central America; country: Costa Rica; countryCode: CR; stateProvince: Alajuela; county: Sector San Cristobal; locality: Area de Conservacion Guanacaste; verbatimLocality: Estacion San Cristobal; verbatimElevation: 640; verbatimLatitude: 10.871; verbatimLongitude: -85.3914; verbatimCoordinateSystem: Decimal; decimalLatitude: 10.871; decimalLongitude: -85.3914; **Identification:** identifiedBy: AJ Fleming; dateIdentified: 2017; **Event:** samplingProtocol: Reared from the larva of the Crambidae, Hoterodes ausonia; verbatimEventDate: 28-Jul-1998; **Record Level:** language: en; institutionCode: CNC; collectionCode: Insects; basisOfRecord: Pinned Specimen
- **Type status:** Other material. **Occurrence:** occurrenceDetails: http://janzen.sas.upenn.edu; catalogNumber: DHJPAR0007450; recordedBy: D.H. Janzen, W. Hallwachs & Guillermo Pereira; individualID: DHJPAR0007450; individualCount: 1; lifeStage: adult; preparations: pinned; otherCatalogNumbers: ASTAT222-06, 03-SRNP-13852, BOLD:AAA9858; **Taxon:** scientificName: Hyphantrophaga myersi; phylum: Arthropoda; class: Insecta; order: Diptera; family: Tachinidae; genus: Hyphantrophaga; specificEpithet: myersi; scientificNameAuthorship: (Aldrich, 1933); **Location:** continent: Central America; country: Costa Rica; countryCode: CR; stateProvince: Guanacaste; county: Sector Horizontes; locality: Area de Conservacion Guanacaste; verbatimLocality: Bejuco; verbatimElevation: 180; verbatimLatitude: 10.7671; verbatimLongitude: -85.5966; verbatimCoordinateSystem: Decimal; decimalLatitude: 10.7671; decimalLongitude: -85.5966; **Identification:** identifiedBy: AJ Fleming; dateIdentified: 2017; **Event:** samplingProtocol: Reared from the larva of the Crambidae, Portentomorpha xanthialis; verbatimEventDate: 26-Jul-2003; **Record Level:** language: en; institutionCode: CNC; collectionCode: Insects; basisOfRecord: Pinned Specimen

#### Description

**Male** (Fig. 21). Length: 5–7 mm. **Head** (Fig. 21b): vertex 1/3 of head width; two reclinate upper orbital setae; ocellar setae arising slightly behind anterior ocellus; ocellar triangle gold, concolorous with fronto-orbital plate; fronto-orbital plate entirely gold and sparsely setulose throughout, setulae not reaching below lowest frontal seta; parafacial shiny silver and bare; facial ridge setose along its entire length; eye with short sparse ommatrichia up to 2X as long as one ommatidium; pedicel light brown; postpedicel black; arista brown, bare, distinctly thickened on basal 1/3–1/4; palpus dark amber yellow and haired. **Thorax** (Fig. 21a, c): pale brassy-silver tomentose dorsally, grey tomentose laterally; four prominent dorsal vittae, outermost two slightly broken across suture, innermost pair unbroken across suture, reaching 2nd postsutural dorsocentral seta; postpronotum with 3–4 setae arranged in a triangle; chaetotaxy: acrostichal setae 3:3; dorsocentral setae 3:4; intra-alar setae 3–4:3; supra-alar setae 2:3; three katepisternal setae; lateral scutellar setae shorter than subapical scutellar setae; apical scutellar setae curving inwards medially and slightly upturned so as to rise above the plane of remaining scutellar setae; one pair of discal scutellar setae; scutellum slightly darker and grey along basal 3/4, apically concolorous with scutum. **Legs** (Fig. 21c): femora and coxae black in ground colour; tibiae yellow in ground colour, densely covered in short black hairs, appearing darkened, almost black; fore femur with dense silver tomentum on posterodorsal surface; hind coxa with a single seta along posterior margin. **Wing** (Fig. 21a): pale translucent, hyaline, not distinctly infuscate; vein R_4+5_ with only two setulae at base. **Abdomen** (Fig. 21a, c): ground colour black; mid-dorsal depression on ST1+2 extending only halfway across tergite; median marginal setae absent on ST1+2 and weak on T3; T4–T5 with a complete row of marginal setae; discal setae absent on all tergites; sex patch covering ventral surfaces of T4–T5; dorsal surface of abdomen covered in brassy-silver tomentum on ST1+2–T4; T5 with brilliant gold tomentum over its entirety. **Terminalia**: sternite 5 (Fig. 21f) with a deeply excavated median cleft, rounded U-shaped, inner margins covered in dense tomentum. Lateral lobes of sternite rounded apically, with 2–3 strong setae surrounded by 2–3 shorter, weaker setulae. Anterior plate of sternite 5 from subequal to slightly longer than apical lobes, unsclerotised "window" rectangular, 1/2 as wide as median cleft and slightly convex. Cerci in posterior view (Fig. 21d) slightly rectangular, shorter than surstyli, blunt and rounded at apex, fused along basal 2/3, separating medially along apical 1/3; in lateral view with a strong downward curve in apical 1/3; densely setulose along basal 2/3 dorsally, setulose ventrally along entire length (visible in lateral view). Surstylus in lateral view (Fig. 21e) almost parallel-sided along its length, rounded at tip; surstylus appearing to be fused with epandrium; when viewed dorsally, surstyli appearing slender and straight, not strongly convergent. Pregonite broad and well-developed, bent medially, apically rounded, devoid of setulae. Postgonite elongate, slender, very slightly hooked at tip, subequal in length to pregonite. Distiphallus vaguely cone-shaped, could be seen as rectangular, weakly flaring apically, with a slender median longitudinal sclerotised reinforcement on its posterior surface and a broad, anterolateral, sclerotised acrophallus on each side, joining the plate of opposite side on anterior surface near apex.

**Female**. Length: 5–7 mm. As male, differing only by the presence of two pairs of proclinate orbital setae.

#### Diagnosis

*Hyphantrophaga myersi* (Aldrich) can be distinguished from all other *Hyphantrophaga* species by the following combination of traits: facial ridge with stout setae along entire length and sternite 5 distinctly golden tomentose.

#### Distribution

Neotropical species recorded from South America: Guyana, Venezuela; Lesser Antilles: Trinidad & Tobago; and Mesoamerica: Costa Rica, ACG (Provinces of Alajuela and Guanacaste), 180–640 m elevation.

#### Ecology

*Hyphantrophaga myersi* has been reared 43 times within the ACG inventory from eight species of Lepidoptera, six in the family Erebidae (*Renodes curviluna* (Druce, 1890), *Anomis illita* Guenée, 1852, *Anomis luridula* Guenée, 1852, *Eulepidotis folium* (Schaus, 1911), *Helia sueroides* (Guenée, 1852) and *Dysschema lycaste* (Klug, 1836)); and two in the family Geometridae (*Semiothisa* sp. and geoJanzen01 Janzen17), in rain forest, dry forest and dry-rain lowland intergrade.

### Hyphantrophaga nigricauda

Fleming & Wood sp. n.

urn:lsid:zoobank.org:act:E866E684-19A6-4A47-A070-0935DFA78F75

#### Materials

- **Type status:** Holotype. **Occurrence:** occurrenceDetails: http://janzen.sas.upenn.edu; catalogNumber: DHJPAR0027908; recordedBy: D.H. Janzen, W. Hallwachs & Leonel Siezar; individualID: DHJPAR0027908; individualCount: 1; sex: male; lifeStage: adult; preparations: pinned; otherCatalogNumbers: ASHYE145-08, 08-SRNP-70578, BOLD:AAA1929; **Taxon:** scientificName: Hyphantrophaga nigricauda; phylum: Arthropoda; class: Insecta; order: Diptera; family: Tachinidae; genus: Hyphantrophaga; specificEpithet: nigricauda; scientificNameAuthorship: Fleming & Wood, 2017; **Location:** continent: Central America; country: Costa Rica; countryCode: CR; stateProvince: Guanacaste; county: Sector Pitilla; locality: Area de Conservacion Guanacaste; verbatimLocality: Leonel; verbatimElevation: 510; verbatimLatitude: 10.9964; verbatimLongitude: -85.4019; verbatimCoordinateSystem: Decimal; decimalLatitude: 10.9964; decimalLongitude: -85.4019; **Identification:** identifiedBy: AJ Fleming; dateIdentified: 2017; **Event:** samplingProtocol: Reared from the larva of the Geometridae, Cyclomia disparilis; verbatimEventDate: 28-Jun-2008; **Record Level:** language: en; institutionCode: CNC; collectionCode: Insects; basisOfRecord: Pinned Specimen
- **Type status:** Paratype. **Occurrence:** occurrenceDetails: http://janzen.sas.upenn.edu; catalogNumber: DHJPAR0007291; recordedBy: D.H. Janzen, W. Hallwachs & Calixto Moraga; individualID: DHJPAR0007291; individualCount: 1; sex: female; lifeStage: adult; preparations: pinned; otherCatalogNumbers: ASTAT063-06, 05-SRNP-32268, BOLD:AAA1929; **Taxon:** scientificName: Hyphantrophaga nigricauda; phylum: Arthropoda; class: Insecta; order: Diptera; family: Tachinidae; genus: Hyphantrophaga; specificEpithet: nigricauda; scientificNameAuthorship: Fleming & Wood, 2017; **Location:** continent: Central America; country: Costa Rica; countryCode: CR; stateProvince: Guanacaste; county: Sector Pitilla; locality: Area de Conservacion Guanacaste; verbatimLocality: Pasmompa; verbatimElevation: 440; verbatimLatitude: 11.0193; verbatimLongitude: -85.41; verbatimCoordinateSystem: Decimal; decimalLatitude: 11.0193; decimalLongitude: -85.41; **Identification:** identifiedBy: AJ Fleming; dateIdentified: 2017; **Event:** samplingProtocol: Reared from the larva of the Geometridae, Cyclomia disparilis; verbatimEventDate: 20-Jul-2005; **Record Level:** language: en; institutionCode: CNC; collectionCode: Insects; basisOfRecord: Pinned Specimen
- **Type status:** Paratype. **Occurrence:** occurrenceDetails: http://janzen.sas.upenn.edu; catalogNumber: DHJPAR0042693; recordedBy: D.H. Janzen, W. Hallwachs & Freddy Quesada; individualID: DHJPAR0042693; individualCount: 1; sex: female; lifeStage: adult; preparations: pinned; otherCatalogNumbers: ASHYH451-11, 11-SRNP-31067, BOLD:AAA1929; **Taxon:** scientificName: Hyphantrophaga nigricauda; phylum: Arthropoda; class: Insecta; order: Diptera; family: Tachinidae; genus: Hyphantrophaga; specificEpithet: nigricauda; scientificNameAuthorship: Fleming & Wood, 2017; **Location:** continent: Central America; country: Costa Rica; countryCode: CR; stateProvince: Guanacaste; county: Sector Pitilla; locality: Area de Conservacion Guanacaste; verbatimLocality: Sendero Laguna; verbatimElevation: 680; verbatimLatitude: 10.9888; verbatimLongitude: -85.4234; verbatimCoordinateSystem: Decimal; decimalLatitude: 10.9888; decimalLongitude: -85.4234; **Identification:** identifiedBy: AJ Fleming; dateIdentified: 2017; **Event:** samplingProtocol: Reared from the larva of the Crambidae, Eulepte Solis15; verbatimEventDate: 20-May-2011; **Record Level:** language: en; institutionCode: CNC; collectionCode: Insects; basisOfRecord: Pinned Specimen
- **Type status:** Paratype. **Occurrence:** occurrenceDetails: http://janzen.sas.upenn.edu; catalogNumber: DHJPAR0007293; recordedBy: D.H. Janzen, W. Hallwachs & Calixto Moraga; individualID: DHJPAR0007293; individualCount: 1; sex: female; lifeStage: adult; preparations: pinned; otherCatalogNumbers: ASTAT065-06, 05-SRNP-32270, BOLD:AAA1929; **Taxon:** scientificName: Hyphantrophaga nigricauda; phylum: Arthropoda; class: Insecta; order: Diptera; family: Tachinidae; genus: Hyphantrophaga; specificEpithet: nigricauda; scientificNameAuthorship: Fleming & Wood, 2017; **Location:** continent: Central America; country: Costa Rica; countryCode: CR; stateProvince: Guanacaste; county: Sector Pitilla; locality: Area de Conservacion Guanacaste; verbatimLocality: Pasmompa; verbatimElevation: 440; verbatimLatitude: 11.0193; verbatimLongitude: -85.41; verbatimCoordinateSystem: Decimal; decimalLatitude: 11.0193; decimalLongitude: -85.41; **Identification:** identifiedBy: AJ Fleming; dateIdentified: 2017; **Event:** samplingProtocol: Reared from the larva of the Geometridae, Cyclomia disparilis; verbatimEventDate: 20-Jul-2005; **Record Level:** language: en; institutionCode: CNC; collectionCode: Insects; basisOfRecord: Pinned Specimen
- **Type status:** Paratype. **Occurrence:** occurrenceDetails: http://janzen.sas.upenn.edu; catalogNumber: DHJPAR0007290; recordedBy: D.H. Janzen, W. Hallwachs & Calixto Moraga; individualID: DHJPAR0007290; individualCount: 1; sex: male; lifeStage: adult; preparations: pinned; otherCatalogNumbers: ASTAT062-06, 05-SRNP-32248, BOLD:AAA1929; **Taxon:** scientificName: Hyphantrophaga nigricauda; phylum: Arthropoda; class: Insecta; order: Diptera; family: Tachinidae; genus: Hyphantrophaga; specificEpithet: nigricauda; scientificNameAuthorship: Fleming & Wood, 2017; **Location:** continent: Central America; country: Costa Rica; countryCode: CR; stateProvince: Guanacaste; county: Sector Pitilla; locality: Area de Conservacion Guanacaste; verbatimLocality: Pasmompa; verbatimElevation: 440; verbatimLatitude: 11.0193; verbatimLongitude: -85.41; verbatimCoordinateSystem: Decimal; decimalLatitude: 11.0193; decimalLongitude: -85.41; **Identification:** identifiedBy: AJ Fleming; dateIdentified: 2017; **Event:** samplingProtocol: Reared from the larva of the Geometridae, Cyclomia disparilis; verbatimEventDate: 16-Jul-2005; **Record Level:** language: en; institutionCode: CNC; collectionCode: Insects; basisOfRecord: Pinned Specimen
- **Type status:** Paratype. **Occurrence:** occurrenceDetails: http://janzen.sas.upenn.edu; catalogNumber: DHJPAR0036438; recordedBy: D.H. Janzen, W. Hallwachs & Guillermo Pereira; individualID: DHJPAR0036438; individualCount: 1; sex: female; lifeStage: adult; preparations: pinned; otherCatalogNumbers: ASHYD1629-09, 09-SRNP-14037, BOLD:AAA1929; **Taxon:** scientificName: Hyphantrophaga nigricauda; phylum: Arthropoda; class: Insecta; order: Diptera; family: Tachinidae; genus: Hyphantrophaga; specificEpithet: nigricauda; scientificNameAuthorship: Fleming & Wood, 2017; **Location:** continent: Central America; country: Costa Rica; countryCode: CR; stateProvince: Guanacaste; county: Sector Santa Rosa; locality: Area de Conservacion Guanacaste; verbatimLocality: Camino Borrachos; verbatimElevation: 295; verbatimLatitude: 10.8429; verbatimLongitude: -85.6161; verbatimCoordinateSystem: Decimal; decimalLatitude: 10.8429; decimalLongitude: -85.6161; **Identification:** identifiedBy: AJ Fleming; dateIdentified: 2017; **Event:** samplingProtocol: Reared from the larva of the Crambidae, Conchylodes platinalis; verbatimEventDate: 01-Aug-2009; **Record Level:** language: en; institutionCode: CNC; collectionCode: Insects; basisOfRecord: Pinned Specimen
- **Type status:** Paratype. **Occurrence:** occurrenceDetails: http://janzen.sas.upenn.edu; catalogNumber: DHJPAR0035694; recordedBy: D.H. Janzen, W. Hallwachs & Jose Perez; individualID: DHJPAR0035694; individualCount: 1; sex: female; lifeStage: adult; preparations: pinned; otherCatalogNumbers: ASHYD1075-09, 09-SRNP-41180, BOLD:AAA1929; **Taxon:** scientificName: Hyphantrophaga nigricauda; phylum: Arthropoda; class: Insecta; order: Diptera; family: Tachinidae; genus: Hyphantrophaga; specificEpithet: nigricauda; scientificNameAuthorship: Fleming & Wood, 2017; **Location:** continent: Central America; country: Costa Rica; countryCode: CR; stateProvince: Alajuela; county: Sector Rincon Rain Forest; locality: Area de Conservacion Guanacaste; verbatimLocality: Sendero Juntas; verbatimElevation: 400; verbatimLatitude: 10.9066; verbatimLongitude: -85.2878; verbatimCoordinateSystem: Decimal; decimalLatitude: 10.9066; decimalLongitude: -85.2878; **Identification:** identifiedBy: AJ Fleming; dateIdentified: 2017; **Event:** samplingProtocol: Reared from the larva of the Geometridae, Cyclomia disparilis; verbatimEventDate: 01-Jul-2009; **Record Level:** language: en; institutionCode: CNC; collectionCode: Insects; basisOfRecord: Pinned Specimen
- **Type status:** Paratype. **Occurrence:** occurrenceDetails: http://janzen.sas.upenn.edu; catalogNumber: DHJPAR0010356; recordedBy: D.H. Janzen, W. Hallwachs & Minor Carmona; individualID: DHJPAR0010356; individualCount: 1; sex: female; lifeStage: adult; preparations: pinned; otherCatalogNumbers: ASTAS187-06, 06-SRNP-42821, BOLD:AAA1929; **Taxon:** scientificName: Hyphantrophaga nigricauda; phylum: Arthropoda; class: Insecta; order: Diptera; family: Tachinidae; genus: Hyphantrophaga; specificEpithet: nigricauda; scientificNameAuthorship: Fleming & Wood, 2017; **Location:** continent: Central America; country: Costa Rica; countryCode: CR; stateProvince: Alajuela; county: Sector Rincon Rain Forest; locality: Area de Conservacion Guanacaste; verbatimLocality: Sendero Guaca; verbatimElevation: 400; verbatimLatitude: 10.9061; verbatimLongitude: -85.2828; verbatimCoordinateSystem: Decimal; decimalLatitude: 10.9061; decimalLongitude: -85.2828; **Identification:** identifiedBy: AJ Fleming; dateIdentified: 2017; **Event:** samplingProtocol: Reared from the larva of the Crambidae, Pleuroptya Solis01; verbatimEventDate: 31-Aug-2006; **Record Level:** language: en; institutionCode: CNC; collectionCode: Insects; basisOfRecord: Pinned Specimen
- **Type status:** Paratype. **Occurrence:** occurrenceDetails: http://janzen.sas.upenn.edu; catalogNumber: DHJPAR0011461; recordedBy: D.H. Janzen, W. Hallwachs & Jose Perez; individualID: DHJPAR0011461; individualCount: 1; sex: male; lifeStage: adult; preparations: pinned; otherCatalogNumbers: ASTAQ848-06, 01-SRNP-5433, BOLD:AAA1929; **Taxon:** scientificName: Hyphantrophaga nigricauda; phylum: Arthropoda; class: Insecta; order: Diptera; family: Tachinidae; genus: Hyphantrophaga; specificEpithet: nigricauda; scientificNameAuthorship: Fleming & Wood, 2017; **Location:** continent: Central America; country: Costa Rica; countryCode: CR; stateProvince: Alajuela; county: Sector Rincon Rain Forest; locality: Area de Conservacion Guanacaste; verbatimLocality: Quebrada Escondida; verbatimElevation: 420; verbatimLatitude: 10.8993; verbatimLongitude: -85.2749; verbatimCoordinateSystem: Decimal; decimalLatitude: 10.8993; decimalLongitude: -85.2749; **Identification:** identifiedBy: AJ Fleming; dateIdentified: 2017; **Event:** samplingProtocol: Reared from the larva of the Geometridae, Cyclomia disparilis; verbatimEventDate: 19-Aug-2001; **Record Level:** language: en; institutionCode: CNC; collectionCode: Insects; basisOfRecord: Pinned Specimen
- **Type status:** Paratype. **Occurrence:** occurrenceDetails: http://janzen.sas.upenn.edu; catalogNumber: DHJPAR0042303; recordedBy: D.H. Janzen, W. Hallwachs & Edwin Apu; individualID: DHJPAR0042303; individualCount: 1; sex: male; lifeStage: adult; preparations: pinned; otherCatalogNumbers: ASHYH067-11, 11-SRNP-69487, BOLD:AAA1929; **Taxon:** scientificName: Hyphantrophaga nigricauda; phylum: Arthropoda; class: Insecta; order: Diptera; family: Tachinidae; genus: Hyphantrophaga; specificEpithet: nigricauda; scientificNameAuthorship: Fleming & Wood, 2017; **Location:** continent: Central America; country: Costa Rica; countryCode: CR; stateProvince: Alajuela; county: Sector Rincon Rain Forest; locality: Area de Conservacion Guanacaste; verbatimLocality: Jacobo; verbatimElevation: 461; verbatimLatitude: 10.9408; verbatimLongitude: -85.3177; verbatimCoordinateSystem: Decimal; decimalLatitude: 10.9408; decimalLongitude: -85.3177; **Identification:** identifiedBy: AJ Fleming; dateIdentified: 2017; **Event:** samplingProtocol: Reared from the larva of the Geometridae, Cyclomia disparilis; verbatimEventDate: 07-Mar-2011; **Record Level:** language: en; institutionCode: CNC; collectionCode: Insects; basisOfRecord: Pinned Specimen
- **Type status:** Paratype. **Occurrence:** occurrenceDetails: http://janzen.sas.upenn.edu; catalogNumber: DHJPAR0007294; recordedBy: D.H. Janzen, W. Hallwachs & Calixto Moraga; individualID: DHJPAR0007294; individualCount: 1; sex: female; lifeStage: adult; preparations: pinned; otherCatalogNumbers: ASTAT066-06, 05-SRNP-32262, BOLD:AAA1929; **Taxon:** scientificName: Hyphantrophaga nigricauda; phylum: Arthropoda; class: Insecta; order: Diptera; family: Tachinidae; genus: Hyphantrophaga; specificEpithet: nigricauda; scientificNameAuthorship: Fleming & Wood, 2017; **Location:** continent: Central America; country: Costa Rica; countryCode: CR; stateProvince: Guanacaste; county: Sector Pitilla; locality: Area de Conservacion Guanacaste; verbatimLocality: Pasmompa; verbatimElevation: 440; verbatimLatitude: 11.0193; verbatimLongitude: -85.41; verbatimCoordinateSystem: Decimal; decimalLatitude: 11.0193; decimalLongitude: -85.41; **Identification:** identifiedBy: AJ Fleming; dateIdentified: 2017; **Event:** samplingProtocol: Reared from the larva of the Geometridae, Cyclomia disparilis; verbatimEventDate: 17-Jul-2005; **Record Level:** language: en; institutionCode: CNC; collectionCode: Insects; basisOfRecord: Pinned Specimen
- **Type status:** Paratype. **Occurrence:** occurrenceDetails: http://janzen.sas.upenn.edu; catalogNumber: DHJPAR0027944; recordedBy: D.H. Janzen, W. Hallwachs & Roster Moraga; individualID: DHJPAR0027944; individualCount: 1; sex: female; lifeStage: adult; preparations: pinned; otherCatalogNumbers: ASHYE181-08, 08-SRNP-21716, BOLD:AAA1929; **Taxon:** scientificName: Hyphantrophaga nigricauda; phylum: Arthropoda; class: Insecta; order: Diptera; family: Tachinidae; genus: Hyphantrophaga; specificEpithet: nigricauda; scientificNameAuthorship: Fleming & Wood, 2017; **Location:** continent: Central America; country: Costa Rica; countryCode: CR; stateProvince: Guanacaste; county: Sector Del Oro; locality: Area de Conservacion Guanacaste; verbatimLocality: Monte Cristo; verbatimElevation: 525; verbatimLatitude: 11.0137; verbatimLongitude: -85.4253; verbatimCoordinateSystem: Decimal; decimalLatitude: 11.0137; decimalLongitude: -85.4253; **Identification:** identifiedBy: AJ Fleming; dateIdentified: 2017; **Event:** samplingProtocol: Reared from the larva of the Geometridae, Cyclomia disparilis; verbatimEventDate: 17-Jul-2008; **Record Level:** language: en; institutionCode: CNC; collectionCode: Insects; basisOfRecord: Pinned Specimen
- **Type status:** Paratype. **Occurrence:** occurrenceDetails: http://janzen.sas.upenn.edu; catalogNumber: DHJPAR0035815; recordedBy: D.H. Janzen, W. Hallwachs & Noe Castillo; individualID: DHJPAR0035815; individualCount: 1; sex: male; lifeStage: adult; preparations: pinned; otherCatalogNumbers: ASHYD1196-09, 09-SRNP-69335, BOLD:AAA1929; **Taxon:** scientificName: Hyphantrophaga nigricauda; phylum: Arthropoda; class: Insecta; order: Diptera; family: Tachinidae; genus: Hyphantrophaga; specificEpithet: nigricauda; scientificNameAuthorship: Fleming & Wood, 2017; **Location:** continent: Central America; country: Costa Rica; countryCode: CR; stateProvince: Alajuela; county: Sector Rincon Rain Forest; locality: Area de Conservacion Guanacaste; verbatimLocality: Jacobo; verbatimElevation: 461; verbatimLatitude: 10.9408; verbatimLongitude: -85.3177; verbatimCoordinateSystem: Decimal; decimalLatitude: 10.9408; decimalLongitude: -85.3177; **Identification:** identifiedBy: AJ Fleming; dateIdentified: 2017; **Event:** samplingProtocol: Reared from the larva of the Geometridae, Cyclomia disparilis; verbatimEventDate: 13-Jul-2009; **Record Level:** language: en; institutionCode: CNC; collectionCode: Insects; basisOfRecord: Pinned Specimen
- **Type status:** Paratype. **Occurrence:** occurrenceDetails: http://janzen.sas.upenn.edu; catalogNumber: DHJPAR0007295; recordedBy: D.H. Janzen, W. Hallwachs & Calixto Moraga; individualID: DHJPAR0007295; individualCount: 1; sex: female; lifeStage: adult; preparations: pinned; otherCatalogNumbers: ASTAT067-06, 05-SRNP-32276, BOLD:AAA1929; **Taxon:** scientificName: Hyphantrophaga nigricauda; phylum: Arthropoda; class: Insecta; order: Diptera; family: Tachinidae; genus: Hyphantrophaga; specificEpithet: nigricauda; scientificNameAuthorship: Fleming & Wood, 2017; **Location:** continent: Central America; country: Costa Rica; countryCode: CR; stateProvince: Guanacaste; county: Sector Pitilla; locality: Area de Conservacion Guanacaste; verbatimLocality: Pasmompa; verbatimElevation: 440; verbatimLatitude: 11.0193; verbatimLongitude: -85.41; verbatimCoordinateSystem: Decimal; decimalLatitude: 11.0193; decimalLongitude: -85.41; **Identification:** identifiedBy: AJ Fleming; dateIdentified: 2017; **Event:** samplingProtocol: Reared from the larva of the Geometridae, Cyclomia disparilis; verbatimEventDate: 14-Jul-2005; **Record Level:** language: en; institutionCode: CNC; collectionCode: Insects; basisOfRecord: Pinned Specimen
- **Type status:** Paratype. **Occurrence:** occurrenceDetails: http://janzen.sas.upenn.edu; catalogNumber: DHJPAR0042702; recordedBy: D.H. Janzen, W. Hallwachs & Freddy Quesada; individualID: DHJPAR0042702; individualCount: 1; sex: male; lifeStage: adult; preparations: pinned; otherCatalogNumbers: ASHYH460-11, 11-SRNP-31063, BOLD:AAA1929; **Taxon:** scientificName: Hyphantrophaga nigricauda; phylum: Arthropoda; class: Insecta; order: Diptera; family: Tachinidae; genus: Hyphantrophaga; specificEpithet: nigricauda; scientificNameAuthorship: Fleming & Wood, 2017; **Location:** continent: Central America; country: Costa Rica; countryCode: CR; stateProvince: Guanacaste; county: Sector Pitilla; locality: Area de Conservacion Guanacaste; verbatimLocality: Sendero Laguna; verbatimElevation: 680; verbatimLatitude: 10.9888; verbatimLongitude: -85.4234; verbatimCoordinateSystem: Decimal; decimalLatitude: 10.9888; decimalLongitude: -85.4234; **Identification:** identifiedBy: AJ Fleming; dateIdentified: 2017; **Event:** samplingProtocol: Reared from the larva of the Crambidae, Eulepte Solis15; verbatimEventDate: 19-May-2011; **Record Level:** language: en; institutionCode: CNC; collectionCode: Insects; basisOfRecord: Pinned Specimen
- **Type status:** Paratype. **Occurrence:** occurrenceDetails: http://janzen.sas.upenn.edu; catalogNumber: DHJPAR0036606; recordedBy: D.H. Janzen, W. Hallwachs & Calixto Moraga; individualID: DHJPAR0036606; individualCount: 1; sex: male; lifeStage: adult; preparations: pinned; otherCatalogNumbers: ASHYE1517-09, 09-SRNP-30852, BOLD:AAA1929; **Taxon:** scientificName: Hyphantrophaga nigricauda; phylum: Arthropoda; class: Insecta; order: Diptera; family: Tachinidae; genus: Hyphantrophaga; specificEpithet: nigricauda; scientificNameAuthorship: Fleming & Wood, 2017; **Location:** continent: Central America; country: Costa Rica; countryCode: CR; stateProvince: Guanacaste; county: Sector Pitilla; locality: Area de Conservacion Guanacaste; verbatimLocality: Sendero Mismo; verbatimElevation: 680; verbatimLatitude: 10.9876; verbatimLongitude: -85.4197; verbatimCoordinateSystem: Decimal; decimalLatitude: 10.9876; decimalLongitude: -85.4197; **Identification:** identifiedBy: AJ Fleming; dateIdentified: 2017; **Event:** samplingProtocol: Reared from the larva of the Thyrididae, Dysodia Janzen10; verbatimEventDate: 24-Sep-2009; **Record Level:** language: en; institutionCode: CNC; collectionCode: Insects; basisOfRecord: Pinned Specimen
- **Type status:** Paratype. **Occurrence:** occurrenceDetails: http://janzen.sas.upenn.edu; catalogNumber: DHJPAR0042558; recordedBy: D.H. Janzen, W. Hallwachs & Freddy Quesada; individualID: DHJPAR0042558; individualCount: 1; sex: female; lifeStage: adult; preparations: pinned; otherCatalogNumbers: ASHYH316-11, 11-SRNP-31057, BOLD:AAA1929; **Taxon:** scientificName: Hyphantrophaga nigricauda; phylum: Arthropoda; class: Insecta; order: Diptera; family: Tachinidae; genus: Hyphantrophaga; specificEpithet: nigricauda; scientificNameAuthorship: Fleming & Wood, 2017; **Location:** continent: Central America; country: Costa Rica; countryCode: CR; stateProvince: Guanacaste; county: Sector Pitilla; locality: Area de Conservacion Guanacaste; verbatimLocality: Sendero Laguna; verbatimElevation: 680; verbatimLatitude: 10.9888; verbatimLongitude: -85.4234; verbatimCoordinateSystem: Decimal; decimalLatitude: 10.9888; decimalLongitude: -85.4234; **Identification:** identifiedBy: AJ Fleming; dateIdentified: 2017; **Event:** samplingProtocol: Reared from the larva of the Crambidae, Eulepte Solis15; verbatimEventDate: 16-May-2011; **Record Level:** language: en; institutionCode: CNC; collectionCode: Insects; basisOfRecord: Pinned Specimen
- **Type status:** Paratype. **Occurrence:** occurrenceDetails: http://janzen.sas.upenn.edu; catalogNumber: DHJPAR0007289; recordedBy: D.H. Janzen, W. Hallwachs & Lucia Rios; individualID: DHJPAR0007289; individualCount: 1; sex: male; lifeStage: adult; preparations: pinned; otherCatalogNumbers: ASTAT061-06, 05-SRNP-22093, BOLD:AAA1929; **Taxon:** scientificName: Hyphantrophaga nigricauda; phylum: Arthropoda; class: Insecta; order: Diptera; family: Tachinidae; genus: Hyphantrophaga; specificEpithet: nigricauda; scientificNameAuthorship: Fleming & Wood, 2017; **Location:** continent: Central America; country: Costa Rica; countryCode: CR; stateProvince: Guanacaste; county: Sector Del Oro; locality: Area de Conservacion Guanacaste; verbatimLocality: Monte Cristo; verbatimElevation: 525; verbatimLatitude: 11.0137; verbatimLongitude: -85.4253; verbatimCoordinateSystem: Decimal; decimalLatitude: 11.0137; decimalLongitude: -85.4253; **Identification:** identifiedBy: AJ Fleming; dateIdentified: 2017; **Event:** samplingProtocol: Reared from the larva of the Geometridae, Cyclomia disparilis; verbatimEventDate: 01-Jul-2005; **Record Level:** language: en; institutionCode: CNC; collectionCode: Insects; basisOfRecord: Pinned Specimen
- **Type status:** Paratype. **Occurrence:** occurrenceDetails: http://janzen.sas.upenn.edu; catalogNumber: DHJPAR0045600; recordedBy: D.H. Janzen, W. Hallwachs & Freddy Quesada; individualID: DHJPAR0045600; individualCount: 1; sex: male; lifeStage: adult; preparations: pinned; otherCatalogNumbers: ACGAZ789-11, 11-SRNP-31069, BOLD:AAA1929; **Taxon:** scientificName: Hyphantrophaga nigricauda; phylum: Arthropoda; class: Insecta; order: Diptera; family: Tachinidae; genus: Hyphantrophaga; specificEpithet: nigricauda; scientificNameAuthorship: Fleming & Wood, 2017; **Location:** continent: Central America; country: Costa Rica; countryCode: CR; stateProvince: Guanacaste; county: Sector Pitilla; locality: Area de Conservacion Guanacaste; verbatimLocality: Sendero Laguna; verbatimElevation: 680; verbatimLatitude: 10.9888; verbatimLongitude: -85.4234; verbatimCoordinateSystem: Decimal; decimalLatitude: 10.9888; decimalLongitude: -85.4234; **Identification:** identifiedBy: AJ Fleming; dateIdentified: 2017; **Event:** samplingProtocol: Reared from the larva of the Crambidae, Eulepte Solis15; verbatimEventDate: 18-May-2011; **Record Level:** language: en; institutionCode: CNC; collectionCode: Insects; basisOfRecord: Pinned Specimen
- **Type status:** Paratype. **Occurrence:** occurrenceDetails: http://janzen.sas.upenn.edu; catalogNumber: DHJPAR0007292; recordedBy: D.H. Janzen, W. Hallwachs & Calixto Moraga; individualID: DHJPAR0007292; individualCount: 1; sex: female; lifeStage: adult; preparations: pinned; otherCatalogNumbers: ASTAT064-06, 05-SRNP-32272, BOLD:AAA1929; **Taxon:** scientificName: Hyphantrophaga nigricauda; phylum: Arthropoda; class: Insecta; order: Diptera; family: Tachinidae; genus: Hyphantrophaga; specificEpithet: nigricauda; scientificNameAuthorship: Fleming & Wood, 2017; **Location:** continent: Central America; country: Costa Rica; countryCode: CR; stateProvince: Guanacaste; county: Sector Pitilla; locality: Area de Conservacion Guanacaste; verbatimLocality: Pasmompa; verbatimElevation: 440; verbatimLatitude: 11.0193; verbatimLongitude: -85.41; verbatimCoordinateSystem: Decimal; decimalLatitude: 11.0193; decimalLongitude: -85.41; **Identification:** identifiedBy: AJ Fleming; dateIdentified: 2017; **Event:** samplingProtocol: Reared from the larva of the Geometridae, Cyclomia disparilis; verbatimEventDate: 16-Jul-2005; **Record Level:** language: en; institutionCode: CNC; collectionCode: Insects; basisOfRecord: Pinned Specimen
- **Type status:** Paratype. **Occurrence:** occurrenceDetails: http://janzen.sas.upenn.edu; catalogNumber: DHJPAR0037607; recordedBy: D.H. Janzen, W. Hallwachs & Elieth Cantillano; individualID: DHJPAR0037607; individualCount: 1; sex: male; lifeStage: adult; preparations: pinned; otherCatalogNumbers: ASHYC4352-10, 09-SRNP-21421, BOLD:AAA1929; **Taxon:** scientificName: Hyphantrophaga nigricauda; phylum: Arthropoda; class: Insecta; order: Diptera; family: Tachinidae; genus: Hyphantrophaga; specificEpithet: nigricauda; scientificNameAuthorship: Fleming & Wood, 2017; **Location:** continent: Central America; country: Costa Rica; countryCode: CR; stateProvince: Guanacaste; county: Sector Del Oro; locality: Area de Conservacion Guanacaste; verbatimLocality: Puente Mena; verbatimElevation: 280; verbatimLatitude: 11.0456; verbatimLongitude: -85.4574; verbatimCoordinateSystem: Decimal; decimalLatitude: 11.0456; decimalLongitude: -85.4574; **Identification:** identifiedBy: AJ Fleming; dateIdentified: 2017; **Event:** samplingProtocol: Reared from the larva of the Geometridae, Cyclomia disparilis; verbatimEventDate: 23-Jul-2009; **Record Level:** language: en; institutionCode: CNC; collectionCode: Insects; basisOfRecord: Pinned Specimen
- **Type status:** Paratype. **Occurrence:** occurrenceDetails: http://janzen.sas.upenn.edu; catalogNumber: DHJPAR0007313; recordedBy: D.H. Janzen, W. Hallwachs & Elieth Cantillano; individualID: DHJPAR0007313; individualCount: 1; sex: male; lifeStage: adult; preparations: pinned; otherCatalogNumbers: ASTAT085-06, 05-SRNP-22072, BOLD:AAA1929; **Taxon:** scientificName: Hyphantrophaga nigricauda; phylum: Arthropoda; class: Insecta; order: Diptera; family: Tachinidae; genus: Hyphantrophaga; specificEpithet: nigricauda; scientificNameAuthorship: Fleming & Wood, 2017; **Location:** continent: Central America; country: Costa Rica; countryCode: CR; stateProvince: Guanacaste; county: Sector Del Oro; locality: Area de Conservacion Guanacaste; verbatimLocality: Monte Cristo; verbatimElevation: 525; verbatimLatitude: 11.0137; verbatimLongitude: -85.4253; verbatimCoordinateSystem: Decimal; decimalLatitude: 11.0137; decimalLongitude: -85.4253; **Identification:** identifiedBy: AJ Fleming; dateIdentified: 2017; **Event:** samplingProtocol: Reared from the larva of the Geometridae, Cyclomia disparilis; verbatimEventDate: 28-Jun-2005; **Record Level:** language: en; institutionCode: CNC; collectionCode: Insects; basisOfRecord: Pinned Specimen

#### Description

**Male** (Fig. 22). Length: 4–7 mm. **Head** (Fig. 22b): vertex 1/5 of head width; two reclinate upper orbital setae; ocellar setae arising beside anterior ocellus; ocellar triangle gold, concolorous with fronto-orbital plate; fronto-orbital plate gold over 90% of surface, sparsely setulose, setulae not extending below lowest frontal seta; parafacial shiny silver and bare; facial ridge bare; pedicel black, concolorous with postpedicel; arista black, very minutely pubescent, distinctly thickened on basal 1/3–1/4; palpus yellow and haired. **Thorax** (Fig. 22a, c): gold tomentose dorsally, grey tomentose laterally; four prominent dorsal vittae, outermost two broken across suture, innermost pair unbroken across suture, reaching 3rd postsutural dorsocentral seta; postpronotum with 3–4 setae arranged in a triangle; chaetotaxy: acrostichal setae 3:3; dorsocentral setae 3:4; intra-alar setae 2:3; supra-alar setae 2:3; three katepisternal setae, basal seta extremely weak and anterior to suture; lateral scutellar setae subequal in length to apical setae, curving inwards medially; lateral scutellar setae subequal in length to basal setae; subapical setae strongest and longest of marginal scutellar setae, these not strongly divergent; apical scutellar setae short and relatively weak, erect and crossed midway; one pair of discal scutellar setae; scutellum with a darkened crescent across basal 30%, remainder concolorous with scutum. **Legs** (Fig. 22c): black throughout; hind coxa setose. **Wing** (Fig. 22a): pale grey translucent, slightly infuscate, veins dark amber brown; vein R_4+5_ with two setulae at base. **Abdomen** (Fig. 22a, c): ground colour black; mid-dorsal depression on ST1+2 reaching hind margin; abdominal tomentum present as gold bands spanning anterior 50% of T3–T4; T5 gold tomentose with black apex; median marginal setae present on ST1+2–T3; a complete row of marginal setae present on T4; discal setae present on T3–T5; sex patch absent. **Terminalia** (Fig. 22d, e, f): anterior margin of sternite 5 (Fig. 22f) with a slight curved medial depression, posterior edge with a deeply excavated median cleft, smoothly U-shaped; posterior lobes of sternite rounded apically and completely devoid of setae; apical plate shorter than posterior lobes. Unsclerotised "window" anterior to median cleft with central rectangular gap as wide as median cleft, branched on either side, with two thin arms reaching around base of posterior lobes. Cerci (Fig. 22d) in posterior view subrectangular and slightly shorter than surstyli, blunt and squared off towards apex, completely separate medially, diverging at tips; in lateral view (Fig. 22e) with a strong, almost 90 degree bend 1/3 along their length apically; densely setulose almost to apex dorsally, setulose ventrally along basal 2/3 (visible in lateral view). Surstylus in lateral view almost parallel-sided along its length, ending in a slightly downcurved apex, making the structure appear blade-like; surstylus appearing fused with epandrium; when viewed dorsally, surstyli appearing strongly divergent and wide open. Pregonite broad and well-developed, bent basally, apical 2/3 cinched, giving it a petiolate appearance, apically rounded, devoid of setulae. Postgonite elongate, slender, very slightly hooked at tip, subequal in length to pregonite. Distiphallus cone-shaped, apically flared, with a slender median longitudinal sclerotised reinforcement on its posterior surface and a broad, anterolateral, sclerotised acrophallus on each side, joining the plate of opposite side on anterior surface near apex; approximately two times as long as basiphallus.

**Female**. Length: 5–7 mm. As male, differing only by the presence of two pairs of proclinate orbital setae.

#### Diagnosis

*Hyphantrophaga nigricauda* **sp. n.** can be distinguished from all other *Hyphantrophaga* species by the following combination of traits: pedicel and arista concolorous, dark brown/black, four postsutural dorsocentral setae, four thoracic vittae, inner pair solid across suture, hind coxa setose, discal setae present on T3–T5 and T5 gold tomentose with a black apex.

#### Etymology

From the Latin adjective “*nigrum*” meaning black and the Latin noun "*cauda*" meaning tail, in reference to the apically black T5.

#### Distribution

Costa Rica, ACG, Alajuela and Guanacaste Provinces, 280–680 m elevation.

#### Ecology

*Hyphantrophaga nigricauda* **sp. n.** has been reared 22 times from five species of Lepidoptera in the families Geometridae, (*Cyclomia disparilis* Schaus, 1911; Crambidae: *Pleuroptya* Solis02, *Eulepte* Solis15 and *Conchylodes platinalis* (Guenée, 1854)); and Thyrididae, (*Dysodia* Janzen10); in rain forest and dry-rain lowland intergrade.

### Hyphantrophaga osvaldoespinozai

Fleming & Wood sp. n.

urn:lsid:zoobank.org:act:E78E0490-A791-42D4-8874-1D62E818C94E

#### Materials

- **Type status:** Holotype. **Occurrence:** occurrenceDetails: http://janzen.sas.upenn.edu; catalogNumber: DHJPAR0010389; recordedBy: D.H. Janzen, W. Hallwachs & Eilyn Camacho; individualID: DHJPAR0010389; individualCount: 1; sex: male; lifeStage: adult; preparations: pinned; otherCatalogNumbers: ASTAS220-06, 06-SRNP-16192, BOLD:AAD2740; **Taxon:** scientificName: Hyphantrophaga *osvaldoespinozai*; phylum: Arthropoda; class: Insecta; order: Diptera; family: Tachinidae; genus: Hyphantrophaga; specificEpithet: osvaldoespinozai; scientificNameAuthorship: Fleming & Wood, 2017; **Location:** continent: Central America; country: Costa Rica; countryCode: CR; stateProvince: Guanacaste; county: Camino Bahia Hachal; locality: Area de Conservacion Guanacaste; verbatimLocality: Camino Bahia Hachal; verbatimElevation: 5; verbatimLatitude: 10.758; verbatimLongitude: -85.6086; verbatimCoordinateSystem: Decimal; decimalLatitude: 10.758; decimalLongitude: -85.6086; **Identification:** identifiedBy: AJ Fleming; dateIdentified: 2017; **Event:** samplingProtocol: Reared from the larva of the Noctuidae, Diastema morata; verbatimEventDate: 26-Jul-2006; **Record Level:** language: en; institutionCode: CNC; collectionCode: Insects; basisOfRecord: Pinned Specimen
- **Type status:** Paratype. **Occurrence:** occurrenceDetails: http://janzen.sas.upenn.edu; catalogNumber: DHJPAR0007478; recordedBy: D.H. Janzen, W. Hallwachs & gusaneros; individualID: DHJPAR0007478; individualCount: 1; sex: female; lifeStage: adult; preparations: pinned; otherCatalogNumbers: ASTAT250-06, 97-SRNP-10178, BOLD:AAD2740; **Taxon:** scientificName: Hyphantrophaga *osvaldoespinozai*; phylum: Arthropoda; class: Insecta; order: Diptera; family: Tachinidae; genus: Hyphantrophaga; specificEpithet: osvaldoespinozai; scientificNameAuthorship: Fleming & Wood, 2017; **Location:** continent: Central America; country: Costa Rica; countryCode: CR; stateProvince: Guanacaste; county: Presa; locality: Area de Conservacion Guanacaste; verbatimLocality: Presa; verbatimElevation: 180; verbatimLatitude: 10.758; verbatimLongitude: -85.6086; verbatimCoordinateSystem: Decimal; decimalLatitude: 10.758; decimalLongitude: -85.6086; **Identification:** identifiedBy: AJ Fleming; dateIdentified: 2017; **Event:** samplingProtocol: Reared from the larva of the Noctuidae, Diastema tigris; verbatimEventDate: 15-Dec-1997; **Record Level:** language: en; institutionCode: CNC; collectionCode: Insects; basisOfRecord: Pinned Specimen
- **Type status:** Paratype. **Occurrence:** occurrenceDetails: http://janzen.sas.upenn.edu; catalogNumber: DHJPAR0007476; recordedBy: D.H. Janzen, W. Hallwachs & gusaneros; individualID: DHJPAR0007476; individualCount: 1; sex: female; lifeStage: adult; preparations: pinned; otherCatalogNumbers: ASTAT248-06, 97-SRNP-9721, BOLD:AAD2740; **Taxon:** scientificName: Hyphantrophaga *osvaldoespinozai*; phylum: Arthropoda; class: Insecta; order: Diptera; family: Tachinidae; genus: Hyphantrophaga; specificEpithet: osvaldoespinozai; scientificNameAuthorship: Fleming & Wood, 2017; **Location:** continent: Central America; country: Costa Rica; countryCode: CR; stateProvince: Guanacaste; county: Bejuco; locality: Area de Conservacion Guanacaste; verbatimLocality: Bejuco; verbatimElevation: 180; verbatimLatitude: 10.758; verbatimLongitude: -85.6086; verbatimCoordinateSystem: Decimal; decimalLatitude: 10.758; decimalLongitude: -85.6086; **Identification:** identifiedBy: AJ Fleming; dateIdentified: 2017; **Event:** samplingProtocol: Reared from the larva of the Noctuidae, Diastema tigris; verbatimEventDate: 20-Nov-1997; **Record Level:** language: en; institutionCode: CNC; collectionCode: Insects; basisOfRecord: Pinned Specimen
- **Type status:** Paratype. **Occurrence:** occurrenceDetails: http://janzen.sas.upenn.edu; catalogNumber: DHJPAR0010244; recordedBy: D.H. Janzen, W. Hallwachs & Dionis Rivera; individualID: DHJPAR0010244; individualCount: 1; sex: male; lifeStage: adult; preparations: pinned; otherCatalogNumbers: ASTAS075-06, 06-SRNP-16266, BOLD:AAD2740; **Taxon:** scientificName: Hyphantrophaga *osvaldoespinozai*; phylum: Arthropoda; class: Insecta; order: Diptera; family: Tachinidae; genus: Hyphantrophaga; specificEpithet: osvaldoespinozai; scientificNameAuthorship: Fleming & Wood, 2017; **Location:** continent: Central America; country: Costa Rica; countryCode: CR; stateProvince: Guanacaste; county: Estacion Junquillal; locality: Area de Conservacion Guanacaste; verbatimLocality: Estacion Junquillal; verbatimElevation: 5; verbatimLatitude: 10.758; verbatimLongitude: -85.6086; verbatimCoordinateSystem: Decimal; decimalLatitude: 10.758; decimalLongitude: -85.6086; **Identification:** identifiedBy: AJ Fleming; dateIdentified: 2017; **Event:** samplingProtocol: Reared from the larva of the Noctuidae, Diastema morata; verbatimEventDate: 07-Jul-2006; **Record Level:** language: en; institutionCode: CNC; collectionCode: Insects; basisOfRecord: Pinned Specimen
- **Type status:** Paratype. **Occurrence:** occurrenceDetails: http://janzen.sas.upenn.edu; catalogNumber: DHJPAR0007479; recordedBy: D.H. Janzen, W. Hallwachs & Roberto Espinosa; individualID: DHJPAR0007479; individualCount: 1; sex: female; lifeStage: adult; preparations: pinned; otherCatalogNumbers: ASTAT251-06, 04-SRNP-14794, BOLD:AAD2740; **Taxon:** scientificName: Hyphantrophaga *osvaldoespinozai*; phylum: Arthropoda; class: Insecta; order: Diptera; family: Tachinidae; genus: Hyphantrophaga; specificEpithet: osvaldoespinozai; scientificNameAuthorship: Fleming & Wood, 2017; **Location:** continent: Central America; country: Costa Rica; countryCode: CR; stateProvince: Guanacaste; county: Finca Jenny; locality: Area de Conservacion Guanacaste; verbatimLocality: Finca Jenny; verbatimElevation: 205; verbatimLatitude: 10.758; verbatimLongitude: -85.6086; verbatimCoordinateSystem: Decimal; decimalLatitude: 10.758; decimalLongitude: -85.6086; **Identification:** identifiedBy: AJ Fleming; dateIdentified: 2017; **Event:** samplingProtocol: Reared from the larva of the Noctuidae, Diastema tigris; verbatimEventDate: 06-Nov-2004; **Record Level:** language: en; institutionCode: CNC; collectionCode: Insects; basisOfRecord: Pinned Specimen
- **Type status:** Paratype. **Occurrence:** occurrenceDetails: http://janzen.sas.upenn.edu; catalogNumber: DHJPAR0010306; recordedBy: D.H. Janzen, W. Hallwachs & Guillermo Pereira; individualID: DHJPAR0010306; individualCount: 1; sex: male; lifeStage: adult; preparations: pinned; otherCatalogNumbers: ASTAS137-06, 06-SRNP-16246, BOLD:AAD2740; **Taxon:** scientificName: Hyphantrophaga *osvaldoespinozai*; phylum: Arthropoda; class: Insecta; order: Diptera; family: Tachinidae; genus: Hyphantrophaga; specificEpithet: osvaldoespinozai; scientificNameAuthorship: Fleming & Wood, 2017; **Location:** continent: Central America; country: Costa Rica; countryCode: CR; stateProvince: Guanacaste; county: Camino Bahia Hachal; locality: Area de Conservacion Guanacaste; verbatimLocality: Camino Bahia Hachal; verbatimElevation: 5; verbatimLatitude: 10.758; verbatimLongitude: -85.6086; verbatimCoordinateSystem: Decimal; decimalLatitude: 10.758; decimalLongitude: -85.6086; **Identification:** identifiedBy: AJ Fleming; dateIdentified: 2017; **Event:** samplingProtocol: Reared from the larva of the Noctuidae, Diastema morata; verbatimEventDate: 12-Jul-2006; **Record Level:** language: en; institutionCode: CNC; collectionCode: Insects; basisOfRecord: Pinned Specimen
- **Type status:** Paratype. **Occurrence:** occurrenceDetails: http://janzen.sas.upenn.edu; catalogNumber: DHJPAR0007477; recordedBy: D.H. Janzen, W. Hallwachs & gusaneros; individualID: DHJPAR0007477; individualCount: 1; sex: female; lifeStage: adult; preparations: pinned; otherCatalogNumbers: ASTAT249-06, 97-SRNP-9719, BOLD:AAD2740; **Taxon:** scientificName: Hyphantrophaga *osvaldoespinozai*; phylum: Arthropoda; class: Insecta; order: Diptera; family: Tachinidae; genus: Hyphantrophaga; specificEpithet: osvaldoespinozai; scientificNameAuthorship: Fleming & Wood, 2017; **Location:** continent: Central America; country: Costa Rica; countryCode: CR; stateProvince: Guanacaste; county: Bejuco; locality: Area de Conservacion Guanacaste; verbatimLocality: Bejuco; verbatimElevation: 180; verbatimLatitude: 10.758; verbatimLongitude: -85.6086; verbatimCoordinateSystem: Decimal; decimalLatitude: 10.758; decimalLongitude: -85.6086; **Identification:** identifiedBy: AJ Fleming; dateIdentified: 2017; **Event:** samplingProtocol: Reared from the larva of the Noctuidae, Diastema tigris; verbatimEventDate: 14-Nov-1997; **Record Level:** language: en; institutionCode: CNC; collectionCode: Insects; basisOfRecord: Pinned Specimen
- **Type status:** Paratype. **Occurrence:** occurrenceDetails: http://janzen.sas.upenn.edu; catalogNumber: DHJPAR0022959; recordedBy: D.H. Janzen, W. Hallwachs & gusaneros; individualID: DHJPAR0022959; individualCount: 1; sex: female; lifeStage: adult; preparations: pinned; otherCatalogNumbers: ASTAW123-08, 94-SRNP-3920, BOLD:AAD2740; **Taxon:** scientificName: Hyphantrophaga *osvaldoespinozai*; phylum: Arthropoda; class: Insecta; order: Diptera; family: Tachinidae; genus: Hyphantrophaga; specificEpithet: osvaldoespinozai; scientificNameAuthorship: Fleming & Wood, 2017; **Location:** continent: Central America; country: Costa Rica; countryCode: CR; stateProvince: Guanacaste; county: Tanquetas; locality: Area de Conservacion Guanacaste; verbatimLocality: Tanquetas; verbatimElevation: 295; verbatimLatitude: 10.758; verbatimLongitude: -85.6086; verbatimCoordinateSystem: Decimal; decimalLatitude: 10.758; decimalLongitude: -85.6086; **Identification:** identifiedBy: AJ Fleming; dateIdentified: 2017; **Event:** samplingProtocol: Reared from the larva of the Noctuidae, Diastema tigris; verbatimEventDate: 07-Aug-1994; **Record Level:** language: en; institutionCode: CNC; collectionCode: Insects; basisOfRecord: Pinned Specimen
- **Type status:** Paratype. **Occurrence:** occurrenceDetails: http://janzen.sas.upenn.edu; catalogNumber: DHJPAR0007274; recordedBy: D.H. Janzen, W. Hallwachs & gusaneros; individualID: DHJPAR0007274; individualCount: 1; sex: female; lifeStage: adult; preparations: pinned; otherCatalogNumbers: ASTAT046-06, 87-SRNP-654,; **Taxon:** scientificName: Hyphantrophaga *osvaldoespinozai*; phylum: Arthropoda; class: Insecta; order: Diptera; family: Tachinidae; genus: Hyphantrophaga; specificEpithet: osvaldoespinozai; scientificNameAuthorship: Fleming & Wood, 2017; **Location:** continent: Central America; country: Costa Rica; countryCode: CR; stateProvince: Guanacaste; county: Bosque San Emilio; locality: Area de Conservacion Guanacaste; verbatimLocality: Bosque San Emilio; verbatimElevation: 300; verbatimLatitude: 10.758; verbatimLongitude: -85.6086; verbatimCoordinateSystem: Decimal; decimalLatitude: 10.758; decimalLongitude: -85.6086; **Identification:** identifiedBy: AJ Fleming; dateIdentified: 2017; **Event:** samplingProtocol: Reared from the larva of the Noctuidae, Diastema tigris; verbatimEventDate: 10-Nov-1987; **Record Level:** language: en; institutionCode: CNC; collectionCode: Insects; basisOfRecord: Pinned Specimen

#### Description

**Male** (Fig. 23). Length: 6–7 mm. **Head** (Fig. 23b): vertex 1/5 of head width; one pair of reclinate upper orbital setae; ocellar setae arising behind anterior ocellus; ocellar triangle silver (slight gold tinge present but overall concolorous with fronto-orbital plate); fronto-orbital plate shiny silver and sparsely setulose, setulae not extending below lowest frontal seta; parafacial silver and bare; facial ridge bare; eye densely haired; pedicel brownish-black with some orange, concolorous with postpedicel; arista brown, very minutely pubescent, distinctly thickened on basal 1/3–1/4; palpus yellow, slender and haired apically. **Thorax** (Fig. 23a, c): dull brassy-grey tomentose dorsally, grey tomentose laterally; densely covered in black setulae along anterior and lateral surfaces, dorsally with dense dark setulae interspersed amongst macrosetae; four prominent dorsal vittae, outermost two broken across suture, innermost pair unbroken across suture, extending to 2nd postsutural dorsocentral seta; postpronotum with three setae arranged in a triangle; chaetotaxy: acrostichal setae 3:3; dorsocentral setae 3:4; intra-alar setae 2:3; supra-alar setae 2:3; 2–3 katepisternal setae, basal seta, when present, extremely weak and anterior to suture; lateral scutellar setae less than 1/2 as long as subapical setae, curving inwards medially, as long as subapical setae; apical scutellar setae subequal in length to basal scutellar setae crossed apically; one pair of discal scutellar setae more widely set than apical setae; scutellum concolorous with scutum. **Legs** (Fig. 23c): dark reddish-brown to black in ground colour; fore femur with dense silver tomentum on posterodorsal surface; hind coxa setose. **Wing** (Fig. 23a, c): pale translucent, hyaline, not distinctly infuscate; vein R_4+5_ with only 2–3 setulae at base. **Abdomen** (Fig. 23a, c): ground colour black; mid-dorsal depression on ST1+2 reaching hind margin; median marginal setae on ST1+2 weak to absent, present on T3; a complete row of marginal setae present on T4; discal setae present on T3–T5, although these can at times appear weak, they are still distinct from abdominal setulae; sex patch absent; distinct brassy-grey tomentum covering all but posterior margin of T3; T3–T4 with darkened patches dorsolaterally, these more concolorous with ST1+2; T5 with brassy-grey tomentum throughout. **Terminalia** (Fig. 23d, e, f): sternite 5 (Fig. 23f) with a deeply excavated median cleft, smoothly U-shaped, with two smaller lobes inside median cleft. Lateral lobes of sternite pointed apically, with 3–4 strong setae surrounded by many shorter, weaker setulae. Anterior plate of sternite 5 from subequal to slightly shorter than apical lobes, unsclerotised window narrow and rectangular, 2X as wide as median cleft. Cerci in posterior view (Fig. 23d) subrectangular and slightly shorter than surstyli, blunt and squared at apex, completely separate medially, slightly divergent apically; in lateral view with a strong downward curve in apical 1/3, slightly lobed at tip; densely setulose along basal 2/3 dorsally, setulose ventrally along entire length (visible in lateral view). Surstylus in lateral view (Fig. 23e) almost parallel sided along its length, ending in a slightly downcurved apex, making the structure appear blade-like; surstylus slightly fused with epandrium; when viewed dorsally, surstyli appearing to point outwards, not strongly convergent. Pregonite short, not well-developed, 0.3 times as long as distiphallus, bare and squared. Postgonite slightly narrow, 1/3 as wide as pregonite, sharply pointed and curved at apex. Distiphallus sail-shaped, apically flared, with a slender median longitudinal sclerotised reinforcement on its posterior surface and a broad, anterolateral, sclerotised acrophallus on each side, joining the plate of opposite side on anterior surface near apex.

**Female**. Length: 6–8 mm. As male, differing only by the presence of two pairs of proclinate orbital setae.

#### Diagnosis

*Hyphantrophaga osvaldoespinozai* **sp. n.** can be distinguished from all other *Hyphantrophaga* species by the following combination of traits: pedicel concolorous with postpedicel, three katepisternal setae, hind coxa setose, tomentum on T5 matching rest of abdominal tomentum, median marginal setae absent from ST1+2 and discal setae present on T3–T5 (can be weak but distinct).

#### Etymology

*Hyphantrophaga osvaldoespinozai* **sp. n.** is named in recognition of Osvaldo Espinoza Obando's dedication and work in finding and rearing the ACG caterpillars that contained tachinid larvae.

#### Distribution

Costa Rica, ACG, Guanacaste Province, 5–300 m elevation.

#### Ecology

*Hyphantrophaga osvaldoespinozai* **sp. n.** has been reared nine times from two species of Lepidoptera in the family Noctuidae, *Diastema tigris* Guenée, 1852 *and Diastema morata* Schaus, 1894, in dry forest.

### Hyphantrophaga pabloumanai

Fleming & Wood sp. n.

urn:lsid:zoobank.org:act:95D2B9E9-61E4-41E8-B8DD-A7052C5E741B

#### Materials

- **Type status:** Holotype. **Occurrence:** occurrenceDetails: http://janzen.sas.upenn.edu; catalogNumber: DHJPAR0007490; recordedBy: D.H. Janzen, W. Hallwachs & Ruth Franco; individualID: DHJPAR0007490; individualCount: 1; sex: male; lifeStage: adult; preparations: pinned; otherCatalogNumbers: ASTAT262-06, 03-SRNP-14280,; **Taxon:** scientificName: Hyphantrophaga pabloumanai; phylum: Arthropoda; class: Insecta; order: Diptera; family: Tachinidae; genus: Hyphantrophaga; specificEpithet: pabloumanai; scientificNameAuthorship: Fleming & Wood, 2017; **Location:** continent: Central America; country: Costa Rica; countryCode: CR; stateProvince: Guanacaste; county: Sector Santa Rosa; locality: Area de Conservacion Guanacaste; verbatimLocality: Area Administrativa; verbatimElevation: 295; verbatimLatitude: 10.8376; verbatimLongitude: -85.6187; verbatimCoordinateSystem: Decimal; decimalLatitude: 10.8376; decimalLongitude: -85.6187; **Identification:** identifiedBy: AJ Fleming; dateIdentified: 2017; **Event:** samplingProtocol: Reared from the larva of the Apatelodidae, Colabata lybia; verbatimEventDate: 05-Oct-2003; **Record Level:** language: en; institutionCode: CNC; collectionCode: Insects; basisOfRecord: Pinned Specimen

#### Description

**Male** (Fig. 24). Length: 6–7 mm. **Head** (Fig. 24b): vertex 1/4 of head width; two pairs of reclinate upper orbital setae; ocellar setae arising beside to slightly behind anterior ocellus; ocellar triangle pale brassy-silver (slight gold tinge present but overall concolorous with fronto-orbital plate); fronto-orbital plate pale brassy-silver over 75%, changing to brilliant silver adjacent to parafacial, densely setulose, setulae not extending below lowest frontal seta; parafacial silver and bare; facial ridge bare; eye densely haired; pedicel orange, lighter than dark brown/black postpedicel; arista brown, very minutely pubescent, distinctly thickened on basal 1/3–1/4; palpus yellow/orange, slender and haired apically. **Thorax** (Fig. 24a, c): dull brassy-grey tomentose dorsally, grey tomentose laterally; densely covered in black setulae along anterior and lateral surfaces, dorsally with dense dark setulae interspersed amongst setae; four prominent dorsal vittae, outermost two broken across suture, innermost pair unbroken across suture, extending to 2nd postsutural dorsocentral seta; postpronotum with 4–5 setae arranged in a triangle; chaetotaxy: acrostichal setae 3:3; dorsocentral setae 3:4; intra-alar setae 2:3; supra-alar setae 2:3; three katepisternal setae, basal seta weak and anterior to suture; lateral scutellar setae less than 1/2 as long as subapical setae; basal scutellar setae curving inwards medially, as long as subapical setae; apical scutellar setae short, crossed apically; one pair of discal scutellar setae more widely set than apical setae; scutellum concolorous with scutum. **Legs** (Fig. 24c): dark reddish-brown to black in ground colour; fore femur with dense silver tomentum on posterodorsal surface; hind coxa bare. **Wing** (Fig. 24a, c): pale translucent, hyaline, not distinctly infuscate; vein R_4+5_ with only two setulae at base. **Abdomen** (Fig. 24a, c): ground colour reddish-brown; mid-dorsal depression on ST1+2 reaching hind margin; median marginal setae present on ST1+2–T3; a complete row of marginal setae present on T4; discal setae only T5; sex patch present on T4–T5; distinct tomentose bands along anterior 2/3 of T3 and T4, broken medially by a dorsocentral stripe; T5 with silver tomentum over its entire dorsal surface; T3–T4 with darkened patches dorsolaterally, these more concolorous with ST1+2. **Terminalia**: not examined.

**Female**. Not known at this time.

#### Diagnosis

*Hyphantrophaga pabloumanai* **sp. n.** can be distinguished from all other *Hyphantrophaga* species by the following combination of traits: fronto-orbital plate silver tomentose, pedicel orange, four postsutural dorsocentral setae, legs reddish-black, hind coxa bare, median marginal setae present on ST1+2, discal setae only on T5.

#### Etymology

*Hyphantrophaga pabloumanai* **sp. n.** is named in recognition of Pablo José Umaña Calderon's dedication and work in finding and rearing the ACG caterpillars that contained tachinid larvae.

#### Distribution

Costa Rica, ACG, Guanacaste province, 295 m elevation.

#### Ecology

*Hyphantrophaga pabloumanai* **sp. n.** has been reared once from one species of Lepidoptera in the family Apatelodidae, *Colabata lybia* Druce, 1898, in dry forest.

### Hyphantrophaga similis

Fleming & Wood sp. n.

urn:lsid:zoobank.org:act:535A05E1-BDB4-4D26-A724-36B865E3ADDE

#### Materials

- **Type status:** Holotype. **Occurrence:** occurrenceDetails: http://janzen.sas.upenn.edu; catalogNumber: DHJPAR0007325; recordedBy: D.H. Janzen, W. Hallwachs & Jose Perez; individualID: DHJPAR0007325; individualCount: 1; sex: male; lifeStage: adult; preparations: pinned; otherCatalogNumbers: ASTAT097-06, 03-SRNP-11568, BOLD:AAE1435; **Taxon:** scientificName: Hyphantrophaga similis; phylum: Arthropoda; class: Insecta; order: Diptera; family: Tachinidae; genus: Hyphantrophaga; specificEpithet: similis; scientificNameAuthorship: Fleming & Wood, 2017; **Location:** continent: Central America; country: Costa Rica; countryCode: CR; stateProvince: Alajuela; county: Sector Rincon Rain Forest; locality: Area de Conservacion Guanacaste; verbatimLocality: Sendero Anonas; verbatimElevation: 405; verbatimLatitude: 10.9053; verbatimLongitude: -85.2788; verbatimCoordinateSystem: Decimal; decimalLatitude: 10.9053; decimalLongitude: -85.2788; **Identification:** identifiedBy: AJ Fleming; dateIdentified: 2017; **Event:** samplingProtocol: Reared from the larva of the Erebidae, Letis buteoDHJ02; verbatimEventDate: 23-Aug-2003; **Record Level:** language: en; institutionCode: CNC; collectionCode: Insects; basisOfRecord: Pinned Specimen
- **Type status:** Paratype. **Occurrence:** occurrenceDetails: http://janzen.sas.upenn.edu; catalogNumber: DHJPAR0007327; recordedBy: D.H. Janzen, W. Hallwachs & Freyci Vargas; individualID: DHJPAR0007327; individualCount: 1; sex: female; lifeStage: adult; preparations: pinned; otherCatalogNumbers: ASTAT099-06, 00-SRNP-20556, BOLD:AAE1435; **Taxon:** scientificName: Hyphantrophaga similis; phylum: Arthropoda; class: Insecta; order: Diptera; family: Tachinidae; genus: Hyphantrophaga; specificEpithet: similis; scientificNameAuthorship: Fleming & Wood, 2017; **Location:** continent: Central America; country: Costa Rica; countryCode: CR; stateProvince: Alajuela; county: Sector Rincon Rain Forest; locality: Area de Conservacion Guanacaste; verbatimLocality: Sendero Rincon; verbatimElevation: 430; verbatimLatitude: 10.8962; verbatimLongitude: -85.2777; decimalLatitude: 10.8962; decimalLongitude: -85.2777; **Identification:** identifiedBy: M. Wood; dateIdentified: 2017; **Event:** samplingProtocol: Reared from the larva of the Erebidae, Letis buteoDHJ01; verbatimEventDate: 28-Nov-2000; **Record Level:** language: en; institutionCode: CNC; collectionCode: Insects; basisOfRecord: Pinned Specimen
- **Type status:** Paratype. **Occurrence:** occurrenceDetails: http://janzen.sas.upenn.edu; catalogNumber: DHJPAR0007328; recordedBy: D.H. Janzen, W. Hallwachs & Freyci Vargas; individualID: DHJPAR0007328; individualCount: 1; sex: male; lifeStage: adult; preparations: pinned; otherCatalogNumbers: ASTAT100-06, 00-SRNP-20740, BOLD:AAE1435; **Taxon:** scientificName: Hyphantrophaga similis; phylum: Arthropoda; class: Insecta; order: Diptera; family: Tachinidae; genus: Hyphantrophaga; specificEpithet: similis; scientificNameAuthorship: Fleming & Wood, 2017; **Location:** continent: Central America; country: Costa Rica; countryCode: CR; stateProvince: Alajuela; county: Sector Rincon Rain Forest; locality: Area de Conservacion Guanacaste; verbatimLocality: Camino Rio Francia; verbatimElevation: 410; verbatimLatitude: 10.9043; verbatimLongitude: -85.2865; verbatimCoordinateSystem: Decimal; decimalLatitude: 10.9043; decimalLongitude: -85.2865; **Identification:** identifiedBy: AJ Fleming; dateIdentified: 2017; **Event:** samplingProtocol: Reared from the larva of the Erebidae, Letis buteoDHJ01; verbatimEventDate: 11-Dec-2000; **Record Level:** language: en; institutionCode: CNC; collectionCode: Insects; basisOfRecord: Pinned Specimen
- **Type status:** Paratype. **Occurrence:** occurrenceDetails: http://janzen.sas.upenn.edu; catalogNumber: DHJPAR0007329; recordedBy: D.H. Janzen, W. Hallwachs & Elieth Cantillano; individualID: DHJPAR0007329; individualCount: 1; sex: male; lifeStage: adult; preparations: pinned; otherCatalogNumbers: ASTAT101-06, 05-SRNP-22366, BOLD:AAE1435; **Taxon:** scientificName: Hyphantrophaga similis; phylum: Arthropoda; class: Insecta; order: Diptera; family: Tachinidae; genus: Hyphantrophaga; specificEpithet: similis; scientificNameAuthorship: Fleming & Wood, 2017; **Location:** continent: Central America; country: Costa Rica; countryCode: CR; stateProvince: Guanacaste; county: Sector Del Oro; locality: Area de Conservacion Guanacaste; verbatimLocality: Margarita; verbatimElevation: 380; verbatimLatitude: 11.0323; verbatimLongitude: -85.4395; verbatimCoordinateSystem: Decimal; decimalLatitude: 11.0323; decimalLongitude: -85.4395; **Identification:** identifiedBy: AJ Fleming; dateIdentified: 2017; **Event:** samplingProtocol: Reared from the larva of the Erebidae, Letis mycerina; verbatimEventDate: 28-Jul-2005; **Record Level:** language: en; institutionCode: CNC; collectionCode: Insects; basisOfRecord: Pinned Specimen
- **Type status:** Paratype. **Occurrence:** occurrenceDetails: http://janzen.sas.upenn.edu; catalogNumber: DHJPAR0045663; recordedBy: D.H. Janzen, W. Hallwachs & Duvalier Briceno; individualID: DHJPAR0045663; individualCount: 1; sex: male; lifeStage: adult; preparations: pinned; otherCatalogNumbers: ACGAZ852-11, 11-SRNP-65874, BOLD:AAE1435; **Taxon:** scientificName: Hyphantrophaga similis; phylum: Arthropoda; class: Insecta; order: Diptera; family: Tachinidae; genus: Hyphantrophaga; specificEpithet: similis; scientificNameAuthorship: Fleming & Wood, 2017; **Location:** continent: Central America; country: Costa Rica; countryCode: CR; stateProvince: Alajuela; county: Brasilia; locality: Area de Conservacion Guanacaste; verbatimLocality: Gallinazo; verbatimElevation: 360; verbatimLatitude: 11.0183; verbatimLongitude: -85.372; verbatimCoordinateSystem: Decimal; decimalLatitude: 11.0183; decimalLongitude: -85.372; **Identification:** identifiedBy: AJ Fleming; dateIdentified: 2017; **Event:** samplingProtocol: Reared from the larva of the Erebidae, Letis buteoDHJ01; verbatimEventDate: 25-Sep-2011; **Record Level:** language: en; institutionCode: CNC; collectionCode: Insects; basisOfRecord: Pinned Specimen
- **Type status:** Paratype. **Occurrence:** occurrenceDetails: http://janzen.sas.upenn.edu; catalogNumber: DHJPAR0007326; recordedBy: D.H. Janzen, W. Hallwachs & Freyci Vargas; individualID: DHJPAR0007326; individualCount: 1; sex: female; lifeStage: adult; preparations: pinned; otherCatalogNumbers: ASTAT098-06, 00-SRNP-14698, BOLD:AAE1435; **Taxon:** scientificName: Hyphantrophaga similis; phylum: Arthropoda; class: Insecta; order: Diptera; family: Tachinidae; genus: Hyphantrophaga; specificEpithet: similis; scientificNameAuthorship: Fleming & Wood, 2017; **Location:** continent: Central America; country: Costa Rica; countryCode: CR; stateProvince: Alajuela; county: Sector Rincon Rain Forest; locality: Area de Conservacion Guanacaste; verbatimLocality: Sendero Rincon; verbatimElevation: 430; verbatimLatitude: 10.8962; verbatimLongitude: -85.2777; verbatimCoordinateSystem: Decimal; decimalLatitude: 10.8962; decimalLongitude: -85.2777; **Identification:** identifiedBy: AJ Fleming; dateIdentified: 2017; **Event:** samplingProtocol: Reared from the larva of the Erebidae, Letis buteoDHJ01; verbatimEventDate: 23-Oct-2000; **Record Level:** language: en; institutionCode: CNC; collectionCode: Insects; basisOfRecord: Pinned Specimen

#### Description

**Male** (Fig. 25). Length: 8–12 mm. **Head** (Fig. 25b): vertex 1/4 of head width; two reclinate upper orbital setae; ocellar setae arising beside anterior ocellus; ocellar triangle silver, concolorous with fronto-orbital plate; fronto-orbital plate silver with a slight gold tinge over frontal setae, setulose, setulae not extending below lowest frontal seta; frontal setae extending up to base of postpedicel; parafacial silver and bare; facial ridge bare; eye densely haired; pedicel black, concolorous with postpedicel; arista brown, very minutely pubescent, distinctly thickened on basal 1/3–1/4. **Thorax** (Fig. 25a, c): dull brassy-grey tomentose dorsally, grey tomentose laterally; four prominent dorsal vittae, outermost two broken across suture, innermost pair unbroken across suture, extending to 3rd postsutural dorsocentral seta; postpronotum with five setae arranged in a triangle; chaetotaxy: acrostichal setae 3:3; dorsocentral setae 4:4; intra-alar setae 3:3; supra-alar setae 2:3; two katepisternal setae; lateral scutellar setae shorter than subapical setae, curving inwards medially; apical scutellar setae subequal in length to basal scutellar setae crossed apically; one pair of discal scutellar setae; scutellum black over basal 20%, remainder concolorous with scutum. **Legs** (Fig. 25c): black in ground colour; fore femur with dense silver tomentum on posterodorsal surface; hind coxa bare. **Wing** (Fig. 25a): pale translucent, hyaline, not distinctly infuscate; vein R_4+5_ with only 2–3 setulae at base. **Abdomen** (Fig. 25a, c): ground colour black; mid-dorsal depression on ST1+2 almost reaching hind margin; median marginal setae present on T3; a complete row of marginal setae present on T4; discal setae only on T5; sex patch covering ventral surfaces of T4–T5 and posterior 1/3 of T3; distinct tomentose bands along anterior edge of T3 and T4, broken medially by a dorsocentral stripe; T5 with silver tomentum covering anterior half. **Terminalia** (Fig. 25d, e, f): sternite 5 (Fig. 25f) with a deeply excavated median cleft, smoothly U-shaped, margins covered in dense tomentum. Lateral lobes of sternite rounded apically, with 2–3 strong setae surrounded by many shorter, weaker setulae. Anterior plate of sternite 5 from subequal to slightly longer than apical lobes; unsclerotised "window" ovoid to slightly rectangular, as wide as median cleft. Cerci in posterior view (Fig. 25d) triangular and slightly shorter than surstyli, blunt and rounded at apex, completely separate medially, appearing slightly divergent; in lateral view with a strong downward curve in apical 1/3; densely setulose along basal 2/3 dorsally, setulose ventrally along entire length (visible in lateral view). Surstylus in lateral view (Fig. 25e) almost parallel-sided along its length, ending in a slightly downcurved apex, making the structure appear blade-like; when viewed dorsally, surstyli appearing to point outwards, not strongly convergent. Pregonite short, not well-developed, 1/2 as long as distiphallus, bare and squared-off apically. Postgonite slightly narrow, 1/3 as wide as pregonite, sharply pointed and curved at apex. Distiphallus vaguely cone-shaped (could be seen as rectangular), only weakly flaring apically with a slender arrow-shaped median longitudinal sclerotised reinforcement on its posterior surface and a broad, anterolateral, apically clubbed sclerotised acrophallus on each side, joining the plate of opposite side on anterior surface near apex.

**Female**. Length: 8–11 mm. As male, differing only by the presence of two pairs of proclinate orbital setae.

#### Diagnosis

*Hyphantrophaga similis* **sp. n.** can be distinguished from all other *Hyphantrophaga* species by the following combination of traits: parafacial, fronto-orbital plate and ocellar triangle all silver, frontal setae extending up to base of postpedicel. Differs from its most similar congener *H. niveifacies* by the colour of the wings and the presence of only two katepisternal setae.

#### Etymology

From the Latin adjective “*similis*”, meaning similar or resembling, in reference to its similarity to the Brazilian species *H. niveifacies* (Macquart, 1851).

#### Distribution

Costa Rica, ACG, Alajuela and Guanacaste Provinces, 320–430 m elevation.

#### Ecology

*Hyphantrophaga similis* **sp. n.** has been reared seven times from three species of Lepidoptera in the family Erebidae, *Letis buteo*DHJ01, *Letis buteo*DHJ02 and *Letis mycerina* (Cramer, 1777), in rain forest and dry-rain lowland intergrade.

### Hyphantrophaga virilis

(Aldrich & Webber, 1924)

#### Materials

- **Type status:** Other material. **Occurrence:** occurrenceDetails: http://janzen.sas.upenn.edu; catalogNumber: DHJPAR0003295; recordedBy: D.H. Janzen, W. Hallwachs & Mariano Pereira; individualID: DHJPAR0003295; individualCount: 1; sex: male; lifeStage: adult; preparations: pinned; otherCatalogNumbers: ASTA202-05, 05-SRNP-56212, BOLD:AAA1577; **Taxon:** scientificName: Hyphantrophaga virilis; phylum: Arthropoda; class: Insecta; order: Diptera; family: Tachinidae; genus: Hyphantrophaga; specificEpithet: virilis; scientificNameAuthorship: (Aldrich & Webber, 1924); **Location:** continent: Central America; country: Costa Rica; countryCode: CR; stateProvince: Guanacaste; county: Sector Mundo Nuevo; locality: Area de Conservacion Guanacaste; verbatimLocality: Vado Miramonte; verbatimElevation: 305; verbatimLatitude: 10.7718; verbatimLongitude: -85.434; verbatimCoordinateSystem: Decimal; decimalLatitude: 10.7718; decimalLongitude: -85.434; **Identification:** identifiedBy: AJ Fleming; dateIdentified: 2017; **Event:** samplingProtocol: Reared from the larva of the Hesperiidae, Saliana fusta; verbatimEventDate: 02-Jul-2005; **Record Level:** language: en; institutionCode: CNC; collectionCode: Insects; basisOfRecord: Pinned Specimen
- **Type status:** Other material. **Occurrence:** occurrenceDetails: http://janzen.sas.upenn.edu; catalogNumber: DHJPAR0003299; recordedBy: D.H. Janzen, W. Hallwachs & Roster Moraga; individualID: DHJPAR0003299; individualCount: 1; sex: male; lifeStage: adult; preparations: pinned; otherCatalogNumbers: ASTA206-05, 04-SRNP-26847, BOLD:AAA1577; **Taxon:** scientificName: Hyphantrophaga virilis; phylum: Arthropoda; class: Insecta; order: Diptera; family: Tachinidae; genus: Hyphantrophaga; specificEpithet: virilis; scientificNameAuthorship: (Aldrich & Webber, 1924); **Location:** continent: Central America; country: Costa Rica; countryCode: CR; stateProvince: Guanacaste; county: Sector Del Oro; locality: Area de Conservacion Guanacaste; verbatimLocality: Bosque Aguirre; verbatimElevation: 620; verbatimLatitude: 11.0006; verbatimLongitude: -85.438; verbatimCoordinateSystem: Decimal; decimalLatitude: 11.0006; decimalLongitude: -85.438; **Identification:** identifiedBy: AJ Fleming; dateIdentified: 2017; **Event:** samplingProtocol: Reared from the larva of the Erebidae, Antiblemma Poole05; verbatimEventDate: 24-Dec-2004; **Record Level:** language: en; institutionCode: CNC; collectionCode: Insects; basisOfRecord: Pinned Specimen
- **Type status:** Other material. **Occurrence:** occurrenceDetails: http://janzen.sas.upenn.edu; catalogNumber: DHJPAR0003301; recordedBy: D.H. Janzen, W. Hallwachs & Freddy Quesada; individualID: DHJPAR0003301; individualCount: 1; sex: male; lifeStage: adult; preparations: pinned; otherCatalogNumbers: ASTA208-05, 04-SRNP-15774, BOLD:AAA1577; **Taxon:** scientificName: Hyphantrophaga virilis; phylum: Arthropoda; class: Insecta; order: Diptera; family: Tachinidae; genus: Hyphantrophaga; specificEpithet: virilis; scientificNameAuthorship: (Aldrich & Webber, 1924); **Location:** continent: Central America; country: Costa Rica; countryCode: CR; stateProvince: Guanacaste; county: Sector Santa Rosa; locality: Area de Conservacion Guanacaste; verbatimLocality: Area Administrativa; verbatimElevation: 295; verbatimLatitude: 10.8376; verbatimLongitude: -85.6187; verbatimCoordinateSystem: Decimal; decimalLatitude: 10.8376; decimalLongitude: -85.6187; **Identification:** identifiedBy: AJ Fleming; dateIdentified: 2017; **Event:** samplingProtocol: Reared from the larva of the Megalopygidae, Norape nigrovenosa; verbatimEventDate: 20-Dec-2004; **Record Level:** language: en; institutionCode: CNC; collectionCode: Insects; basisOfRecord: Pinned Specimen
- **Type status:** Other material. **Occurrence:** occurrenceDetails: http://janzen.sas.upenn.edu; catalogNumber: DHJPAR0003302; recordedBy: D.H. Janzen, W. Hallwachs & Noemi Espinosa; individualID: DHJPAR0003302; individualCount: 1; sex: male; lifeStage: adult; preparations: pinned; otherCatalogNumbers: ASTA209-05, 04-SRNP-15262, BOLD:AAA1577; **Taxon:** scientificName: Hyphantrophaga virilis; phylum: Arthropoda; class: Insecta; order: Diptera; family: Tachinidae; genus: Hyphantrophaga; specificEpithet: virilis; scientificNameAuthorship: (Aldrich & Webber, 1924); **Location:** continent: Central America; country: Costa Rica; countryCode: CR; stateProvince: Guanacaste; county: Sector Santa Rosa; locality: Area de Conservacion Guanacaste; verbatimLocality: Bosque San Emilio; verbatimElevation: 300; verbatimLatitude: 10.8439; verbatimLongitude: -85.6138; verbatimCoordinateSystem: Decimal; decimalLatitude: 10.8439; decimalLongitude: -85.6138; **Identification:** identifiedBy: AJ Fleming; dateIdentified: 2017; **Event:** samplingProtocol: Reared from the larva of the Hesperiidae, Mysoria ambigua; verbatimEventDate: 27-Nov-2004; **Record Level:** language: en; institutionCode: CNC; collectionCode: Insects; basisOfRecord: Pinned Specimen
- **Type status:** Other material. **Occurrence:** occurrenceDetails: http://janzen.sas.upenn.edu; catalogNumber: DHJPAR0003303; recordedBy: D.H. Janzen, W. Hallwachs & Ruth Franco; individualID: DHJPAR0003303; individualCount: 1; sex: male; lifeStage: adult; preparations: pinned; otherCatalogNumbers: ASTA210-05, 04-SRNP-15402, BOLD:AAA1577; **Taxon:** scientificName: Hyphantrophaga virilis; phylum: Arthropoda; class: Insecta; order: Diptera; family: Tachinidae; genus: Hyphantrophaga; specificEpithet: virilis; scientificNameAuthorship: (Aldrich & Webber, 1924); **Location:** continent: Central America; country: Costa Rica; countryCode: CR; stateProvince: Guanacaste; county: Potrerillos; locality: Area de Conservacion Guanacaste; verbatimLocality: Rio Azufrado; verbatimElevation: 95; verbatimLatitude: 10.8122; verbatimLongitude: -85.5444; verbatimCoordinateSystem: Decimal; decimalLatitude: 10.8122; decimalLongitude: -85.5444; **Identification:** identifiedBy: AJ Fleming; dateIdentified: 2017; **Event:** samplingProtocol: Reared from the larva of the Notodontidae, Calledema plusia; verbatimEventDate: 07-Dec-2004; **Record Level:** language: en; institutionCode: CNC; collectionCode: Insects; basisOfRecord: Pinned Specimen
- **Type status:** Other material. **Occurrence:** occurrenceDetails: http://janzen.sas.upenn.edu; catalogNumber: DHJPAR0003309; recordedBy: D.H. Janzen, W. Hallwachs & Ruth Franco; individualID: DHJPAR0003309; individualCount: 1; sex: male; lifeStage: adult; preparations: pinned; otherCatalogNumbers: ASTA216-05, 04-SRNP-14653, BOLD:AAA1577; **Taxon:** scientificName: Hyphantrophaga virilis; phylum: Arthropoda; class: Insecta; order: Diptera; family: Tachinidae; genus: Hyphantrophaga; specificEpithet: virilis; scientificNameAuthorship: (Aldrich & Webber, 1924); **Location:** continent: Central America; country: Costa Rica; countryCode: CR; stateProvince: Guanacaste; county: Sector Santa Rosa; locality: Area de Conservacion Guanacaste; verbatimLocality: Bosque Humedo; verbatimElevation: 290; verbatimLatitude: 10.8514; verbatimLongitude: -85.608; verbatimCoordinateSystem: Decimal; decimalLatitude: 10.8514; decimalLongitude: -85.608; **Identification:** identifiedBy: AJ Fleming; dateIdentified: 2017; **Event:** samplingProtocol: Reared from the larva of the Hesperiidae, Nascus Burns01; verbatimEventDate: 18-Nov-2004; **Record Level:** language: en; institutionCode: CNC; collectionCode: Insects; basisOfRecord: Pinned Specimen
- **Type status:** Other material. **Occurrence:** occurrenceDetails: http://janzen.sas.upenn.edu; catalogNumber: DHJPAR0003310; recordedBy: D.H. Janzen, W. Hallwachs & Harry Ramirez; individualID: DHJPAR0003310; individualCount: 1; sex: male; lifeStage: adult; preparations: pinned; otherCatalogNumbers: ASTA217-05, 04-SRNP-49374, BOLD:AAA1577; **Taxon:** scientificName: Hyphantrophaga virilis; phylum: Arthropoda; class: Insecta; order: Diptera; family: Tachinidae; genus: Hyphantrophaga; specificEpithet: virilis; scientificNameAuthorship: (Aldrich & Webber, 1924); **Location:** continent: Central America; country: Costa Rica; countryCode: CR; stateProvince: Guanacaste; county: Sector Cacao; locality: Area de Conservacion Guanacaste; verbatimLocality: Sendero Guayabal; verbatimElevation: 500; verbatimLatitude: 10.8857; verbatimLongitude: -85.4818; verbatimCoordinateSystem: Decimal; decimalLatitude: 10.8857; decimalLongitude: -85.4818; **Identification:** identifiedBy: AJ Fleming; dateIdentified: 2017; **Event:** samplingProtocol: Reared from the larva of the Erebidae, Pachydota saduca; verbatimEventDate: 15-Nov-2004; **Record Level:** language: en; institutionCode: CNC; collectionCode: Insects; basisOfRecord: Pinned Specimen
- **Type status:** Other material. **Occurrence:** occurrenceDetails: http://janzen.sas.upenn.edu; catalogNumber: DHJPAR0003315; recordedBy: D.H. Janzen, W. Hallwachs & Gloria Sihezar; individualID: DHJPAR0003315; individualCount: 1; sex: male; lifeStage: adult; preparations: pinned; otherCatalogNumbers: ASTA222-05, 04-SRNP-61141, BOLD:AAA1577; **Taxon:** scientificName: Hyphantrophaga virilis; phylum: Arthropoda; class: Insecta; order: Diptera; family: Tachinidae; genus: Hyphantrophaga; specificEpithet: virilis; scientificNameAuthorship: (Aldrich & Webber, 1924); **Location:** continent: Central America; country: Costa Rica; countryCode: CR; stateProvince: Alajuela; county: Sector San Cristobal; locality: Area de Conservacion Guanacaste; verbatimLocality: Puente Palma; verbatimElevation: 460; verbatimLatitude: 10.9163; verbatimLongitude: -85.3787; verbatimCoordinateSystem: Decimal; decimalLatitude: 10.9163; decimalLongitude: -85.3787; **Identification:** identifiedBy: AJ Fleming; dateIdentified: 2017; **Event:** samplingProtocol: Reared from the larva of the Hesperiidae, Polyctor polyctor; verbatimEventDate: 03-Jan-2005; **Record Level:** language: en; institutionCode: CNC; collectionCode: Insects; basisOfRecord: Pinned Specimen
- **Type status:** Other material. **Occurrence:** occurrenceDetails: http://janzen.sas.upenn.edu; catalogNumber: DHJPAR0003316; recordedBy: D.H. Janzen, W. Hallwachs & Roster Moraga; individualID: DHJPAR0003316; individualCount: 1; sex: male; lifeStage: adult; preparations: pinned; otherCatalogNumbers: ASTA223-05, 04-SRNP-26832, BOLD:AAA1577; **Taxon:** scientificName: Hyphantrophaga virilis; phylum: Arthropoda; class: Insecta; order: Diptera; family: Tachinidae; genus: Hyphantrophaga; specificEpithet: virilis; scientificNameAuthorship: (Aldrich & Webber, 1924); **Location:** continent: Central America; country: Costa Rica; countryCode: CR; stateProvince: Guanacaste; county: Sector Del Oro; locality: Area de Conservacion Guanacaste; verbatimLocality: Bosque Aguirre; verbatimElevation: 620; verbatimLatitude: 11.0006; verbatimLongitude: -85.438; verbatimCoordinateSystem: Decimal; decimalLatitude: 11.0006; decimalLongitude: -85.438; **Identification:** identifiedBy: AJ Fleming; dateIdentified: 2017; **Event:** samplingProtocol: Reared from the larva of the Crambidae, Syllepte amandoDHJ02; verbatimEventDate: 22-Jan-2005; **Record Level:** language: en; institutionCode: CNC; collectionCode: Insects; basisOfRecord: Pinned Specimen
- **Type status:** Other material. **Occurrence:** occurrenceDetails: http://janzen.sas.upenn.edu; catalogNumber: DHJPAR0003321; recordedBy: D.H. Janzen, W. Hallwachs & Jose Alberto Sanchez; individualID: DHJPAR0003321; individualCount: 1; sex: male; lifeStage: adult; preparations: pinned; otherCatalogNumbers: ASTA228-05, 05-SRNP-58573, BOLD:AAA1577; **Taxon:** scientificName: Hyphantrophaga virilis; phylum: Arthropoda; class: Insecta; order: Diptera; family: Tachinidae; genus: Hyphantrophaga; specificEpithet: virilis; scientificNameAuthorship: (Aldrich & Webber, 1924); **Location:** continent: Central America; country: Costa Rica; countryCode: CR; stateProvince: Guanacaste; county: Sector Mundo Nuevo; locality: Area de Conservacion Guanacaste; verbatimLocality: Vado Agria; verbatimElevation: 560; verbatimLatitude: 10.7588; verbatimLongitude: -85.3754; verbatimCoordinateSystem: Decimal; decimalLatitude: 10.7588; decimalLongitude: -85.3754; **Identification:** identifiedBy: AJ Fleming; dateIdentified: 2017; **Event:** samplingProtocol: Reared from the larva of the Notodontidae, Elasmia mandela; verbatimEventDate: 23-Aug-2005; **Record Level:** language: en; institutionCode: CNC; collectionCode: Insects; basisOfRecord: Pinned Specimen
- **Type status:** Other material. **Occurrence:** occurrenceDetails: http://janzen.sas.upenn.edu; catalogNumber: DHJPAR0003322; recordedBy: D.H. Janzen, W. Hallwachs & Mariano Pereira; individualID: DHJPAR0003322; individualCount: 1; sex: male; lifeStage: adult; preparations: pinned; otherCatalogNumbers: ASTA229-05, 05-SRNP-58078, BOLD:AAA1577; **Taxon:** scientificName: Hyphantrophaga virilis; phylum: Arthropoda; class: Insecta; order: Diptera; family: Tachinidae; genus: Hyphantrophaga; specificEpithet: virilis; scientificNameAuthorship: (Aldrich & Webber, 1924); **Location:** continent: Central America; country: Costa Rica; countryCode: CR; stateProvince: Guanacaste; county: Sector Mundo Nuevo; locality: Area de Conservacion Guanacaste; verbatimLocality: Mamones; verbatimElevation: 365; verbatimLatitude: 10.7707; verbatimLongitude: -85.4287; verbatimCoordinateSystem: Decimal; decimalLatitude: 10.7707; decimalLongitude: -85.4287; **Identification:** identifiedBy: AJ Fleming; dateIdentified: 2017; **Event:** samplingProtocol: Reared from the larva of the Notodontidae, Elasmia mandela; verbatimEventDate: 13-Aug-2005; **Record Level:** language: en; institutionCode: CNC; collectionCode: Insects; basisOfRecord: Pinned Specimen
- **Type status:** Other material. **Occurrence:** occurrenceDetails: http://janzen.sas.upenn.edu; catalogNumber: DHJPAR0003324; recordedBy: D.H. Janzen, W. Hallwachs & Ruth Franco; individualID: DHJPAR0003324; individualCount: 1; sex: male; lifeStage: adult; preparations: pinned; otherCatalogNumbers: ASTA231-05, 04-SRNP-14651, BOLD:AAA1577; **Taxon:** scientificName: Hyphantrophaga virilis; phylum: Arthropoda; class: Insecta; order: Diptera; family: Tachinidae; genus: Hyphantrophaga; specificEpithet: virilis; scientificNameAuthorship: (Aldrich & Webber, 1924); **Location:** continent: Central America; country: Costa Rica; countryCode: CR; stateProvince: Guanacaste; county: Sector Santa Rosa; locality: Area de Conservacion Guanacaste; verbatimLocality: Bosque Humedo; verbatimElevation: 290; verbatimLatitude: 10.8514; verbatimLongitude: -85.608; verbatimCoordinateSystem: Decimal; decimalLatitude: 10.8514; decimalLongitude: -85.608; **Identification:** identifiedBy: AJ Fleming; dateIdentified: 2017; **Event:** samplingProtocol: Reared from the larva of the Hesperiidae, Nascus Burns01; verbatimEventDate: 11-Nov-2004; **Record Level:** language: en; institutionCode: CNC; collectionCode: Insects; basisOfRecord: Pinned Specimen
- **Type status:** Other material. **Occurrence:** occurrenceDetails: http://janzen.sas.upenn.edu; catalogNumber: DHJPAR0003330; recordedBy: D.H. Janzen, W. Hallwachs & Mariano Pereira; individualID: DHJPAR0003330; individualCount: 1; sex: male; lifeStage: adult; preparations: pinned; otherCatalogNumbers: ASTA237-05, 04-SRNP-35033, BOLD:AAA1577; **Taxon:** scientificName: Hyphantrophaga virilis; phylum: Arthropoda; class: Insecta; order: Diptera; family: Tachinidae; genus: Hyphantrophaga; specificEpithet: virilis; scientificNameAuthorship: (Aldrich & Webber, 1924); **Location:** continent: Central America; country: Costa Rica; countryCode: CR; stateProvince: Guanacaste; county: Sector Cacao; locality: Area de Conservacion Guanacaste; verbatimLocality: Sendero Ponderosa; verbatimElevation: 1060; verbatimLatitude: 10.9146; verbatimLongitude: -85.4626; verbatimCoordinateSystem: Decimal; decimalLatitude: 10.9146; decimalLongitude: -85.4626; **Identification:** identifiedBy: AJ Fleming; dateIdentified: 2017; **Event:** samplingProtocol: Reared from the larva of the Erebidae, Pachydota drucei; verbatimEventDate: 06-Mar-2004; **Record Level:** language: en; institutionCode: CNC; collectionCode: Insects; basisOfRecord: Pinned Specimen
- **Type status:** Other material. **Occurrence:** occurrenceDetails: http://janzen.sas.upenn.edu; catalogNumber: DHJPAR0003332; recordedBy: D.H. Janzen, W. Hallwachs & Lucia Rios; individualID: DHJPAR0003332; individualCount: 1; sex: male; lifeStage: adult; preparations: pinned; otherCatalogNumbers: ASTA239-05, 04-SRNP-20238, BOLD:AAA1577; **Taxon:** scientificName: Hyphantrophaga virilis; phylum: Arthropoda; class: Insecta; order: Diptera; family: Tachinidae; genus: Hyphantrophaga; specificEpithet: virilis; scientificNameAuthorship: (Aldrich & Webber, 1924); **Location:** continent: Central America; country: Costa Rica; countryCode: CR; stateProvince: Guanacaste; county: Sector Del Oro; locality: Area de Conservacion Guanacaste; verbatimLocality: Canyon Rio Mena; verbatimElevation: 560; verbatimLatitude: 10.9962; verbatimLongitude: -85.4556; verbatimCoordinateSystem: Decimal; decimalLatitude: 10.9962; decimalLongitude: -85.4556; **Identification:** identifiedBy: AJ Fleming; dateIdentified: 2017; **Event:** samplingProtocol: Reared from the larva of the Hesperiidae, Polyctor polyctor; verbatimEventDate: 17-Feb-2004; **Record Level:** language: en; institutionCode: CNC; collectionCode: Insects; basisOfRecord: Pinned Specimen
- **Type status:** Other material. **Occurrence:** occurrenceDetails: http://janzen.sas.upenn.edu; catalogNumber: DHJPAR0003333; recordedBy: D.H. Janzen, W. Hallwachs & Mariano Pereira; individualID: DHJPAR0003333; individualCount: 1; sex: male; lifeStage: adult; preparations: pinned; otherCatalogNumbers: ASTA240-05, 03-SRNP-3649, BOLD:AAA1577; **Taxon:** scientificName: Hyphantrophaga virilis; phylum: Arthropoda; class: Insecta; order: Diptera; family: Tachinidae; genus: Hyphantrophaga; specificEpithet: virilis; scientificNameAuthorship: (Aldrich & Webber, 1924); **Location:** continent: Central America; country: Costa Rica; countryCode: CR; stateProvince: Guanacaste; county: Sector Cacao; locality: Area de Conservacion Guanacaste; verbatimLocality: Casa Fran; verbatimElevation: 1140; verbatimLatitude: 10.9366; verbatimLongitude: -85.4669; verbatimCoordinateSystem: Decimal; decimalLatitude: 10.9366; decimalLongitude: -85.4669; **Identification:** identifiedBy: AJ Fleming; dateIdentified: 2017; **Event:** samplingProtocol: Reared from the larva of the Nymphalidae, Mesotaenia barnesi; verbatimEventDate: 14-May-2003; **Record Level:** language: en; institutionCode: CNC; collectionCode: Insects; basisOfRecord: Pinned Specimen
- **Type status:** Other material. **Occurrence:** occurrenceDetails: http://janzen.sas.upenn.edu; catalogNumber: DHJPAR0003336; recordedBy: D.H. Janzen, W. Hallwachs & Freddy Quesada; individualID: DHJPAR0003336; individualCount: 1; sex: male; lifeStage: adult; preparations: pinned; otherCatalogNumbers: ASTA243-05, 03-SRNP-3459, BOLD:AAA1577; **Taxon:** scientificName: Hyphantrophaga virilis; phylum: Arthropoda; class: Insecta; order: Diptera; family: Tachinidae; genus: Hyphantrophaga; specificEpithet: virilis; scientificNameAuthorship: (Aldrich & Webber, 1924); **Location:** continent: Central America; country: Costa Rica; countryCode: CR; stateProvince: Guanacaste; county: Sector Cacao; locality: Area de Conservacion Guanacaste; verbatimLocality: Sendero Maritza; verbatimElevation: 760; verbatimLatitude: 10.9364; verbatimLongitude: -85.4776; verbatimCoordinateSystem: Decimal; decimalLatitude: 10.9364; decimalLongitude: -85.4776; **Identification:** identifiedBy: AJ Fleming; dateIdentified: 2017; **Event:** samplingProtocol: Reared from the larva of the Geometridae, geometrid 01-SRNP-6789; verbatimEventDate: 18-Apr-2003; **Record Level:** language: en; institutionCode: CNC; collectionCode: Insects; basisOfRecord: Pinned Specimen
- **Type status:** Other material. **Occurrence:** occurrenceDetails: http://janzen.sas.upenn.edu; catalogNumber: DHJPAR0003339; recordedBy: D.H. Janzen, W. Hallwachs & Freddy Quesada; individualID: DHJPAR0003339; individualCount: 1; sex: male; lifeStage: adult; preparations: pinned; otherCatalogNumbers: ASTA246-05, 02-SRNP-9335,; **Taxon:** scientificName: Hyphantrophaga virilis; phylum: Arthropoda; class: Insecta; order: Diptera; family: Tachinidae; genus: Hyphantrophaga; specificEpithet: virilis; scientificNameAuthorship: (Aldrich & Webber, 1924); **Location:** continent: Central America; country: Costa Rica; countryCode: CR; stateProvince: Guanacaste; county: Sector Cacao; locality: Area de Conservacion Guanacaste; verbatimLocality: Sendero Abajo; verbatimElevation: 1020; verbatimLatitude: 10.9255; verbatimLongitude: -85.4716; verbatimCoordinateSystem: Decimal; decimalLatitude: 10.9255; decimalLongitude: -85.4716; **Identification:** identifiedBy: AJ Fleming; dateIdentified: 2017; **Event:** samplingProtocol: Reared from the larva of the Pyralidae, Omphalocera cariosa; verbatimEventDate: 01-Aug-2002; **Record Level:** language: en; institutionCode: CNC; collectionCode: Insects; basisOfRecord: Pinned Specimen
- **Type status:** Other material. **Occurrence:** occurrenceDetails: http://janzen.sas.upenn.edu; catalogNumber: DHJPAR0005680; recordedBy: D.H. Janzen, W. Hallwachs & Ruth Franco; individualID: DHJPAR0005680; individualCount: 1; sex: male; lifeStage: adult; preparations: pinned; otherCatalogNumbers: ASTAI108-06, 03-SRNP-25554, BOLD:AAA1577; **Taxon:** scientificName: Hyphantrophaga virilis; phylum: Arthropoda; class: Insecta; order: Diptera; family: Tachinidae; genus: Hyphantrophaga; specificEpithet: virilis; scientificNameAuthorship: (Aldrich & Webber, 1924); **Location:** country: Costa Rica; countryCode: CR; stateProvince: Guanacaste; county: Sector Santa Rosa; locality: Area de Conservacion Guanacaste; verbatimLocality: Area Administrativa; verbatimElevation: 295; verbatimLatitude: 10.8376; verbatimLongitude: -85.6187; verbatimCoordinateSystem: Decimal; decimalLatitude: 10.8376; decimalLongitude: -85.6187; **Event:** samplingProtocol: Reared from the larva of the Hesperiidae, Mysoria ambigua; verbatimEventDate: 08-Nov-2003; **Record Level:** language: en; institutionCode: CNC; collectionCode: Insects; basisOfRecord: Pinned Specimen
- **Type status:** Other material. **Occurrence:** occurrenceDetails: http://janzen.sas.upenn.edu; catalogNumber: DHJPAR0006292; recordedBy: D.H. Janzen, W. Hallwachs & Manuel Pereira; individualID: DHJPAR0006292; individualCount: 1; sex: male; lifeStage: adult; preparations: pinned; otherCatalogNumbers: ASTAI720-06, 05-SRNP-47173, BOLD:AAA1577; **Taxon:** scientificName: Hyphantrophaga virilis; phylum: Arthropoda; class: Insecta; order: Diptera; family: Tachinidae; genus: Hyphantrophaga; specificEpithet: virilis; scientificNameAuthorship: (Aldrich & Webber, 1924); **Location:** country: Costa Rica; countryCode: CR; stateProvince: Guanacaste; county: Sector Cacao; locality: Area de Conservacion Guanacaste; verbatimLocality: Cuesta Caimito; verbatimElevation: 640; verbatimLatitude: 10.8908; verbatimLongitude: -85.4719; verbatimCoordinateSystem: Decimal; decimalLatitude: 10.8908; decimalLongitude: -85.4719; **Event:** samplingProtocol: Reared from the larva of the Noctuidae, Dyops cuprescens; verbatimEventDate: 13-Sep-2005; **Record Level:** language: en; institutionCode: CNC; collectionCode: Insects; basisOfRecord: Pinned Specimen
- **Type status:** Other material. **Occurrence:** occurrenceDetails: http://janzen.sas.upenn.edu; catalogNumber: DHJPAR0006364; recordedBy: D.H. Janzen, W. Hallwachs & Ruth Franco; individualID: DHJPAR0006364; individualCount: 1; sex: male; lifeStage: adult; preparations: pinned; otherCatalogNumbers: ASTAI792-06, 03-SRNP-25976, BOLD:AAA1577; **Taxon:** scientificName: Hyphantrophaga virilis; phylum: Arthropoda; class: Insecta; order: Diptera; family: Tachinidae; genus: Hyphantrophaga; specificEpithet: virilis; scientificNameAuthorship: (Aldrich & Webber, 1924); **Location:** country: Costa Rica; countryCode: CR; stateProvince: Guanacaste; county: Sector Santa Rosa; locality: Area de Conservacion Guanacaste; verbatimLocality: Cafetal; verbatimElevation: 280; verbatimLatitude: 10.8583; verbatimLongitude: -85.6109; verbatimCoordinateSystem: Decimal; decimalLatitude: 10.8583; decimalLongitude: -85.6109; **Event:** samplingProtocol: Reared from the larva of the Hesperiidae, Saliana fusta; verbatimEventDate: 22-Oct-2003; **Record Level:** language: en; institutionCode: CNC; collectionCode: Insects; basisOfRecord: Pinned Specimen
- **Type status:** Other material. **Occurrence:** occurrenceDetails: http://janzen.sas.upenn.edu; catalogNumber: DHJPAR0006383; recordedBy: D.H. Janzen, W. Hallwachs & Guillermo Pereira; individualID: DHJPAR0006383; individualCount: 1; sex: male; lifeStage: adult; preparations: pinned; otherCatalogNumbers: ASTAI811-06, 01-SRNP-18100, BOLD:AAA1577; **Taxon:** scientificName: Hyphantrophaga virilis; phylum: Arthropoda; class: Insecta; order: Diptera; family: Tachinidae; genus: Hyphantrophaga; specificEpithet: virilis; scientificNameAuthorship: (Aldrich & Webber, 1924); **Location:** country: Costa Rica; countryCode: CR; stateProvince: Guanacaste; county: Sector Santa Rosa; locality: Area de Conservacion Guanacaste; verbatimLocality: Cafetal; verbatimElevation: 280; verbatimLatitude: 10.8583; verbatimLongitude: -85.6109; verbatimCoordinateSystem: Decimal; decimalLatitude: 10.8583; decimalLongitude: -85.6109; **Event:** samplingProtocol: Reared from the larva of the Notodontidae, Tachuda plumipesICG02; verbatimEventDate: 29-Nov-2001; **Record Level:** language: en; institutionCode: CNC; collectionCode: Insects; basisOfRecord: Pinned Specimen
- **Type status:** Other material. **Occurrence:** occurrenceDetails: http://janzen.sas.upenn.edu; catalogNumber: DHJPAR0005545; recordedBy: D.H. Janzen, W. Hallwachs & Mariano Pereira; individualID: DHJPAR0005545; individualCount: 1; sex: male; lifeStage: adult; preparations: pinned; otherCatalogNumbers: ASTA664-06, 05-SRNP-65176, BOLD:AAA1577; **Taxon:** scientificName: Hyphantrophaga virilis; phylum: Arthropoda; class: Insecta; order: Diptera; family: Tachinidae; genus: Hyphantrophaga; specificEpithet: virilis; scientificNameAuthorship: (Aldrich & Webber, 1924); **Location:** country: Costa Rica; countryCode: CR; stateProvince: Guanacaste; county: Sector Mundo Nuevo; locality: Area de Conservacion Guanacaste; verbatimLocality: Estacion La Perla; verbatimElevation: 325; verbatimLatitude: 10.7674; verbatimLongitude: -85.4331; verbatimCoordinateSystem: Decimal; decimalLatitude: 10.7674; decimalLongitude: -85.4331; **Event:** samplingProtocol: Reared from the larva of the Hesperiidae, Mysoria ambigua; verbatimEventDate: 23-Nov-2005; **Record Level:** language: en; institutionCode: CNC; collectionCode: Insects; basisOfRecord: Pinned Specimen
- **Type status:** Other material. **Occurrence:** occurrenceDetails: http://janzen.sas.upenn.edu; catalogNumber: DHJPAR0006621; recordedBy: D.H. Janzen, W. Hallwachs & Guillermo Pereira; individualID: DHJPAR0006621; individualCount: 1; sex: male; lifeStage: adult; preparations: pinned; otherCatalogNumbers: ASTA799-06, 05-SRNP-64402, BOLD:AAA1577; **Taxon:** scientificName: Hyphantrophaga virilis; phylum: Arthropoda; class: Insecta; order: Diptera; family: Tachinidae; genus: Hyphantrophaga; specificEpithet: virilis; scientificNameAuthorship: (Aldrich & Webber, 1924); **Location:** country: Costa Rica; countryCode: CR; stateProvince: Guanacaste; county: Sector Santa Rosa; locality: Area de Conservacion Guanacaste; verbatimLocality: Bosque Humedo; verbatimElevation: 290; verbatimLatitude: 10.8514; verbatimLongitude: -85.608; verbatimCoordinateSystem: Decimal; decimalLatitude: 10.8514; decimalLongitude: -85.608; **Event:** samplingProtocol: Reared from the larva of the Hesperiidae, Gindanes brontinus; verbatimEventDate: 23-Jan-2005; **Record Level:** language: en; institutionCode: CNC; collectionCode: Insects; basisOfRecord: Pinned Specimen
- **Type status:** Other material. **Occurrence:** occurrenceDetails: http://janzen.sas.upenn.edu; catalogNumber: DHJPAR0006688; recordedBy: D.H. Janzen, W. Hallwachs & Mariano Pereira; individualID: DHJPAR0006688; individualCount: 1; sex: male; lifeStage: adult; preparations: pinned; otherCatalogNumbers: ASTA866-06, 05-SRNP-65604,; **Taxon:** scientificName: Hyphantrophaga virilis; phylum: Arthropoda; class: Insecta; order: Diptera; family: Tachinidae; genus: Hyphantrophaga; specificEpithet: virilis; scientificNameAuthorship: (Aldrich & Webber, 1924); **Location:** country: Costa Rica; countryCode: CR; stateProvince: Guanacaste; county: Sector Mundo Nuevo; locality: Area de Conservacion Guanacaste; verbatimLocality: Vado Miramonte; verbatimElevation: 305; verbatimLatitude: 10.7718; verbatimLongitude: -85.434; verbatimCoordinateSystem: Decimal; decimalLatitude: 10.7718; decimalLongitude: -85.434; **Event:** samplingProtocol: Reared from the larva of the Depressariidae, Anadasmus Janzen25; verbatimEventDate: 16-Dec-2005; **Record Level:** language: en; institutionCode: CNC; collectionCode: Insects; basisOfRecord: Pinned Specimen
- **Type status:** Other material. **Occurrence:** occurrenceDetails: http://janzen.sas.upenn.edu; catalogNumber: DHJPAR0007064; recordedBy: D.H. Janzen, W. Hallwachs & Manuel Rios; individualID: DHJPAR0007064; individualCount: 1; sex: male; lifeStage: adult; preparations: pinned; otherCatalogNumbers: ASTAV306-06, 06-SRNP-30587,; **Taxon:** scientificName: Hyphantrophaga virilis; phylum: Arthropoda; class: Insecta; order: Diptera; family: Tachinidae; genus: Hyphantrophaga; specificEpithet: virilis; scientificNameAuthorship: (Aldrich & Webber, 1924); **Location:** country: Costa Rica; countryCode: CR; stateProvince: Guanacaste; county: Sector Pitilla; locality: Area de Conservacion Guanacaste; verbatimLocality: Loaiciga; verbatimElevation: 445; verbatimLatitude: 11.0198; verbatimLongitude: -85.4134; verbatimCoordinateSystem: Decimal; decimalLatitude: 11.0198; decimalLongitude: -85.4134; **Event:** samplingProtocol: Reared from the larva of the Hesperiidae, Xenophanes tryxus; verbatimEventDate: 15-Mar-2006; **Record Level:** language: en; institutionCode: CNC; collectionCode: Insects; basisOfRecord: Pinned Specimen
- **Type status:** Other material. **Occurrence:** occurrenceDetails: http://janzen.sas.upenn.edu; catalogNumber: DHJPAR0007090; recordedBy: D.H. Janzen, W. Hallwachs & Jose Alberto Sanchez; individualID: DHJPAR0007090; individualCount: 1; sex: male; lifeStage: adult; preparations: pinned; otherCatalogNumbers: ASTAV332-06, 06-SRNP-55067,; **Taxon:** scientificName: Hyphantrophaga virilis; phylum: Arthropoda; class: Insecta; order: Diptera; family: Tachinidae; genus: Hyphantrophaga; specificEpithet: virilis; scientificNameAuthorship: (Aldrich & Webber, 1924); **Location:** country: Costa Rica; countryCode: CR; stateProvince: Guanacaste; county: Sector Mundo Nuevo; locality: Area de Conservacion Guanacaste; verbatimLocality: Mamones; verbatimElevation: 365; verbatimLatitude: 10.7707; verbatimLongitude: -85.4287; verbatimCoordinateSystem: Decimal; decimalLatitude: 10.7707; decimalLongitude: -85.4287; **Event:** samplingProtocol: Reared from the larva of the Bombycidae, Epia muscosa; verbatimEventDate: 10-Feb-2006; **Record Level:** language: en; institutionCode: CNC; collectionCode: Insects; basisOfRecord: Pinned Specimen
- **Type status:** Other material. **Occurrence:** occurrenceDetails: http://janzen.sas.upenn.edu; catalogNumber: DHJPAR0007095; recordedBy: D.H. Janzen, W. Hallwachs & Jose Alberto Sanchez; individualID: DHJPAR0007095; individualCount: 1; sex: male; lifeStage: adult; preparations: pinned; otherCatalogNumbers: ASTAV337-06, 06-SRNP-55244,; **Taxon:** scientificName: Hyphantrophaga virilis; phylum: Arthropoda; class: Insecta; order: Diptera; family: Tachinidae; genus: Hyphantrophaga; specificEpithet: virilis; scientificNameAuthorship: (Aldrich & Webber, 1924); **Location:** country: Costa Rica; countryCode: CR; stateProvince: Guanacaste; county: Sector Mundo Nuevo; locality: Area de Conservacion Guanacaste; verbatimLocality: Mamones; verbatimElevation: 365; verbatimLatitude: 10.7707; verbatimLongitude: -85.4287; verbatimCoordinateSystem: Decimal; decimalLatitude: 10.7707; decimalLongitude: -85.4287; **Event:** samplingProtocol: Reared from the larva of the Nymphalidae, Colobura dirce; verbatimEventDate: 10-Feb-2006; **Record Level:** language: en; institutionCode: CNC; collectionCode: Insects; basisOfRecord: Pinned Specimen
- **Type status:** Other material. **Occurrence:** occurrenceDetails: http://janzen.sas.upenn.edu; catalogNumber: DHJPAR0007124; recordedBy: D.H. Janzen, W. Hallwachs & Carolina Cano; individualID: DHJPAR0007124; individualCount: 1; sex: male; lifeStage: adult; preparations: pinned; otherCatalogNumbers: ASTAV366-06, 06-SRNP-1423, BOLD:AAA1577; **Taxon:** scientificName: Hyphantrophaga virilis; phylum: Arthropoda; class: Insecta; order: Diptera; family: Tachinidae; genus: Hyphantrophaga; specificEpithet: virilis; scientificNameAuthorship: (Aldrich & Webber, 1924); **Location:** country: Costa Rica; countryCode: CR; stateProvince: Alajuela; county: Sector San Cristobal; locality: Area de Conservacion Guanacaste; verbatimLocality: Rio Blanco Abajo; verbatimElevation: 500; verbatimLatitude: 10.9004; verbatimLongitude: -85.3725; verbatimCoordinateSystem: Decimal; decimalLatitude: 10.9004; decimalLongitude: -85.3725; **Event:** samplingProtocol: Reared from the larva of the Hesperiidae, Talides sinois; verbatimEventDate: 26-Mar-2006; **Record Level:** language: en; institutionCode: CNC; collectionCode: Insects; basisOfRecord: Pinned Specimen
- **Type status:** Other material. **Occurrence:** occurrenceDetails: http://janzen.sas.upenn.edu; catalogNumber: DHJPAR0007150; recordedBy: D.H. Janzen, W. Hallwachs & Gloria Sihezar; individualID: DHJPAR0007150; individualCount: 1; sex: male; lifeStage: adult; preparations: pinned; otherCatalogNumbers: ASTAV392-06, 06-SRNP-81, BOLD:AAA1577; **Taxon:** scientificName: Hyphantrophaga virilis; phylum: Arthropoda; class: Insecta; order: Diptera; family: Tachinidae; genus: Hyphantrophaga; specificEpithet: virilis; scientificNameAuthorship: (Aldrich & Webber, 1924); **Location:** country: Costa Rica; countryCode: CR; stateProvince: Alajuela; county: Sector San Cristobal; locality: Area de Conservacion Guanacaste; verbatimLocality: Sendero Huerta; verbatimElevation: 527; verbatimLatitude: 10.9305; verbatimLongitude: -85.3722; verbatimCoordinateSystem: Decimal; decimalLatitude: 10.9305; decimalLongitude: -85.3722; **Event:** samplingProtocol: Reared from the larva of the Crambidae, Pantographa suffusalis; verbatimEventDate: 22-Feb-2006; **Record Level:** language: en; institutionCode: CNC; collectionCode: Insects; basisOfRecord: Pinned Specimen
- **Type status:** Other material. **Occurrence:** occurrenceDetails: http://janzen.sas.upenn.edu; catalogNumber: DHJPAR0010315; recordedBy: D.H. Janzen, W. Hallwachs & Lucia Vargas; individualID: DHJPAR0010315; individualCount: 1; sex: male; lifeStage: adult; preparations: pinned; otherCatalogNumbers: ASTAS146-06, 06-SRNP-13968, BOLD:AAA1577; **Taxon:** scientificName: Hyphantrophaga virilis; phylum: Arthropoda; class: Insecta; order: Diptera; family: Tachinidae; genus: Hyphantrophaga; specificEpithet: virilis; scientificNameAuthorship: (Aldrich & Webber, 1924); **Location:** country: Costa Rica; countryCode: CR; stateProvince: Guanacaste; county: Sector Santa Rosa; locality: Area de Conservacion Guanacaste; verbatimLocality: Cuesta Canyon Tigre; verbatimElevation: 270; verbatimLatitude: 10.817; verbatimLongitude: -85.6437; verbatimCoordinateSystem: Decimal; decimalLatitude: 10.817; decimalLongitude: -85.6437; **Event:** samplingProtocol: Reared from the larva of the Noctuidae, Euscirrhopterus poeyi; verbatimEventDate: 18-Jun-2006; **Record Level:** language: en; institutionCode: CNC; collectionCode: Insects; basisOfRecord: Pinned Specimen
- **Type status:** Other material. **Occurrence:** occurrenceDetails: http://janzen.sas.upenn.edu; catalogNumber: DHJPAR0010448; recordedBy: D.H. Janzen, W. Hallwachs & Harry Ramirez; individualID: DHJPAR0010448; individualCount: 1; sex: male; lifeStage: adult; preparations: pinned; otherCatalogNumbers: ASTAS279-06, 06-SRNP-45690, BOLD:AAA1577; **Taxon:** scientificName: Hyphantrophaga virilis; phylum: Arthropoda; class: Insecta; order: Diptera; family: Tachinidae; genus: Hyphantrophaga; specificEpithet: virilis; scientificNameAuthorship: (Aldrich & Webber, 1924); **Location:** country: Costa Rica; countryCode: CR; stateProvince: Guanacaste; county: Sector Cacao; locality: Area de Conservacion Guanacaste; verbatimLocality: Quebrada Otilio; verbatimElevation: 550; verbatimLatitude: 10.89; verbatimLongitude: -85.4797; verbatimCoordinateSystem: Decimal; decimalLatitude: 10.89; decimalLongitude: -85.4797; **Event:** samplingProtocol: Reared from the larva of the Erebidae, Phyprosopus parthenope; verbatimEventDate: 21-Jul-2006; **Record Level:** language: en; institutionCode: CNC; collectionCode: Insects; basisOfRecord: Pinned Specimen
- **Type status:** Other material. **Occurrence:** occurrenceDetails: http://janzen.sas.upenn.edu; catalogNumber: DHJPAR0010497; recordedBy: D.H. Janzen, W. Hallwachs & Jose Alberto Sanchez; individualID: DHJPAR0010497; individualCount: 1; sex: male; lifeStage: adult; preparations: pinned; otherCatalogNumbers: ASTAS328-06, 06-SRNP-56269, BOLD:AAA1577; **Taxon:** scientificName: Hyphantrophaga virilis; phylum: Arthropoda; class: Insecta; order: Diptera; family: Tachinidae; genus: Hyphantrophaga; specificEpithet: virilis; scientificNameAuthorship: (Aldrich & Webber, 1924); **Location:** country: Costa Rica; countryCode: CR; stateProvince: Guanacaste; county: Sector Mundo Nuevo; locality: Area de Conservacion Guanacaste; verbatimLocality: Estacion La Perla; verbatimElevation: 325; verbatimLatitude: 10.7674; verbatimLongitude: -85.4331; verbatimCoordinateSystem: Decimal; decimalLatitude: 10.7674; decimalLongitude: -85.4331; **Event:** samplingProtocol: Reared from the larva of the Nymphalidae, Colobura dirce; verbatimEventDate: 29-Jun-2006; **Record Level:** language: en; institutionCode: CNC; collectionCode: Insects; basisOfRecord: Pinned Specimen
- **Type status:** Other material. **Occurrence:** occurrenceDetails: http://janzen.sas.upenn.edu; catalogNumber: DHJPAR0010500; recordedBy: D.H. Janzen, W. Hallwachs & Mariano Pereira; individualID: DHJPAR0010500; individualCount: 1; sex: male; lifeStage: adult; preparations: pinned; otherCatalogNumbers: ASTAS331-06, 06-SRNP-56178,; **Taxon:** scientificName: Hyphantrophaga virilis; phylum: Arthropoda; class: Insecta; order: Diptera; family: Tachinidae; genus: Hyphantrophaga; specificEpithet: virilis; scientificNameAuthorship: (Aldrich & Webber, 1924); **Location:** country: Costa Rica; countryCode: CR; stateProvince: Guanacaste; county: Sector Mundo Nuevo; locality: Area de Conservacion Guanacaste; verbatimLocality: Estacion La Perla; verbatimElevation: 325; verbatimLatitude: 10.7674; verbatimLongitude: -85.4331; verbatimCoordinateSystem: Decimal; decimalLatitude: 10.7674; decimalLongitude: -85.4331; **Event:** samplingProtocol: Reared from the larva of the Nymphalidae, Myscelia pattenia; verbatimEventDate: 28-Jun-2006; **Record Level:** language: en; institutionCode: CNC; collectionCode: Insects; basisOfRecord: Pinned Specimen
- **Type status:** Other material. **Occurrence:** occurrenceDetails: http://janzen.sas.upenn.edu; catalogNumber: DHJPAR0015986; recordedBy: D.H. Janzen, W. Hallwachs & Mariano Pereira; individualID: DHJPAR0015986; individualCount: 1; sex: male; lifeStage: adult; preparations: pinned; otherCatalogNumbers: ASTAP015-06, 06-SRNP-57962, BOLD:AAA1577; **Taxon:** scientificName: Hyphantrophaga virilis; phylum: Arthropoda; class: Insecta; order: Diptera; family: Tachinidae; genus: Hyphantrophaga; specificEpithet: virilis; scientificNameAuthorship: (Aldrich & Webber, 1924); **Location:** country: Costa Rica; countryCode: CR; stateProvince: Guanacaste; county: Sector Mundo Nuevo; locality: Area de Conservacion Guanacaste; verbatimLocality: Vado Lonchocarpus; verbatimElevation: 490; verbatimLatitude: 10.7623; verbatimLongitude: -85.4; verbatimCoordinateSystem: Decimal; decimalLatitude: 10.7623; decimalLongitude: -85.4; **Event:** samplingProtocol: Reared from the larva of the Nymphalidae, Mechanitis isthmia; verbatimEventDate: 09-Sep-2006; **Record Level:** language: en; institutionCode: CNC; collectionCode: Insects; basisOfRecord: Pinned Specimen
- **Type status:** Other material. **Occurrence:** occurrenceDetails: http://janzen.sas.upenn.edu; catalogNumber: DHJPAR0016037; recordedBy: D.H. Janzen, W. Hallwachs & Jose Alberto Sanchez; individualID: DHJPAR0016037; individualCount: 1; sex: male; lifeStage: adult; preparations: pinned; otherCatalogNumbers: ASTAP066-06, 06-SRNP-57772, BOLD:AAA1577; **Taxon:** scientificName: Hyphantrophaga virilis; phylum: Arthropoda; class: Insecta; order: Diptera; family: Tachinidae; genus: Hyphantrophaga; specificEpithet: virilis; scientificNameAuthorship: (Aldrich & Webber, 1924); **Location:** country: Costa Rica; countryCode: CR; stateProvince: Guanacaste; county: Sector Mundo Nuevo; locality: Area de Conservacion Guanacaste; verbatimLocality: Porton Rivas; verbatimElevation: 570; verbatimLatitude: 10.7586; verbatimLongitude: -85.3727; verbatimCoordinateSystem: Decimal; decimalLatitude: 10.7586; decimalLongitude: -85.3727; **Event:** samplingProtocol: Reared from the larva of the Notodontidae, Boriza tonac; verbatimEventDate: 10-Sep-2006; **Record Level:** language: en; institutionCode: CNC; collectionCode: Insects; basisOfRecord: Pinned Specimen
- **Type status:** Other material. **Occurrence:** occurrenceDetails: http://janzen.sas.upenn.edu; catalogNumber: DHJPAR0016080; recordedBy: D.H. Janzen, W. Hallwachs & Jose Alberto Sanchez; individualID: DHJPAR0016080; individualCount: 1; sex: male; lifeStage: adult; preparations: pinned; otherCatalogNumbers: ASTAP109-06, 06-SRNP-58018, BOLD:AAA1577; **Taxon:** scientificName: Hyphantrophaga virilis; phylum: Arthropoda; class: Insecta; order: Diptera; family: Tachinidae; genus: Hyphantrophaga; specificEpithet: virilis; scientificNameAuthorship: (Aldrich & Webber, 1924); **Location:** country: Costa Rica; countryCode: CR; stateProvince: Guanacaste; county: Sector Mundo Nuevo; locality: Area de Conservacion Guanacaste; verbatimLocality: Mamones; verbatimElevation: 365; verbatimLatitude: 10.7707; verbatimLongitude: -85.4287; verbatimCoordinateSystem: Decimal; decimalLatitude: 10.7707; decimalLongitude: -85.4287; **Event:** samplingProtocol: Reared from the larva of the Erebidae, Anomis editrix; verbatimEventDate: 26-Sep-2006; **Record Level:** language: en; institutionCode: CNC; collectionCode: Insects; basisOfRecord: Pinned Specimen
- **Type status:** Other material. **Occurrence:** occurrenceDetails: http://janzen.sas.upenn.edu; catalogNumber: DHJPAR0016324; recordedBy: D.H. Janzen, W. Hallwachs & Manuel Pereira; individualID: DHJPAR0016324; individualCount: 1; sex: male; lifeStage: adult; preparations: pinned; otherCatalogNumbers: ASTAP353-06, 06-SRNP-35176, BOLD:AAA1577; **Taxon:** scientificName: Hyphantrophaga virilis; phylum: Arthropoda; class: Insecta; order: Diptera; family: Tachinidae; genus: Hyphantrophaga; specificEpithet: virilis; scientificNameAuthorship: (Aldrich & Webber, 1924); **Location:** country: Costa Rica; countryCode: CR; stateProvince: Guanacaste; county: Sector Cacao; locality: Area de Conservacion Guanacaste; verbatimLocality: Casa Fran; verbatimElevation: 1140; verbatimLatitude: 10.9366; verbatimLongitude: -85.4669; verbatimCoordinateSystem: Decimal; decimalLatitude: 10.9366; decimalLongitude: -85.4669; **Event:** samplingProtocol: Reared from the larva of the Hesperiidae, Ridens biolleyi; verbatimEventDate: 06-Sep-2006; **Record Level:** language: en; institutionCode: CNC; collectionCode: Insects; basisOfRecord: Pinned Specimen
- **Type status:** Other material. **Occurrence:** occurrenceDetails: http://janzen.sas.upenn.edu; catalogNumber: DHJPAR0016347; recordedBy: D.H. Janzen, W. Hallwachs & Manuel Pereira; individualID: DHJPAR0016347; individualCount: 1; sex: male; lifeStage: adult; preparations: pinned; otherCatalogNumbers: ASTAP376-06, 06-SRNP-47353, BOLD:AAA1577; **Taxon:** scientificName: Hyphantrophaga virilis; phylum: Arthropoda; class: Insecta; order: Diptera; family: Tachinidae; genus: Hyphantrophaga; specificEpithet: virilis; scientificNameAuthorship: (Aldrich & Webber, 1924); **Location:** country: Costa Rica; countryCode: CR; stateProvince: Guanacaste; county: Sector Cacao; locality: Area de Conservacion Guanacaste; verbatimLocality: Sendero Pajarito; verbatimElevation: 600; verbatimLatitude: 10.8899; verbatimLongitude: -85.4743; verbatimCoordinateSystem: Decimal; decimalLatitude: 10.8899; decimalLongitude: -85.4743; **Event:** samplingProtocol: Reared from the larva of the Hesperiidae, Pseudonascus paulliniae; verbatimEventDate: 27-Oct-2006; **Record Level:** language: en; institutionCode: CNC; collectionCode: Insects; basisOfRecord: Pinned Specimen
- **Type status:** Other material. **Occurrence:** occurrenceDetails: http://janzen.sas.upenn.edu; catalogNumber: DHJPAR0017045; recordedBy: D.H. Janzen, W. Hallwachs & Calixto Moraga; individualID: DHJPAR0017045; individualCount: 1; sex: male; lifeStage: adult; preparations: pinned; otherCatalogNumbers: ASTAP483-07, 06-SRNP-65757, BOLD:AAA1577; **Taxon:** scientificName: Hyphantrophaga virilis; phylum: Arthropoda; class: Insecta; order: Diptera; family: Tachinidae; genus: Hyphantrophaga; specificEpithet: virilis; scientificNameAuthorship: (Aldrich & Webber, 1924); **Location:** country: Costa Rica; countryCode: CR; stateProvince: Guanacaste; county: Sector Pitilla; locality: Area de Conservacion Guanacaste; verbatimLocality: Loaiciga; verbatimElevation: 445; verbatimLatitude: 11.0198; verbatimLongitude: -85.4134; verbatimCoordinateSystem: Decimal; decimalLatitude: 11.0198; decimalLongitude: -85.4134; **Event:** samplingProtocol: Reared from the larva of the Hesperiidae, Justinia Burns01; verbatimEventDate: 10-Feb-2007; **Record Level:** language: en; institutionCode: CNC; collectionCode: Insects; basisOfRecord: Pinned Specimen
- **Type status:** Other material. **Occurrence:** occurrenceDetails: http://janzen.sas.upenn.edu; catalogNumber: DHJPAR0017185; recordedBy: D.H. Janzen, W. Hallwachs & Roster Moraga; individualID: DHJPAR0017185; individualCount: 1; sex: male; lifeStage: adult; preparations: pinned; otherCatalogNumbers: ASTAP623-07, 07-SRNP-20244, BOLD:AAA1577; **Taxon:** scientificName: Hyphantrophaga virilis; phylum: Arthropoda; class: Insecta; order: Diptera; family: Tachinidae; genus: Hyphantrophaga; specificEpithet: virilis; scientificNameAuthorship: (Aldrich & Webber, 1924); **Location:** country: Costa Rica; countryCode: CR; stateProvince: Guanacaste; county: Sector Del Oro; locality: Area de Conservacion Guanacaste; verbatimLocality: Bosque Aguirre; verbatimElevation: 620; verbatimLatitude: 11.0006; verbatimLongitude: -85.438; verbatimCoordinateSystem: Decimal; decimalLatitude: 11.0006; decimalLongitude: -85.438; **Event:** samplingProtocol: Reared from the larva of the Hesperiidae, Cynea anthracinus; verbatimEventDate: 12-Mar-2007; **Record Level:** language: en; institutionCode: CNC; collectionCode: Insects; basisOfRecord: Pinned Specimen
- **Type status:** Other material. **Occurrence:** occurrenceDetails: http://janzen.sas.upenn.edu; catalogNumber: DHJPAR0016486; recordedBy: D.H. Janzen, W. Hallwachs & Anabelle Cordoba; individualID: DHJPAR0016486; individualCount: 1; sex: male; lifeStage: adult; preparations: pinned; otherCatalogNumbers: ASTAP690-07, 06-SRNP-9688, BOLD:AAA1577; **Taxon:** scientificName: Hyphantrophaga virilis; phylum: Arthropoda; class: Insecta; order: Diptera; family: Tachinidae; genus: Hyphantrophaga; specificEpithet: virilis; scientificNameAuthorship: (Aldrich & Webber, 1924); **Location:** country: Costa Rica; countryCode: CR; stateProvince: Alajuela; county: Sector San Cristobal; locality: Area de Conservacion Guanacaste; verbatimLocality: Puente Palma; verbatimElevation: 460; verbatimLatitude: 10.9163; verbatimLongitude: -85.3787; verbatimCoordinateSystem: Decimal; decimalLatitude: 10.9163; decimalLongitude: -85.3787; **Event:** samplingProtocol: Reared from the larva of the Crambidae, Omiodes humeralis; verbatimEventDate: 01-Jan-2007; **Record Level:** language: en; institutionCode: CNC; collectionCode: Insects; basisOfRecord: Pinned Specimen
- **Type status:** Other material. **Occurrence:** occurrenceDetails: http://janzen.sas.upenn.edu; catalogNumber: DHJPAR0016522; recordedBy: D.H. Janzen, W. Hallwachs & Jose Cortez; individualID: DHJPAR0016522; individualCount: 1; sex: male; lifeStage: adult; preparations: pinned; otherCatalogNumbers: ASTAP726-07, 06-SRNP-59750, BOLD:AAA1577; **Taxon:** scientificName: Hyphantrophaga virilis; phylum: Arthropoda; class: Insecta; order: Diptera; family: Tachinidae; genus: Hyphantrophaga; specificEpithet: virilis; scientificNameAuthorship: (Aldrich & Webber, 1924); **Location:** country: Costa Rica; countryCode: CR; stateProvince: Guanacaste; county: Sector Mundo Nuevo; locality: Area de Conservacion Guanacaste; verbatimLocality: Porton Rivas; verbatimElevation: 570; verbatimLatitude: 10.7586; verbatimLongitude: -85.3727; verbatimCoordinateSystem: Decimal; decimalLatitude: 10.7586; decimalLongitude: -85.3727; **Event:** samplingProtocol: Reared from the larva of the Lasiocampidae, Euglyphis jessiehillae; verbatimEventDate: 22-Dec-2006; **Record Level:** language: en; institutionCode: CNC; collectionCode: Insects; basisOfRecord: Pinned Specimen
- **Type status:** Other material. **Occurrence:** occurrenceDetails: http://janzen.sas.upenn.edu; catalogNumber: DHJPAR0016776; recordedBy: D.H. Janzen, W. Hallwachs & Jose Alberto Sanchez; individualID: DHJPAR0016776; individualCount: 1; sex: male; lifeStage: adult; preparations: pinned; otherCatalogNumbers: ASTAP886-07, 06-SRNP-58934, BOLD:AAA1577; **Taxon:** scientificName: Hyphantrophaga virilis; phylum: Arthropoda; class: Insecta; order: Diptera; family: Tachinidae; genus: Hyphantrophaga; specificEpithet: virilis; scientificNameAuthorship: (Aldrich & Webber, 1924); **Location:** country: Costa Rica; countryCode: CR; stateProvince: Guanacaste; county: Sector Mundo Nuevo; locality: Area de Conservacion Guanacaste; verbatimLocality: Porton Rivas; verbatimElevation: 570; verbatimLatitude: 10.7586; verbatimLongitude: -85.3727; verbatimCoordinateSystem: Decimal; decimalLatitude: 10.7586; decimalLongitude: -85.3727; **Event:** samplingProtocol: Reared from the larva of the Hesperiidae, Carrhenes canescens; verbatimEventDate: 08-Nov-2006; **Record Level:** language: en; institutionCode: CNC; collectionCode: Insects; basisOfRecord: Pinned Specimen
- **Type status:** Other material. **Occurrence:** occurrenceDetails: http://janzen.sas.upenn.edu; catalogNumber: DHJPAR0016817; recordedBy: D.H. Janzen, W. Hallwachs & Jose Alberto Sanchez; individualID: DHJPAR0016817; individualCount: 1; sex: male; lifeStage: adult; preparations: pinned; otherCatalogNumbers: ASTAP927-07, 06-SRNP-58813, BOLD:AAA1577; **Taxon:** scientificName: Hyphantrophaga virilis; phylum: Arthropoda; class: Insecta; order: Diptera; family: Tachinidae; genus: Hyphantrophaga; specificEpithet: virilis; scientificNameAuthorship: (Aldrich & Webber, 1924); **Location:** country: Costa Rica; countryCode: CR; stateProvince: Guanacaste; county: Sector Mundo Nuevo; locality: Area de Conservacion Guanacaste; verbatimLocality: Vado Lonchocarpus; verbatimElevation: 490; verbatimLatitude: 10.7623; verbatimLongitude: -85.4; verbatimCoordinateSystem: Decimal; decimalLatitude: 10.7623; decimalLongitude: -85.4; **Event:** samplingProtocol: Reared from the larva of the Pyralidae, Accinctapubes albifasciataDHJ01; verbatimEventDate: 31-Oct-2006; **Record Level:** language: en; institutionCode: CNC; collectionCode: Insects; basisOfRecord: Pinned Specimen
- **Type status:** Other material. **Occurrence:** occurrenceDetails: http://janzen.sas.upenn.edu; catalogNumber: DHJPAR0016690; recordedBy: D.H. Janzen, W. Hallwachs & Jose Alberto Sanchez; individualID: DHJPAR0016690; individualCount: 1; sex: male; lifeStage: adult; preparations: pinned; otherCatalogNumbers: ASTAP995-07, 06-SRNP-58842, BOLD:AAA1577; **Taxon:** scientificName: Hyphantrophaga virilis; phylum: Arthropoda; class: Insecta; order: Diptera; family: Tachinidae; genus: Hyphantrophaga; specificEpithet: virilis; scientificNameAuthorship: (Aldrich & Webber, 1924); **Location:** country: Costa Rica; countryCode: CR; stateProvince: Guanacaste; county: Sector Mundo Nuevo; locality: Area de Conservacion Guanacaste; verbatimLocality: Vado Lonchocarpus; verbatimElevation: 490; verbatimLatitude: 10.7623; verbatimLongitude: -85.4; verbatimCoordinateSystem: Decimal; decimalLatitude: 10.7623; decimalLongitude: -85.4; **Event:** samplingProtocol: Reared from the larva of the Pyralidae, Accinctapubes albifasciataDHJ01; verbatimEventDate: 28-Oct-2006; **Record Level:** language: en; institutionCode: CNC; collectionCode: Insects; basisOfRecord: Pinned Specimen
- **Type status:** Other material. **Occurrence:** occurrenceDetails: http://janzen.sas.upenn.edu; catalogNumber: DHJPAR0021850; recordedBy: D.H. Janzen, W. Hallwachs & Lucia Vargas; individualID: DHJPAR0021850; individualCount: 1; sex: male; lifeStage: adult; preparations: pinned; otherCatalogNumbers: ASTAT988-07, 07-SRNP-14584, BOLD:AAA1577; **Taxon:** scientificName: Hyphantrophaga virilis; phylum: Arthropoda; class: Insecta; order: Diptera; family: Tachinidae; genus: Hyphantrophaga; specificEpithet: virilis; scientificNameAuthorship: (Aldrich & Webber, 1924); **Location:** country: Costa Rica; countryCode: CR; stateProvince: Guanacaste; county: Sector Santa Rosa; locality: Area de Conservacion Guanacaste; verbatimLocality: Bosque San Emilio; verbatimElevation: 300; verbatimLatitude: 10.8439; verbatimLongitude: -85.6138; verbatimCoordinateSystem: Decimal; decimalLatitude: 10.8439; decimalLongitude: -85.6138; **Event:** samplingProtocol: Reared from the larva of the Notodontidae, Elasmia mandela; verbatimEventDate: 30-Aug-2007; **Record Level:** language: en; institutionCode: CNC; collectionCode: Insects; basisOfRecord: Pinned Specimen
- **Type status:** Other material. **Occurrence:** occurrenceDetails: http://janzen.sas.upenn.edu; catalogNumber: DHJPAR0021852; recordedBy: D.H. Janzen, W. Hallwachs & Jose Alberto Sanchez; individualID: DHJPAR0021852; individualCount: 1; sex: male; lifeStage: adult; preparations: pinned; otherCatalogNumbers: ASTAT990-07, 07-SRNP-58979, BOLD:AAA1577; **Taxon:** scientificName: Hyphantrophaga virilis; phylum: Arthropoda; class: Insecta; order: Diptera; family: Tachinidae; genus: Hyphantrophaga; specificEpithet: virilis; scientificNameAuthorship: (Aldrich & Webber, 1924); **Location:** country: Costa Rica; countryCode: CR; stateProvince: Guanacaste; county: Sector Mundo Nuevo; locality: Area de Conservacion Guanacaste; verbatimLocality: Quebrada Tibio Perla; verbatimElevation: 330; verbatimLatitude: 10.7626; verbatimLongitude: -85.4298; verbatimCoordinateSystem: Decimal; decimalLatitude: 10.7626; decimalLongitude: -85.4298; **Event:** samplingProtocol: Reared from the larva of the Nymphalidae, Siderone galanthis; verbatimEventDate: 12-Sep-2007; **Record Level:** language: en; institutionCode: CNC; collectionCode: Insects; basisOfRecord: Pinned Specimen
- **Type status:** Other material. **Occurrence:** occurrenceDetails: http://janzen.sas.upenn.edu; catalogNumber: DHJPAR0021863; recordedBy: D.H. Janzen, W. Hallwachs & Jose Alberto Sanchez; individualID: DHJPAR0021863; individualCount: 1; sex: male; lifeStage: adult; preparations: pinned; otherCatalogNumbers: ASTAT1001-07, 07-SRNP-58945, BOLD:AAA1577; **Taxon:** scientificName: Hyphantrophaga virilis; phylum: Arthropoda; class: Insecta; order: Diptera; family: Tachinidae; genus: Hyphantrophaga; specificEpithet: virilis; scientificNameAuthorship: (Aldrich & Webber, 1924); **Location:** country: Costa Rica; countryCode: CR; stateProvince: Guanacaste; county: Sector Mundo Nuevo; locality: Area de Conservacion Guanacaste; verbatimLocality: Quebrada Tibio Perla; verbatimElevation: 330; verbatimLatitude: 10.7626; verbatimLongitude: -85.4298; verbatimCoordinateSystem: Decimal; decimalLatitude: 10.7626; decimalLongitude: -85.4298; **Event:** samplingProtocol: Reared from the larva of the Notodontidae, Ianassa rusticaDHJ05; verbatimEventDate: 09-Sep-2007; **Record Level:** language: en; institutionCode: CNC; collectionCode: Insects; basisOfRecord: Pinned Specimen
- **Type status:** Other material. **Occurrence:** occurrenceDetails: http://janzen.sas.upenn.edu; catalogNumber: DHJPAR0021952; recordedBy: D.H. Janzen, W. Hallwachs & Johan Vargas; individualID: DHJPAR0021952; individualCount: 1; sex: male; lifeStage: adult; preparations: pinned; otherCatalogNumbers: ASTAT1090-07, 07-SRNP-14986, BOLD:AAA1577; **Taxon:** scientificName: Hyphantrophaga virilis; phylum: Arthropoda; class: Insecta; order: Diptera; family: Tachinidae; genus: Hyphantrophaga; specificEpithet: virilis; scientificNameAuthorship: (Aldrich & Webber, 1924); **Location:** country: Costa Rica; countryCode: CR; stateProvince: Guanacaste; county: Potrerillos; locality: Area de Conservacion Guanacaste; verbatimLocality: Rio Azufrado; verbatimElevation: 95; verbatimLatitude: 10.8122; verbatimLongitude: -85.5444; verbatimCoordinateSystem: Decimal; decimalLatitude: 10.8122; decimalLongitude: -85.5444; **Event:** samplingProtocol: Reared from the larva of the Geometridae, Opisthoxia uncinata; verbatimEventDate: 06-Sep-2007; **Record Level:** language: en; institutionCode: CNC; collectionCode: Insects; basisOfRecord: Pinned Specimen
- **Type status:** Other material. **Occurrence:** occurrenceDetails: http://janzen.sas.upenn.edu; catalogNumber: DHJPAR0021959; recordedBy: D.H. Janzen, W. Hallwachs & Johan Vargas; individualID: DHJPAR0021959; individualCount: 1; sex: male; lifeStage: adult; preparations: pinned; otherCatalogNumbers: ASTAT1097-07, 07-SRNP-15034, BOLD:AAA1577; **Taxon:** scientificName: Hyphantrophaga virilis; phylum: Arthropoda; class: Insecta; order: Diptera; family: Tachinidae; genus: Hyphantrophaga; specificEpithet: virilis; scientificNameAuthorship: (Aldrich & Webber, 1924); **Location:** country: Costa Rica; countryCode: CR; stateProvince: Guanacaste; county: Sector Horizontes; locality: Area de Conservacion Guanacaste; verbatimLocality: Vado Esperanza; verbatimElevation: 85; verbatimLatitude: 10.7894; verbatimLongitude: -85.551; verbatimCoordinateSystem: Decimal; decimalLatitude: 10.7894; decimalLongitude: -85.551; **Event:** samplingProtocol: Reared from the larva of the Pieridae, Ganyra josephina; verbatimEventDate: 10-Sep-2007; **Record Level:** language: en; institutionCode: CNC; collectionCode: Insects; basisOfRecord: Pinned Specimen
- **Type status:** Other material. **Occurrence:** occurrenceDetails: http://janzen.sas.upenn.edu; catalogNumber: DHJPAR0021988; recordedBy: D.H. Janzen, W. Hallwachs & Roster Moraga; individualID: DHJPAR0021988; individualCount: 1; sex: male; lifeStage: adult; preparations: pinned; otherCatalogNumbers: ASTAT1126-07, 07-SRNP-23254, BOLD:AAA1577; **Taxon:** scientificName: Hyphantrophaga virilis; phylum: Arthropoda; class: Insecta; order: Diptera; family: Tachinidae; genus: Hyphantrophaga; specificEpithet: virilis; scientificNameAuthorship: (Aldrich & Webber, 1924); **Location:** country: Costa Rica; countryCode: CR; stateProvince: Guanacaste; county: Sector Orosi; locality: Area de Conservacion Guanacaste; verbatimLocality: Quebrada Las Yeguitas; verbatimElevation: 560; verbatimLatitude: 10.9616; verbatimLongitude: -85.4958; verbatimCoordinateSystem: Decimal; decimalLatitude: 10.9616; decimalLongitude: -85.4958; **Event:** samplingProtocol: Reared from the larva of the Notodontidae, Ianassa druceiDHJ04; verbatimEventDate: 13-Sep-2007; **Record Level:** language: en; institutionCode: CNC; collectionCode: Insects; basisOfRecord: Pinned Specimen
- **Type status:** Other material. **Occurrence:** occurrenceDetails: http://janzen.sas.upenn.edu; catalogNumber: DHJPAR0029753; recordedBy: D.H. Janzen, W. Hallwachs & Guillermo Pereira; individualID: DHJPAR0029753; individualCount: 1; sex: male; lifeStage: adult; preparations: pinned; otherCatalogNumbers: ASHYM1174-09, 08-SRNP-14629, BOLD:AAA1577; **Taxon:** scientificName: Hyphantrophaga virilis; phylum: Arthropoda; class: Insecta; order: Diptera; family: Tachinidae; genus: Hyphantrophaga; specificEpithet: virilis; scientificNameAuthorship: (Aldrich & Webber, 1924); **Location:** country: Costa Rica; countryCode: CR; stateProvince: Guanacaste; county: Sector Santa Rosa; locality: Area de Conservacion Guanacaste; verbatimLocality: Sendero Natural; verbatimElevation: 290; verbatimLatitude: 10.8357; verbatimLongitude: -85.6125; verbatimCoordinateSystem: Decimal; decimalLatitude: 10.8357; decimalLongitude: -85.6125; **Event:** samplingProtocol: Reared from the larva of the Geometridae, see description; verbatimEventDate: 19-Aug-2008; **Record Level:** language: en; institutionCode: CNC; collectionCode: Insects; basisOfRecord: Pinned Specimen
- **Type status:** Other material. **Occurrence:** occurrenceDetails: http://janzen.sas.upenn.edu; catalogNumber: DHJPAR0029762; recordedBy: D.H. Janzen, W. Hallwachs & Guillermo Pereira; individualID: DHJPAR0029762; individualCount: 1; sex: male; lifeStage: adult; preparations: pinned; otherCatalogNumbers: ASHYM1183-09, 08-SRNP-14503, BOLD:AAA1577; **Taxon:** scientificName: Hyphantrophaga virilis; phylum: Arthropoda; class: Insecta; order: Diptera; family: Tachinidae; genus: Hyphantrophaga; specificEpithet: virilis; scientificNameAuthorship: (Aldrich & Webber, 1924); **Location:** country: Costa Rica; countryCode: CR; stateProvince: Guanacaste; county: Sector Santa Rosa; locality: Area de Conservacion Guanacaste; verbatimLocality: Sendero Natural; verbatimElevation: 290; verbatimLatitude: 10.8357; verbatimLongitude: -85.6125; verbatimCoordinateSystem: Decimal; decimalLatitude: 10.8357; decimalLongitude: -85.6125; **Event:** samplingProtocol: Reared from the larva of the Notodontidae, Boriza tonac; verbatimEventDate: 23-Aug-2008; **Record Level:** language: en; institutionCode: CNC; collectionCode: Insects; basisOfRecord: Pinned Specimen
- **Type status:** Other material. **Occurrence:** occurrenceDetails: http://janzen.sas.upenn.edu; catalogNumber: DHJPAR0029767; recordedBy: D.H. Janzen, W. Hallwachs & Guillermo Pereira; individualID: DHJPAR0029767; individualCount: 1; sex: male; lifeStage: adult; preparations: pinned; otherCatalogNumbers: ASHYM1188-09, 08-SRNP-14562, BOLD:AAA1577; **Taxon:** scientificName: Hyphantrophaga virilis; phylum: Arthropoda; class: Insecta; order: Diptera; family: Tachinidae; genus: Hyphantrophaga; specificEpithet: virilis; scientificNameAuthorship: (Aldrich & Webber, 1924); **Location:** country: Costa Rica; countryCode: CR; stateProvince: Guanacaste; county: Sector Santa Rosa; locality: Area de Conservacion Guanacaste; verbatimLocality: Bosque San Emilio; verbatimElevation: 300; verbatimLatitude: 10.8439; verbatimLongitude: -85.6138; verbatimCoordinateSystem: Decimal; decimalLatitude: 10.8439; decimalLongitude: -85.6138; **Event:** samplingProtocol: Reared from the larva of the Notodontidae, Elasmia mandela; verbatimEventDate: 24-Aug-2008; **Record Level:** language: en; institutionCode: CNC; collectionCode: Insects; basisOfRecord: Pinned Specimen
- **Type status:** Other material. **Occurrence:** occurrenceDetails: http://janzen.sas.upenn.edu; catalogNumber: DHJPAR0030074; recordedBy: D.H. Janzen, W. Hallwachs & Johan Vargas; individualID: DHJPAR0030074; individualCount: 1; sex: male; lifeStage: adult; preparations: pinned; otherCatalogNumbers: ASHYB818-09, 08-SRNP-16975, BOLD:AAA1577; **Taxon:** scientificName: Hyphantrophaga virilis; phylum: Arthropoda; class: Insecta; order: Diptera; family: Tachinidae; genus: Hyphantrophaga; specificEpithet: virilis; scientificNameAuthorship: (Aldrich & Webber, 1924); **Location:** country: Costa Rica; countryCode: CR; stateProvince: Guanacaste; county: Sector Santa Rosa; locality: Area de Conservacion Guanacaste; verbatimLocality: Bosque Humedo; verbatimElevation: 290; verbatimLatitude: 10.8514; verbatimLongitude: -85.608; verbatimCoordinateSystem: Decimal; decimalLatitude: 10.8514; decimalLongitude: -85.608; **Event:** samplingProtocol: Reared from the larva of the Nymphalidae, Mechanitis isthmia; verbatimEventDate: 16-Jan-2009; **Record Level:** language: en; institutionCode: CNC; collectionCode: Insects; basisOfRecord: Pinned Specimen
- **Type status:** Other material. **Occurrence:** occurrenceDetails: http://janzen.sas.upenn.edu; catalogNumber: DHJPAR0030078; recordedBy: D.H. Janzen, W. Hallwachs & Lucia Vargas; individualID: DHJPAR0030078; individualCount: 1; sex: male; lifeStage: adult; preparations: pinned; otherCatalogNumbers: ASHYB822-09, 08-SRNP-16895, BOLD:AAA1577; **Taxon:** scientificName: Hyphantrophaga virilis; phylum: Arthropoda; class: Insecta; order: Diptera; family: Tachinidae; genus: Hyphantrophaga; specificEpithet: virilis; scientificNameAuthorship: (Aldrich & Webber, 1924); **Location:** country: Costa Rica; countryCode: CR; stateProvince: Guanacaste; county: Sector Santa Rosa; locality: Area de Conservacion Guanacaste; verbatimLocality: Cafetal; verbatimElevation: 280; verbatimLatitude: 10.8583; verbatimLongitude: -85.6109; verbatimCoordinateSystem: Decimal; decimalLatitude: 10.8583; decimalLongitude: -85.6109; **Event:** samplingProtocol: Reared from the larva of the Hesperiidae, Celaenorrhinus eligius; verbatimEventDate: 31-Dec-2008; **Record Level:** language: en; institutionCode: CNC; collectionCode: Insects; basisOfRecord: Pinned Specimen
- **Type status:** Other material. **Occurrence:** occurrenceDetails: http://janzen.sas.upenn.edu; catalogNumber: DHJPAR0030080; recordedBy: D.H. Janzen, W. Hallwachs & Guillermo Pereira; individualID: DHJPAR0030080; individualCount: 1; sex: male; lifeStage: adult; preparations: pinned; otherCatalogNumbers: ASHYB824-09, 08-SRNP-16271, BOLD:AAA1577; **Taxon:** scientificName: Hyphantrophaga virilis; phylum: Arthropoda; class: Insecta; order: Diptera; family: Tachinidae; genus: Hyphantrophaga; specificEpithet: virilis; scientificNameAuthorship: (Aldrich & Webber, 1924); **Location:** country: Costa Rica; countryCode: CR; stateProvince: Guanacaste; county: Sector Santa Rosa; locality: Area de Conservacion Guanacaste; verbatimLocality: Bosque San Emilio; verbatimElevation: 300; verbatimLatitude: 10.8439; verbatimLongitude: -85.6138; verbatimCoordinateSystem: Decimal; decimalLatitude: 10.8439; decimalLongitude: -85.6138; **Event:** samplingProtocol: Reared from the larva of the Hesperiidae, Mysoria ambigua; verbatimEventDate: 10-Dec-2008; **Record Level:** language: en; institutionCode: CNC; collectionCode: Insects; basisOfRecord: Pinned Specimen
- **Type status:** Other material. **Occurrence:** occurrenceDetails: http://janzen.sas.upenn.edu; catalogNumber: DHJPAR0040981; recordedBy: D.H. Janzen, W. Hallwachs & Roster Moraga; individualID: DHJPAR0040981; individualCount: 1; sex: male; lifeStage: adult; preparations: pinned; otherCatalogNumbers: ASHYF896-11, 10-SRNP-22425, BOLD:AAA1577; **Taxon:** scientificName: Hyphantrophaga virilis; phylum: Arthropoda; class: Insecta; order: Diptera; family: Tachinidae; genus: Hyphantrophaga; specificEpithet: virilis; scientificNameAuthorship: (Aldrich & Webber, 1924); **Location:** country: Costa Rica; countryCode: CR; stateProvince: Guanacaste; county: Sector Del Oro; locality: Area de Conservacion Guanacaste; verbatimLocality: Monte Cristo; verbatimElevation: 525; verbatimLatitude: 11.0137; verbatimLongitude: -85.4253; verbatimCoordinateSystem: Decimal; decimalLatitude: 11.0137; decimalLongitude: -85.4253; **Event:** samplingProtocol: Reared from the larva of the Hesperiidae, Cynea anthracinus; verbatimEventDate: 14-Dec-2010; **Record Level:** language: en; institutionCode: CNC; collectionCode: Insects; basisOfRecord: Pinned Specimen
- **Type status:** Other material. **Occurrence:** occurrenceDetails: http://janzen.sas.upenn.edu; catalogNumber: DHJPAR0040990; recordedBy: D.H. Janzen, W. Hallwachs & Mariano Pereira; individualID: DHJPAR0040990; individualCount: 1; sex: male; lifeStage: adult; preparations: pinned; otherCatalogNumbers: ASHYF905-11, 10-SRNP-56976, BOLD:AAA1577; **Taxon:** scientificName: Hyphantrophaga virilis; phylum: Arthropoda; class: Insecta; order: Diptera; family: Tachinidae; genus: Hyphantrophaga; specificEpithet: virilis; scientificNameAuthorship: (Aldrich & Webber, 1924); **Location:** country: Costa Rica; countryCode: CR; stateProvince: Guanacaste; county: Sector Mundo Nuevo; locality: Area de Conservacion Guanacaste; verbatimLocality: Punta Plancha; verbatimElevation: 420; verbatimLatitude: 10.7416; verbatimLongitude: -85.4273; verbatimCoordinateSystem: Decimal; decimalLatitude: 10.7416; decimalLongitude: -85.4273; **Event:** samplingProtocol: Reared from the larva of the Pyralidae, Carthara abruptaDHJ02; verbatimEventDate: 16-Nov-2010; **Record Level:** language: en; institutionCode: CNC; collectionCode: Insects; basisOfRecord: Pinned Specimen
- **Type status:** Other material. **Occurrence:** occurrenceDetails: http://janzen.sas.upenn.edu; catalogNumber: DHJPAR0046535; recordedBy: D.H. Janzen, W. Hallwachs & Mariano Pereira; individualID: DHJPAR0046535; individualCount: 1; sex: male; lifeStage: adult; preparations: pinned; otherCatalogNumbers: ACGBA708-12, 11-SRNP-56306, BOLD:AAA1577; **Taxon:** scientificName: Hyphantrophaga virilis; phylum: Arthropoda; class: Insecta; order: Diptera; family: Tachinidae; genus: Hyphantrophaga; specificEpithet: virilis; scientificNameAuthorship: (Aldrich & Webber, 1924); **Location:** country: Costa Rica; countryCode: CR; stateProvince: Guanacaste; county: Sector Mundo Nuevo; locality: Area de Conservacion Guanacaste; verbatimLocality: Quebrada Tibio Perla; verbatimElevation: 330; verbatimLatitude: 10.7626; verbatimLongitude: -85.4298; verbatimCoordinateSystem: Decimal; decimalLatitude: 10.7626; decimalLongitude: -85.4298; **Event:** samplingProtocol: Reared from the larva of the Nolidae, Iscadia purpurascens; verbatimEventDate: 01-Sep-2011; **Record Level:** language: en; institutionCode: CNC; collectionCode: Insects; basisOfRecord: Pinned Specimen
- **Type status:** Other material. **Occurrence:** occurrenceDetails: http://janzen.sas.upenn.edu; catalogNumber: DHJPAR0006302; recordedBy: D.H. Janzen, W. Hallwachs & Freddy Quesada; individualID: DHJPAR0006302; individualCount: 1; sex: female; lifeStage: adult; preparations: pinned; otherCatalogNumbers: ASTAI730-06, 05-SRNP-60214, BOLD:AAA1577; **Taxon:** scientificName: Hyphantrophaga virilis; phylum: Arthropoda; class: Insecta; order: Diptera; family: Tachinidae; genus: Hyphantrophaga; specificEpithet: virilis; scientificNameAuthorship: (Aldrich & Webber, 1924); **Location:** country: Costa Rica; countryCode: CR; stateProvince: Guanacaste; county: Sector Santa Rosa; locality: Area de Conservacion Guanacaste; verbatimLocality: Finca Jenny; verbatimElevation: 205; verbatimLatitude: 10.8633; verbatimLongitude: -85.5744; verbatimCoordinateSystem: Decimal; decimalLatitude: 10.8633; decimalLongitude: -85.5744; **Event:** samplingProtocol: Reared from the larva of the Notodontidae, Tachuda plumipesICG02; verbatimEventDate: 25-Sep-2005; **Record Level:** language: en; institutionCode: CNC; collectionCode: Insects; basisOfRecord: Pinned Specimen
- **Type status:** Other material. **Occurrence:** occurrenceDetails: http://janzen.sas.upenn.edu; catalogNumber: DHJPAR0006309; recordedBy: D.H. Janzen, W. Hallwachs & Harry Ramirez; individualID: DHJPAR0006309; individualCount: 1; sex: female; lifeStage: adult; preparations: pinned; otherCatalogNumbers: ASTAI737-06, 02-SRNP-24305, BOLD:AAA1577; **Taxon:** scientificName: Hyphantrophaga virilis; phylum: Arthropoda; class: Insecta; order: Diptera; family: Tachinidae; genus: Hyphantrophaga; specificEpithet: virilis; scientificNameAuthorship: (Aldrich & Webber, 1924); **Location:** country: Costa Rica; countryCode: CR; stateProvince: Guanacaste; county: Sector Cacao; locality: Area de Conservacion Guanacaste; verbatimLocality: Cuesta Caimito; verbatimElevation: 640; verbatimLatitude: 10.8908; verbatimLongitude: -85.4719; verbatimCoordinateSystem: Decimal; decimalLatitude: 10.8908; decimalLongitude: -85.4719; **Event:** samplingProtocol: Reared from the larva of the Noctuidae, Dyops cuprescens; verbatimEventDate: 13-Dec-2002; **Record Level:** language: en; institutionCode: CNC; collectionCode: Insects; basisOfRecord: Pinned Specimen
- **Type status:** Other material. **Occurrence:** occurrenceDetails: http://janzen.sas.upenn.edu; catalogNumber: DHJPAR0006322; recordedBy: D.H. Janzen, W. Hallwachs & Ruth Franco; individualID: DHJPAR0006322; individualCount: 1; sex: female; lifeStage: adult; preparations: pinned; otherCatalogNumbers: ASTAI750-06, 03-SRNP-14269,; **Taxon:** scientificName: Hyphantrophaga virilis; phylum: Arthropoda; class: Insecta; order: Diptera; family: Tachinidae; genus: Hyphantrophaga; specificEpithet: virilis; scientificNameAuthorship: (Aldrich & Webber, 1924); **Location:** country: Costa Rica; countryCode: CR; stateProvince: Guanacaste; county: Sector Santa Rosa; locality: Area de Conservacion Guanacaste; verbatimLocality: Area Administrativa; verbatimElevation: 295; verbatimLatitude: 10.8376; verbatimLongitude: -85.6187; verbatimCoordinateSystem: Decimal; decimalLatitude: 10.8376; decimalLongitude: -85.6187; **Event:** samplingProtocol: Reared from the larva of the Notodontidae, Sericochroa felderi; verbatimEventDate: 12-Aug-2003; **Record Level:** language: en; institutionCode: CNC; collectionCode: Insects; basisOfRecord: Pinned Specimen
- **Type status:** Other material. **Occurrence:** occurrenceDetails: http://janzen.sas.upenn.edu; catalogNumber: DHJPAR0019577; recordedBy: D.H. Janzen, W. Hallwachs & Mariano Pereira; individualID: DHJPAR0019577; individualCount: 1; sex: female; lifeStage: adult; preparations: pinned; otherCatalogNumbers: ASTAB125-07, 07-SRNP-55882, BOLD:AAA1577; **Taxon:** scientificName: Hyphantrophaga virilis; phylum: Arthropoda; class: Insecta; order: Diptera; family: Tachinidae; genus: Hyphantrophaga; specificEpithet: virilis; scientificNameAuthorship: (Aldrich & Webber, 1924); **Location:** country: Costa Rica; countryCode: CR; stateProvince: Guanacaste; county: Sector Mundo Nuevo; locality: Area de Conservacion Guanacaste; verbatimLocality: Quebrada Tibio Perla; verbatimElevation: 330; verbatimLatitude: 10.7626; verbatimLongitude: -85.4298; verbatimCoordinateSystem: Decimal; decimalLatitude: 10.7626; decimalLongitude: -85.4298; **Event:** samplingProtocol: Reared from the larva of the Noctuidae, Neotuerta sabulosa; verbatimEventDate: 06-Jun-2007; **Record Level:** language: en; institutionCode: CNC; collectionCode: Insects; basisOfRecord: Pinned Specimen
- **Type status:** Other material. **Occurrence:** occurrenceDetails: http://janzen.sas.upenn.edu; catalogNumber: DHJPAR0019602; recordedBy: D.H. Janzen, W. Hallwachs & Jose Alberto Sanchez; individualID: DHJPAR0019602; individualCount: 1; sex: female; lifeStage: adult; preparations: pinned; otherCatalogNumbers: ASTAB150-07, 07-SRNP-56934, BOLD:AAA1577; **Taxon:** scientificName: Hyphantrophaga virilis; phylum: Arthropoda; class: Insecta; order: Diptera; family: Tachinidae; genus: Hyphantrophaga; specificEpithet: virilis; scientificNameAuthorship: (Aldrich & Webber, 1924); **Location:** country: Costa Rica; countryCode: CR; stateProvince: Guanacaste; county: Sector Mundo Nuevo; locality: Area de Conservacion Guanacaste; verbatimLocality: Quebrada Tibio Perla; verbatimElevation: 330; verbatimLatitude: 10.7626; verbatimLongitude: -85.4298; verbatimCoordinateSystem: Decimal; decimalLatitude: 10.7626; decimalLongitude: -85.4298; **Event:** samplingProtocol: Reared from the larva of the Riodinidae, Melanis sanguinea; verbatimEventDate: 20-Jun-2007; **Record Level:** language: en; institutionCode: CNC; collectionCode: Insects; basisOfRecord: Pinned Specimen
- **Type status:** Other material. **Occurrence:** occurrenceDetails: http://janzen.sas.upenn.edu; catalogNumber: DHJPAR0023210; recordedBy: D.H. Janzen, W. Hallwachs & Guillermo Pereira; individualID: DHJPAR0023210; individualCount: 1; sex: female; lifeStage: adult; preparations: pinned; otherCatalogNumbers: ASTAW371-08, 07-SRNP-16103, BOLD:AAA1577; **Taxon:** scientificName: Hyphantrophaga virilis; phylum: Arthropoda; class: Insecta; order: Diptera; family: Tachinidae; genus: Hyphantrophaga; specificEpithet: virilis; scientificNameAuthorship: (Aldrich & Webber, 1924); **Location:** country: Costa Rica; countryCode: CR; stateProvince: Guanacaste; county: Sector Santa Rosa; locality: Area de Conservacion Guanacaste; verbatimLocality: Rio Azufrado; verbatimElevation: 95; verbatimLatitude: 10.8122; verbatimLongitude: -85.5444; verbatimCoordinateSystem: Decimal; decimalLatitude: 10.8122; decimalLongitude: -85.5444; **Event:** samplingProtocol: Reared from the larva of the Notodontidae, Calledema plusia; verbatimEventDate: 23-Nov-2007; **Record Level:** language: en; institutionCode: CNC; collectionCode: Insects; basisOfRecord: Pinned Specimen
- **Type status:** Other material. **Occurrence:** occurrenceDetails: http://janzen.sas.upenn.edu; catalogNumber: DHJPAR0023223; recordedBy: D.H. Janzen, W. Hallwachs & Jose Alberto Sanchez; individualID: DHJPAR0023223; individualCount: 1; sex: female; lifeStage: adult; preparations: pinned; otherCatalogNumbers: ASTAW384-08, 07-SRNP-60273, BOLD:AAA1577; **Taxon:** scientificName: Hyphantrophaga virilis; phylum: Arthropoda; class: Insecta; order: Diptera; family: Tachinidae; genus: Hyphantrophaga; specificEpithet: virilis; scientificNameAuthorship: (Aldrich & Webber, 1924); **Location:** country: Costa Rica; countryCode: CR; stateProvince: Guanacaste; county: Sector Mundo Nuevo; locality: Area de Conservacion Guanacaste; verbatimLocality: Sendero Aguacate; verbatimElevation: 335; verbatimLatitude: 10.769; verbatimLongitude: -85.4346; verbatimCoordinateSystem: Decimal; decimalLatitude: 10.769; decimalLongitude: -85.4346; **Event:** samplingProtocol: Reared from the larva of the Hesperiidae, Mysoria ambigua; verbatimEventDate: 26-Nov-2007; **Record Level:** language: en; institutionCode: CNC; collectionCode: Insects; basisOfRecord: Pinned Specimen
- **Type status:** Other material. **Occurrence:** occurrenceDetails: http://janzen.sas.upenn.edu; catalogNumber: DHJPAR0023617; recordedBy: D.H. Janzen, W. Hallwachs & Lucia Rios; individualID: DHJPAR0023617; individualCount: 1; sex: female; lifeStage: adult; preparations: pinned; otherCatalogNumbers: ASTAW474-08, 08-SRNP-20258, BOLD:AAA1577; **Taxon:** scientificName: Hyphantrophaga virilis; phylum: Arthropoda; class: Insecta; order: Diptera; family: Tachinidae; genus: Hyphantrophaga; specificEpithet: virilis; scientificNameAuthorship: (Aldrich & Webber, 1924); **Location:** country: Costa Rica; countryCode: CR; stateProvince: Guanacaste; county: Sector Del Oro; locality: Area de Conservacion Guanacaste; verbatimLocality: Bosque Aguirre; verbatimElevation: 620; verbatimLatitude: 11.0006; verbatimLongitude: -85.438; verbatimCoordinateSystem: Decimal; decimalLatitude: 11.0006; decimalLongitude: -85.438; **Event:** samplingProtocol: Reared from the larva of the Pieridae, Eurema xanthochlora; verbatimEventDate: 05-Feb-2008; **Record Level:** language: en; institutionCode: CNC; collectionCode: Insects; basisOfRecord: Pinned Specimen
- **Type status:** Other material. **Occurrence:** occurrenceDetails: http://janzen.sas.upenn.edu; catalogNumber: DHJPAR0023619; recordedBy: D.H. Janzen, W. Hallwachs & Lucia Rios; individualID: DHJPAR0023619; individualCount: 1; sex: female; lifeStage: adult; preparations: pinned; otherCatalogNumbers: ASTAW476-08, 08-SRNP-20268, BOLD:AAA1577; **Taxon:** scientificName: Hyphantrophaga virilis; phylum: Arthropoda; class: Insecta; order: Diptera; family: Tachinidae; genus: Hyphantrophaga; specificEpithet: virilis; scientificNameAuthorship: (Aldrich & Webber, 1924); **Location:** country: Costa Rica; countryCode: CR; stateProvince: Guanacaste; county: Sector Del Oro; locality: Area de Conservacion Guanacaste; verbatimLocality: Bosque Aguirre; verbatimElevation: 620; verbatimLatitude: 11.0006; verbatimLongitude: -85.438; verbatimCoordinateSystem: Decimal; decimalLatitude: 11.0006; decimalLongitude: -85.438; **Event:** samplingProtocol: Reared from the larva of the Pieridae, Eurema xanthochlora; verbatimEventDate: 05-Feb-2008; **Record Level:** language: en; institutionCode: CNC; collectionCode: Insects; basisOfRecord: Pinned Specimen
- **Type status:** Other material. **Occurrence:** occurrenceDetails: http://janzen.sas.upenn.edu; catalogNumber: DHJPAR0023620; recordedBy: D.H. Janzen, W. Hallwachs & Lucia Rios; individualID: DHJPAR0023620; individualCount: 1; sex: female; lifeStage: adult; preparations: pinned; otherCatalogNumbers: ASTAW477-08, 08-SRNP-20264, BOLD:AAA1577; **Taxon:** scientificName: Hyphantrophaga virilis; phylum: Arthropoda; class: Insecta; order: Diptera; family: Tachinidae; genus: Hyphantrophaga; specificEpithet: virilis; scientificNameAuthorship: (Aldrich & Webber, 1924); **Location:** country: Costa Rica; countryCode: CR; stateProvince: Guanacaste; county: Sector Del Oro; locality: Area de Conservacion Guanacaste; verbatimLocality: Bosque Aguirre; verbatimElevation: 620; verbatimLatitude: 11.0006; verbatimLongitude: -85.438; verbatimCoordinateSystem: Decimal; decimalLatitude: 11.0006; decimalLongitude: -85.438; **Event:** samplingProtocol: Reared from the larva of the Pieridae, Eurema xanthochlora; verbatimEventDate: 05-Feb-2008; **Record Level:** language: en; institutionCode: CNC; collectionCode: Insects; basisOfRecord: Pinned Specimen
- **Type status:** Other material. **Occurrence:** occurrenceDetails: http://janzen.sas.upenn.edu; catalogNumber: DHJPAR0023634; recordedBy: D.H. Janzen, W. Hallwachs & Lucia Rios; individualID: DHJPAR0023634; individualCount: 1; sex: female; lifeStage: adult; preparations: pinned; otherCatalogNumbers: ASTAW491-08, 08-SRNP-20524, BOLD:AAA1577; **Taxon:** scientificName: Hyphantrophaga virilis; phylum: Arthropoda; class: Insecta; order: Diptera; family: Tachinidae; genus: Hyphantrophaga; specificEpithet: virilis; scientificNameAuthorship: (Aldrich & Webber, 1924); **Location:** country: Costa Rica; countryCode: CR; stateProvince: Guanacaste; county: Sector Del Oro; locality: Area de Conservacion Guanacaste; verbatimLocality: Bosque Aguirre; verbatimElevation: 620; verbatimLatitude: 11.0006; verbatimLongitude: -85.438; verbatimCoordinateSystem: Decimal; decimalLatitude: 11.0006; decimalLongitude: -85.438; **Event:** samplingProtocol: Reared from the larva of the Hesperiidae, Astraptes inflatio; verbatimEventDate: 09-Mar-2008; **Record Level:** language: en; institutionCode: CNC; collectionCode: Insects; basisOfRecord: Pinned Specimen
- **Type status:** Other material. **Occurrence:** occurrenceDetails: http://janzen.sas.upenn.edu; catalogNumber: DHJPAR0023639; recordedBy: D.H. Janzen, W. Hallwachs & Harry Ramirez; individualID: DHJPAR0023639; individualCount: 1; sex: female; lifeStage: adult; preparations: pinned; otherCatalogNumbers: ASTAW496-08, 08-SRNP-45029, BOLD:AAA1577; **Taxon:** scientificName: Hyphantrophaga virilis; phylum: Arthropoda; class: Insecta; order: Diptera; family: Tachinidae; genus: Hyphantrophaga; specificEpithet: virilis; scientificNameAuthorship: (Aldrich & Webber, 1924); **Location:** country: Costa Rica; countryCode: CR; stateProvince: Guanacaste; county: Sector Cacao; locality: Area de Conservacion Guanacaste; verbatimLocality: Sendero Pajarito; verbatimElevation: 600; verbatimLatitude: 10.8899; verbatimLongitude: -85.4743; verbatimCoordinateSystem: Decimal; decimalLatitude: 10.8899; decimalLongitude: -85.4743; **Event:** samplingProtocol: Reared from the larva of the Lasiocampidae, Euglyphis jessiehillae; verbatimEventDate: 13-Mar-2008; **Record Level:** language: en; institutionCode: CNC; collectionCode: Insects; basisOfRecord: Pinned Specimen
- **Type status:** Other material. **Occurrence:** occurrenceDetails: http://janzen.sas.upenn.edu; catalogNumber: DHJPAR0023660; recordedBy: D.H. Janzen, W. Hallwachs & Anabelle Cordoba; individualID: DHJPAR0023660; individualCount: 1; sex: female; lifeStage: adult; preparations: pinned; otherCatalogNumbers: ASTAW517-08, 07-SRNP-5016, BOLD:AAA1577; **Taxon:** scientificName: Hyphantrophaga virilis; phylum: Arthropoda; class: Insecta; order: Diptera; family: Tachinidae; genus: Hyphantrophaga; specificEpithet: virilis; scientificNameAuthorship: (Aldrich & Webber, 1924); **Location:** country: Costa Rica; countryCode: CR; stateProvince: Alajuela; county: Sector San Cristobal; locality: Area de Conservacion Guanacaste; verbatimLocality: Finca San Gabriel; verbatimElevation: 645; verbatimLatitude: 10.8777; verbatimLongitude: -85.3934; verbatimCoordinateSystem: Decimal; decimalLatitude: 10.8777; decimalLongitude: -85.3934; **Event:** samplingProtocol: Reared from the larva of the Pieridae, Dismorphia praxinoe; verbatimEventDate: 15-Jan-2008; **Record Level:** language: en; institutionCode: CNC; collectionCode: Insects; basisOfRecord: Pinned Specimen
- **Type status:** Other material. **Occurrence:** occurrenceDetails: http://janzen.sas.upenn.edu; catalogNumber: DHJPAR0024460; recordedBy: D.H. Janzen, W. Hallwachs & Anabelle Cordoba; individualID: DHJPAR0024460; individualCount: 1; sex: female; lifeStage: adult; preparations: pinned; otherCatalogNumbers: ASTAW570-08, 08-SRNP-351, BOLD:AAA1577; **Taxon:** scientificName: Hyphantrophaga virilis; phylum: Arthropoda; class: Insecta; order: Diptera; family: Tachinidae; genus: Hyphantrophaga; specificEpithet: virilis; scientificNameAuthorship: (Aldrich & Webber, 1924); **Location:** country: Costa Rica; countryCode: CR; stateProvince: Alajuela; county: Sector San Cristobal; locality: Area de Conservacion Guanacaste; verbatimLocality: Puente Palma; verbatimElevation: 460; verbatimLatitude: 10.9163; verbatimLongitude: -85.3787; verbatimCoordinateSystem: Decimal; decimalLatitude: 10.9163; decimalLongitude: -85.3787; **Event:** samplingProtocol: Reared from the larva of the Hesperiidae, Polyctor cleta; verbatimEventDate: 14-Mar-2008; **Record Level:** language: en; institutionCode: CNC; collectionCode: Insects; basisOfRecord: Pinned Specimen
- **Type status:** Other material. **Occurrence:** occurrenceDetails: http://janzen.sas.upenn.edu; catalogNumber: DHJPAR0024470; recordedBy: D.H. Janzen, W. Hallwachs & Gloria Sihezar; individualID: DHJPAR0024470; individualCount: 1; sex: female; lifeStage: adult; preparations: pinned; otherCatalogNumbers: ASTAW580-08, 08-SRNP-767, BOLD:AAA1577; **Taxon:** scientificName: Hyphantrophaga virilis; phylum: Arthropoda; class: Insecta; order: Diptera; family: Tachinidae; genus: Hyphantrophaga; specificEpithet: virilis; scientificNameAuthorship: (Aldrich & Webber, 1924); **Location:** country: Costa Rica; countryCode: CR; stateProvince: Alajuela; county: Sector San Cristobal; locality: Area de Conservacion Guanacaste; verbatimLocality: Vado Rio Cucaracho; verbatimElevation: 640; verbatimLatitude: 10.8702; verbatimLongitude: -85.3915; verbatimCoordinateSystem: Decimal; decimalLatitude: 10.8702; decimalLongitude: -85.3915; **Event:** samplingProtocol: Reared from the larva of the Notodontidae, Tithraustes noctilucesICG02; verbatimEventDate: 21-Mar-2008; **Record Level:** language: en; institutionCode: CNC; collectionCode: Insects; basisOfRecord: Pinned Specimen
- **Type status:** Other material. **Occurrence:** occurrenceDetails: http://janzen.sas.upenn.edu; catalogNumber: DHJPAR0024472; recordedBy: D.H. Janzen, W. Hallwachs & Elda Araya; individualID: DHJPAR0024472; individualCount: 1; sex: female; lifeStage: adult; preparations: pinned; otherCatalogNumbers: ASTAW582-08, 08-SRNP-508, BOLD:AAA1577; **Taxon:** scientificName: Hyphantrophaga virilis; phylum: Arthropoda; class: Insecta; order: Diptera; family: Tachinidae; genus: Hyphantrophaga; specificEpithet: virilis; scientificNameAuthorship: (Aldrich & Webber, 1924); **Location:** country: Costa Rica; countryCode: CR; stateProvince: Alajuela; county: Sector San Cristobal; locality: Area de Conservacion Guanacaste; verbatimLocality: Finca San Gabriel; verbatimElevation: 645; verbatimLatitude: 10.8777; verbatimLongitude: -85.3934; verbatimCoordinateSystem: Decimal; decimalLatitude: 10.8777; decimalLongitude: -85.3934; **Event:** samplingProtocol: Reared from the larva of the Tortricidae, Anacrusis nephrodes; verbatimEventDate: 20-Mar-2008; **Record Level:** language: en; institutionCode: CNC; collectionCode: Insects; basisOfRecord: Pinned Specimen
- **Type status:** Other material. **Occurrence:** occurrenceDetails: http://janzen.sas.upenn.edu; catalogNumber: DHJPAR0024475; recordedBy: D.H. Janzen, W. Hallwachs & Anabelle Cordoba; individualID: DHJPAR0024475; individualCount: 1; sex: female; lifeStage: adult; preparations: pinned; otherCatalogNumbers: ASTAW585-08, 08-SRNP-349, BOLD:AAA1577; **Taxon:** scientificName: Hyphantrophaga virilis; phylum: Arthropoda; class: Insecta; order: Diptera; family: Tachinidae; genus: Hyphantrophaga; specificEpithet: virilis; scientificNameAuthorship: (Aldrich & Webber, 1924); **Location:** country: Costa Rica; countryCode: CR; stateProvince: Alajuela; county: Sector San Cristobal; locality: Area de Conservacion Guanacaste; verbatimLocality: Puente Palma; verbatimElevation: 460; verbatimLatitude: 10.9163; verbatimLongitude: -85.3787; verbatimCoordinateSystem: Decimal; decimalLatitude: 10.9163; decimalLongitude: -85.3787; **Event:** samplingProtocol: Reared from the larva of the Hesperiidae, Polyctor cleta; verbatimEventDate: 06-Mar-2008; **Record Level:** language: en; institutionCode: CNC; collectionCode: Insects; basisOfRecord: Pinned Specimen
- **Type status:** Other material. **Occurrence:** occurrenceDetails: http://janzen.sas.upenn.edu; catalogNumber: DHJPAR0024561; recordedBy: D.H. Janzen, W. Hallwachs & Manuel Pereira; individualID: DHJPAR0024561; individualCount: 1; sex: female; lifeStage: adult; preparations: pinned; otherCatalogNumbers: ASTAW671-08, 08-SRNP-35348, BOLD:AAA1577; **Taxon:** scientificName: Hyphantrophaga virilis; phylum: Arthropoda; class: Insecta; order: Diptera; family: Tachinidae; genus: Hyphantrophaga; specificEpithet: virilis; scientificNameAuthorship: (Aldrich & Webber, 1924); **Location:** country: Costa Rica; countryCode: CR; stateProvince: Guanacaste; county: Sector Cacao; locality: Area de Conservacion Guanacaste; verbatimLocality: Sendero Derrumbe; verbatimElevation: 1220; verbatimLatitude: 10.9292; verbatimLongitude: -85.4643; verbatimCoordinateSystem: Decimal; decimalLatitude: 10.9292; decimalLongitude: -85.4643; **Event:** samplingProtocol: Reared from the larva of the Geometridae, Carpela Janzen01; verbatimEventDate: 10-May-2008; **Record Level:** language: en; institutionCode: CNC; collectionCode: Insects; basisOfRecord: Pinned Specimen
- **Type status:** Other material. **Occurrence:** occurrenceDetails: http://janzen.sas.upenn.edu; catalogNumber: DHJPAR0029756; recordedBy: D.H. Janzen, W. Hallwachs & Guillermo Pereira; individualID: DHJPAR0029756; individualCount: 1; sex: female; lifeStage: adult; preparations: pinned; otherCatalogNumbers: ASHYM1177-09, 08-SRNP-14508,; **Taxon:** scientificName: Hyphantrophaga virilis; phylum: Arthropoda; class: Insecta; order: Diptera; family: Tachinidae; genus: Hyphantrophaga; specificEpithet: virilis; scientificNameAuthorship: (Aldrich & Webber, 1924); **Location:** country: Costa Rica; countryCode: CR; stateProvince: Guanacaste; county: Sector Santa Rosa; locality: Area de Conservacion Guanacaste; verbatimLocality: Sendero Natural; verbatimElevation: 290; verbatimLatitude: 10.8357; verbatimLongitude: -85.6125; verbatimCoordinateSystem: Decimal; decimalLatitude: 10.8357; decimalLongitude: -85.6125; **Event:** samplingProtocol: Reared from the larva of the Notodontidae, Elasmia mandela; verbatimEventDate: 22-Aug-2008; **Record Level:** language: en; institutionCode: CNC; collectionCode: Insects; basisOfRecord: Pinned Specimen
- **Type status:** Other material. **Occurrence:** occurrenceDetails: http://janzen.sas.upenn.edu; catalogNumber: DHJPAR0029758; recordedBy: D.H. Janzen, W. Hallwachs & Guillermo Pereira; individualID: DHJPAR0029758; individualCount: 1; sex: female; lifeStage: adult; preparations: pinned; otherCatalogNumbers: ASHYM1179-09, 08-SRNP-14565, BOLD:AAA1577; **Taxon:** scientificName: Hyphantrophaga virilis; phylum: Arthropoda; class: Insecta; order: Diptera; family: Tachinidae; genus: Hyphantrophaga; specificEpithet: virilis; scientificNameAuthorship: (Aldrich & Webber, 1924); **Location:** country: Costa Rica; countryCode: CR; stateProvince: Guanacaste; county: Sector Santa Rosa; locality: Area de Conservacion Guanacaste; verbatimLocality: Bosque San Emilio; verbatimElevation: 300; verbatimLatitude: 10.8439; verbatimLongitude: -85.6138; verbatimCoordinateSystem: Decimal; decimalLatitude: 10.8439; decimalLongitude: -85.6138; **Event:** samplingProtocol: Reared from the larva of the Notodontidae, Elasmia mandela; verbatimEventDate: 23-Aug-2008; **Record Level:** language: en; institutionCode: CNC; collectionCode: Insects; basisOfRecord: Pinned Specimen
- **Type status:** Other material. **Occurrence:** occurrenceDetails: http://janzen.sas.upenn.edu; catalogNumber: DHJPAR0029760; recordedBy: D.H. Janzen, W. Hallwachs & Guillermo Pereira; individualID: DHJPAR0029760; individualCount: 1; sex: female; lifeStage: adult; preparations: pinned; otherCatalogNumbers: ASHYM1181-09, 08-SRNP-14561, BOLD:AAA1577; **Taxon:** scientificName: Hyphantrophaga virilis; phylum: Arthropoda; class: Insecta; order: Diptera; family: Tachinidae; genus: Hyphantrophaga; specificEpithet: virilis; scientificNameAuthorship: (Aldrich & Webber, 1924); **Location:** country: Costa Rica; countryCode: CR; stateProvince: Guanacaste; county: Sector Santa Rosa; locality: Area de Conservacion Guanacaste; verbatimLocality: Bosque San Emilio; verbatimElevation: 300; verbatimLatitude: 10.8439; verbatimLongitude: -85.6138; verbatimCoordinateSystem: Decimal; decimalLatitude: 10.8439; decimalLongitude: -85.6138; **Event:** samplingProtocol: Reared from the larva of the Notodontidae, Elasmia mandela; verbatimEventDate: 23-Aug-2008; **Record Level:** language: en; institutionCode: CNC; collectionCode: Insects; basisOfRecord: Pinned Specimen
- **Type status:** Other material. **Occurrence:** occurrenceDetails: http://janzen.sas.upenn.edu; catalogNumber: DHJPAR0029763; recordedBy: D.H. Janzen, W. Hallwachs & Guillermo Pereira; individualID: DHJPAR0029763; individualCount: 1; sex: female; lifeStage: adult; preparations: pinned; otherCatalogNumbers: ASHYM1184-09, 08-SRNP-14574, BOLD:AAA1577; **Taxon:** scientificName: Hyphantrophaga virilis; phylum: Arthropoda; class: Insecta; order: Diptera; family: Tachinidae; genus: Hyphantrophaga; specificEpithet: virilis; scientificNameAuthorship: (Aldrich & Webber, 1924); **Location:** country: Costa Rica; countryCode: CR; stateProvince: Guanacaste; county: Sector Santa Rosa; locality: Area de Conservacion Guanacaste; verbatimLocality: Bosque San Emilio; verbatimElevation: 300; verbatimLatitude: 10.8439; verbatimLongitude: -85.6138; verbatimCoordinateSystem: Decimal; decimalLatitude: 10.8439; decimalLongitude: -85.6138; **Event:** samplingProtocol: Reared from the larva of the Notodontidae, Elasmia mandela; verbatimEventDate: 24-Aug-2008; **Record Level:** language: en; institutionCode: CNC; collectionCode: Insects; basisOfRecord: Pinned Specimen
- **Type status:** Other material. **Occurrence:** occurrenceDetails: http://janzen.sas.upenn.edu; catalogNumber: DHJPAR0029764; recordedBy: D.H. Janzen, W. Hallwachs & Guillermo Pereira; individualID: DHJPAR0029764; individualCount: 1; sex: female; lifeStage: adult; preparations: pinned; otherCatalogNumbers: ASHYM1185-09, 08-SRNP-14572, BOLD:AAA1577; **Taxon:** scientificName: Hyphantrophaga virilis; phylum: Arthropoda; class: Insecta; order: Diptera; family: Tachinidae; genus: Hyphantrophaga; specificEpithet: virilis; scientificNameAuthorship: (Aldrich & Webber, 1924); **Location:** country: Costa Rica; countryCode: CR; stateProvince: Guanacaste; county: Sector Santa Rosa; locality: Area de Conservacion Guanacaste; verbatimLocality: Bosque San Emilio; verbatimElevation: 300; verbatimLatitude: 10.8439; verbatimLongitude: -85.6138; verbatimCoordinateSystem: Decimal; decimalLatitude: 10.8439; decimalLongitude: -85.6138; **Event:** samplingProtocol: Reared from the larva of the Notodontidae, Elasmia mandela; verbatimEventDate: 24-Aug-2008; **Record Level:** language: en; institutionCode: CNC; collectionCode: Insects; basisOfRecord: Pinned Specimen
- **Type status:** Other material. **Occurrence:** occurrenceDetails: http://janzen.sas.upenn.edu; catalogNumber: DHJPAR0029765; recordedBy: D.H. Janzen, W. Hallwachs & Guillermo Pereira; individualID: DHJPAR0029765; individualCount: 1; sex: female; lifeStage: adult; preparations: pinned; otherCatalogNumbers: ASHYM1186-09, 08-SRNP-14584, BOLD:AAA1577; **Taxon:** scientificName: Hyphantrophaga virilis; phylum: Arthropoda; class: Insecta; order: Diptera; family: Tachinidae; genus: Hyphantrophaga; specificEpithet: virilis; scientificNameAuthorship: (Aldrich & Webber, 1924); **Location:** country: Costa Rica; countryCode: CR; stateProvince: Guanacaste; county: Sector Santa Rosa; locality: Area de Conservacion Guanacaste; verbatimLocality: Bosque San Emilio; verbatimElevation: 300; verbatimLatitude: 10.8439; verbatimLongitude: -85.6138; verbatimCoordinateSystem: Decimal; decimalLatitude: 10.8439; decimalLongitude: -85.6138; **Event:** samplingProtocol: Reared from the larva of the Notodontidae, Elasmia mandela; verbatimEventDate: 24-Aug-2008; **Record Level:** language: en; institutionCode: CNC; collectionCode: Insects; basisOfRecord: Pinned Specimen
- **Type status:** Other material. **Occurrence:** occurrenceDetails: http://janzen.sas.upenn.edu; catalogNumber: DHJPAR0029769; recordedBy: D.H. Janzen, W. Hallwachs & Guillermo Pereira; individualID: DHJPAR0029769; individualCount: 1; sex: female; lifeStage: adult; preparations: pinned; otherCatalogNumbers: ASHYM1190-09, 08-SRNP-14439, BOLD:AAA1577; **Taxon:** scientificName: Hyphantrophaga virilis; phylum: Arthropoda; class: Insecta; order: Diptera; family: Tachinidae; genus: Hyphantrophaga; specificEpithet: virilis; scientificNameAuthorship: (Aldrich & Webber, 1924); **Location:** country: Costa Rica; countryCode: CR; stateProvince: Guanacaste; county: Sector Santa Rosa; locality: Area de Conservacion Guanacaste; verbatimLocality: Bosque San Emilio; verbatimElevation: 300; verbatimLatitude: 10.8439; verbatimLongitude: -85.6138; verbatimCoordinateSystem: Decimal; decimalLatitude: 10.8439; decimalLongitude: -85.6138; **Event:** samplingProtocol: Reared from the larva of the Notodontidae, Boriza tonac; verbatimEventDate: 24-Aug-2008; **Record Level:** language: en; institutionCode: CNC; collectionCode: Insects; basisOfRecord: Pinned Specimen
- **Type status:** Other material. **Occurrence:** occurrenceDetails: http://janzen.sas.upenn.edu; catalogNumber: DHJPAR0029770; recordedBy: D.H. Janzen, W. Hallwachs & Guillermo Pereira; individualID: DHJPAR0029770; individualCount: 1; sex: female; lifeStage: adult; preparations: pinned; otherCatalogNumbers: ASHYM1191-09, 08-SRNP-14594, BOLD:AAA1577; **Taxon:** scientificName: Hyphantrophaga virilis; phylum: Arthropoda; class: Insecta; order: Diptera; family: Tachinidae; genus: Hyphantrophaga; specificEpithet: virilis; scientificNameAuthorship: (Aldrich & Webber, 1924); **Location:** country: Costa Rica; countryCode: CR; stateProvince: Guanacaste; county: Sector Santa Rosa; locality: Area de Conservacion Guanacaste; verbatimLocality: Sendero Natural; verbatimElevation: 290; verbatimLatitude: 10.8357; verbatimLongitude: -85.6125; verbatimCoordinateSystem: Decimal; decimalLatitude: 10.8357; decimalLongitude: -85.6125; **Event:** samplingProtocol: Reared from the larva of the Notodontidae, Boriza tonac; verbatimEventDate: 24-Aug-2008; **Record Level:** language: en; institutionCode: CNC; collectionCode: Insects; basisOfRecord: Pinned Specimen
- **Type status:** Other material. **Occurrence:** occurrenceDetails: http://janzen.sas.upenn.edu; catalogNumber: DHJPAR0029773; recordedBy: D.H. Janzen, W. Hallwachs & Guillermo Pereira; individualID: DHJPAR0029773; individualCount: 1; sex: female; lifeStage: adult; preparations: pinned; otherCatalogNumbers: ASHYM1194-09, 08-SRNP-14576, BOLD:AAA1577; **Taxon:** scientificName: Hyphantrophaga virilis; phylum: Arthropoda; class: Insecta; order: Diptera; family: Tachinidae; genus: Hyphantrophaga; specificEpithet: virilis; scientificNameAuthorship: (Aldrich & Webber, 1924); **Location:** country: Costa Rica; countryCode: CR; stateProvince: Guanacaste; county: Sector Santa Rosa; locality: Area de Conservacion Guanacaste; verbatimLocality: Bosque San Emilio; verbatimElevation: 300; verbatimLatitude: 10.8439; verbatimLongitude: -85.6138; verbatimCoordinateSystem: Decimal; decimalLatitude: 10.8439; decimalLongitude: -85.6138; **Event:** samplingProtocol: Reared from the larva of the Notodontidae, Elasmia mandela; verbatimEventDate: 24-Aug-2008; **Record Level:** language: en; institutionCode: CNC; collectionCode: Insects; basisOfRecord: Pinned Specimen
- **Type status:** Other material. **Occurrence:** occurrenceDetails: http://janzen.sas.upenn.edu; catalogNumber: DHJPAR0029774; recordedBy: D.H. Janzen, W. Hallwachs & Guillermo Pereira; individualID: DHJPAR0029774; individualCount: 1; sex: female; lifeStage: adult; preparations: pinned; otherCatalogNumbers: ASHYM1195-09, 08-SRNP-14600, BOLD:AAA1577; **Taxon:** scientificName: Hyphantrophaga virilis; phylum: Arthropoda; class: Insecta; order: Diptera; family: Tachinidae; genus: Hyphantrophaga; specificEpithet: virilis; scientificNameAuthorship: (Aldrich & Webber, 1924); **Location:** country: Costa Rica; countryCode: CR; stateProvince: Guanacaste; county: Sector Santa Rosa; locality: Area de Conservacion Guanacaste; verbatimLocality: Sendero Natural; verbatimElevation: 290; verbatimLatitude: 10.8357; verbatimLongitude: -85.6125; verbatimCoordinateSystem: Decimal; decimalLatitude: 10.8357; decimalLongitude: -85.6125; **Event:** samplingProtocol: Reared from the larva of the Notodontidae, Boriza tonac; verbatimEventDate: 25-Aug-2008; **Record Level:** language: en; institutionCode: CNC; collectionCode: Insects; basisOfRecord: Pinned Specimen
- **Type status:** Other material. **Occurrence:** occurrenceDetails: http://janzen.sas.upenn.edu; catalogNumber: DHJPAR0029778; recordedBy: D.H. Janzen, W. Hallwachs & Johan Vargas; individualID: DHJPAR0029778; individualCount: 1; sex: female; lifeStage: adult; preparations: pinned; otherCatalogNumbers: ASHYM1199-09, 08-SRNP-14553, BOLD:AAA1577; **Taxon:** scientificName: Hyphantrophaga virilis; phylum: Arthropoda; class: Insecta; order: Diptera; family: Tachinidae; genus: Hyphantrophaga; specificEpithet: virilis; scientificNameAuthorship: (Aldrich & Webber, 1924); **Location:** country: Costa Rica; countryCode: CR; stateProvince: Guanacaste; county: Sector Santa Rosa; locality: Area de Conservacion Guanacaste; verbatimLocality: Bosque San Emilio; verbatimElevation: 300; verbatimLatitude: 10.8439; verbatimLongitude: -85.6138; verbatimCoordinateSystem: Decimal; decimalLatitude: 10.8439; decimalLongitude: -85.6138; **Event:** samplingProtocol: Reared from the larva of the Notodontidae, Boriza tonac; verbatimEventDate: 26-Aug-2008; **Record Level:** language: en; institutionCode: CNC; collectionCode: Insects; basisOfRecord: Pinned Specimen
- **Type status:** Other material. **Occurrence:** occurrenceDetails: http://janzen.sas.upenn.edu; catalogNumber: DHJPAR0029779; recordedBy: D.H. Janzen, W. Hallwachs & Guillermo Pereira; individualID: DHJPAR0029779; individualCount: 1; sex: female; lifeStage: adult; preparations: pinned; otherCatalogNumbers: ASHYM1200-09, 08-SRNP-14617, BOLD:AAA1577; **Taxon:** scientificName: Hyphantrophaga virilis; phylum: Arthropoda; class: Insecta; order: Diptera; family: Tachinidae; genus: Hyphantrophaga; specificEpithet: virilis; scientificNameAuthorship: (Aldrich & Webber, 1924); **Location:** country: Costa Rica; countryCode: CR; stateProvince: Guanacaste; county: Sector Santa Rosa; locality: Area de Conservacion Guanacaste; verbatimLocality: Sendero Natural; verbatimElevation: 290; verbatimLatitude: 10.8357; verbatimLongitude: -85.6125; verbatimCoordinateSystem: Decimal; decimalLatitude: 10.8357; decimalLongitude: -85.6125; **Event:** samplingProtocol: Reared from the larva of the Notodontidae, Boriza tonac; verbatimEventDate: 26-Aug-2008; **Record Level:** language: en; institutionCode: CNC; collectionCode: Insects; basisOfRecord: Pinned Specimen
- **Type status:** Other material. **Occurrence:** occurrenceDetails: http://janzen.sas.upenn.edu; catalogNumber: DHJPAR0029784; recordedBy: D.H. Janzen, W. Hallwachs & Guillermo Pereira; individualID: DHJPAR0029784; individualCount: 1; sex: female; lifeStage: adult; preparations: pinned; otherCatalogNumbers: ASHYM1205-09, 08-SRNP-14461, BOLD:AAA1577; **Taxon:** scientificName: Hyphantrophaga virilis; phylum: Arthropoda; class: Insecta; order: Diptera; family: Tachinidae; genus: Hyphantrophaga; specificEpithet: virilis; scientificNameAuthorship: (Aldrich & Webber, 1924); **Location:** country: Costa Rica; countryCode: CR; stateProvince: Guanacaste; county: Sector Santa Rosa; locality: Area de Conservacion Guanacaste; verbatimLocality: Bosque San Emilio; verbatimElevation: 300; verbatimLatitude: 10.8439; verbatimLongitude: -85.6138; verbatimCoordinateSystem: Decimal; decimalLatitude: 10.8439; decimalLongitude: -85.6138; **Event:** samplingProtocol: Reared from the larva of the Notodontidae, Boriza tonac; verbatimEventDate: 27-Aug-2008; **Record Level:** language: en; institutionCode: CNC; collectionCode: Insects; basisOfRecord: Pinned Specimen
- **Type status:** Other material. **Occurrence:** occurrenceDetails: http://janzen.sas.upenn.edu; catalogNumber: DHJPAR0029786; recordedBy: D.H. Janzen, W. Hallwachs & Guillermo Pereira; individualID: DHJPAR0029786; individualCount: 1; sex: female; lifeStage: adult; preparations: pinned; otherCatalogNumbers: ASHYM1207-09, 08-SRNP-14614, BOLD:AAA1577; **Taxon:** scientificName: Hyphantrophaga virilis; phylum: Arthropoda; class: Insecta; order: Diptera; family: Tachinidae; genus: Hyphantrophaga; specificEpithet: virilis; scientificNameAuthorship: (Aldrich & Webber, 1924); **Location:** country: Costa Rica; countryCode: CR; stateProvince: Guanacaste; county: Sector Santa Rosa; locality: Area de Conservacion Guanacaste; verbatimLocality: Sendero Natural; verbatimElevation: 290; verbatimLatitude: 10.8357; verbatimLongitude: -85.6125; verbatimCoordinateSystem: Decimal; decimalLatitude: 10.8357; decimalLongitude: -85.6125; **Event:** samplingProtocol: Reared from the larva of the Notodontidae, Boriza tonac; verbatimEventDate: 29-Aug-2008; **Record Level:** language: en; institutionCode: CNC; collectionCode: Insects; basisOfRecord: Pinned Specimen
- **Type status:** Other material. **Occurrence:** occurrenceDetails: http://janzen.sas.upenn.edu; catalogNumber: DHJPAR0029787; recordedBy: D.H. Janzen, W. Hallwachs & Guillermo Pereira; individualID: DHJPAR0029787; individualCount: 1; sex: female; lifeStage: adult; preparations: pinned; otherCatalogNumbers: ASHYM1208-09, 08-SRNP-14441, BOLD:AAA1577; **Taxon:** scientificName: Hyphantrophaga virilis; phylum: Arthropoda; class: Insecta; order: Diptera; family: Tachinidae; genus: Hyphantrophaga; specificEpithet: virilis; scientificNameAuthorship: (Aldrich & Webber, 1924); **Location:** country: Costa Rica; countryCode: CR; stateProvince: Guanacaste; county: Sector Santa Rosa; locality: Area de Conservacion Guanacaste; verbatimLocality: Bosque San Emilio; verbatimElevation: 300; verbatimLatitude: 10.8439; verbatimLongitude: -85.6138; verbatimCoordinateSystem: Decimal; decimalLatitude: 10.8439; decimalLongitude: -85.6138; **Event:** samplingProtocol: Reared from the larva of the Notodontidae, Boriza tonac; verbatimEventDate: 29-Aug-2008; **Record Level:** language: en; institutionCode: CNC; collectionCode: Insects; basisOfRecord: Pinned Specimen
- **Type status:** Other material. **Occurrence:** occurrenceDetails: http://janzen.sas.upenn.edu; catalogNumber: DHJPAR0029790; recordedBy: D.H. Janzen, W. Hallwachs & Guillermo Pereira; individualID: DHJPAR0029790; individualCount: 1; sex: female; lifeStage: adult; preparations: pinned; otherCatalogNumbers: ASHYM1211-09, 08-SRNP-14456, BOLD:AAA1577; **Taxon:** scientificName: Hyphantrophaga virilis; phylum: Arthropoda; class: Insecta; order: Diptera; family: Tachinidae; genus: Hyphantrophaga; specificEpithet: virilis; scientificNameAuthorship: (Aldrich & Webber, 1924); **Location:** country: Costa Rica; countryCode: CR; stateProvince: Guanacaste; county: Sector Santa Rosa; locality: Area de Conservacion Guanacaste; verbatimLocality: Bosque San Emilio; verbatimElevation: 300; verbatimLatitude: 10.8439; verbatimLongitude: -85.6138; verbatimCoordinateSystem: Decimal; decimalLatitude: 10.8439; decimalLongitude: -85.6138; **Event:** samplingProtocol: Reared from the larva of the Notodontidae, Boriza tonac; verbatimEventDate: 31-Aug-2008; **Record Level:** language: en; institutionCode: CNC; collectionCode: Insects; basisOfRecord: Pinned Specimen
- **Type status:** Other material. **Occurrence:** occurrenceDetails: http://janzen.sas.upenn.edu; catalogNumber: DHJPAR0029794; recordedBy: D.H. Janzen, W. Hallwachs & Lucia Vargas; individualID: DHJPAR0029794; individualCount: 1; sex: female; lifeStage: adult; preparations: pinned; otherCatalogNumbers: ASHYM1215-09, 08-SRNP-14753, BOLD:AAA1577; **Taxon:** scientificName: Hyphantrophaga virilis; phylum: Arthropoda; class: Insecta; order: Diptera; family: Tachinidae; genus: Hyphantrophaga; specificEpithet: virilis; scientificNameAuthorship: (Aldrich & Webber, 1924); **Location:** country: Costa Rica; countryCode: CR; stateProvince: Guanacaste; county: Sector Santa Rosa; locality: Area de Conservacion Guanacaste; verbatimLocality: Bosque San Emilio; verbatimElevation: 300; verbatimLatitude: 10.8439; verbatimLongitude: -85.6138; verbatimCoordinateSystem: Decimal; decimalLatitude: 10.8439; decimalLongitude: -85.6138; **Event:** samplingProtocol: Reared from the larva of the Megalopygidae, Megalopygidae 82-SRNP-848; **Record Level:** language: en; institutionCode: CNC; collectionCode: Insects; basisOfRecord: Pinned Specimen
- **Type status:** Other material. **Occurrence:** occurrenceDetails: http://janzen.sas.upenn.edu; catalogNumber: DHJPAR0030064; recordedBy: D.H. Janzen, W. Hallwachs & Johan Vargas; individualID: DHJPAR0030064; individualCount: 1; sex: female; lifeStage: adult; preparations: pinned; otherCatalogNumbers: ASHYB808-09, 08-SRNP-16977, BOLD:AAA1577; **Taxon:** scientificName: Hyphantrophaga virilis; phylum: Arthropoda; class: Insecta; order: Diptera; family: Tachinidae; genus: Hyphantrophaga; specificEpithet: virilis; scientificNameAuthorship: (Aldrich & Webber, 1924); **Location:** country: Costa Rica; countryCode: CR; stateProvince: Guanacaste; county: Sector Santa Rosa; locality: Area de Conservacion Guanacaste; verbatimLocality: Bosque Humedo; verbatimElevation: 290; verbatimLatitude: 10.8514; verbatimLongitude: -85.608; verbatimCoordinateSystem: Decimal; decimalLatitude: 10.8514; decimalLongitude: -85.608; **Event:** samplingProtocol: Reared from the larva of the Nymphalidae, Mechanitis isthmia; verbatimEventDate: 16-Jan-2009; **Record Level:** language: en; institutionCode: CNC; collectionCode: Insects; basisOfRecord: Pinned Specimen
- **Type status:** Other material. **Occurrence:** occurrenceDetails: http://janzen.sas.upenn.edu; catalogNumber: DHJPAR0030073; recordedBy: D.H. Janzen, W. Hallwachs & Johan Vargas; individualID: DHJPAR0030073; individualCount: 1; sex: female; lifeStage: adult; preparations: pinned; otherCatalogNumbers: ASHYB817-09, 08-SRNP-16963, BOLD:AAA1577; **Taxon:** scientificName: Hyphantrophaga virilis; phylum: Arthropoda; class: Insecta; order: Diptera; family: Tachinidae; genus: Hyphantrophaga; specificEpithet: virilis; scientificNameAuthorship: (Aldrich & Webber, 1924); **Location:** country: Costa Rica; countryCode: CR; stateProvince: Guanacaste; county: Sector Santa Rosa; locality: Area de Conservacion Guanacaste; verbatimLocality: Bosque Humedo; verbatimElevation: 290; verbatimLatitude: 10.8514; verbatimLongitude: -85.608; verbatimCoordinateSystem: Decimal; decimalLatitude: 10.8514; decimalLongitude: -85.608; **Event:** samplingProtocol: Reared from the larva of the Nolidae, Iscadia Poole05; verbatimEventDate: 14-Jan-2009; **Record Level:** language: en; institutionCode: CNC; collectionCode: Insects; basisOfRecord: Pinned Specimen
- **Type status:** Other material. **Occurrence:** occurrenceDetails: http://janzen.sas.upenn.edu; catalogNumber: DHJPAR0030464; recordedBy: D.H. Janzen, W. Hallwachs & Elda Araya; individualID: DHJPAR0030464; individualCount: 1; sex: female; lifeStage: adult; preparations: pinned; otherCatalogNumbers: ASHYB1207-09, 09-SRNP-112, BOLD:AAA1577; **Taxon:** scientificName: Hyphantrophaga virilis; phylum: Arthropoda; class: Insecta; order: Diptera; family: Tachinidae; genus: Hyphantrophaga; specificEpithet: virilis; scientificNameAuthorship: (Aldrich & Webber, 1924); **Location:** country: Costa Rica; countryCode: CR; stateProvince: Alajuela; county: Sector San Cristobal; locality: Area de Conservacion Guanacaste; verbatimLocality: Quebrada Garcia; verbatimElevation: 495; verbatimLatitude: 10.8607; verbatimLongitude: -85.4256; verbatimCoordinateSystem: Decimal; decimalLatitude: 10.8607; decimalLongitude: -85.4256; **Event:** samplingProtocol: Reared from the larva of the Tortricidae, Anacrusis nephrodes; verbatimEventDate: 23-Feb-2009; **Record Level:** language: en; institutionCode: CNC; collectionCode: Insects; basisOfRecord: Pinned Specimen
- **Type status:** Other material. **Occurrence:** occurrenceDetails: http://janzen.sas.upenn.edu; catalogNumber: DHJPAR0034373; recordedBy: D.H. Janzen, W. Hallwachs & Manuel Pereira; individualID: DHJPAR0034373; individualCount: 1; sex: female; lifeStage: adult; preparations: pinned; otherCatalogNumbers: ASHYC1025-09, 09-SRNP-35335, BOLD:AAA1577; **Taxon:** scientificName: Hyphantrophaga virilis; phylum: Arthropoda; class: Insecta; order: Diptera; family: Tachinidae; genus: Hyphantrophaga; specificEpithet: virilis; scientificNameAuthorship: (Aldrich & Webber, 1924); **Location:** country: Costa Rica; countryCode: CR; stateProvince: Guanacaste; county: Sector Cacao; locality: Area de Conservacion Guanacaste; verbatimLocality: Sendero Arenales; verbatimElevation: 1080; verbatimLatitude: 10.9247; verbatimLongitude: -85.4674; verbatimCoordinateSystem: Decimal; decimalLatitude: 10.9247; decimalLongitude: -85.4674; **Event:** samplingProtocol: Reared from the larva of the Geometridae, geometrid 02-8481; verbatimEventDate: 22-May-2009; **Record Level:** language: en; institutionCode: CNC; collectionCode: Insects; basisOfRecord: Pinned Specimen
- **Type status:** Other material. **Occurrence:** occurrenceDetails: http://janzen.sas.upenn.edu; catalogNumber: DHJPAR0035671; recordedBy: D.H. Janzen, W. Hallwachs & Daniel M.Acuna; individualID: DHJPAR0035671; individualCount: 1; sex: female; lifeStage: adult; preparations: pinned; otherCatalogNumbers: ASHYD1052-09, 09-SRNP-56316, BOLD:AAA1577; **Taxon:** scientificName: Hyphantrophaga virilis; phylum: Arthropoda; class: Insecta; order: Diptera; family: Tachinidae; genus: Hyphantrophaga; specificEpithet: virilis; scientificNameAuthorship: (Aldrich & Webber, 1924); **Location:** country: Costa Rica; countryCode: CR; stateProvince: Guanacaste; county: Sector Pailas; locality: Area de Conservacion Guanacaste; verbatimLocality: Gemelos; verbatimElevation: 1276; verbatimLatitude: 10.7693; verbatimLongitude: -85.3466; verbatimCoordinateSystem: Decimal; decimalLatitude: 10.7693; decimalLongitude: -85.3466; **Event:** samplingProtocol: Reared from the larva of the Crambidae, same as 06-21595; verbatimEventDate: 05-Jul-2009; **Record Level:** language: en; institutionCode: CNC; collectionCode: Insects; basisOfRecord: Pinned Specimen
- **Type status:** Other material. **Occurrence:** occurrenceDetails: http://janzen.sas.upenn.edu; catalogNumber: DHJPAR0035675; recordedBy: D.H. Janzen, W. Hallwachs & Winnie Hallwachs; individualID: DHJPAR0035675; individualCount: 1; sex: female; lifeStage: adult; preparations: pinned; otherCatalogNumbers: ASHYD1056-09, 09-SRNP-14232, BOLD:AAA1577; **Taxon:** scientificName: Hyphantrophaga virilis; phylum: Arthropoda; class: Insecta; order: Diptera; family: Tachinidae; genus: Hyphantrophaga; specificEpithet: virilis; scientificNameAuthorship: (Aldrich & Webber, 1924); **Location:** country: Costa Rica; countryCode: CR; stateProvince: Guanacaste; county: Sector Santa Rosa; locality: Area de Conservacion Guanacaste; verbatimLocality: Area Administrativa; verbatimElevation: 295; verbatimLatitude: 10.8376; verbatimLongitude: -85.6187; verbatimCoordinateSystem: Decimal; decimalLatitude: 10.8376; decimalLongitude: -85.6187; **Event:** samplingProtocol: Reared from the larva of the Nymphalidae, Lycorea atergatis; verbatimEventDate: 02-Sep-2009; **Record Level:** language: en; institutionCode: CNC; collectionCode: Insects; basisOfRecord: Pinned Specimen
- **Type status:** Other material. **Occurrence:** occurrenceDetails: http://janzen.sas.upenn.edu; catalogNumber: DHJPAR0036643; recordedBy: D.H. Janzen, W. Hallwachs & Manuel Pereira; individualID: DHJPAR0036643; individualCount: 1; sex: female; lifeStage: adult; preparations: pinned; otherCatalogNumbers: ASHYE1554-09, 09-SRNP-35330, BOLD:AAA1577; **Taxon:** scientificName: Hyphantrophaga virilis; phylum: Arthropoda; class: Insecta; order: Diptera; family: Tachinidae; genus: Hyphantrophaga; specificEpithet: virilis; scientificNameAuthorship: (Aldrich & Webber, 1924); **Location:** country: Costa Rica; countryCode: CR; stateProvince: Guanacaste; county: Sector Cacao; locality: Area de Conservacion Guanacaste; verbatimLocality: Sendero Arenales; verbatimElevation: 1080; verbatimLatitude: 10.9247; verbatimLongitude: -85.4674; verbatimCoordinateSystem: Decimal; decimalLatitude: 10.9247; decimalLongitude: -85.4674; **Event:** samplingProtocol: Reared from the larva of the Geometridae, geometrid 02-8481; verbatimEventDate: 30-May-2009; **Record Level:** language: en; institutionCode: CNC; collectionCode: Insects; basisOfRecord: Pinned Specimen
- **Type status:** Other material. **Occurrence:** occurrenceDetails: http://janzen.sas.upenn.edu; catalogNumber: DHJPAR0036649; recordedBy: D.H. Janzen, W. Hallwachs & Dunia Garcia; individualID: DHJPAR0036649; individualCount: 1; sex: female; lifeStage: adult; preparations: pinned; otherCatalogNumbers: ASHYE1560-09, 09-SRNP-35968, BOLD:AAA1577; **Taxon:** scientificName: Hyphantrophaga virilis; phylum: Arthropoda; class: Insecta; order: Diptera; family: Tachinidae; genus: Hyphantrophaga; specificEpithet: virilis; scientificNameAuthorship: (Aldrich & Webber, 1924); **Location:** country: Costa Rica; countryCode: CR; stateProvince: Guanacaste; county: Sector Cacao; locality: Area de Conservacion Guanacaste; verbatimLocality: Sendero Nayo; verbatimElevation: 1090; verbatimLatitude: 10.9245; verbatimLongitude: -85.4695; verbatimCoordinateSystem: Decimal; decimalLatitude: 10.9245; decimalLongitude: -85.4695; **Event:** samplingProtocol: Reared from the larva of the Geometridae, Macaria pernicata; verbatimEventDate: 26-Jun-2009; **Record Level:** language: en; institutionCode: CNC; collectionCode: Insects; basisOfRecord: Pinned Specimen
- **Type status:** Other material. **Occurrence:** occurrenceDetails: http://janzen.sas.upenn.edu; catalogNumber: DHJPAR0036650; recordedBy: D.H. Janzen, W. Hallwachs & Dunia Garcia; individualID: DHJPAR0036650; individualCount: 1; sex: female; lifeStage: adult; preparations: pinned; otherCatalogNumbers: ASHYE1561-09, 09-SRNP-35962, BOLD:AAA1577; **Taxon:** scientificName: Hyphantrophaga virilis; phylum: Arthropoda; class: Insecta; order: Diptera; family: Tachinidae; genus: Hyphantrophaga; specificEpithet: virilis; scientificNameAuthorship: (Aldrich & Webber, 1924); **Location:** country: Costa Rica; countryCode: CR; stateProvince: Guanacaste; county: Sector Cacao; locality: Area de Conservacion Guanacaste; verbatimLocality: Sendero Nayo; verbatimElevation: 1090; verbatimLatitude: 10.9245; verbatimLongitude: -85.4695; verbatimCoordinateSystem: Decimal; decimalLatitude: 10.9245; decimalLongitude: -85.4695; **Event:** samplingProtocol: Reared from the larva of the Geometridae, Macaria pernicata; verbatimEventDate: 26-Jun-2009; **Record Level:** language: en; institutionCode: CNC; collectionCode: Insects; basisOfRecord: Pinned Specimen
- **Type status:** Other material. **Occurrence:** occurrenceDetails: http://janzen.sas.upenn.edu; catalogNumber: DHJPAR0036653; recordedBy: D.H. Janzen, W. Hallwachs & Dunia Garcia; individualID: DHJPAR0036653; individualCount: 1; sex: female; lifeStage: adult; preparations: pinned; otherCatalogNumbers: ASHYE1564-09, 09-SRNP-35956, BOLD:AAA1577; **Taxon:** scientificName: Hyphantrophaga virilis; phylum: Arthropoda; class: Insecta; order: Diptera; family: Tachinidae; genus: Hyphantrophaga; specificEpithet: virilis; scientificNameAuthorship: (Aldrich & Webber, 1924); **Location:** country: Costa Rica; countryCode: CR; stateProvince: Guanacaste; county: Sector Cacao; locality: Area de Conservacion Guanacaste; verbatimLocality: Sendero Nayo; verbatimElevation: 1090; verbatimLatitude: 10.9245; verbatimLongitude: -85.4695; verbatimCoordinateSystem: Decimal; decimalLatitude: 10.9245; decimalLongitude: -85.4695; **Event:** samplingProtocol: Reared from the larva of the Geometridae, Macaria pernicata; verbatimEventDate: 27-Jun-2009; **Record Level:** language: en; institutionCode: CNC; collectionCode: Insects; basisOfRecord: Pinned Specimen
- **Type status:** Other material. **Occurrence:** occurrenceDetails: http://janzen.sas.upenn.edu; catalogNumber: DHJPAR0036656; recordedBy: D.H. Janzen, W. Hallwachs & Manuel Pereira; individualID: DHJPAR0036656; individualCount: 1; sex: female; lifeStage: adult; preparations: pinned; otherCatalogNumbers: ASHYE1567-09, 09-SRNP-35922, BOLD:AAA1577; **Taxon:** scientificName: Hyphantrophaga virilis; phylum: Arthropoda; class: Insecta; order: Diptera; family: Tachinidae; genus: Hyphantrophaga; specificEpithet: virilis; scientificNameAuthorship: (Aldrich & Webber, 1924); **Location:** country: Costa Rica; countryCode: CR; stateProvince: Guanacaste; county: Sector Cacao; locality: Area de Conservacion Guanacaste; verbatimLocality: Sendero Nayo; verbatimElevation: 1090; verbatimLatitude: 10.9245; verbatimLongitude: -85.4695; verbatimCoordinateSystem: Decimal; decimalLatitude: 10.9245; decimalLongitude: -85.4695; **Event:** samplingProtocol: Reared from the larva of the Geometridae, Macaria pernicata; verbatimEventDate: 28-Jun-2009; **Record Level:** language: en; institutionCode: CNC; collectionCode: Insects; basisOfRecord: Pinned Specimen
- **Type status:** Other material. **Occurrence:** occurrenceDetails: http://janzen.sas.upenn.edu; catalogNumber: DHJPAR0036657; recordedBy: D.H. Janzen, W. Hallwachs & Dunia Garcia; individualID: DHJPAR0036657; individualCount: 1; sex: female; lifeStage: adult; preparations: pinned; otherCatalogNumbers: ASHYE1568-09, 09-SRNP-35951, BOLD:AAA1577; **Taxon:** scientificName: Hyphantrophaga virilis; phylum: Arthropoda; class: Insecta; order: Diptera; family: Tachinidae; genus: Hyphantrophaga; specificEpithet: virilis; scientificNameAuthorship: (Aldrich & Webber, 1924); **Location:** country: Costa Rica; countryCode: CR; stateProvince: Guanacaste; county: Sector Cacao; locality: Area de Conservacion Guanacaste; verbatimLocality: Sendero Nayo; verbatimElevation: 1090; verbatimLatitude: 10.9245; verbatimLongitude: -85.4695; verbatimCoordinateSystem: Decimal; decimalLatitude: 10.9245; decimalLongitude: -85.4695; **Event:** samplingProtocol: Reared from the larva of the Geometridae, Macaria pernicata; verbatimEventDate: 26-Jun-2009; **Record Level:** language: en; institutionCode: CNC; collectionCode: Insects; basisOfRecord: Pinned Specimen
- **Type status:** Other material. **Occurrence:** occurrenceDetails: http://janzen.sas.upenn.edu; catalogNumber: DHJPAR0036658; recordedBy: D.H. Janzen, W. Hallwachs & Manuel Pereira; individualID: DHJPAR0036658; individualCount: 1; sex: female; lifeStage: adult; preparations: pinned; otherCatalogNumbers: ASHYE1569-09, 09-SRNP-35939, BOLD:AAA1577; **Taxon:** scientificName: Hyphantrophaga virilis; phylum: Arthropoda; class: Insecta; order: Diptera; family: Tachinidae; genus: Hyphantrophaga; specificEpithet: virilis; scientificNameAuthorship: (Aldrich & Webber, 1924); **Location:** country: Costa Rica; countryCode: CR; stateProvince: Guanacaste; county: Sector Cacao; locality: Area de Conservacion Guanacaste; verbatimLocality: Sendero Nayo; verbatimElevation: 1090; verbatimLatitude: 10.9245; verbatimLongitude: -85.4695; verbatimCoordinateSystem: Decimal; decimalLatitude: 10.9245; decimalLongitude: -85.4695; **Event:** samplingProtocol: Reared from the larva of the Geometridae, Macaria pernicata; verbatimEventDate: 30-Jun-2009; **Record Level:** language: en; institutionCode: CNC; collectionCode: Insects; basisOfRecord: Pinned Specimen
- **Type status:** Other material. **Occurrence:** occurrenceDetails: http://janzen.sas.upenn.edu; catalogNumber: DHJPAR0036659; recordedBy: D.H. Janzen, W. Hallwachs & Dunia Garcia; individualID: DHJPAR0036659; individualCount: 1; sex: female; lifeStage: adult; preparations: pinned; otherCatalogNumbers: ASHYE1570-09, 09-SRNP-35952, BOLD:AAA1577; **Taxon:** scientificName: Hyphantrophaga virilis; phylum: Arthropoda; class: Insecta; order: Diptera; family: Tachinidae; genus: Hyphantrophaga; specificEpithet: virilis; scientificNameAuthorship: (Aldrich & Webber, 1924); **Location:** country: Costa Rica; countryCode: CR; stateProvince: Guanacaste; county: Sector Cacao; locality: Area de Conservacion Guanacaste; verbatimLocality: Sendero Nayo; verbatimElevation: 1090; verbatimLatitude: 10.9245; verbatimLongitude: -85.4695; verbatimCoordinateSystem: Decimal; decimalLatitude: 10.9245; decimalLongitude: -85.4695; **Event:** samplingProtocol: Reared from the larva of the Geometridae, Macaria pernicata; verbatimEventDate: 26-Jun-2009; **Record Level:** language: en; institutionCode: CNC; collectionCode: Insects; basisOfRecord: Pinned Specimen
- **Type status:** Other material. **Occurrence:** occurrenceDetails: http://janzen.sas.upenn.edu; catalogNumber: DHJPAR0039307; recordedBy: D.H. Janzen, W. Hallwachs & Mariano Pereira; individualID: DHJPAR0039307; individualCount: 1; sex: female; lifeStage: adult; preparations: pinned; otherCatalogNumbers: ASTAV870-10, 10-SRNP-55350, BOLD:AAA1577; **Taxon:** scientificName: Hyphantrophaga virilis; phylum: Arthropoda; class: Insecta; order: Diptera; family: Tachinidae; genus: Hyphantrophaga; specificEpithet: virilis; scientificNameAuthorship: (Aldrich & Webber, 1924); **Location:** country: Costa Rica; countryCode: CR; stateProvince: Guanacaste; county: Sector Mundo Nuevo; locality: Area de Conservacion Guanacaste; verbatimLocality: Estacion La Perla; verbatimElevation: 325; verbatimLatitude: 10.7674; verbatimLongitude: -85.4331; verbatimCoordinateSystem: Decimal; decimalLatitude: 10.7674; decimalLongitude: -85.4331; **Event:** samplingProtocol: Reared from the larva of the Crambidae, Syllepte belialis; verbatimEventDate: 17-Jun-2010; **Record Level:** language: en; institutionCode: CNC; collectionCode: Insects; basisOfRecord: Pinned Specimen

#### Description

**Male** (Fig. 26). Length: 6–10 mm. **Head** (Fig. 26b): vertex 1/5 of head width; two reclinate upper orbital setae; ocellar setae arising beside anterior ocellus; ocellar triangle gold, concolorous with fronto-orbital plate; fronto-orbital plate gold over 80% of surface, sparsely setulose, setulae not extending beyond lowest frontal seta; parafacial shiny silver and bare; facial ridge bare; eye with short sparse ommatrichia up to 2X as long as one ommatidium; pedicel black, concolorous with postpedicel; arista brown, very minutely pubescent, distinctly thickened on basal 1/3–1/4; palpus yellow and haired. **Thorax** (Fig. 26a, c): brilliant gold tomentose dorsally, grey tomentose laterally; four prominent dorsal vittae, outermost two broken across suture, innermost pair unbroken across suture, not reaching 2nd postsutural dorsocentral seta; postpronotum with three setae arranged in a triangle; chaetotaxy: acrostichal setae 3:3; dorsocentral setae 3:3; intra-alar setae 2:3; supra-alar setae 2:3; two katepisternal setae; lateral scutellar setae 2/3 as long as basal scutellar setae, lateral scutellar setae 1/2 as thick as both basal and subapical scutellar setae; subapical scutellar setae subequal to basal scutellar setae; apical scutellar setae 1/2 length of subapical scutellar setae, convergent but not crossed apically; 1–2 pairs of discal scutellar setae; scutellum with two dark grey bilateral crescents along basal 10%, remainder concolorous with scutum. **Legs** (Fig. 26c): yellow in ground colour with dense covering of black hairs, making them appear darker; fore femur with dense silver tomentum on posterodorsal surface; hind coxa setose. **Wing** (Fig. 26a): pale translucent, veins yellow; vein R_4+5_ with 1–2 setulae at base. **Abdomen** (Fig. 26a, c): ground colour black; mid-dorsal depression on ST1+2 reaching hind margin; median marginal setae present on ST1+2–T3; a complete row of marginal setae present on T4; discal setae present on T3–T5; sex patch present on T4; ST1+2 black; distinct gold tomentose bands along anterior 9/10 of T3–T5; T3 silver tomentose ventrally; T5 gold tomentose ventrally. **Terminalia** (Fig. 26d, e, f): anterior margin of sternite 5 (Fig. 26f) with a shallow curved medial depression, posterior margin with a deeply excavated pointed U-shaped medial cleft; posterior lobes of sternite rounded triangular apically, with many short setulae; unsclerotised "window" absent. Cerci in posterior view (Fig. 26d) long and slender, together appearing as a narrow isosceles triangle and very slightly longer than surstyli, blunt at apex, completely separate medially and straight, not strongly divergent from each other; in lateral view strongly tapered along anterior 1/3 with a strong basal hump; densely setulose along dorsal 4/5, apparently bare ventrally. Surstylus in lateral view (Fig. 26e) almost parallel-sided along its length, with rounded apices, blade-like; when viewed dorsally, surstyli appearing slender and straight, not divergent, entirely covered in short, stout spines. Pregonite broad and well-developed, slightly bent; basal 2/3 slightly cinched, giving it a very slightly clubbed appearance, apically rounded and bare. Postgonite elongate, equallly wide along its length, 1/2 as wide as pregonite, with a slight curve at tip, subequal in length to pregonite. Distiphallus rectangular, with a very slight apical flare, a slender median longitudinal sclerotised reinforcement on its posterior surface and a broad, anterolateral, sclerotised acrophallus, joined as a plate on anterior surface near apex.

**Female**. Length: 6–10 mm. As male, differing only by the presence of two pairs of proclinate orbital setae.

#### Diagnosis

*Hyphantrophaga virlis* can be distinguished from all other *Hyphantrophaga* species by the following combination of traits: three postsutural dorsocentral setae, hind coxa setose, median marginal setae present on ST1+2 and discal setae present on T3–T5.

#### Distribution

From south-eastern Canada and north-eastern USA, west to Kansas and south to Costa Rica; Costa Rica, ACG (Provinces of Alajuela and Guanacaste), 2–1220 m elevation.

#### Ecology

Within the ACG inventory, *Hyphantrophaga virilis* has been reared 669 times from species spanning 30 families of Lepidoptera, as follows: one species in the family Apatelodidae; two species in the family Bombycidae; 18 species in the family Crambidae; four species in the family Depressariidae; 24 species in the family Erebidae; one species in the family Euteliidae; one species in the family Gelechiidae; 13 species in the family Geometridae; one species in the family Hedylidae; 64 species in the family Hesperiidae; one species in the family Immidae; two species in the family Lasiocampidae; one species in the family Limacodidae; two species in the family Megalopygidae; one species in the family Mimallonidae; 12 species in the family Noctuidae; four species in the family Nolidae; 24 species in the family Notodontidae; 18 species in the family Nymphalidae; three species in the family Papilionidae; one species in the family Phiditiidae; eight species in the family Pieridae; one species in the family Pterophoridae; six species in the family Pyralidae; six species in the family Riodinidae; four species in the family Saturniidae; five species in the family Sphingidae; three species in the family Thyrididae; and one species in the family Tortricidae; in rain forest, dry forest and dry-rain lowland intergrade.

## Identification Keys

### Key to the species of *Hyphantrophaga* Townsend, of the Mesoamerican region

**Table:** 

1	Hind coxa setose with at least 1 hair (Fig. 1b)	2
–	Hind coxa bare	18
2	Tomentum on T5 bright gold, contrasting with tomentum of T1+2–T4	*Hyphantrophaga myersi* (Aldrich, 1933)
–	Tomentum on T5 matching that on rest of abdominal tergites	3
3	Discal setae present on T3–T5	4
–	Discal setae present only on T5	13
4	Thorax with 3 postsutural dorsocentral setae	*Hyphantrophaga virilis* (Aldrich & Webber, 1924)
–	Thorax with 4 postsutural dorsocentral setae	5
5	Fronto-orbital plate silver tomentose; abdomen and thorax entirely silver/grey tomentose	6
–	Fronto-orbital plate brassy/gold tomentose; abdomen and thorax either with both silver and gold tomentum or brassy-grey tomentose	8
6	Pedicel bright orange; legs bright orange with black tarsi	*Hyphantrophaga autographae* (Sellers, 1943)
–	Pedicel black; legs dark brown/black	7
7	Transverse abdominal brassy tomentose bands along anterior edges of T3–T5 unbroken medially, covering almost 70% of tergites; T5 with silver tomentum throughout	*Hyphantrophaga duniagarciae* **sp. n.**
–	Distinct brassy-grey tomentum covering all but posterior margin of T3 and T4; T3 and T4 with darkened patches dorsolaterally, patches concolorous with ST1+2; T5 with brassy-grey tomentum throughout	*Hyphantrophaga osvaldoespinozai* **sp. n.**
8	Pedicel and arista orange-brown, lighter than postpedicel	9
–	Pedicel and arista black, concolorous with postpedicel	10
9	Fronto-orbital plate and parafacial entirely gold; fronto-orbital plate sparsely setulose along upper half	*Hyphantrophaga blandoides* Thompson, 1963
–	Fronto-orbital plate gold, parafacial silver; fronto-orbital plate sparsely setulose, setulae extending to lowest frontal seta	*Hyphantrophaga guillermopereirai* **sp. n.**
10	Median pair of thoracic vittae interrupted at suture	*Hyphantrophaga ciriloumanai* **sp. n.**
–	Median pair of thoracic vittae continuous across suture	11
11	Tomentose bands on T3 and T4 extending only on basal half (up to 50%) of tergites; T5 gold tomentose with a black apex	*Hyphantrophaga nigricauda* **sp. n.**
–	Tomentose bands on T3 and T4 extending over 70% of tergites; T5 entirely covered in gold tomentum	12
12	Dorsal tomentum of thorax of brilliant yellow-gold colour; chaetotaxy as follows: acrostichal setae 4:3, intra-alar setae 3:3, three katepisternal setae	*Hyphantrophaga eliethcantillanoae* **sp. n.**
–	Dorsal tomentum of thorax of pale-brassy colour; chaetotaxy as follows: acrostichal setae 3:3, intra-alar setae 2:3, two katepisternal setae	*Hyphantrophaga adrianguadamuzi* **sp. n.**
13	2 katepisternal setae	14
–	3 katepisternal setae	15
14	3 postsutural dorsocentral setae	*Hyphantrophaga calva***sp. n.** in part (females)
–	4 postsutural dorsocentral setae	*Hyphantrophaga luciariosae* **sp. n.**
15	Pedicel brownish orange; entire fly grey tomentose; sex patch of male indistinct/absent (Fig. 1d)	16
–	Pedicel brown to black; fly not entirely grey tomentose; sex patch of male present (Fig. 1c)	17
16	Ocellar triangle gold; fronto-orbital plate sparsely setulose throughout; abdominal tergites with silver tomentum throughout, brassy toned at tergal edges	*Hyphantrophaga calixtomoragai* **sp. n.**
–	Ocellar triangle silver, concolorous with fronto-orbital plate and parafacial; fronto-orbital plate sparsely setulose only along upper half; abdominal tergites with silver tomentum throughout, lacking brassy tones	*Hyphantrophaga manuelriosi* **sp. n.**
17	Found parasitising only medium-sized, naked, ringed nymphalid larvae from open heavily insolated grassland and pasture habitats	*Hyphantrophaga danausophaga***sp. n.** in part
–	Found parasitising only large, hairy, non-ringed cryptic nymphalid larvae in densely shaded habitat within the forest	*Hyphantrophaga morphophaga***sp. n.** in part
18	Lateral surface of thorax with pale blond setulae	*Hyphantrophaga albopilosa* **sp. n.**
–	Lateral surfaces of thorax with only black setulae throughout	19
19	Median marginal setae present on ST1+2	20
–	Median marginal setae absent on ST1+2	27
20	Ocellar triangle brassy-silver, appearing gold under some angles of light; 3 postsutural dorsocentral setae; abdomen ground colour entirely black, with silver or gold tomentum	21
–	Ocellar triangle brassy-gold tinged; 3–4 postsutural dorsocentral setae; abdomen ranging from dark reddish-brown to entirely reddish	22
21	Pedicel orange; facial ridge bare; fronto-orbital plate silver over 80%	*Hyphantrophaga fasciata* (Curran, 1934)
–	Pedicel black; facial ridge haired over 1/2 its length; fronto-orbital plate gold over 60%	*Hyphantrophaga hazelcambroneroae* **sp. n.**
22	Thorax with three post-sutural dorsocentral setae	23
–	Thorax with four post-sutural dorsocentral setae	24
23	Palpus yellow/orange throughout and thorax with 2 katepisternal setae	*Hyphantrophaga calva***sp. n.** in part (males)
–	Palpus dark brown/black basally with orange at tip and thorax with 3 katepisternal setae	*Hyphantrophaga angustata* (van der Wulp)
24	Ground colour of abdomen light orange-brown throughout; ground colour of thorax light orange-brown; ST1+2–T5 with silver tomentum ventrally; coxae and femora orangy-brown, tibiae yellow	*Hyphantrophaga adamsoni* Thompson, 1963
–	Ground colour of abdomen dark, reddish-brown with black tones; ground colour of thorax black/dark grey; ground colour of T4 and T5 ventrally dark brown, only T3 with silver tomentum on ventral surface; legs dark reddish-brown to black	25
25	Pedicel orange, of distinctly lighter colour than postpedicel; thorax with 2 katepisternal setae	*Hyphantrophaga pabloumanai* **sp. n.**
–	Pedicel black to dark brown; thorax with 3 katepisternal setae	26
26	Legs dark reddish-brown; tomentum covering more than 50% of abdominal T3, ventral surface of tergite with silver tomentum reaching edge of tergite	*Hyphantrophaga eldaarayae* **sp. n.**
–	Legs black; tomentum covering less than 50% of abdominal T3, tomentum on ventral surface of tergite covering up to 90% of tergite, but not reaching ventral edge	*Hyphantrophaga diniamartinezae* **sp. n.**
27	Thorax with 2 katepisternal setae	28
–	Thorax with 3 katepisternal setae	29
28	Dorsum of thorax brassy tomentose with four distinct unfused dorsal vittae, inner pair extending up to third postsutural dorsocentral seta	*Hyphantrophaga similis* **sp. n.**
–	Dorsum of thorax gold tomentose with four dorsal vittae fused into two prominent dark lines extending across thorax	*Hyphantrophaga gilberthampiei* **sp. n.**
29	Legs light coloured, yellow; abdominal tergites entirely brassy-silver tomentose in the form of bands spanning the tergite; in males, sex patch not extending dorsally on T4	*Hyphantrophaga edwinapui* **sp. n.**
–	Legs dark coloured, if yellow in ground colour then so densely covered in black setulae as to appear dark; abdominal tergites not entirely brassy-silver tomentose, but tomentum broken into bilateral silver patches; in males, sex patch extending dorsally on T4	30
30	Legs entirely black; T4 with silver tomentum covering approximately 20% of dorsal surface; ground colour of abdomen dark, reddish-brown to black; scutellum black over 20% of its surface, with gold tomentum over remainder	*Hyphantrophaga anacordobae* **sp. n.**
–	Legs reddish-brown, covered with dense dark hairs and with some light coloured spots at base of femora, adjacent to coxae; T4 with silver tomentum covering more than 50% of dorsal surface; ground colour of abdomen light orange-brown; scutellum gold tomentum covering entire surface margin	31
31	Found parasitising only medium-sized, naked, ringed nymphalid larvae from open, heavily insolated grassland and pasture habitats	*Hyphantrophaga danausophaga***sp. n.** in part
–	Found parasitising only large, hairy, non-ringed, cryptic nymphalid larvae from densely shaded covered habitats within the forest	*Hyphantrophaga morphophaga***sp. n.** in part

This key was written, based on characters present in males; in cases where sexual dimorphism is present, the species keys to males and females separately. This key is confined to the Mesoamerican biogeographical region, defined as ranging from the Isthmus of Tehuantepec in the north to the Colombian border with Panama in the south.

## Analysis

### *Hyphantrophaga morphophaga* species-group

Two of our new species, *Hyphantrophaga morphophaga* and *Hyphantrophaga danausophaga*, require a complex explanation, but one that the results of the overall ACG inventory are suggesting will become commonplace as more in-situ exploration of tropical species takes place through the interfacing of genetics with natural history traits. Only two traits are currently known to separate them: their habitat and their sympatric host caterpillars. These traits are not available for typical museum specimens. While the ACG caterpillar and parasitoid inventory began in 1978, the first *Morpho* Fabricius, 1807 caterpillar (Nymphalidae) was not found until 1981 and the first *H. morphophaga* was reared in 1984. Since then, it has been reared only from 11% of 1135 wild-caught *Morpho helenor* (Cramer, 1776) caterpillars feeding on 33 species of woody Fabaceae in dry forest and rain forest interior and from 21% of 62 caterpillars feeding on two species of Dichapetalaceae in rain forest interior and from 7% of 123 *Morpho polyphemus catalina* Correa & Chacon, 1984 wild-caught caterpillars feeding on five species of Fabaceae and one species of Capparaceae, all in dense shade of interior ACG cloud forest (Janzen and Hallwachs 2018). It has never been reared from the other two common rain forest species of *Morpho* found by the ACG inventory (408 *Morpho amathonte* (Deyrolle, 1860), feeding mostly on the same species of Fabaceae and 222 *Morpho theseus justitiae* (Deyrolle, 1860), feeding primarily on Menispermaceae in the shade of the same forests), though both are parasitised by two other species of *Hyphantrophaga* that are easily distinguished by morphology and DNA barcodes. The first *Danaus plexippus* (Linnaeus, 1758) caterpillar was found in 1998 and the first *H. danausophaga* was reared the same year. Since then, it has been reared only from 15% of 603 wild-caught *D. plexippus* caterpillars, 7% of 117 wild-caught *Danaus gilippus* (Cramer, 1775) caterpillars and 1% of 302 wild-caught *Lycorea halia atergatis* (Doubleday, 1847) caterpillars. *Lycorea h. atergatis* is not only a Nymphalidae, but it is also similar to *Danaus* Kluk, 1802 in caterpillar morphology (ringed, naked, ostentatious), pupal morphology and food plants (those with milky latex growing on insolated forest edges) and is phylogenetically closely related (Brower et al. 2010).

These two species of *Hyphantrophaga* cannot be distinguished by external morphology, terminalia, colour pattern or DNA barcodes. In any NJ tree, they are highly intermingled within a single BIN, with no apparent base pair difference. However, in 1995, the first specimens of *H. morphophaga* were presented to one of us (DMW) with no background information, as just another tachinid fly. They were given the interim name *Hyphantrophaga* Wood05. When, in 1999, the first specimens of *H. danausophaga* were likewise presented to DMW, again with no background information, they were compared with *H.* Wood05 and deemed to be different enough to be baptised, at the time, *H.* Wood06; however, today, we cannot find the morphological traits that led to this initial discrimination. As the years passed and the number of records accumulated, two hypotheses emerged:

A. The two belong to a single species that parasitises only medium-sized, naked, ringed and ostentatious *Danaus* and *Lycorea* Doubleday, 1847 caterpillars feeding on Asclepiadaceae and other latex-generating plants in open, intensely insolated, pastures and their edges in rain forest and dry forest and simultaneously parasitises only large, hairy, non-ringed cryptic caterpillars of *Morpho helenor* and *M. p. catalina* (never parasitising the co-occurring *M. amathonte* and *M. t. justitiae*) feeding on Fabaceae and Menispermaceae in the dense forest shade rather than in the open pastures, strongly populated with *Asclepias curassavica* (Asclepiadaceae), the primary ACG food plant of *D. plexippus* and *D. gilippus*.

B. These are two extremely similar species, one living in fully insolated habitats and parasitising just a few species of ostentatious, naked *Danaus* and *Lycorea* caterpillars (Fig. 27) that feed on Asclepiadaceae and other latex-baring plants and the other living in dense shade and parasitising just two species of large hairy cryptic *Morpho* caterpillars (Fig. 28) that feed almost entirely on Fabaceae in dense shade.

We take hypothesis B to represent the more likely natural history and therefore describe two new species, inconvenient as that is for curators and other users of specimens of these flies, who do not have rearing records for them. When funds are available for deep exploration of their nuclear genomes, they will be amongst the first species to be explored. If they are found to be different at the nuclear genome level, that will not change how inconvenient cases like these are for identification systems that are based solely on morphology or DNA barcodes.

### DNA Barcoding

**Figure 29.**
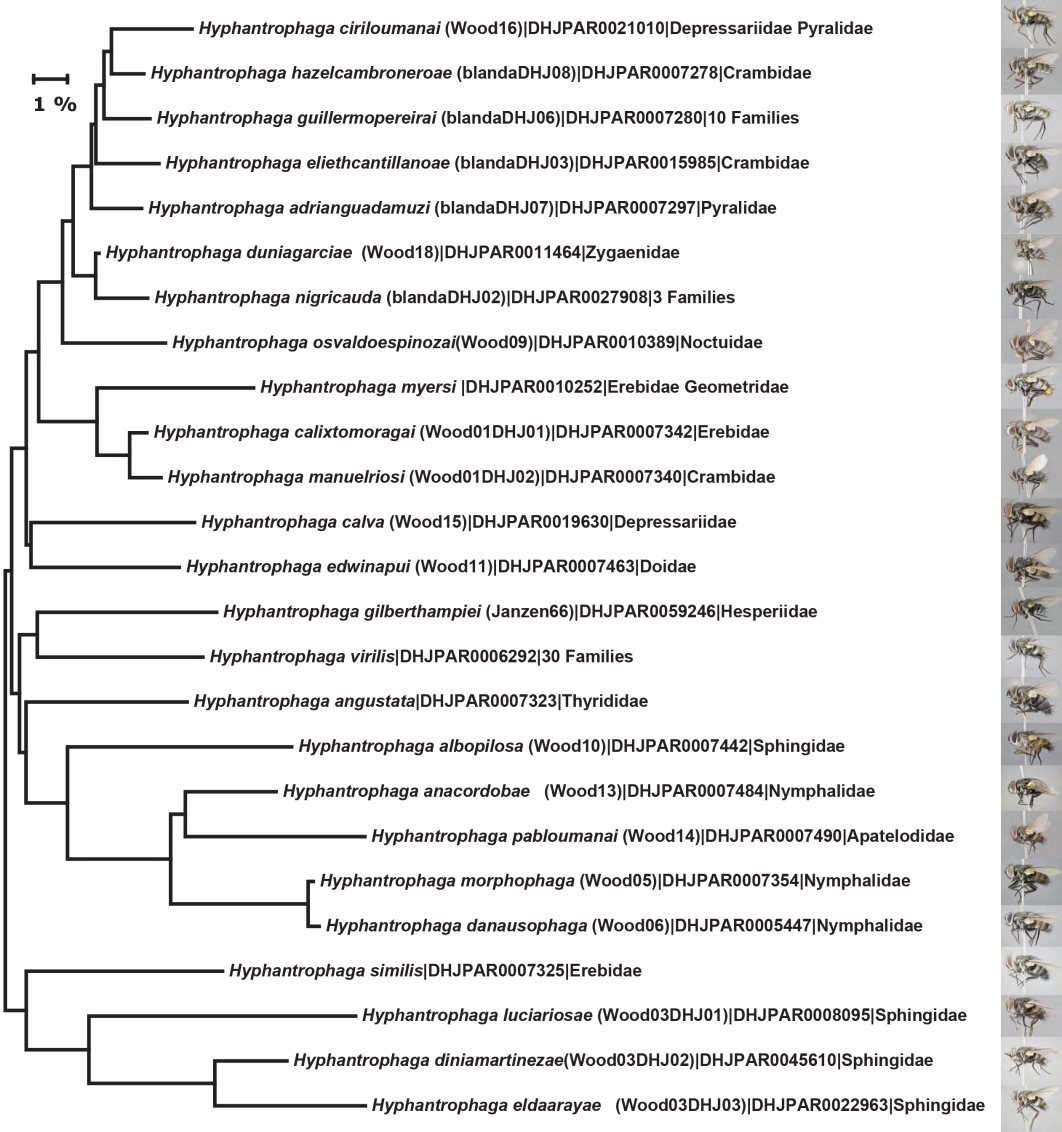
CO1 NJ tree for the *Hyphantrophaga* spp. of ACG (Costa Rica). Tip labels are the species names [(older interim name)|sample accession|host family(ies)].

A phylogenetic tree based on CO1 barcodes was used to visually confirm the variation present within and between each species and is presented in Fig. 29. For the *Hyphantrophaga* CO1 barcodes, we calculated the best DNA substitution model using the model test module in MEGA6 (Tamura et al. 2013) to find the model with the lowest BIC scores (Bayesian Information Criterion). We found that in this case, the TN92-G model (Tamura 1992) best described the data. We then built a Neighbour-Joining tree (Saitou and Nei 1987) based on TN92-G distances, using 25 nucleotide sequences from each species. All ambiguous positions were removed for each sequence pair. There were a total of 693 positions in the final dataset. For each species of *Hyphantrophaga*, we used a single CO1 DNA sequence for this tree. Twenty-two of the 25 sequences selected belonged to the holotypes designated herein, the remaining three being from ACG vouchers of previously known species.

**Figure 30.**
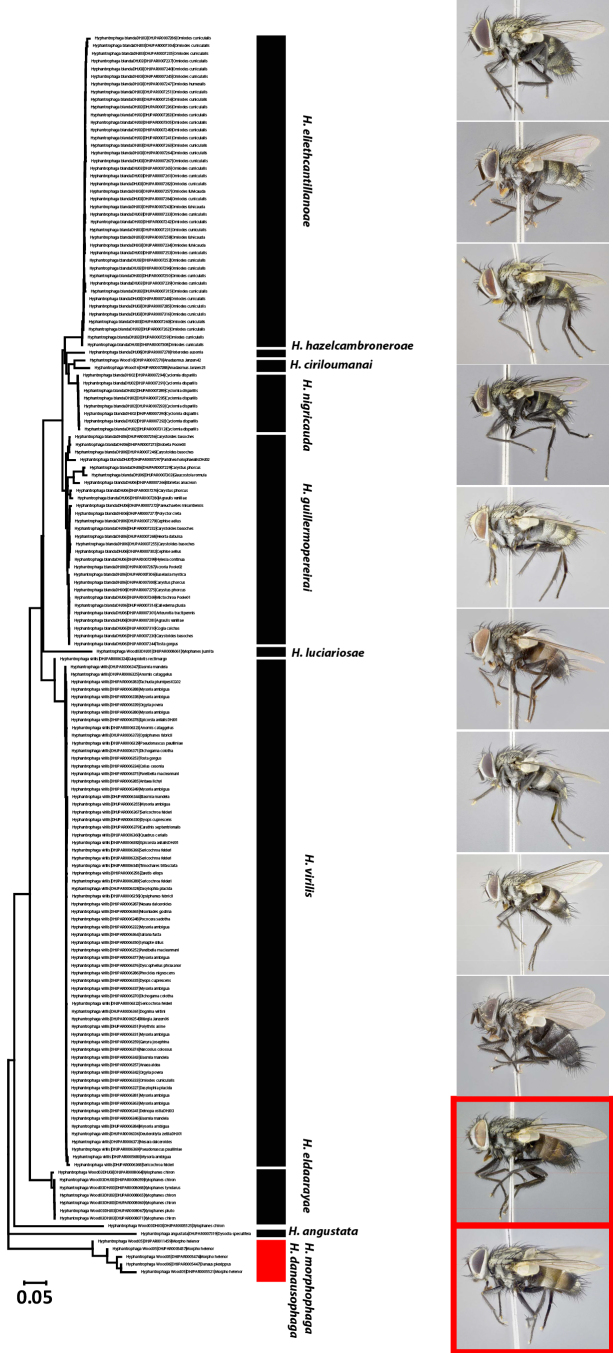
ITS2 NJ tree for *Hyphantrophaga* of ACG. Tip labels are interim species name|sample accession|host family.

For a sub-set of the ACG *Hyphantrophaga* specimens, we also sequenced the ribosomal spacer region ITS2 (Fig. 30). The NJ tree was constructed based on p-dist (Nei and Kumar 2000). The average p-dist between species was 0.25 (all species pairs included). For one species pair (*H. morphophaga* and *H. danausophaga*), there were no observed differences between species.

## Supplementary Material

XML Treatment for Hyphantrophaga

XML Treatment for Hyphantrophaga adrianguadamuzi

XML Treatment for Hyphantrophaga albopilosa

XML Treatment for Hyphantrophaga anacordobae

XML Treatment for Hyphantrophaga angustata

XML Treatment for Hyphantrophaga calixtomoragai

XML Treatment for Hyphantrophaga calva

XML Treatment for Hyphantrophaga ciriloumanai

XML Treatment for Hyphantrophaga danausophaga

XML Treatment for Hyphantrophaga diniamartinezae

XML Treatment for Hyphantrophaga duniagarciae

XML Treatment for Hyphantrophaga edwinapui

XML Treatment for Hyphantrophaga eldaarayae

XML Treatment for Hyphantrophaga eliethcantillanoae

XML Treatment for Hyphantrophaga gilberthampiei

XML Treatment for Hyphantrophaga guillermopereirai

XML Treatment for Hyphantrophaga hazelcambroneroae

XML Treatment for Hyphantrophaga luciariosae

XML Treatment for Hyphantrophaga manuelriosi

XML Treatment for Hyphantrophaga morphophaga

XML Treatment for Hyphantrophaga myersi

XML Treatment for Hyphantrophaga nigricauda

XML Treatment for Hyphantrophaga osvaldoespinozai

XML Treatment for Hyphantrophaga pabloumanai

XML Treatment for Hyphantrophaga similis

XML Treatment for Hyphantrophaga virilis

## Figures and Tables

**Figure 1a.**
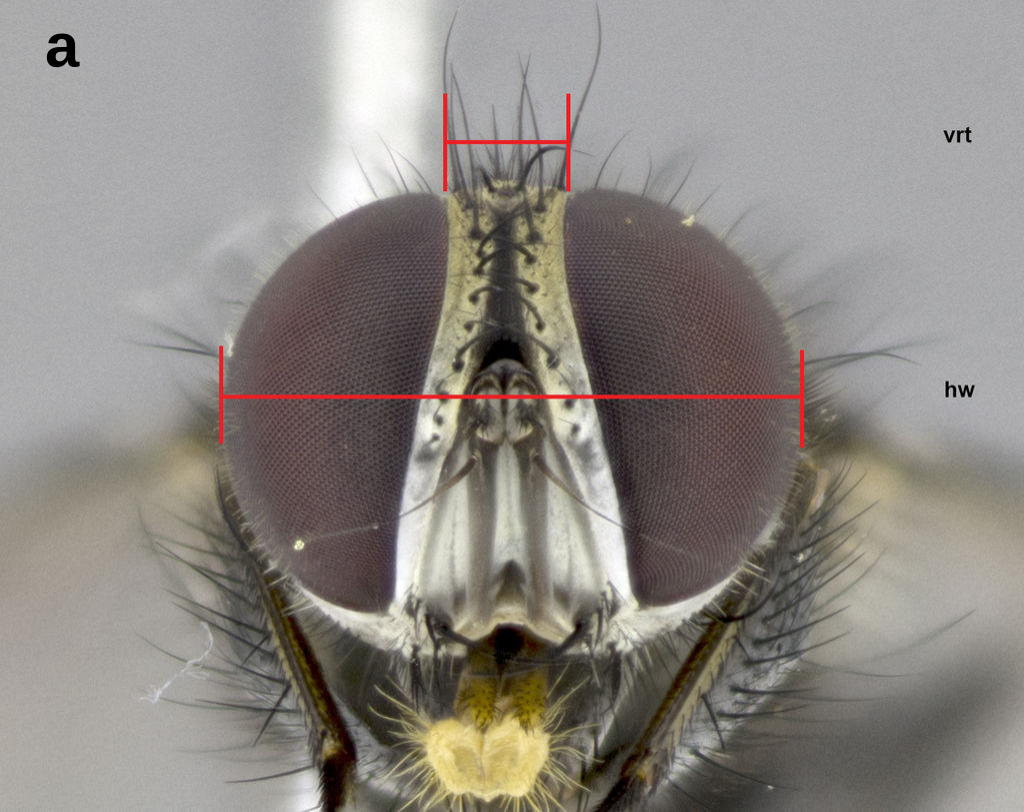
Sample of measured areas from front of head as shown on *Hyphantrophaga virilis* (Aldrich & Webber, 1924); vrt, vertex; hw, head width

**Figure 1b.**
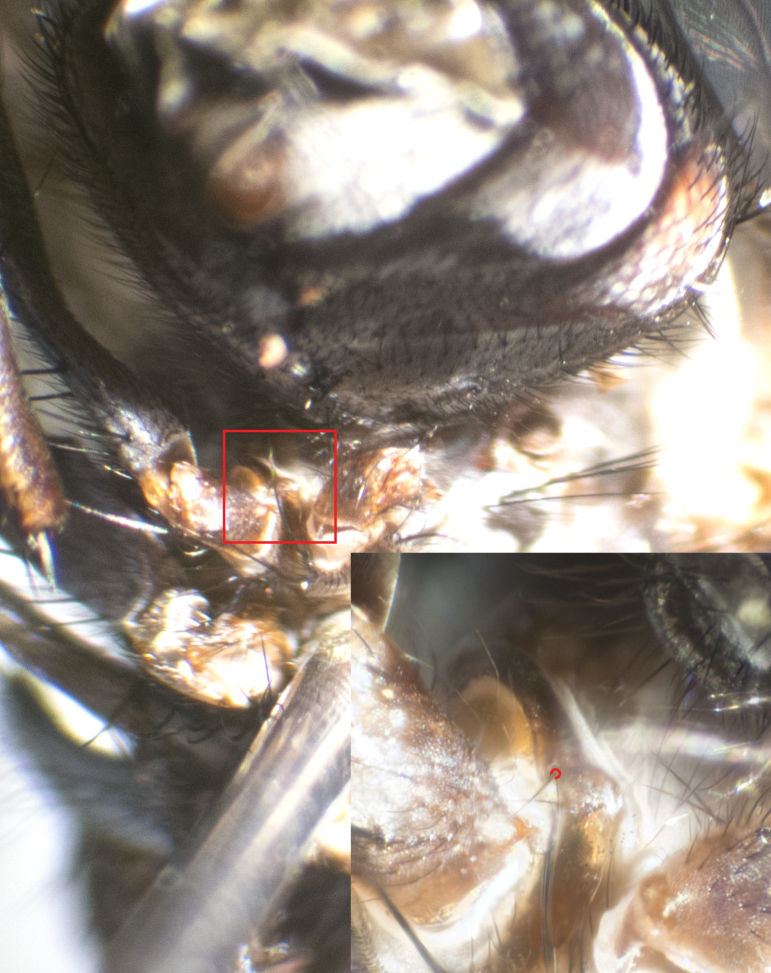
Detailed view of hind coxa showing setose hind margin in *Hyphantrophaga virilis*

**Figure 1c.**
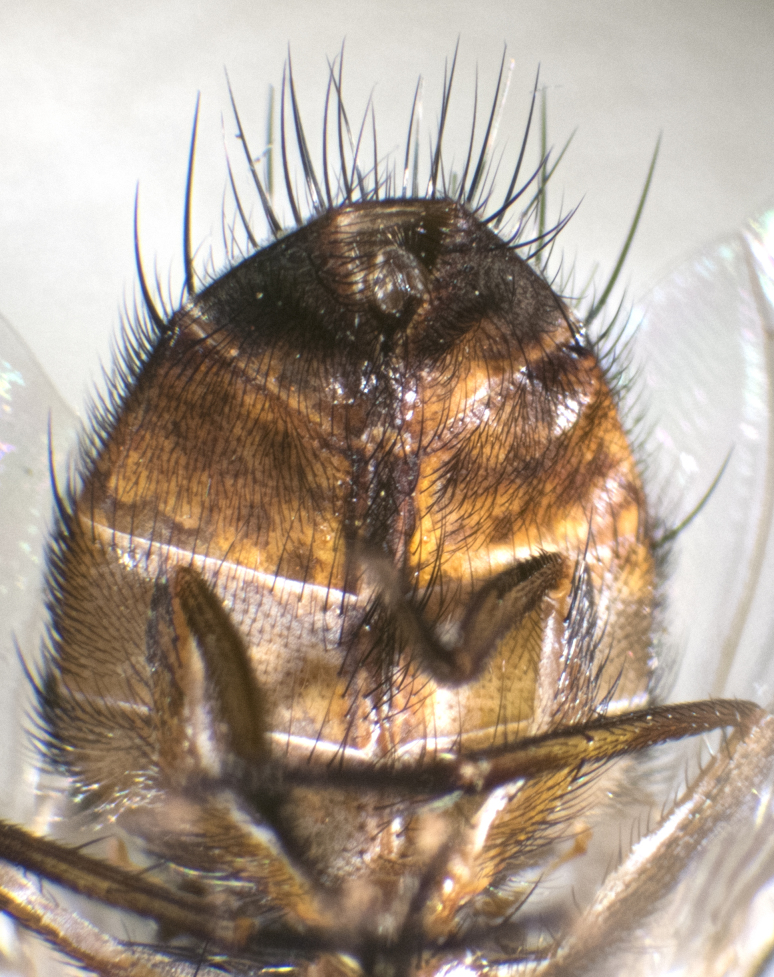
Ventral view of abdomen in *Hyphantrophaga albopilosa* **sp. n.** showing presence of sex patches on T4 and T5

**Figure 1d.**
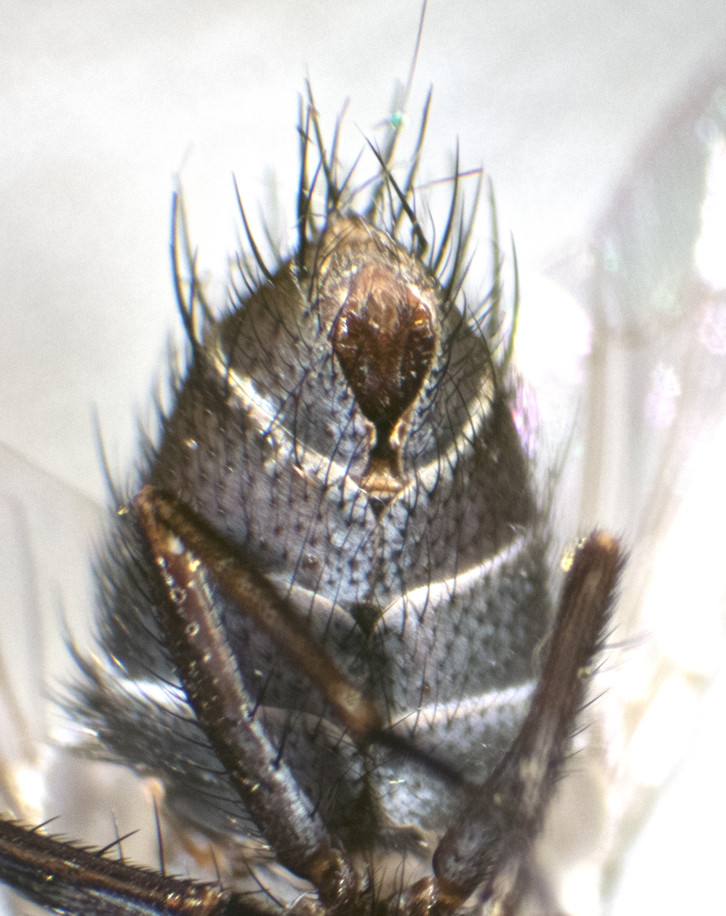
Ventral view of abdomen in *Hyphantrophaga guillermopereirai* **sp. n.** showing absence of sex patches

**Figure 1e.**
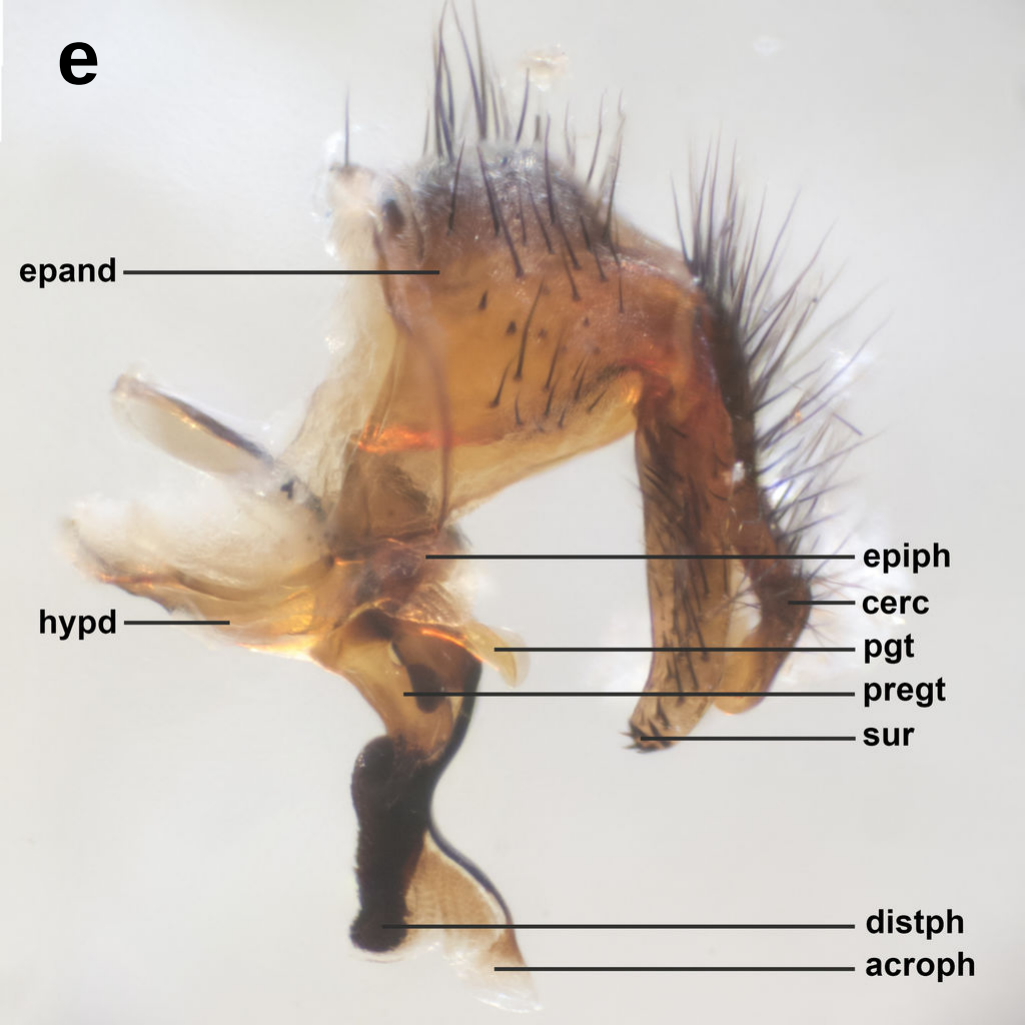
Detailed lateral view of male genitalia of *Hyphantrophaga nigricauda* **sp. n.** Abbreviations: acroph = acrophallus; cerc = cercus; distph = distiphallus; epand = epandrium; epiph = epiphallus; hypd = hypandrium; pgt = postgonite; pregt = pregonite; sur = surstylus

**Figure 1f.**
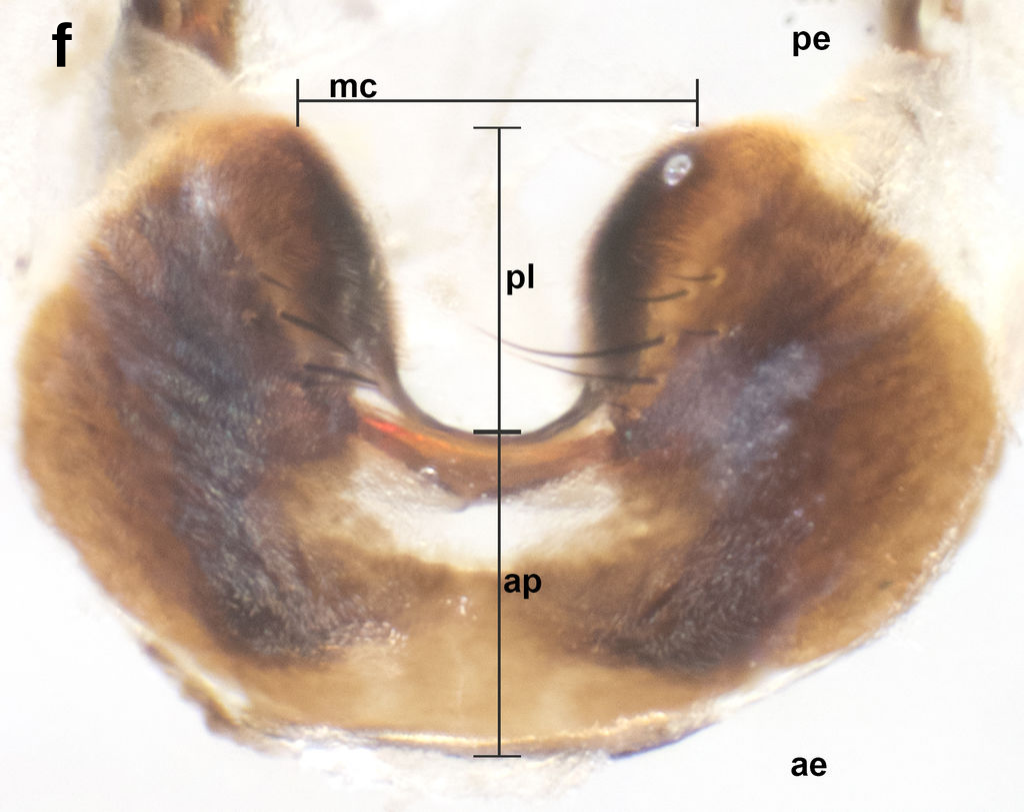
Detailed ventral view of sternite 5 of *Hyphantrophaga myersi* (Aldrich, 1933). Abbreviations: ae = anterior edge; ap = anterior plate; mc = median cleft; pe = posterior edge; pl = posterior lobes

**Figure 2a.**
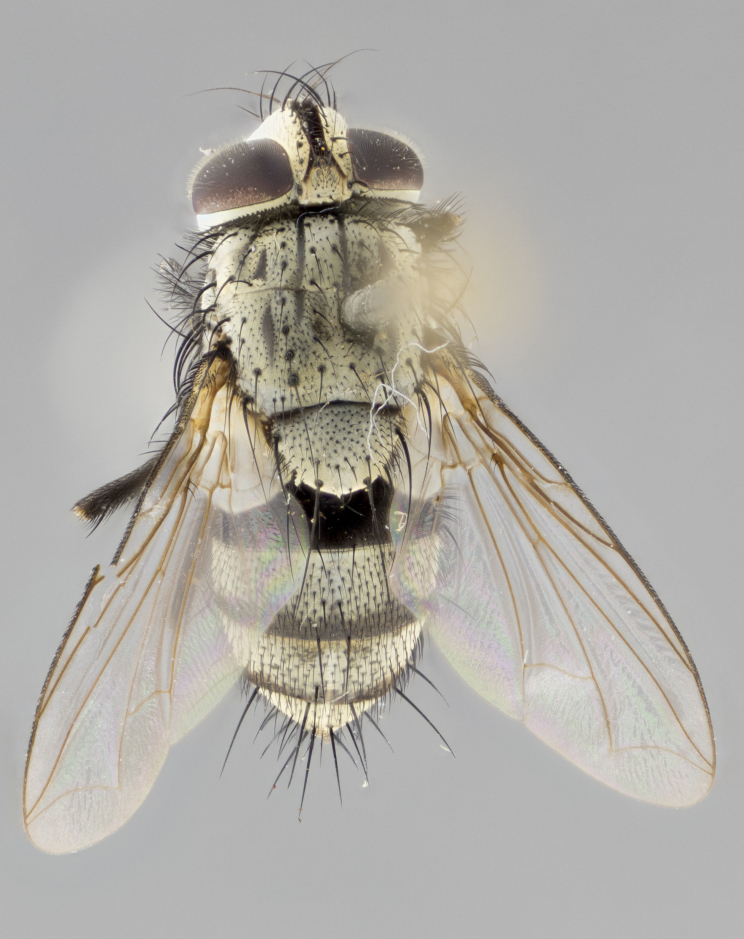
dorsal view

**Figure 2b.**
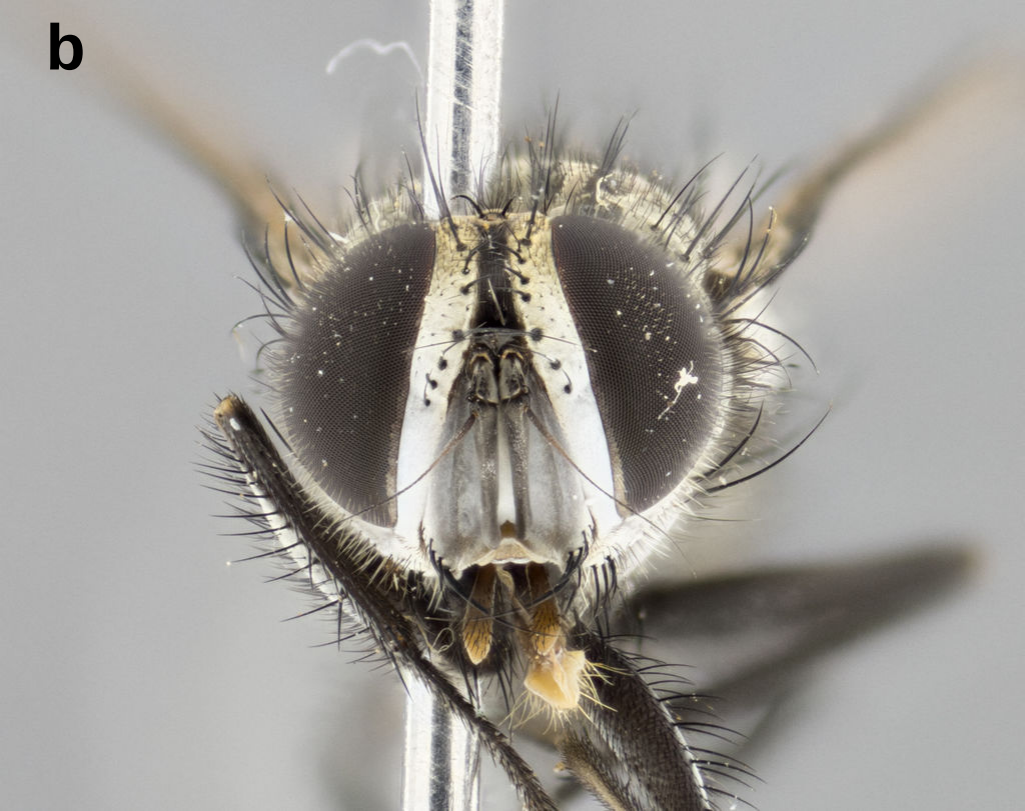
frontal view

**Figure 2c.**
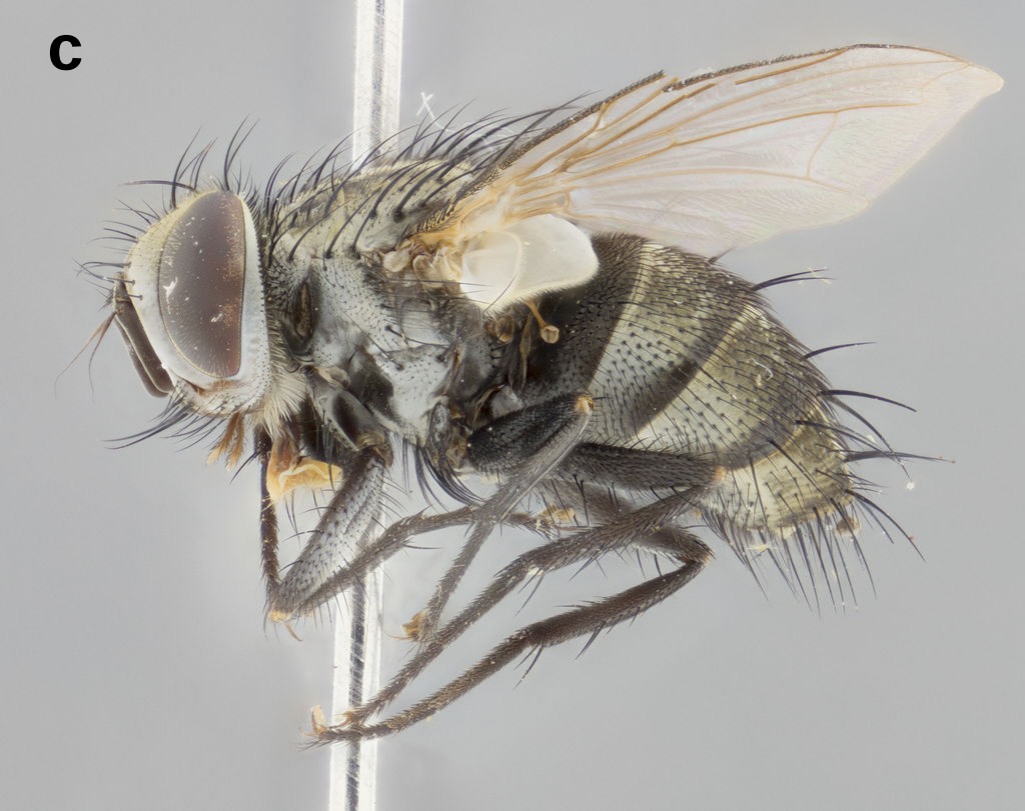
lateral view

**Figure 2d.**
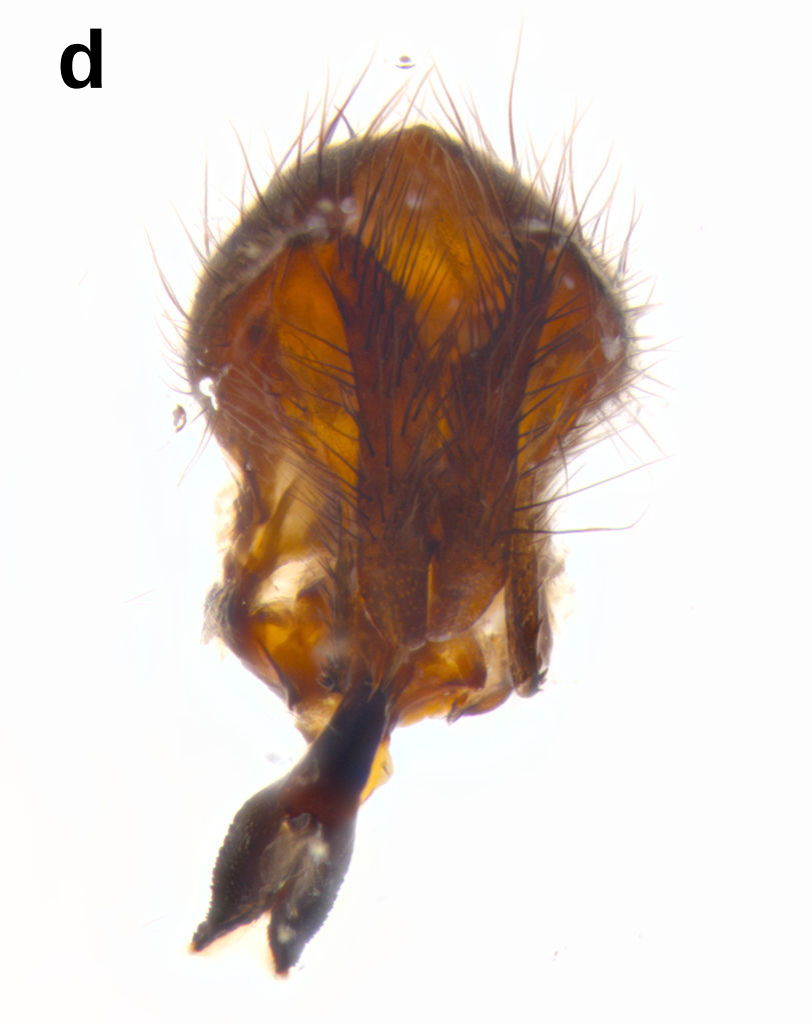
dorsal view

**Figure 2e.**
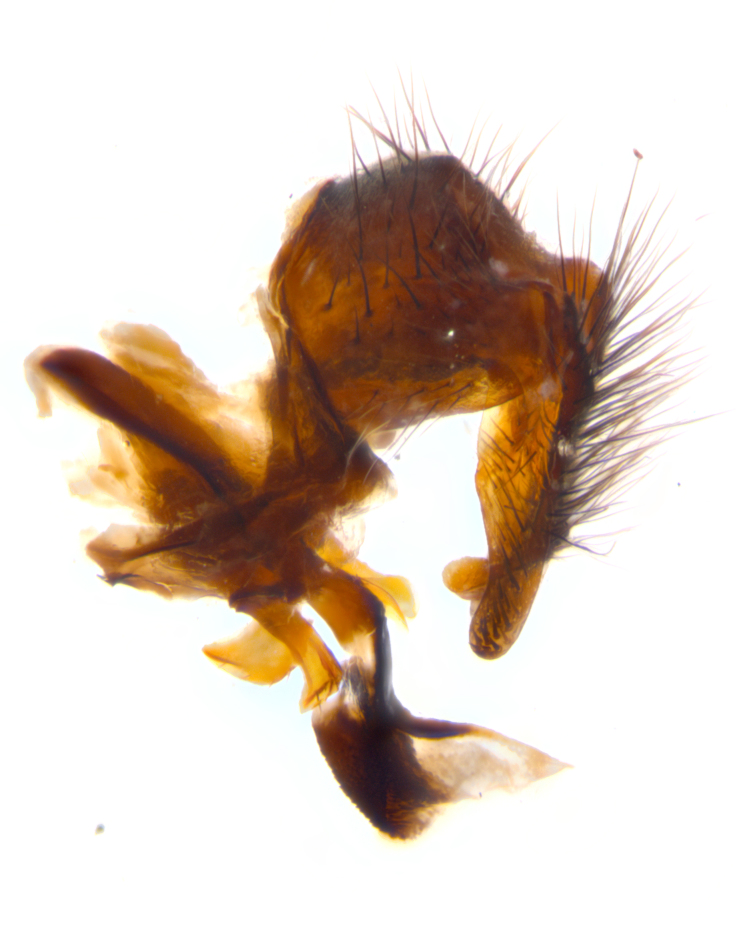
lateral view

**Figure 2f.**
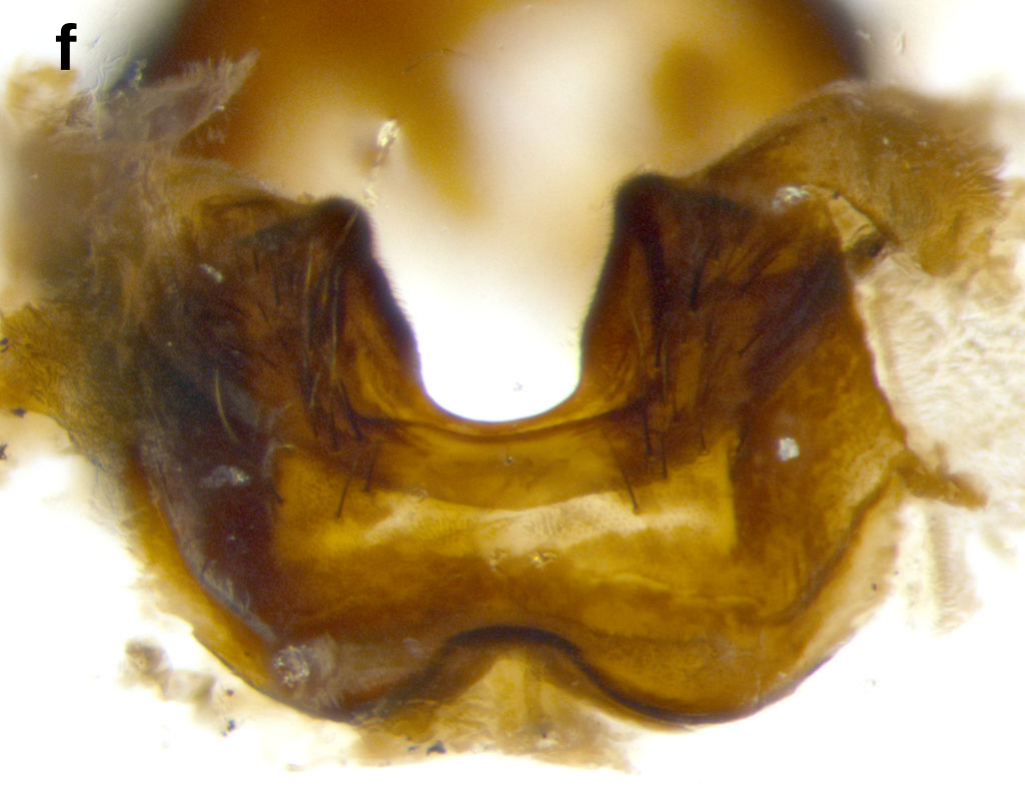
sternite 5, ventral view

**Figure 3a.**
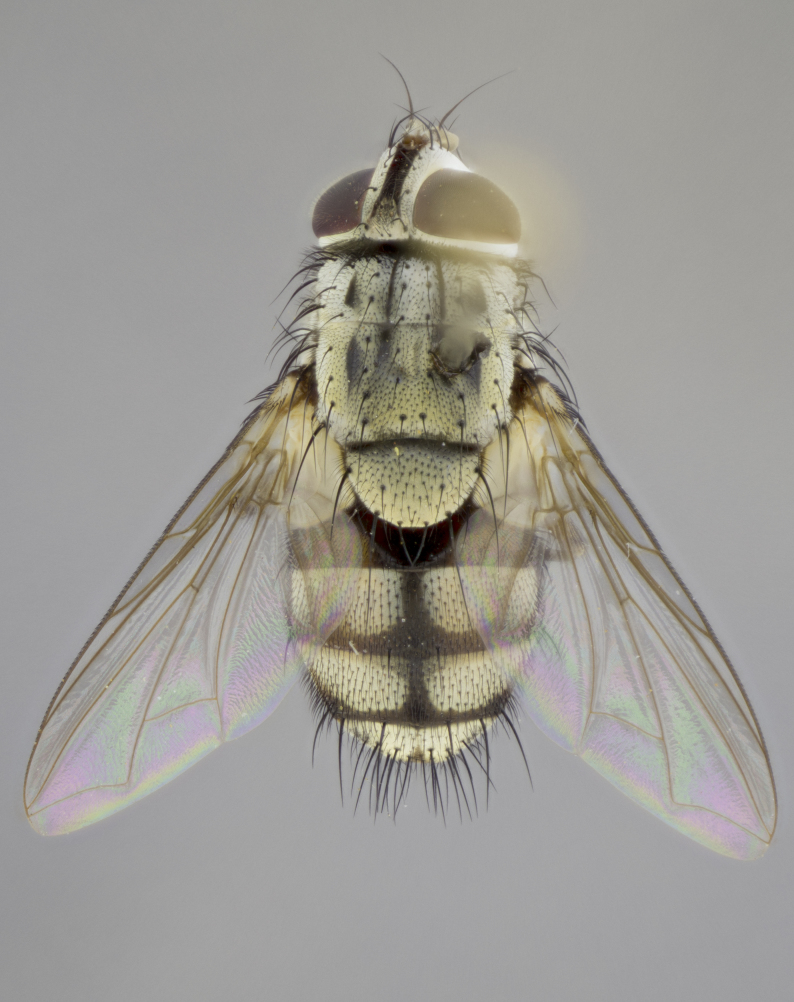
dorsal view

**Figure 3b.**
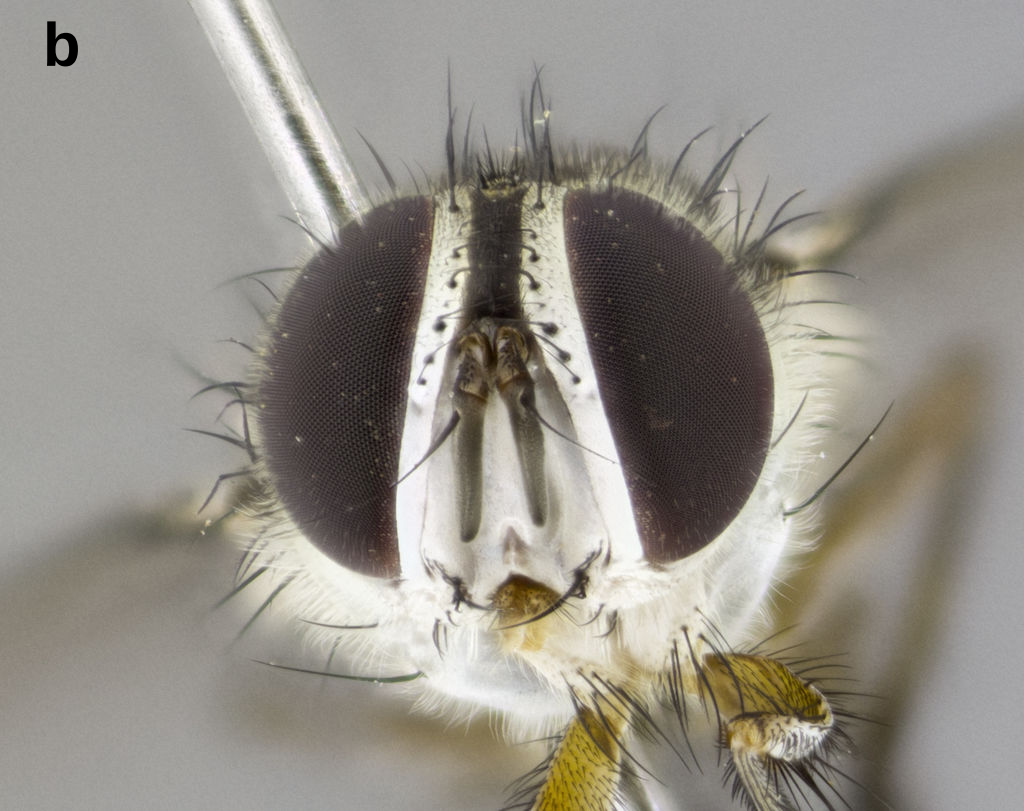
frontal view

**Figure 3c.**
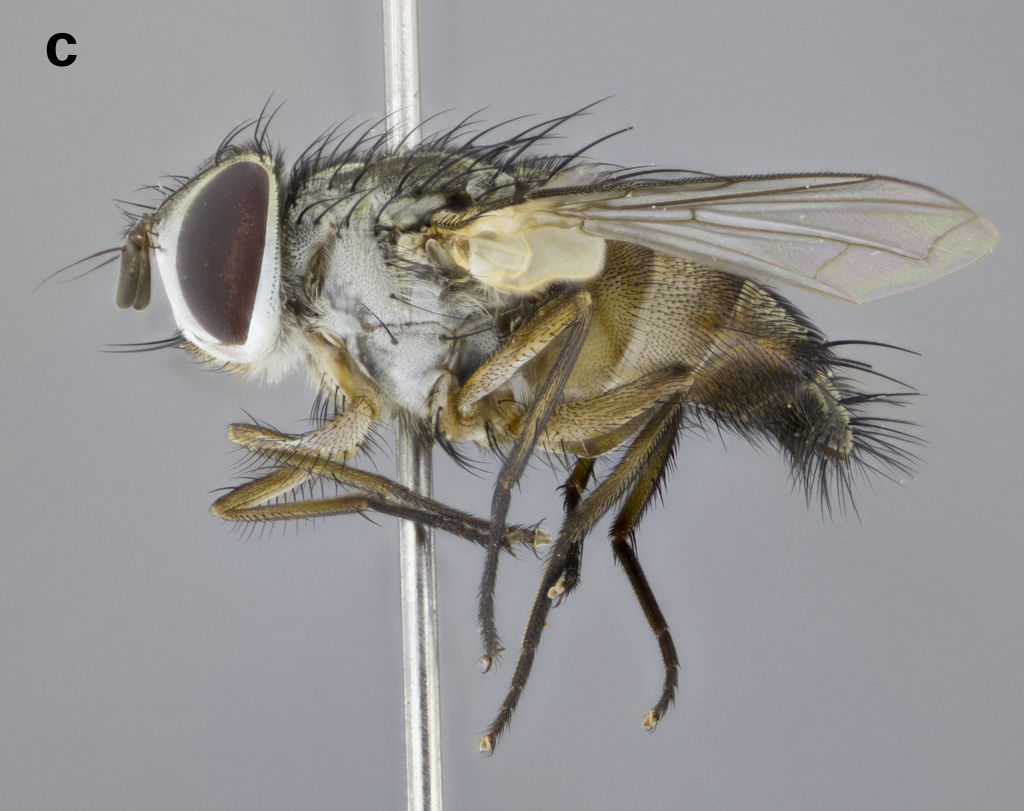
lateral view

**Figure 3d.**
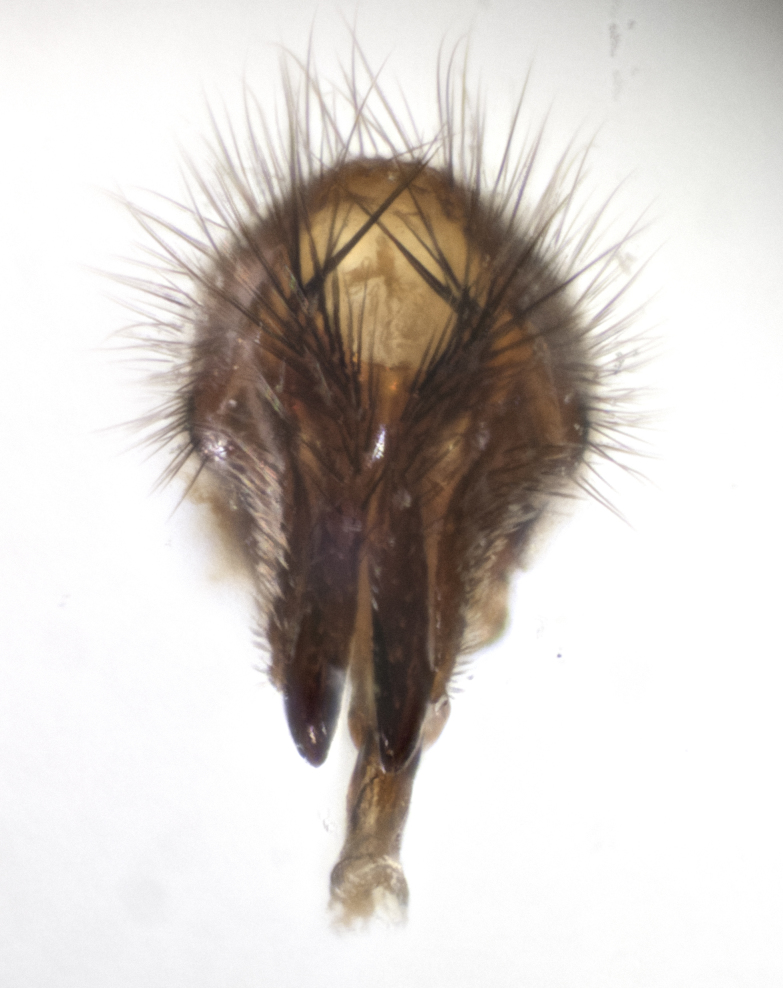
dorsal view

**Figure 3e.**
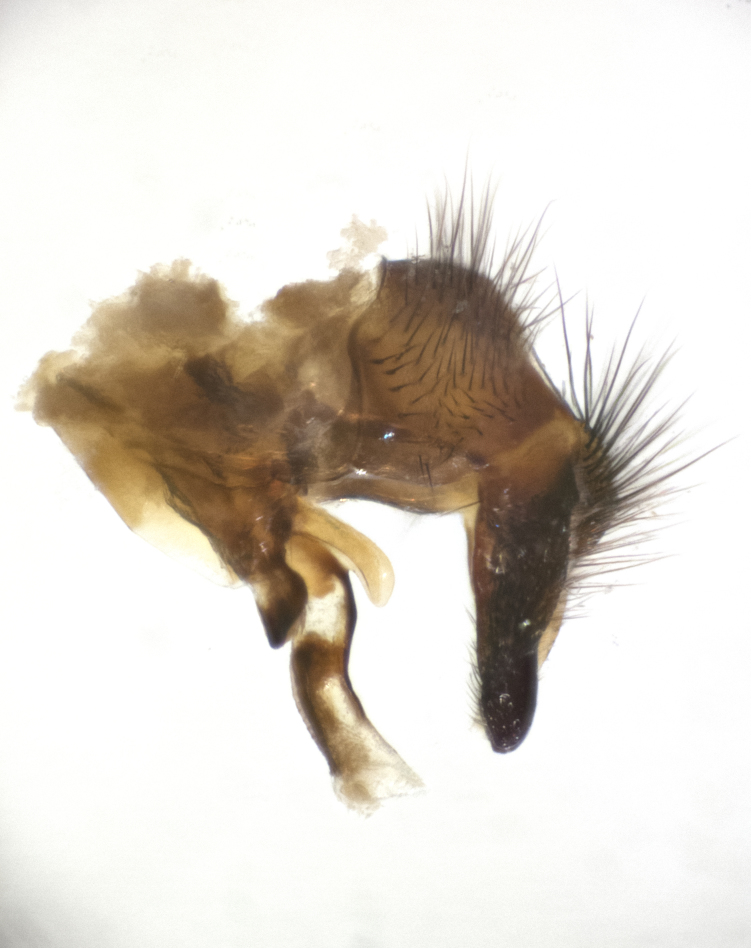
lateral view

**Figure 3f.**
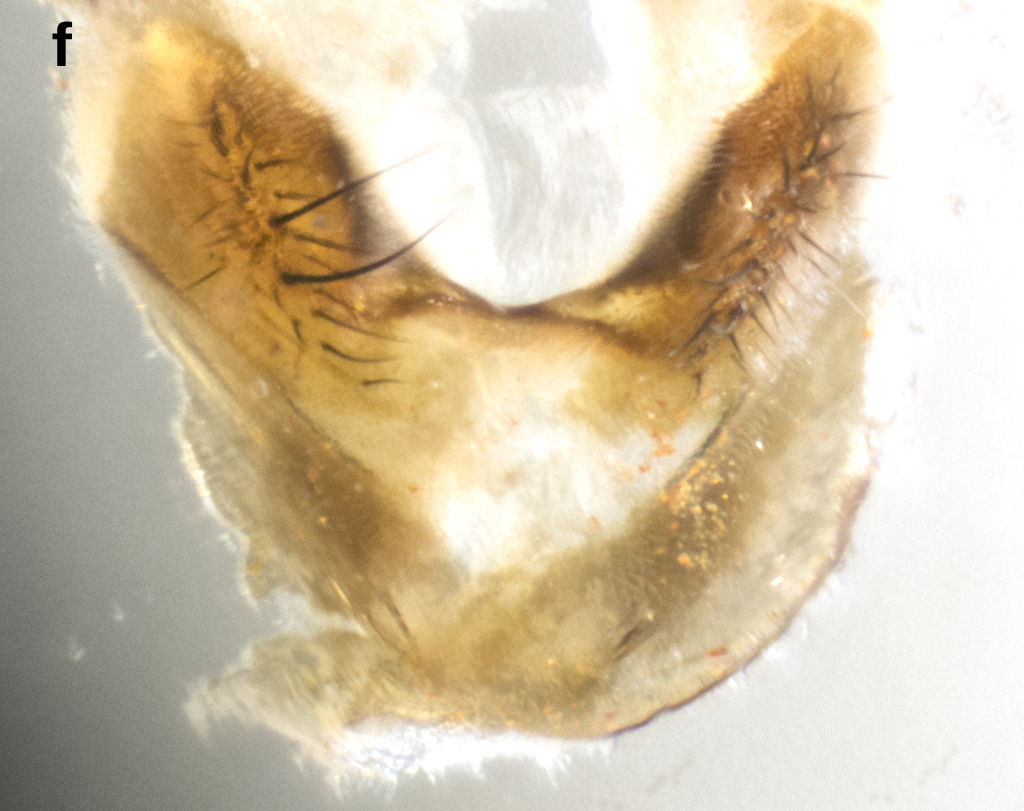
sternite 5, ventral view

**Figure 4a.**
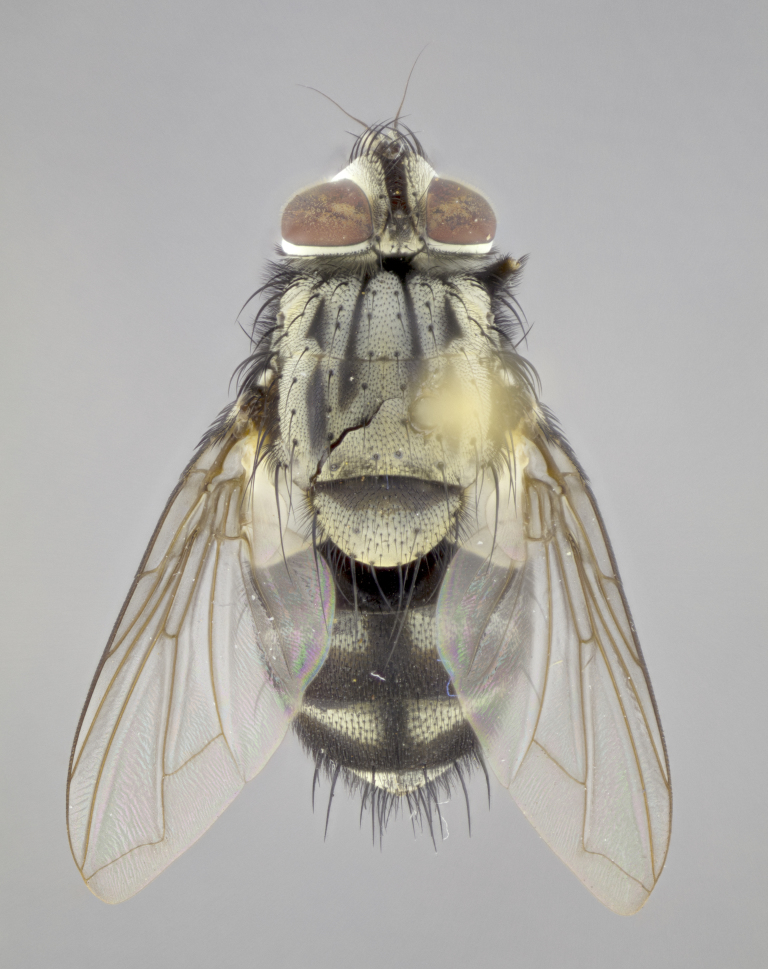
dorsal view

**Figure 4b.**
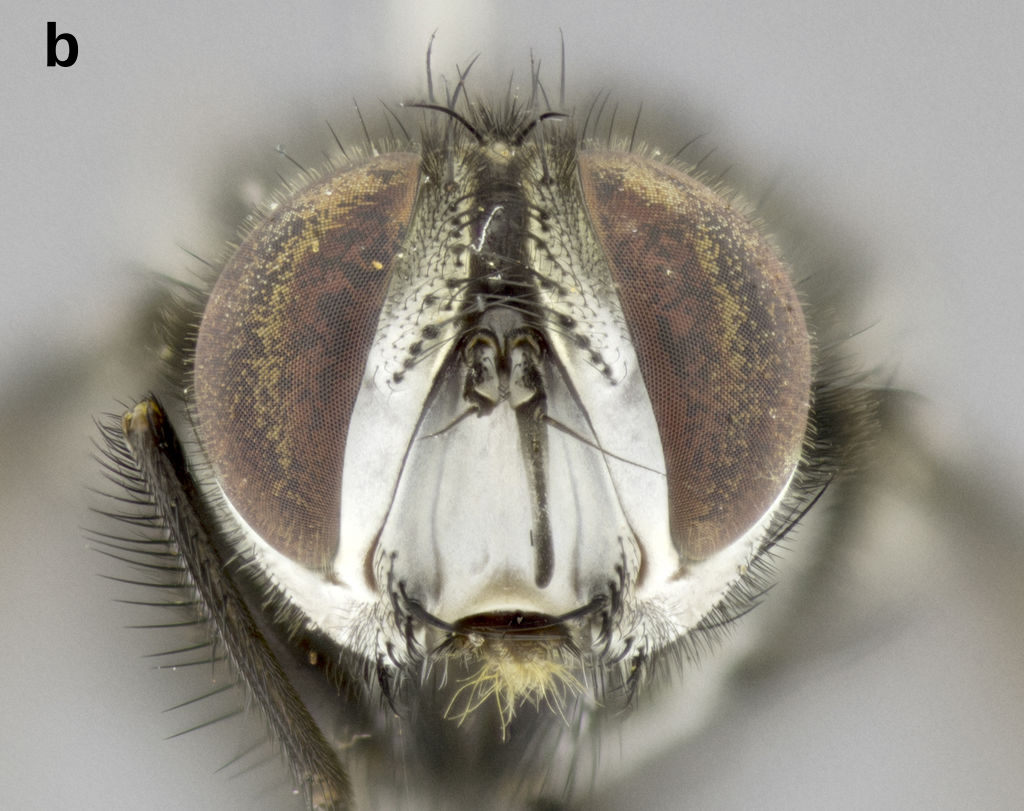
frontal view

**Figure 4c.**
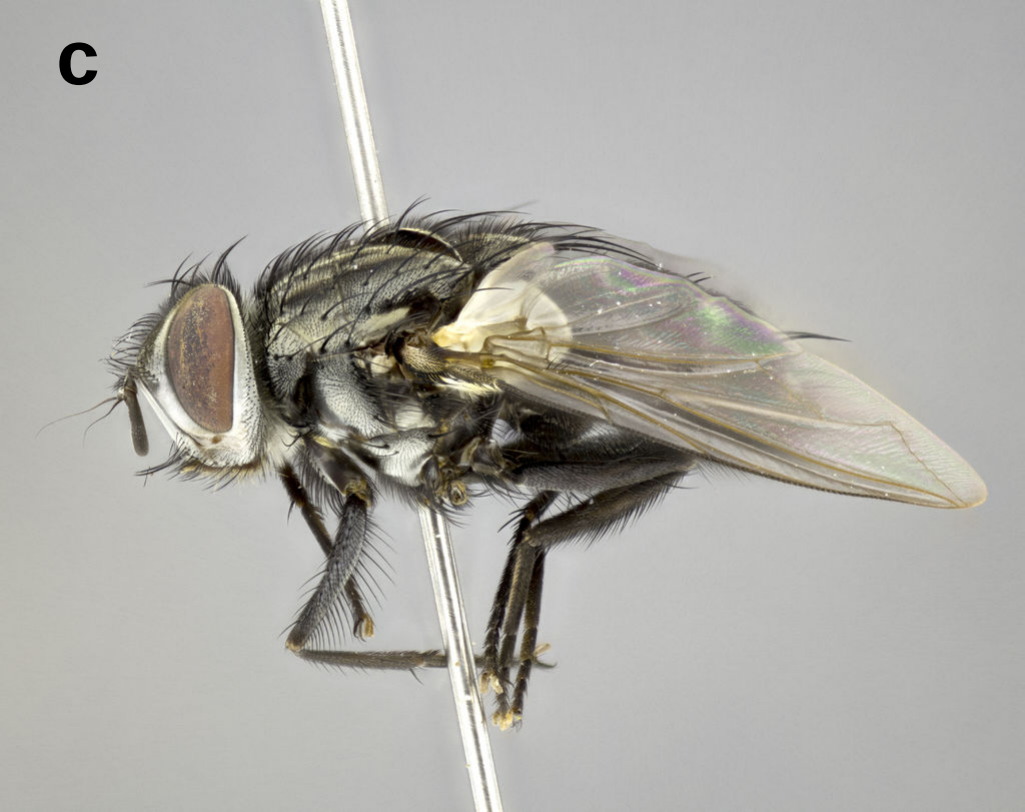
lateral view

**Figure 4d.**
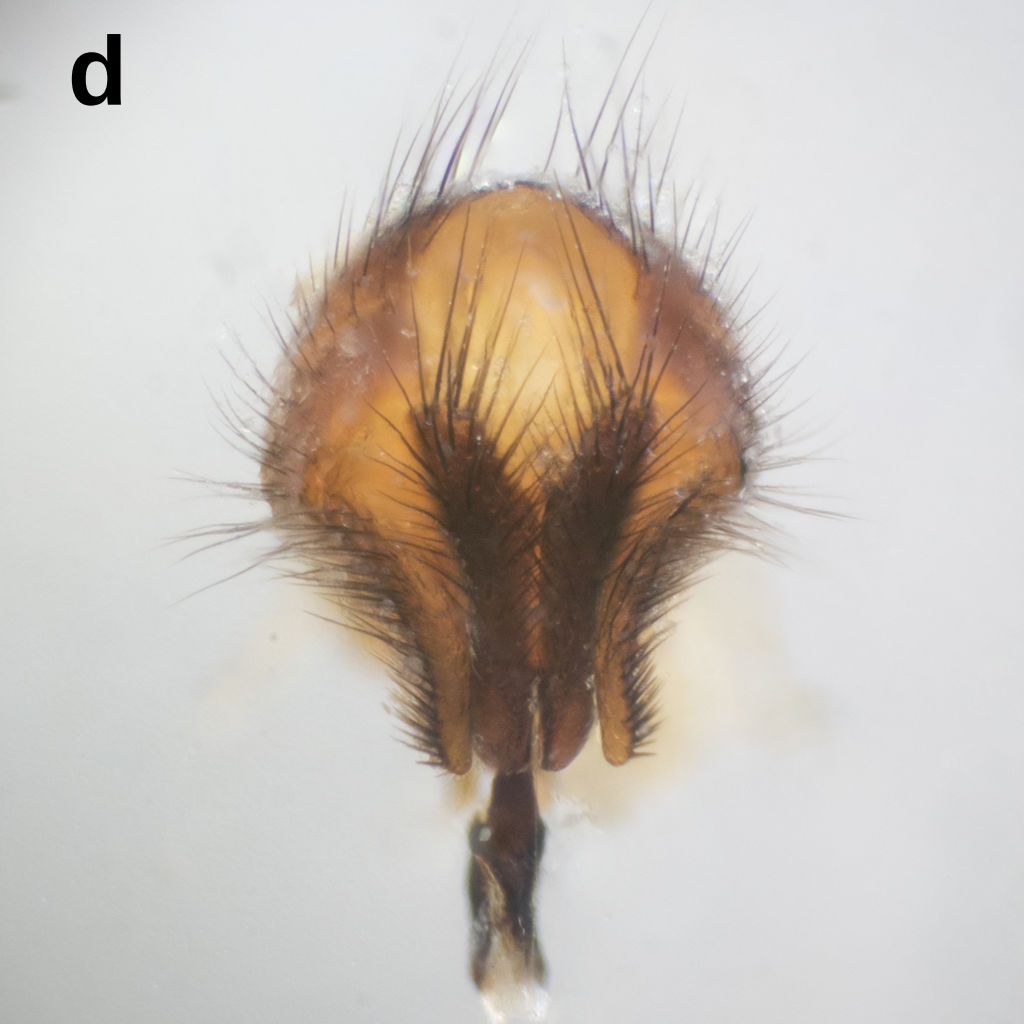
dorsal view

**Figure 4e.**
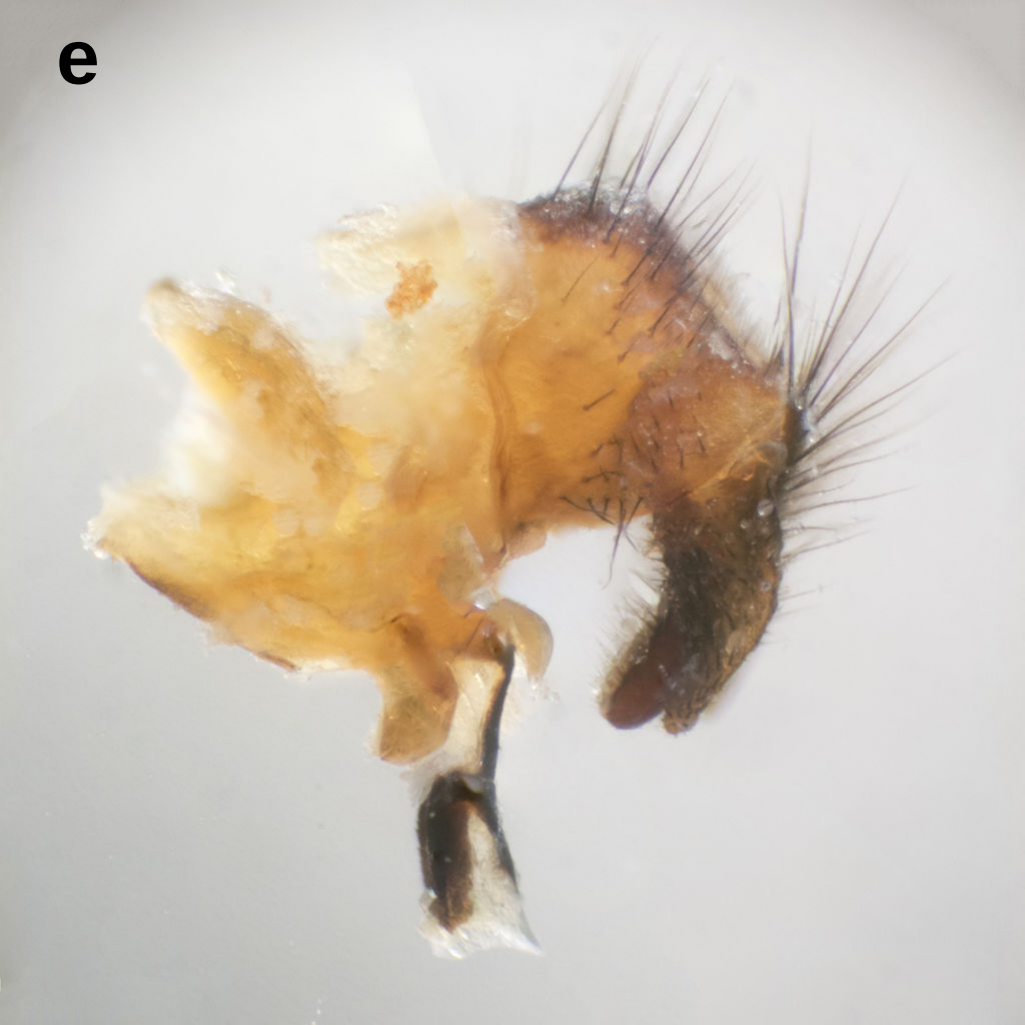
lateral view

**Figure 4f.**
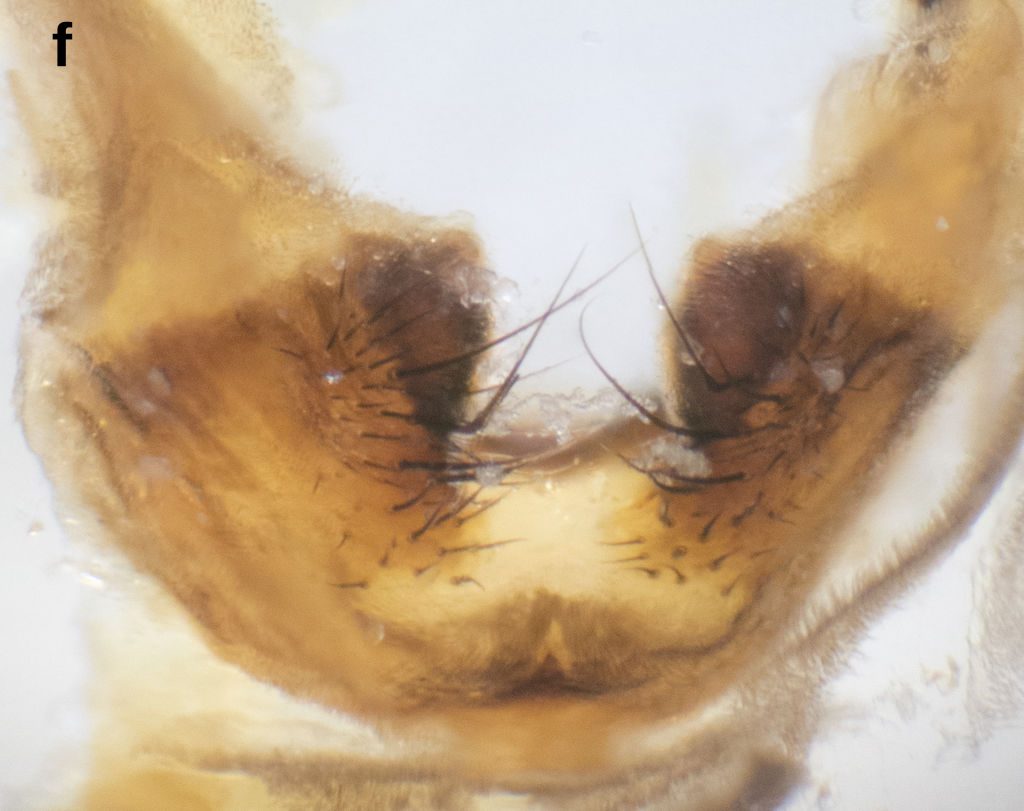
sternite 5, ventral view

**Figure 5a.**
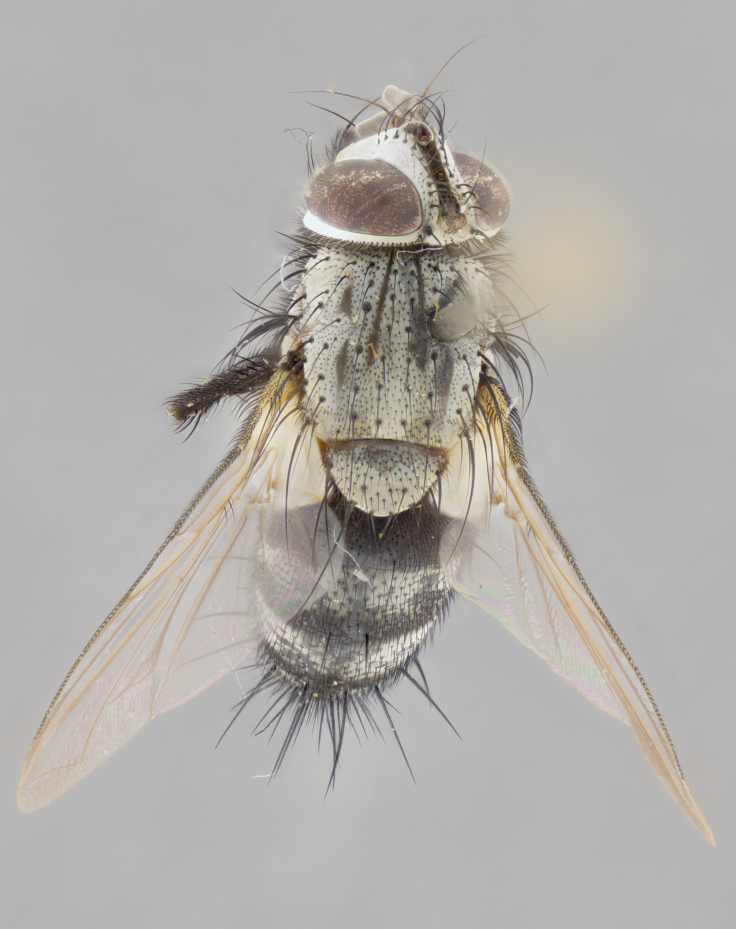
dorsal view

**Figure 5b.**
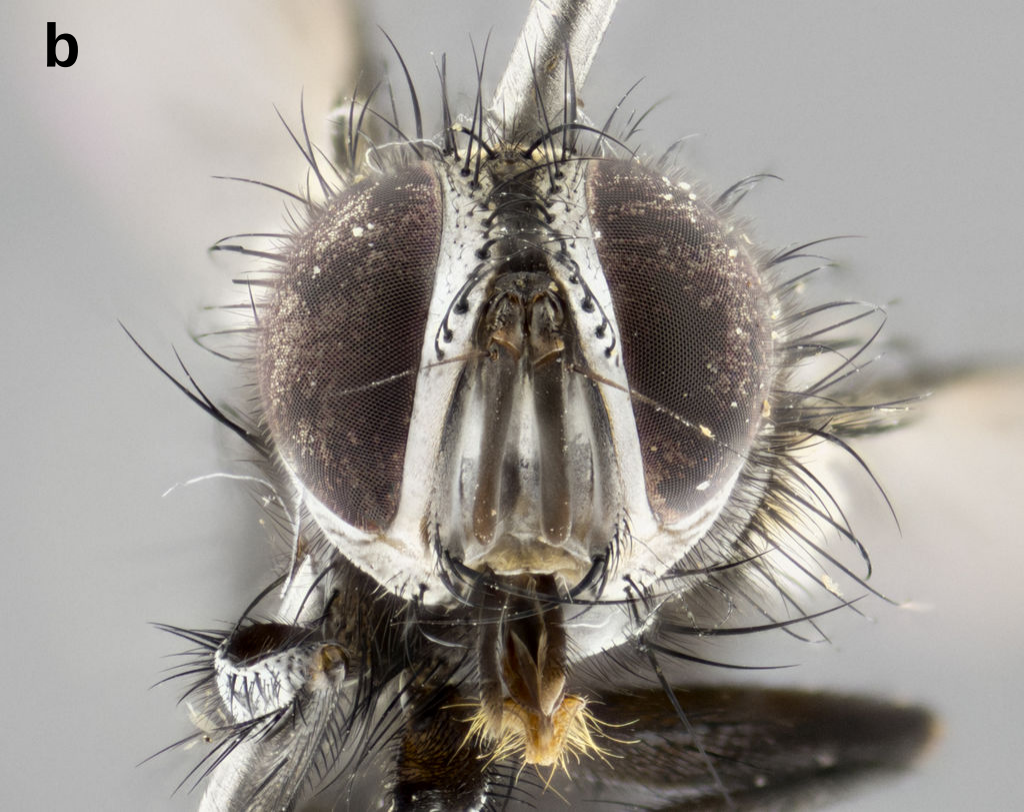
frontal view

**Figure 5c.**
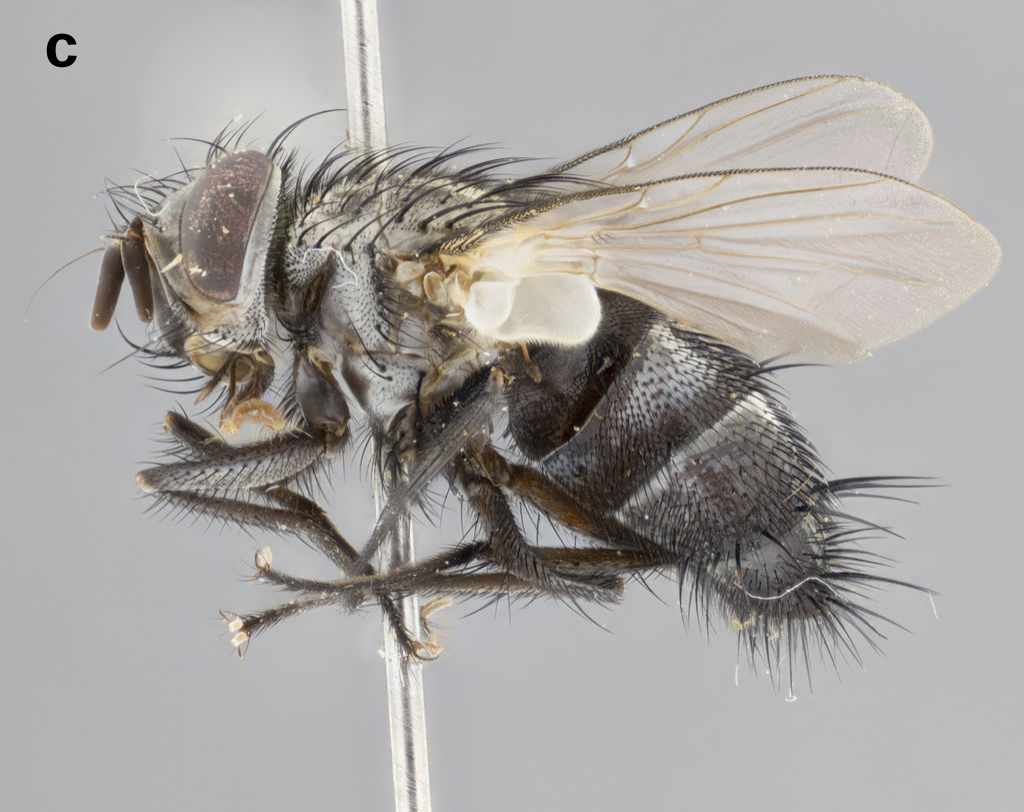
lateral view

**Figure 5d.**
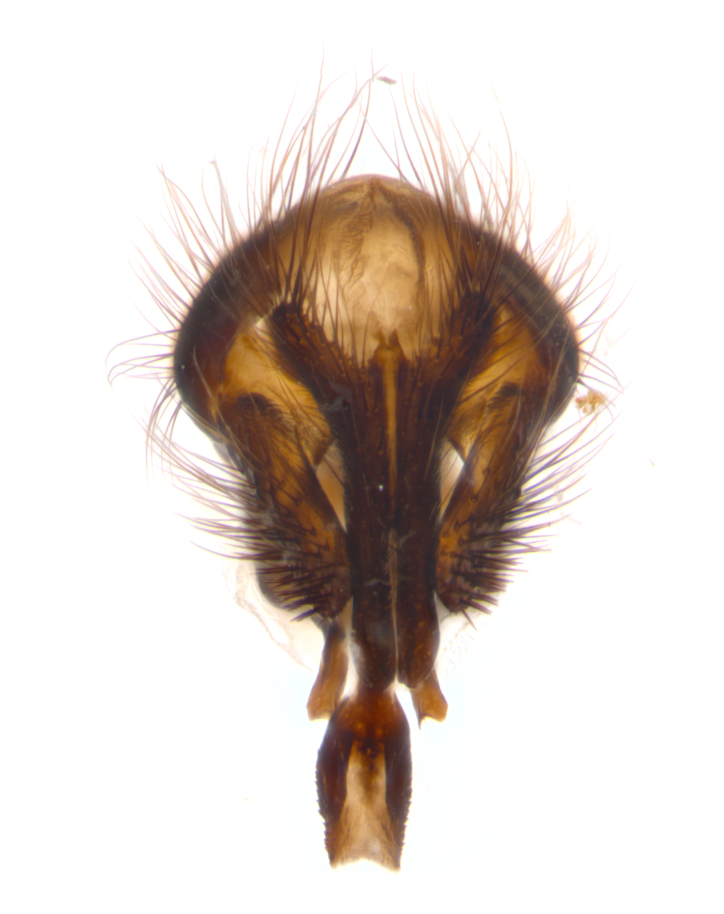
dorsal view

**Figure 5e.**
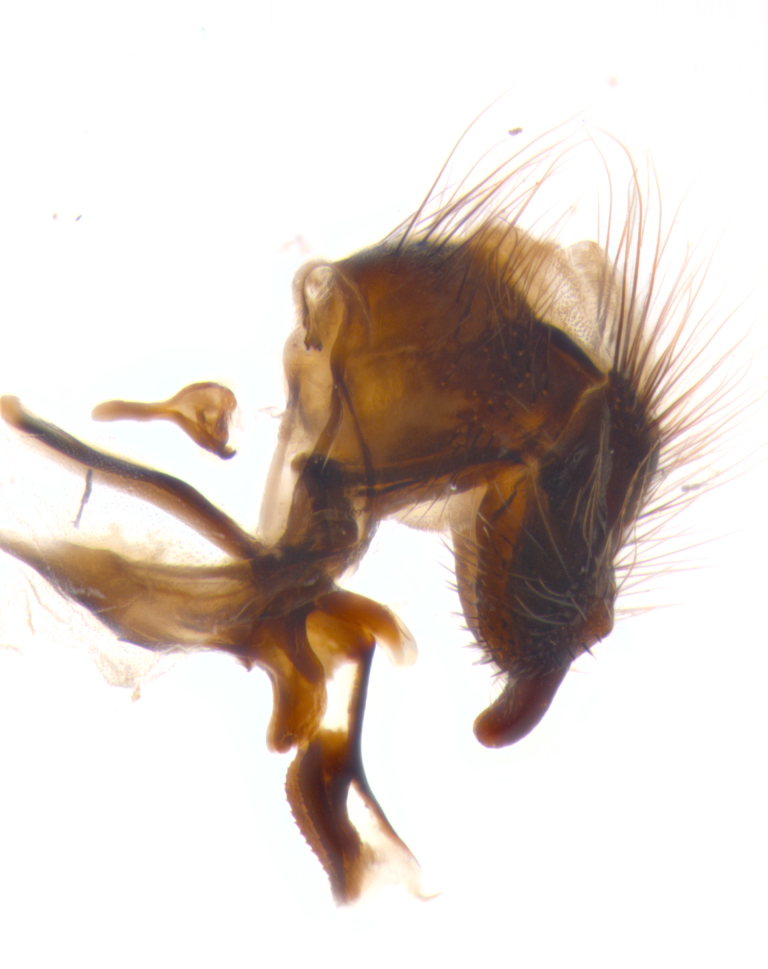
lateral view

**Figure 5f.**
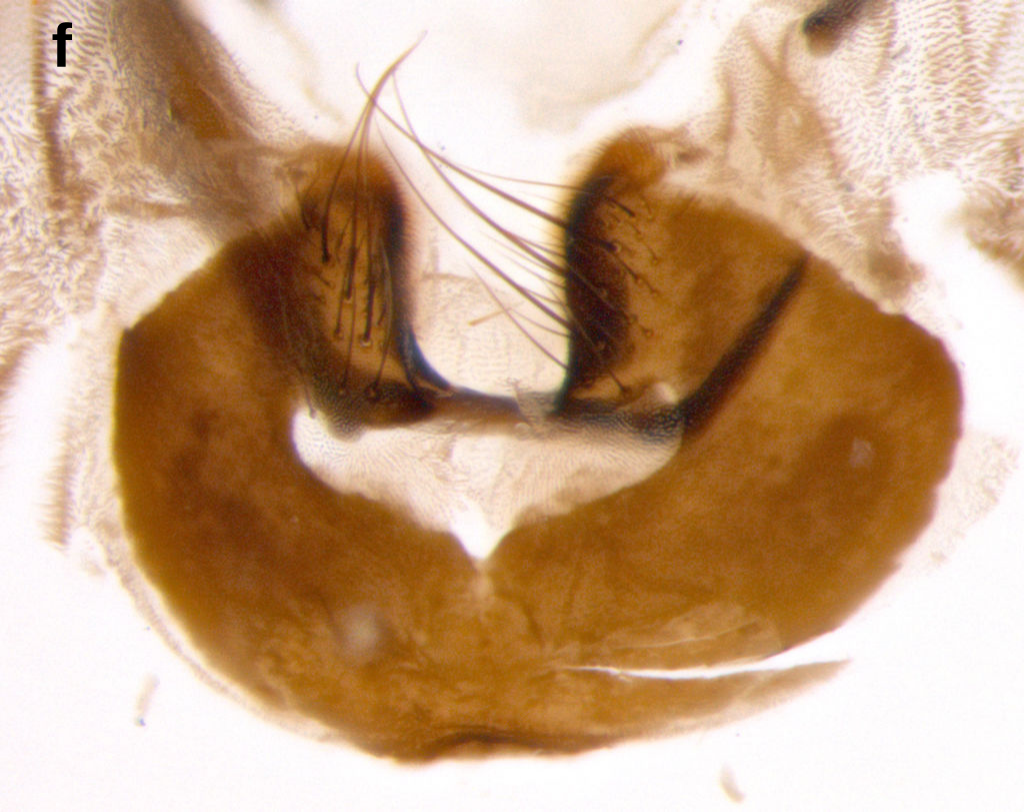
sternite 5, ventral view

**Figure 6a.**
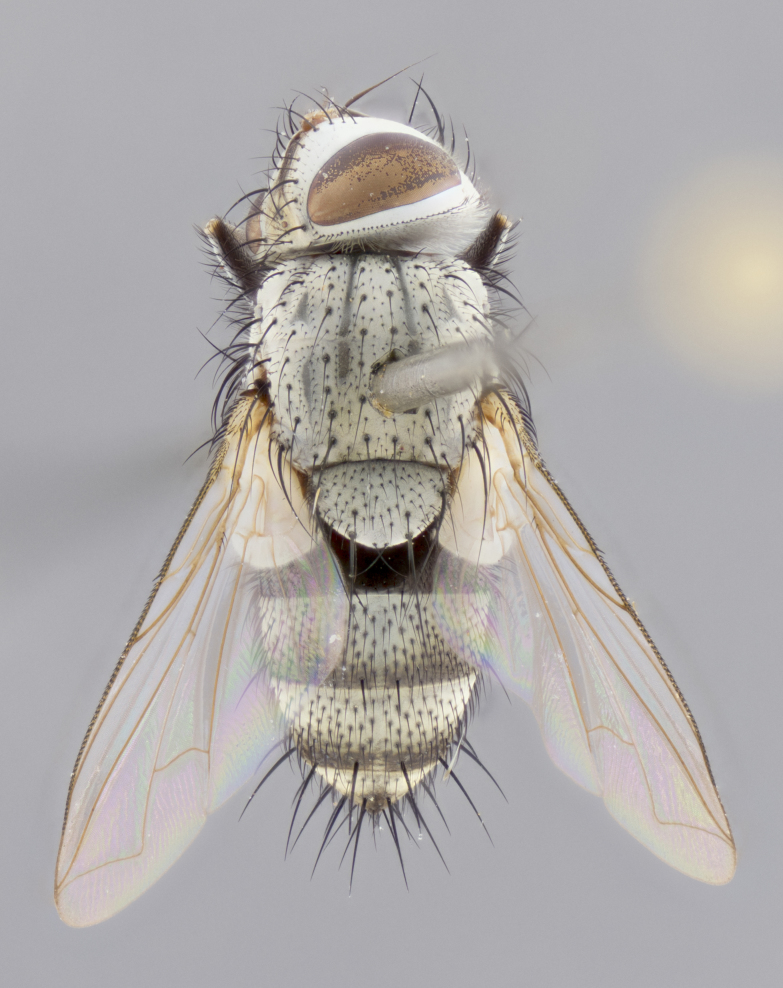
dorsal view

**Figure 6b.**
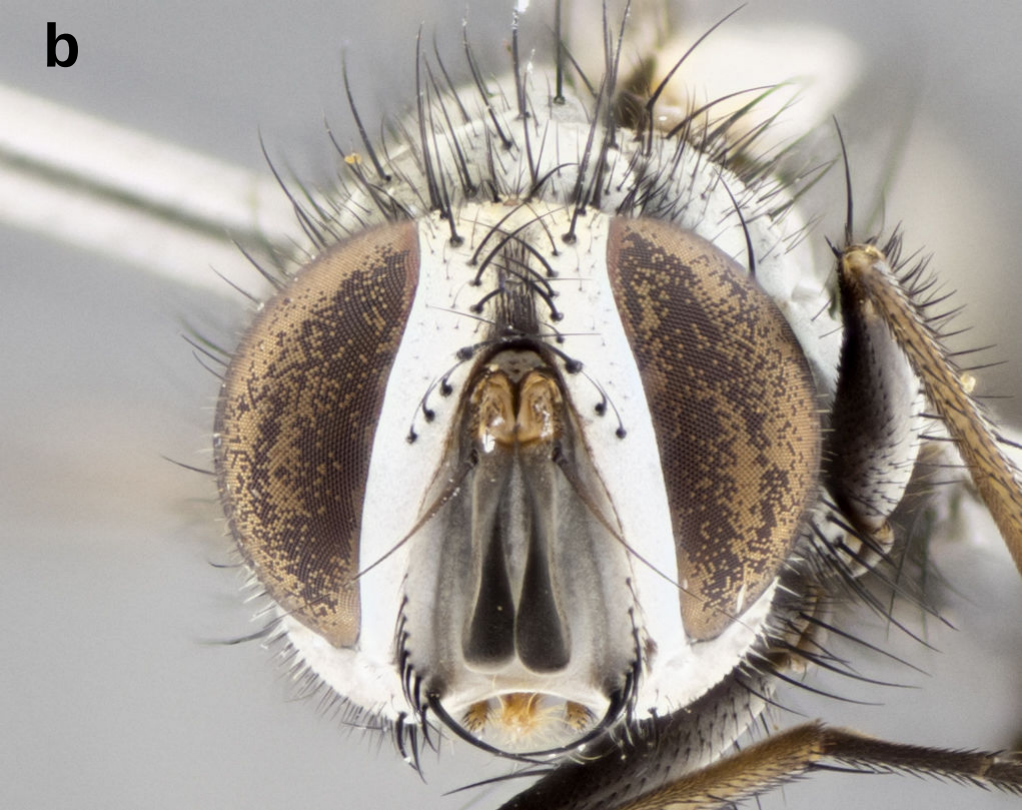
frontal view

**Figure 6c.**
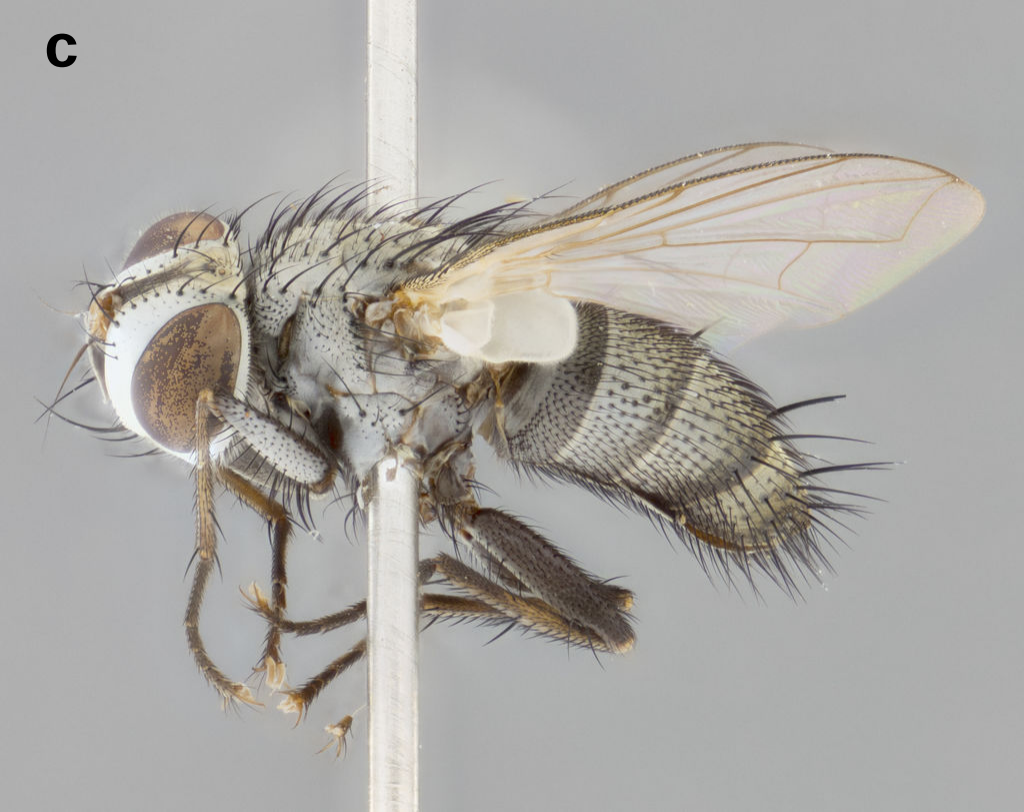
lateral view

**Figure 6d.**
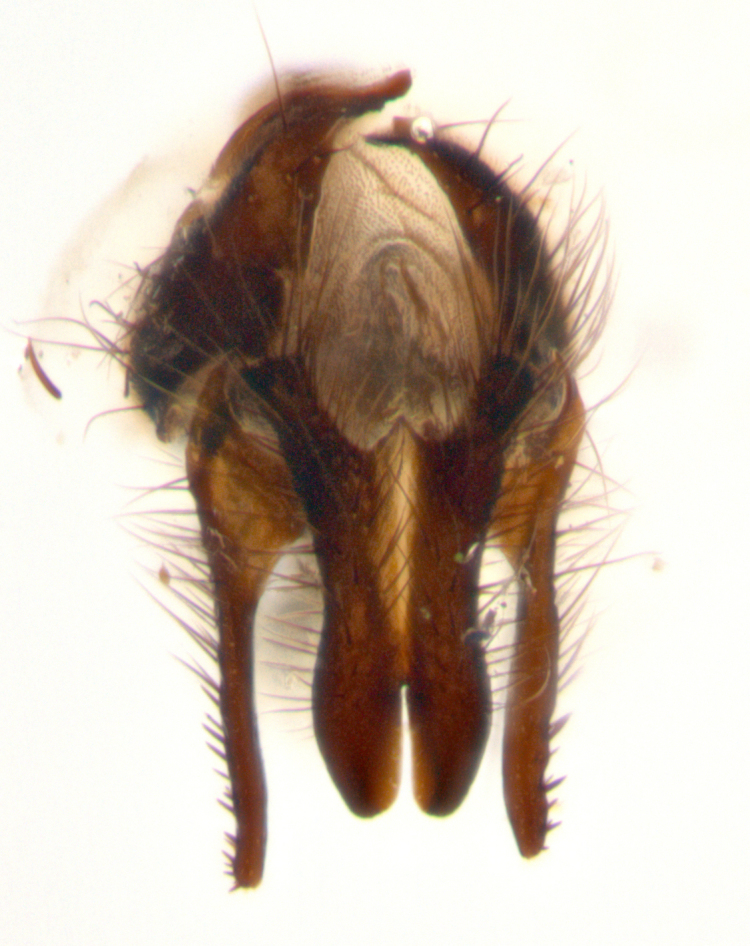
dorsal view

**Figure 6e.**
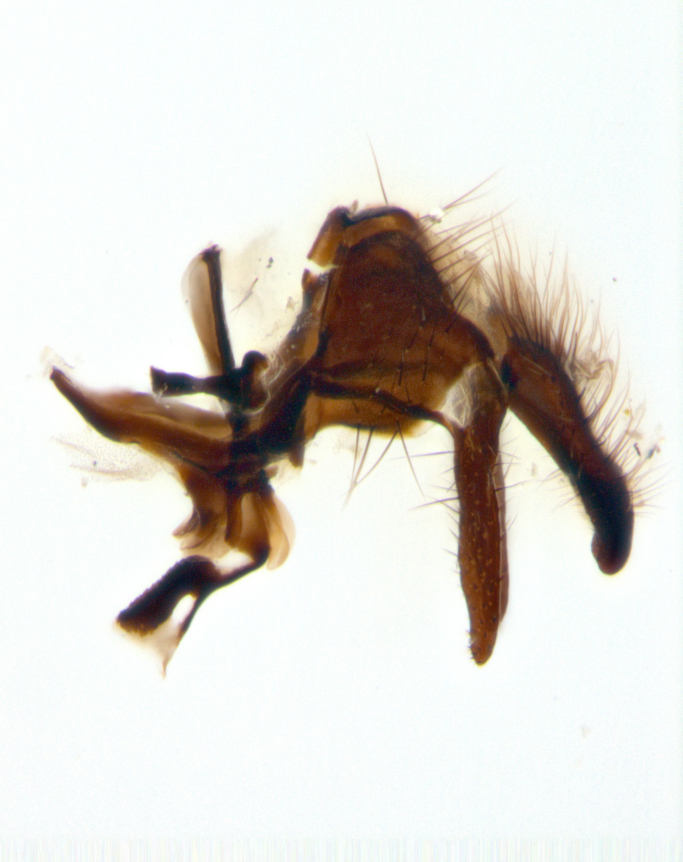
lateral view

**Figure 6f.**
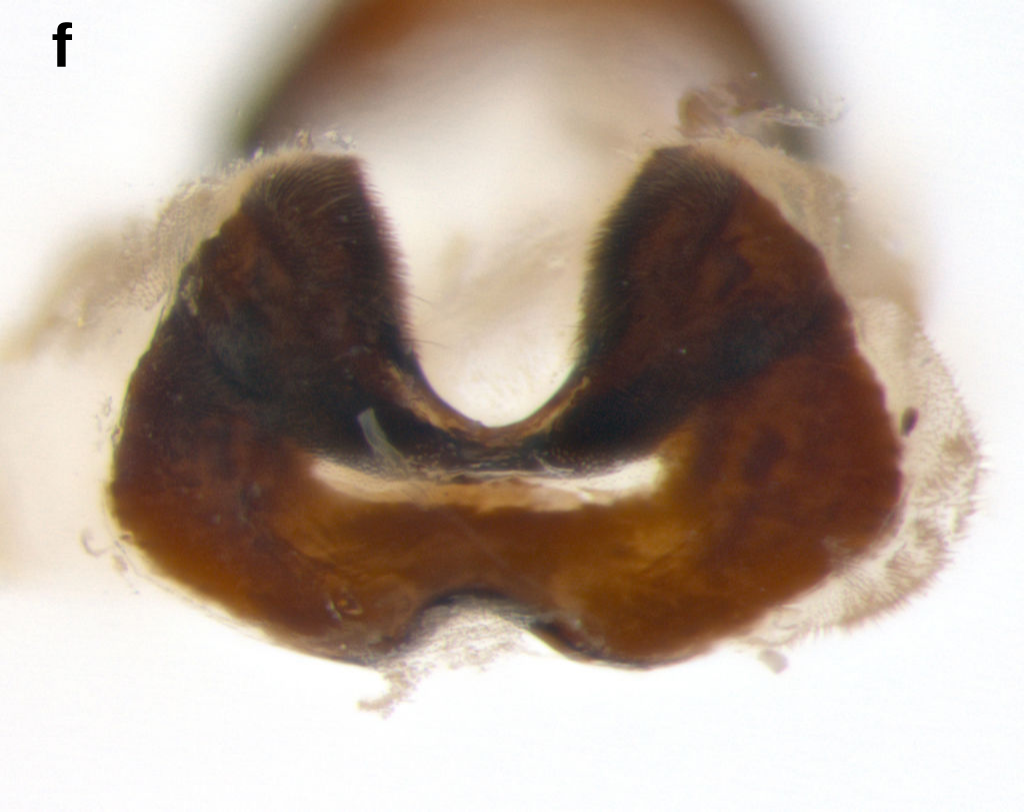
sternite 5, ventral view

**Figure 7a.**
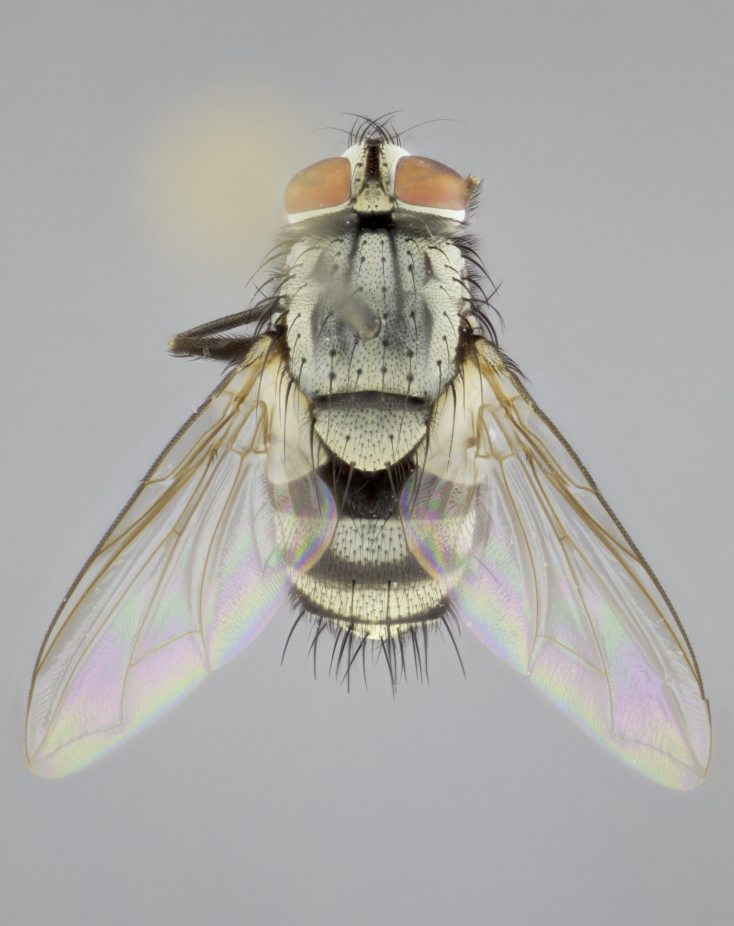
dorsal view

**Figure 7b.**
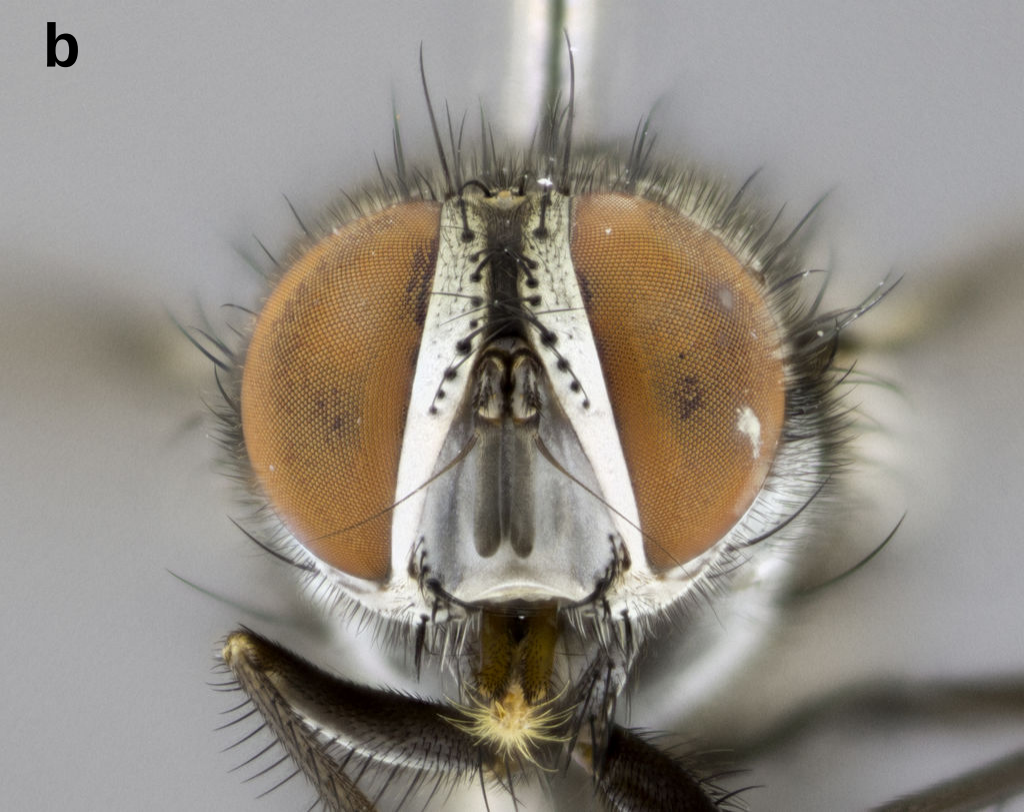
frontal view

**Figure 7c.**
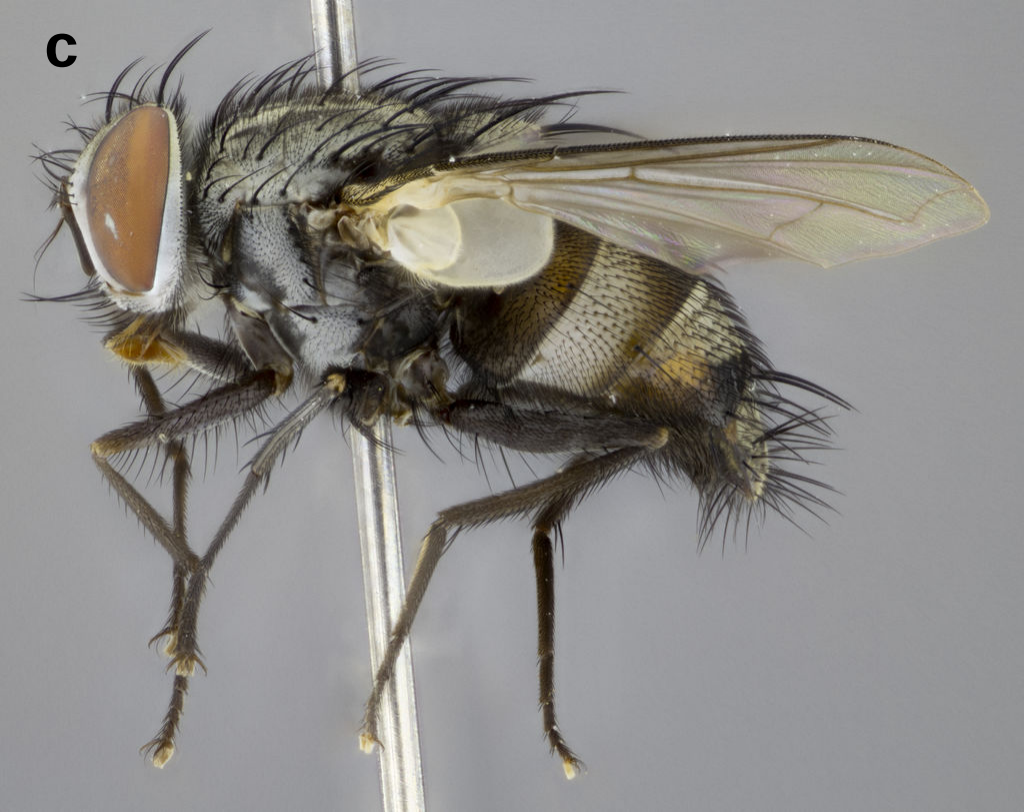
lateral view

**Figure 7d.**
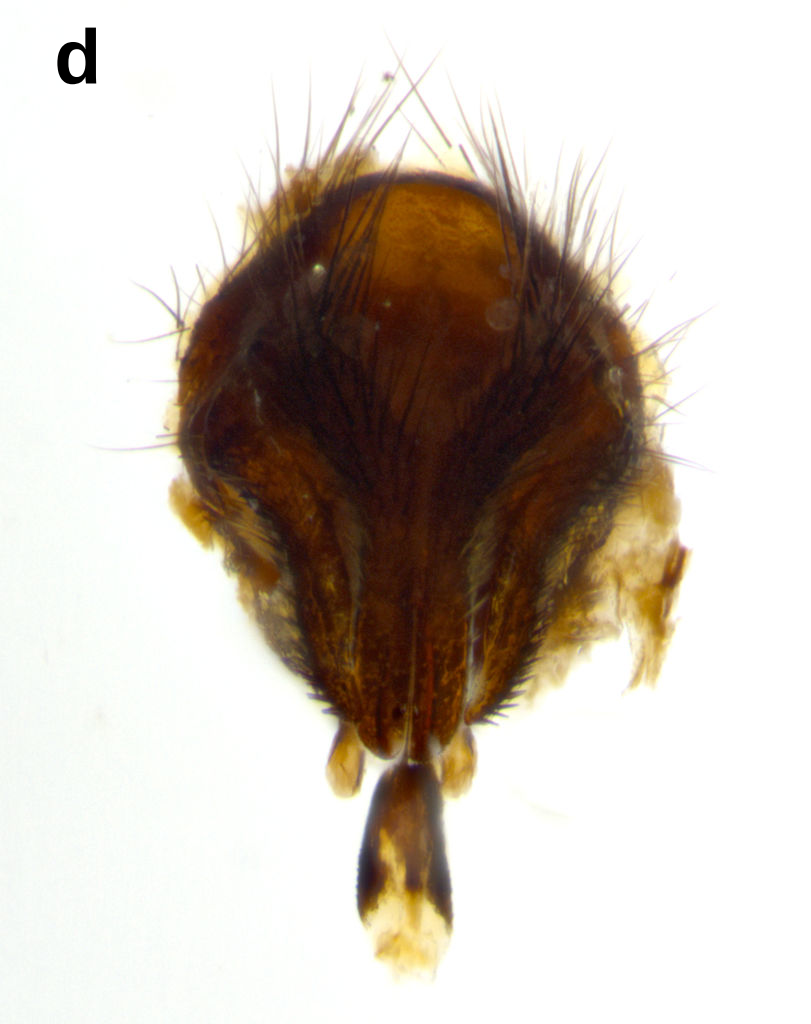
dorsal view

**Figure 7e.**
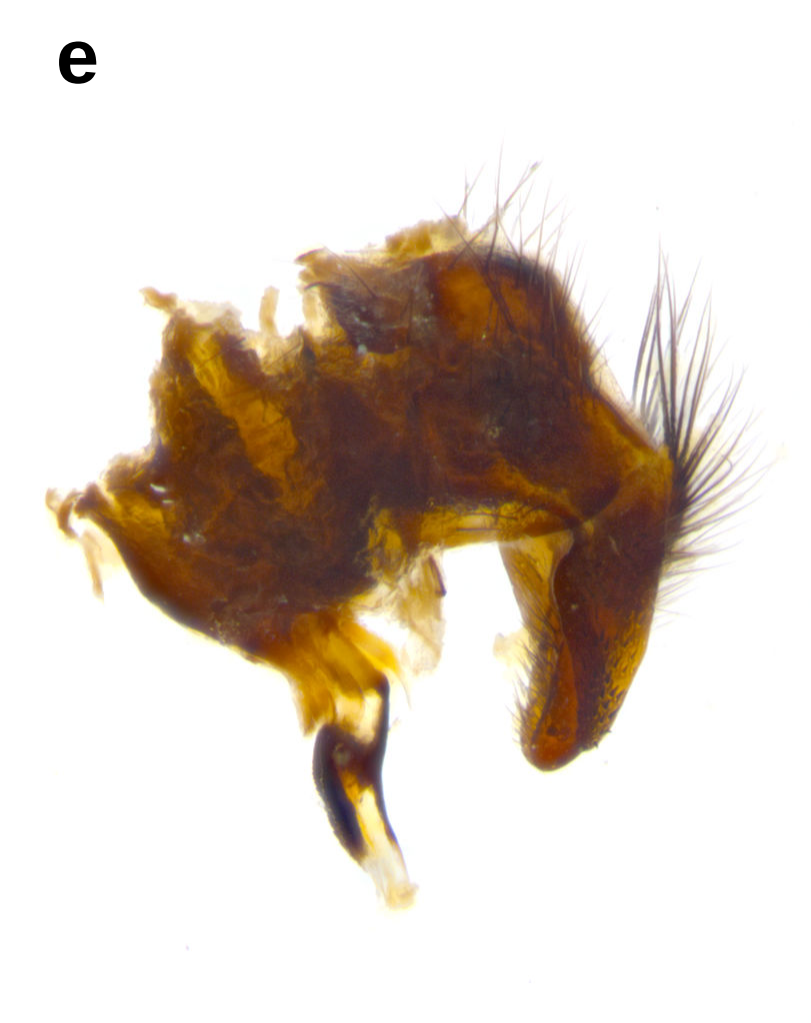
lateral view

**Figure 7f.**
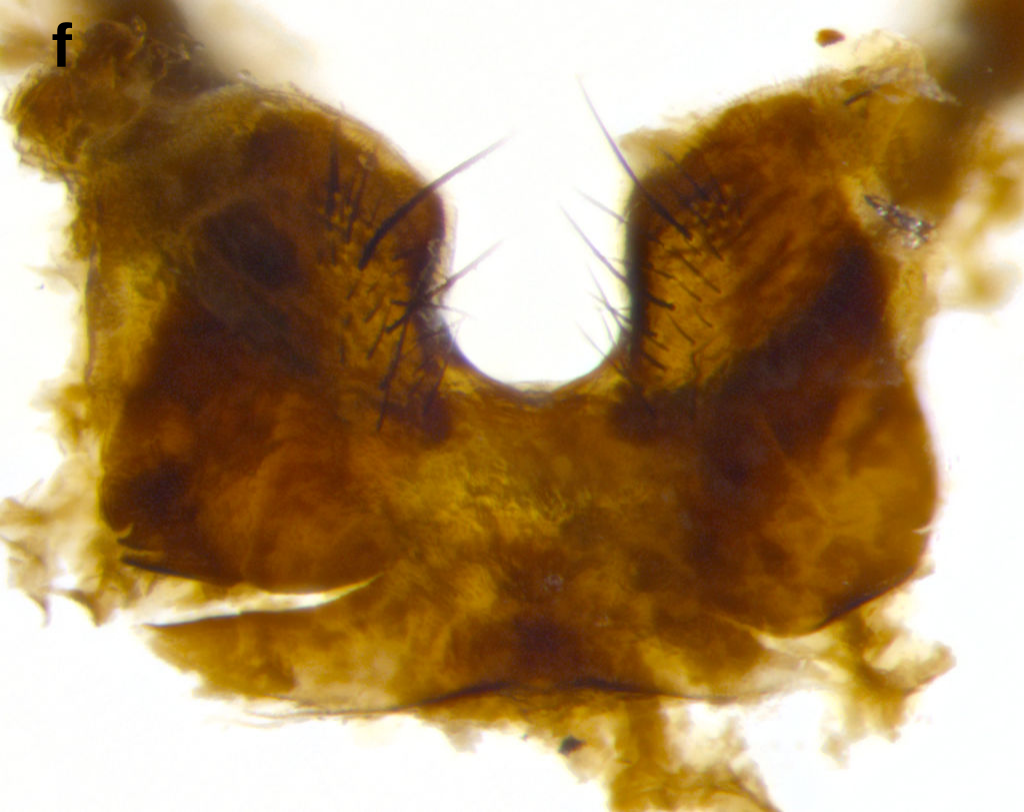
sternite 5, ventral view

**Figure 8a.**
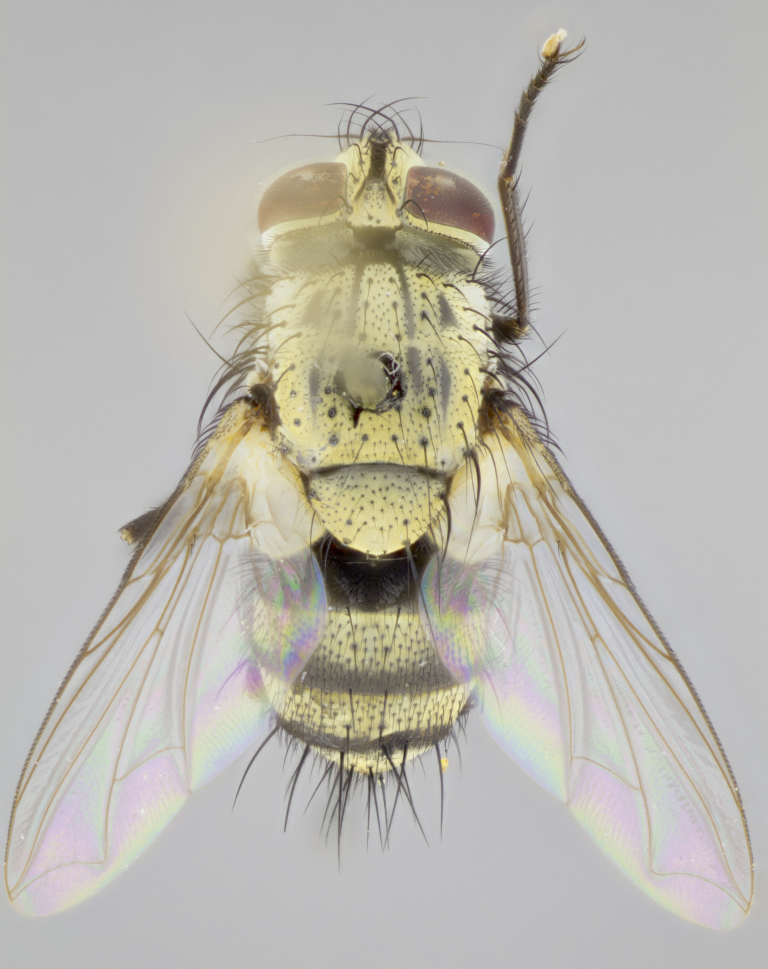
dorsal view

**Figure 8b.**
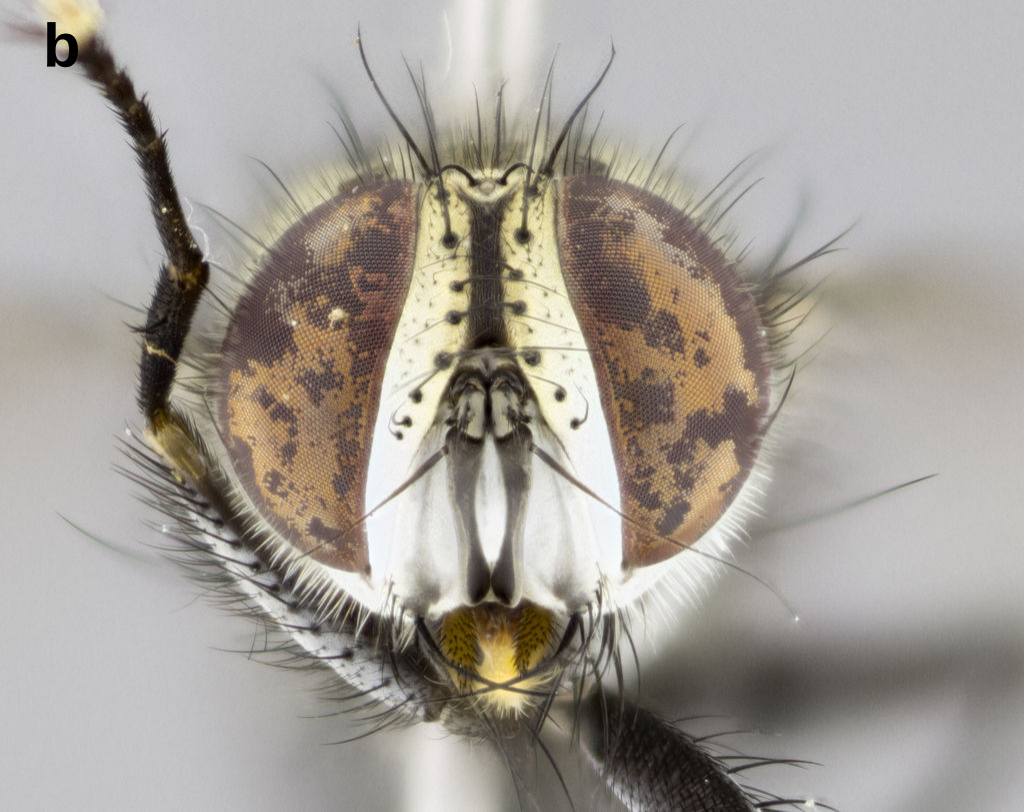
frontal view

**Figure 8c.**
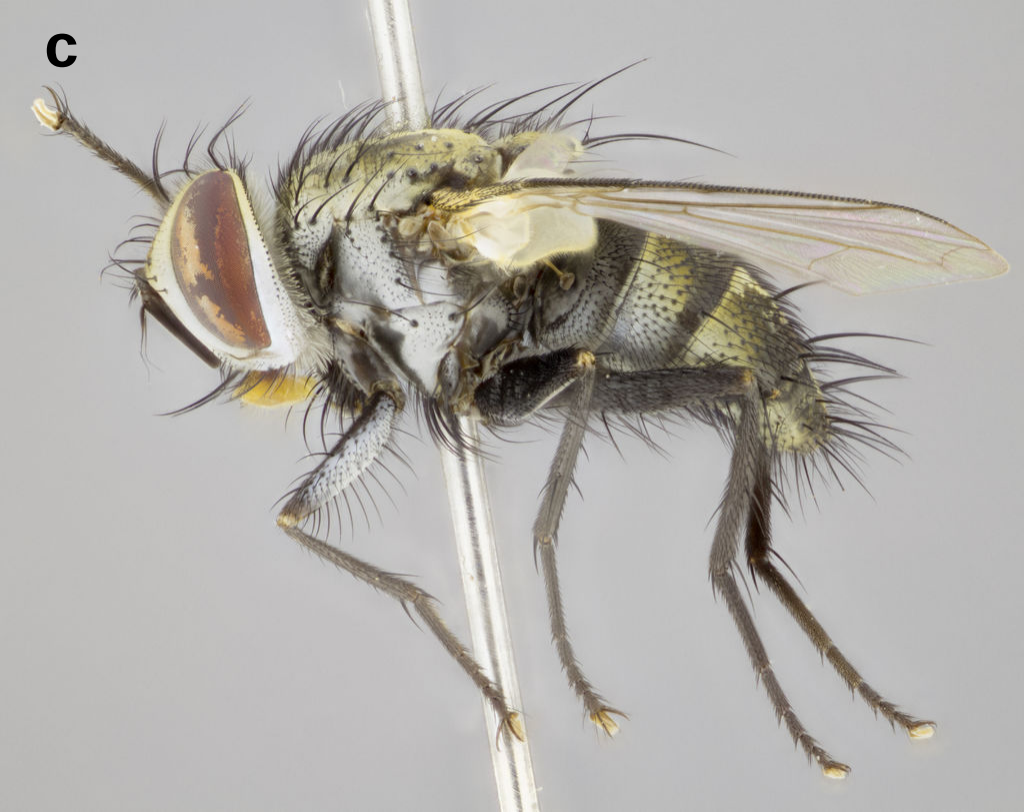
lateral view

**Figure 8d.**
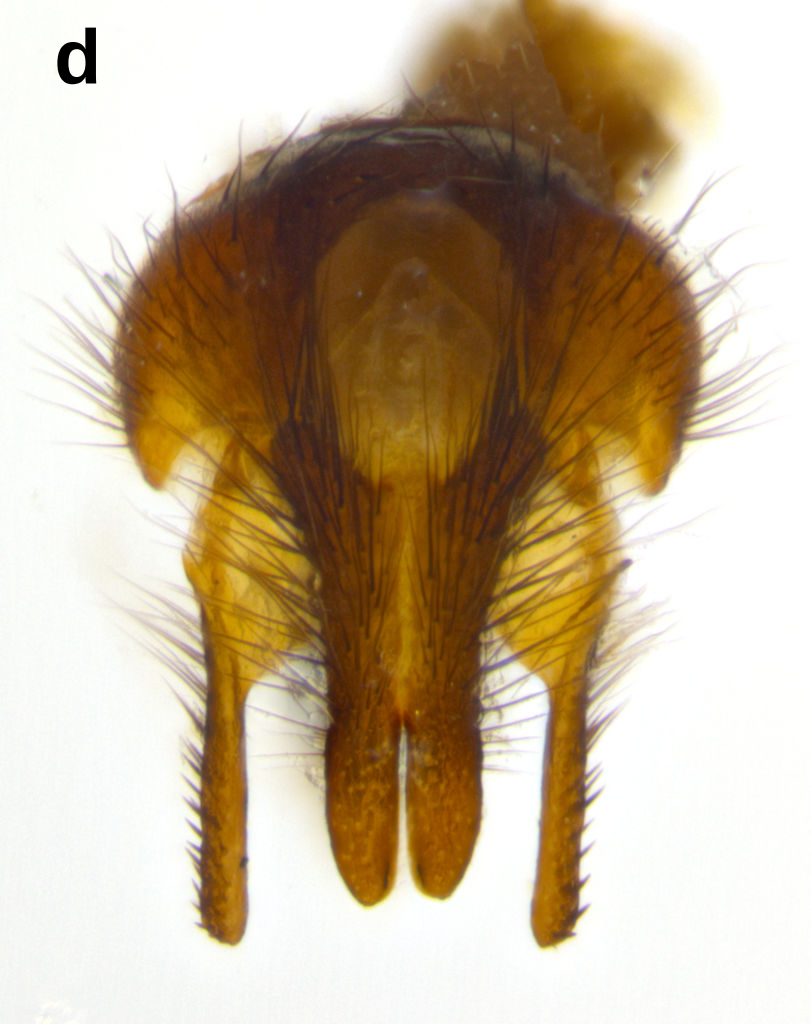
dorsal view

**Figure 8e.**
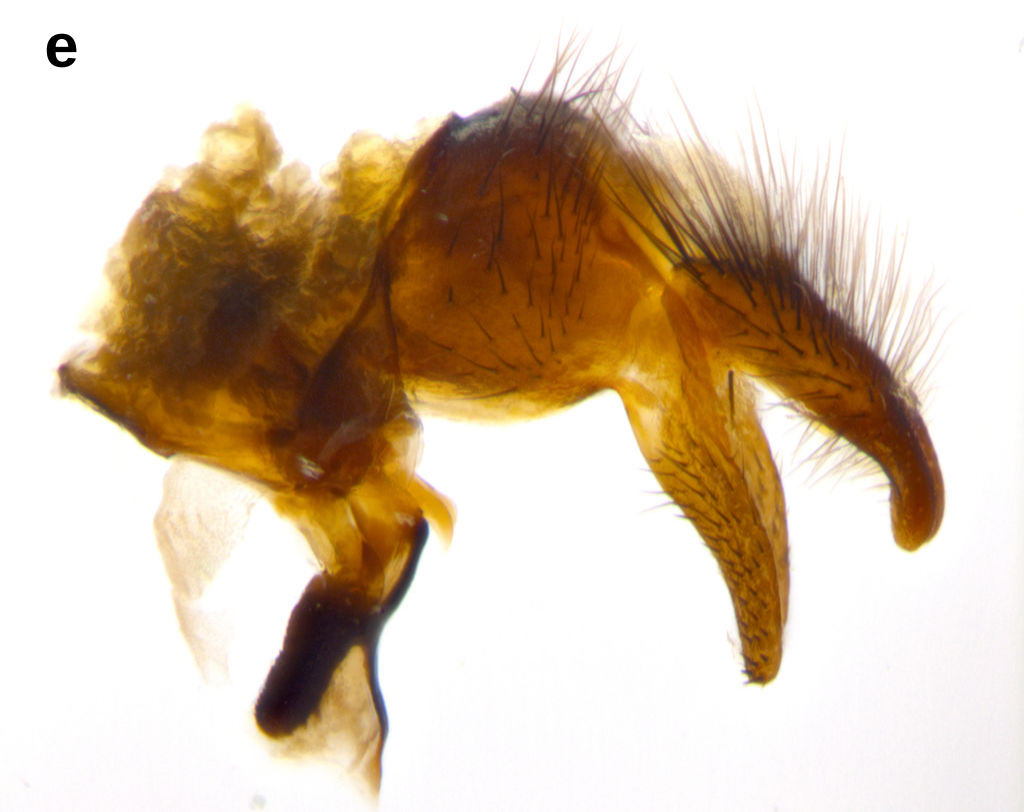
lateral view

**Figure 8f.**
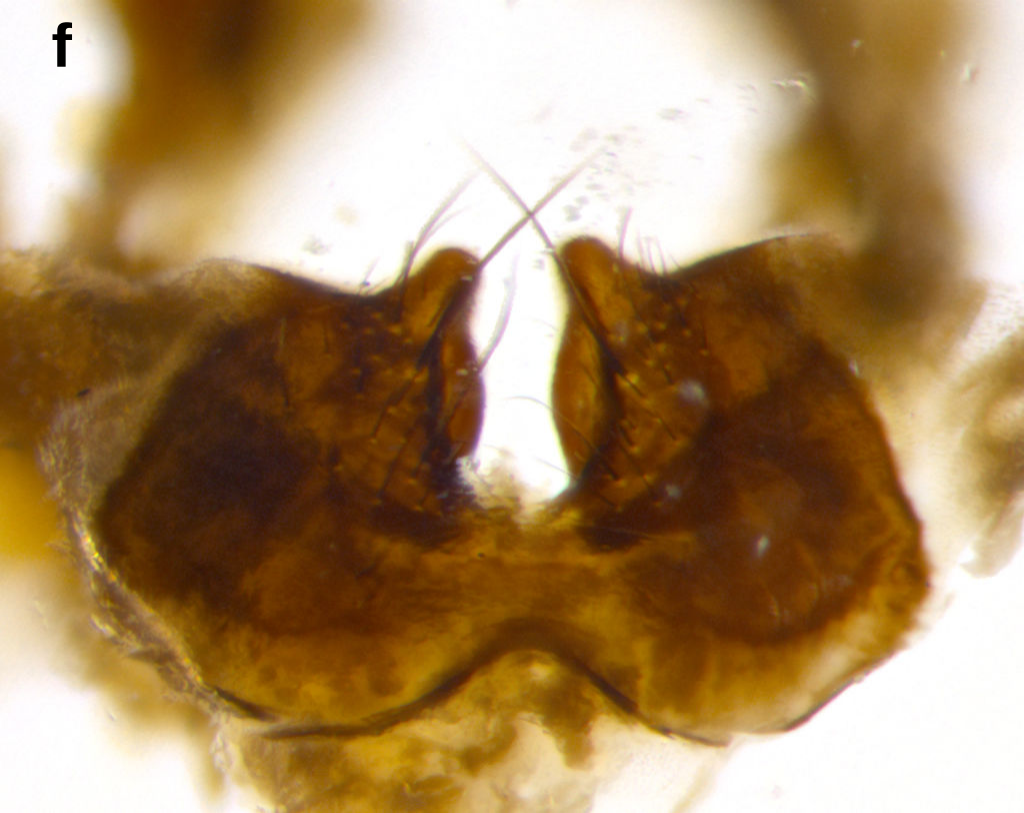
sternite 5, ventral view

**Figure 9a.**
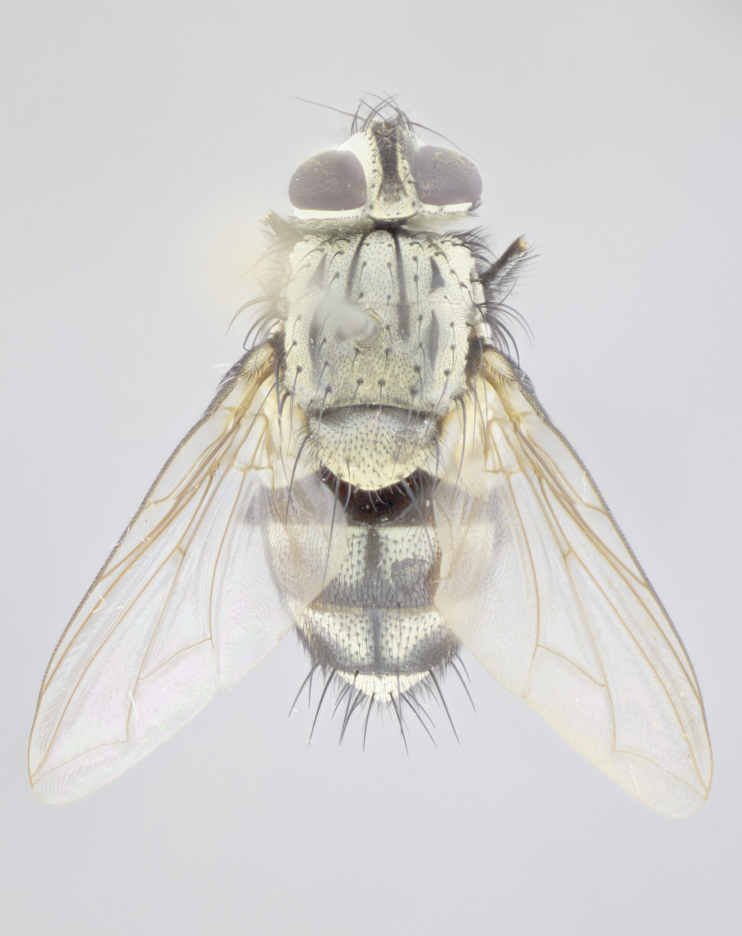
dorsal view

**Figure 9b.**
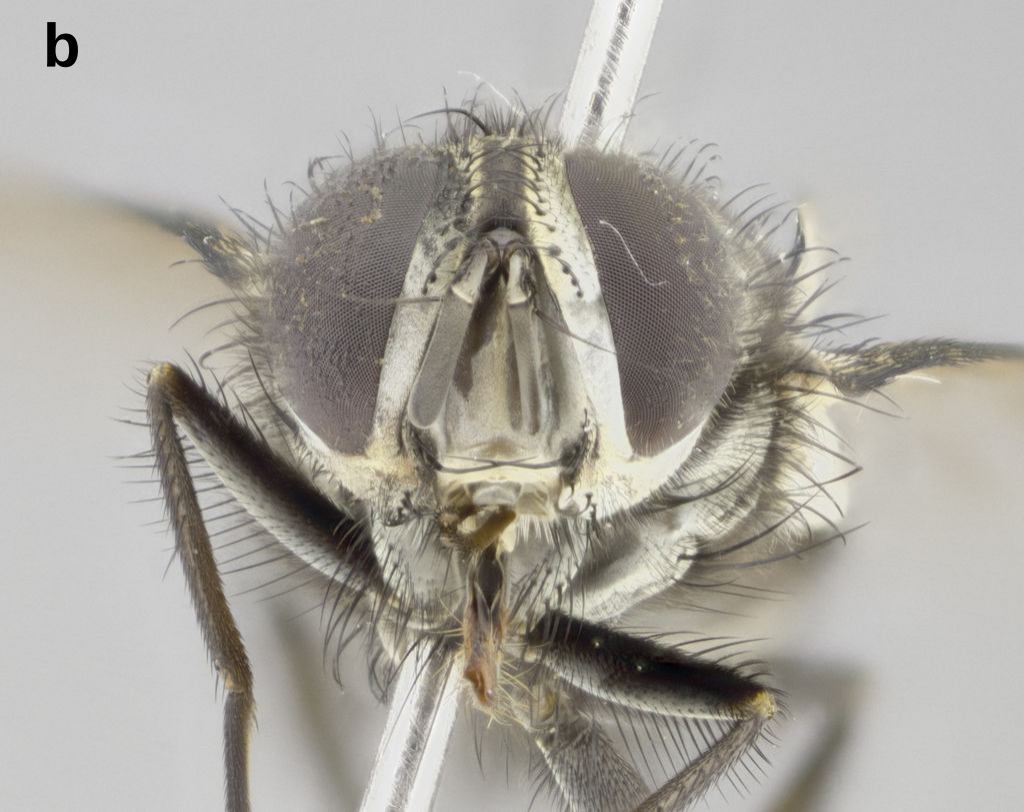
frontal view

**Figure 9c.**
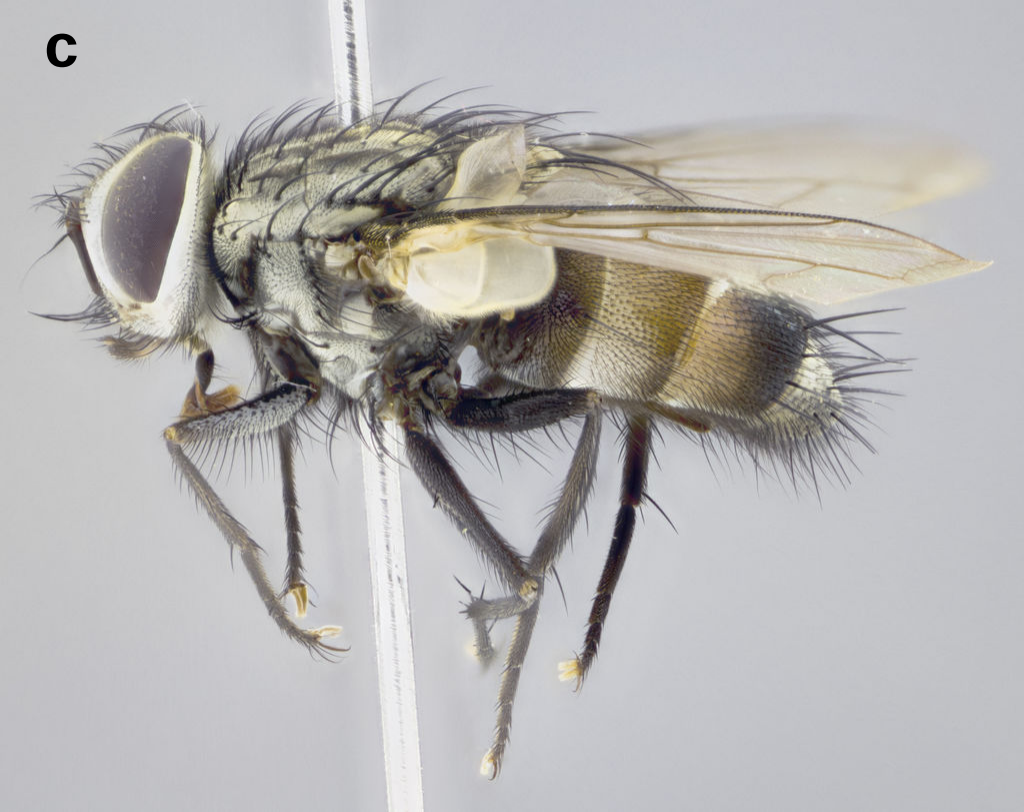
lateral view

**Figure 9d.**
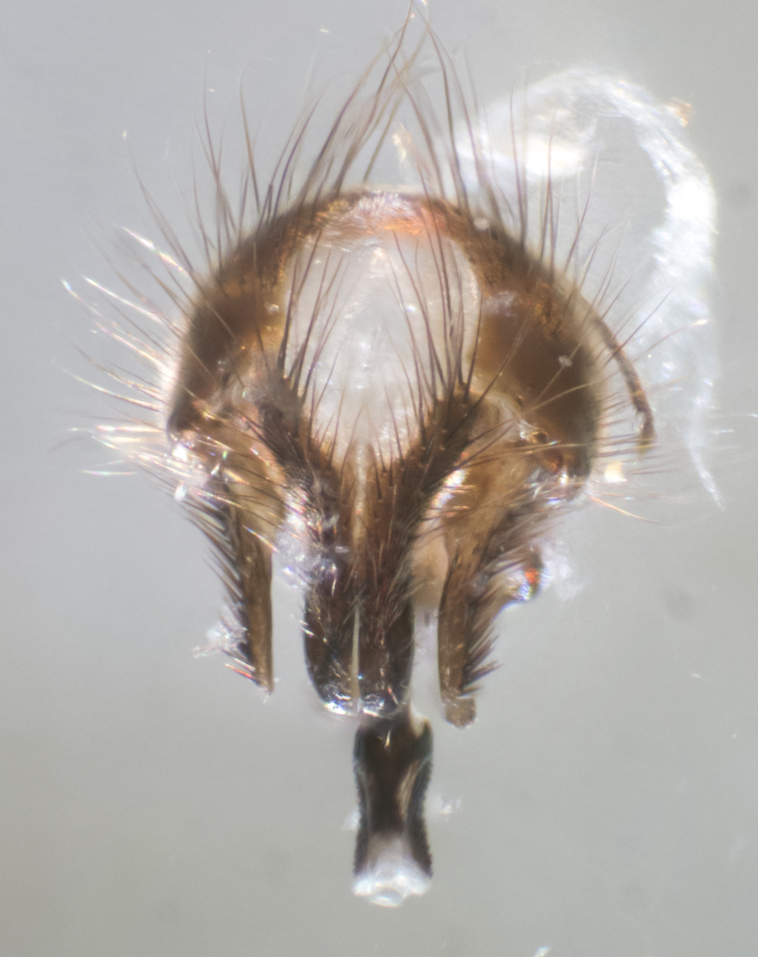
dorsal view

**Figure 9e.**
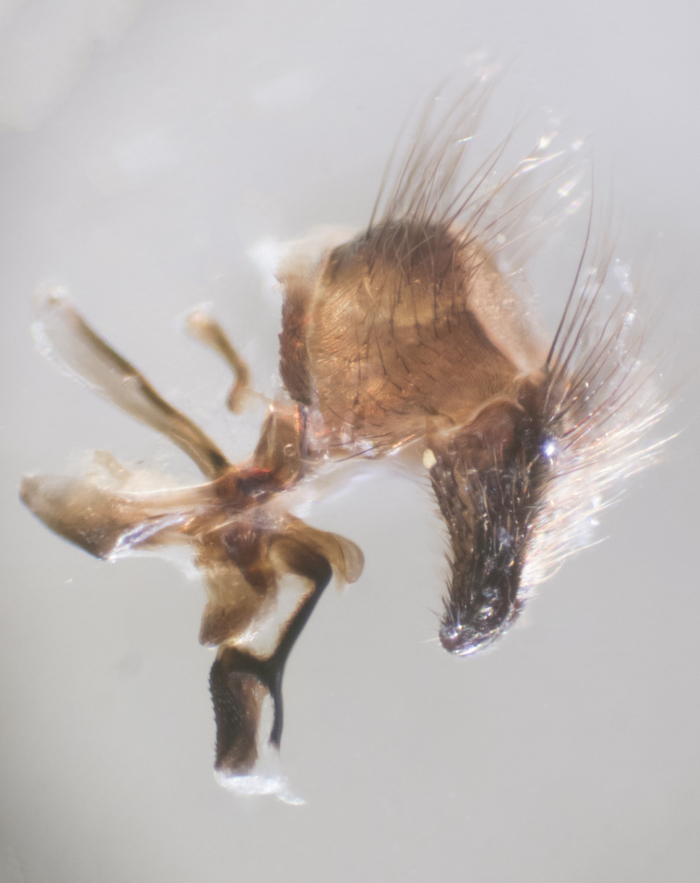
lateral view

**Figure 9f.**
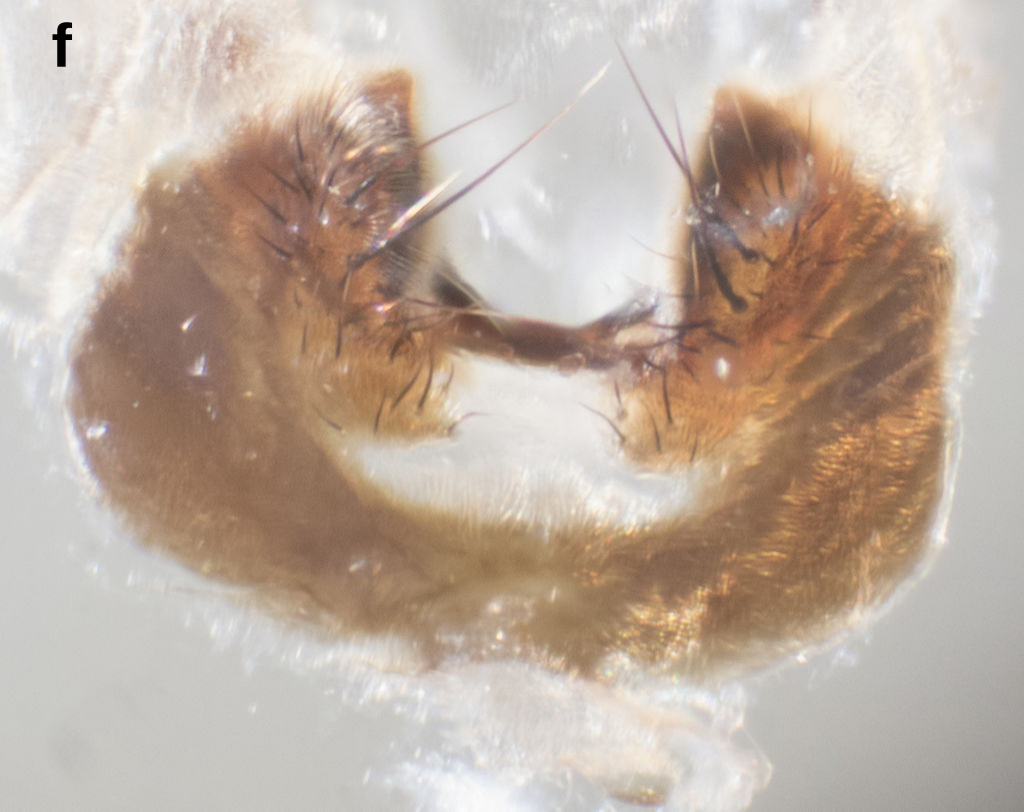
sternite 5, ventral view

**Figure 10a.**
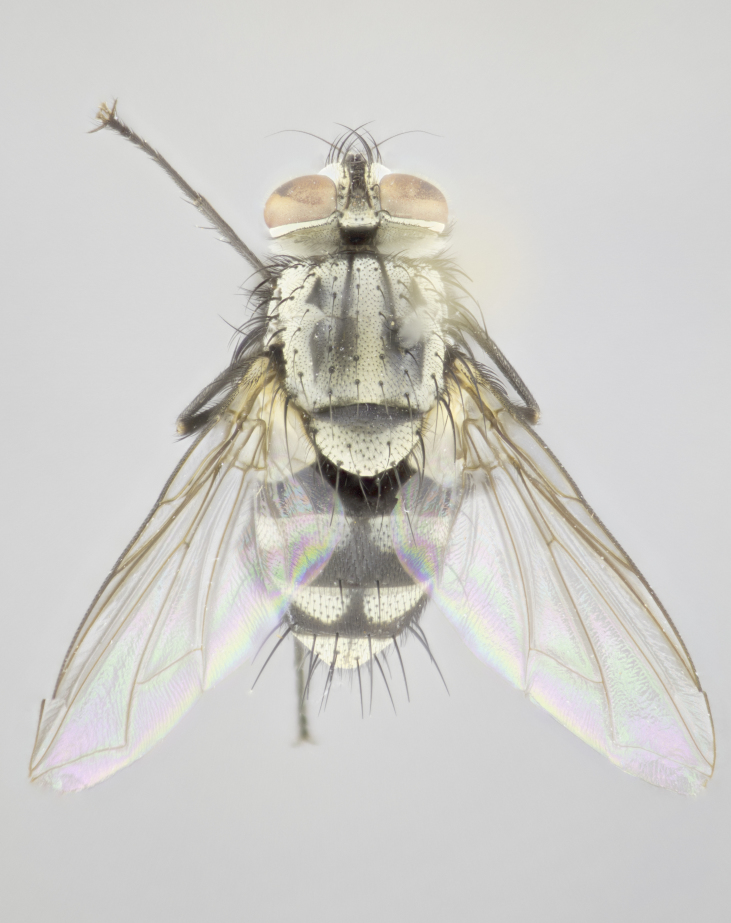
dorsal view

**Figure 10b.**
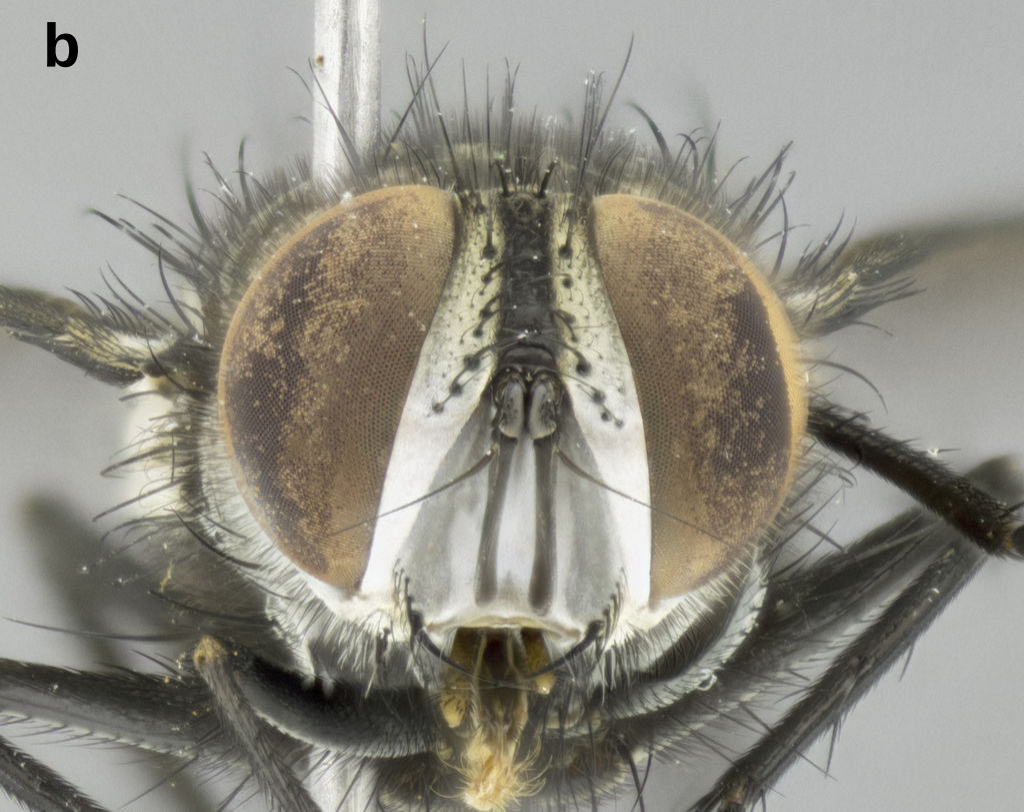
frontal view

**Figure 10c.**
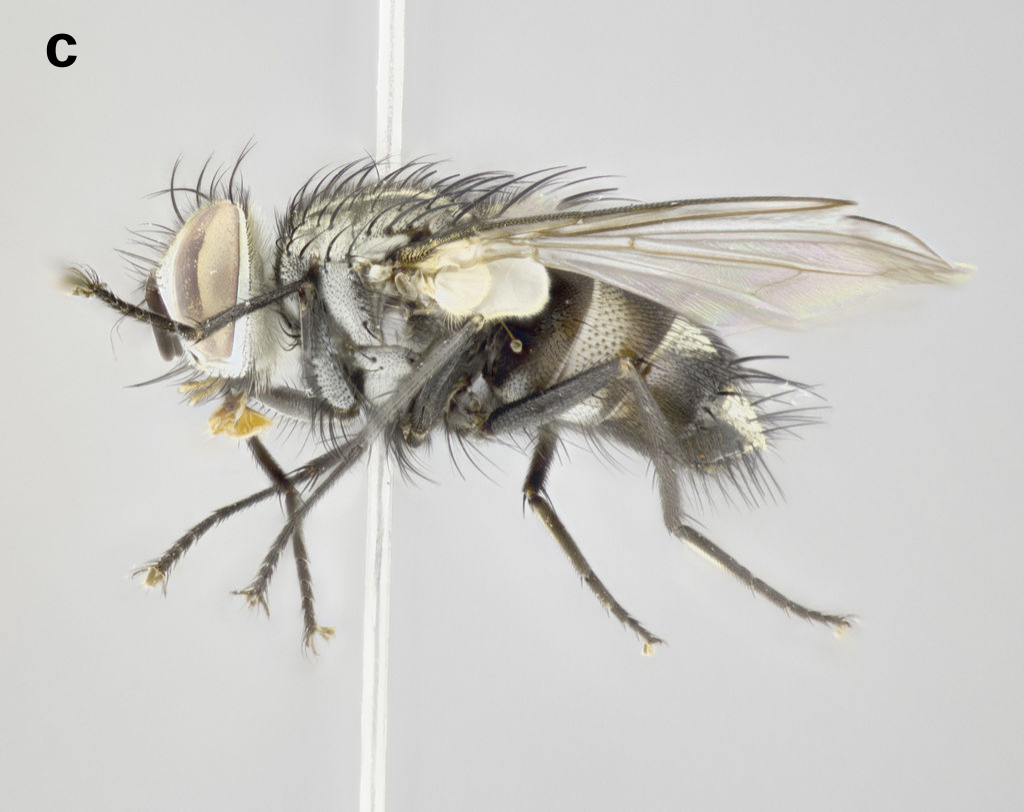
lateral view

**Figure 10d.**
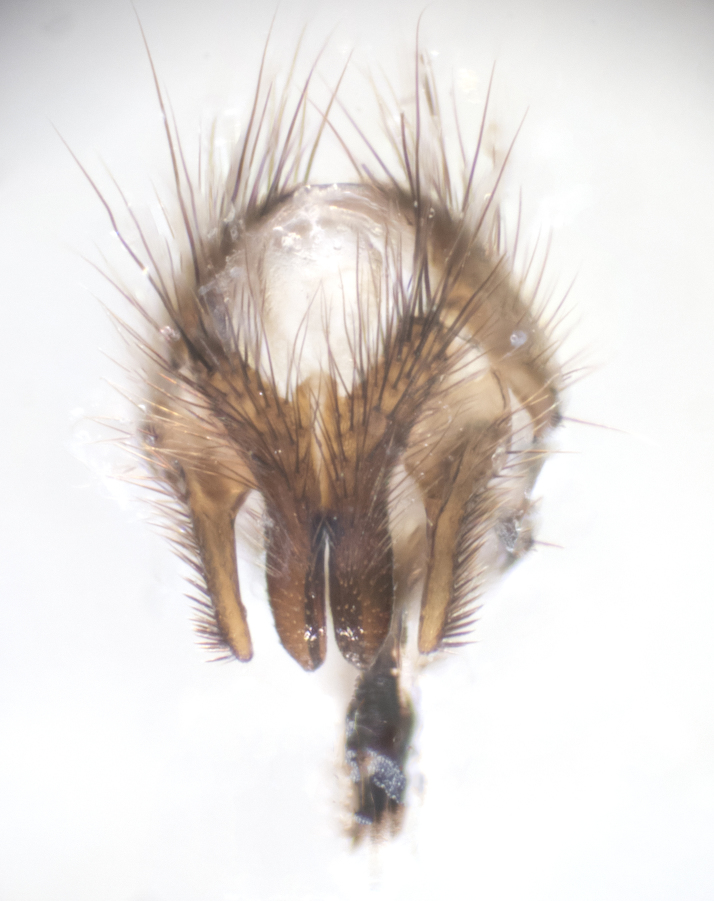
dorsal view

**Figure 10e.**
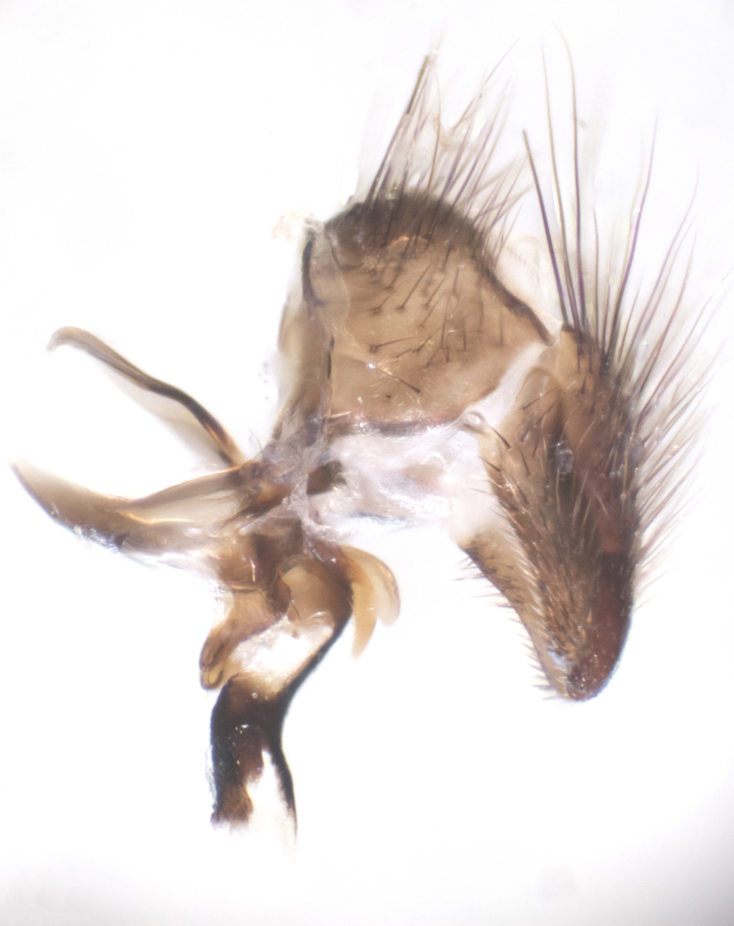
lateral view

**Figure 10f.**
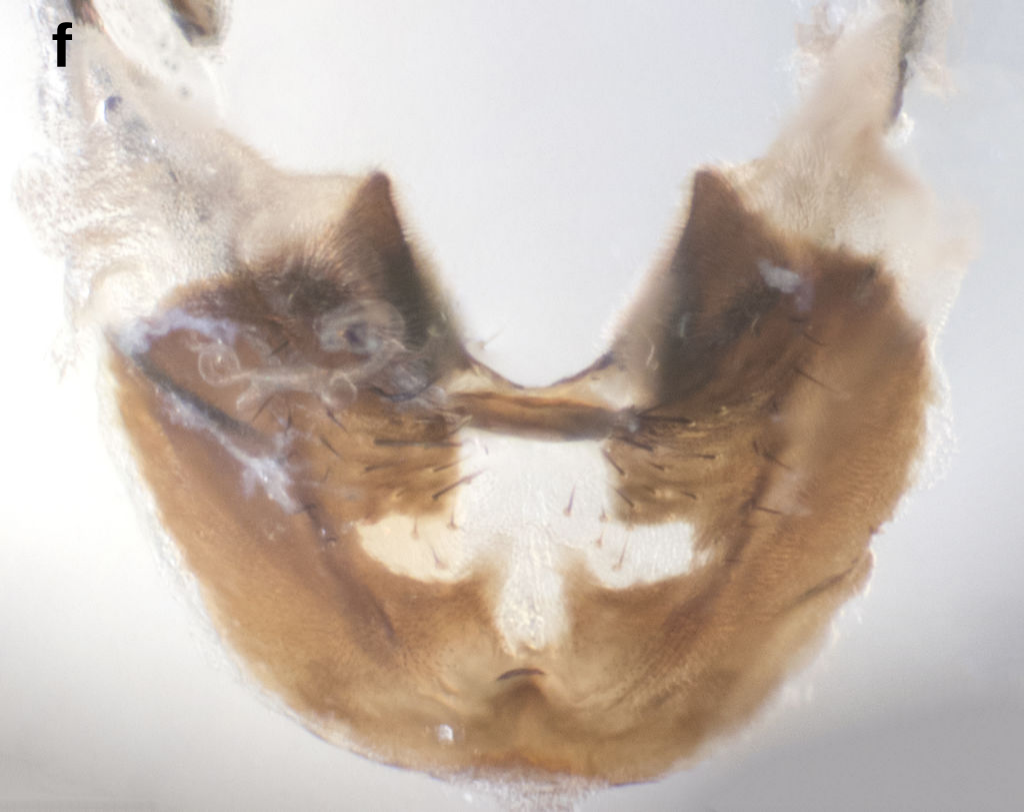
sternite 5, ventral view

**Figure 11a.**
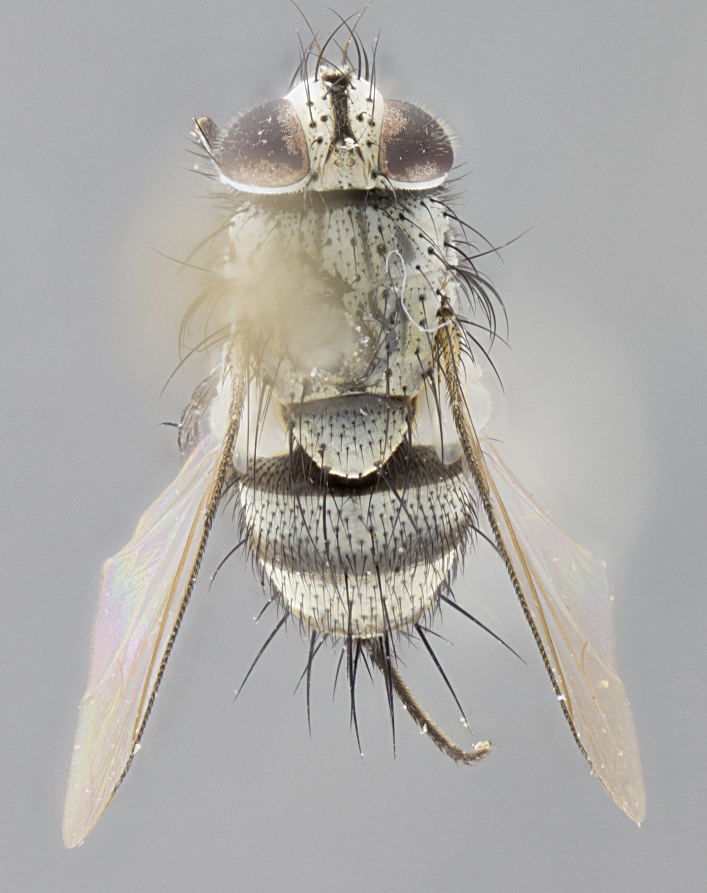
dorsal view

**Figure 11b.**
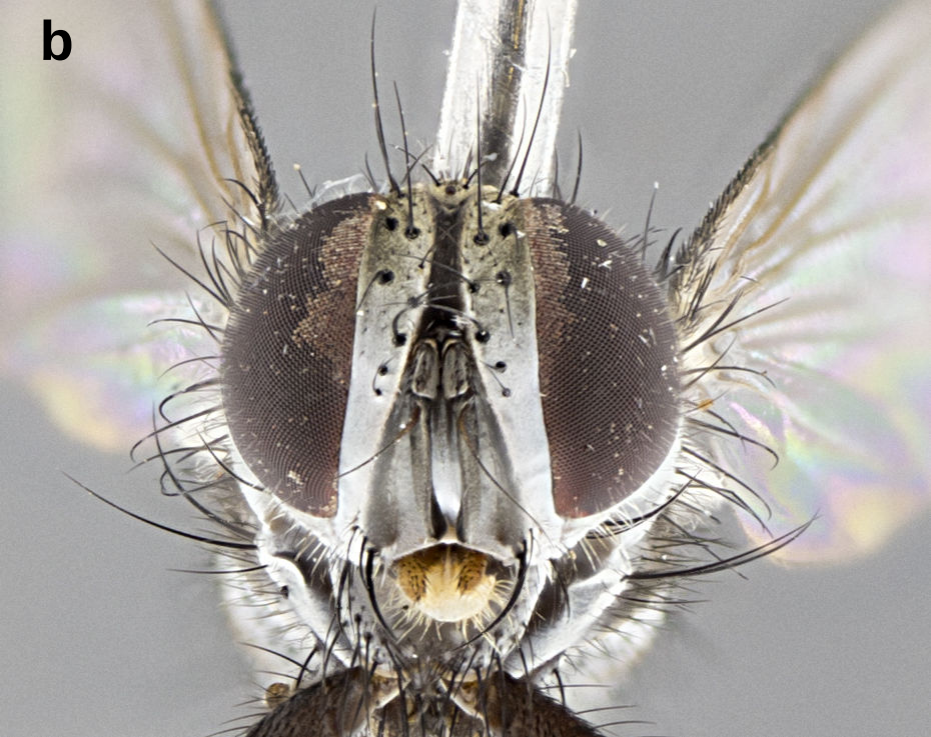
frontal view

**Figure 11c.**
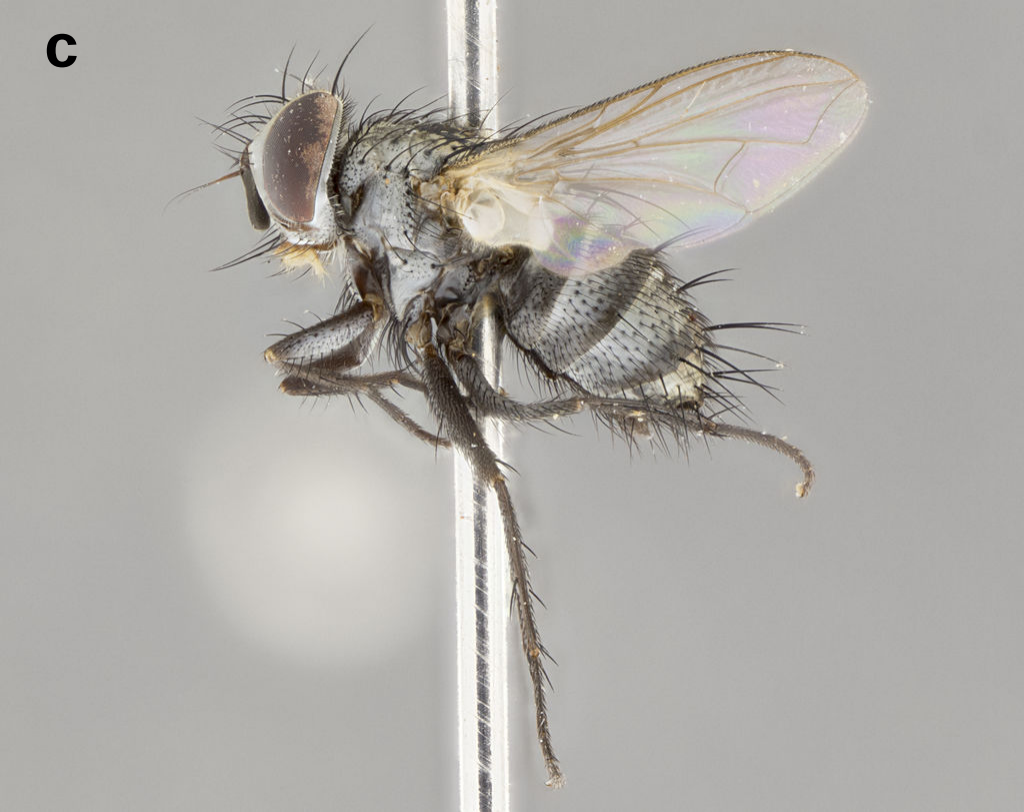
lateral view

**Figure 12a.**
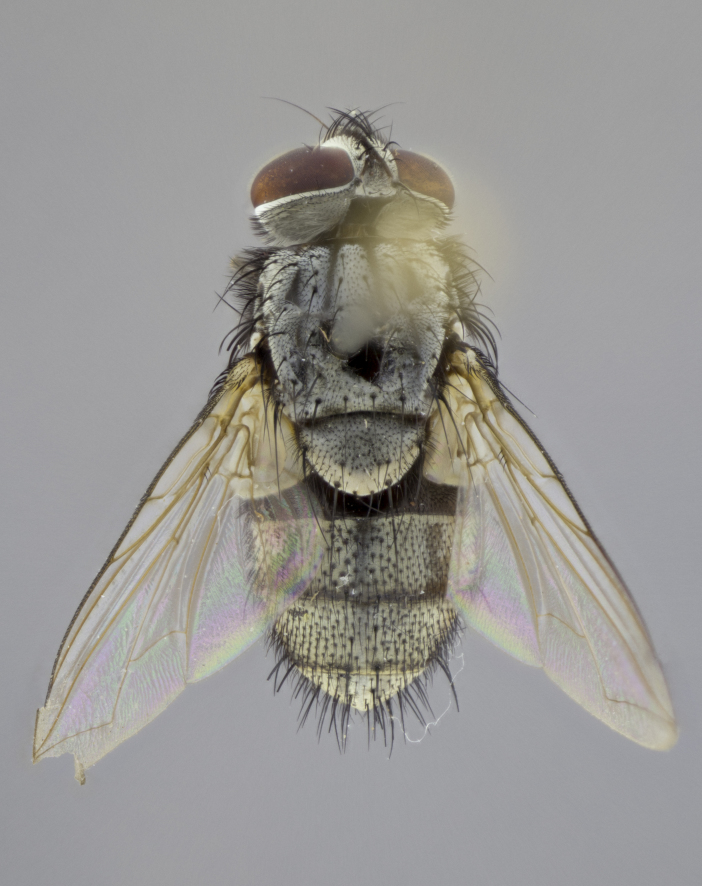
dorsal view

**Figure 12b.**
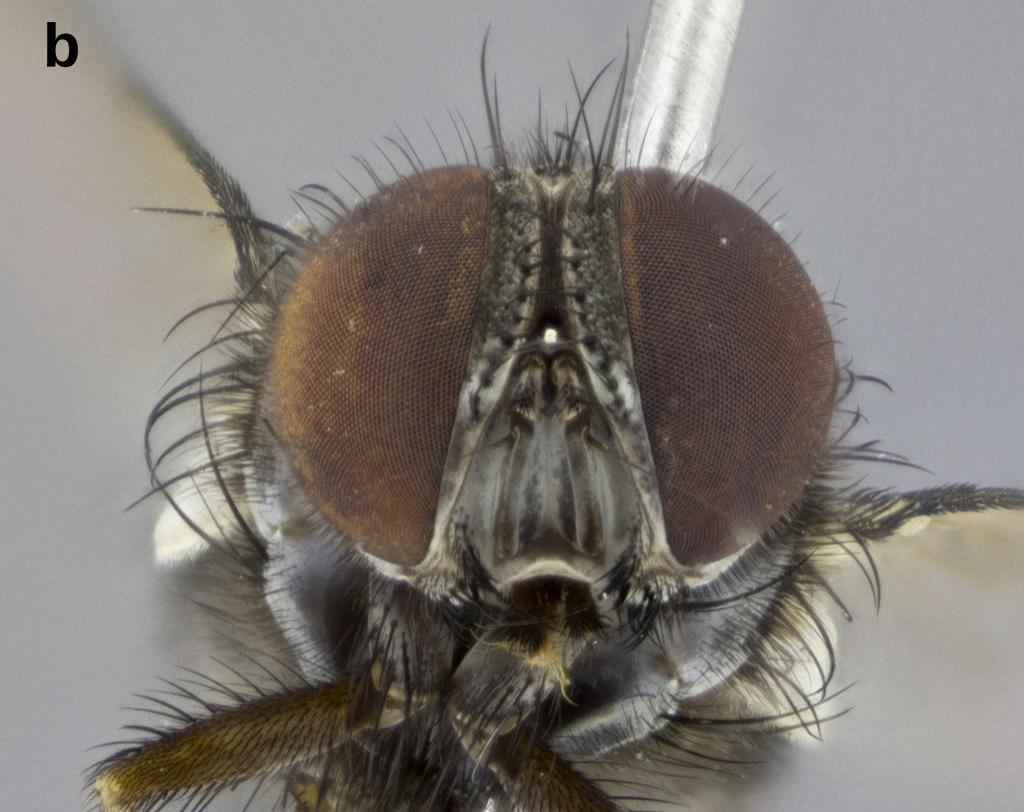
frontal view

**Figure 12c.**
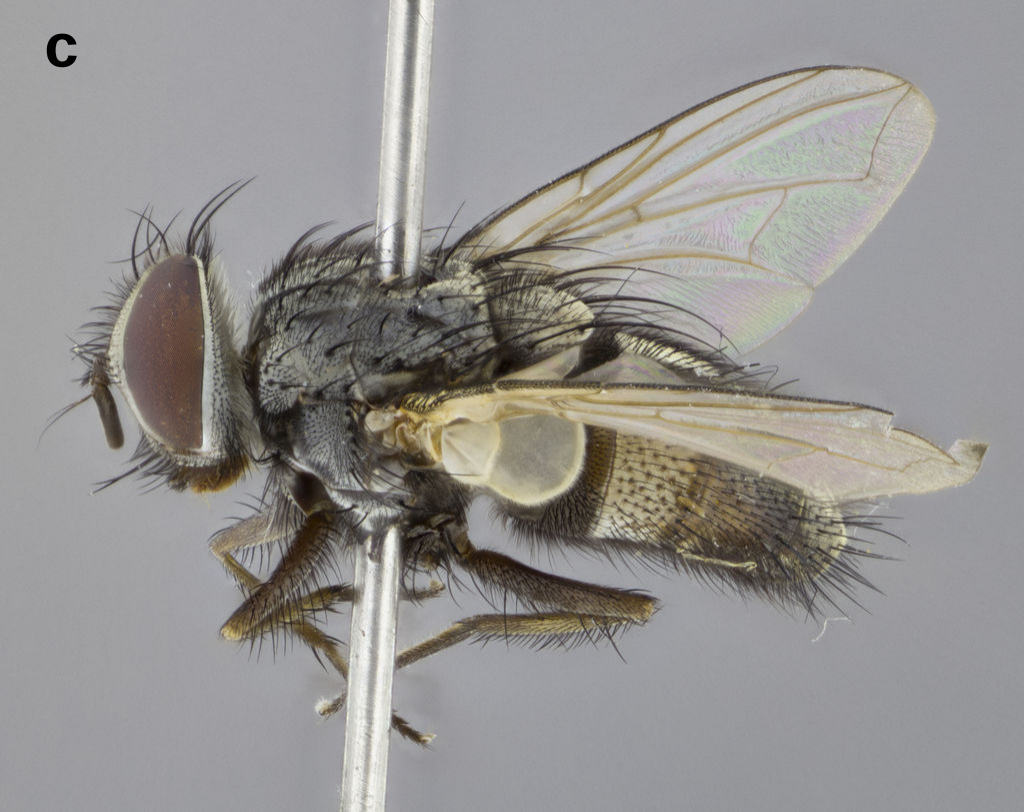
lateral view

**Figure 12d.**
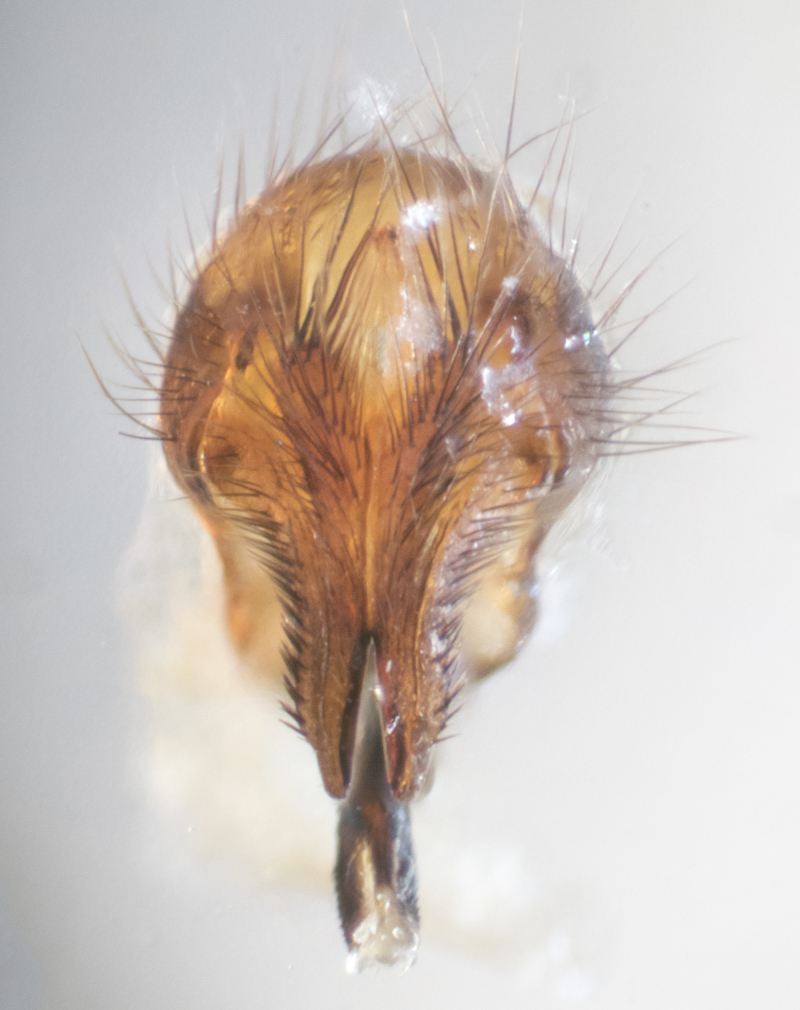
dorsal view

**Figure 12e.**
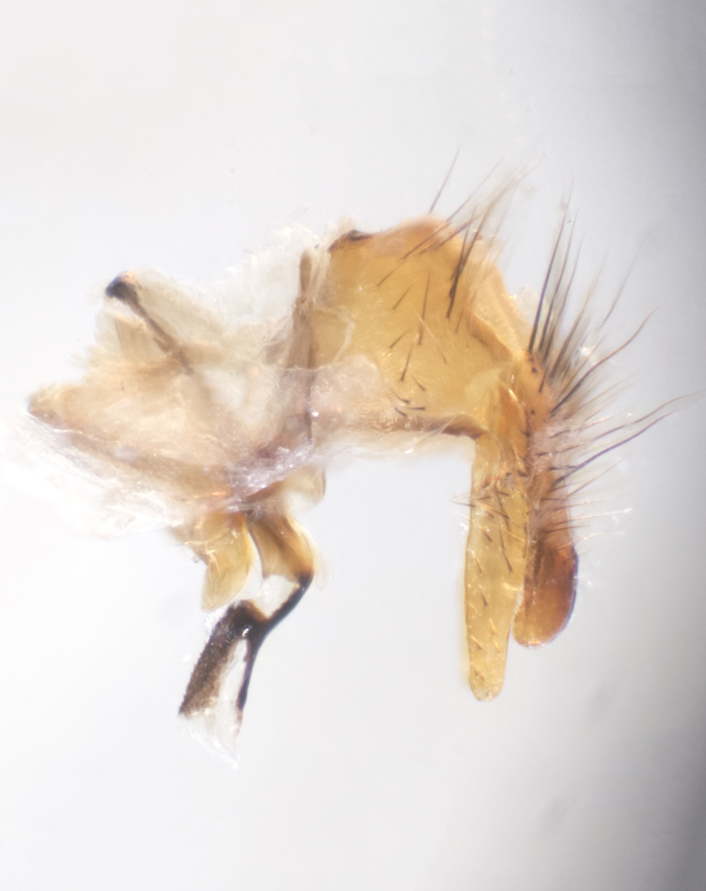
lateral view

**Figure 12f.**
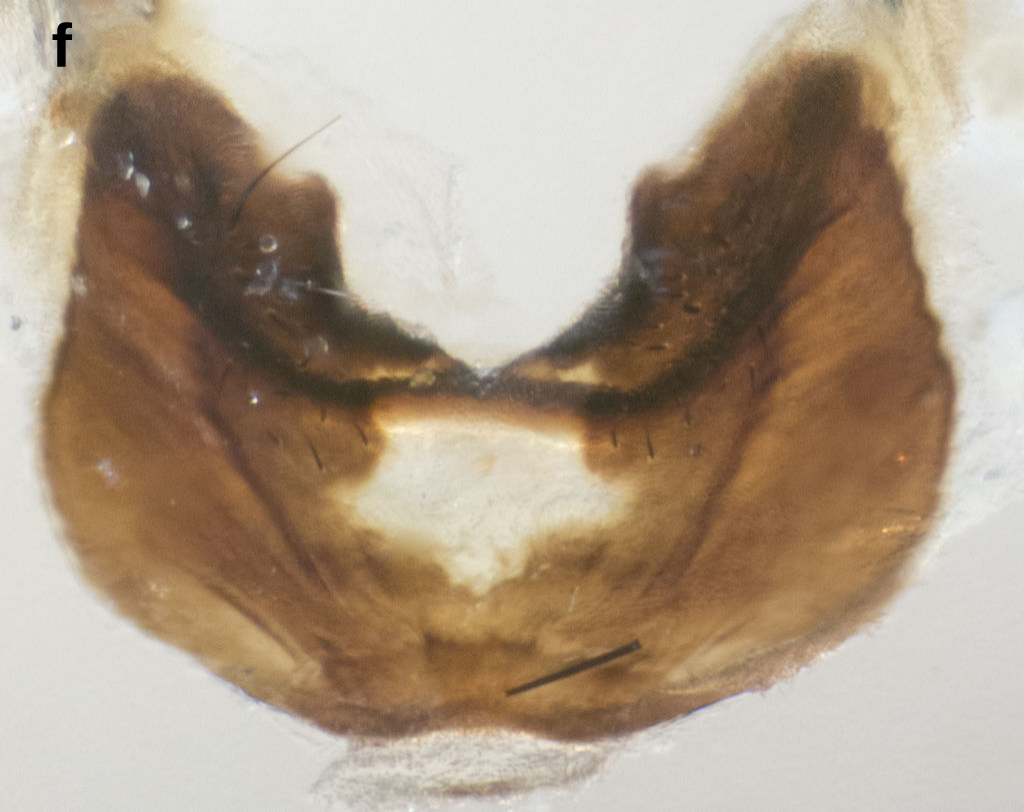
sternite 5, ventral view

**Figure 13a.**
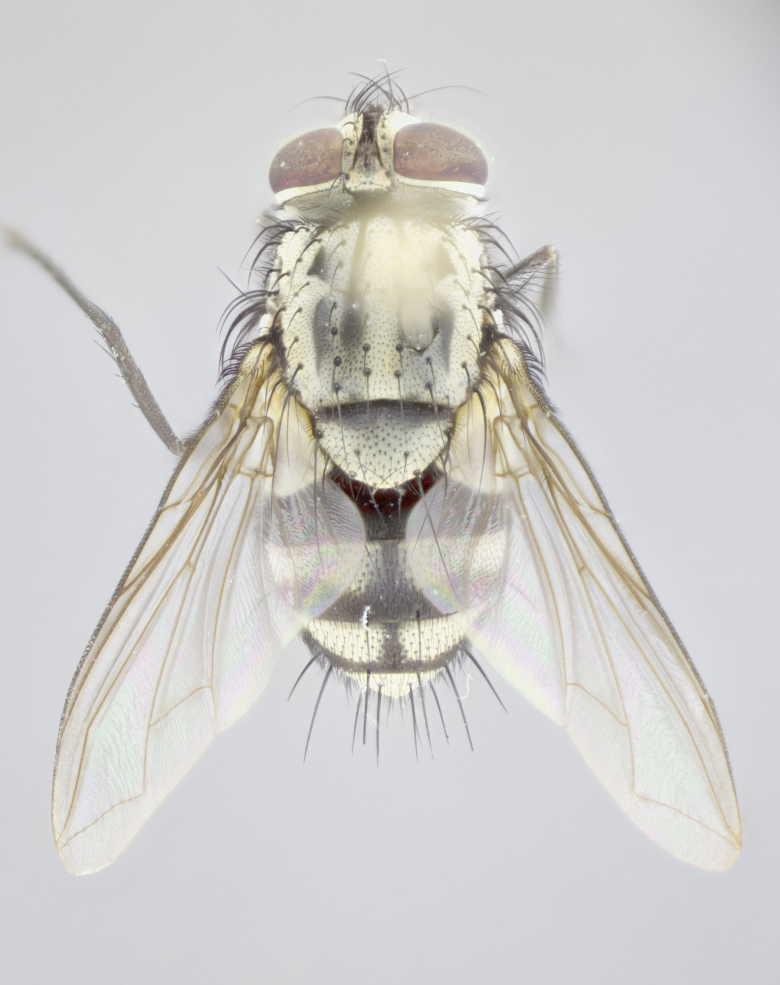
dorsal view

**Figure 13b.**
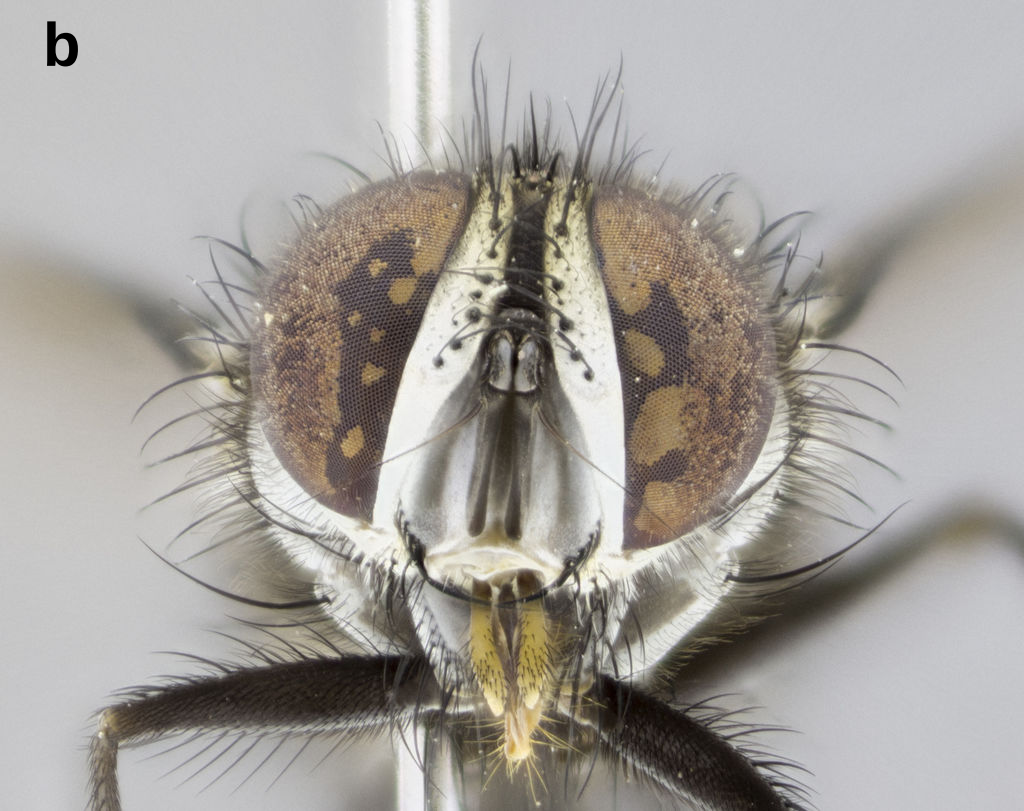
frontal view

**Figure 13c.**
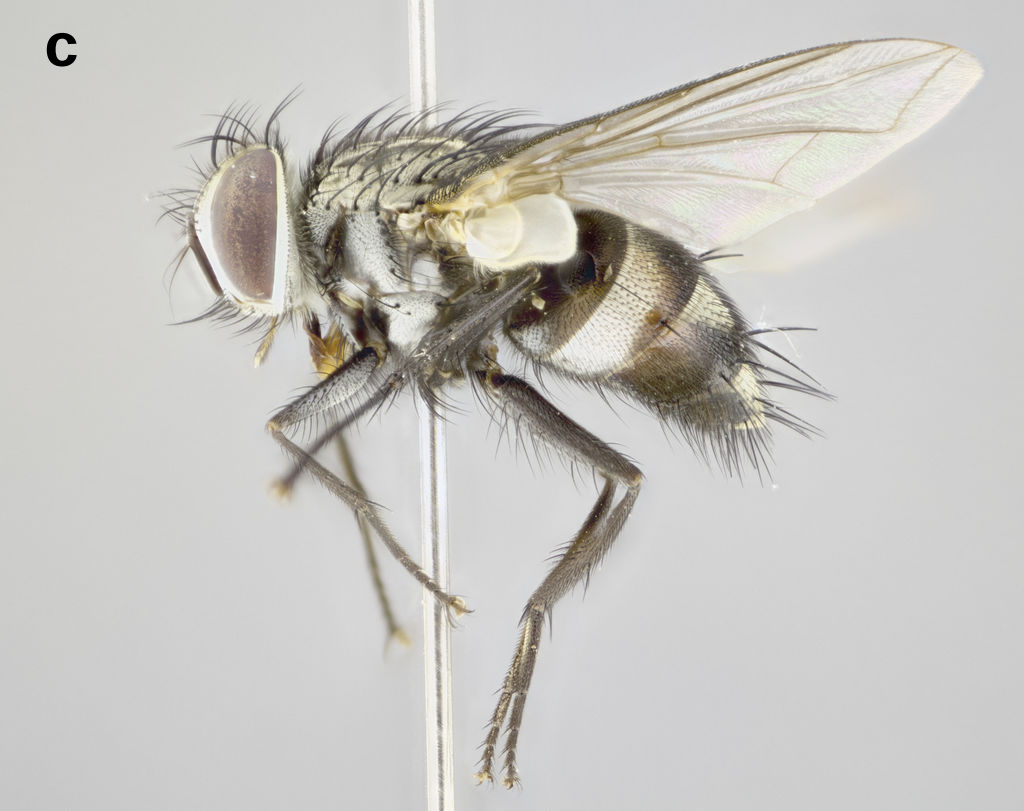
lateral view

**Figure 13d.**
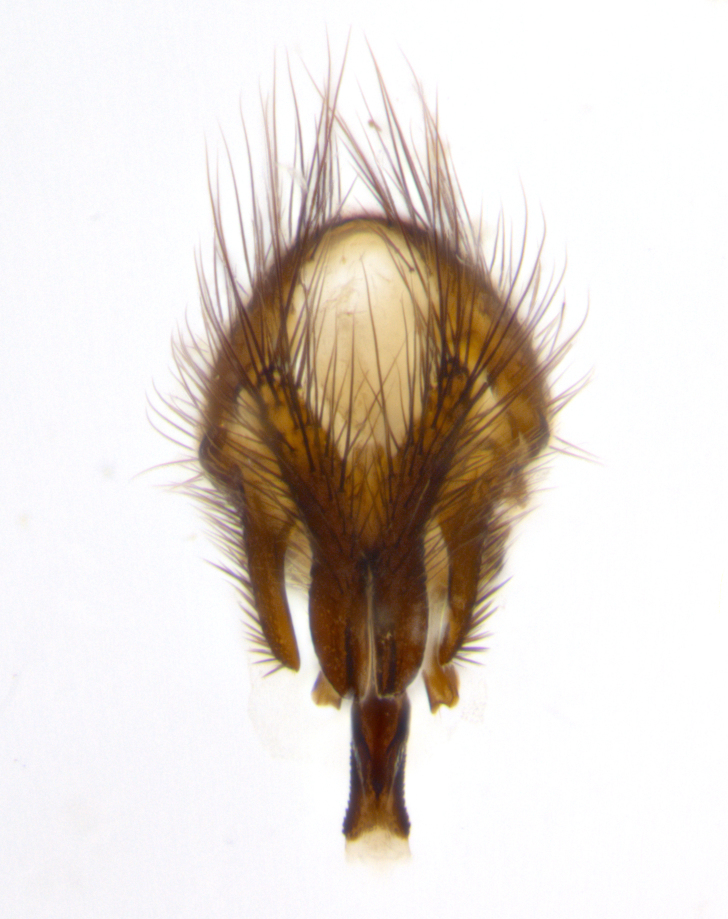
dorsal view

**Figure 13e.**
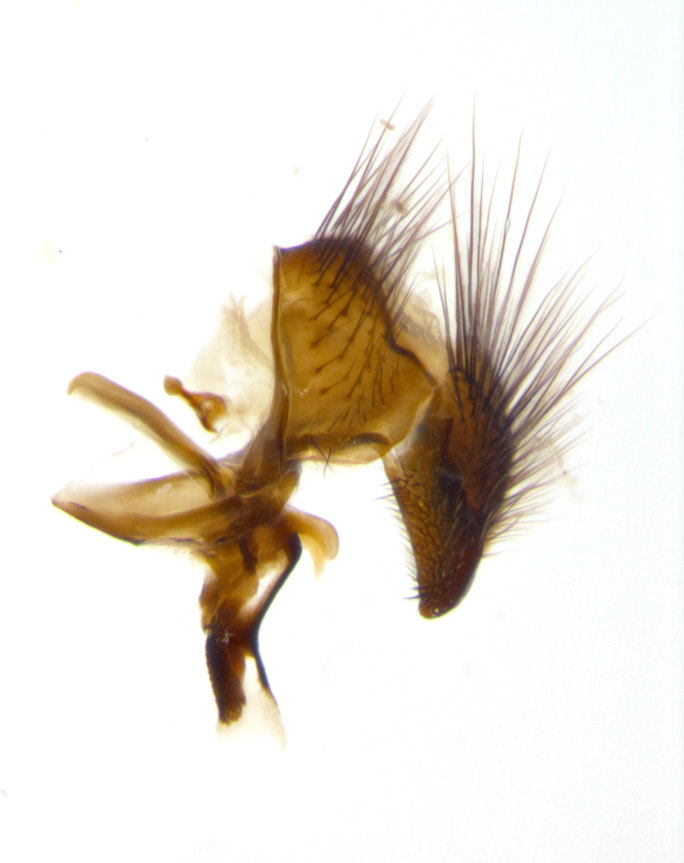
lateral view

**Figure 13f.**
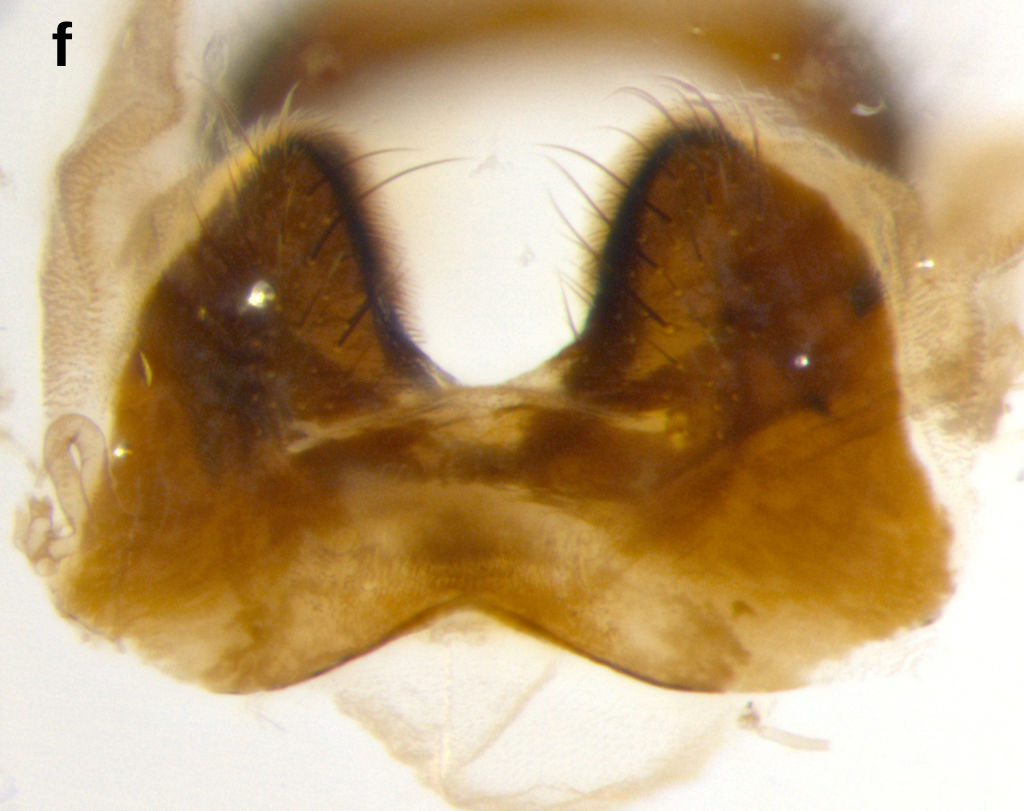
sternite 5, ventral view

**Figure 14a.**
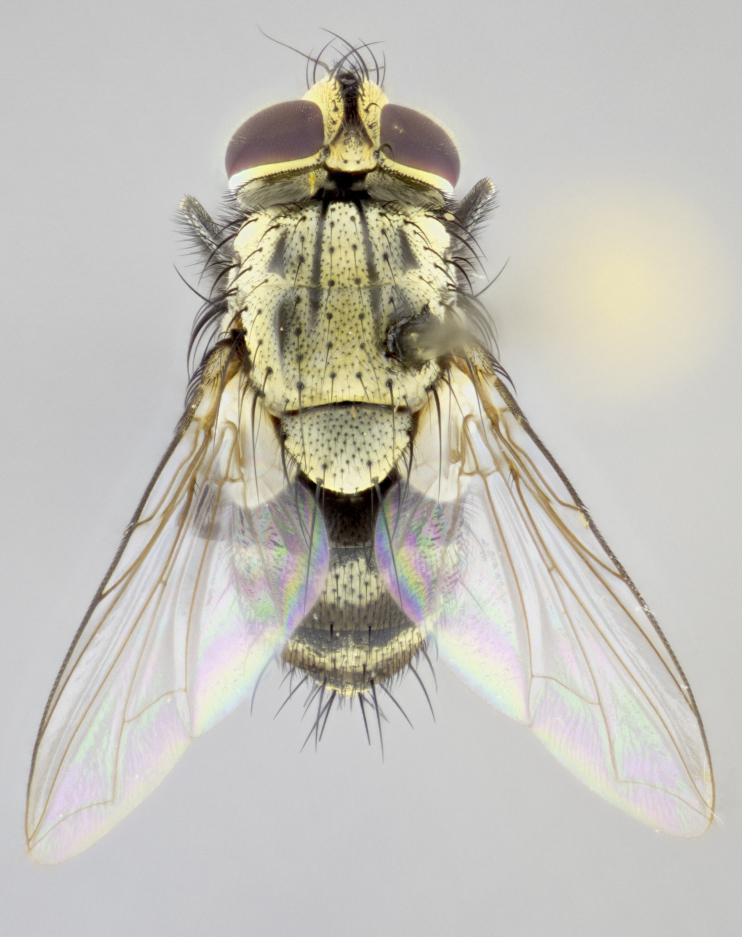
dorsal view

**Figure 14b.**
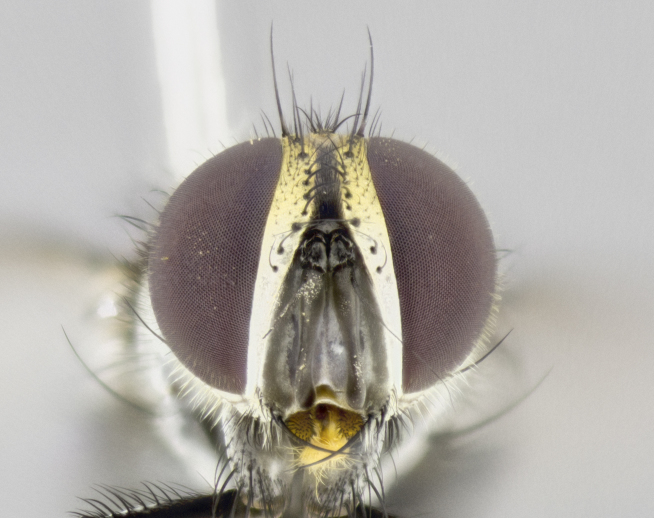
frontal view

**Figure 14c.**
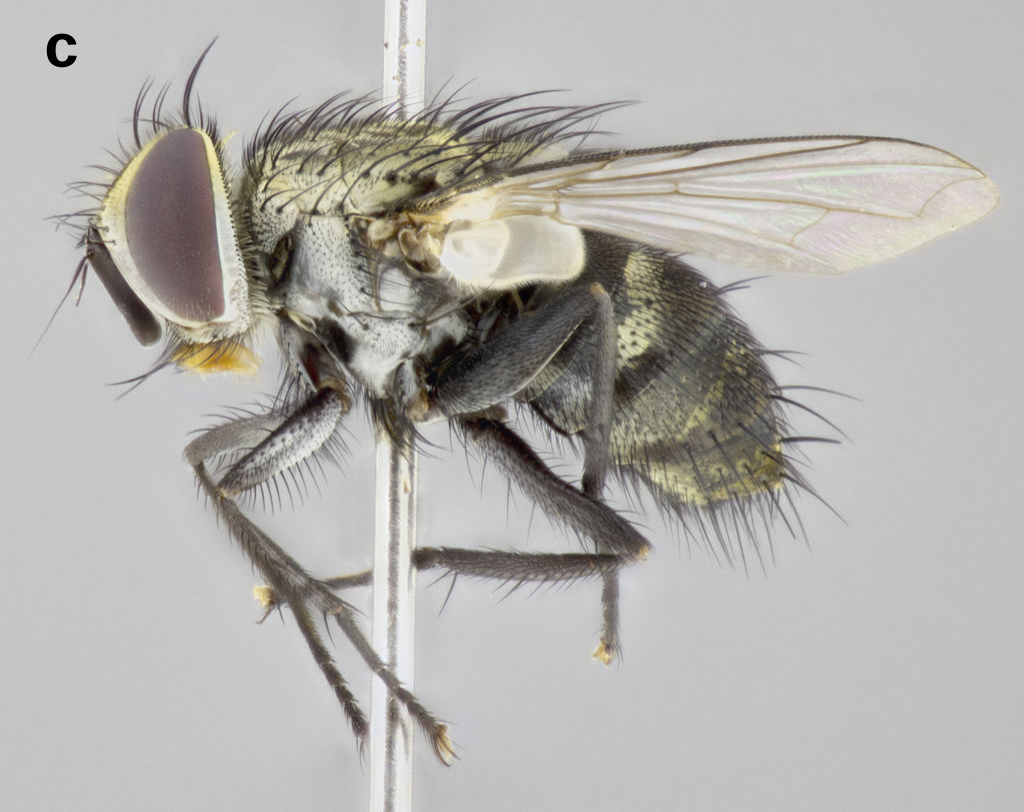
lateral view

**Figure 14d.**
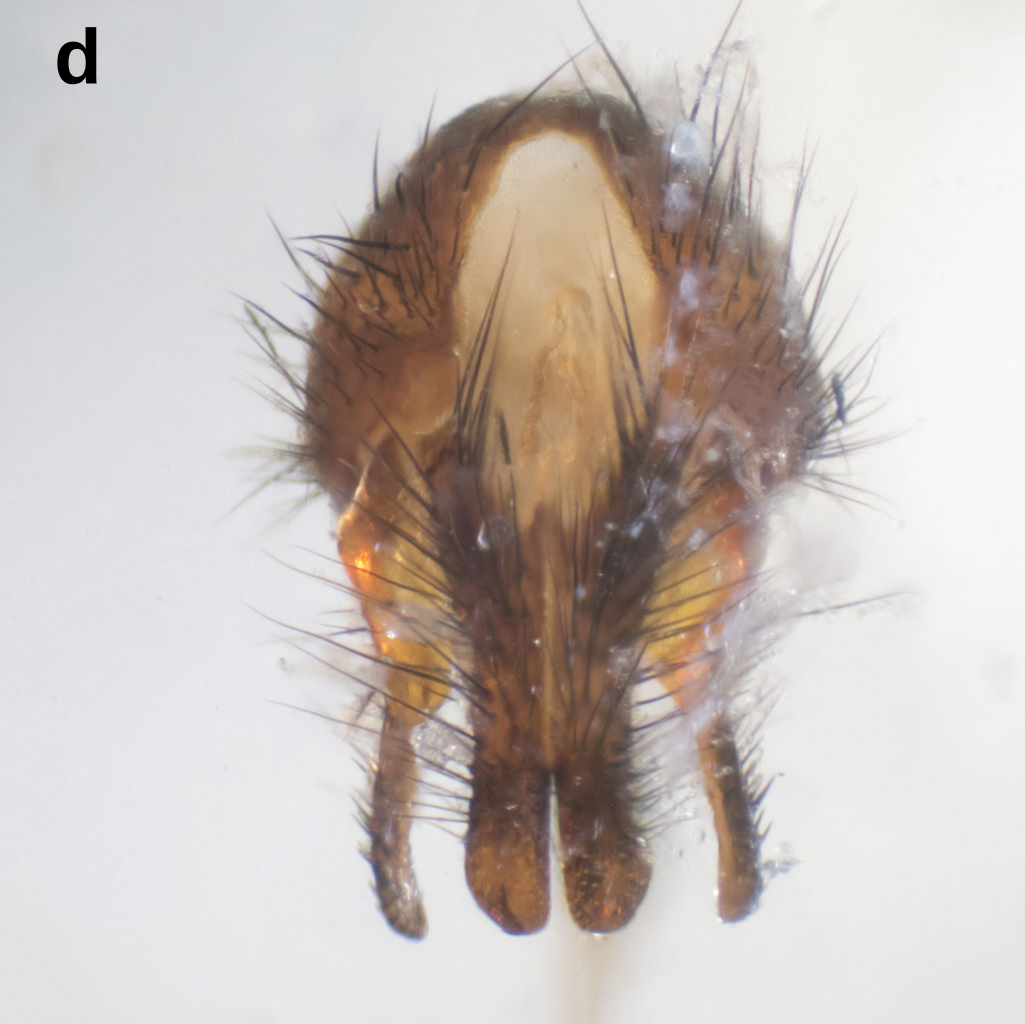
dorsal view

**Figure 14e.**
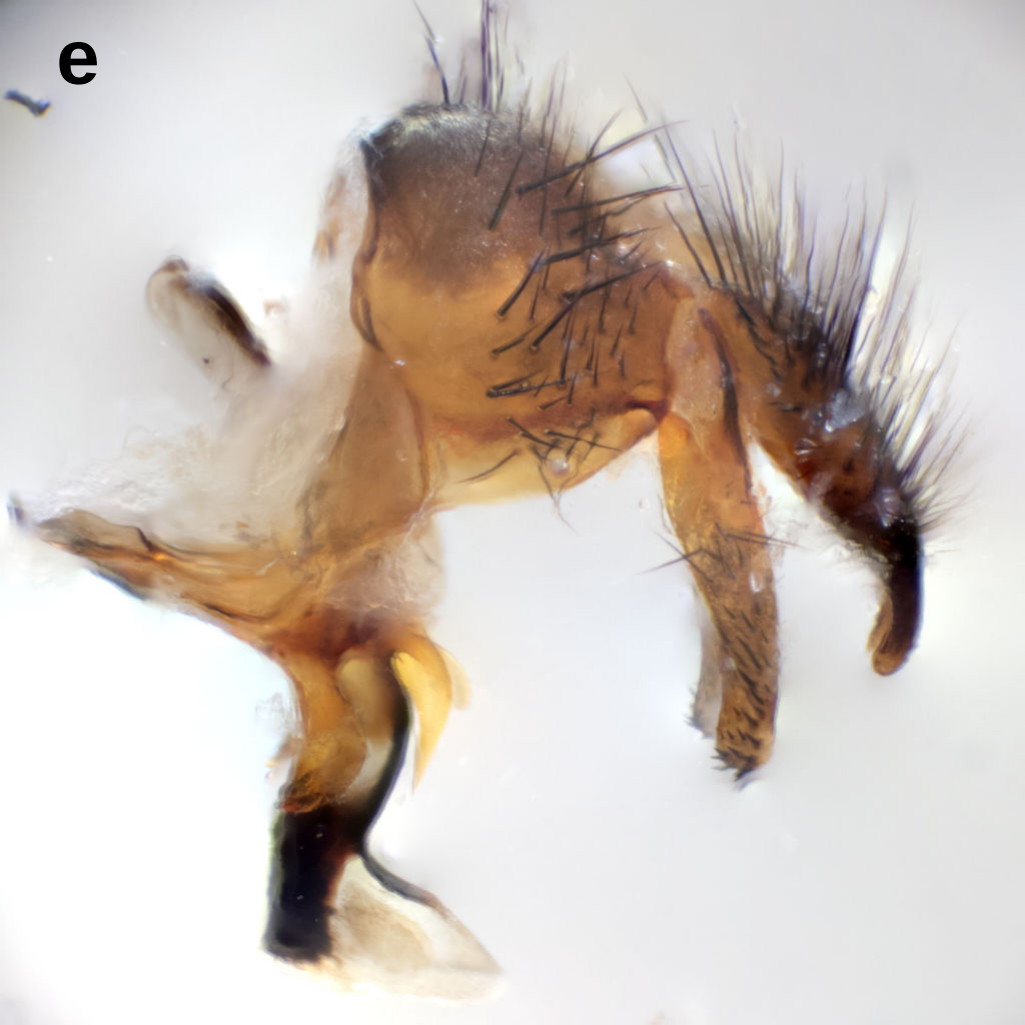
lateral view

**Figure 14f.**
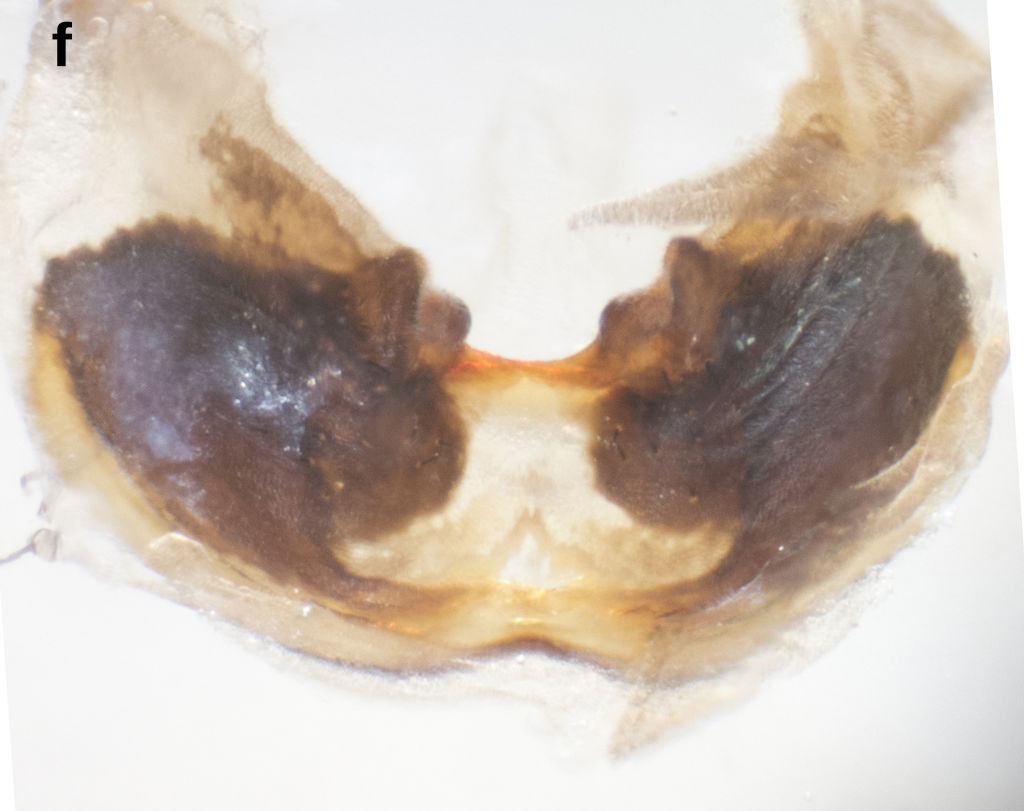
sternite 5, ventral view

**Figure 15a.**
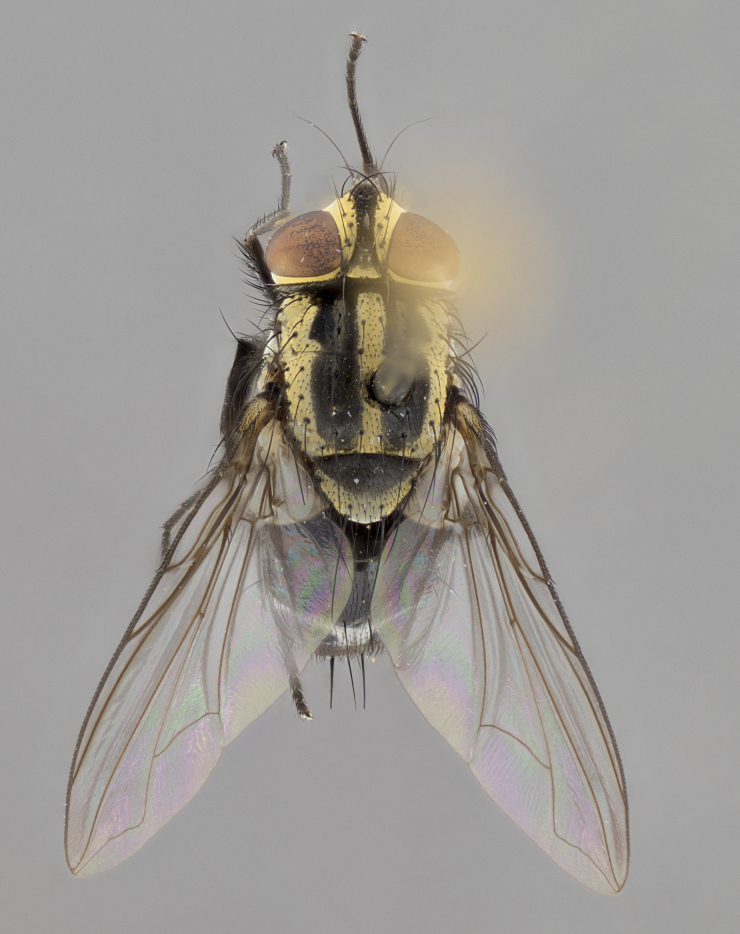
dorsal view

**Figure 15b.**
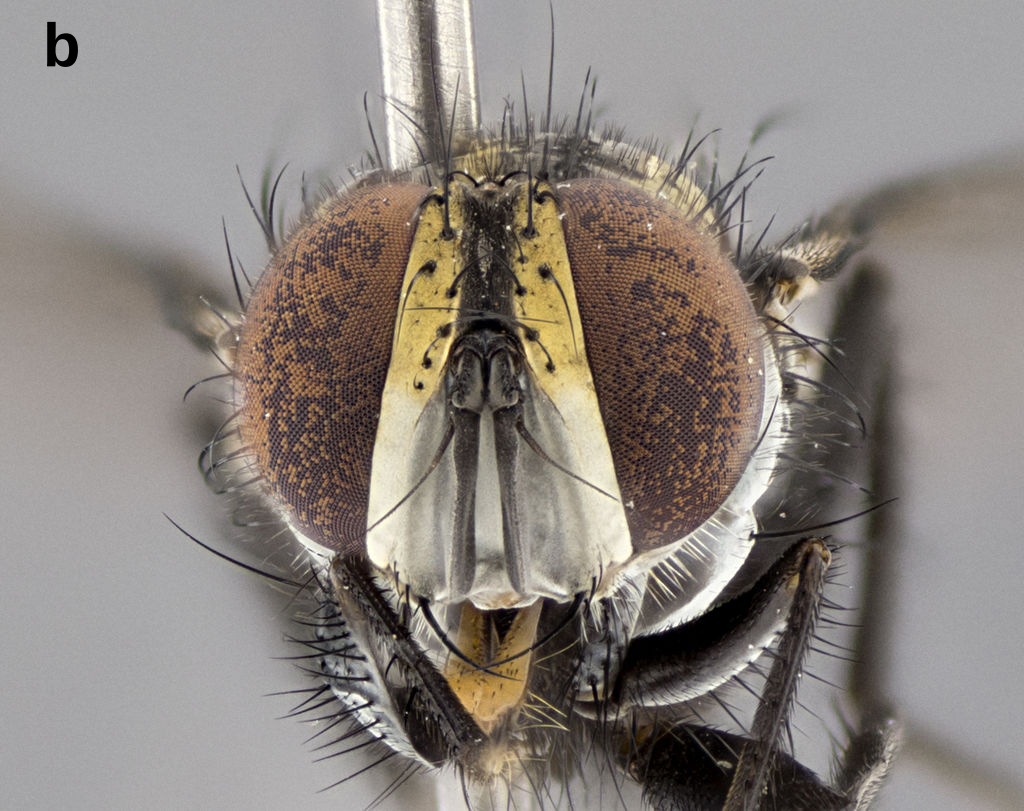
frontal view

**Figure 15c.**
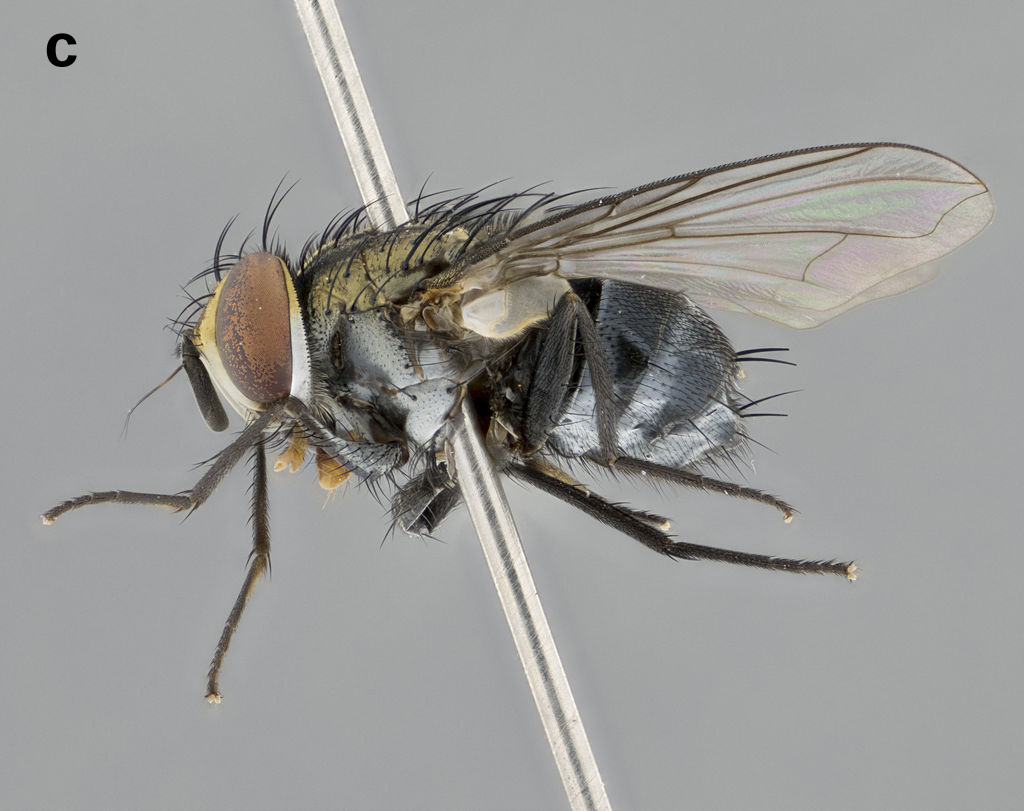
lateral view

**Figure 16a.**
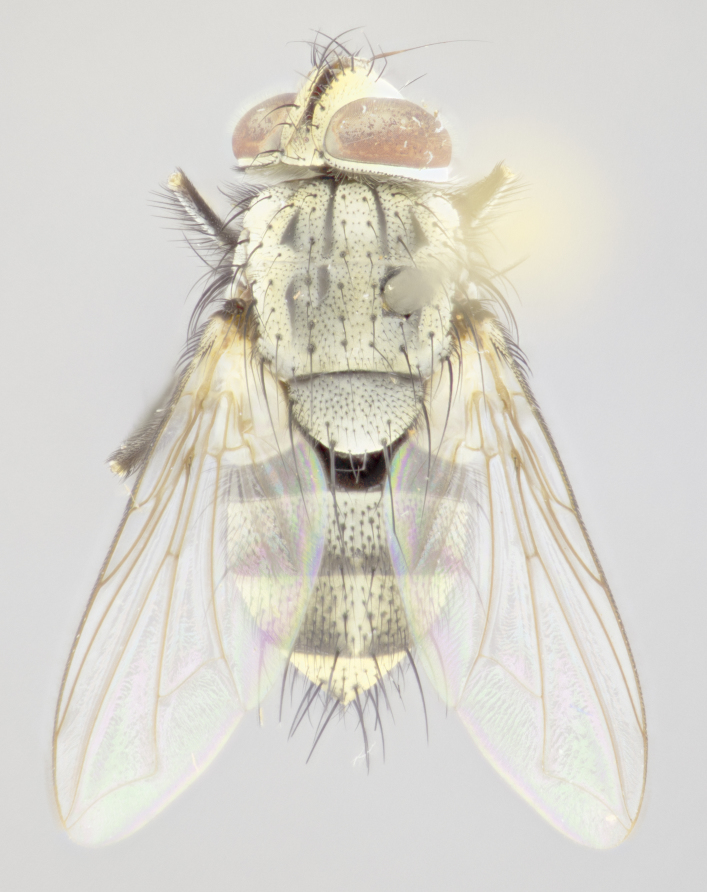
dorsal view

**Figure 16b.**
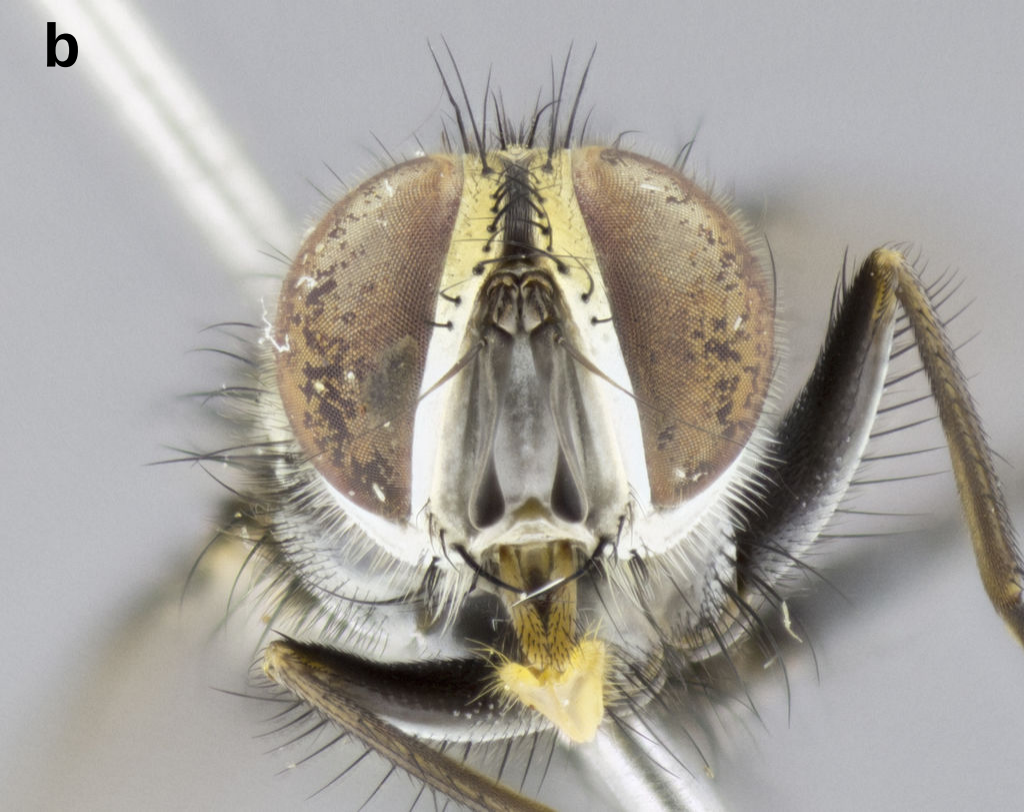
frontal view

**Figure 16c.**
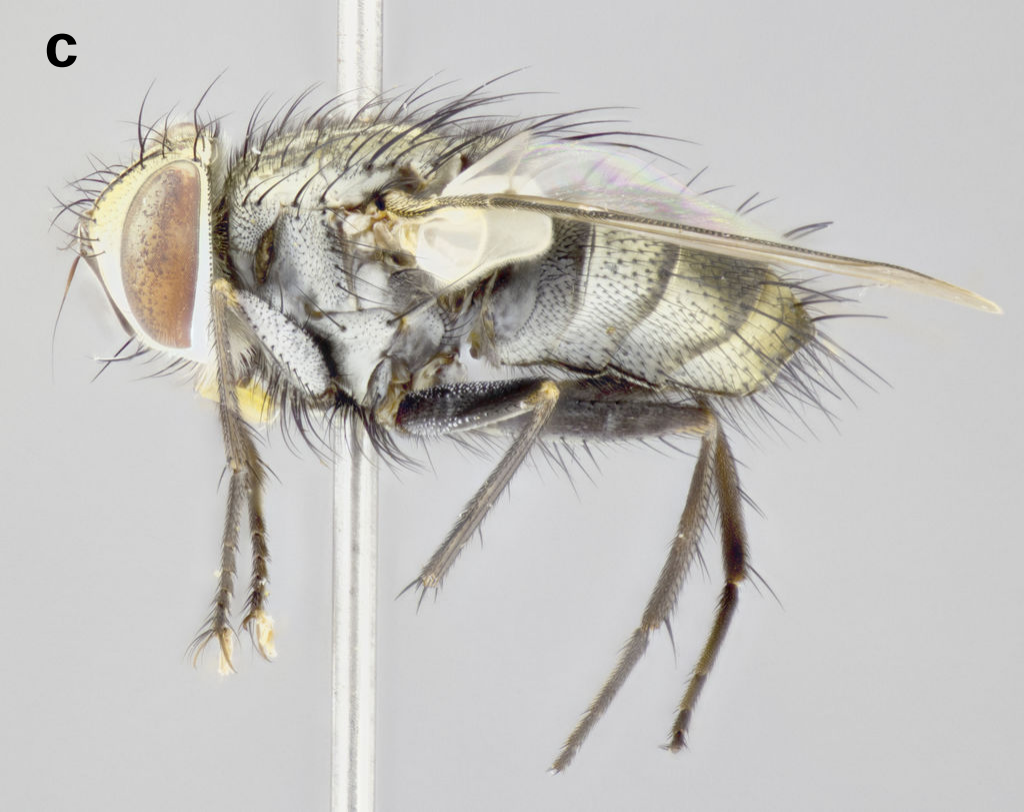
lateral view

**Figure 16d.**
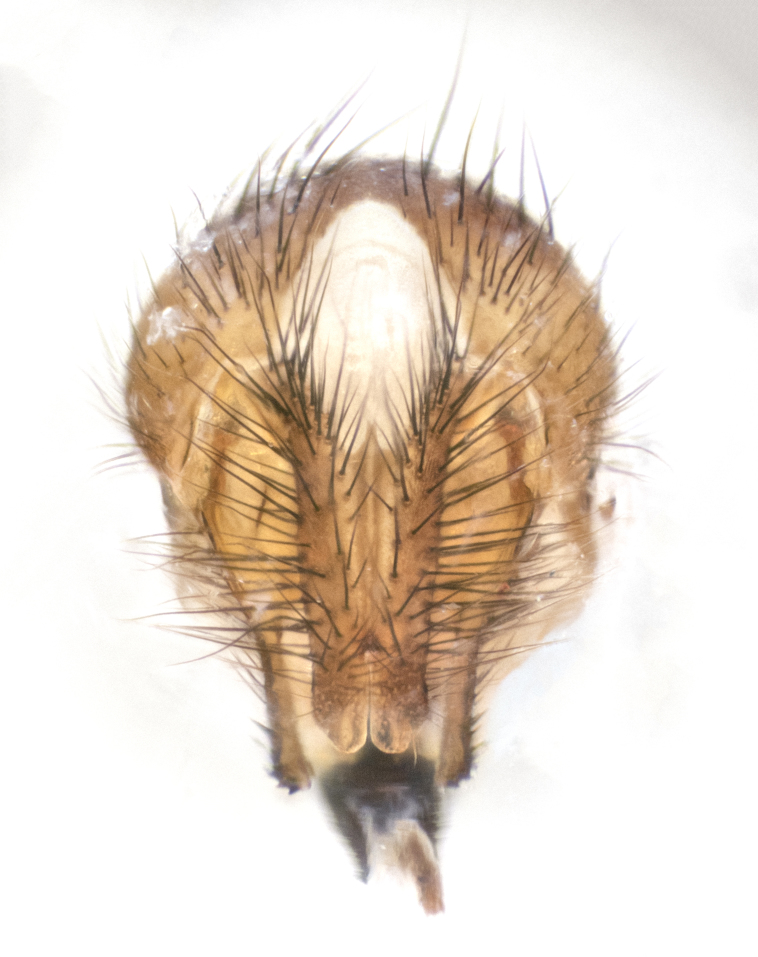
dorsal view

**Figure 16e.**
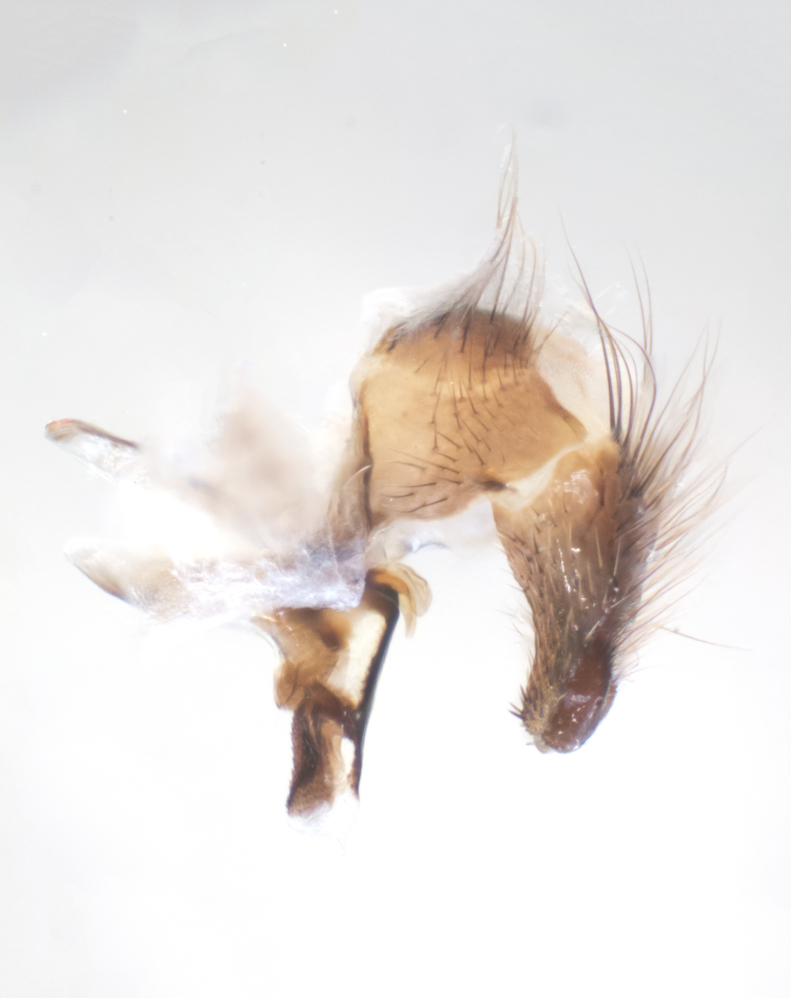
lateral view

**Figure 16f.**
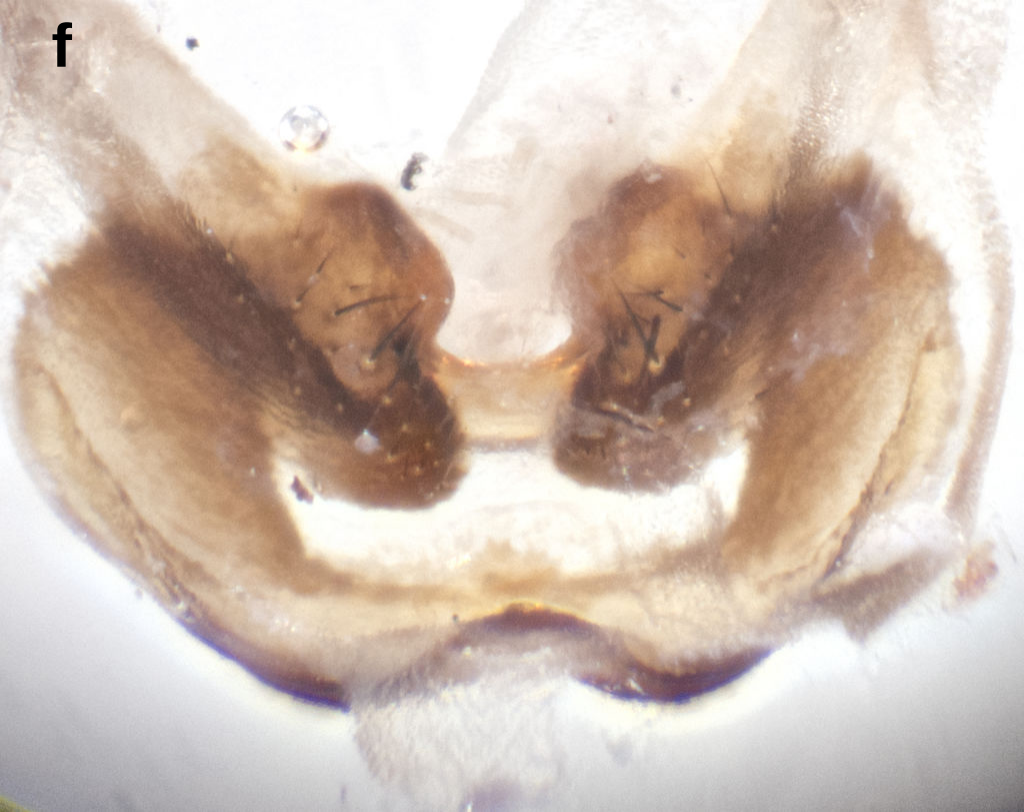
sternite 5, ventral view

**Figure 17a.**
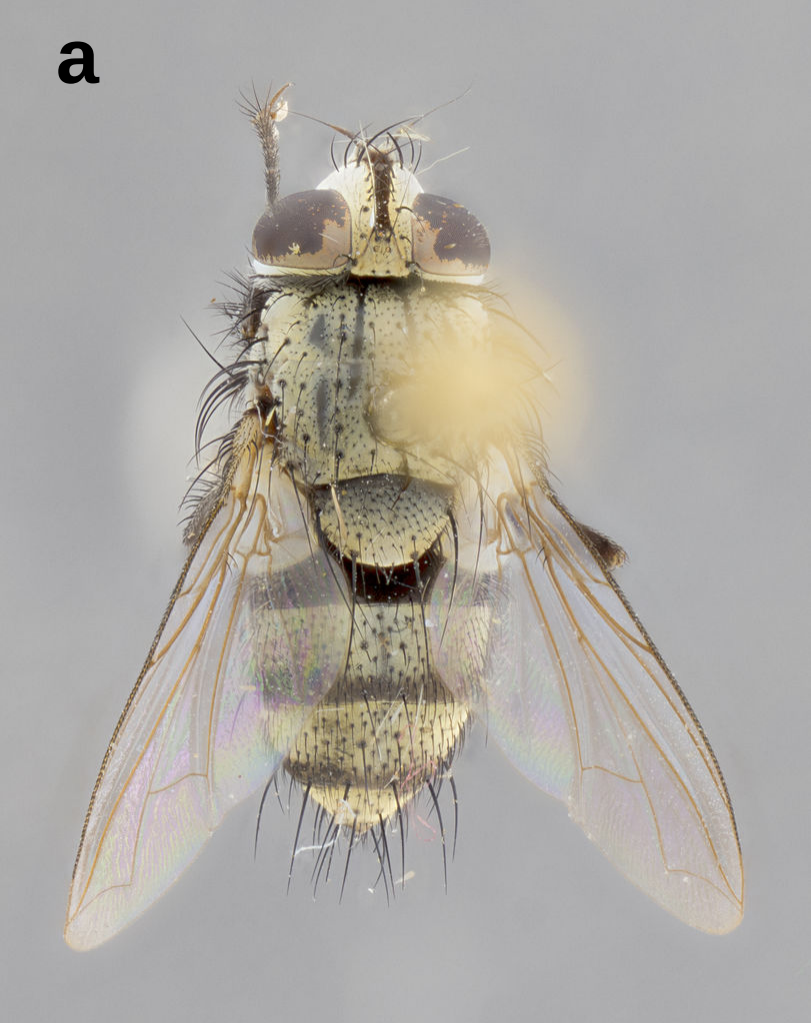


**Figure 17b.**
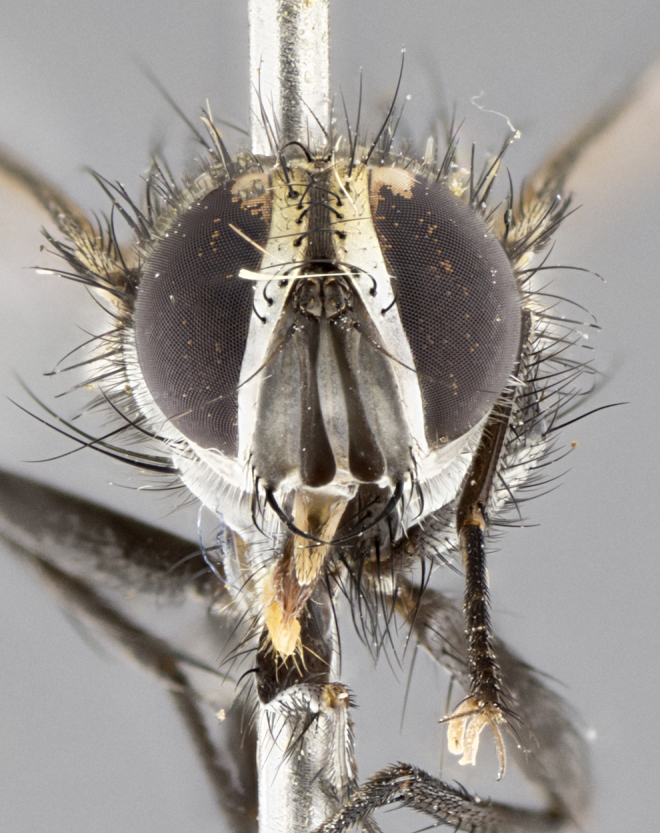


**Figure 17c.**
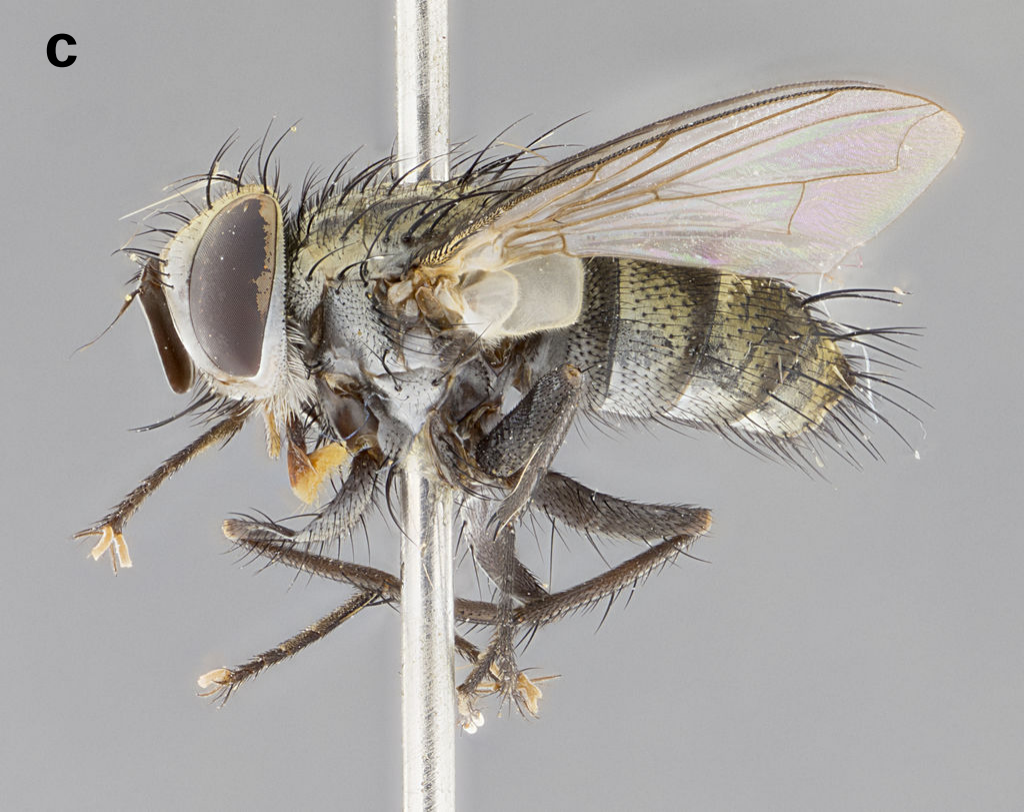


**Figure 17d.**
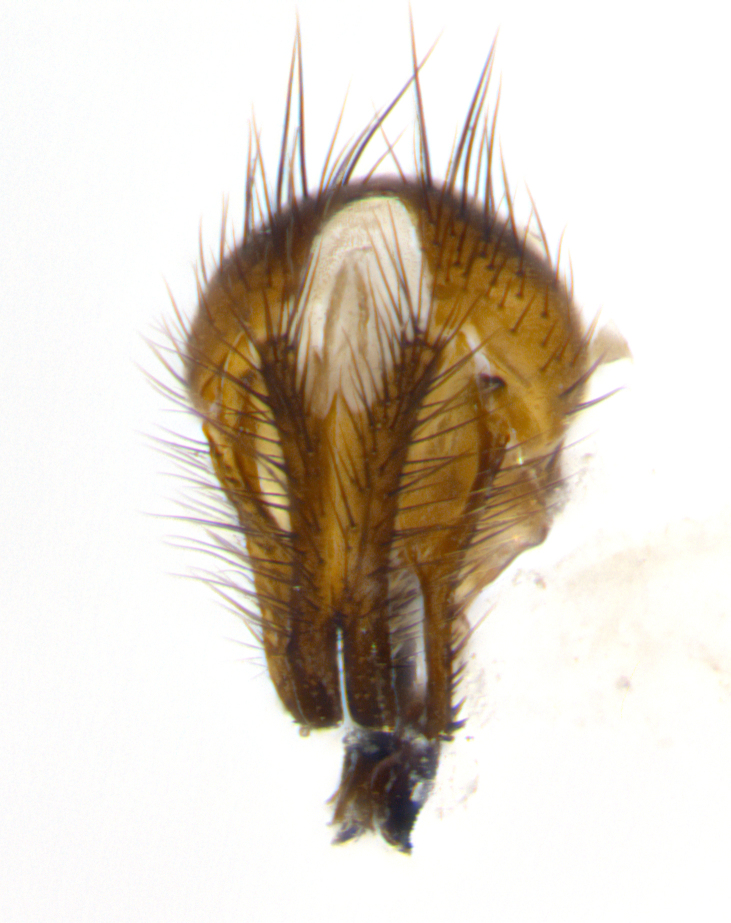


**Figure 17e.**
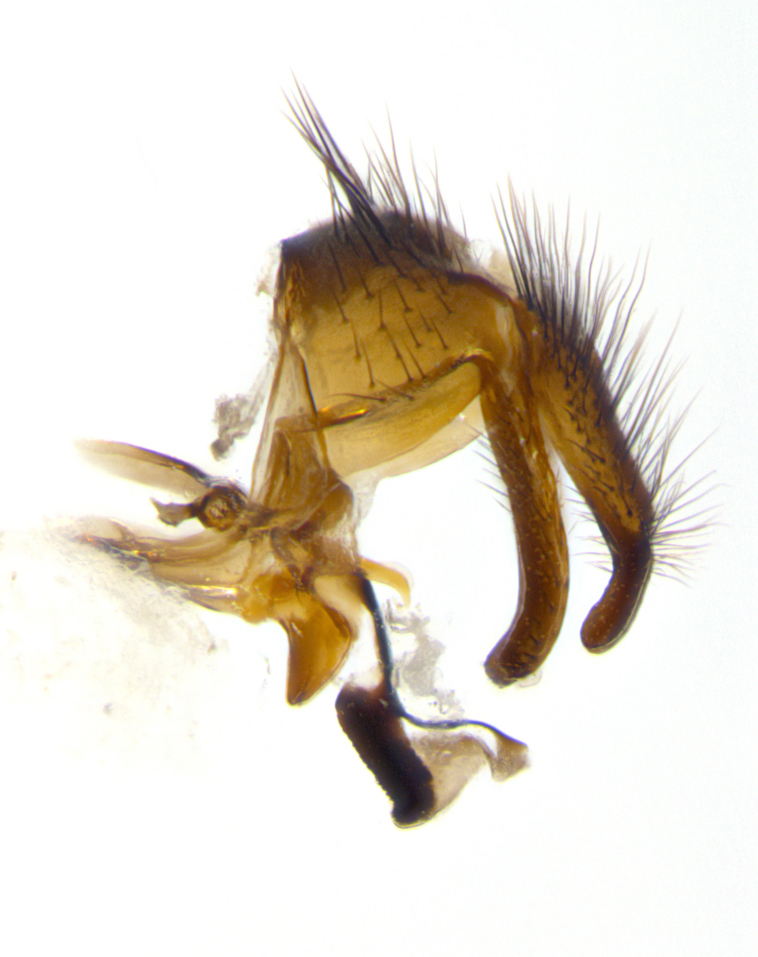


**Figure 17f.**
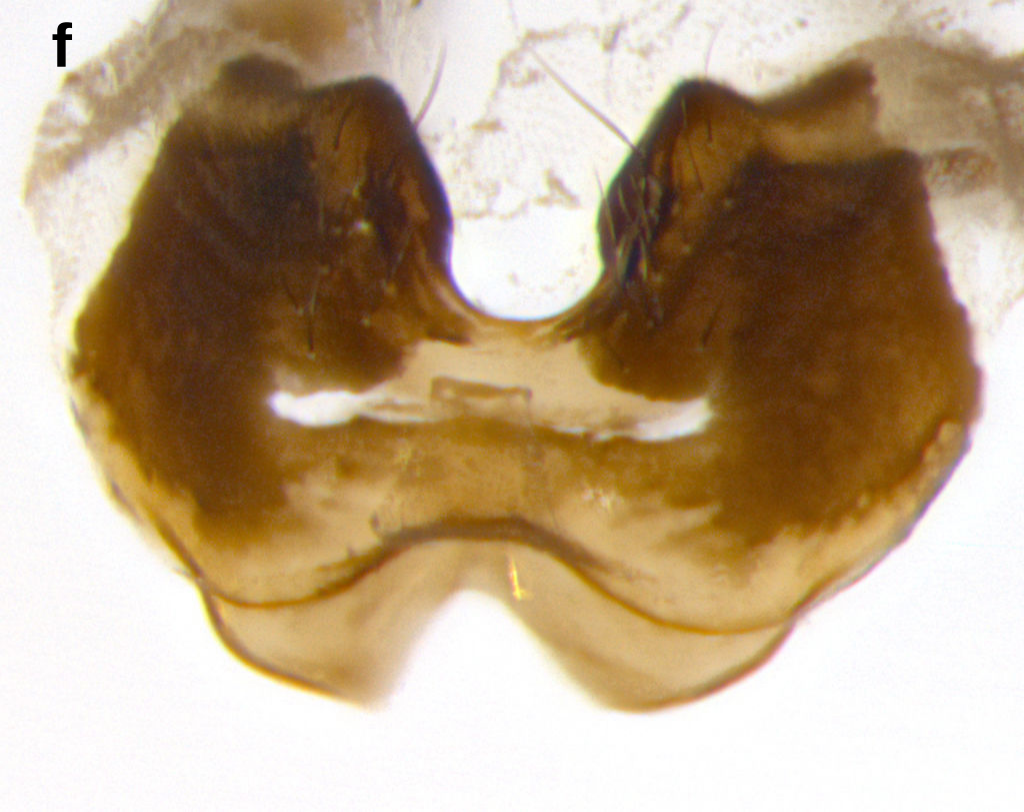


**Figure 18a.**
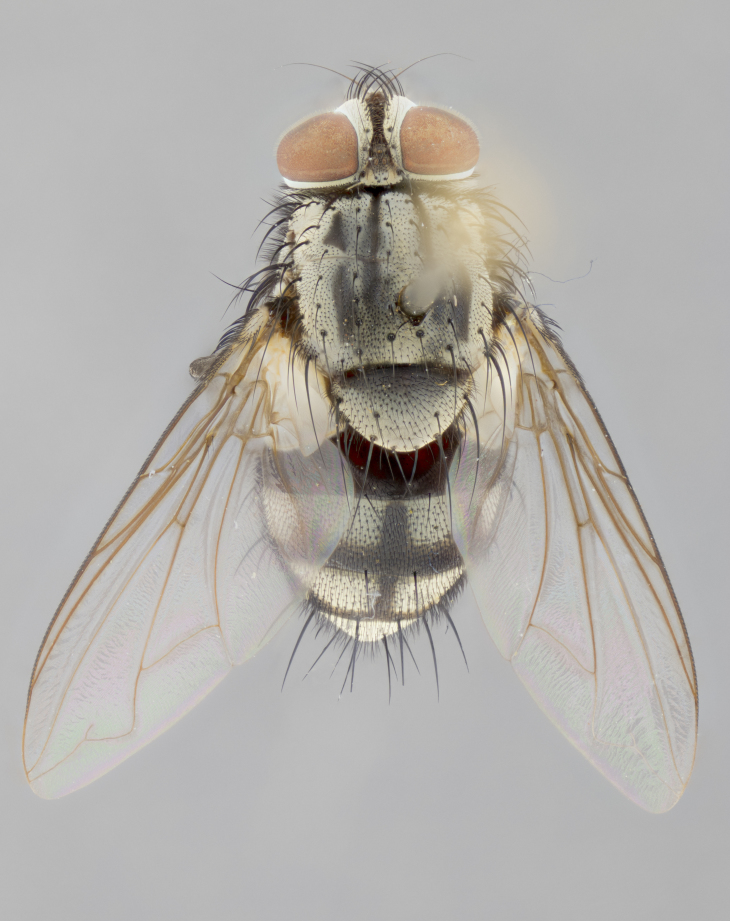
dorsal view

**Figure 18b.**
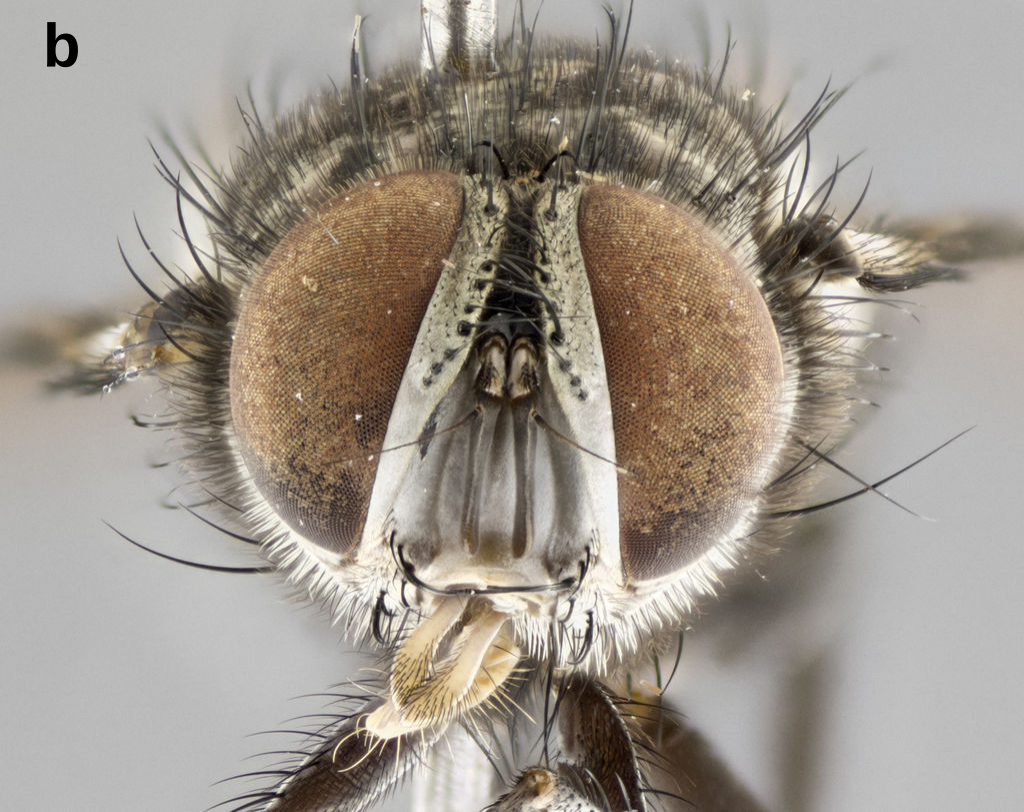
frontal view

**Figure 18c.**
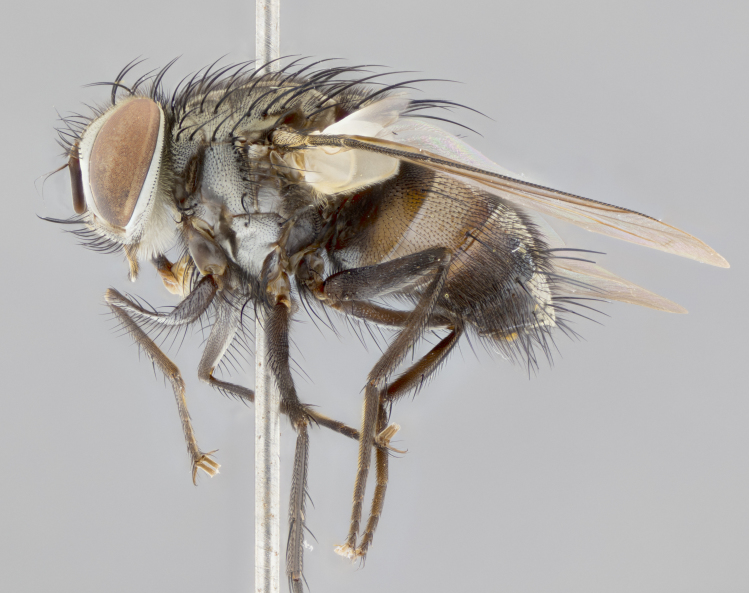
lateral view

**Figure 18d.**
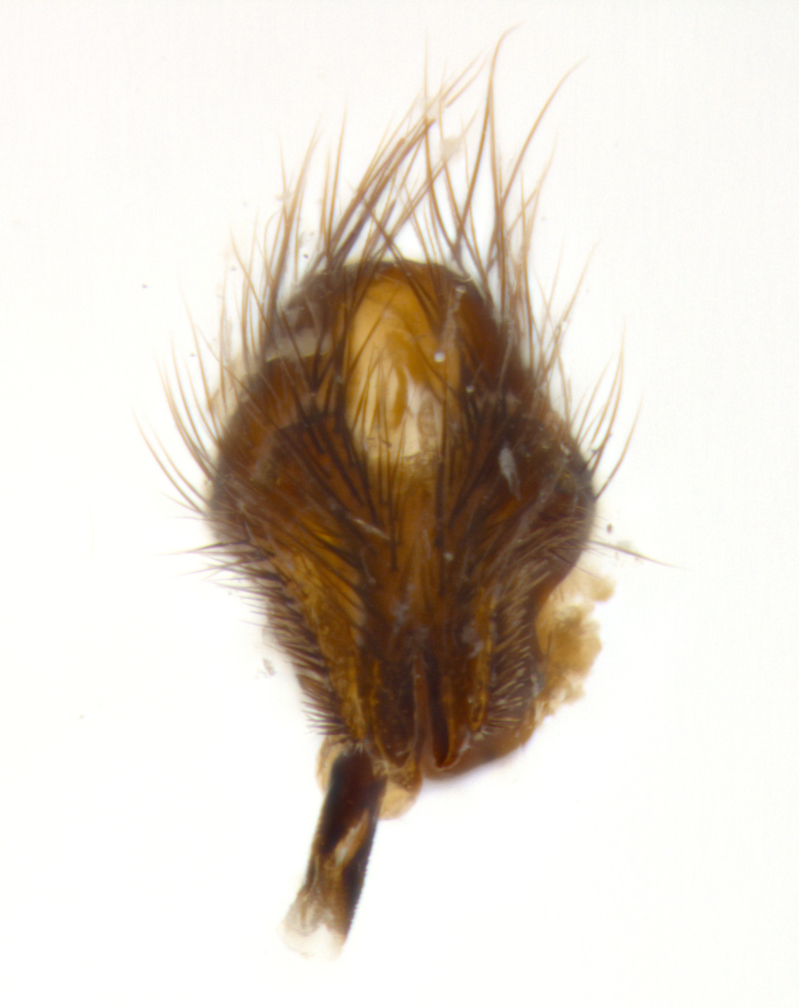
dorsal view

**Figure 18e.**
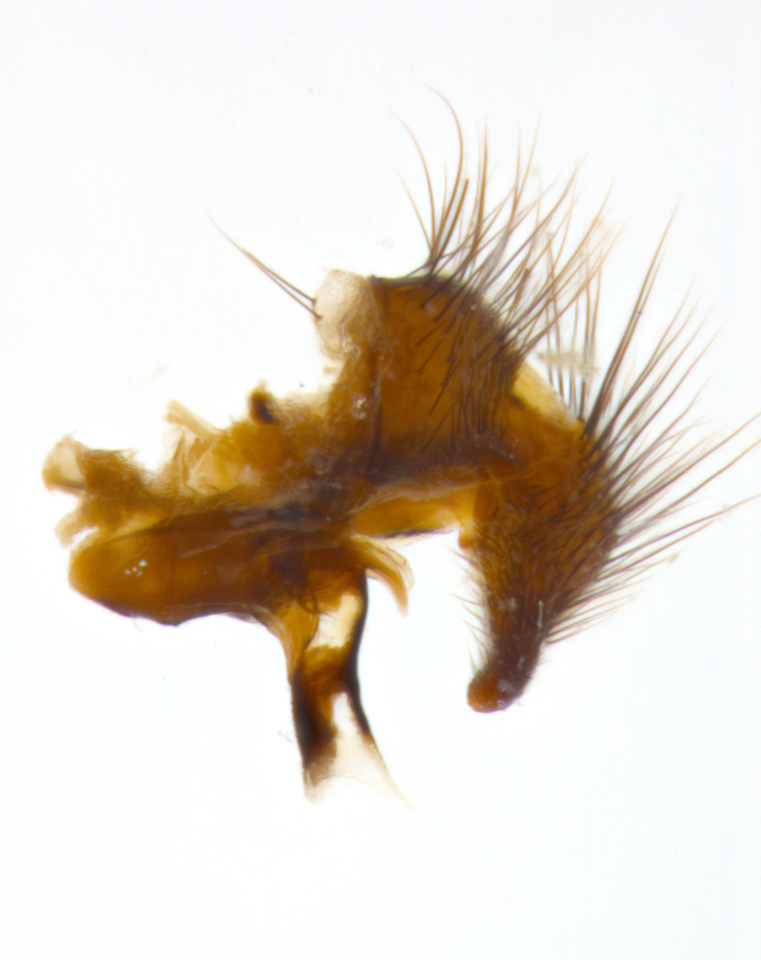
lateral view

**Figure 18f.**
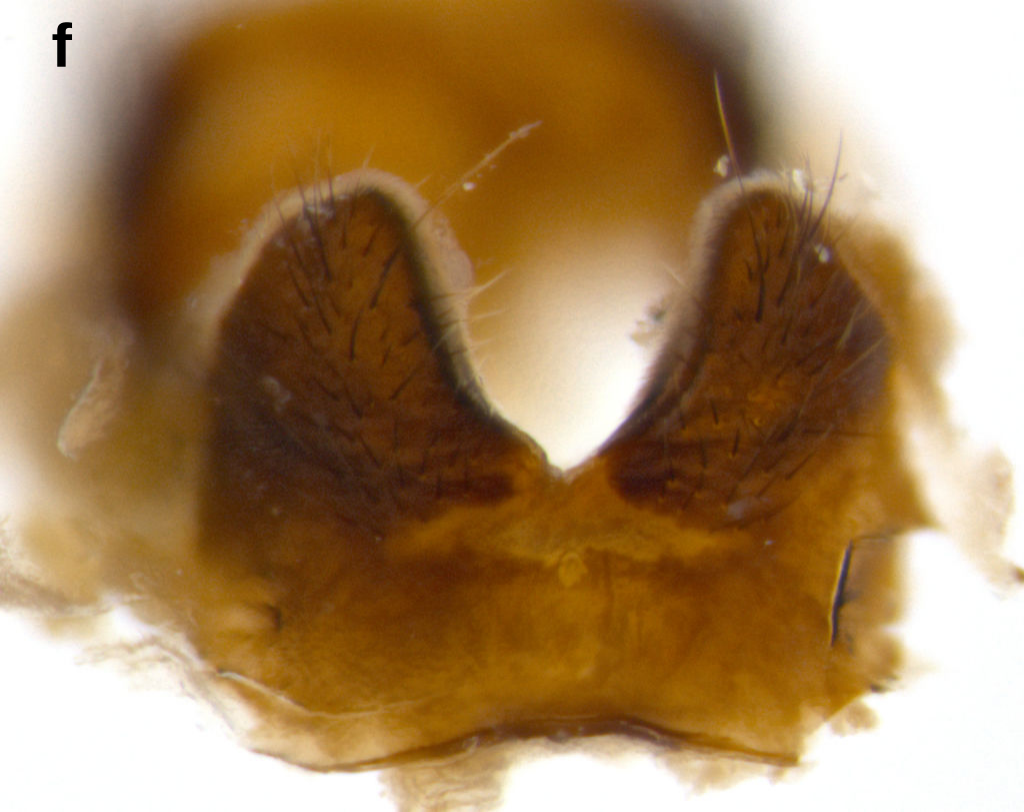
sternite 5, ventral view

**Figure 19a.**
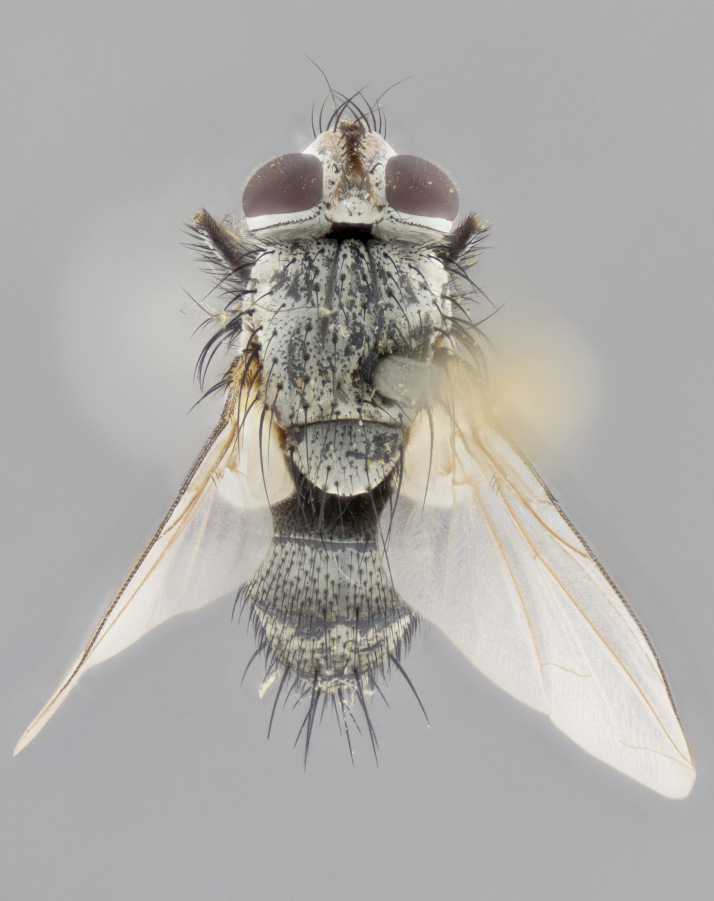
dorsal view

**Figure 19b.**
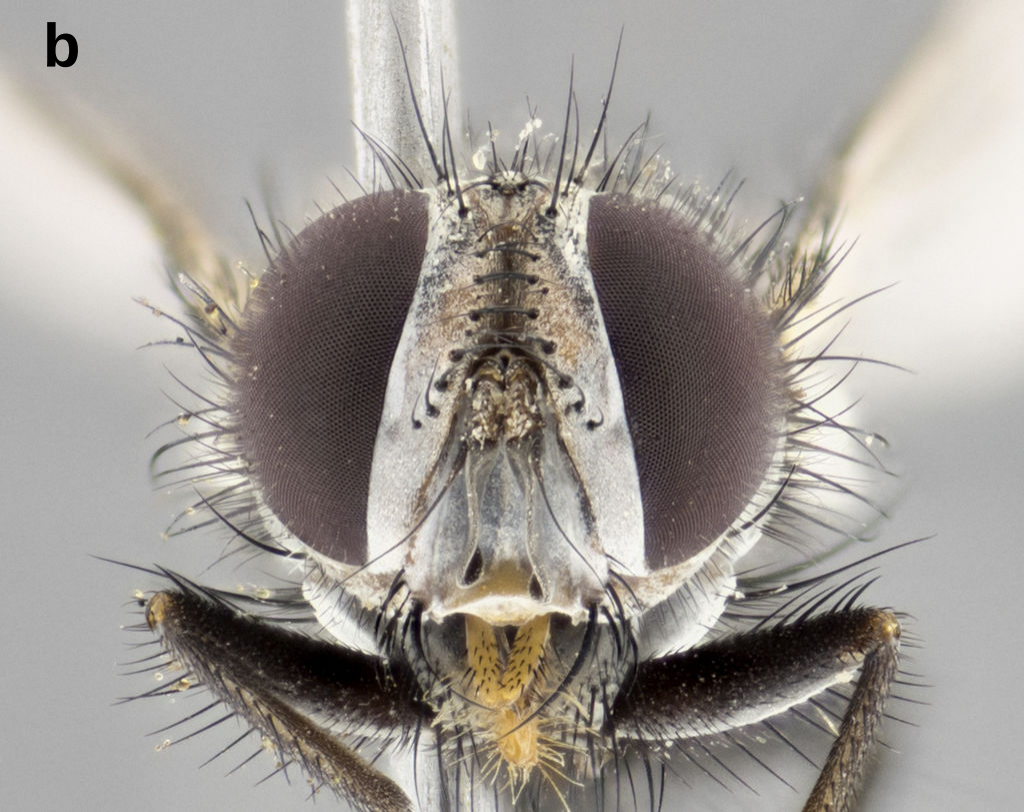
frontal view

**Figure 19c.**
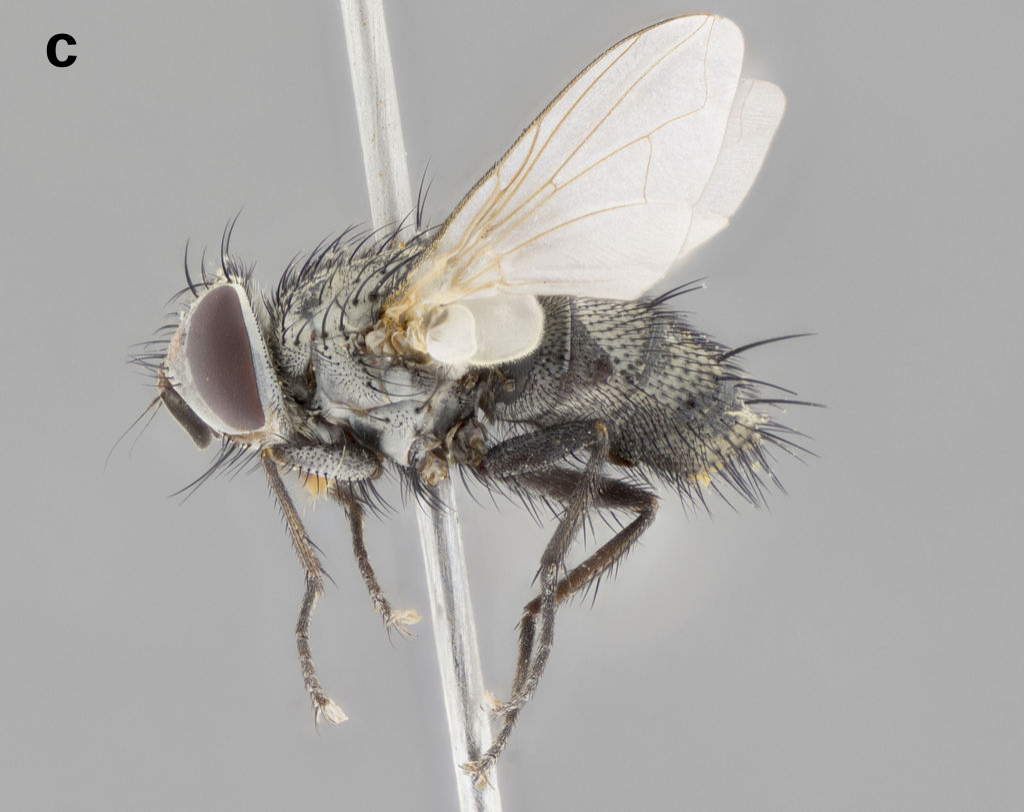
lateral view

**Figure 19d.**
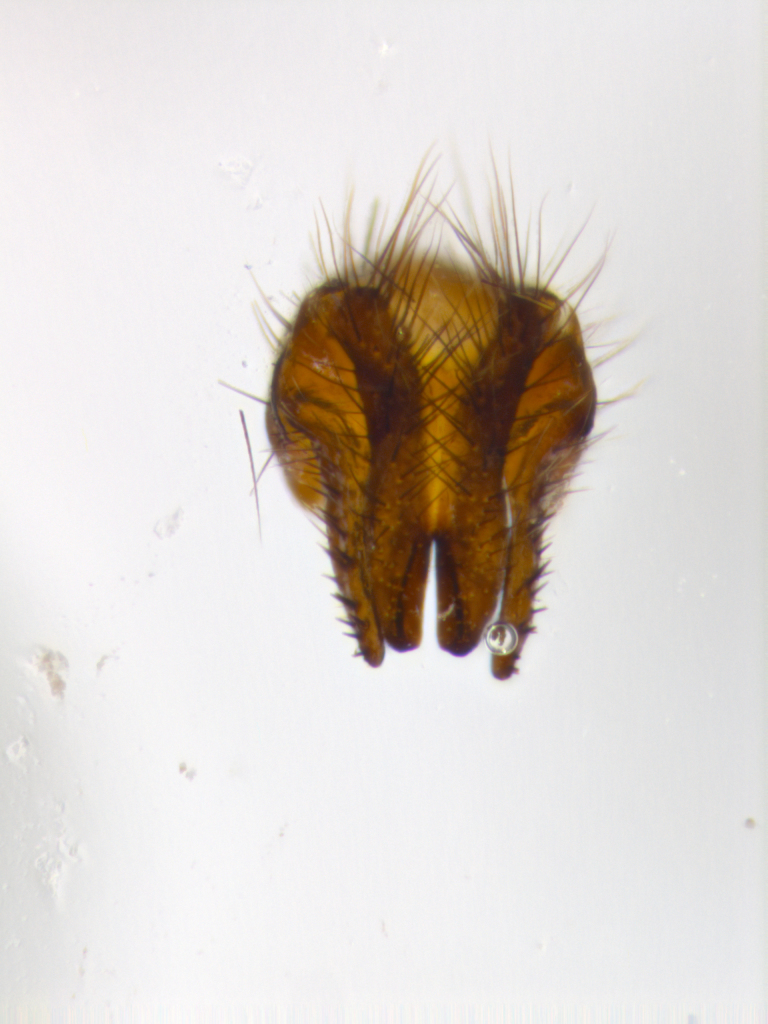
dorsal view

**Figure 19e.**
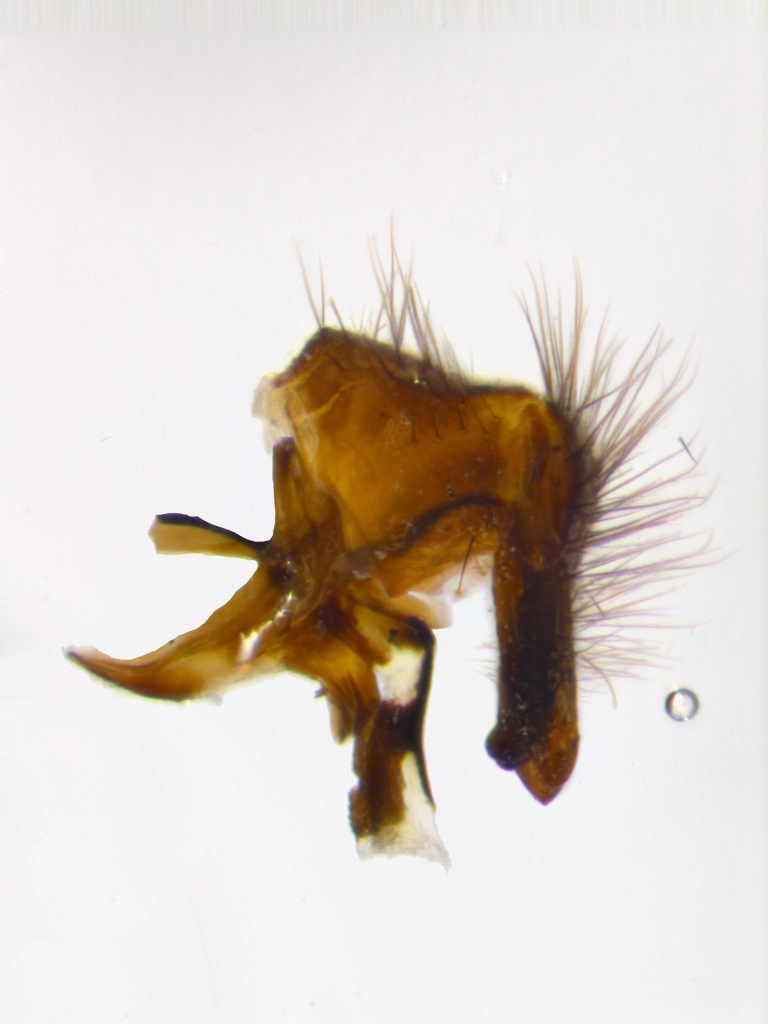
lateral view

**Figure 19f.**
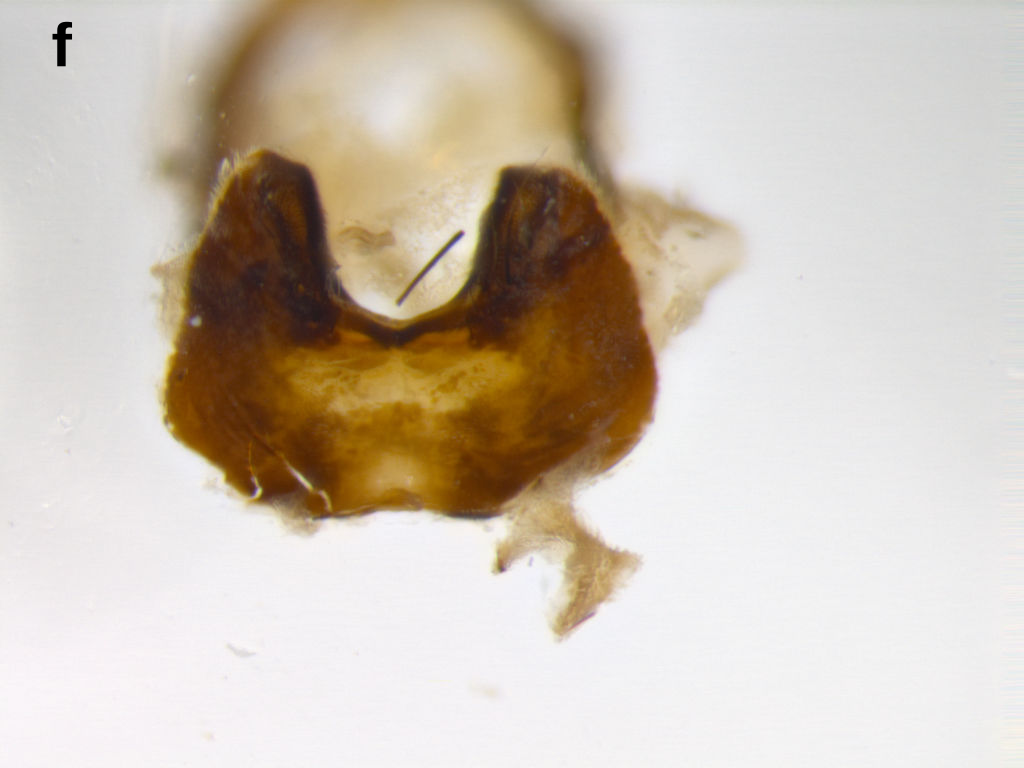
sternite 5, ventral view

**Figure 20a.**
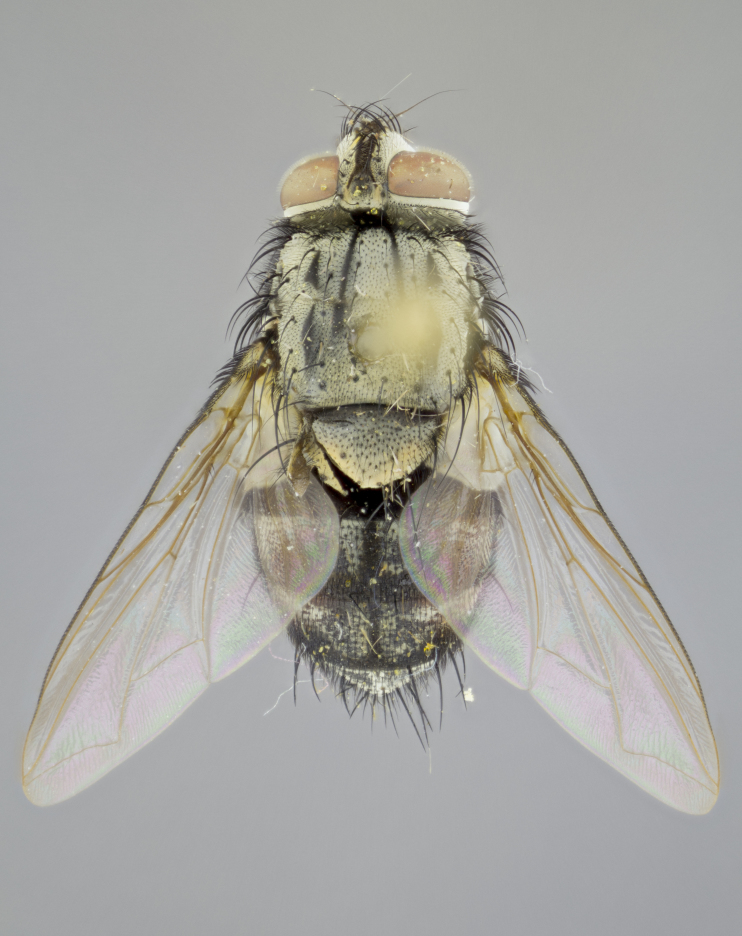
dorsal view

**Figure 20b.**
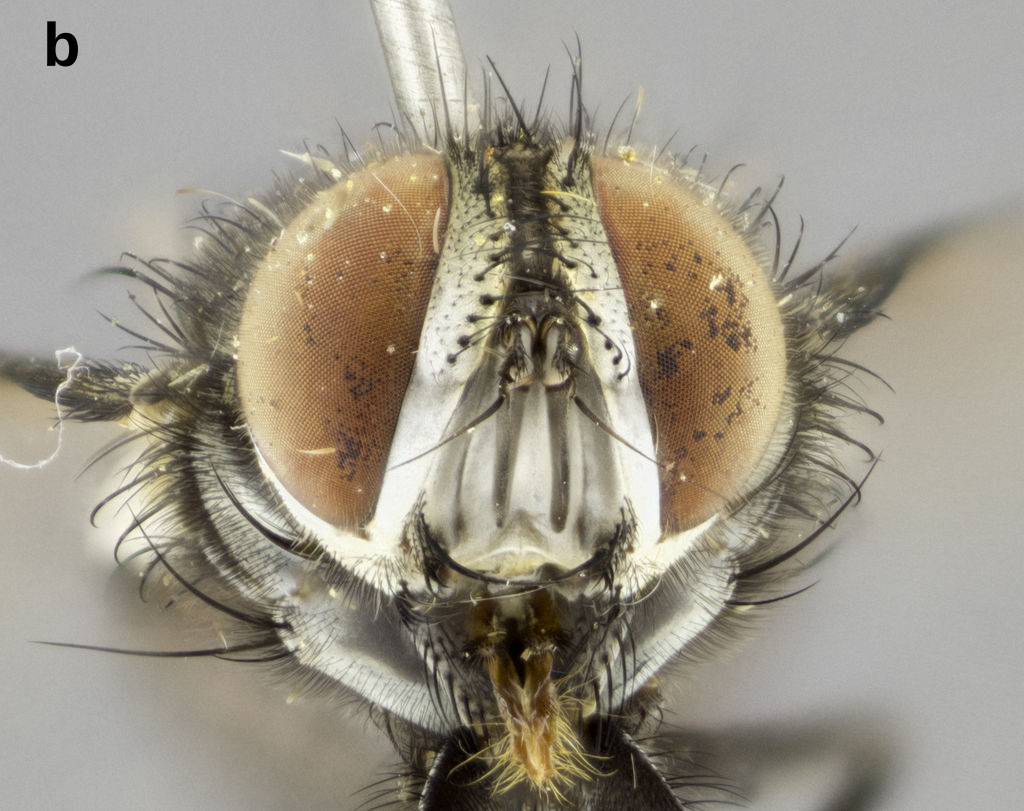
frontal view

**Figure 20c.**
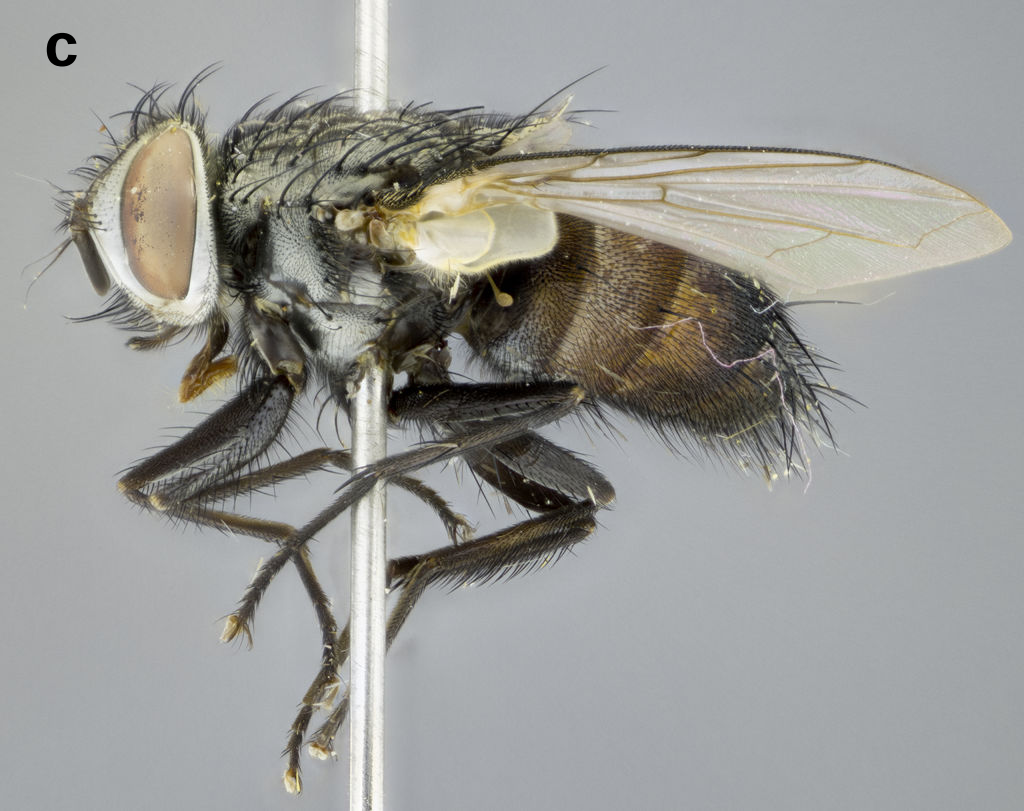
lateral view

**Figure 20d.**
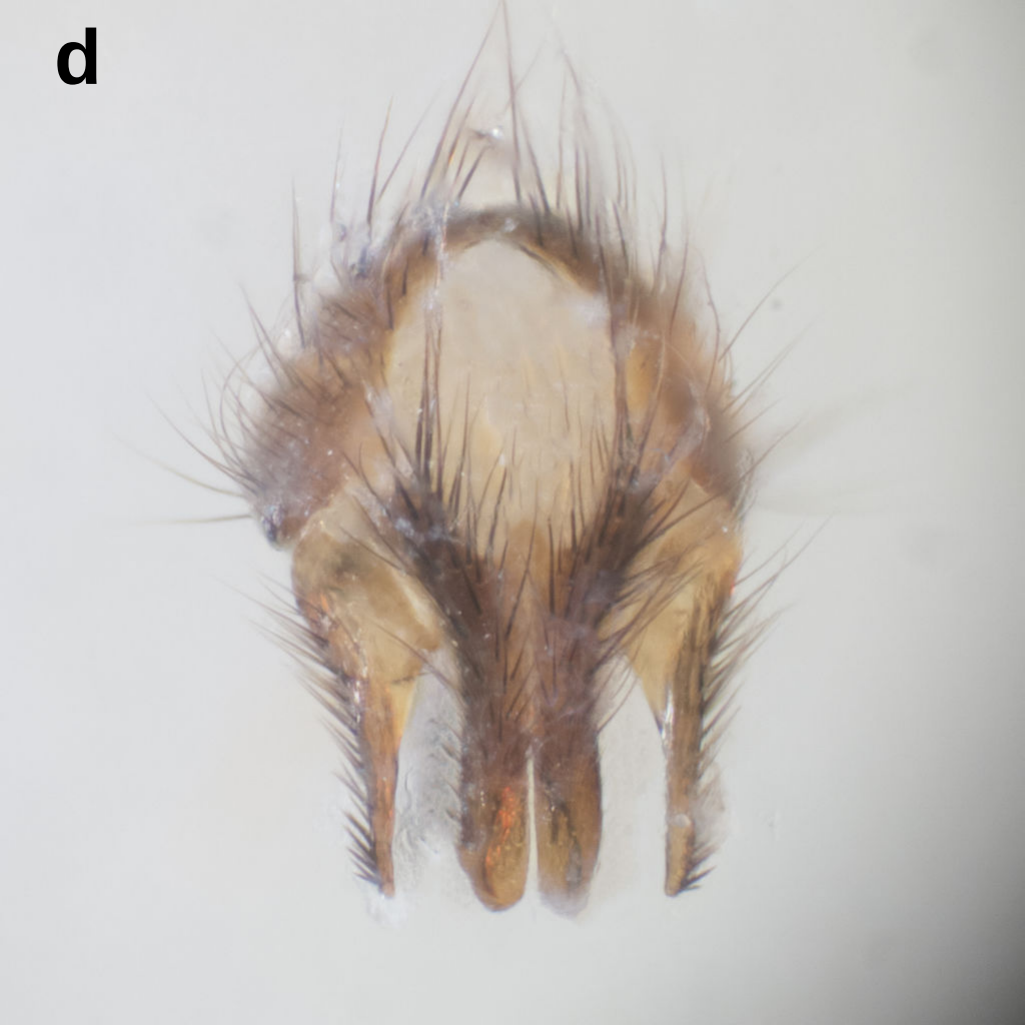
dorsal view

**Figure 20e.**
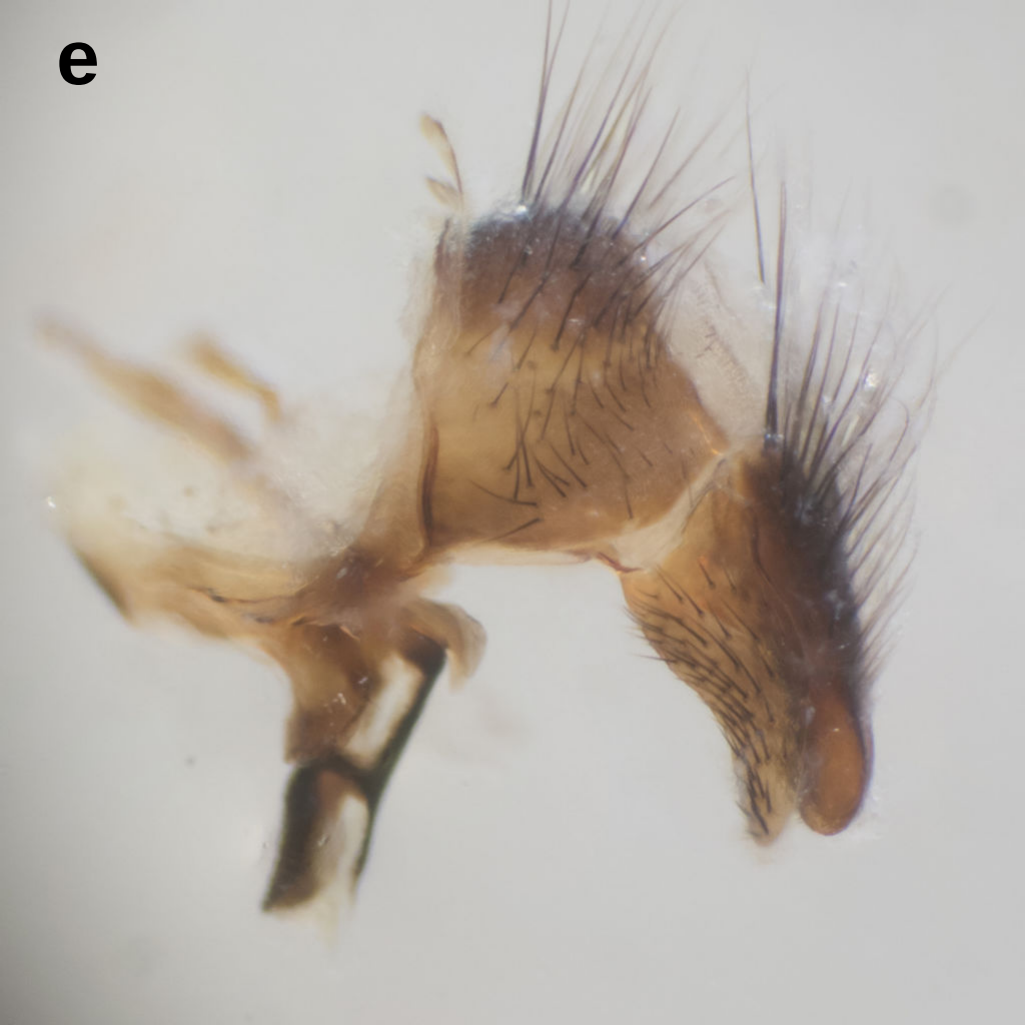
lateral view

**Figure 20f.**
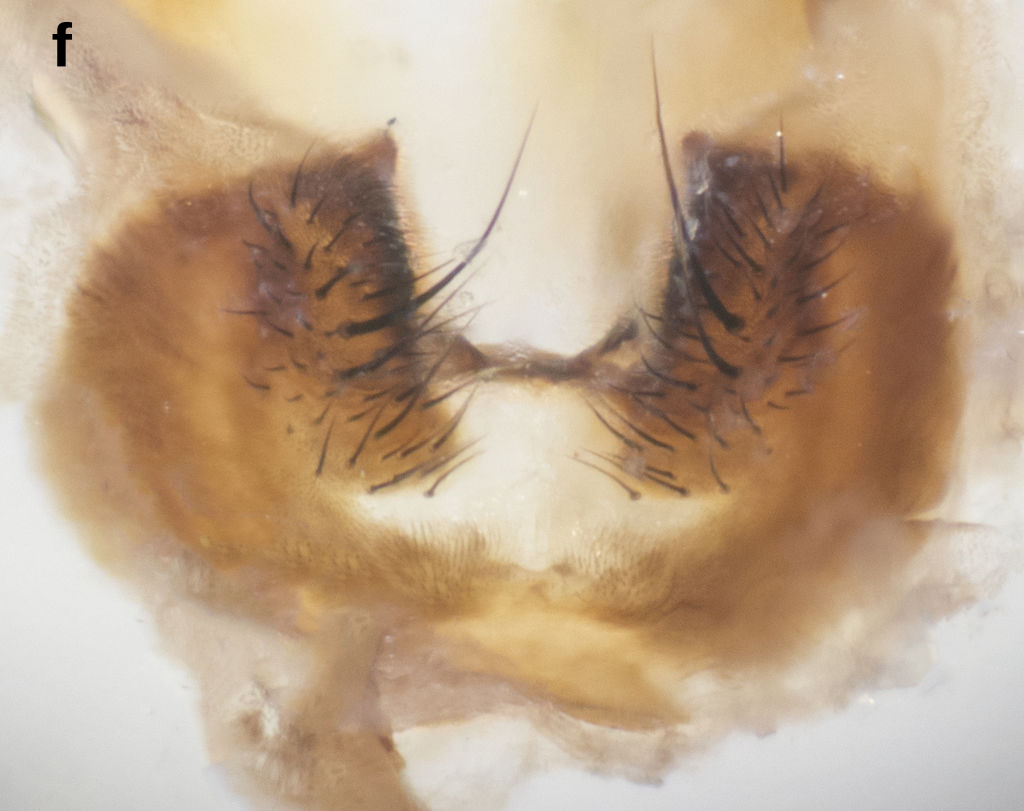
sternite 5, ventral view

**Figure 21a.**
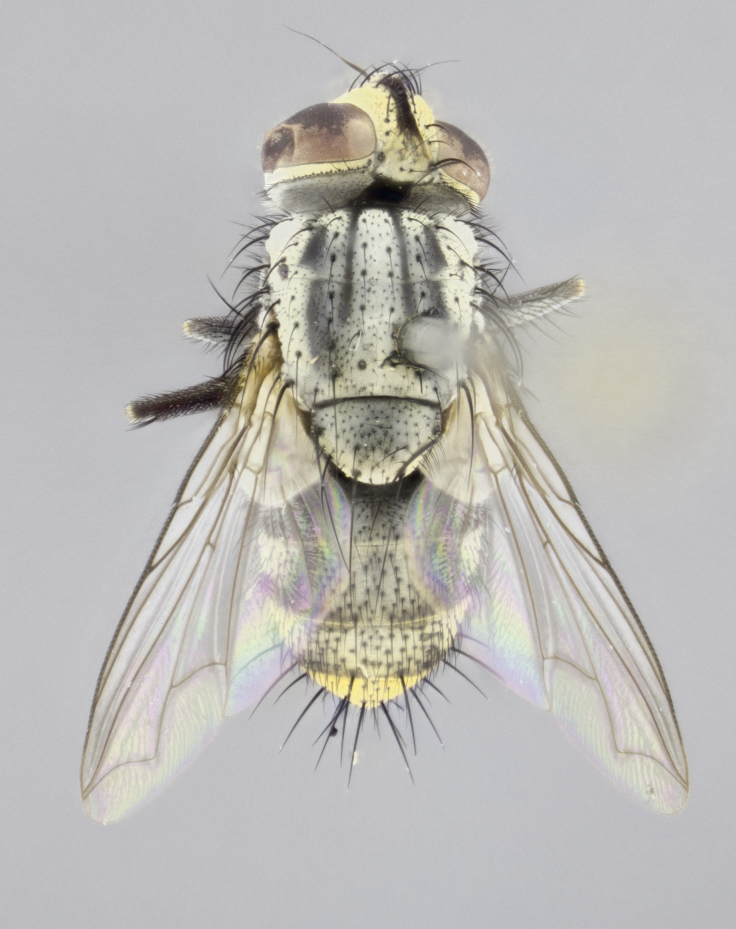
dorsal view

**Figure 21b.**
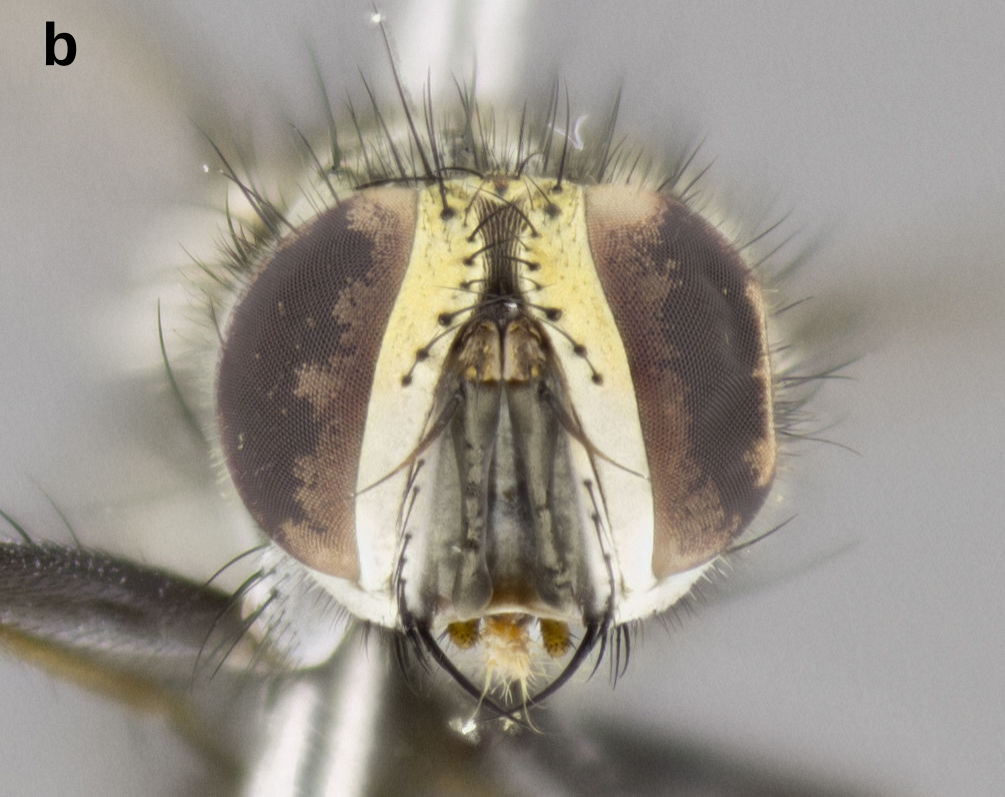
frontal view

**Figure 21c.**
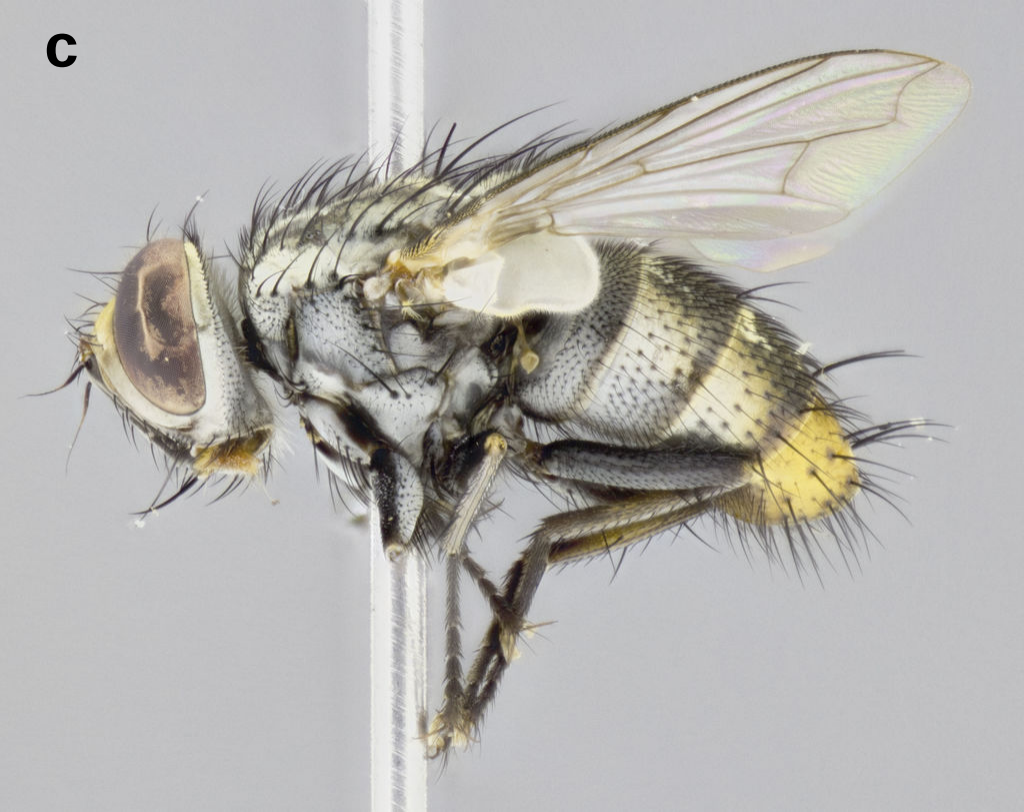
lateral view

**Figure 21d.**
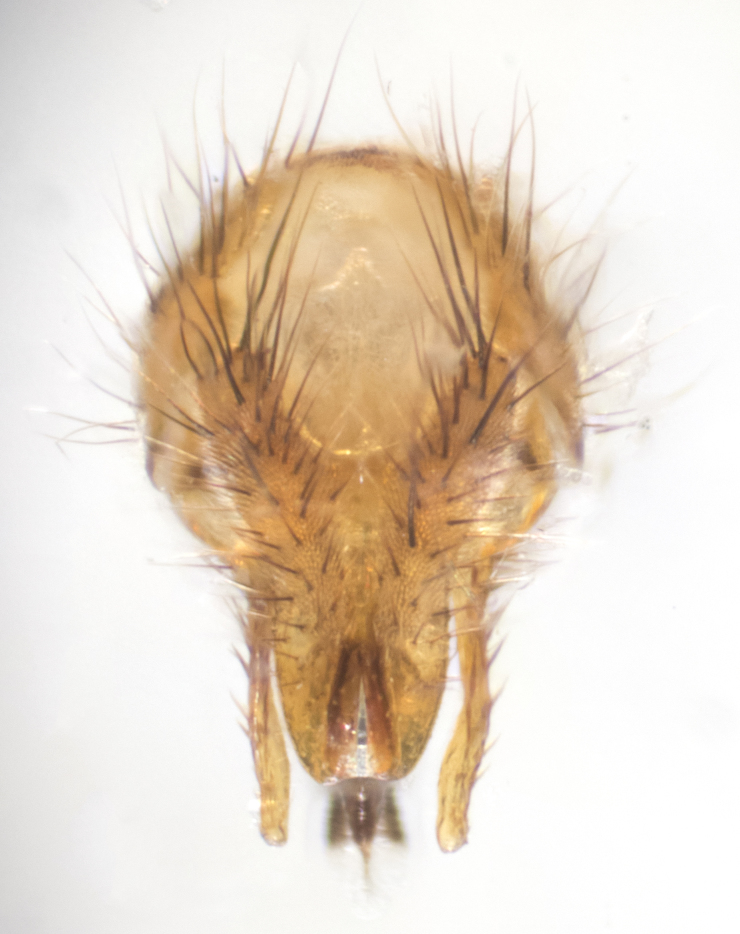
dorsal view

**Figure 21e.**
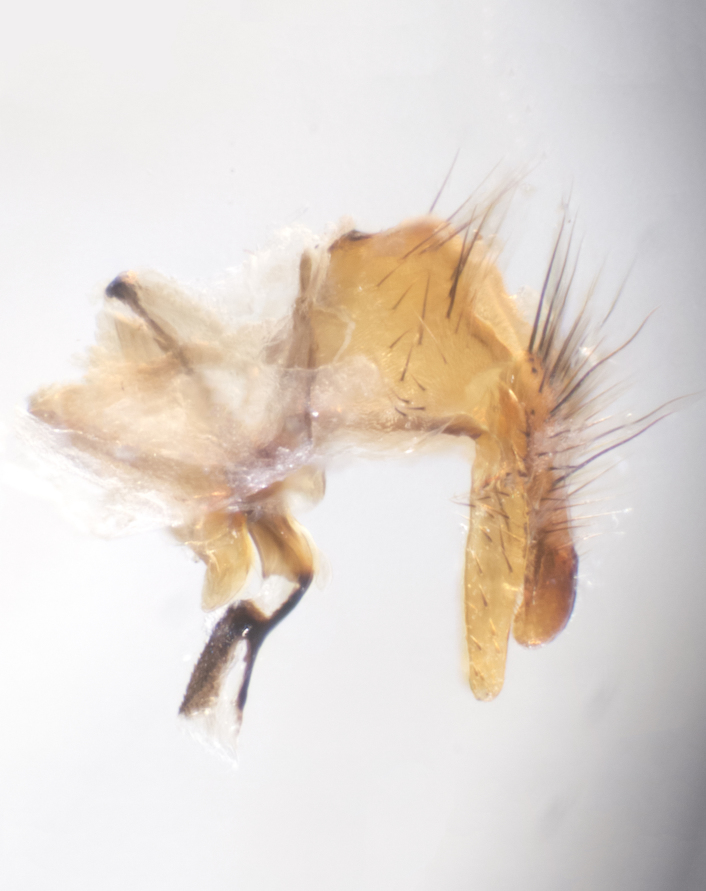
lateral view

**Figure 21f.**
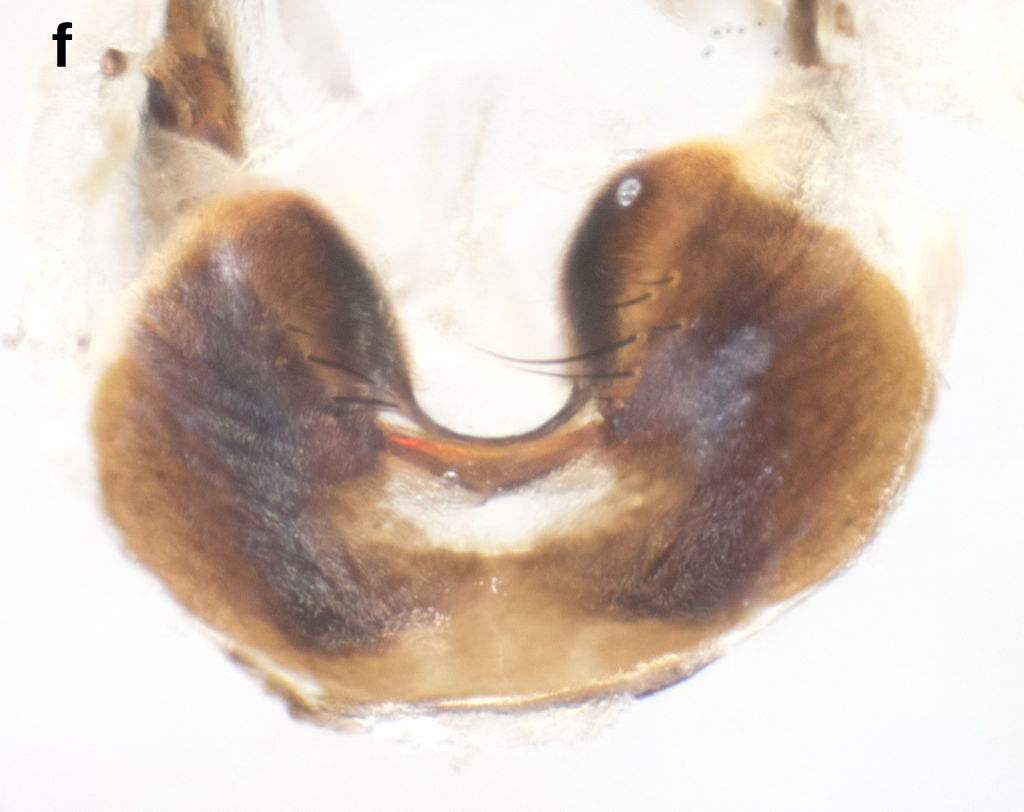
sternite 5, ventral view

**Figure 22a.**
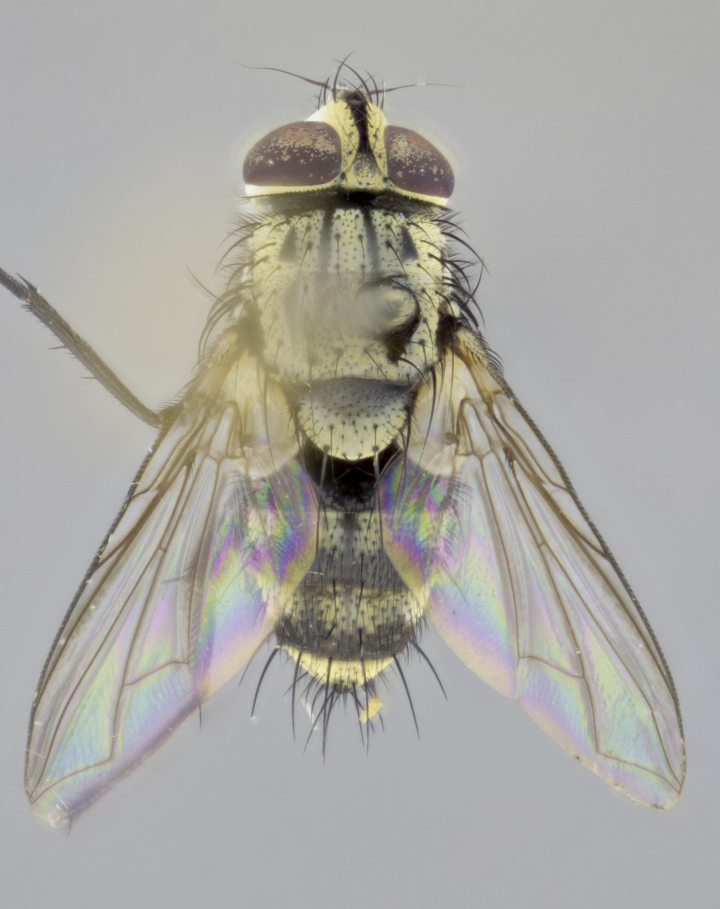
dorsal view

**Figure 22b.**
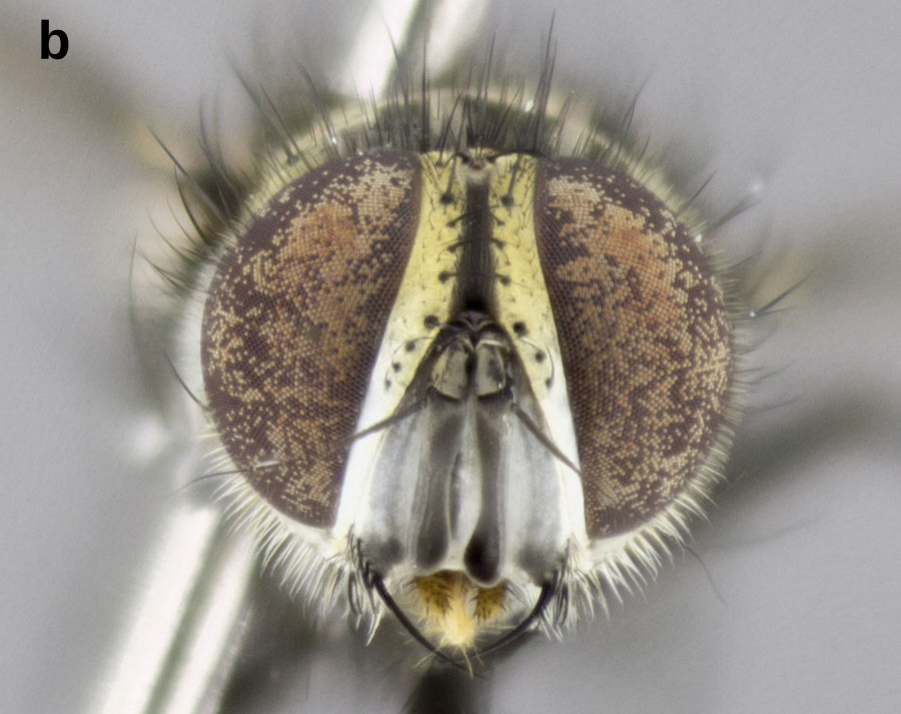
frontal view

**Figure 22c.**
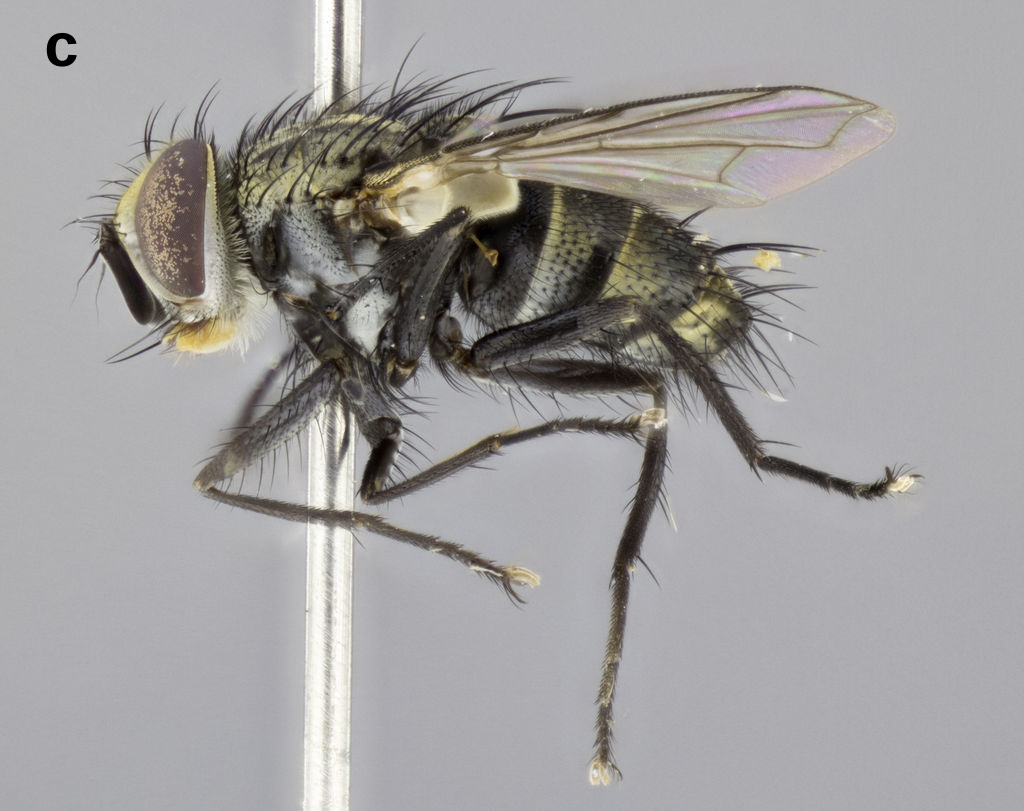
lateral view

**Figure 22d.**
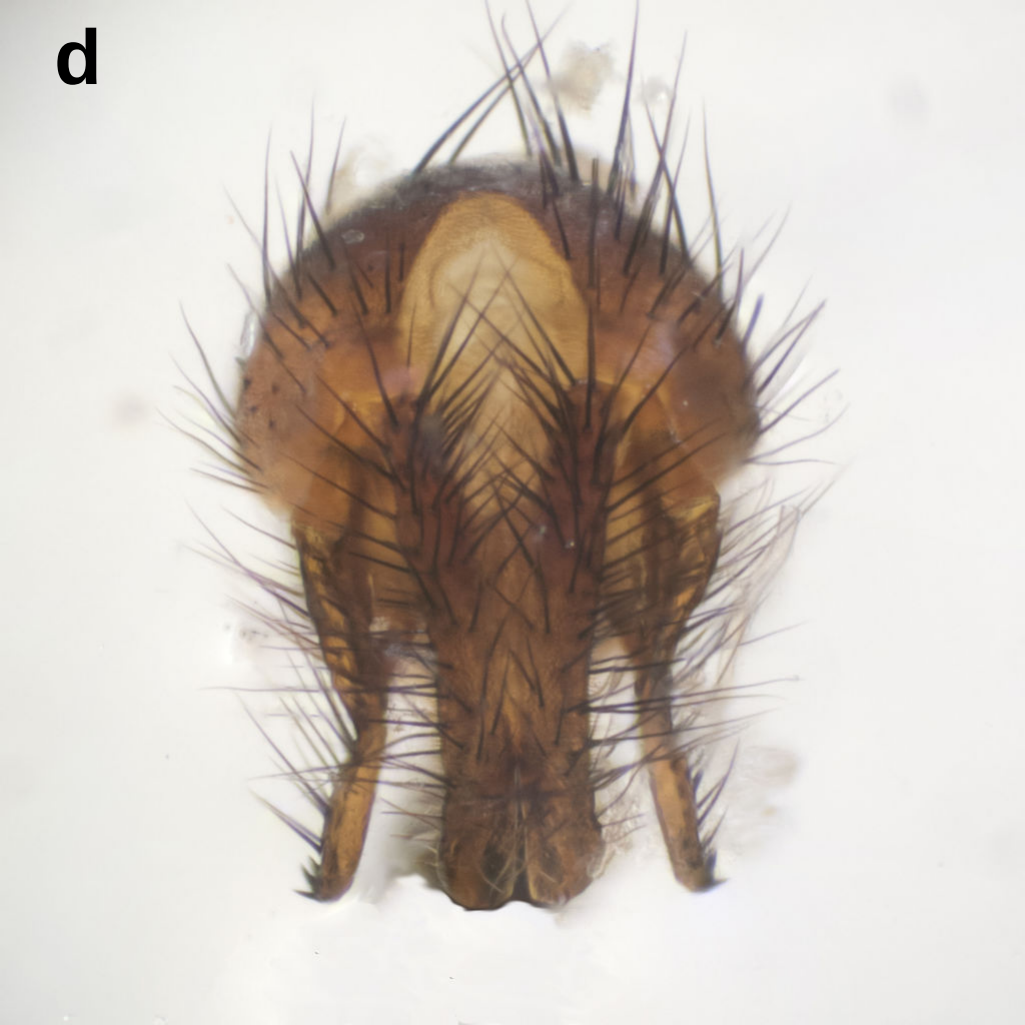
dorsal view

**Figure 22e.**
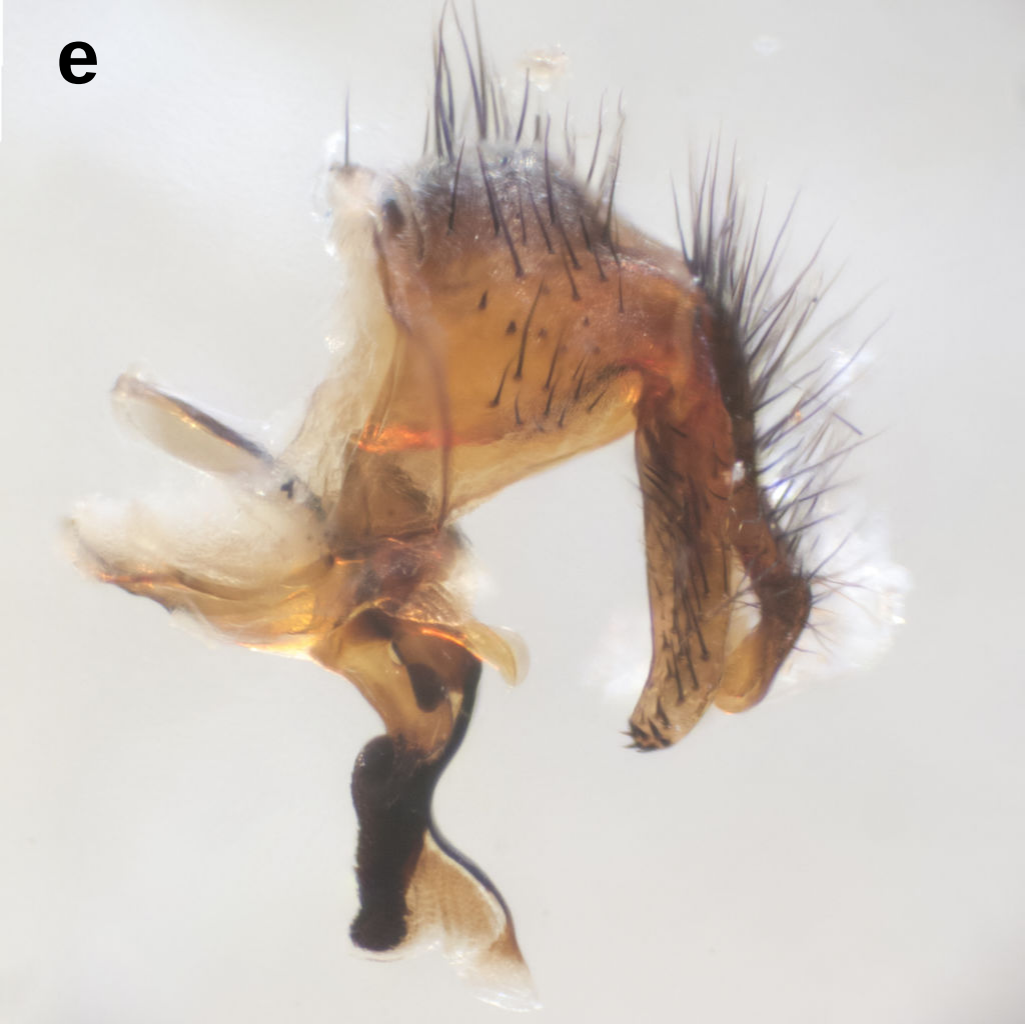
lateral view

**Figure 22f.**
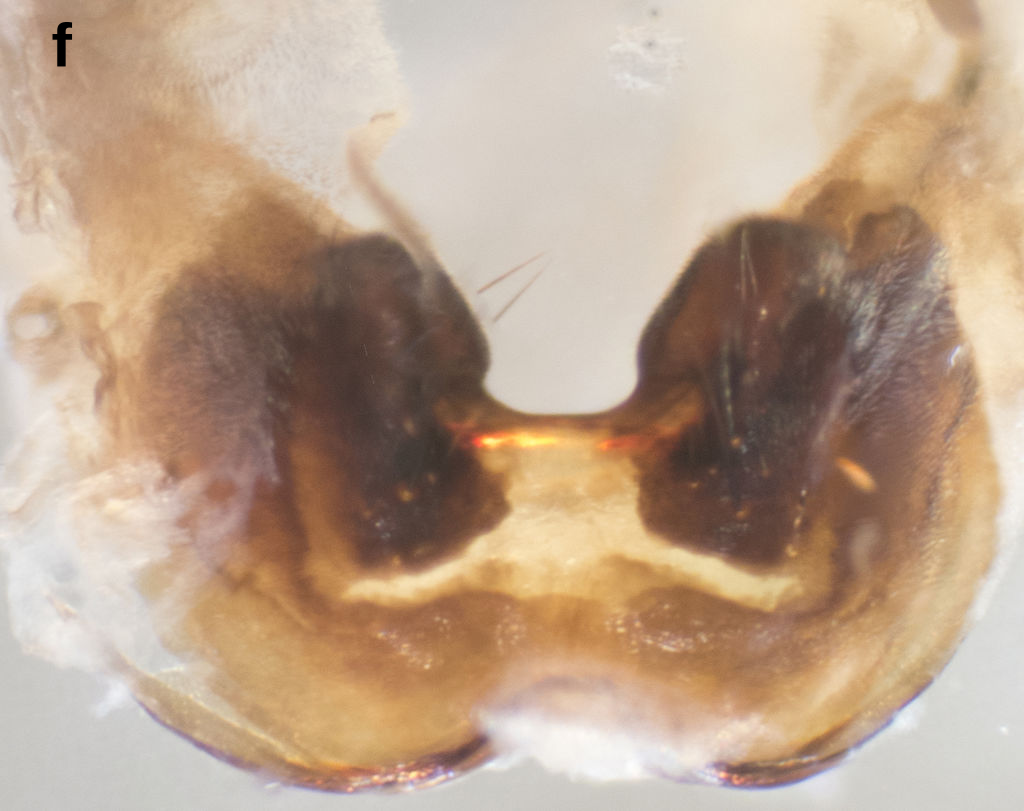
sternite 5, ventral view

**Figure 23a.**
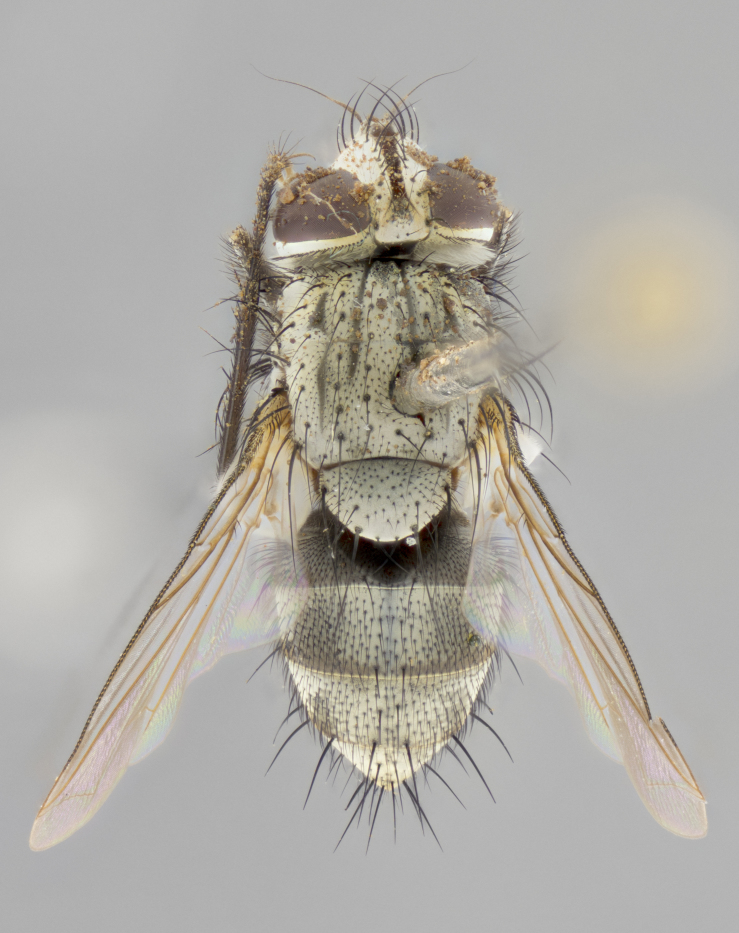
dorsal view

**Figure 23b.**
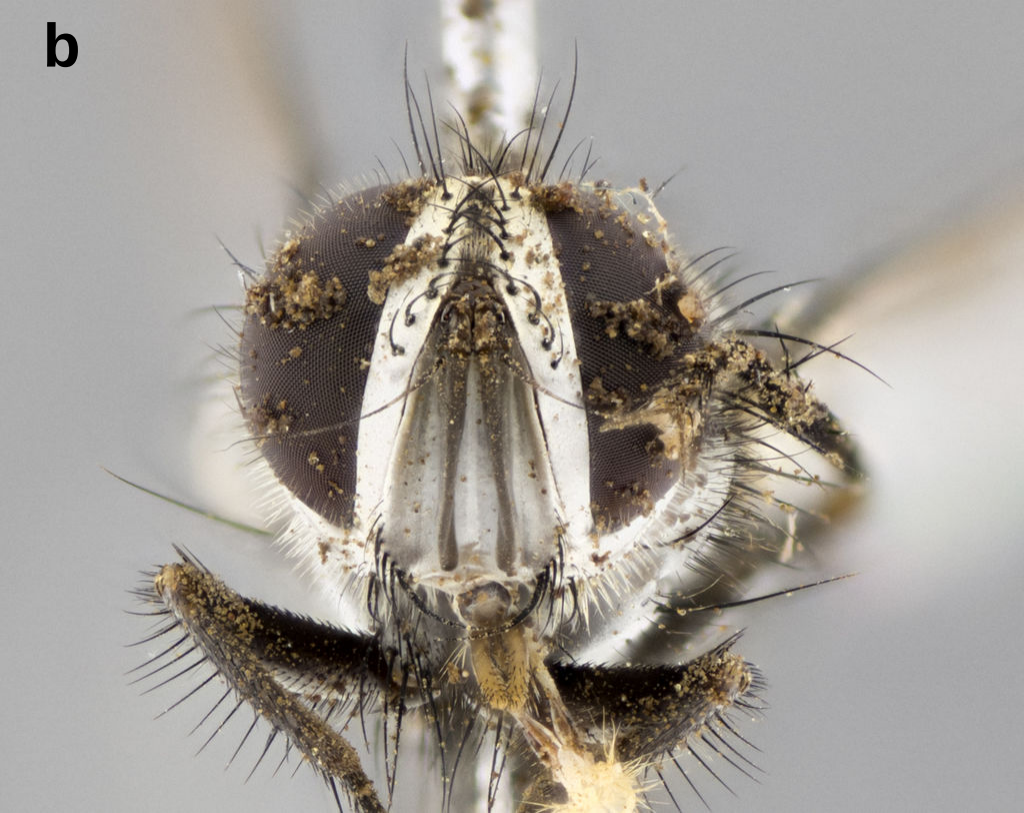
frontal view

**Figure 23c.**
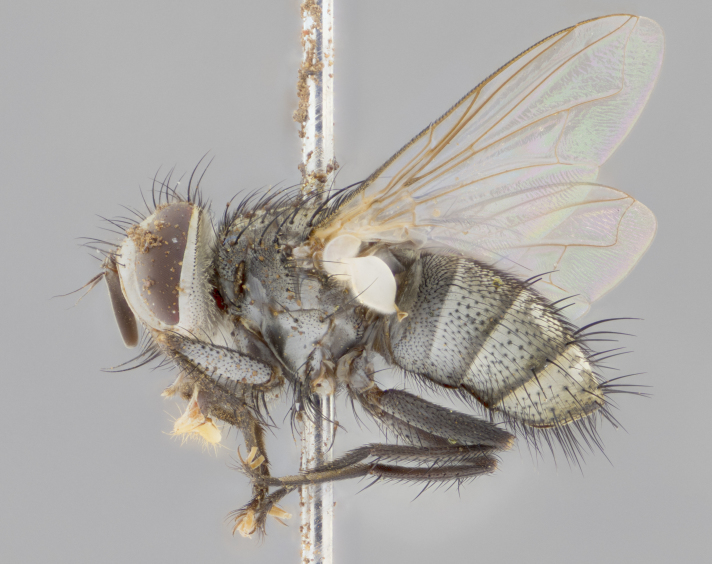
lateral view

**Figure 23d.**
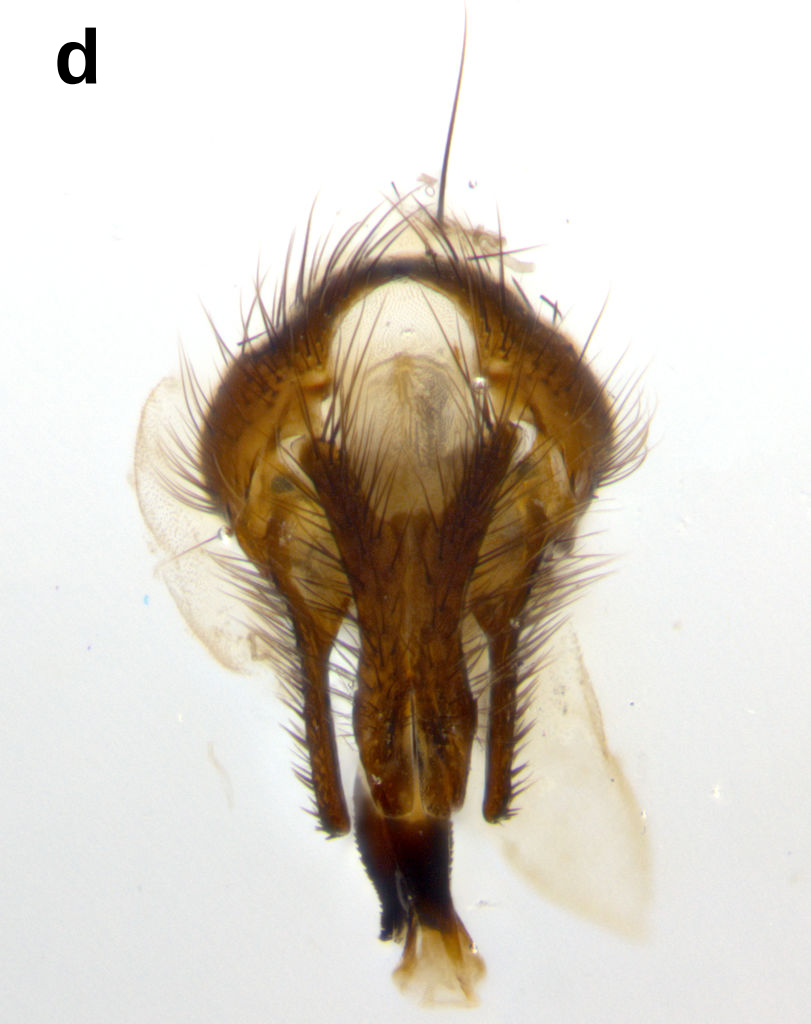
dorsal view

**Figure 23e.**
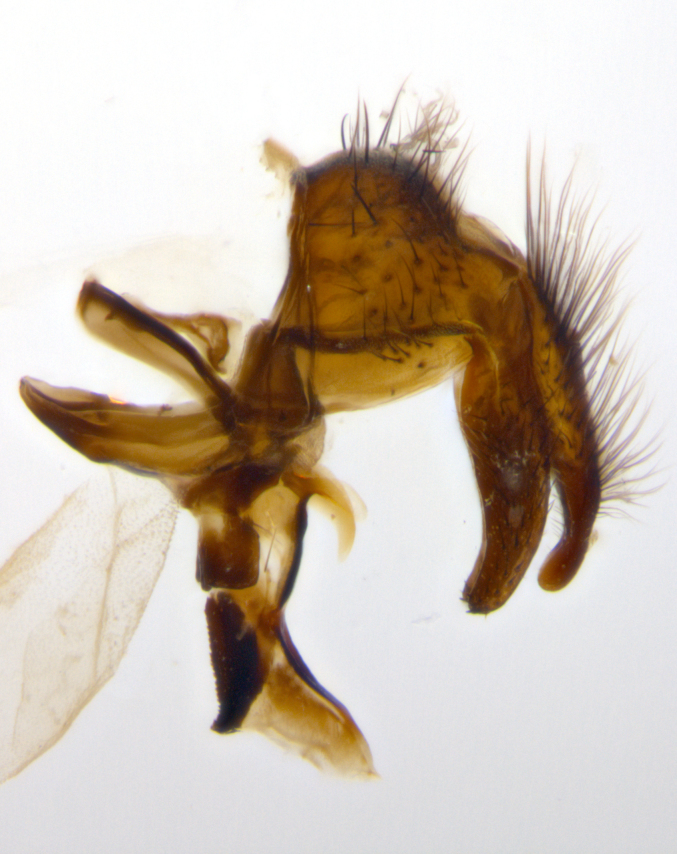
lateral view

**Figure 23f.**
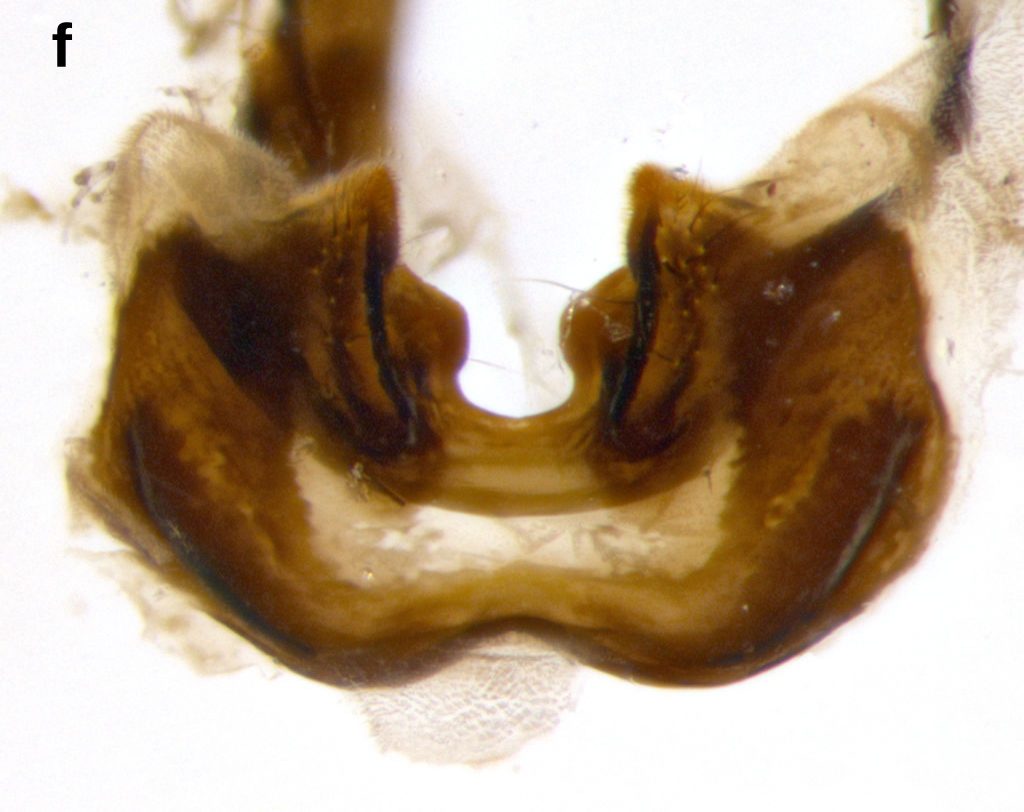
sternite 5, ventral view

**Figure 24a.**
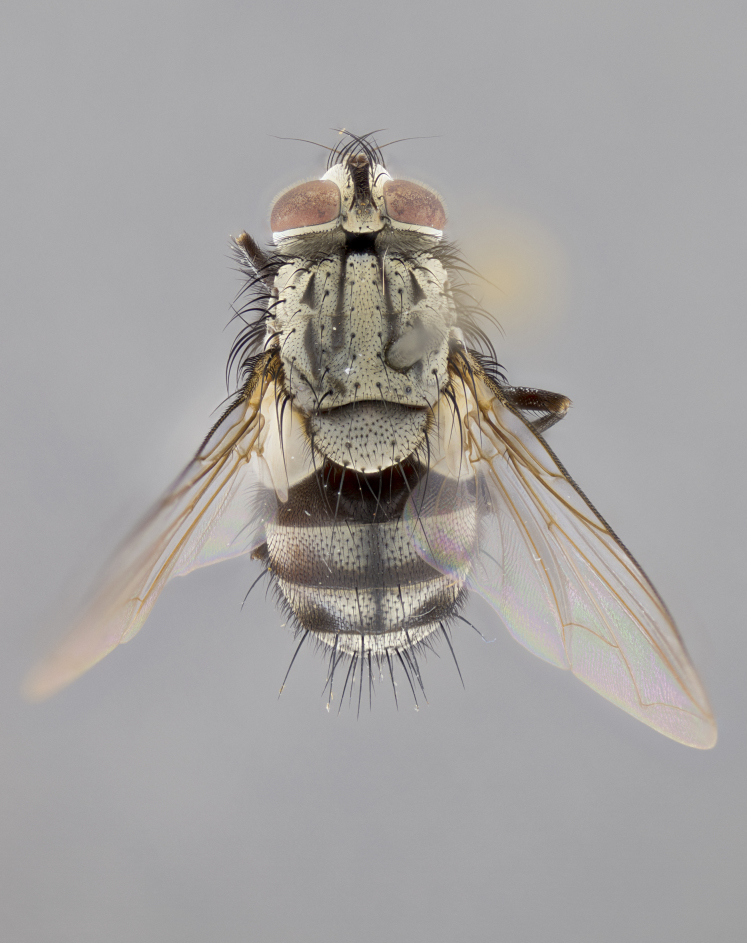
dorsal view

**Figure 24b.**
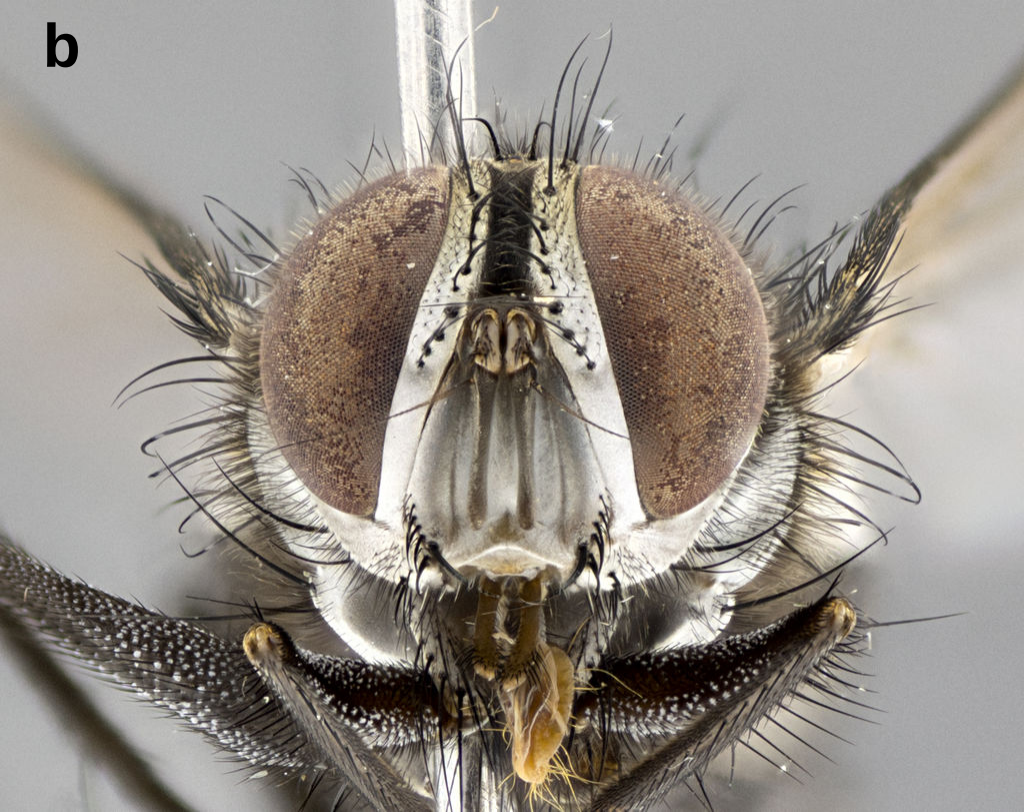
frontal view

**Figure 24c.**
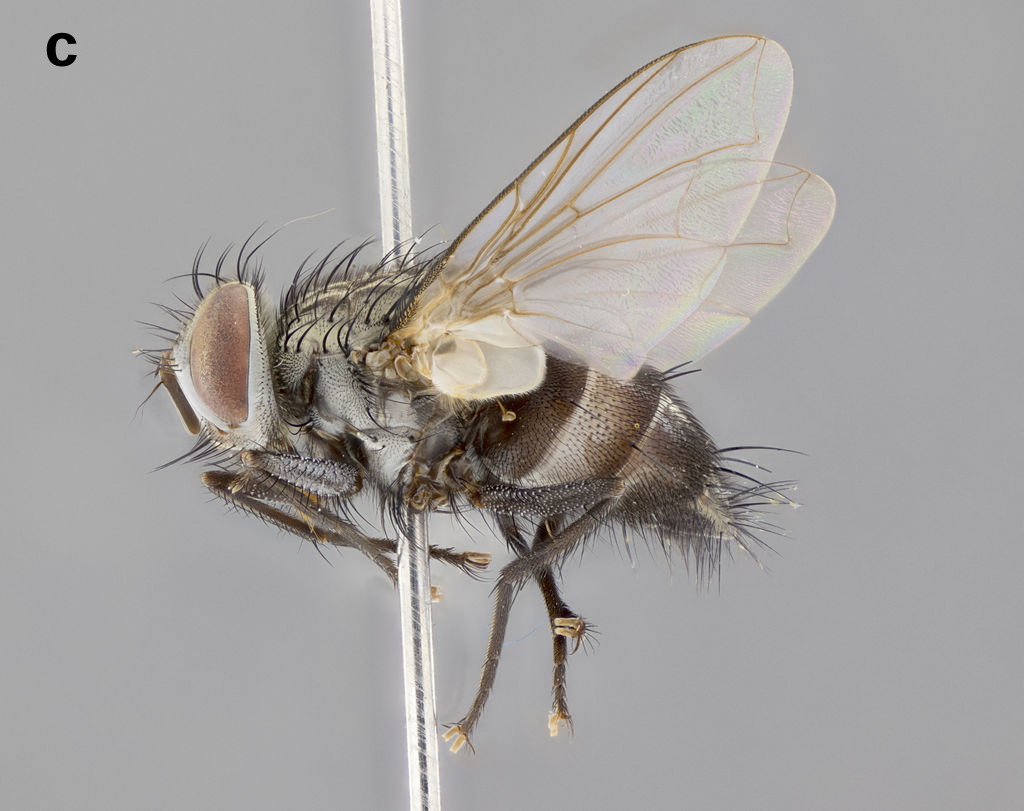
lateral view

**Figure 25a.**
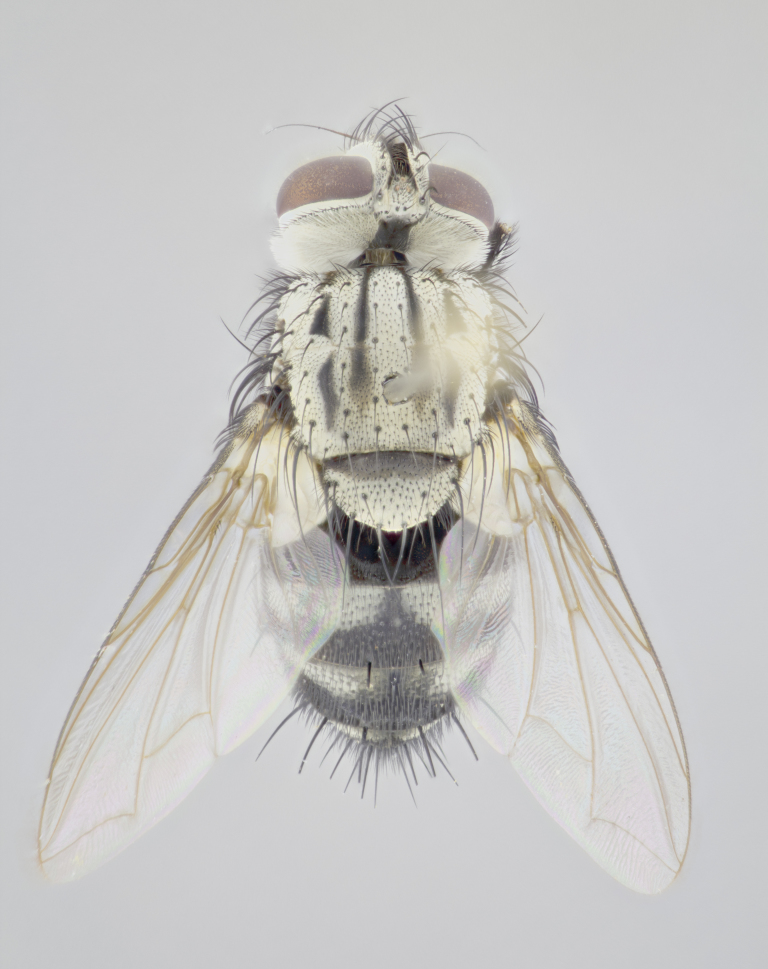
dorsal view

**Figure 25b.**
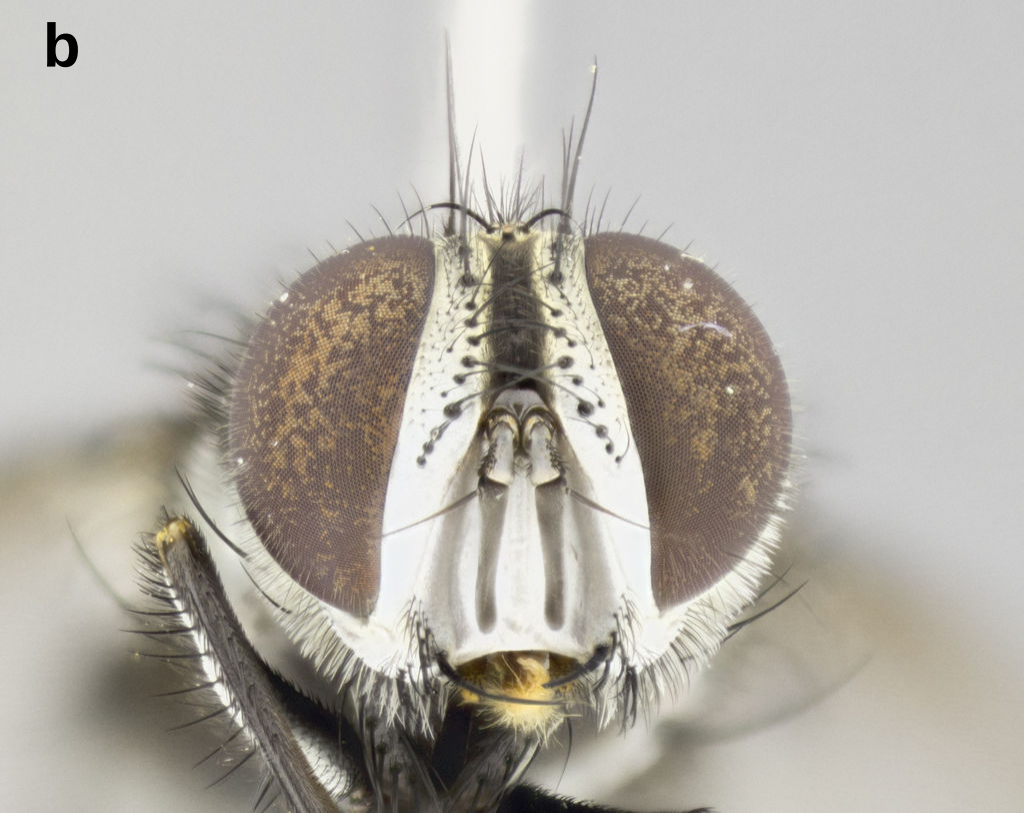
frontal view

**Figure 25c.**
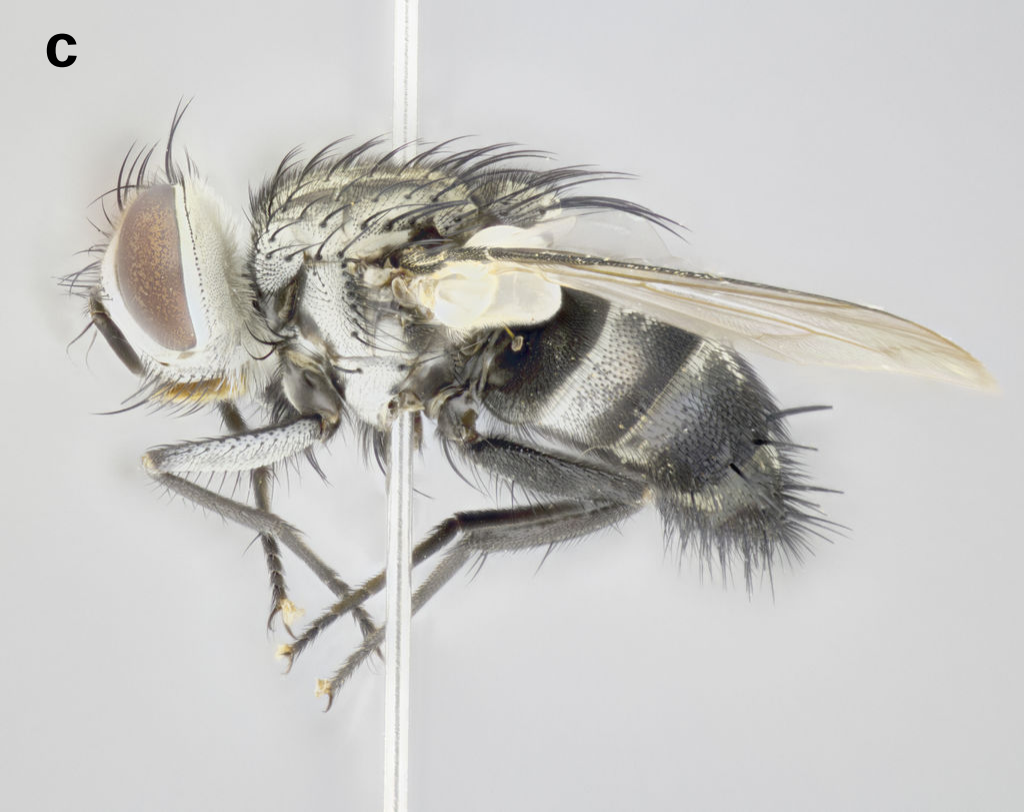
lateral view

**Figure 25d.**
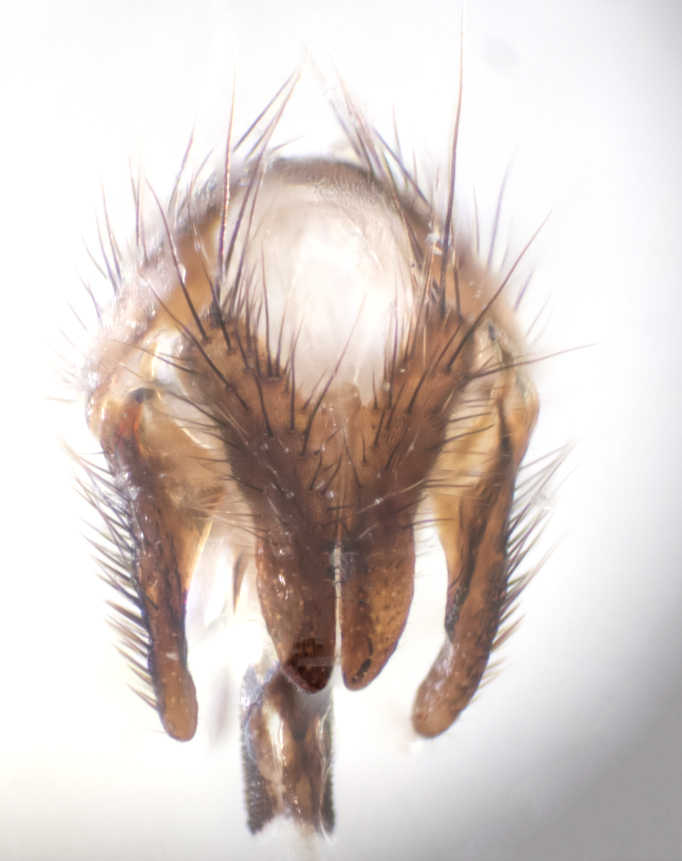
dorsal view

**Figure 25e.**
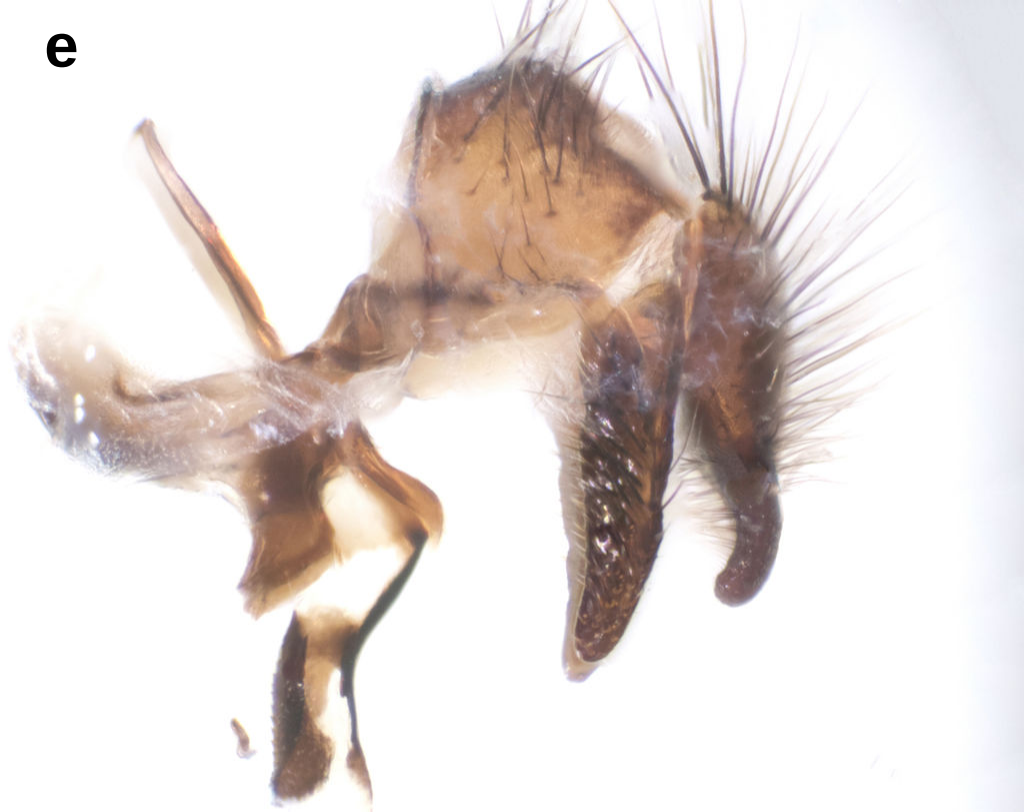
lateral view

**Figure 25f.**
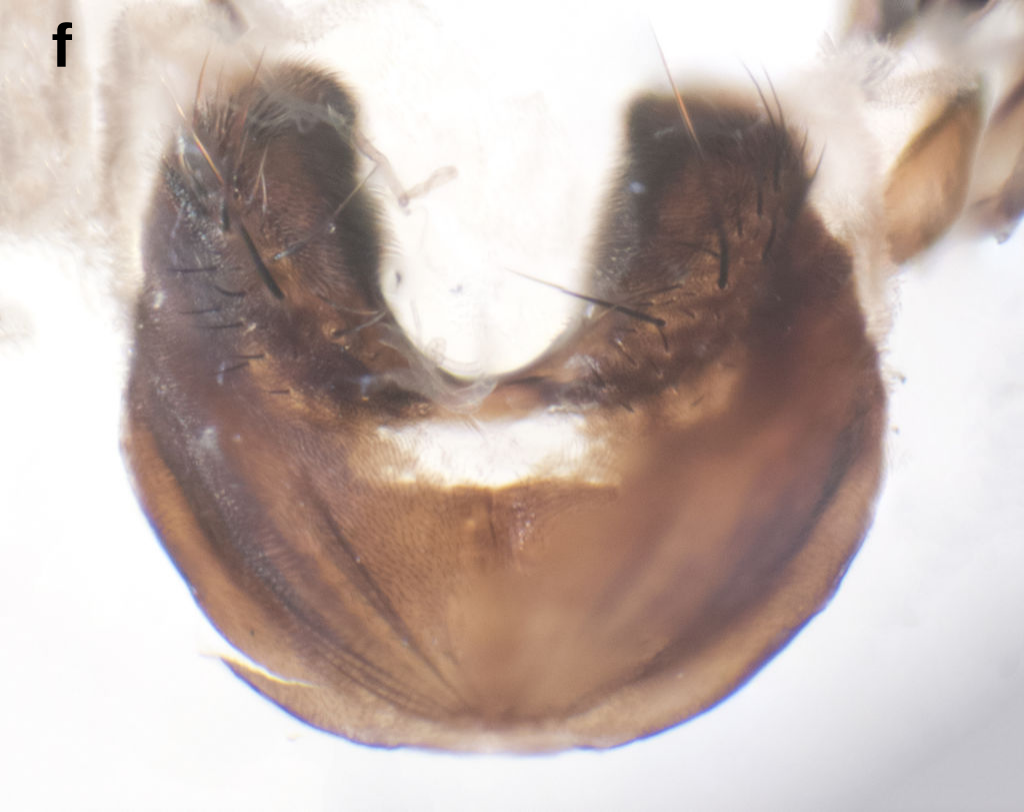
sternite 5, ventral view

**Figure 26a.**
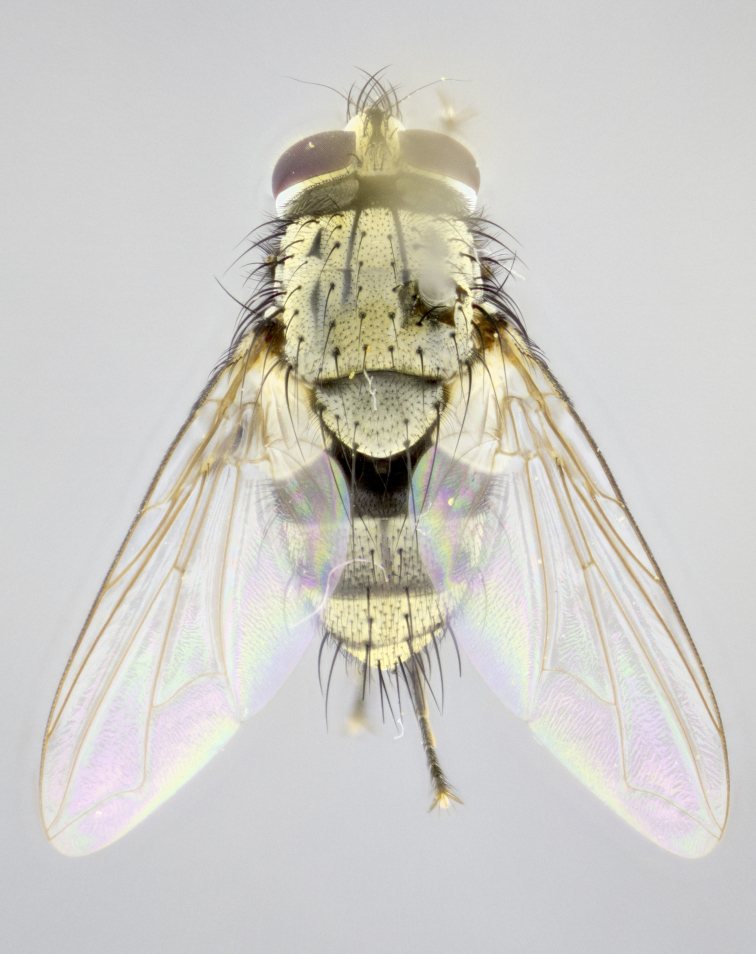
dorsal view

**Figure 26b.**
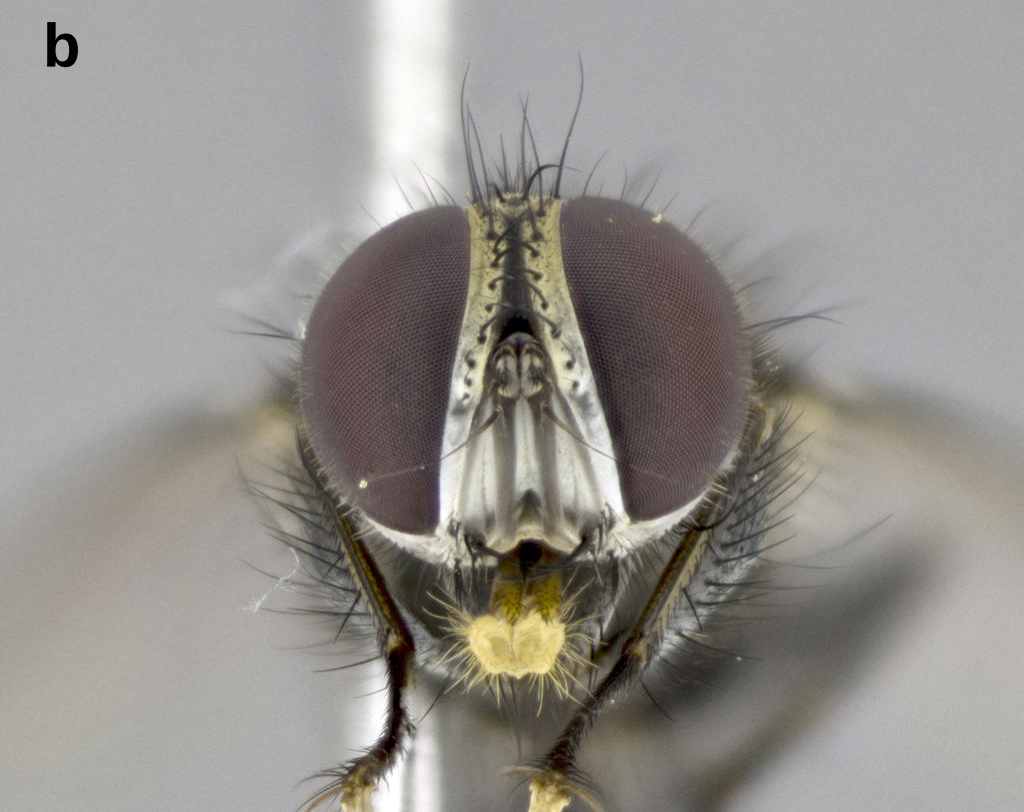
frontal view

**Figure 26c.**
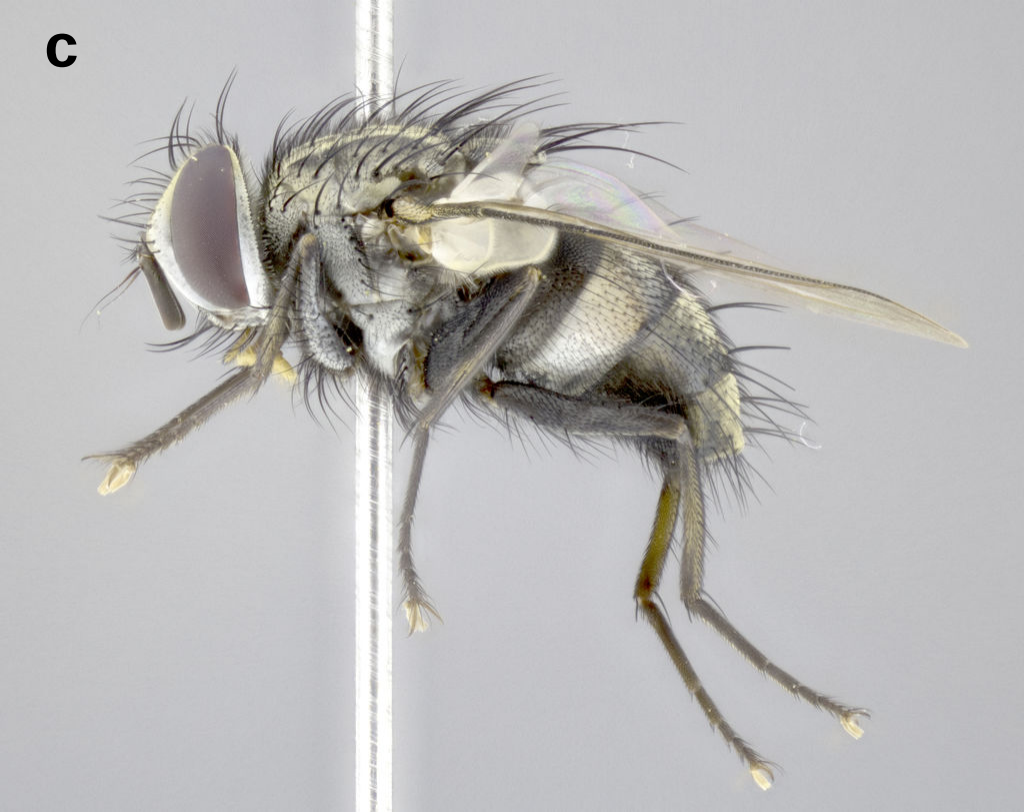
lateral view

**Figure 26d.**
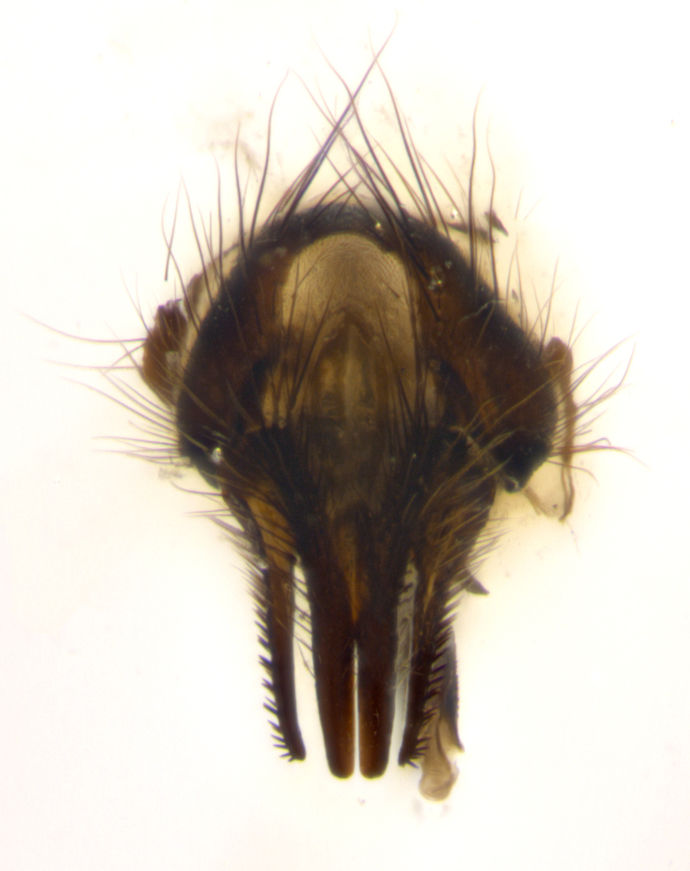
dorsal view

**Figure 26e.**
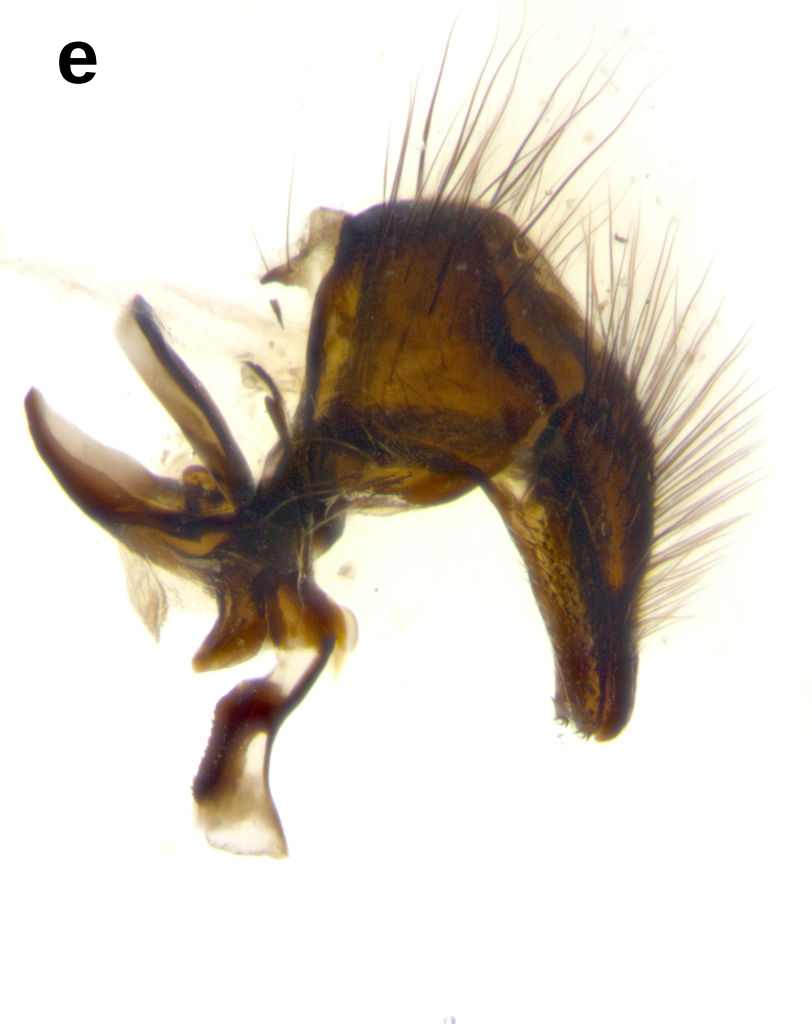
lateral view

**Figure 26f.**
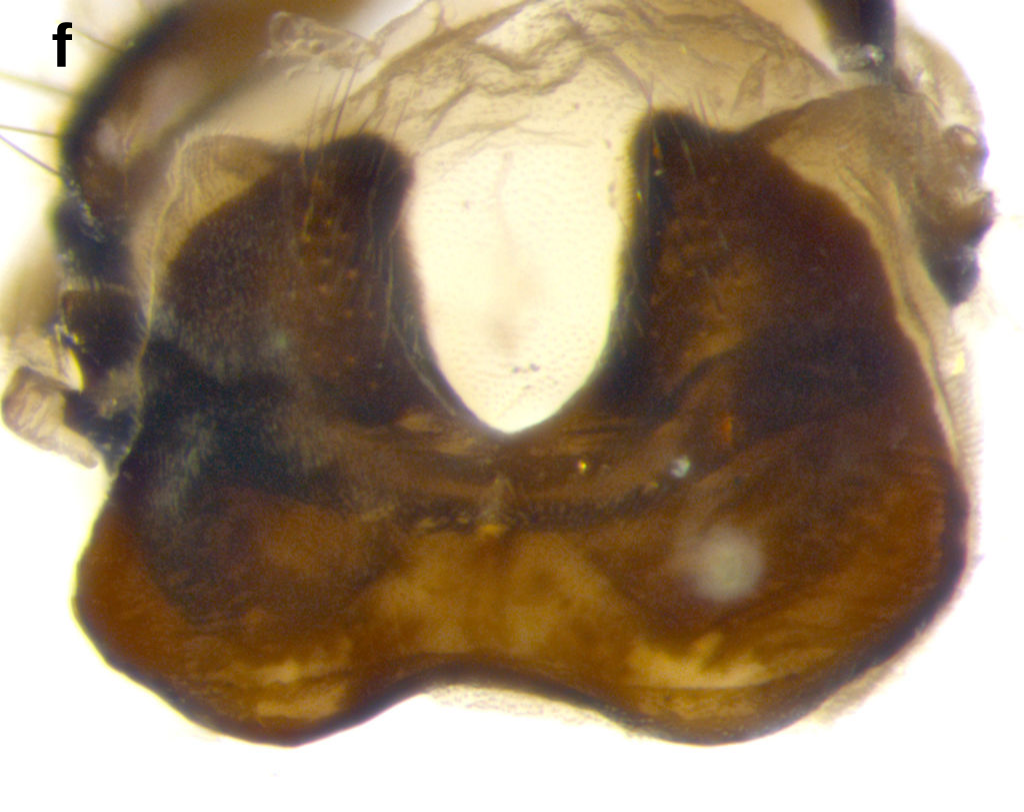
sternite 5, ventral view

**Figure 27a.**
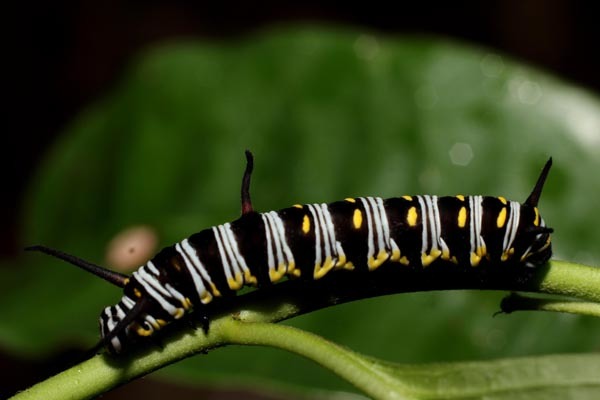
*Danaus gilippus*DHJ02 voucher n. 13-SRNP-10060, feeding on Asclepiadaceae. Image voucher n. DHJ498910.jpg

**Figure 27b.**
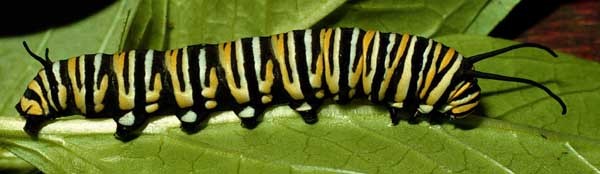
*Danaus plexippus* voucher n. 98-SRNP-7073, feeding on Asclepiadaceae. Image voucher n. DHJ46548.jpg

**Figure 27c.**
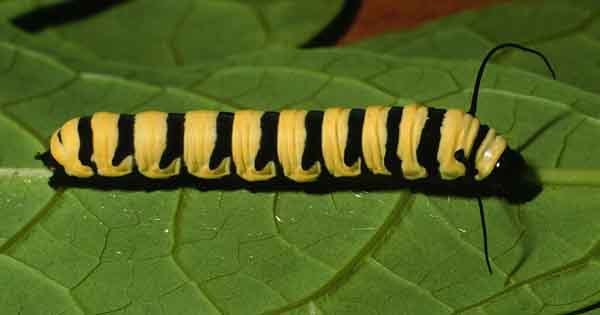
*Lycorea h. atergatis* voucher n. 99-SRNP-5616, feeding on Caricaceae. Image voucher n. DHJ51243.jpg

**Figure 27d.**
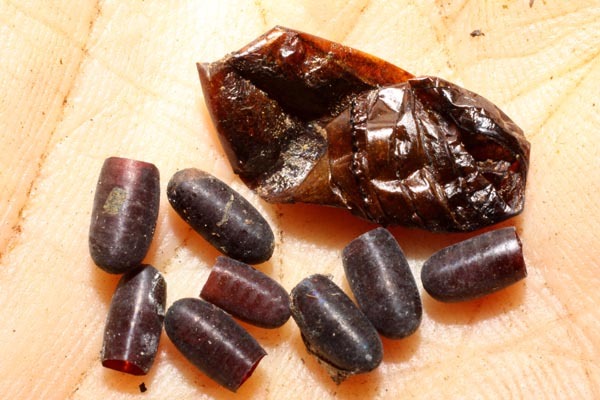
Puparia of *Hyphantrophaga danausophaga* voucher n. DHJPAR0034375, reared from *Danaus plexippus* voucher n. 09-SRNP-35341. Image voucher n. DHJ470083.jpg

**Figure 28a.**
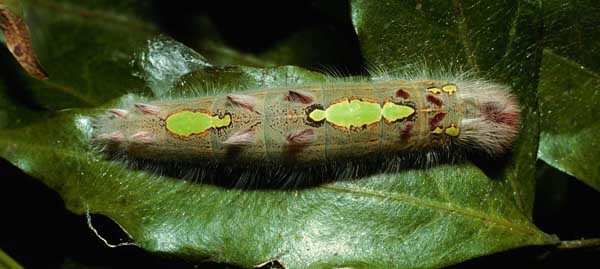
*Morpho p. catalina* voucher n. 02-SRNP-8076, feeding on Fabaceae. Image voucher n. DHJ66311.jpg

**Figure 28b.**
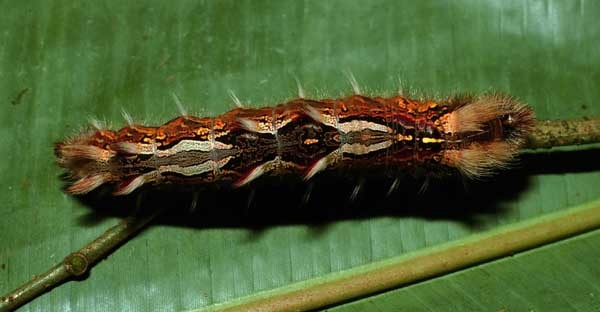
*Morpho helenor* voucher n. 89-SRNP-735a, feeding on Fabaceae. Image voucher n. DHJ11620.jpg

**Figure 28c.**
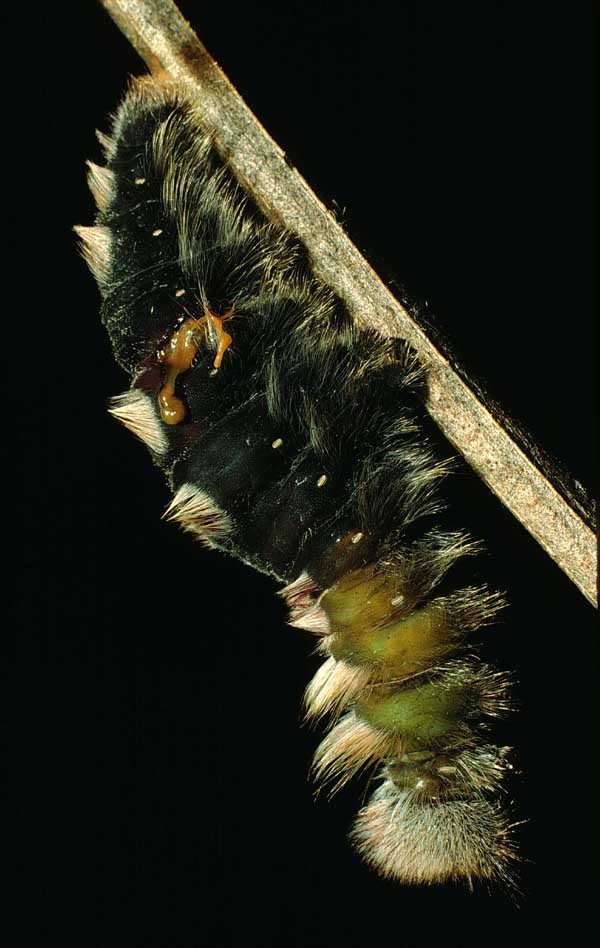
*Morpho p. catalina* voucher n. 95-SRNP-4612, feeding on Fabaceae, parasitized by *Hyphantrophaga morphophaga* voucher n. DHJPAR0007354. Image voucher n. DHJ23257.jpg

**Figure 28d.**
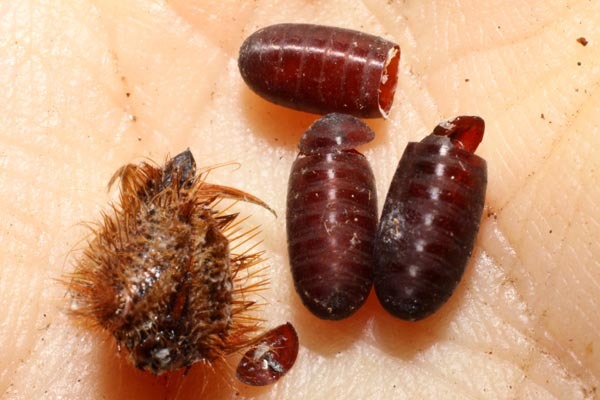
Puparia of *Hyphantrophaga morphophaga* voucher n. DHJPAR0016681, reared from *Morpho helenor* voucher n. 06-SRNP-36610. Image voucher n. DHJ469258.jpg
